# Identification of miRNAs and their targets through high-throughput sequencing and degradome analysis in male and female *Asparagus officinalis*

**DOI:** 10.1186/s12870-016-0770-z

**Published:** 2016-04-12

**Authors:** Jingli Chen, Yi Zheng, Li Qin, Yan Wang, Lifei Chen, Yanjun He, Zhangjun Fei, Gang Lu

**Affiliations:** Key Laboratory of Horticultural Plant Growth, Development and Biotechnology, Agricultural Ministry of China, Department of Horticulture, Zhejiang University, Hangzhou, 310058 PR China; Boyce Thompson Institute for Plant Research, Cornell University, Tower Road, Ithaca, New York 14853 USA; USDA Robert W. Holley Center for Agriculture and Health, Tower Road Ithaca, New York, 14853 USA

**Keywords:** *Asparagus officinalis*, miRNAs, High-throughput sequencing, Degradome analysis, Sex determination

## Abstract

**Background:**

MicroRNAs (miRNAs), a class of non-coding small RNAs (sRNAs), regulate various biological processes. Although miRNAs have been identified and characterized in several plant species, miRNAs in *Asparagus officinalis* have not been reported. As a dioecious plant with homomorphic sex chromosomes, asparagus is regarded as an important model system for studying mechanisms of plant sex determination.

**Results:**

Two independent sRNA libraries from male and female asparagus plants were sequenced with Illumina sequencing, thereby generating 4.13 and 5.88 million final clean reads, respectively. Both libraries predominantly contained 24-nt sRNAs, followed by 21-nt sRNAs. Further analysis identified 154 conserved miRNAs, which belong to 26 families, and 39 novel miRNA candidates seemed to be specific to asparagus. Comparative profiling revealed that 63 miRNAs exhibited significant differential expression between male and female plants, which was confirmed by real-time quantitative PCR analysis. Among them, 37 miRNAs were significantly up-regulated in the female library, whereas the others were preferentially expressed in the male library. Furthermore, 40 target mRNAs representing 44 conserved and seven novel miRNAs were identified in asparagus through high-throughput degradome sequencing. Functional annotation showed that these target mRNAs were involved in a wide range of developmental and metabolic processes.

**Conclusions:**

We identified a large set of conserved and specific miRNAs and compared their expression levels between male and female asparagus plants. Several asparagus miRNAs, which belong to the miR159, miR167, and miR172 families involved in reproductive organ development, were differentially expressed between male and female plants, as well as during flower development. Consistently, several predicted targets of asparagus miRNAs were associated with floral organ development. These findings suggest the potential roles of miRNAs in sex determination and reproductive developmental processes in asparagus.

**Electronic supplementary material:**

The online version of this article (doi:10.1186/s12870-016-0770-z) contains supplementary material, which is available to authorized users.

## Background

MicroRNAs (miRNAs) are a class of endogenous non-coding RNAs with lengths of 20–25 nucleotide (nt) and function as gene expression regulators [1]. To date, 28,645 conserved and species-specific miRNAs from 223 species have been deposited in miRBase 21 (ftp://mirbase.org/pub/mirbase). Plant miRNAs were first reported in *Arabidopsis thaliana* in 2002, and subsequently identified in a large number of plant species. Plant miRNAs originate from single-stranded primary transcripts (pri-miRNAs), which display stem-loop structures, via the cleavage of a short duplex from the stem region by DCL1 [2]. Increasing evidence demonstrates that miRNAs play important roles in multiple biological processes, including growth, development, and stress responses [3–6], by translation inhibition or by cleaving their specific mRNA targets [7]. Extensive studies have been performed to understand the functions of miRNAs in various species during the past decade [3–5]. The rapid advancement of high-throughput sequencing technologies has provided a highly efficient means to explore large miRNA families. These sequencing technologies have been successfully used in various species to identify and characterize a large number of novel miRNAs due to their advantage in detecting novel miRNAs with low copy number [5–7].

Plant miRNAs post-transcriptionally regulate target mRNAs via perfect or nearly perfect complementary base pairing of the miRNA. The miRNAs would cleave their specific targets at the 10 th or 11th complementary base by effector mediated AGO1 protein complex, which directly leads to protein translation inhibition or mRNA cleavage [8]. Selection and annotation of miRNA targets are essential steps to understand the biological function of miRNAs. Prediction of miRNA target genes can be performed using several methods, such as computational target prediction, AGO protein co-immunoprecipitation, and RNA ligase-mediated rapid amplification of cDNA ends (5′ RLM-RACE) [9]. With recent advances on sequencing technologies, degradome analysis combined with high-throughput sequencing and bioinformatics analysis has been proved to be an efficient approach for miRNA target prediction. Degradome sequencing has been successfully applied in *Arabidopsis*, rice, and other plant species [10–12].

Evidence has suggested that miRNAs are involved in several regulatory pathways that control reproductive development in plants. For example, miR156 and miR172 affect flowering time when over-expressed in *Arabidopsis* and maize [13–15]. MiR172 regulates flower development by targeting *APETALA2* (*AP2*) and *AP2* homologs in *Arabidopsis* [16]. Recent studies have reported the potential role of miRNAs in sex determination. In maize, the translation of *IDS1* can be inhibited by *ts4* miRNA (miRNA172), resulting in male florets; by contrast, a loss-of-function mutation in the *ts4* or a mutation in the miRNA-binding site of the *ids1* gene would produce normal IDS1 protein, thus resulting in female florets [17, 18]. MiRNAs are likely to be important in sex determination and differentiation in dioecious species [19]. Nevertheless, to the best of our knowledge, the mechanism through which miRNAs control plant sex determination has not been elucidated. Although numerous sex chromosome-specific miRNAs have been identified in some dioecious species [20], the detailed functions of these miRNAs remain unclear.

Garden asparagus (*Asparagus officinalis* L.) is widely cultivated as a valuable vegetable crop worldwide because of its important nutritional and medicinal value attributed to its abundant amounts of flavonoids, saponins, and several vitamins. Previous works have shown that asparagus exhibits antioxidant, anti-cancer, and immunity promoting properties [21]. Asparagus is a dioecious species that belongs to Liliaceae family. The sex of garden asparagus is determined by its sex chromosomes; the males are heterogametic (XY), whereas the females are homogametic (XX) [22]. Garden asparagus is a diploid species containing 20 chromosomes; of which, the chromosome L5 has been identified as its sex chromosome [23]. Unlike other dioecious plants, such as white campion (*Silene latifolia*) and *Marchantia polymorpha*, asparagus contains homomorphic sex chromosomes. The primitive Y chromosome of asparagus only diverge from their homomorphic X chromosome in a short male-specific and non-recombining region; asparagus is currently regarded as a model plant for studying the evolution of sex chromosomes, considering that its sex chromosomes originated approximately 2 MYA [24]. However, genomic information of asparagus remains limited. Approximately 8,700 EST sequences for asparagus are currently available in the NCBI databases and a transcriptome dataset generated by high-throughput sequencing technology was recently published [25]. To date, information regarding asparagus miRNAs or even the Liliaceae family is insufficient, and only a few miRNAs have been described in detail. In the present study, we constructed two small RNA (sRNA) libraries of male and female asparagus and performed high-throughput Illumina sequencing to identify conserved and asparagus-specific miRNAs. Differentially expressed miRNAs between male and female plants were identified and further verified by real-time quantitative RT-PCR (qRT-PCR). Furthermore, potential targets for all asparagus miRNAs were predicted through degradome sequencing. Gene ontology (GO) analysis indicated that several predicted targets of asparagus miRNAs are associated with organ development, substance metabolism, signal transduction, and stress responses. Interestingly, several miRNAs are known to be involved in plant reproductive organ development; hence, miRNAs exhibit important roles in sex determination and differentiation.

## Results

### Small RNA profiles in *A. officinalis*

Two independent sRNA libraries were generated from the pooled total RNAs from female and male asparagus individuals. These libraries were sequenced with the high-throughput Illumina HiSeq platform to identify miRNAs from asparagus. A total of 9,336,830 and 14,970,830 raw reads were obtained from the female and male RNA libraries, respectively. After trimming the adapter sequences and removing low quality and short sequences (<15 nt long), 6,906,565 and 11,732,973 reads were retained for the female and male flowers, respectively. The sequences belonging to rRNAs, tRNAs, anRNA, and snoRNAs were further filtered according to the Rfam database (11.0). The remaining 4,133,319 and 5,883,039 clean reads, which represent 1,699,714 and 2,343,563 unique reads, were used for miRNA identification from female and male individuals, respectively (Table 1).

**Table 1:** Statistics of high-throughput sequencing reads

Category	Female	Male
Raw reads	9336830	14970830
Adaptor sequence and <15 bp removed	2430265	3237857
Clean reads	6906565	11732973
rRNA removed	2773246	5849934
Final clean reads	4133319	5883039
Unique reads	1699714	2343563

**Fig. 1:**
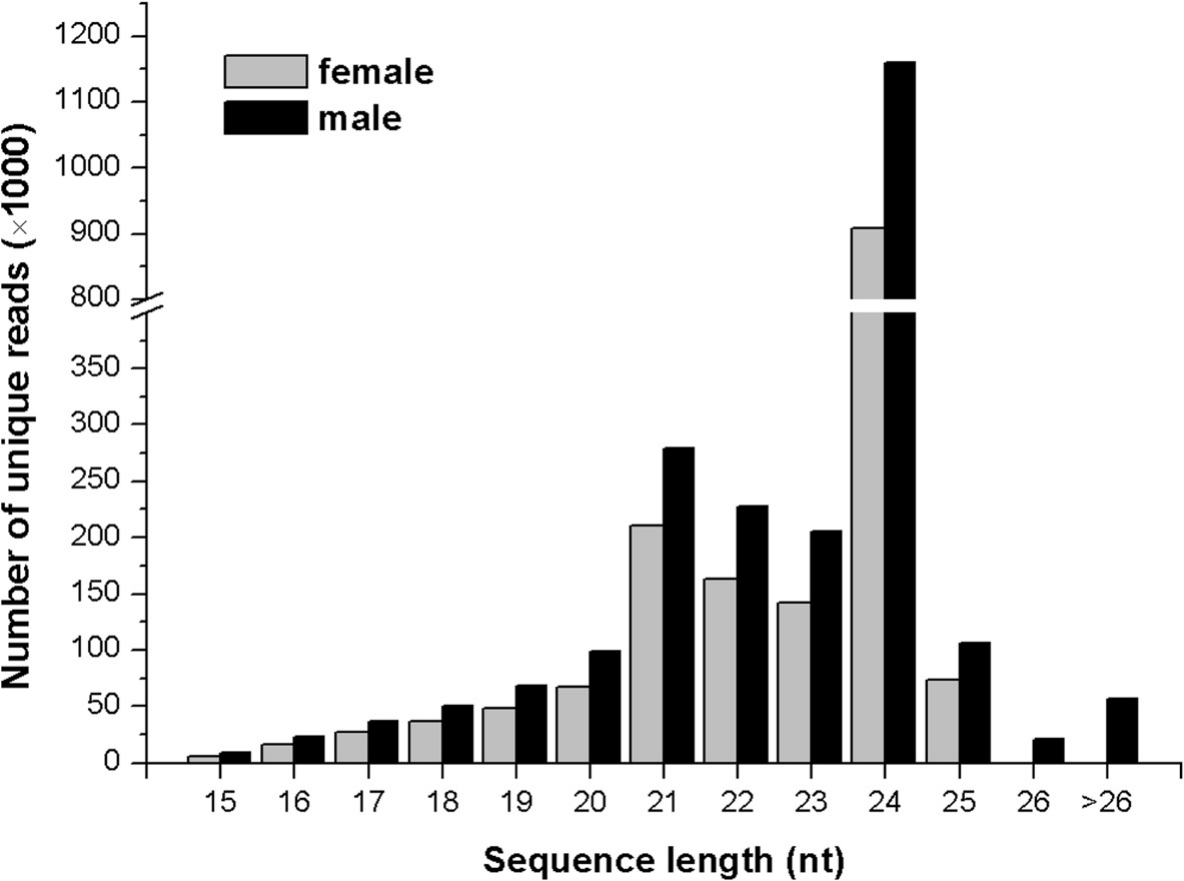
Length distribution of unique sRNAs in male and female libraries of *A. officinalis*. Most of the generated reads were 24 (>30 %) and 21(>19 %) nucleotides long

In both libraries, the majority of the unique sRNA reads were 20–24 nt in length (male, 73.08 %; female, 79.81 %). The most abundant sRNAs in the libraries were 24-nt RNAs, followed by 21-nt RNAs (Fig. 1). The portion of 24-nt sRNAs was approximately 30.84 % and 32.60 % in male and female plants, respectively, and the portion of 21-nt sRNAs was approximately 19.75 % and 25.56 %. These results are consistent with the typical size distribution of sRNAs reported in other plant species, such as *Arabidopsis* [26, 27], *Oryza sativa* [28], *Medicago truncatula* [29], and *Citrus trifoliata* [30]; in these species, 24-nt sRNAs are the most abundant and diverse class of small non-coding RNAs (sncRNAs) sequenced in the sRNA libraries.

### Identification of conserved miRNAs in *A. officinalis*

**Fig. 2:**
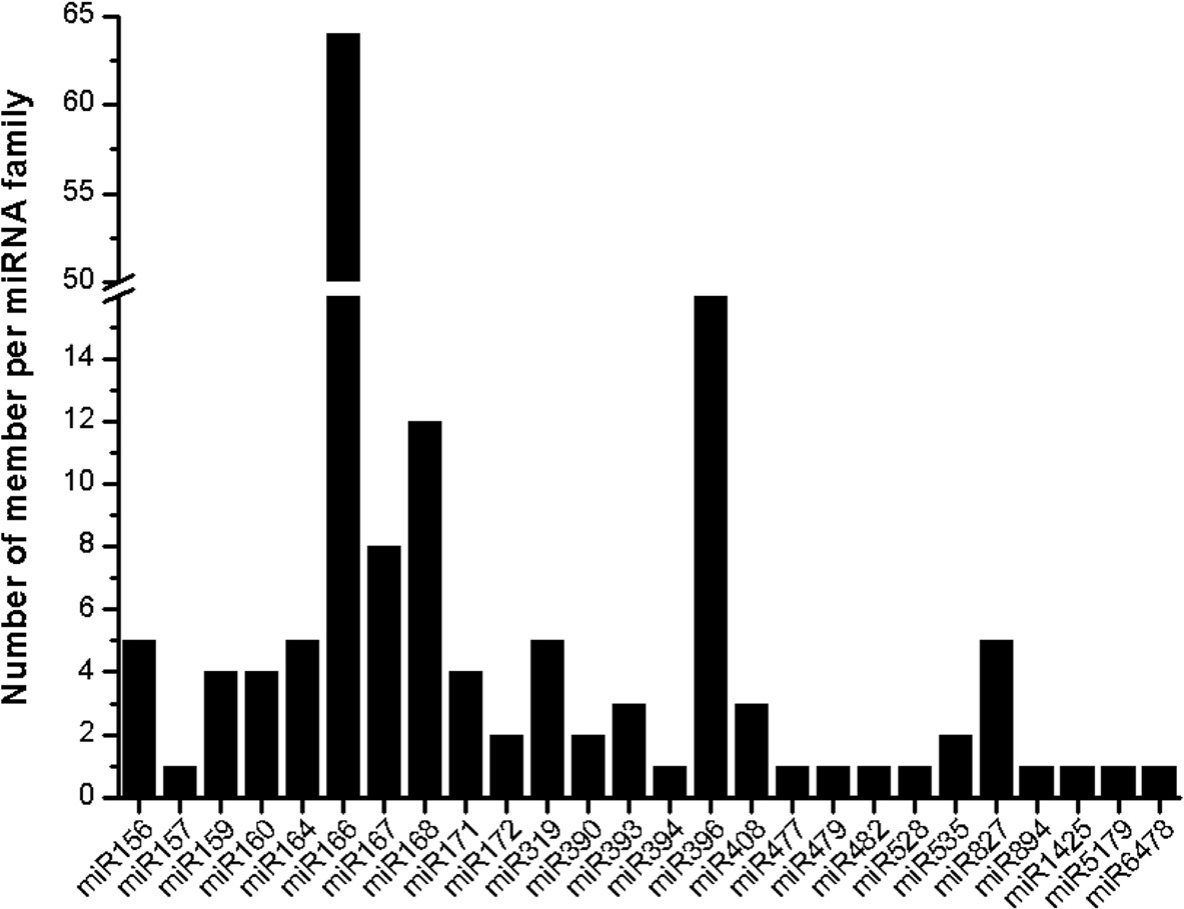
Number of identified miRNAs in each conserved miRNA family in *A. officinalis*

**Fig. 3:**
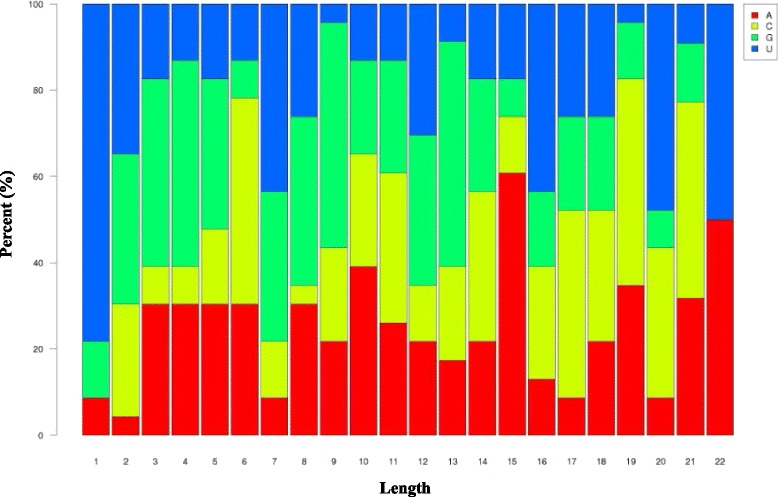
Relative nucleotide bias at each miRNA position compared with the total RNA. Uridine (U) was the most common nucleotide at the 5′ end (>78 %), and the 10^th^ nucleotides, which match to the cleavage site of targets, were mainly adenine (A) (~40 %)

Eligible sRNAs were mapped with miRBase 21 (ftp://mirbase.org/pub/mirbase/21/) to identify conserved miRNAs from all our data sets. After the BLASTN search and further sequence analysis, 154 non-redundant miRNAs were identified to have high sequence similarity to known miRNAs (Additional file 1). These miRNAs could be classified into 26 miRNA families. Among the identified families, the miR166 family contained the largest number of members (63), followed by miR396 and miR168 families, with 16 and 12 members, respectively. By contrast, the miR157, miR394, miR482, miR528, miR894, miR1425, and miR5179 families had only one member each (Fig. 2). Nucleotide sequence analysis of these miRNAs revealed that uridine (U) is the most common nucleotide at the 5′ end (>78 %); the 10th nucleotide matched to the cleavage site of the targets and was mainly adenine (A; ~40 %). However, the majority of the nucleotides at position 11, another common cleavage site, was A or cytosine (C) (Fig. 3).

The majority of the conserved miRNAs were 21-nt in length (67.97 %), thereby representing the most abundant class of miRNAs in plants. The next represented class was the 20-nt miRNAs, which included 23.52 % of all the identified miRNAs (Additional file 1: Table S1). The miRNA length distribution is consistent with previously reported values for other plants [31]. Among the 26 miRNA families, 16 were found to be highly conserved among different plant species; these families include miR396, miR390, miR166, miR171, miR172, and miR159. Specifically, miR166b-3, miR167a, miR171f, and miR396a-5p were highly conserved in 74, 59, 48, and 53 species in miRBase (http://www.mirbase.org/). Some known but less-conserved miRNAs were also found in asparagus. Interestingly, most asparagus miRNAs have been identified mainly in monocot plants. For example, miR1425, miR166k, and miR166e were previously identified in *O. sativa,* whereas miR396a was found in *Zea mays.*

Only aof-miR172a, aof-miR535a, aof-miR535b, aof-miR160b, aof-miR160d, and aof-miR482 miRNAs matched to the respective pre-miRNAs from the asparagus unigenes because of limited asparagus genome information (Additional file 1: Table S2). To further verify the identified miRNAs, we used the genome sequences of *O. sativa*, a model monocot plant, as the reference for the identification of the precursors of the potential miRNAs. Finally, the pre-miRNA sequences of 42 miRNAs were identified based on the *O. sativa* genome sequences (Additional file 1: Table S2).

The relative abundance of asparagus miRNAs were estimated as transcripts per million (TPM). The TPM values drastically varied among 26 miRNA families. Some miRNAs were highly expressed in both male and female plants, which accumulated at more than 1000 TPM. These miRNAs include aof-miR159a, aof-miR164b, aof-miR166d-1, aof-miR166g-3p, aof-miR166h-3p, aof-miR167h, aof-miR396b-2, aof-miR396b-2, and aof-miR535b. However, some miRNAs were expressed at lower levels in asparagus plants (Additional file 1: Table S1). Different conserved miRNAs, even those in the same family, exhibited different expression levels. For example, aof-miR166d-1 miRNA presented the highest abundance level, with 26,780 and 21,203 TPM in the female and male libraries, respectively. However, some miR166 members showed relatively lower expression levels. The lowest levels were observed for aof-miR166a-5, aof-miR166i-9, aof-miR166e-9, and aof-miR166e-2 (Additional file 1: Table S1). These results are consistent with the high-throughput sequencing of sRNAs from radish, Chinese cabbage, and apricot [32, 33].

### Identification of novel candidate miRNAs in asparagus

The remaining sRNA sequences were mapped with asparagus unigenes, and their hairpin structures were predicted to identify novel miRNAs in asparagus. Based on the annotation criteria for novel miRNAs [34], 39 candidate miRNAs with lengths between 20 nt to 24 nt were identified; of which, 38.5 % were 21-nt long (Table 2). The length of the novel miRNA precursors ranged from 66 nt to 220 nt, with an average length of 161 nt (Additional file 1: Table S3). The minimum free energy (MFE) for the hairpin structures of these miRNA precursors was lower than −18 kcal/mol. Moreover, the minimal folding free energy index (MFEI) of these candidates ranged from 0.7 to 1.5, with an average value of 1.22, which is higher than that of other RNA types, such as tRNAs (0.64), rRNAs (0.59), and mRNAs (0.62–0.66) [35]. These results suggest that the secondary structures of these novel miRNAs are stable.

**Table 2:** Novel miRNAs identified from *A. officinalis*

miRNA-name	Sequence	Length	GC%	Expression (TPM)		Folding energy	MFEI	Unigene			
				Female	Male			Unigene	Start	End	Strand
aof-miRn01	AAAUUCCAGACGGUCGGCGGGC	22	63.2	11.43	11.92	–193.90	1.53	UN09382	28	210	+
						–212.60	1.44	UN07381	24	233	+
aof-miRn02	AAAUUCCAGACGGUCGGCGGGCU	23	60.9	10.29	12.24	–213.30	1.44	UN07381	23	234	+
aof-miRn03	AAUAGAUGAGAUGAGAUGAGUUGU	24	33.4	5.03	7.06	–87.94	2.00	UN00358	39	209	+
aof-miRn04	AAUUCCAGACGGUCGGCGGGC	21	66.7	10.29	14.12	–212.60	1.44	UN07381	24	233	+
						–193.90	1.53	UN09382	28	210	+
aof-miRn05	AAUUCCAGACGGUCGGCGGGCU	22	63.6	11.89	13.49	–213.30	1.44	UN07381	23	234	+
aof-miRn06	AGACGGUCGGCGGGCUGAAU	20	65.0	20.12	28.39	–215.50	1.44	UN07381	19	238	+
aof-miRn07	AGACGGUCGGCGGGCUGAAUC	21	66.7	24.46	33.10	–215.50	1.43	UN07381	19	239	+
aof-miRn08	AGCGGGGUGUUCUGAUCCAUA	21	52.4	5.03	1.88	–33.40	0.90	UN45561	155	243	+
aof-miRn09	AGCGGGGUGUUCUGAUCCAUACAA	24	50.0	9.83	3.29	–33.40	0.90	UN45561	155	243	+
aof-miRn10	AUGCGAGCGGGGUGUUCUGAUCCA	24	58.3	18.98	13.49	–42.70	1.02	UN45561	150	248	+
aof-miRn11	AUUCCAGACGGUCGGCGGGC	20	70.0	13.95	10.67	–193.90	1.53	UN09382	28	210	+
						–212.60	1.44	UN07381	24	233	+
aof-miRn12	AUUCCAGACGGUCGGCGGGCU	21	66.7	14.86	9.73	–213.30	1.44	UN07381	23	234	+
aof-miRn13	CAGACGGUCGGCGGGCUGAA	20	70.0	9.15	12.39	–215.20	1.43	UN07381	19	237	+
aof-miRn14	CAGACGGUCGGCGGGCUGAAU	21	66.7	23.32	31.69	–215.50	1.44	UN07381	19	238	+
aof-miRn15	CAGACGGUCGGCGGGCUGAAUC	22	68.2	25.15	40.94	–215.70	1.42	UN07381	18	239	+
aof-miRn16	CCAGACGGUCGGCGGGCUGA	20	75.0	13.49	16.00	–215.20	1.43	UN07381	20	236	+
aof-miRn17	CCAGACGGUCGGCGGGCUGAA	21	71.4	4.57	9.10	–215.20	1.43	UN07381	19	237	+
aof-miRn18	CCAGACGGUCGGCGGGCUGAAU	22	68.2	18.29	17.88	–215.70	1.44	UN07381	18	238	+
aof-miRn19	CCAGACGGUCGGCGGGCUGAAUC	23	69.6	22.64	33.26	–215.70	1.43	UN07381	18	239	+
aof-miRn20	CCUGGUUCCCUGUAUGCCACC	21	61.9	27.44	18.51	–45.50	0.99	UN21911	249	331	+
aof-miRn21	CGAAAUUCCAGACGGUCGGCGGGC	24	66.7	8.69	8.16	–193.90	1.53	UN09382	28	210	+
						–212.60	1.44	UN07381	24	233	+
aof-miRn22	CGAACCCUGGUCGAUUGUUUU	21	47.6	10.29	12.71	–52.44	0.65	UN41527	26	234	+
aof-miRn23	CGAACCCUGGUCGAUUGUUUUU	22	45.5	2.52	6.43	–53.04	0.65	UN41527	25	235	+
aof-miRn24	CGAUUGUUUUUGGGAUGCGCU	21	47.6	4.34	5.65	–32.70	0.62	UN21248	7	116	+
aof-miRn25	CUGGUUCCCUGUAUGCCACCC	21	61.9	25.38	17.26	–45.50	1.03	UN21911	250	330	+
aof-miRn25*	GCGUGCAUGGAACCAAGCAUG	21	52.2	5.11	2.56	–93.80	1.00	UN21911	250	330	+
aof-miRn26	GAAAUUCCAGACGGUCGGCGGGC	23	65.2	7.77	6.27	–193.90	1.53	UN09382	28	210	+
						–212.60	1.44	UN07381	24	233	+
aof-miRn27	GAAAUUCCAGACGGUCGGCGGGCU	24	62.5	6.63	5.80	–213.30	1.44	UN07381	23	234	+
aof-miRn28	GUGCCUGGUUCCCUGUAUGCC	21	61.9	730.06	744.81	–50.40	1.03	UN21911	246	334	+
aof-miRn29	GUGCUUCCCCUCGUUGUCACC	21	61.9	0.00	10.04	–43.60	0.78	UN05913	103	192	+
aof-miRn30	UAAAUAGUCGGGGUUGCCAACC	22	50.0	3.89	9.57	–43.20	1.20	UN31232	131	39	-
aof-miRn31	UAAAUAGUCGGGGUUGGCAACC	22	50.0	5.94	6.27	–43.20	1.14	UN42854	153	60	-
						–68.90	1.50	UN31232	27	143	+
aof-miRn32	UGAUUAUGUAGUGGUCCCUCC	21	47.6	7.77	9.88	–63.94	0.98	UN22735	161	338	+
aof-miRn33	UGGCGUGCAUGGAAUCAAGCA	21	52.4	13.72	5.80	–49.10	1.02	UN21911	247	333	+
aof-miRn34	UGGUCGAUUGUUUUUGGGAUG	21	42.9	26.75	21.49	–30.90	0.63	UN21248	10	112	+
aof-miRn35	UGGUCGAUUGUUUUUGGGAUGC	22	45.5	4.57	6.12	–31.40	0.63	UN21248	9	113	+
aof-miRn36	UGUGAUUAUGUAGUGGUCCCUCC	23	47.8	8.46	6.12	–63.94	0.98	UN22735	161	338	+
aof-miRn37	UGUGAUUAUGUAGUGGUCCCUCCA	24	45.8	5.72	5.96	–65.64	1.01	UN22735	160	339	+
aof-miRn38	UUGCCUACUCCGCCCAUUCCCC	22	63.6	251.74	222.75	–45.10	1.13	UN22494	52	141	+
aof-miRn39	UUUCCAAUGCCUCCCAUUCCGG	22	54.5	109.06	102.91	–23.70	0.91	UN16232	530	464	-
						–27	0.96	UN17918	30	96	+

*TPM* transcripts per million, *MFEI* minimal folding free energy index of the hairpin structures

^*^ indicated miRNA star (miRNA^*^)

The abundance of miRNAs was significantly different among the identified novel miRNAs. Sequencing data showed that aof-miRn28, aof-miRn38, and aof-miRn39 miRNAs had relatively higher abundance in both male and female libraries; other family members demonstrated lower abundance of reads, and aof-miRn29 was not found in the female library. Similar to previous studies [30, 31, 36], the newly identified miRNAs generally showed lower abundance levels than the conserved miRNAs. These novel species-specific miRNAs are considered to be young miRNAs that arose recently through evolution.

The majority of these identified novel miRNAs were generated from one locus, whereas seven novel miRNAs including aof-miRn1, aof-miRn04, aof-miRn11, aof-miRn21, aof-miRn26, aof-miRn31 and aof-miRn39 had more than one pre-miRNAs (Additional file 1: Table S3). On the other hand, some unigenes could be bidirectionally transcribed. For example, the unigene UN31232 produced aof-miRn31, whereas its antisense transcript was predicted to generate another miRNA, namely, aof-miRn30. Similar findings were reported in other plants, such as soybean [37], switchgrass [38], and *Panax ginseng* [39]. Furthermore, in the present study, miRNAs could be located in either the 5′-arm or 3′-arm of the stem-loop precursor. For the unigene UN42854, aof-miRn31 was located in the 3′-arm; conversely, this miRNA was also located in the 5′-arm of UN31232. Moreover, UN16232 and UN17918 are the precursors of aof-miRn31, and originate from the 5′-arm and 3′-arm, respectively. Interestingly, among novel candidates, 18 miRNAs were predicted to be generated from UN07381. Therefore, this unigene may be required to transcribe several miRNAs in asparagus.

### Differentially expressed miRNAs between male and female plants

The normalized expression levels of miRNAs were compared between male and female plants to identify sex-biased miRNAs. MiRNAs with more than 2-fold changes in their expression levels and adjusted *p* < 0.05 are presented in Table 3. The results showed that 56 conserved miRNAs and seven novel candidate miRNAs were differentially expressed between male and female RNA libraries. Among them, 37 miRNAs were significantly up-regulated in the female library, whereas the other 26 miRNAs were preferentially expressed in the male library. Notably, aof-miRn29 was only expressed in male plants, thereby indicating that this novel miRNA may have a specific role in male flower organ development in asparagus.

**Table 3:** Differentially expressed miRNAs between asparagus male and female plants

miRNA-name	Sequence	Length	Family	Expression (TPM)		Female/male	Log_2_ (fold change)
				Female	Male		
aof-miR1425-5p	UAGGAUUCAAUCCUUGCUGCU	21	miR1425	1.37	6.27	0.219	–2.1910
aof-miR156k	UGACAGAAGAGAGAGAGCAC	20	miR156	59.22	2.51	23.594	4.5603
aof-miR156a	UUGACAGAAGAGAGUGAGCAC	21	miR156	0.23	7.69	0.030	–5.0589
aof-miR159b	UUUGGAUUGAAGGGAGCUCUG	21	miR159	4.80	52.86	0.091	–3.4580
aof-miR160c	GCGUGCGAGGAGCCAAGCAUA	21	miR160	5.72	0.78	7.333	2.8744
aof-miR160b	UGCCUGGUUCCCUGUAUGCC	20	miR160	11.89	5.96	1.943	0.9583
aof-miR160d	UGCCUGGUUCCCUGUAUGCCA	21	miR160	114.55	51.14	2.240	1.1635
aof-miR160a	UGCCUGGCUCCCUGUAUGCCA	21	miR160	21.95	8.47	2.591	1.3735
aof-miR164c	GGAGAAGCAGGGCACGUGCA	20	miR164	13.72	6.75	2.033	1.0236
aof-miR164a	UGGAGAAGCAGGACACGUGC	20	miR164	5.03	2.35	2.140	1.0976
aof-miR164e	UGGAGAAGCAGGACACGUGCA	21	miR164	48.70	11.29	4.314	2.1090
aof-miR166a-2	GGAAUGUUGUCUGGCUCGUG	20	miR166	6.40	1.25	5.120	2.3561
aof-miR166b-1	GGAAUGUUGUCUGGCUCGUGG	21	miR166	83.00	35.14	2.362	1.2400
aof-miR166e-2	UCCGACCAGGCUUCAUUCCCC	21	miR166	5.72	0.63	9.079	3.1825
aof-miR166e-4	UCGCACCAGGCUUCAUUCCCC	21	miR166	5.49	1.88	2.920	1.5460
aof-miR166e-6	UCGGACCACGCUUCAUUCCCC	21	miR166	6.86	2.98	2.302	1.2029
aof-miR166e-7	UCGGACCAGACUUCAUUCCCC	21	miR166	10.75	28.55	0.377	–1.4074
aof-miR166e-8	UCGGACCAGCCUUCAUUCCCC	21	miR166	8.00	21.80	0.367	–1.4461
aof-miR166e-9	UCGGACCAGCCUUCAUUCCUC	21	miR166	0.91	5.02	0.181	–2.4659
aof-miR166e-10	UCGGACCAGGCCUCAUUCCCC	21	miR166	12.58	5.33	2.360	1.2388
aof-miR166e-11	UCGGACCAGGCUCCAUUCCCC	21	miR166	8.46	1.73	4.890	2.2898
aof-miR165a	UCGGACCAGGCUUCAUCCCCC	21	miR166	661.69	1436.29	0.461	–1.1172
aof-miR166l-2	UCGGACCAGGCUUCAUUUCUC	21	miR166	2.52	6.12	0.412	–1.2793
aof-miR166i-6	UCGGACCAGUCUUCAUUCCCC	21	miR166	1.60	13.33	0.120	–3.0589
aof-miR166k-1	CUCGGACCAGGCUUCAUCCCC	21	miR166	1.37	15.37	0.089	–3.4901
aof-miR166d-2	UCGGACCAGGCUUCAUUACCC	21	miR166	5.03	1.25	4.024	2.0086
aof-miR166d-4	UCGGCCCAGGCUUCAUUCCCC	21	miR166	17.61	1.57	11.217	3.4876
aof-miR166d-5	UCGGGCCAGGCUUCAUUCCCC	21	miR166	46.19	5.02	9.201	3.2018
aof-miR166d-6	UCGGGCCAGGCUUCAUUCCUC	21	miR166	6.63	1.10	6.027	2.5914
aof-miR166i-9	UCGGUCCAGGCUUCAUUCCCC	21	miR166	8.69	0.94	9.245	3.2087
aof-miR166b-p3	UCUCAGACCAGGCUUCAUUCC	21	miR166	6.63	2.67	2.483	1.3121
aof-miR166a-5	UCUCGGACCCGGCUUCAUUCC	21	miR166	0.46	7.37	0.062	–4.0116
aof-miR166m-1	UCGGACCAGGCUUCAUUCCUUU	22	miR166	2.29	6.27	0.365	–1.4540
aof-miR167g	UGAAGCUGCCAGCAUGAUC	19	miR167	117.74	54.25	2.170	1.1177
aof-miR167b	GGUCAUGCUCUGACAGCCUCACU	23	miR167	10.52	3.29	3.198	1.6772
aof-miR168d	CGCUUGGUGCAGGUCGGGAA	20	miR168	5.26	2.04	2.578	1.3663
aof-miR168f	CCCGCCUUGCACCAAGUGAAU	21	miR168	1.14	11.61	0.098	–3.3511
aofmiR168e	UCGCUUGGUGCAGGUCGGGU	20	miR168	13.03	1.88	6.931	2.7931
aof-miR168a-2	UCGCUUGGUGCAGAUCGGGAC	21	miR168	6.63	57.41	0.115	–3.1203
aof-miR168a-4	GAUCCCGCCUUGCACCAAGUGAAU	24	miR168	0.69	7.53	0.092	–3.4422
aof-miR171f	UGAUUGAGCCGUGCCAAUAUC	21	miR171	128.27	39.06	3.284	1.7155
aof-miR172b	GUGGCACCAUCAAGAUUCACA	21	miR172	27.67	8.00	3.459	1.7904
aof-miR390c	AGCUCAGGAGGGAUAGCGCC	20	miR390	5.94	1.57	3.783	1.9195
aof-miR390a	AAGCUCAGGAGGGAUAGCGCC	21	miR390	332.68	94.91	3.505	1.8094
aof-miR393a	UCCAAAGGGAUCGCAUUGAUC	21	miR393	5.49	2.35	2.336	1.2240
aof-miR393b	UCCAAAGGGAUCGCAUUGAUCU	22	miR393	37.95	9.10	4.170	2.0600
aof-miR396a-5p	UUCCACAGCUUUCUUGAACU	20	miR396	24.24	60.55	0.400	–1.3219
aof-miR396f	UCCACAGGCUUUCUUGAACUG	21	miR396	3.66	18.20	0.201	–2.3147
aof-miR396g	UUCCACAGCCUUCUUGAACUG	21	miR396	1.83	6.12	0.299	–1.7418
aof-miR396b-4	UUCCACAGCUUUCUUGAACUU	21	miR396	3.89	10.82	0.360	–1.4739
aof-miR408a	UGCACUGCCUCUUCCCUGGC	20	miR408	7.09	23.69	0.299	–1.7418
aof-miR408b	UGCACUGCCUCUUCCCUGGCU	21	miR408	32.24	170.99	0.189	–2.4035
cme-miR408c	UGCACUGCCUCUUCCCUGGCUU	22	miR408	13.49	59.61	0.226	–2.1456
aof-miR171h	UGAGCCGAACCAAUAUCACUC	21	miR479	185.20	46.59	3.975	1.9910
aof-miR5179	UUUUGCUCAAGACCGCGCAAC	21	miR5179	97.17	47.37	2.051	1.0363
aof-miR827c	UUAGAUGACCAUCAACAAACA	21	miR827	377.95	167.85	2.252	1.1712
**aof-miRn08**	**AGCGGGGUGUUCUGAUCCAUA**	**21**		**5.03**	**1.88**	**2.676**	**1.4201**
**aof-miRn09**	**AGCGGGGUGUUCUGAUCCAUACAA**	**24**		**9.83**	**3.29**	**2.988**	**1.5792**
**aof-miRn17**	**CCAGACGGUCGGCGGGCUGAA**	**21**		**4.57**	**9.10**	**0.502**	**–0.9942**
**aof-miRn23**	**CGAACCCUGGUCGAUUGUUUUU**	**22**		**2.52**	**6.43**	**0.392**	**–1.3511**
**aof-miRn29**	**GUGCUUCCCCUCGUUGUCACC**	**21**		**0.00**	**10.04**	**0.000**	
**aof-miRn30**	**UAAAUAGUCGGGGUUGCCAACC**	**22**		**3.89**	**9.57**	**0.406**	**–1.3004**
**aof-miRn33**	**UGGCGUGCAUGGAAUCAAGCA**	**21**		**13.72**	**5.80**	**2.366**	**1.2425**

TPM transcripts per million. Bold font highlights novel miRNAs in *A. officinalis*. All the differentially expressed miRNAs were screened out at the restrictive condition of *p* value < 0.05

**Fig. 4:**
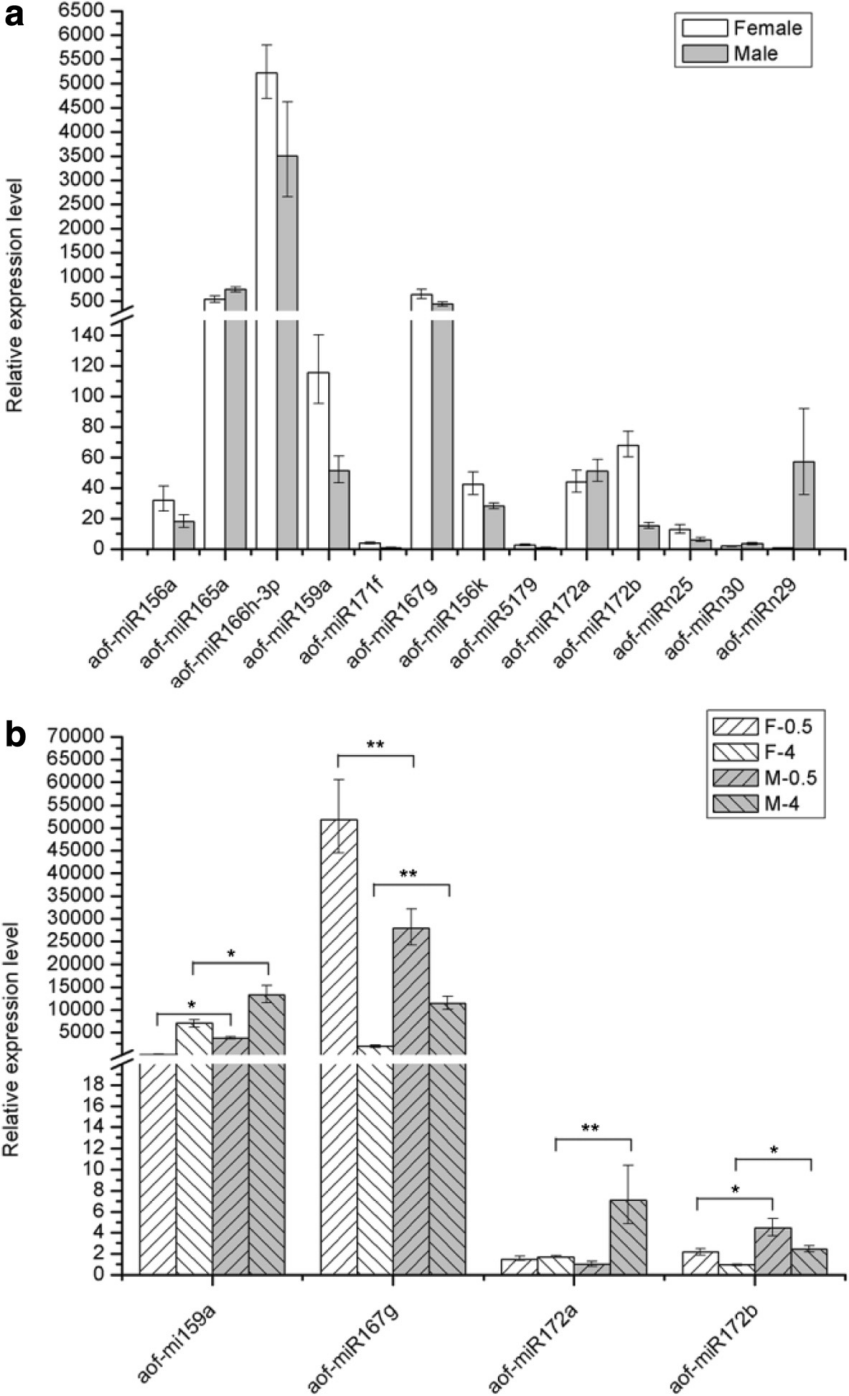
Comparison of miRNA expression levels between asparagus male and female individuals through qRT-PCR. **a** Expression levels of 13 selected miRNAs between male and female plants. **b** Expression profiles of aof-miR159a, aof-miR167g, aof-miR172a and aof-miR172b during male and female flower development. F-0.5, 0.5 mm female flower buds; M-0.5, 0.5 mm male flower buds; F-4, 4.0 mm female flower; M-4, 4.0 mm male flower. *or **indicates a statistically significant difference between male and female flowers at the same stage at *P <* 0.05 or 0.01, respectively

To confirm the expression patterns of miRNAs in asparagus derived from the high-throughput sequencing, 15 identified miRNAs were selected and subjected to qRT-PCR analysis in either plants or flowers. The results of qRT-PCR analysis were consistent with the sequencing data (Fig. 4a), except for aof-miR156k, which exhibited more than 23-fold higher expression levels in the female library than that in the male library based on sequencing data, whereas only 1.5-fold levels were detected in female plants than that in male plants by qRT-PCR analysis. Therefore, the sequence data is trustworthy and can be used for further analyses.

The expression profiles of differentially expressed miRNAs in male and female flower buds were estimated during the early (with a length of 0.5 mm) and late (with a length of 4 mm) stages through qRT-PCR to evaluate the correlations between expression levels of these miRNAs, including aof-miR167g, aof-miR172a, aof-miR172b, and aof-miR159a with their potential roles in sex determination or flower development. These miRNAs exhibited differential expression patterns during asparagus flower development (Fig. 4b). The expression of aof-miR167g was decreased in both male and female flowers during development, with TPM of approximately 26-fold higher in 0.5 mm female floral buds than that in 4 mm ones. By contrast, changes in male flowers were less than 2-fold. Aof-miR159a exhibited opposite expression pattern, with 32-fold higher abundance in late male flowers (4 mm) than that in young male floral buds (0.5 mm). Meanwhile, the expressions levels of aof-miR159a in male flowers were approximately 17-fold higher than that in female flowers at the early developmental stage. Furthermore, the expression level of aof-miR160d and aof-miR396f were further estimated in 0.5 mm, 2 mm, and 4 mm floral buds (Additional file 2: Figure S1), Comparison between male and female flowers showed that the expression level of aof-miR160d was higher in female flowers than that in male flowers, especially in the 2 mm floral buds, whereas the expression level of aof-miR396f showed a higher expression level in 2 mm male flower than female one, reaching to about 4-fold change.

### MiRNA putative target prediction and annotation using degradome analysis

Putative targets were predicted by high-throughput degradome sequencing to determine the function of the identified miRNAs in asparagus [12]. The male and female samples were mixed and used to construct a degradome library. A total of ~10.13 million raw reads were obtained, which represent 7,532,780 (74.4 %) unique sequences. These reads were mapped to the asparagus unigene sequences assembled from published asparagus ESTs (http://www.ncbi.nlm.nih.gov/nucest) to identify potential miRNA targets. A total of 2,486,559 (~33 %) unique sequences could be mapped to the reference asparagus unigene data. After initial processing and analyzing by CleaveLand4 (http://sites.psu.edu/axtell/software/cleaveland4/), 40 target gene sequences for 44 conserved and seven novel miRNAs were identified based on the available asparagus dataset (Additional file 3: Table S4).

**Fig. 5:**
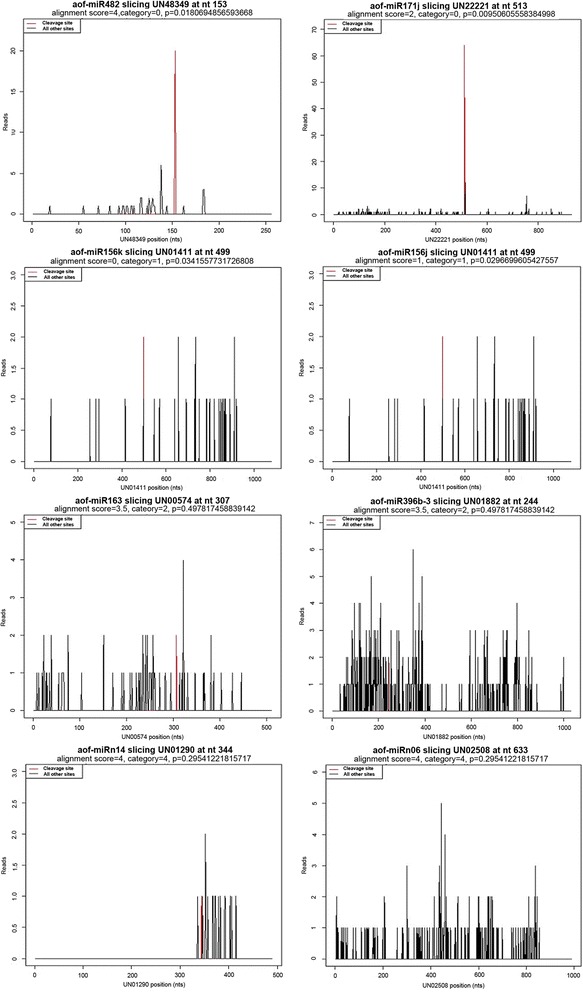
Target plot (t-plots) of representative validated asparagus miRNA targets in different categories as confirmed by degradome sequencing

Relative abundance was plotted for each transcript. The sliced-target transcripts were grouped into five categories, namely, category 0, 1, 2, 3, and 4, according to the relative abundance of tags at the target sites as previously reported in *Arabidopsis* [11], rice [12], and maize [40]. These transcripts contained more than one raw read at the position, except for category 4 with only one raw read [41]. The miRNAs and corresponding targets in the four categories are shown in Additional file 3 and Fig. 5. Among the 40 identified targets, 15, 12, and 12 targets were classified into categories 0, 2, and 4, respectively. Only one target was found in category 1, and no target belonged to category 3. These results indicate that most of the predicted targets are efficiently cleaved by their corresponding miRNAs.

Target prediction analysis showed that the identified targets regulated a wide range of biological processes. Most of the conserved targets were transcription factor genes, such as translation initiation factor, hormone response factor, AP2-like factor, and scarecrow-like protein, which regulate plant growth and development, as well as stress responses [42–44]. The mRNAs of heat shock proteins (HSPs), histones, transport inhibitor response proteins, dehydrogenases, kinesin-like protein, sulfite reductases and some putative uncharacterized proteins are likely to be targeted by asparagus miRNAs. Interestingly, several targets identified in the present study were previously reported to be involved in reproductive development in plants; these targets included MYB proteins targeted by miR159 [45], AP2-like transcription factors regulated by miRNA172 [46, 47], and *ARF6* or *ARF8* controlled by miR167 [48].

Target analysis showed that a single miRNA can simultaneously regulate several target genes, which usually belong to a large gene family. As predicted, some highly conserved miRNAs such as miR156, miR396, miR167, and miR482, had multiple targets, which is consistent with previous reports in *Arabidopsis* [12]. For example, the miR156 family can regulate several target genes such as the squamosa promoter-binding-like protein, histone H2B.11, and methyltransferase (Additional file 3). On the other hand, the majority of miRNAs from the same family and even some miRNAs from different families could regulate the same target genes. Meanwhile, one mRNA could be a potential target of several different miRNAs. For example, aof-miR167a and aof-miR827 can regulate the expression of 6-phosphogluconate dehydrogenase. Moreover, aof-miR156 and aof-miR157 had the same target sequence, namely, UN13110, and both have been predicted to target the same EST sequence for squamosa binding proteins in *Phaseolus vulgaris* [49].

Although novel asparagus miRNAs were sequenced at relatively lower levels compared with known miRNAs, seven out of 39 novel miRNAs were found to have candidate targets in asparagus (Additional file 3: Table S4). Among these miRNAs, aof-miRn06 and aof-miRn17 had only one target gene, whereas aof-miRn39 had three target genes, including a mediator of RNA polymerase II transcription subunit 26b. No targets were identified for the other 32 novel miRNAs in the degradome sequencing data, which may be partly attributed to the limited genome and transcriptome information in asparagus. Notably, aof-miRn13 and aof-miRn14 shared the same targets, thereby suggesting that both may belong to the same miRNA family or may refer to the same miRNA because of the high similarity of their nucleotide sequences.

### Verification of miRNA-guided cleavage of target mRNAs in asparagus

**Fig. 6:**
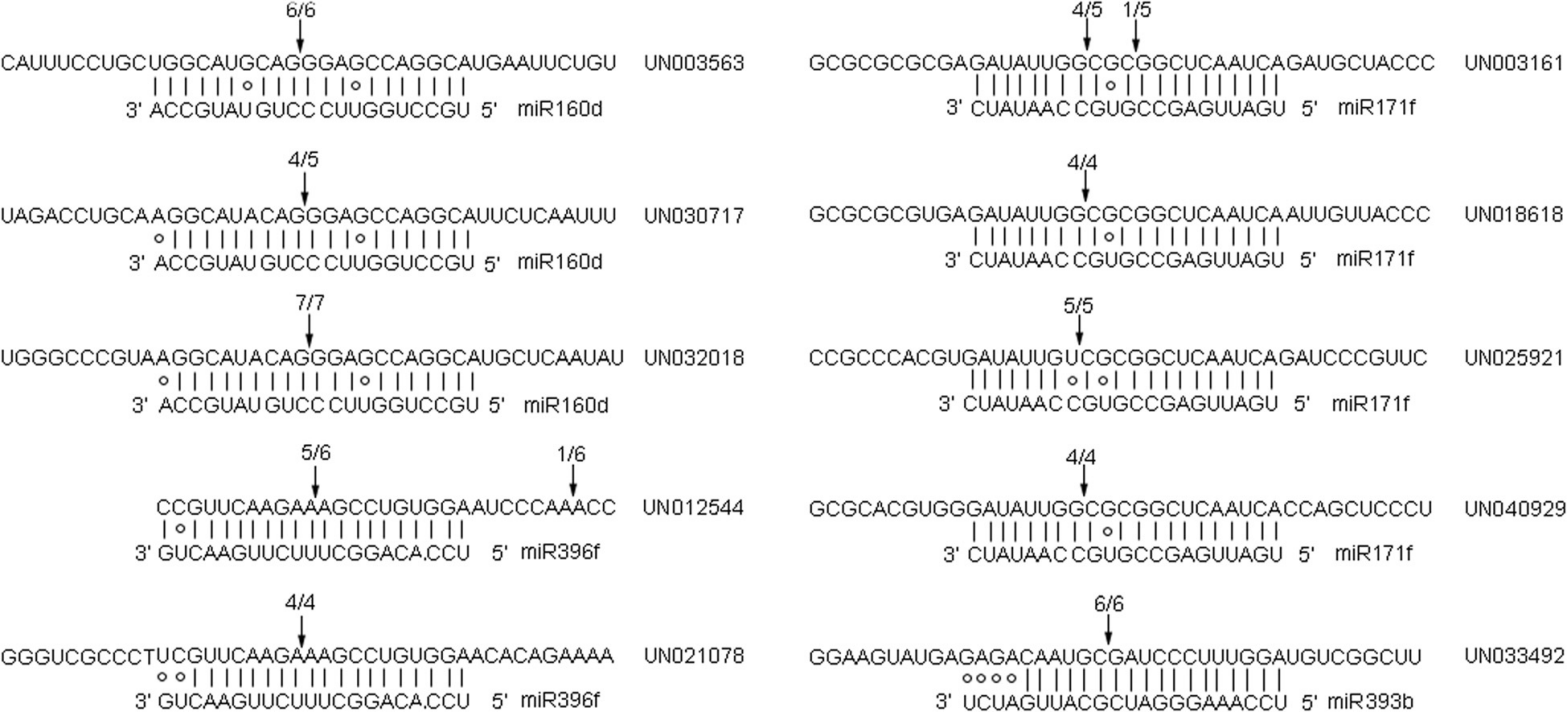
Detection of predicted miRNA target genes in asparagus by 5′ RLM-RACE. Arrows point to the cleavage sites of targeted mRNAs for four asparagus miRNAs. The Watson-Crick pairing (vertical dashes) and mismatch pairing (circles) are shown in the complementary pairing area of miRNA and its target. The denominator and numerator of the fraction indicate the number of sequenced monoclonal sequences and the number of monoclonal sequences with the cleavage site at the arrow, respectively. Only the monoclonal sequences with the cleavage sites in the complementary pairing area of miRNA/target or nearby 10 nucleotides were counted

The psRNA Target (http://plantgrn.noble.org/psRNATarget/) was used to predict the target unigenes of asparagus miRNAs by querying specific miRNA sequences against the asparagus unigene database created by transcriptome sequencing (RNA-seq). The results were combined with degradome analysis data. Ten predicted mRNAs for four asparagus miRNAs were selected, and their cleavage products were detected by 5′ RLM-RACE to verify the miRNA-guided target cleavage. As shown in Fig. 6, all of the 10 predicted targets were found to contain one or two specific cleavage sites, which correspond to the complementary miRNA sequences. All four tested asparagus miRNAs guided target cleavage, mostly at the 10th or 11th nucleotide (Fig. 6). In our degradome sequencing data, scarecrow-like protein 6, which belongs to GRAS family, was predicted as the target of miR171f (Additional file 3). Consistently, four GRAS family transcription factors including UN003161, UN018618, UN025921, and UN040929, were also identified as targets cleaved by miR171f in the 5′ RLM-RACE experiment. Two growth-regulating factors (UN012544 and UN021078) were identified to be cleaved by miR396f, which is consistent with degradome sequencing data. Three auxin response factors (UN003563, UN030717, and UN032018) and a transport inhibitor response protein (UN033492) were also identified as the target genes of miR160d and miR393b, respectively, which is consistent with previous studies [50, 51].

### GO functional analysis of targets regulated by asparagus miRNA

Putative target genes were subjected to GO analysis and mapped to the metabolic pathways from the KEGG database to elucidate the miRNA–target interaction and ensure the accuracy of gene annotation. Only 19 out of 40 target unigenes could be mapped to the function and metabolic pathway databases; these genes were regulated by 26 corresponding miRNAs (Additional file 4: Table S5). These target genes were also found to be involved in a wide spectrum of biological processes (Table 4), cellular components, and molecular functions including transcription regulation, auxin–mediated signaling pathways, circadian rhythm, cell division, flower and seed development, metabolic processes, and defense responses. Several miRNAs from different families were involved in the same biological process. For instance, the miR167 and miR393 families participated in auxin mediated signaling pathways. The miR156, miR167, and miR171 families were associated with transcription regulation via DNA-dependent mechanisms. In addition, the majority of the target genes were involved in nucleic acid and protein binding, as well as protein dimerization. Furthermore, GO classification demonstrated that most of the predicted proteins (19 unigenes) were located in the nucleus. The other proteins functioned in the extracellular region, endoplasmic reticulum, cytoplasm, and Golgi apparatus.

**Table 4:** GO term analysis on different expressed miRNAs between asparagus male and female plant

GO biological process	miRNAs	Unique gene
Transcription, DNA − dependent	miR156k; miR156j; miR167a; miR167e; miR167c; miR171b; miR171f; miR171j; miR171a	UN01411; UN21824; UN04709; UN08763; UN12573; UN06775; UN22221
Regulation of transcription, DNA− dependent	miR156k; miR156j; miR167a; miR167e; miR167c; miR171b; miR171f; miR171j; miR171a	UN01411; UN21824; UN04709; UN08763; UN12573; UN06775; UN22221
Auxin mediated signaling pathway	miR167a; miR167e; miR167c; miR393a; miR393c; miR393b	UN21824; UN04709; UN08763; UN12573; UN06775; UN15466
Circadian rhythm	miR171b; miR171f; miR171j; miR171a	UN22221
Root hair cell tip growth	miR171b; miR171f; miR171j; miR171a	UN22221
Cell division	miR171b; miR171f; miR171j; miR171a	UN22221
Mitotic cell cycle	miR396a-3p; miR396d;	UN14593
Nucleosome assembly	miR156k; miR156j;	UN15310
Methionine biosynthetic process	miR156k; miR396b-4;	UN17597; UN27824
Flower development	miR167a;	UN08763; UN12573
Histidine biosynthetic process	miR396b-4;	UN27824
Carbohydrate metabolic process	miR166d-6;	UN14719
Purine nucleotide biosynthetic process	miR396b-4;	UN27824
One − carbon metabolic process	miR396b-4;	UN27824
Transport	miR408a	UN10287
Defense response	miR166d-6;	UN14719
Purine base biosynthetic process	miR396b-4;	UN27824
Folic acid-containing compound biosynthetic process	miR396b-4;	UN27824
Meristem maintenance	miR172a	UN21982
Specification of floral organ identity	miR172a	UN21982
Electron transport chain	miR408a	UN10287
Cell differentiation	miR172a	UN21982
Seed development	miR172a	UN21982

### Expression profiles of target genes for differentially expressed miRNAs

**Fig. 7:**
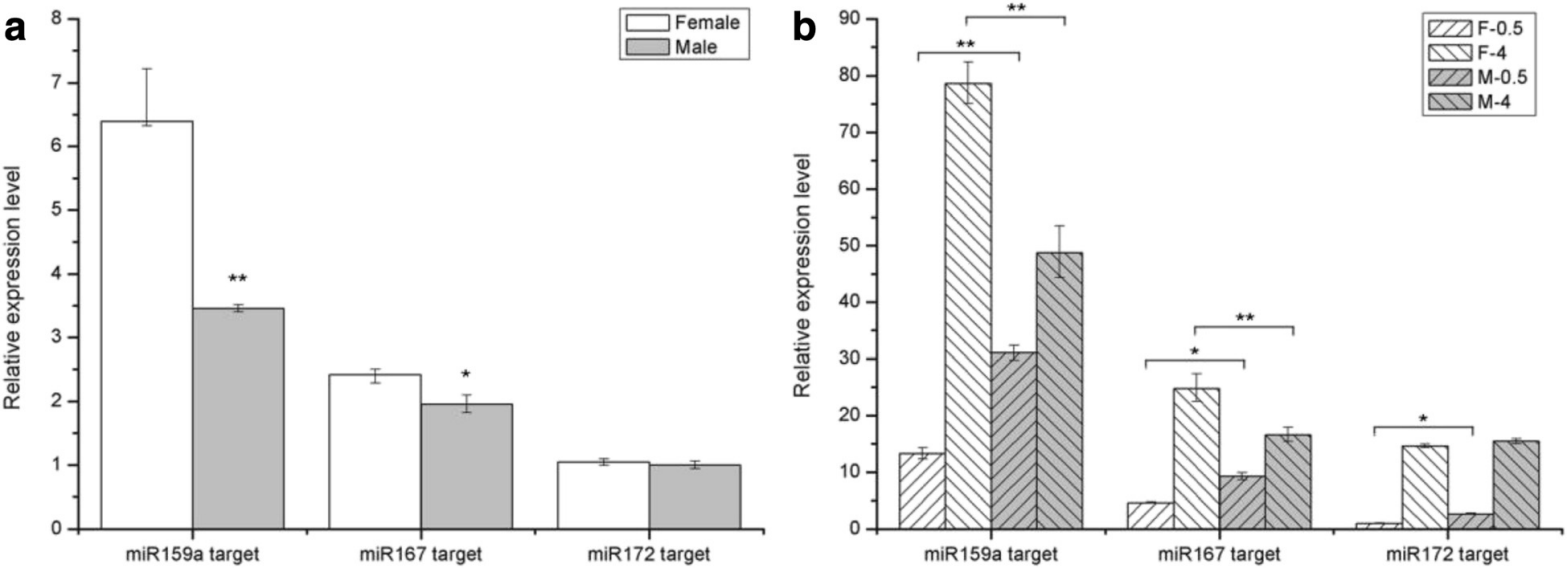
Comparison of the expression levels of miRNA target genes between male and female individuals through qRT-PCR. **a** The expression level of *EIF-2* (UN00815), *ARF6* (UN12573) and *AP2* (UN21982), targeted by aof-miR159, aof-miR167 and aof-miR172 family, respectively, in male and female plants. *or **indicates a statistically significant difference between male and female plants at *P <* 0.05 or 0.01, respectively. **b** The expression level of target genes in 0.5 mm and 4 mm female and male flower buds. F-0.5 and F-4 represent 0.5 mm and 4 mm female flower respectively; M-0.5 and M-4 represent 0.5 mm and 4 mm male flower, respectively. *or **indicates a statistically significant difference between male and female flowers at the same stage at *P <* 0.05 or 0.01, respectively

QRT-PCR was used to detect the expression profiles of miRNA specific targets during male and female floral development and validate the mechanism of a given miRNA to regulate the expression of its target gene. The expression patterns were estimated in male and female plants, as well as in 0.5 mm and 4 mm flowers for three unigenes, namely UN00815, UN12573 and UN21982, as putative target sequence of the aof-miR159, aof-miR167 and aof-miR172 family, respectively (Fig. 7). Meanwhile, three targets (UN003563, UN030717 and UN032018) for aof-miR160d and three targets (UN021544, UN021078 and UN028196) for aof-miR396f were predicted from the unigene database created by RNA-seq, and their expression levels were estimated in 0.5 mm, 2 mm and 4 mm male and female flowers (Additional file 2). A negative correlation was observed between the expression of miRNAs and their targets (Figs. 4b and 7, Additional file 2). The targets of aof-miR160d and aof-miR396f were differentially expressed between male and female flower at one or more than one developmental stage, suggesting that aof-miR160d and aof-miR396f may participate in floral development via negatively regulating their targets. MiR172 had significantly higher expression level in 0.5 mm flowers than that in 4 mm flowers of male or female plants. Conversely, its putative target sequence, UN21982, was homologous with the floral homeotic protein AP2 in *Arabidopsis* and expressed at a significantly lower level in 0.5 mm flowers than that in 4 mm flowers. *AP2* negatively regulates multiple floral organ identity genes in *Arabidopsis* [52]. Similarly, miR167 levels were lower in 4 mm flowers than those in 0.5 mm flowers in the present study. By contrast, the target gene UN12573, which encodes an auxin response factor 6 (*ARF6*), demonstrated higher expression levels in 4 mm flowers than those in 0.5 mm flowers. These findings indicate that miR172 and miR167 could regulate the putative targets *AP2* and *ARF6,* respectively. The inverse relationship between miRNAs and their target genes is consistent with the predicted mechanism of miRNA function. However, the altered expression levels of miR159 and its putative target UN00815, which encodes an eukaryotic translation initiation factor 2 subunit β (EIF-2), followed the same trend during floral development; as such, a complex regulating regulatory mechanism exits between miRNAs and their target genes [53]. MiRNAs have been proposed to upregulate their target genes upon cell cycle arrest, although the underlying mechanism has not been elucidated [54].

## Discussion

MiRNAs are key components of numerous cellular events in plant development and responses to various stresses. Increasing evidence has indicated that plant miRNAs are also involved in development and morphogenesis. With the development of high-throughput sequencing and bioinformatics approaches, miRNAs have been identified from various plant species with or without fully sequenced genomes, including *O. sativa, R. sativus, Populus trichocarpa, Pinus contorta, M. truncatula, C. trifoliata, Panicum virgatum,* and *P. ginseng* [4, 6, 25, 28, 30, 32, 38]. Asparagus is a perennial vegetable, important in various countries because of its importance to health and economy. To date, no asparagus miRNAs data have been reported in the plant miRNA database. In the present study, sRNA libraries of male and female asparagus plants were constructed and sequenced using the Illumina HiSeq system, which generated 4–5 million clean shorter reads (up to 35 bp) per sample. From these sRNA sequences, a total of 154 conserved miRNAs belonging to 26 miRNA families and 39 novel candidate miRNAs were identified in garden asparagus (Additional file 1: Table S1).

Previous studies predicted conserved miRNAs according to their homology to known miRNAs in miRBase. In the present study, homology-based predictions of conserved miRNAs in asparagus were validated by precursor sequence folding and the genuine hairpin structures. Some miRNAs that were previously identified in a wide range of plants were also found in the present study; such miRNAs include the miR159, miR166, miR171, miR172, and miR396 families. The miR166 family contained the highest number of miRNA members. Meanwhile, some highly conserved miRNA families were sequenced with more than 1,000 TPM; these families included the miR159, miR164, miR166, miR167, miR396, and miR535 families (Additional file 1). Therefore, these highly expressed miRNAs may have an essential role in the regulation of asparagus growth and development. Some conserved miRNAs in asparagus have also been studied in detail in other plants. For example, miR171 in *Arabidopsis* targets mRNAs coding for the GRAS domain or scarecrow-like proteins, a family of transcription factors whose members have been implicated in axillary meristem formation, gibberellin and light signaling, gametogenesis, and root radial patterning [55–57]. A previous study showed that miR393 could target mRNAs coding for the TIR1 (an F-box protein) family in *Arabidopsis,* which is required for auxin responses in plant development [51]; miR393 was also strongly upregulated by cold, dehydration, and NaCl treatments [58].

Despite the lack of complete genomic sequences, the availability of asparagus EST and transcriptome sequences contributed to the identification of novel asparagus miRNAs. Based on the hairpin structures of pre-miRNAs, 39 novel candidate miRNAs that met the analytical criteria were identified in this study. The number of potential specific miRNAs in asparagus is comparable with some plant species, such as *B. oleracea* (26) [33], *P. ginseng* (28) [39], *P. vulgaris* (29) [49], and strawberry (37) [59]. Among the novel candidate miRNAs, aof-miRn28, aof-miRn38, and aof-miRn39 presented higher expression levels than the other miRNAs in both male and female individuals, thereby implying their special role in asparagus growth and development. The functions of most novel candidate miRNAs remain unknown because of limited asparagus genome information.

The selection and annotation of miRNA targets are essential steps in the designation of miRNA function in plants. Degradome sequencing based on high-throughput sequencing technology has been used to identify the targets of miRNAs and understand the miRNA regulatory network [10]. In the present study, 40 potential targets were found for 51 miRNAs; these miRNAs were grouped into four categories (Additional file 3). The 5′ RLM-RACE experiment was then performed to detect and verify the cleavage products of the 10 predicted mRNAs for four asparagus miRNAs; the results showed that target cleavage often occurred at the 10th or 11th nucleotide. Similar to previous reports, the targeted genes by of the test miRNAs had a wide range of functions; the majority of the targets were translation and transcription factors, which are involved in growth and development processes. In the present study, *ARF6* and *ARF8* could be regulated by aof-miR167, which participate in flower and fruit development in *Arabidopsis* and tomato [60]. Translation initiation factors could be targeted by aof-miR159, which is involved in anther development [61] and seed germination [62] by modulating hormone signaling pathways. Another transcription factor, squamosa promoter-binding-like protein 9 (SPL9) is a predicted target of the miR156 family, which exhibits crucial role in root development and transition from juvenile to adult phases [63]. Several miRNA targets could encode proteins involved in responses to environmental stresses. The HSP family is one of the largest groups of proteins induced by heat shock stress; these proteins are present in almost all organisms and have significant functions in cellular homeostasis under adverse environment conditions [64]. In the present study, aof-miR396b-3 and aof-miR396c-p5 could regulate the expression of *HSPs*. Furthermore, the transport inhibitor response protein was found to be the target of the miR393 family; this protein was thought to be strongly upregulated under salt stress conditions [57].

Asparagus has become a model plant for investigation of early sex chromosome evolution [65]. The sexual dimorphism in asparagus is controlled by a region called the *M* locus, which is located on a pair of homomorphic sex chromosomes (chromosome L5) [22, 23]. However, cloning the sex-determining region is hindered by the presence of highly repetitive sequences localized to the centromeric and pericentromeric regions in asparagus chromosomes [66]. The sex determination mechanism in dioecious plants remains unclear. Given that miRNAs are likely to participate in sex differentiation/maintenance, we employed high-throughput sequencing in the present study to explore the complex miRNA-mediated regulatory networks, which control asparagus reproductive development, especially sex determination. A total of 63 miRNAs were found to be significantly and differentially expressed between male and female asparagus (by more than 2-fold, adjusted *P* < 0.05), which may be associated with sex determination. Based on the sequencing data, 37 miRNAs including aof-miR159a, aof-miR167g, and aof-miR172b were significantly upregulated in female plants, whereas the other 26 miRNAs including aof-miR165a and aof-miRn30 were upregulated in male plants. In particular, aof-miRn29 was only detected in male plants (Table 3). These differentially expressed miRNAs were further verified by qRT-PCR. Previous studies showed that most of these differentially expressed miRNAs performed multiple regulatory functions in floral organ formation and differentiation. The miR159 family is thought to target mRNAs encoding MYB proteins, it has been showed that MYB33 which bind to the promoter of the floral meristem identity gene LEAFY, as well as MYB65, could redundantly facilitate anther development in *Arabidopsis* [67]. In the present study, aof-miR159 was predicted to target *EIF-2*, which functions in the early stages of protein synthesis by forming a ternary complex with GTP and the initiator tRNA followed by binding to the 40S ribosomal subunit, however, there is no evidence at present to show the correlation of *EIF-2* with sex differentiation and floral organ development. The miR172 family in asparagus could target mRNAs encoding floral homeotic protein AP2, which affects *Arabidopsis* flower development [46]. Furthermore, miR172 in maize could target AP2 to control sex determination [47]. Scarecrow-like protein 6, which was suggested to influence gibberellin signaling in *Arabidopsis* [55], was predicted to be targeted by four miR171 family mumbers in asparagus. Since gibberellin could promote stamen and anther development in *Arabidopsis* [68], and promote masculinity in *Cannabis sativa* and *Spinacia oleracea* [69], the scarecrow-like protein 6 could be inferred to have a role in sex determination in asparagus.

Auxin is implicated in various physiological and developmental processes in plants. ARF has been reported to regulate flower and leaf development by controlling auxin responses [70]. MiR319 was reported to target *ARF2* genes, whereas miR160a may target *ARF16*/*17. ARF2* is a transcriptional suppressor involved in regulation of ethylene, auxin, ABA, and brassinosteroid to control the onset of leaf senescence, floral organ abscission and ovule development [71]. *ARF2* promotes transitions between multiple stages of *Arabidopsis* development and positively regulates flower development [72]. In *Arabidopsis*, *ARF6* and *ARF8* were validated as targets of miR167 and essential for the fertility of ovules and anthers [48]. Recently, Liu et al. [60] suggested that the miR167 family is essential for regulating gynoecium and stamen development in immature tomato flowers by modulating the expression levels of *SlARF6* and *SlARF8*. In the present study, the expression level of some miR167 family genes was significantly higher in female asparagus than that in male plants. The levels of aof-miR167g and aof-miR167b were at least 2-fold higher in female flowers than that in male ones. The high levels of miR167 in females can down-regulate *ARF*6 or *ARF*8 expression and thus regulate fruit and seed development. We found that the expression levels of ARF6 increased during flower development. *ARF6* was highly expressed in 4 mm female and male flowers, as indicated by the low expression of its corresponding miRNA in 4 mm flowers. Previous studies have shown that sex differentiation did not occur in 0.5 mm asparagus flowers [73], thereby implying the important roles of miR167 and *ARF6* in floral differentiation in the later development stages. Furthermore, the expression level of *ARF6* was higher in 4 mm female flowers than that in males, thereby suggesting that *ARF6* may be required to support gynoecium growth, as proved by previous studies [48]. Further research on miRNAs is worthwhile, particularly with regard to floral differentiation and sex determination.

## Conclusions

High-throughput sequencing technology was used for the first time to identify 154 known and 39 candidate novel miRNAs in *A. officinalis* plants. Through degradome sequencing and bioinformatics analysis, 40 non-redundant targets for conserved and novel miRNA were identified. These potential targets in asparagus are involved in diverse biological processes, including hormone signaling, flower development, metabolism and transcription regulation. The expression levels of the identified miRNAs and their corresponding targets were compared between male and female plants and verified by qRT-PCR. Several important miRNAs with different expression levels between male and female individuals are suggested to be sex-chromosome specific and associated with reproductive organ development and sex determination in asparagus. Given that the complete asparagus genome sequence remains unavailable, the full set of asparagus miRNAs and their targets should be further investigated. Nonetheless, our research provides a basis to elucidate the complex miRNA-mediated regulatory networks that control development and other physiological processes in asparagus.

## Methods

### Plant material

The asparagus cultivar “Grand” (California Asparagus Seed and Transplants, Inc.) was grown in a controlled greenhouse at an average temperature of 22 °C to 32 °C during the day and 18 °C to 26 °C at night, with a relative humidity of 65 %–80 %. The leaves, roots, stems, and flowers of the 3-year-old male and female plants were collected. Samples from 10 individuals were pooled, immediately frozen in liquid nitrogen and stored at -80 °C until further use.

According to previous studies, the development of asparagus flowers can be divided into 13 stages [73, 74]. Sex determination occurs at stage “-1”, when the length of flower buds is less than approximately 0.7 mm. Male and female flower buds with lengths of 0.5 ± 0.1 mm, 2.0 ± 0.5 mm, and 4 ± 0.5 mm, which respectively represent the periods before, during and after sex determination, were separately sampled from male and female plants. Each of the six floral samples was collected from more than 15 different plants and subsequently mixed, and immediately frozen in liquid nitrogen. Total RNA was isolated using the modified Trizol method and used for qRT-PCR analysis.

### Small RNA library construction and high-throughput sequencing

Total RNA was isolated with TRIzol reagent (Invitrogen, USA) according to the manufacturer’s protocols. Genomic DNA contamination was removed using RQ1 RNase-Free DNase (Promega, USA). The quantity and quality of the total RNA were assayed with a NanoDrop ND-1000 spectrophotometer (NanoDrop). Equal amounts of total RNA from each organ were pooled to obtain the respective total RNAs of male and female plants. The sRNA fractions between 10–55 nt were isolated from the total RNA pool with Novex 15 % TBE-urea gels (Invitrogen, USA).

SRNA libraries were constructed with the Illumina Truseq Small RNA Preparation kit and subjected to next generation sequencing using Illumina HiSeq technology at LC Sciences (China), according to the manufacturer’s protocol.

### Asparagus EST sequence collection and de novo assembly

A total of 8422 asparagus ESTs were collected from GenBank. Approximately 210,000 asparagus transcriptome sequences generated using the 454 pyrosequencing technology as described by Mercati et al. [25] were downloaded from NCBI Short Sequence Archive (Accession Nos. SRX212313 and SRX212315). All these sequences were processed to remove low quality, low complexity and vector sequences using SeqClean (http://sourceforge.net/projects/seqclean/). The cleaned sequences were de novo assembled into unigenes using iAssembler using default parameters [75]. The unigene sequences were provided as Additional file 5.

### Bioinformatics analysis of sequencing data

Raw sequences for the two libraries were cleaned of sequence adapter, low quality tags and small sequences (< 15 nt long). The identical adaptor trimmed sequences in the range of 15–45 nt were then selected for mapping of putative mRNA and non-coding RNA including rRNA, tRNA, snRNA, and snoRNA, deposited at the Rfam (ftp://ftp.sanger.ac.uk/pub/databases/Rfam/11.0/) and NCBI GenBank databases. The remaining sequences were clustered into unique sRNAs with normalized counts for the individual sequence reads. Unique sRNAs with TPM ≥5 in at least one sample and lengths between 20–24 nt were included for miRNA identification. These sRNAs were aligned to the reference sequences including asparagus unigenes or rice genome) with perfect matches. The flanking sequences of sRNAs (200 bp from each side) were extracted and theoretically folded with the RNAfold program (http://mfold.rna.albany.edu/?q=mfold). The potential miRNA candidates were predicted using Mireap (http://sourceforge.net/projects/mireap/) by detecting the secondary hairpin structure, the Dicer cleavage site, and the MFE according to the criteria described by Meyers et al [34]. On the other hand, all these unique sRNAs were also used to query against the mature miRNA sequences in miRBase 21 (ftp://mirbase.org/pub/mirbase) using bowtie allowing up to two mismatches to identify conserved miRNAs [76]. The Fisher’s exact test and *χ*2 test were used to identify differentially expressed miRNAs. The miRNAs with more than 2-fold changes in their expression levels and adjusted *p* values < 0.05 were considered as differentially expressed.

### Degradome sequencing and data analysis

Total RNA from different organs of male and female asparagus plants was equally mixed and used to construct the degradome library according to the method described by Ma et al. [77] with minor modifications. Approximately 150 ng of polyA-enriched RNAs were ligated to the RNA oligonucleotide adaptor containing a 3′ *Mme* I recognition site. The ligation products were used to generate first-strand cDNA by reverse transcription, followed by PCR amplification. After purification and digestion with *Mme* I, the target PCR product was ligated to a double stranded DNA adaptor and gel-purified for PCR amplification. The final cDNA library was purified and sequenced with the Illumina GAIIx platform according to the manufacturer’s instructions.

After sequencing, the adaptor sequences and low quality sequencing reads were removed. The remaining sequences with lengths of 20–21 nt were used to identify potentially cleaved targets by the CleaveLand pipeline, as previously described [78]. The degradome reads were mapped to the asparagus unigene datasets. Only the perfectly matched alignment(s) for a given read were kept and extended to 35–36 nt by adding 15 nt of the upstream sequence. Alignments were retained when the 5′ end of the degradome sequence position coincided with the 10th nucleotide of the miRNA. All the identified targets were subjected to BLASTX analysis to search for similarity, followed by GO term analysis to analyze the miRNA-gene regulatory network.

### QRT-PCR

Fifteen miRNAs were selected and subjected to qRT-PCR to verify the miRNA expression levels derived from high-throughput sequencing. The miRNA specific forward primers and stem-loop RT primers were designed with the primer premier 5.0 software. All the primers are listed in Additional file 6: Table S6. Meanwhile, the expression profiles of nine target genes were estimated by qRT-PCR with primers listed in Additional file 6: Table S7. The specificity and amplification efficiency of each pair of primers were examined through a BLAST search in the NCBI database and by running standard curves with melting curves. The qRT-PCR reactions were run on the CFX96 Real Time System machine (Bio-RAD, USA). Two biological replicates were used to estimate the expression level of miRNAs in male and female plants, three biological replicates were used for other qRT-PCR analysis, each biological replicate has three technical replicates. Each reaction was performed with 20 μL of reaction volume containing 3 pmol specific primers and 12.5 μl SYBR Green Master Mix Reagent (Takara, Japan). The *AoFb15* gene (UN012008) was used as the internal control for the qRT-PCR analysis of target genes. The relative transcript levels were calculated with the 2^-ΔΔCT^ method (Applied Biosystems). Statistical analysis for the expression data was performed using Tukey’s HSD at the *P <* 0.05 or *P <* 0.01 level of significance in SAS software (Version 9.3, SAS Institute, USA).

### Modified 5′ RLM-RACE for the mapping of mRNA cleavage sites

Total RNA was extracted from roots, stems, leaves and flowers of male and female plants with the mirVana kit (Ambion, USA). Poly(A) + mRNA was purified from total RNA using the Oligotex mRNA Kit (Qiagen, Germany), according to the manufacturer’s instructions. A modified procedure for 5′ RLM-RACE was followed with the GeneRacer Kit (Invitrogen, USA), as previously described [79]. Nested PCR amplifications were performed with the GeneRacer 5′- nested primer and the gene-specific primer (Additional file 6: Table S8). PCR products were separated by agarose gel electrophoresis. Distinct bands with the appropriate sizes for miRNA-mediated cleavage were purified (Axygen, USA), ligated to the pGEM-T Easy vector (Promega, USA), cloned, and then sequenced.

### Availability of supporting data

The sRNA sequence data from this study have been submitted to Gene Expression Omnibus (GEO) with the accession number GSE72594 (http://www.ncbi.nlm.nih.gov/geo/query/acc.cgi?acc=GSE72594).

## Additional files

Additional file 1: Table S1. Conserved and novel miRNAs identified from *A. officinalis*; **Table S2.** Pre-miRNA sequences of conserved miRNAs identified from *A. officinalis*; **Table S3.** Predicted novel miRNAs and their pre-miRNA sequences in *A. officinalis*. (XLS 131 kb) Additional file 2: Figure S1. Expression levels of aof-miR160d, aof-miR396f and their targets in male and female floral development. The targets of aof-miR160d and aof-miR396f were predicted from the unigene database created by RNA-seq. F represents female flower, M represents male flower. *or **indicates a statistically significant difference between male and female plants at *P <* 0.05 or 0.01, respectively. (DOC 139 kb) Additional file 3: Table S4. Target genes of miRNAs identified in asparagus using high-throughput degradome sequencing analysis. (XLSX 15 kb) Additional file 4: Table S5. GO term analysis for the whole collection of asparagus miRNA targets identified by degradome sequencing. The target genes are classified into biological process, cellular component and molecular function. (XLSX 12 kb) Additional file 5: Unigene sequences assembled using iAssembler in *A. officinalis*. (DOCX 7867 kb) Additional file 6: Table S6. Primers of real time qRT-PCR analysis for asparagus miRNAs, **Table S7.** Primers for qRT-PCR analysis for some selected target genes; **Table S8.** Primers for RLM-5′ RACE experiment. (XLSX 13 kb)

## Abbreviations

MiRNA: microRNA
qRT-PCR: quantitative reverse transcription polymerase chain reaction
5′ RLM-RACE: RNA ligase-mediated 5′ rapid amplification of cDNA ends
EST: expressed sequence tag
NCBI: national center for biotechnology information
GO: gene ontology
Rfam: RNA family database
sncRNA: small non-coding RNA
BLAST: basic local alignment search tool
TPM: transcripts per million
MFEI: minimum free energy index
