# Supplementary material for: Identification of miRNAs and their targets through high-throughput sequencing and degradome analysis in male and female Asparagus officinalis
Source: BMC Plant Biol. 2016 Apr 12;16:80. doi: 10.1186/s12870-016-0770-z (PMC4828810; doi:10.1186/s12870-016-0770-z)
Supplement: Additional file 2: Figure S1. — Expression levels of aof-miR160d, aof-miR396f and their targets in male and female floral development. The targets of aof-miR160d and aof-miR396f were predicted from the unigene database created by RNA-seq. F represents female flower, M represents male flower. *or **indicates a statistically significant difference between male and female plants at P < 0.05 or 0.01, respectively. (DOC 139 kb) [file 12870_2016_770_MOESM2_ESM.doc]

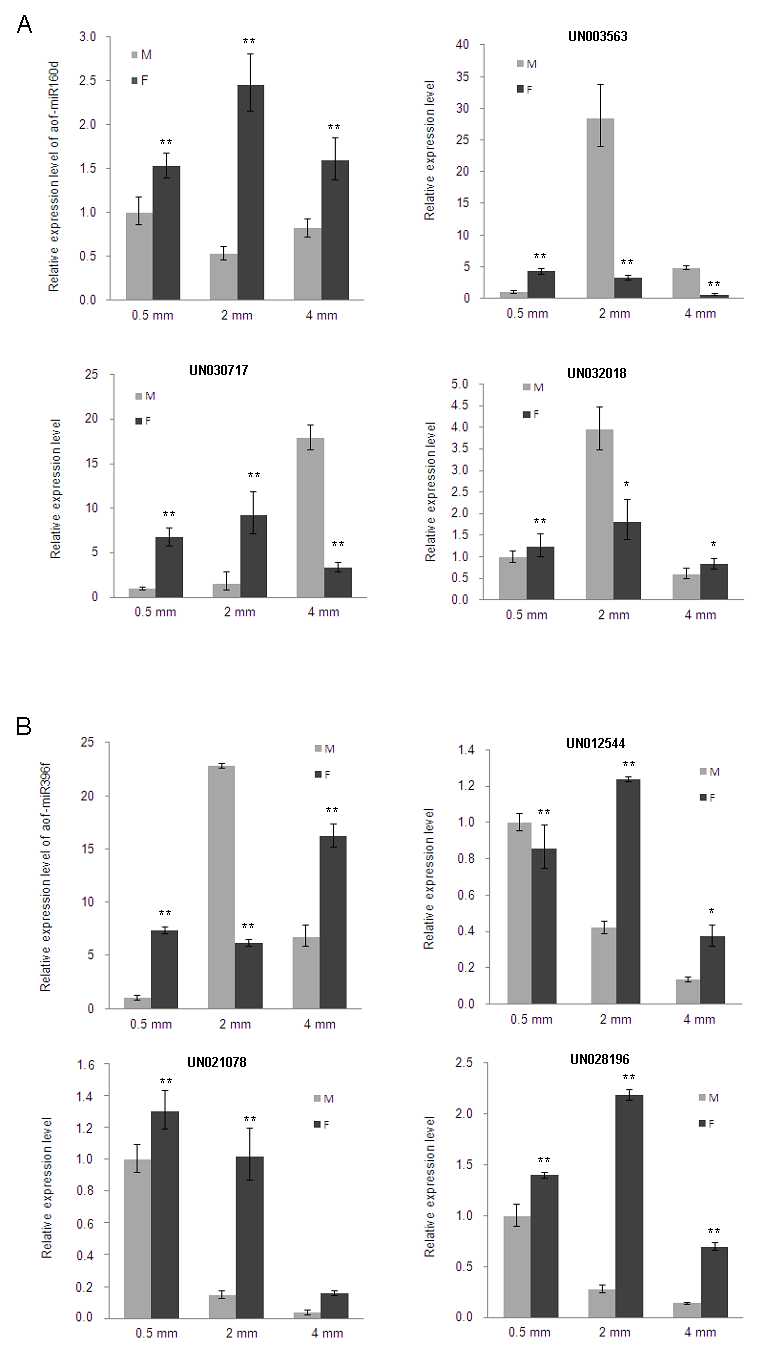


**Additional file 2: Figure S1.** Expression levels of aof-miR160d, aof-miR396f and their targets in male and female floral development. The targets of aof-miR160d and aof-miR396f were predicted from the unigene database created by RNA-seq. *Indicates the difference between male and female flowers at the same stage is significant (*P*-values < 0.05 or 0.01). F represents female flower, M represents male flower.
