# Supplementary material for: Identification of miRNAs and their targets through high-throughput sequencing and degradome analysis in male and female Asparagus officinalis
Source: BMC Plant Biol. 2016 Apr 12;16:80. doi: 10.1186/s12870-016-0770-z (PMC4828810; doi:10.1186/s12870-016-0770-z)
Supplement: Additional file 5: — Unigene sequences assembled using iAssembler in A. officinalis. (DOCX 7867 kb) [file 12870_2016_770_MOESM5_ESM.docx]

>UN00001

AATTTCTGTACCCTTTAAATCTTGAGAGGATCGCTTCCCATTTAATGCTCTTCAATTACTTCTTGTCTTTTCTTTTCTTTTTTTTAATTTGATTTTTTGCTTGAGTTAAGAGTCGTGGAGTCGGCGAGTTGGGTTTATTCTCCAAGTTTTTGAAGGGACGGATGGATAGAAGTAATAATACCTAATTACCTTCCCCTACGGGGGAGTTAAAGAAGCGGAGCTCTCATATTTATTTATTGTACAGAGATCTTTCTTACTTCAGTCAGTGGTCATTGAGAAATAAAATAAATAAAAAATATTAAAATATCGCTAAG

>UN00002

CTTTAACATTACTAAAAACCATGTCTTAAACTTGCACATGCTTCAATCTTAGATAAAATTTAATGACCATAAAAATGAACCCTGTTTACAGGCTAGTGATACTGAGCTCTGCTGCATCTGCTTCGACCAAGTATGCACAATCGAAGTTCAAGACTGCGGTCACCAAATGTGCGCTCACTGCACACTCTCCCTCTGTTGCCACAACAAACCAAACCCTACAACCTTATGCTTGCCCGCCCCAGCCTGCCCCTTTTGTAGAAGCAACATAGCTCGGTTGGTTGTAGCCAAGAACAAGACCAAGGAGGAGCTCGACAAAACAAGCCATTCTTTTAAGCTTAGACGTTCGAGAAGGTCCCGGAACTTTGGGGAAGGAAGCAGCAGCTTCAAAGACTTGTCGTCAGCCATGGGCTCTTTCGGTAAGATGAGTAACCGCGGTTCTGGACGAATAGCCGATAGCAGTGATAACCTGGACAAGCCTTAAGAGTGCTGGTTCATTGCTCGTGTGTAGATTTTGGTCATTGTAAGTGCAAAAGAGAAATCAAATAAGCAACCTTTTTGAGGAAGGGGGTTGATCCCTGTAGGGGAAACCGAAGCATATGCCCCTTCGTGTTTCAAGCTTTAGCGGCCCCCAGAGAGCATGGGCTCTGCTTGTTGTCTACTTTGAAACAGTTGTGAGATATAGATTGAATTGGATGGACGATTC

>UN00003

GGTTTTAACTTAGTTAACCTCATATGGGACTATTTTACACTACTGCAGGTGTTAGCTTCGGCACTTATAACACTGCGATAGTAAAGCAACTACAAGAGATTTTTGATGCAGATTGGAATTCTCCATACGCTATCCCAGTTGCGCCGCTATCAGCTGCATAAAGTTTTTTGATAGCGTGGAGAGGATTTGTGCATTCGGGTAATTATCTATAAATTGGTTCATAGTTGGAAATGTGGTTATTGAACAGTCAAGGTAAAAGGTGATGAGAGTTGGATGTTTTAGTGGGGTGGTATACTTCTTTGCGAA

>UN00004

AAAACCTTTTATTTTCGTCTATTATTTTCTGATTATAGGGTTTATATACAAACAATGAAATATTCTTCACCCTTACCTCTGTGAGAGTACAACAAATGGCTAGCTCATATCAGCCAACAATACAATAATTATTTACAAATATATGGCACGAAACATTTGATCTTCAATAACCACACAAGTTTTTCTTCTTTAAGGGAGGAACTGCTTCGTTGCCAGGGAATTCCCATCTAGCGATCAATCCATCCCAAGACGAGCTCACAAGCATAGGATAGTATGGATGCCAGCTGCAGTCTCTTACAGTTAGCTGGTGGCAGTCTAGTTTTGCAACGCGGTCTCCAGTCACCACATCATAGATATAAACACAATTGTACGTAGGGAACCCAAGT

>UN00005

TCTCCTCATTCCTCCCCCTCTATAAAAAGCCTGCGCACTCCCCCTCCTCAAAACATCACTCACTGGAGCAATAATGGCCTCATCCTCCCTTGCCCTCTTCCTCACCCTCATCCTCCTCCTCCTCCTCCTCGACATCGCCGCCAGCTCCTCATGCGGCGGCTATTGCCGGGGGGC

>UN00006

TTTTAGACGGTAGATATTACTATAAGTTTCACCCAGGAGTACTGCTGGTGATGACCCCCCTCACAGTGGCAAGTGATCTGCAATATCACACGGCAAACTAGTTGCGAGAACTAACTCAATACTGGACCTAGATCTGTATACAATCAGAGAAATATATACGGAGTAGCTTAGTAGTATACTTGATATGGTTGAGCTTCCGAGATATTAGACGAGACATAGCAGTAATATTATTGACCCAGAAGTTG

>UN00007

TGTCTCTACCTCTGCTGCTCCAGACCAGCCACCTATTTCTCTTGATGCAGGCAAAGTGAACTCAAATGGAGTTGCTAATCTGGCTGGAAATGGGAATAATGGATCCGTACCGCCGAAAAGCCAACAAAATTCGTCAGTGACATCAAATGGTTCATATGGTAGAGGTGCCTTGCCAAATGGGCATCCTTCCACTGGTTACCAGGATCCTAGATTCTCTTTTGATGGAATGAGATCTCCTGTACCTTGGTATGATTCTGCTGTATACTCTGATTCTCAGCACCGACTGGGTACAAATAATGCTGTTTCACATGTTACAAACTCTACGTCTGGAAGAAATCAGAACTTGAGACCTCTCCCT

>UN00008

GTTATACAACAACACCATTGAATGTTTCGTAATGAAATCAACTCACAAGTTGAATAAATGTGACATTGCAGAGTGAAATGGCGGCAAGAGCCATAGAGTATTGCAGTTCAGAAAAGAAACATTAATTTGTCTGTGTAATAATAATAAAAGGGAGCGTCTACAGTCTACAGACGCGGGTAAATTTCAGTTCTTGGCAAAGAGGCCGCCGAAGCCACCAAACTTCTTCTTGTTCGGAACGGAACGGAGGTGGTCGACTTCTTGGACCCCTCGGTAGCGCTCTTCCCTTCTACCTTGTTGAGGAT

>UN00009

GTAGGACGTGTACTCAGTCTGGGCCTTGCCGCCGCCGGAGGTCAGCGACCGGATCAAGGCGCTGACGGCGACCCTCCGGTCGGAGTTCGGTGGGCCCCAATTCGACCCGCACATCACCGTCGTCGGAGCGATCAGGCTCACCCGCGAATCCGCGACCCGGTATCTTAGAACCGCATCCGAATCGCTCTCCCCTTACGCCGCACGCGTCGCCGCCGTCGCACGTGGCGGGTTCTTCTACCAGTGTGTTTACCTCCTCATCGAGCCGACCCCCGA

>UN00010

ACTTACTATTAATTTATTATTTCCTTTGTTTTATTTGGTTAACAACAATTCCCGAGGGAAGGGATTGTTACAGGAGAACTCCTATGCAAGAAAATTACAATTAAATGCTCAATATACAAGATTATCACGGCTTAATTGAACTAAACAAATCATGTATTGAACGTTTGGGAGCTTCAGGACGAGAAACGATCTCCACAACCTTGTAGAAAGAATCTGGGCAAGATAGTGCTTCTACTGCAACTTCTGCCACCTGATCCCTCGATATCGAACCTTCACAAAGAGTATCCTCTGGCTCCA

>UN00011

CCGTAAAATAAGTTGAAGTTTATTGAAAATGTAACATAAATAGTTTGTTTAACATTACACAATGGAAACACGTGTTGGACACGACATGGCCTCACAATGGAGGCACAATGGAGTCTACATGATACTTTAGAAAATCAAACCAGATAAGTCACCCGAAGCTGACAATAGCACTTGTAAAGATGTCTTCTAACATACATAGTTCAAGGTTTTGAAGTTACTCTTTGCCGGTCAGGTAAAAGGCGACGTTCTCTTTGCTTAATCAATGCATCACACACTTTGGCAGCATCAAGACAATCTCTAACATTATGGACCCTTATGATATTAGCACCGTCGAGAATTCCGATCGTAACCGCTGCAACTGTGGCAGGT

>UN00012

AAAAGTTAGATAGAAAGCTGACATTAGGACCCACTTTTTTTACATTTTCCATCCTTTTTACATGGTAAAAACATACAACCAAACAGCCCCTTAGGGCTTGGAGTCACAGTCGTGCCATTCACCTCGGTCTACACTGTGAAGACCCATACTTGGGGCCGACCGATACAAAGCCTACATGGTCCTAAGTTCAAAACCGTCACACTATATAGTTAGTCTGCTTGCAATTAACGCAAAGGAAAAAAATAAACAAACATCGAGCCTTTCAGGCAGCAGTTCGTCCAAACAT

>UN00013

GAATCCAACTACAAATATACCAATCTATTAATGTAGCACACAAAGAATGTAATTAAGACCAGTCCTCTGTAGGTTCCAACTACAATTAAGAACTGTAATCAACATAAAAAACGAAAAAGCCCGGACGATTGACGACGACAAAGAAACGGGGATTCTGCAGACCAGAAGTTACAAATCCAAGAACAAAATCAAAGACTATTAACAGGTCAGCCACAATACACCGCGGTGAAACTCCTCTGAACGACAAAACGCGTAGTCACGAGCTCAATACTCCCACTTCTTCAGCTCCATTCCTTCAGGCGGGCTGCCTTCCACTTTCTTTTTCCCCACATCTTTCCAGTTGGTGGACAGCACCGTGCCATTGGACTCCACAAAGGATTTGCTCATGGCACGTCTAGTATCCTCATCAGCATCTCGGTAAATATCACGGAACAGCTTATTCAACGCTGCATCGCCATCAAGCTTCTCTTCTTTCTCCTCCTTCTTAACTTGCGCCTCCAGCTTGTCCCAATCTACCTTAGCTTTGGAAGATGGGTAAGATGGTTTCGTCTGTTTTGGACCTGACACCGCATTTATCTTCTGTGGAACATTTCTCTTGTCACTAAATTCCAAAGATGTCCATGTGATGGCTTCAGCTTTGAAAAGGCGAATTTCAATTTTGGTGGAAAGTACCTGATACCTACATTTCTCAGGTACAATCTTTCCAAATAATCGTGGTTGAAAATGATAGGGTTCTTCACCAGGAACGTTAACAACAACACTCAACGTTTGTTCGCCAAAATCTACTGATACATTTCCAGGAGGGATACCTTTAGCAAATATAGTCAACACAACTTCTGTAGTAGAATTATAGTAGTCATGTCTATATTTCGGTTTGGCAGGTGCTGCTGCTGCTGCTGCTGTAGATGGTTTGGGTAACTCCTGTGCGGACCCAACACTCACAGAAGCTCCATTTGAAGCAGGGGGGGCATTGCTCACTTTCTTCGAAAAGCTCATGGTTTCTTCTGCAATTTTCTCATCGCATTCTTTAATTAATTTGGTGAATCTTAGATCTCCAGGTGCCAATTTCAGGCCTGCTTCAAGAGCTACCTTCGCAGTCTGATGCTCTTCAAGCTTGACGCAAGCAGTACCCTTACGCAAATATGCTTTGGACATTGAAGGATCAAGCTCAATTGCTTTACTTGCATCGGCAACAGCTTCGGTGAAGCTGTTGATCTTGATGTTGGCCTGGGCTCGATCCGCGTATAGATCAGCGTTCATAGGATCCATCTCGATCGCCTGAGAGTAGAGGTCGACGGCCAGCTCGAAGTCGTCGTCGACGAACGCCTCCTTGGCTTTCTTCTCGAGATCCGATGCCATTAGATTCAGAGAAGGAATCAGAGAGAGATGAGACCAAGCGATGACTTGGAGAGGGAGCGCTGCGCAAAAGAGGGAGCC

>UN00014

ACAAGACCAAGTGAAGAGGACTGCTCCCGGGGCATCCATATCCACCAGTGGGTGCACAAACAAGAGCTCCTTTATCCAGTAAAATATGAACGCATTCTGGTCTCCTTTGCCTCGCCGCTAGATGCAACGGCGTAGCCCCATTGTTATCGCTAACATTTACAAATCTAGAAAATCCCCAAGAATCAGCAACTGAGGTTGAATTGGCAGCAGATAGAATTGCTTCTAAGCAATCCGAATGGCCGTAATATGCAGCATAATGCAAACAACTTCTACCATGCA

>UN00015

CTACATGTAAAGGGGAGATTTTTTTTTAAGTTAGAAACATCATCCATCCCAAATTTCTCCACATTACAAGGATCCGAACATCACCCCATGCTTAATAGAGAACTGTATGCAAAAGATTGTTGTGGAGATTTAAAAAAGAAAAATCCCAGTCCTTCGACAAAAATCTTCTAGTCTTCATCTTCAATCAGTAAACTTGATTTTCTTTGGAGCAGGCTGTTTCTCGCCCACCAGTTCTGGGCATTCATAAGTACAAATAGACCAAGAACAAAGAACTAAA

>UN00016

GAAGACTATCTTCATTTGCTGTTGATCGCAAAGAAAAGGGGCTTGGGAGAAAGTTAGTTGTCGGTAAAATTGGGGGTCAAGCTGTTTCTTGTATCCCACTTAGACTTGTTTTCAGTAGGTTGAATGAAGCATTGAGCAGTTCATCACGATCAGCCATCTAAGCTTTTGTTGTGCCCTGAGACCGAGATTACTCACTGGATCTTTTTGAATCTATGGAGGATGAGAATGATTGCTAAGTTGGAGATTGTAAATTTTTGTGGTCCTCTTGTTTATATGTTTTGTAACCTGAGTTTGTGTCGAG

>UN00017

TAATTTAGGTAATCACAACAAATTAAAAATAAAAAATTAACGAAATTAAAATCAATAGTTTGATTTTTTTAATATTATTAATTATAACAAACAATCATGACGAATCGAGAATAAATATGTGAAAATTTGTGATGAAGAAAATGATAAATTAATGTTATAATTGTCCAAAATGCTTGTGTTGATTTATGCTACAAAATTAGAAGTCGAGAAATTGAGAGAATTTATAACTTAGAATATCAAAAAAAAGTGAATCCCCACTAATACTTAGTATTTAAACTTTAGACATTAATGG

>UN00018

CCTAATAATTAGAAATCTTATTCCACTGCATATGAAATACAGCGCTACAAACGAAACACACAGACACTGAAAACACACAACATAAGCATACAACGCAAACACAAGTTATTAATACCACTCAAGAGTACTCCCAATCTAACACTTTGAAAGGGAAACTCCACAAGTAGAAGCAACCTTTTTAGCATTGCCAGAACTAATGTAACCCTTCAAGCTTGGGTTCCTCATGTATCCACAGAAGCAAGGCTGCTGCTTCCTCAGTTGGTTGCAGCATCCATTTGTCGGTGGTGCACCACCAGTGATCGATCCCAAGCAAGGGCTCAGCTCGGCCGGGTTGCAGGTCTGGGACCGGACAGTGGTCGGAGCTTGGCTCAGGAGGAGGGCTGGGATGAAGAGGAGGAGGAAGAGTGAGGACTTCATATTGTTG

>UN00019

TTCGAAGGTCCGATGTACTCGGCCTACGCCGAGCTCAGAGAATGGAAGCTCAGAACAAAGAACCTGAAATTACGTAGCAGCCCTCTCCCTCCTCCAACTCCGTCAAGGAAACCAAGCCCCCCGATGGTCTCACGATCCATCCAAGATTTCTCAATGGCCTTCAGGAAAAGAAAACAAGAAGCCCCCTTCAAACCCGAAGCGCGGGCACGCGCCATTCGGCACCCCGCCTCCTGCAACCCCGAAGCACGTGTCGACGAGGGCATCAGGTGGGGTTATGTGCCTCCCCGA

>UN00020

GATGCACTCTAAACTTTTACTAACAGTTTATAAATGGTTTACAAACACATCCGGTAACAATTTATAAATGGTTTAACAAATACATTCCAGTAACAAAAGCTACATAACAGCGTGTGACATACAAACGAGCTGTATTTTCTCTACTTTCAACAATTTTGATGATA

>UN00021

TACTAAACTAAAGTTTAGTACTAAAAGTAAACTTTAGTTAATTGATGAGAAGGTTCAACCGACTACTTTGGTACCAAAACAAGTTGGAAACATGTATACTGCATCGCTCTATGCAGCCTTTGTATCTGTTCTTCACAATAAGCATAGCACTCTGGTTGGTCAGCGGATCGTTATGTTCTCGTATGGTAGCGGTTTATCTTCTACAATGTTCTCATTTAAGATCCAAGATGGTCGGCATCCCTTCACTGTATCAAATATTGCTAGTGTGATGAATGTCGATGGGAAGCTAGACTCTAGACATGTGGTATGTCTCCTGTTACCTTTTTGCAAGTGAGCCAGTTAAAAGTTTCTCAAAAAATTTATGTGATGTTTTAAAAA

>UN00022

CTAGAAAACTGACAGTCAAGATTTTTATGACTTCATCACAAGTTTTCAATTCATTGGCTGAAAATATAAAAGGCTAAAGTTAATGATATGAATTATTTGAGTTCACAACATCCACATCTGAGTTGCGGTTTATTACAGAAACAGAAGACATCAGTTCTCTGCGTTTGAGCTCAGAATCTTCAGAAGACATTGATCCACATTGAGAGATGCTCCTAACACTTGTGGAGACAAAGCCTCAAGAATCGATTCCTTCCCTGTTAAAAATCCAAACACAGGCCGCGGACTCATGGTAATTGAGAACCACTCAATCCCTTCCGCCTCCGGCAATCACAGAAACCACGGCAAATTTCGGAACTACGACAAGATCTCCTTCTTTCACCTCCGCGTCTAAAATACACTTACCATTCTTACCTACGATTTGAATCCCTGA

>UN00023

TTAATTTTGGGTATCAGTTTACCAAAGATTATTTTCTACAAACCGGACAAGTCACTGGTGTCAGTATTTCTTCTTATAGATGCTAGCATCCCTGCAAAGAAAATTGATCTGGAATATGCTTAGTTGGCTTGGCCAGAATGAGATACCAATGACGCTAATCTTCACTAAGTGCGACAAAAGAAAGAAGAAGAAGAATGGAGGAAAGAGACCTGAAGAGAATGTCGAAGATTTTCAGAATTTGATTCGCGATTTCTTCCAATCTGCTCCACCTTG

>UN00024

GCAGAACTGAATGCTACTCGTGAGAAAAATGGAGTCTACATACCAAAAGAAAGATATTCACAAGAGGAGGCTGAAAGAAAGGCAATGGCAGAGCAAATTGAGCAAATGGGGGTCTCACTAGAAACCCAACAAAAGCAAATTGAGGATTTACAGGAAAGTAATGACACACAACTGCATCTTGTTGCAGATCTGAGAAAACAACTTGATGCAACAGAGAAAAGCTTGAACCATACTAGCAAGTTATTAGCTATTGCAAAGGAGGATCTCCAGCAATCCCAATATACCCTGAAAGAGAAAGACTTCATCAT

>UN00025

AAATTAAAATATGTACGTCAATAAATAGTAGTGAGTGGCACTAAATCAAGGTTTGAGAGACAAGTAAATAATTTATTTCGCTCTCTTGTTAAACAAAACAAGAGTTGCAACCATCGCAGCACCTAATAATTGAAGACTTGGACCACTTGATGAACATATTCAGAAATTATGCTAAACATGCAAGCAGAGCTATAGCATCACAATATTTTGGGAAAATTTAAAAAAAAAAACAGGAAAAGAAAGAAAGGGGGCAAAAAAGAACACGACTAGAATCAGCAACAAACGAACCCTCAACAAAATCTTCCTTGCAAACCAGTGAACTTCTCAAGCCATAGCATCTGATCTAAAGTTGACGCTTTTCCTGTCGAACTAGAGAGGAGTTGAATCGACTAAAGCGTCCAGCATTCTCTTTTCCATATTCTCCTCAAGAAAACCCAAAACTAGAACGTACTA

>UN00026

GTAATCAAAATATAGAATAACATTGATAATTGATTCAGAAACTGTTACACAAACAACAGAACTTGATGGACCATTGAGACCTTACTTTTTTTGTAGCAGACAAAAATTTAATGCATACATTATTATAGTGTAAAAGAAACTGACCTAGACTTATTGTCCATCAGCACAAATTGCACGTAAATAGAATAACTGGATTATGTATGATCTCCTTTGCTTGAAAGCCATGATCATGGAACAGAAACTCAGCATGAAA

>UN00027

TCGATCTTATCGAAACCCTAATCCTTGACAAAAGCTCTCGATTGCGTCCTGATTTTGTTCGAATTCTCTTTGAATTCTCTTGCGAGTTTGATCCAGGTTGCATCTCTCTTGGCCATGCGACCCCTGATCTCTGCGATCGACCTCTGGAGAGAGGAAACCCTAGGTTGTTATCGAGACCTCAGAATTTCTCTGTGTTTTCAGTTGATTTCTCGTAATGGCGGCGGAGAATCATGCTCCAAGCCCTAGAGGGCTTCTTTGCAACGCGGGAGCTGGCGCTGCTGCAGGTATGCTCGCGGCTACTTTCGTGTGTCCCTTAGATGTGATTAAGACTAGATTTCAAGTTCATGGGCTGCCTAAGCTCGGTAATAGTAGCGTTAAAGGCAGTCTTATAATGGGGAGTTGGTAACAAATAGTTCAAAAGGAAGGTGTGCGTGGCATGTATCGTGGCCTTTCACCGACTGTGCTAGCATTACTTCCAAATTGGGCTGTGTACTTCACTGTCTATGAGCAACTCAAAGATCTGCTATATTCTAATGATGGGGATCATCAACTCTCTGTAGGTGCTAATATGATAGCTGCATCTGGTGCTGGAGCTGCGACAACGATCGCGACTAATCCACTCTGGGTGGTAAAGACAAGATTTCAAACACAAGGAATGAGGGCTGGCGTGGTGCCATATCATGGCACGATTACTGCTTTAAGGAGAATAGCTCATGAGGAAGGCATTAGAGGATTATA

>UN00028

GTTTCTTAGTTTGTTAAGTTACTAATTGTAAGTAAGAGTGAAAAGGTTCTTCTTACTGTGAAACCACAATATGGATTTGGTGAGAAAGGCAGGCCAGCTTCAGGCGAGGAAGGTGCTGTGCCTCTGAACGCAACTCTAAATATAGAACTTGAGCTGGTCTCTTGGAAGACTGTTACAGAGGTCGGTGATGACAAGAAGATCCTGAAGAAGATCCTCAAGGAAGGGGAGGGCTATGAAAAGCCAAATGATGGCGCAGTTGTTAAAGTGAATTAACCGGAAAACTGCAAGATGGGACTGTTC

>UN00029

AATCTCGACTTCATGGCCGTTGTAGTTCTCGTTTAGGTTACGGTCCACAACTGAATCTAGCTTCTTATTTCGCTCTAGCTTCTTCACATGATCGAGCAGTAAAACATCGTCTTCATCCTGCAGGCGCGAGAAGTCTATTGCACGGGTTGTCCTGTGACAAGTTCAAGAAGCGTGACACCATATCCAAAAAACATCAGTCCTCTCAGATGATTTTCCAGTAGATAAATATTCA

>UN00030

TAAACAAATAACAAAAAAACCCTAAAGAACAAACCAATAGTACTTTAAGTACGACGAACGAGGATTTCAGCACATTCTTCGTGTTCTCAACACGAACGTCGATGGAAAGCAAAAGATCATGTTCGCTCTCACATCGATCAAGGGTATCGGCCGTCGATTCGCCAACATCGTCTGCAAAAAGCGGATGTTGACATGAACAAGAGGCCGGAGAGCTCTCAGCTGCTGAGCTTGAGAATCTGATGACGATCGTTGCTAATCCTCGTCAGTTCAAGATCCCGGATTGGTTTTTGAACAGAAAGAAGGATTATAAAGATGGGAGGTACTCTCAGGTTGTTTCCAATGCTTTGGATATGAAGCTCAGGGATGATTTGGAACGCTTGAAGAAAATCAGGAACCACCGGTGGGCTGAGGCACTACGTGGGGCCTTCGTGTC

>UN00031

TCAGGGACGAATCACCTGCATGTTTTGTTGTTTAGTGTACTTTCATCATGTATTATAAACTGAAATAATACATGATTGCTCTATTATTGAGCAAAATTACATAACTAGCTGTAAATAGTTGGACAAGCTTAGTTGTTATGTAATTTTTCTTATTAAACTAGTGAAAGAGCCAATGCACACATGTTGACCCGTACAGATGGGAACATCTTGGATGCATCTGTGCATCATATAGTTTCAGAAGTTGTTGCCTATTGAAATTGTATTTTGACCTTCATGTTGATAGAAAACATGTATTTTACTCCCTTGAGATA

>UN00032

TTCTTTAAGTTGGTACTATTCCTACTTAAGTTTTCTTTAGTCCATTGATGATCACTTGATTGAGCTGCTTTGTGGATGATCCGCTCCGTTAAACTTGATCTACATATTCTAAAACCACAACACACCAAATTTGGGGTGAACCCGTCGTGGTTTGATCCTCCGTCGAGCCAGGTAAAAAGTTCTATTCATTTGAAACCGTTAAAATTGATACGAGTGGATGAATGGTCAAACCAATGTTACATTAAACGTTGGGCGCTTCTAGGCGTCGGACTTGGCGCCCAACGCTTAGTCGCCTAGGCGGGCGCCTAAGCGGTTTCTAGGCGTTTTTTTTTTA

>UN00033

GTTCGTACCATGTCGAACCCGGTGTACGCCCAGTACACGACGGCGGCGGCCTGGAACACGCCCTTGGCGCCGAAGGGCGCAAACGGGGCTAGATTGGCCGTCTTCGAGTGGGCAGACCCCGCGGCGATGACAAAGGCGATGACGGCGACGCCGACGACCGACGTAATCCAATTCAGCGTCGAGGTTCGCCTCGTGCCGAGGGCGGCAATCGAGGAGACAGTGGTGAGTACCACGACGGCGATCGGGTCGAGGAGGTTGTAGCCCTCGGCGAGCCCCGGCGCGCGGATGCGGAGCGCATCGGCGTCGAGGTTGATGAGAGTGGAGAAGTACGAGGTCCACGAGCGGGCGAGGCCGGCGGCGCCGACGAGGGCTTCGAGGAGGATGTTGGCGGCGGCGATGTAGGCGACGAAGTCGTCCGTA

>UN00034

GTTTTCTTTCTTCCTTCTTGCCGCTTGCTTCCCCTTCCCAAACCAACGAAGTCGGAAAAAAACCCTAATTTCCCCCGTCACTCGCCCTCTCCAACTCTCGATCTCAGCCTCTAAATTCACTCGGATCTACCGATCGACGTCCAATTTGGGTCGAAATTCTCGACTCCAATAGCGTAAATCCTCTGCTTCCCAATCTCGACAGCTCAAAACCGCCGCATTTTGAAATGAGCCTTCAGATTGGGGAAATCCCCAGTGCTGGAGCTTATTGGAGGTAG

>UN00035

AGGTCTTTCGGGTATTAAAATTTCCCCTAGACTTGGATTCTGGTGTCTTCTTCAATCGGTGAAAGACTAAATCGGCAACGCGTGTTAGGCGTTGACTTATCCCTACTTAGGTTTCAGGATAAAATTCCTCATACGTGTAAGGTACGGATTATGATGTTATTAATAACAACCTGCCACAATATCGAATATGTGGGCTTTTGTCCTTTTTTTACGACAAAAAACTAACCGGCAACGCGTGTTAGGCATTGACTTATCCCTTCTTAGGCTTCGGG

>UN00036

CCTAAATTACAGTAGTTCTGTGAATACAATAACCAAGCAATCAACAACCCAAATTATGTAACAGATCACTGAAGTTTCACAAATATCTCCAAAAATCATTCGGCACCCAAAACCCCAGCTCCGCAAGGGATGCCAATTTGACAAATTATCCACAATTACATAGAACAAGTCCATCCCCTTGAACAACTAAT

>UN00037

TTCTAGTCTTTTTAGTCTTTTAGTATTAGTTTTCTGTAGAGAGATGGCTCTAATTCCTCAAATCTTCGGTCAGAGGAGCAACGTTTTCGACCCGTTCTCCCTCGATGTCTGGGATCCCTTTCAGGGCTGGCCTTTCGACTCATCTCGCTCTCTCGCTGATCGTTCTGGTGCCGGCGGCGCTCTCAGCGAGGCCTCTGCCTTTGCGAGCACCCGCATCGACTGGAAAGAGACGCCCGAGGCTCACGTCTTCAAGGCCGA

>UN00038

GATCTTCCTCCATTATTTCTTCTGGATCCTCCTCTTCTTCTTCATCATTTTCATCATCAGTCTCAGATTTGTTTCCTTCTTCATATTCAACTTTGTCAGCACCATTATCTGAGTTTTTTTCTATTTTAGGTTCACCTTTCTCATCTGCAGACCCACGCTCACCCATTGTTTCATCATTATCTTCTGCAGCATCTTTTTTTCCAGATTTTTCATTTTGCACTGGAGACTCTTCAGTGACCTTAGAACGCTTTGATGAGCTCTCTTTGTTATCCCTTTCAGATTTTTCATCTCTTTGTCTCTTGCGCTGGTTC

>UN00039

AAGCGAGAAGGAAGTCACTTAATCAACAAATACATCTGAAATTGTAAAGGGAAAAAAATCATATAAATGCAATAACAACACCGTAAATGTCTACAATTAAGTTAAATGTATGCACGTAATTTACAACCCTTGAGGACATCACAAACTATCAAAAGTTTGTCCTGGGACAGCGTATTTTTAAACCATGTAAATAAGTGACAAAGCTTAGCTGGACATTATCATCCACCACTTGTACAGACCAAAGCTGTAGATTATAGTGATAAAGGCAACCCTTACAGGGTAAAATTTGAACCTTCCTTTTTTTTTTTTTTAGTAAAAACTAAAATTTATTTATCAGCGTGATACG

>UN00040

GTAAAAGGGGTAAGGTTCAATTCTTACGCGATTCAAAGAAAAGCATGGAACTGAGTGTAACTGAGTTGCAAAAGAATCCATTAAATTATACTCAGGTTTCTGTGCTTGCTGATGATATCCTAAAGAATGTGGAGTATGATGCTTTGAGGATTGTTTACAACAAGTTCCTATTCAGTTGTCCAATTCATCCCGACGATTTCAACCATACTATCTCCTGAGGTGGTGGAGAGAGAGTCTGAATCTGGAGGAAGACTTGGTGATCTGGATTCATATGAAATAGAAGGGGCAGAAACCAAAGCTGAAGTACTTCAGAATCTTACAGAATTCCAGTTTTCTTGCATGATGTTCAACGCAGTCCTAGAAAATGCATGCAGTGAACTTGGAGCAAGAATGTCGGCCA

>UN00041

CTACTACATCAAATTGTAGAAACATTAATCAATTAATGGCAAAGATGACTCCTCCTCCTCTCCTGATCCTCATCTTAGGCCTTGCCATCACAACACAATATTGCTCTGCGGATGACGTTCTCCTCTCCCCCGAGGCGCTATACAGCGGCCGCTCCCTAAACTATGGCCCCTACATGTTCACAATGCAGAACGACTGCAACTTGGTTTTGTACGACTCTGGAAACCCCATTTGGGCGAGCAACACCGGGGGGCGAGGGTACAACTGCCGTTGCGTGATGCAGCGCGACGGCAACCTCGTTGTCTACGACCAGAACAACAACGCGGTGTGGGCCAGCGGCACCTCGGGGGGCGACGGCAATTACGTGCTCGTTCTGCAGAAAGATCGCAATGTGGTGATCTATGGACCGGCGCGTTGGTCCACTGGTACGAATTATAGAGGATCGCGTGGAGTTGTCGTTGTTGCTGCGCATAATTCTACAGCTGCTGCTGCTGCGCAGAATTCTACAGCTGCAGTTGTTGCTGCGCAGAATTCTACAGCTGCAGTTGTTGCTGCTGCTGCTGGGGAAGTGATTGCTGATGTCGGTGCCAGCGATGCGCGTTGATTATATTTGTTAGTAATATTGAGGGATGCTTGCTGAATCTAGTTGTCTGAATAAAATGAGATGAATGCAGGCTAGAATGCATGCTTACGCACGTGGTGTTTTTTTAATTTCGTGGATGCAAGCGGTGTCATCGCTGTACTTCATGTCCACTGGCATGAGGTCTAAATAAAGGATTGGGATCCTCCCTCTGTGGACCGAATTCGGTCAATTCATCAATCAGATGTTTACTAGCATCAATCAGTCTTCACAATTTATTCACAAACTCCAAAGCAACAGGAGGAGCGACATCAAACCTATCTACATGCACAACACAGAGCAGCATTTTCATGACCCGCAAATACAGTTCAATTATATTTGTAACTAGATATACATTATATTATAACCTGGATAGTATGTTTAGAATAAAAGGAAGGTAATATCAGTAGAGATTGTGACTTGCGTCCTCCTCGCCATTTAGCAGTTTGTAGTACAATAACAATTAACAAAAGAACCCCTAAGAAAAGAACTAAC

>UN00042

AACTTAAGTTTTTCCTTCTTGTAACTTAGTAAACACAGTCACCTACAAGCATCGAGGCTACAAGAAGAAAAGTTCTAATCACCTACACAAGCAAACTAATTACACATTCACCATCTCAACAAAAAAAAGGAAAAAAAAAAAGAAATTCATCCTGAGGGCGAGCACAAAATTAGAAAATGGTGGTCGACATTCATTTGCAGAAAAAAAAACATGAGGATCACGAGACCCGAGTGAATGGATGTGGTTAACCTTGTTTGATTTCCTTCATTCGT

>UN00043

TTTCAACTTAAATATGTCACACGCTTAAGCGAAAATAACAATTTTAGTTATCTGTATTTTATCACATGTCAGATATCAGCATGTGTATATGTGTGCTGTCGTTCATACCTACAGTCTTATATTAACGTGTGTGATCTTATATCTACCCCACCAATGACAATAAATAAGTACGAGATTCCATCAAAGCCTCTTCTAGATGGTTAAAAGTGTGGCATTGTAATTTACAAGTTAT

>UN00044

GACACATAATCACTTCTTAAAACAGATACGCATCATACAAGCCTTGATCACACAACAAGAGTTTTTTTTATTTATTTTACTGCCTCTGCAGAGATGATTATGGACAAAAAATTTCCAATAAAATTGATTTACATTGGTCACGGCATAGCCACCAAATACTGCAACTTTTCGAATAACGTATACATTCTGCATCCTCAAACAAACTTATGACGCAGTAATGTGACTGTTCTCTGCCAGGATGCTGTCCAGTAATGTGCCGCAAGCATCTTCCAGTAAATCTAAAACCTTTTCAAATCCTTGGGGGCCTCCATAGTAAGGATCAGGGACTTCAGTTTCCTTGTGTTTTCTACAGTAGGAGCACATTAGCTTAACCTTTTTTGGGCATCGGCTGGAAGTGTACTTCCTTTAAAGTTCTTTCC

>UN00045

AGTTAATTTGGTTAAGATTGGGACCGATATTGAAGATTTTAAGTGCTCTTGGCTTATTGTCAAGCCCTTGAGCGCGCCAATGAAACCAAATGAAACTTCTATCGATCCCGCTTGTGTTACAAAAGTGAAAGCCGTTTACTAATGACCTGAATCTGCAGTCTGTGTTCGCGGAGTATGAGGCTGCAAGCTATGAACAGCTGATCTCTTCCATTGAAGCTCAGCCCAGCAAAGCTGTTCAGGAGGTGTTGAAGTCTTTCTTGCACAAGATTTACAAGAGACAGAAGTGATCTGGCTCAGAAAAAATCAGTGGCAAGTTCTTCATCATGGGTTTGTACTTTTTTTGTTGTCATTTTCTTGTTTCTCTTGATTTCTGTAGCAATGGCACGTTTCTATCCCTGTGCTTTAATTTTTTTTTCACCATTATTCTGTATTAGATTAGATTGCCATGTGTTCTGTATGGGTGTTCTATTGTTGTGTTTTGGAAGATTAGACCAAGTAGTAAATTAGAAACAATAACTTTAAGTAAAC

>UN00046

TTTCCCGTTCCGTTCTTAATTCTTCCTAAGTCTAGCAGCCGCAGCAGCCATGCCGTTCAAGCGCTACGTGGAGATTGGGAGAGTGGCGCTCGTTAACTACGGAAAGGATTATGGCAGGCTCGTCGTCATCGTTGATGTCCTCGATCAGAACAGAGCTCTGGTCGATGCCCCTGACATGGTACGTGGCCAGATGAATTTCAAGAGGCTAACCCTGACTGATATCAAGATTGACATCCCAAGGATCCCGAAAAAGAAGTCCTTGATTGCTGCAATGGAAGCTGCTGATGTGAAGACAAATGGGAGAACAGCTCCTGGGGAAGAAGTTGATCGTGCA

>UN00047

GTATTAACCTTTTCTTTATTTAATCAATAAGAACTGGACACAATAGATAGAATAATCAAGCATTTCACTGATATCACATGACAAGATGAAAACTCTTATACTGAAAACAATAACCTAAACATCAATTACCAGAAAATAAAACCTCAATTCAAATTTACTTCCATAACCCAGATAACAAAATTGAAACTTCGGAACACAGAGGTCTCTTACAGTTGTTCACCAAGAAGCTCATCCCTACTTCTTCGTCTTCATCCCCTTCTTGCTCCCACCATTAACCACATCATCCGGCACGACCTTCAACAGCTTCCTCTCCTTCGACTTCGCAATCTTCTTCAGCGTCTTCGGGTTCGTAATCTTCTGCAGCTTCGTCCCAGACTTGAGCACATTCTCCTTCTTCTTCTTCTCCCTCTCCTCCCTCTTCTTCCTCCTCTCCGCCTTATTCGATCTAATCTCCTCTTTTAATTCATTCACCCTACTACCCTAA

>UN00048

GTTATTTCAAACTAGAAAGTGTGCTGATATCAAAGATAGATCGATCGTCAACAAAAAATGGGAACAAAAAAATAATTAGCATTACAAATTATCGGTGTATTATGCCCCAAAAAAGACATTATTCCATGAGAATAATTAACAGAAAATTCCAGCAGTCACGCTATTTGCAGTTCCATCTCAGTACAAAAAAAAAAGCTGTAATGGATCTTAGGGGAAGTGGCTCTTTAAATACTCCCCATAGCCTTTGTCTACAACTATCAAAACCCATACTTTC

>UN00049

TCTCGTCACTTCTTTTTATTTTTAATGGCGAAATTAATATCTTTTATAGGATCTACTCCGATCGCAGCTCCAGCAGGTTCCACCTCCGGCAACACCGCCAGAGCCAACCTACCAACCCTTAGTAAACCCTCATCAGCCTCCTCCTCACCTTAGCCATCTTCTTCTGAGCTCCACCAAAACACTTTCATTCTTTTTACATGTATGTGACTTCCTACCTACACATAAAAAAACGACCGTTTAATAATTTGAACACTGTTTTGATGTATTATTGTTATTATTATT

>UN00050

TACTAATACAACCTTACTTTAGGCCAGATCTTCCAACCTTTTCAGAAAACCAAGTTCACTGCGCGCCCCACCCCGTAAGTACTGTGTTGCCTACGCTTGCGCCCCAAAACGGTTGCTGACTTTGTACAAGTCATCCGGGCGCGCTACGGCAACACAGACCTAACGACTTAGACTAGCTTGCTGGTTTTCCATCATCCAATCCACAACAAATCGAATGAAACCGCTTCGGATTTCTTTTGATTCAAACCCTTGTTCCGCTCTCGCTCGCCGCTACTAACGGTTTCTCTTTTGATTTTCCTTCCTTTAGCTACTCAGATGTTTCAGTTCGCTAAGTTTGAAAAAGTCCAAAGAGCGCAGACTAGCCACGGAGCTTGGATACGGTTTCCCGATCGGAGATCCATGGATCACAGACGCTCTCTCTCCCCATTGGTCCTTTTCGTCCTTCTGAAAGCAAGCGTCCTTCCTTCTCAATGCCCGGGCAA

>UN00051

GTTAACTAAAGAAATATACAAATATGTAGAGTAATCAAAAGTTATCTTTCCTTTTTTTTAAGTAAGATCAAACATGAGCATACTTAATAGAGCACCTTATTACATCCAGGAACCAGAGCTTGAAGAAATTTCATCCGCTCACTGATCTTTTCTCGCCTAGCCTGTTCACGAATAAATTAGTCAAAGAGATGGTTAAATTCAGTGGTTCAGCTGTACCTTTCACCTGAAAGTAATCTCTATGCTATAACTCCACAATCATATGCTGAATAGTACAAAGAGCCAGTACATCTA

>UN00052

TGAAGATATCATCACATAATTATTTCGGCAAAACAAATAAGACATAACCAGCCTAGGGCTCAGTCAAGTCACCTTACATGCGACATATATTCACATGCACTTCAAAGTTTCTATTCCCTACAAGGATGGCTCAAATCAAGAGAGAACGCTAATCCGATGGCCTCGTGATCGAAAGAACCATAAAGCTTCAGCAAAAGGAAGACAAACTTCCTAGACTCCGAACACAGACGCAATTTCCCTAGTTCATCTCATCTGAAGCAAAAGTCTATGCTTTGACACGGACCACAAATGAACCTCTCGCAGAGCTGGTTGCCGACCTGGTAACCGATGGCCTCGATTCGACGGGCGGCGAGGTCGGGCTTCACGGAGGAGTATGCGGACACCATCTCCGTTATCACCCCCTCCATGCAGCTCTCGCACACCTCGCGATACCCCTAAC

>UN00053

TTTGTACTCGTGTCCGTCCACCGTCAACCGCCGCGCCGTCGCGCCGCCGCGCCGCCGCGTCTAAACCCTACCTGAGCTTAATTCAAGCGTGCTTCTTTGAATCTGAACCCTAGTTCCTCATCCTGCTTGGGTCCAGGCCGTGGATCTCTGATCTCCGTCGCGGTTGCCGGCGAAGGAGGCGATCGGGGTTCTATTTGACCAGATCTCGTTATCTGGTGTTTCAGAGATGAATGCTAGTTTCTCAACAATATGCAGG

>UN00054

TAACGTTAAGGTAAGGAAGGGTGGTCAGGCGACCTCTCGGGGTTTCACCCGTCGGTGTCGGGCAACATGTTGTGAGGGAGCTTCTAGGGTTTGAGGGTCCGGGTTGAAGATGCATGGTGCACTGTGATGGTAGGTAGGTAGGAAGGCTACATTTGTTGTTCTTGGTTTCTCTCTCCTGT

>UN00055

TAGCCGTTGTAATTTGCATGCTGGATTGGTGCCAGAATTGGGTGCCAAATATTGACTTGTCGTGGAATGAGATTGGAAGAGTATTGAAGGCAGGGTACTATTATAGATGATTTTGGAAGAGCGATTTGCGTTACTATCTAGTTTCTGCTGACATTTTTCTCTGAACTTCTTTCTATGGTGAATTTTCAGCAAAGAGAGTCTGATAGCTTAGACTGAAAAGGACATGTTCAAGGCCTTGAAGGAGATTAAGTCAGCATTAATCCATCAAGTTTTTATGGAATTTGATGTACGTGATGTCATAAAACCGTCGCTGAAGGTATTGAAAAAAATAAATTCCTCATACTGAAGTAGTGCCCATCCTATGGGATGCACTAATGGATACAATTA

>UN00056

GTACTTCAGGTTGAGGTAAATTCCTGTTAGCTTTAAAAAATGCCGCAGAGAATGGAGCCGGGTCAAGTATCGTTATCGGTAATATCCTCATCAAGGATAACATATTTGGCAGCTCCATTAAAAGTTGAGGCGTTTATTTCTCCATTCCCACTTGGATGACCTTTTTTTATTTTTTGATATTTGTACCAGTTGAATAAACTAACCCCAACCATTATTGTGAAAAGCCCAATTCCTTTCAACCAGGTGAACTTGGTCATGGAAGTAGAATACAGCGACCTGAAAAGCCCTTTCATTAGTTTCCAT

>UN00057

TTATTACTACAGCACAATGAACTTCCTCAACTATCCCCACAGGTACCAGACAACGGTCCTTGGAATTATAACTTCATGCCGGTGAAGCATACGGTGAGCATGAGATATGGTGTAAAGCTTGGAACACCTAGGGATTATTACCATGAAGATCATAGACCGACGTCTTTTTTCTTGAGTTCAGTTAATTTAGAAGAGGGAGAGACTGCTGAGGCTGATCGCGAGGATACCTTCACGTAGTGATGGCC

>UN00058

CGACCGAGATAGACCAGCTCAGAAGAACACTCCGTGAAAAGAGGAGAATTCGGATATTCTTCAGATGACGAAAGCACAGGATCAGCAAACCCACACTGCTTTGTCTGATGAGTTGAGATCCCTGGAGCAGAAGCTAGCCGAGACAGAAGAGATCCGCTGCGAGGACTTCAGCCACCATCGAGGAGTCCATCAGGGCTGAACTTAGGCAAGCTTATGATAGAGAA

>UN00059

TTACATGCATTGAAGCATACAATTTTTGGTAAGCCCTATATAACCTCTTTTGTTGTCCAGAGCTATTTATGGGCGGGGAGAATTCTGATGATACATACTGATCAAGGTATATACTTCGGTATATAAAATGCTATAGACCAGTGGGTCCACCAATGCACAAAC

>UN00060

TTTTTTTTTACTAAAAATAAGAACTTACTAGAACGTTAATCACATTATTTCACTTGTTTTGAGTACATGTATATATCTTTCTAGCGTTCATATCAAAAATCATTATTCTCTCGCTTGACATGTAAAACCAGGCAACGACCATTAAGTTCTACAAATAAAATCTACCAAGTTTGTACAGAAGGAACAATAGCTTGGCCTCCAAAACGGAACGAATAGGATTAACCCGAACTGTGATTTATTATCAAGAAAACACTGTAACCGTGATTAAACACGATGGAGAAGGAACGGTAGCACTAATGCAAGAAAGAGGGGTGAAAAATCGTAGGATACTGTTGCATGGATTTTTGTAGATGAGGCGGCGAGGTACATTAATAA

>UN00061

TACCTTACGAATACGAAGTTTTAGGAAATACCCCGAAATTACCTAAAAGACAAAAAGGAAACAGTGTTGAAGGAGGAGATGGCGAAGAGTTCGTTCAAGCTTGAGCATGACTTTGAGAAGAGACGGGCTGAAGCGGCGAGAATCAGGGATAAGTACCCAGATAGAATTCCGGTGATTGTGGAGAAGGCAGAGAGAAGCGACATACCAAATATTGACAAGAAAAAGTATCTGGTTCCAGCAGATTTGACGGTGGGGCAGTTTGTATATGTAATTCGCAGAGAATC

>UN00062

AGTCATCAAAAAGTAAACCAAATAGGATAACTACCAAAACTTCATTAAAAAACTCCAATCAAGTATAGCGAATAATGTGTAGGAGGCAAGTCATATCTTACAAAGTCTCCTCGTCTTAGCTCTCCACCCAAAAACATTCTCACAAATTCCTCCTTTCTCTCGACCTTTTTCAAAAACATTGTCAAGCCTCTAGAACAGGTAAACCTTCTTGAAAAGCTTCAAATAAAGATGGTTCAACCTCACGAAACTGAGTGGTAGACCGGCTTCTCGAAATACGTGATCTCTTCGGCGACATGGCTAGCCCAAGTTCCAAGCTTGAATAAGGAATTCCAACCCAAAGAGATGAACCC

>UN00063

GTATTCTCAACTAGCTCATTTTTATTGGCTTTATTATACCAATTGTTTATTAGTATATAATCAAACAATGACTTATCAACTCATATATCGTAACTCATATATCGTTTTGTATGACAAATTACAATGAATATTAATGCACAGATGCAGCTTTATCAGAATTTGGGACTGATGATACAACAAGCAAACTCAGCTTTTCCTGCGATCTTCATGGGGCATTGGCATTAACAGGAATCAGCACAGCACGATATGAGACCCAAAGAATAAAGTAAACAATAAGAAATTTAAGAAAGAAA

>UN00064

GGTCTCCACAAGTGAGAAGCATTTTACTTCCTTTCTTGTAAACCAGCAGTGTAACATATTCATACATCCTAAAAGCTTATTGCCCTATACAACCAAAAACCATTAGTAAAATGGTTGAGTTCTTCCCTCTCTACTCGTAGAAGACCTCCTGGTTAATACATTGGGCTCCTGTTCATTTACCCATCTACCTCTAAAATCTCCAGTTAGTTTCCTACACTCACTACTTGAATCATCAGAATACAAAACTGAGGGCTCTCCCTACCCTCTACCTCTCTCCTTTCCCAACCCAACCTCTCTCCATAACTTAATCCATCACTTTCCCCGGCGAAATTATGCCTCGCTACCAAAGGGGACGACATGAAACCATCAGAATCCTCAGAGAACACTGAAGAATTCATACTGAACCGACCCGAGCCTCCAAAAATGTACTCAGAGAGCTCCTCTGTAGTGCTGACTAGGTAGGAACTACTACGA

>UN00065

ACTTAAGGTTTAGTAAGTCTTATACCAAGTATCGTAGAGGGATATTGGATGGTCAAATGTGCTGTAGGCTCCAAAGCCTGCCTCTTAGGAAAAGCAGTTACATGCAGTTATCTCCGTCAAGACAACTACCTAGAGATTGATGTGAATGTTGGATCTTCATCTGTTGCAAGAAACGTCGTTGGTTTAGTTCTTGGGAAAATCACAGGCCTTGTTGTTGACCTTGCGATCCTGATTGAGGCAAGGGAAGAGAATGAACTGCCTGAATACATTCCG

>UN00066

CGATCAAAATCGATGGAAGCAACAAAATTAAACGAATTGAAGCTGTTCATAGAGCAGTGCCGCAACAGTCCCGCTCTCCTCTCCGATCCTTCCCTCGCTTTCTTCAGAGATTATCTCGAGAGTTTGGGGGCTAAATTGCCCCCCTCTGCTTATGGCAAGGCTCAATCACCGAGAAGTCGCGAAGGGAAGAGCGAAATGATTGATGAAAGTGATGAAGATGTGGATGAAGATGATTTTGAAGGAAAGGCTGCTCCGCCTGAAGAGGAACCGGATGATGAAATTATTGAGTCTGACATTGAGCTTGAAGGGGAAACAGTTGAACCAGACAATGAGCCTCCCCAAAAGATGGGAGATCCAACTGTCGAGGTTACAGAAGAGAACCGTGATGCTGCTCAAGTGGCGAAGGGAAAGGCCATCGAAGCAATTTCAGAAGGAAACTTTGATGGAGCAATTGAACATCTGACTGAGGCTATTTTACTTGAATCCAACATCAGCAATTATGTATGGCACTTAAGAGCTACTGTATATTATCAAGATGAAGAAACCAAATGCTGCTAATTCGTGATGCAACTGGCGGCATTGGAGATCAACCCCGAATTCTGCCAAAGGCTACCAAGGGCTCCGTGGTATAGCTTTATCCATGCTTGGTCAAATGGGAGGAAACTTGGCCAAGGATCTGCACATGGCGTTCCAAGTTGGATTACGACGAAGAAGAATTGGATCTTGTGCTTCAAAAAAGGTTTGAACCCCA

>UN00067

TTTCTTAAAGATCATGACAATTTTATAAAATATAAATAAAAACAAACGACATTAATTCTTGGCAGCCGTAAGTAAAATCCACAGTAGTTTCTCCAGTAAAAATATTTTATGATAATCTTGTACACTCCCCACTCAATAGTTCAACATAATTTTGTGAAATTTAACACCTTTTCTTCCCTTCCTCTATGCAGGTCAGTAACGGCTCCGAACACGAGCCACATCACTGATATTGGCTCTGCGCGTCCAAAACGCCGCAAGCCAAATCCTGGCAATGAAGAGCAAATAAACAACAAACAATTCCCACAATATTCATCTCGATTACGCCCGTCCAAAGCG

>UN00068

TGACCGTCGGGCGACCCCGCCTCCCGCATCGGAAGTTGCGGGTCGGGGAGGGCGCGCGGAAGCTCGTCCGGCCAAAATGTCGTGTTTCCCGGCGTTCGGGGCGACGGCGAGGGGTCCGGGCCCGCGTTTCCCTGCGTTCGGCGCCGGGAGCGGGCTAGGAGGGCGGGAGAGGCGCGCGGGGTGACGTATAAGGGGGGAAGGAGAAGGGGAGTGGACGGG

>UN00069

ACAGTCCACAGAGATATCCAACTCATCAAATATAGTCTTAACCTTTGCTAGGAAACCATACTGGCCAAGCATCCGAGTGCTCACTATATCCGACATGGTGATTATTTGATTTCAAGACTATACTGGTCAATATAGCCCCAGTCATATCTCTTGCTTTAGTGATGAGAGTACCAGGAGCTGAGGGATTGTACGAATTCTTGACCCTAACCGGAATATCAGTTTCCCTAGCAGGCCGCATTGACTGGGGATGCAAAACCTGTGCACCAAAGTAGGCAAGTTCAGCTGCCTCATCGAATGTCAGGTATGGTACAGGAGTTGCATTCGGACAAATATTAGGATCACATGTCAAGACACCATAAACATCCTTCCACACCTGTATCTCTCGTAACCCCAAGGCTTTACCAATTGTAGTTGCTGTTAAGTCACTACCACCCCTTCCTAAGGTGGTCACAGAAGAGGATTTCCAACCTTTTCCCAGAAAACCAGTCACAATTGGAATTGCTGGATCATCAATCCAATCTCCATGCAATCTCTTGGCTACAGCAGGATAAGTTGCTTCAAGGATCTCCGCATTTGTAAAGTCGTCTGTGGTTATAAAGCCAATATCAAATGCATCATACTGTCGTGCTTTAGCACCAATTTTGTTTAAATATGCTGCAAAAATTCTAGTAGACATGCGTTCCCCAAATGAAACCAGATGGTCTCTTGTACGAAGTGTCAGCTCTT

>UN00070

TCTCGAAAGATCGCCTTGGGTGGCCGATACTCCCGGGACTTCTTCAACAGATATGGACGAATTTAGAAGAACAACATTCGCCGCCTCAGATTCTGGCCTCTTCACAAAGTTCTTATGGAAAAATACGACTTCAGCGAGCAAGATGCCACAGACATGGCCGATTTTCTTGTCCCAGTACTTGACTTTGTCCCCGAGAAGCGACCATCTGCTGCACAATTACTTATTCATCCTTGGCTGGATGCAGGGCCAAAGGCTTTGTCAGCCCACAGCAGAGCCTTCAGATGATAAGCAGAAAAAGGATAAAGGATGAAAGAGAGGCGATGGCAACAGAACTTGGTAATATTGCCATTGGTGGTCAATCTCAT

>UN00071

AAAGTTAAATGTCAAGATATTCCTAATATAAAGTATAATCTATTTCTAGTCACGAAAATTATTATAATCAATTTCATATAGAAAATCTTAACCAACAAAATCTATTACAATCAATTTTATATAGAAGATCTTAACCAACAAAGTCTATTACAATCAATTTCATATAGAGTATCTTAACCAAAGAGAGGAAAAATATGTCGAAGTCCTTGGTTTTTTGACTTTAGTGAGCCCATCAAAACATCAATCTCGCATCCTTCATCCCCTGAAATTAATTCAAAAAAATTCAAATAAGTAGAATATAAACATCAAAAACCTATAAAATGAAGTCTTAAGATTCTTTTAGAAGCAAAATGAAACTACCACGCGTCGTATCATGGTTTCTTTTTCGTTAAGTACTTAATTC

>UN00072

AGATTCAGATGGATGGTGAGCTAGCTGTAAACTTGCTGAGGGTTTTGCCATTGCAAATTTTACCAGTTTTATTGGCATACAGTATTATCGAAGGAGCACAAATAGTAAATTATTCTTGACAATTTTATCTGGTAAAAAGCCCAGATGTGATGCTAAGCCTTAGCTCAGTGTAATATGGTTCCAGTTTGTTTCTTATTTTTTTTTTAAAGAGAGAATGAAGAGGTTTCT

>UN00073

ATTTGCTGAGCATCTACCAACATATAGGGATTATTGCTGAGCATCTACCAACGTGTATTGTACAAATAACAGCAAATCTTCGTTATCGACATACAAGCATCCCTAATAATCGAGGATAAAGATACTTATTGATTCTTCTGGAATTGGATCACTGCTGCAGCCACCACAAAAGGACTCCTGGAAGTGTATGAAAGATCAAAACTCCATGTAGTCTTTTCTCTTCTCTGTTATCTGTAGGTGAGCGATTCAAAAGTCGGAACAGCGTCCATTGCGACTACCCAAATACCCCGTAACCGACGTAGGGACTACGGGACCCAAC

>UN00074

CGTGTCACCGGGGATGTCCTTCTCCGGGCGCGGCGGCGGGAGCGGCGGCGGGTTCTGCGGCGGCGGGTGAGGAGAAGGAGGAGAAGAAGGAGAAGACGGCGTTCGATGTGAAGCTGGAGGGGTTCGACGCCGCGGCCAAGATCAAGGTGATTAAAGAGGTGAGGGGGTTCACTAGAGCTAGGGCTCAAGGAGGCTAAGGAGCTGGTGGAGAAGGCGCCGGTGGTGGTGAAGAGCGGGGTCACGAAGGAGGAGGCGGAGAAGATCGTGGAGTAAGTTGAAGGGATCGGCGCCAAAGTCGCCATGGAGTGAGGAGCAGCGTCTTGGCGAGGTACAGTTCTTGTTTGGATCTGTATAACCTAAATGACTGATTGTTTTAAGATCAAGAAGATGACGAATGCT

>UN00075

TCCGTATATCAGGTACTACACTATTAGCATCTAGAAAAGACTGAGGAAAGCACTAACAGTAGGCCCAACTACATGAGCTTGACTGAATCCACCAAGGCAAAGCAGAAGATTTGCAAGGATTCTGAATACAGCCCTTCATTCTCTTCTCAATTAGCAAGTGCAGCATCATGGAAAGATAAGAGCTTGATGAGAAATCATGGAAGATGAGAATATTGTTGCTGTAATGTGAGGCCGTAGTGTTATCTTTTGCCACTTGTGGAAGTTTTAATTTGATCTTCAATCT

>UN00076

CTGATTTGGCAGATTCCTCGCCATTGCCTGCTTTCTTGGCTTTCTTCACTTCGTAATCCTTGACAGCAGCCTTGATAGCATCCTCTGCAAGCATGCTGCAGTGGAGCTTCACTGGTGGAAGGGAAAGATGCTTTGCTATTTCAGTGTTTTTGATGGTCACAACTTCCTCCATCTGTTTTCCCTTAACCCATTCAGTAGCAACAGACGAAGATGCAATCGCCGACCCGCAACCAAAGGTCTTGAAACAAGCATACAAACGAATACTTACCCCGTACTACAATACGTAACGAAAC

>UN00077

TTTCTACTTGATTTCGGTACCATGATCAGAACTATCATGACTTTTTCCAGTTTCATTATCAGCATGTGCATGTTCATCTAAAGCAGGAGACCCTCGTAAACTTTTCGTTTTAGTATCAGCAATTCTATTCCTTAGATCTTCTGGCTGCAATACTTCTTCTCTCACTGGCTGAGCAGGTTCTTCTGCAATTTTCTCCTCAGTCTCGGATGCATTCTTAGCATCAGCGTCATAAAAGTACTTTTCCAAACATTGCTTTATAAACTTCTTATGAGCATCCAAAGATAATTTCTCCATCCCTAGGTCTTTTTCTAATGCCCTTCGAACACCTTCAAGAGTCAAGTTATCGGCTTGCTGCTTGAAATCGGGGAGGCGAGAGAGCATCGCCTTCGTTATCTCAGCCTCCACCTCCTCCTTCGTCTTCGTCGCCGCCGCTGCTGATTCTTCTTCCTCCATCTCTCTCGCTCGTGAGAAGGGGAAGGACTAAACTAGAAGA

>UN00078

CTTATATAGTAGGGAGTCATCTGCAAATGGGGCCCCCTATGCTAAATGTTAACCTTCCAGCTGGTGAACCTCGGTTGTTGGCAGAAGCTAAGTACCAAATAAGAATAGTGAAGATTGCAGAGGTGATATTGGTTAGAAGGAGCTTGGCTTGGATAACTGGCATCATTTTCCTGGTTTCTACGCTAATGGGGATATACTACTTACTTTACATGCCTCTAACAAGGGACACCCTCCTTTATTCCAACGTAAAACTCGACTAGAATTAAGACTAGAAAGAAACGACAATTAATCGTCAAGAATCTGCCTCGGCATCCGCTTTCTCAGAATTAGATAAGAGCGCTGCTCTCTTGTTGCATTCTTATTTTCTTATTTGCTACCGGATTATTCCAGTCTGTATTCAGTACAAATTATTTTGTGTAGTTAGTTCTTGTAGAGTAAAAATAAGTCCTAAGTAATTGTTCCTGTACTTTGATTTGTTTTGTAAACATTCTGTGGGACAGAATGTAAGAGGCTATGATATGTATTATAATTTCCCCCACTTCGC

>UN00079

AAAACTATAACCACGTAACAGAACTATATTAAGAGCACTAAGCTCGTCTACATAGCATAGCAAGCACCGGAAGCACTCATTATTTTCACATCATGACTCCACATATCACAAGTAGCATAGTCAAAATAACAGCTTATTACACAATTATTGATAAACAGAGTTTTTAAAGCTCCTCTTGCATTTGATACAACTGGAAGTTCCCTTCCAGGTTCTTGTGCACTTCTACTTTATACTTCTCCTCCTTGCTTCCTCTCCTCAGTTTAAGAAGCAAGTCGAACTTAGCTAGGTCTTCAATCATCTTGCTTGGCACCAAGGTCAGCAGCAGTGGCACCGGAAGAGGAGTCGGACAAGGGAAGTAGAACTACCTAAGGAAGAACTAACCTTAAGAAAAGTT

>UN00080

CTTTAGAGAACGGCAGGACACTGAGCCATTTAATTTAGACAAATATCAGTTTGAGAGTGGGATGGAGCTGCCAATAAACATCATAATATATAACCCACCATAATACAACATTTATAAAACTGAAGGGAAGAAAAAATGAACAGGGGGAGAAAAAGCCAATTCATGTTCAAAAGTTGTCTGATACGAAAATACTTCCAAGAGCACATTGCCCTTGCTCCCACTCCTCCGCTATTAACTAGAGAGGGCTCCTGTCTTCAGCGCCTGAAATTATCTACATTCTCGTGAGCTCCTGCTAACTGCTTTTTTCTCAAA

>UN00081

TTAGTTAATTTTAGGTCCATATGGGTCTCAGTGACCAGGACATCGTTGACTACTTTACTAGGGGTACAACACTCTGGGGAGGTGCCACAAGGAGCGATCAGGATTTGAGGGGGCATGGACTTCAAACCCTCTTATCTTCGACAATTCATACTTCAAGGAACTTCTGAGTGGAGAGAAGGAGGGTCTTCTTCAGCTTCCATCCGATAAGGCTCTCTTGAGTGATCCAGTCTTCCGGCCTCTTGTCGAGAAATACGCTGCTGATGAAGATGCTTTCTTTAGTGACTATGCTGAAGCCCACCTAAAGCTTTCCGAGCTTGGATTTGCTGAGGCTTGAAAGGTTATCTATGGATGATTGTGAGAAATGTATGATGTCAAGTTAAAATAAGAGCATCCTGGCTATATAGGTTGTAGGCCAACTTAGTTTATTTTGTGATTTGGTGGTTGTCCTATTTCTGAAACTTTATGGGCTATACTAAGTTTAAGTTTTAAAGTAAAAGTT

>UN00082

GTATCTCAACAGCTCATTTTTATTGGCTTTATTATACCAATTGTTTATTAGTATATAATCAAACAATGACTTATCAACTCATATATCGTAACTCATATATCGTTTTGTATGACAAATTACAATGAATATTAATGCACAGATGCAGCTTTATCAGAATTTTGGACTGATGATACAACAAGCAAACTCAGCTTTTCCTGCGATCTTCATGGGGCATTGGCATTAACAGGAATCAGCACAGCACGATATGAGCCCAAGATAAGTAACATGATTTGAGTTGATACGTTCACTTATCTCCATCAAAGCTCCACCTTCTCAAATTCGTGGTGCCCGTTCGTATCTTTGTCGCCTGGAGCTGGTAGTTCCTGGCCCTGACCATTAGAGGTCTTCACTTCAACGTTCTCCTCGATATTGCTCGGTTTTCCCTTATTCTTATCTCGCCACCCGAGTCCAAATGGCATAGAAAGCAAGCCTACTTTTCTGGCAGGGAAATCTTGAGATGGATCCTGATTGTATCTTGCCGGTTGATTATCCGC

>UN00083

GTTAAGTTTATTTTATTGTAATTAGTCTCTTTGTTACATTTAGAGCTATTTGGTATGTGGGAATCGGATCAAAGTGAGAATAGAATGAAGTAAGGAATGAAATGGAATGAGTGTGAGAATGGAATGCTCATTTCCATTCTCACGTTTGGGTGCGATTAGCGAATGAAGTGGGAATGCAAGTC

>UN00084

TTGATCATCACAGGATAACCAATTTCATCCGCAAGCCTGATAGCCTCTTCCGTGCTCTGCAATAGGCCATCACTTCCAGGTACAGTTGGAACACCTGCTTTCTTCATTGTTTCCCTGGCAGTAGATTTATCACCCATGACTCTGATGCTGTCAGGATTAGGTCCAATAAAGTTGATTCCATGTTCTCTGCATATGTCAACAAAACCGGCATTTTCAGACAAAGGAAACCATACCCAGGGTGCAACATGGTGCATCCACGACTAACGGCAG

>UN00085

CATATCTTTTTTCACAAGAAATAAGGGGAAGATCATCAAACAATACATGAACAACGAATCGTATATATCCTAAGTGAGATGCATTTCGCAAAGGAGGAGCAAACTCTTGGCGTCATAATGTAATTATGCAAAAGCTGGAGATGTGTTCTTTTCATTGTGAGGAATTGTCATTTGAATGAAAAGCAAAAGGGCTTGTTTGATGGAGACCCTTAGCAATCTGTAGCCACCTATTTTACTCAAATGGAAGGTTTTGATGCAGAAGTTTCTGGTTGGTGTTATCTTGATGGTCCCTTGCTATGCCATTGAATCGGAGCAGAAGTTTCTGGTTGGTGTTATCTTGATGGTCCCTTGCTATGCCATTGAATCGGTACAGAAATGATGAAAGCCAAGATTGAGATTGATACTGACAGGTTCCTAGTGAGTATTTGACATTGAGGAACAAGTTTTTGGAAACAAATAGAGAATATAGTCTTTGACCGAGAAGTTTCATGTTTTGTAATCAAATGTTGATGTACATTGTTTGAATGTTAGTTCGGTACGATTATTTTTTGAGTTAGAAATGTTTGTCGTGTGGATTTAATACTTTGTATTTTTTTATACTATACTGATGTATGTTGGCACTTATCATTATGGTTGATTAT

>UN00086

AAGTAAAAGTAATTCGTTCCTATCCAATTCAATCTATATCTCACAACTGTTTCAAAGTAGACAACAAGCAGAGCCCATGCTCTCTGGGGGCCGCTAAAGCTTGAAACACGAAGGGGCATATGCTTCGGTTTCCCCTACAGGGATCAACCCCCTTCCTCAAAAAGGTTGCTTATTTGATTTCTCTTTTGCACTTACAATGACCAAAATCTACACACGAGCAATGAACCAGCACTCTTAAGGCTTGTCCAGGTTATCACTGCTATCGGCTATTCGTCCAGAACCGCGGTTACTCTATTCTTTACCGTAAAGTAGTCCCTATTGGTCTTGTACGTACTAAGTTCTTTTGTAAGTACTTAAGT

>UN00087

ACGTAGATCAACAAGGCATCACTTCTATATTTCTGTTTTGATAGAAATCAAAATGCCAAAAGTCGCTCTAACATCAAAAAAGAGGAGAAGCAAGCAAAGCAGTCCTCTTCAGTTTTCACTGAAGGCCACTTTCAACATACTTTGCTTCTTCCTCTTTCAGTTTCCAATCAAACCAGTAACGCCCCCTACAACCCACACACCAAAGAATCGTGACTTAAATAAATCTACAACAACTTGTACAATACCAATTCACGCTACATTGGTTTCAC

>UN00088

CCTGTTGCTGTCCGCAGAACTCTTGTATTTTGCTTTTGGGCTTGTGTCTTGGGCTTTGGCTTGGACTTTTGGGCTTGGCATTTTCTCTTGATTTTTCATTGGGCTTTGAATTATTTGAAAGAATTCCTTTGGCTTCATGGGCTTCATTTCTTCATTAAATCAAGGGGTTTTTGTTGAGATATTGGAGTTTGAGATTTATTCATCATTAACCTGCAATTAACAGATAAAAACAATTATTAGTGGTATATTTATTCATATAAAATAGATAATGAGGGTATAAAATTGTAATAAAAATATGATTATCAGAAGTACAATTACGAAGAATAAATAGCGATAATCTTCTGCATATGTAGGAAGTGAACTTTTTAGGTCGAGCCAGTTAATTAAGGATGAGTTAGATGAAGTGGCTTTGACTGAGAAGAGAAAGAGGTTTACTGAATACGTAATGAATATGAATTTACCTA

>UN00089

TTCAAAATTGAAAATTTTTTAGATGATGTGACACGTGAAGTAAAAAGATTAAAAAACCTCATTTTCTCTTTATTCTTTAATTATATAATTATTATATTTATTCTTTAATAATATAATTCATTCTTTAATAATATAATATAGTAATGATAATAATAAGACAAAAATAATATAATAAATGAAAAAATAAAAAAAAAAATAGAAATAAATAAAAAAGAAAAATAGATAAGATATCTACGAATTTGATCAGTAATTCAATTTAGATAATGATTTAATGATTCGAAACGGAGATCTTATCTCCAATAGAATAGATAGATATA

>UN00090

ATGTCAAGAAGAAGGTGAGGAGTTTTTGGGTGTTGGATTTTTGTTATTTCCTATTTGTTTGTTTCTTGAGGGGTGAAAGATTCGATTTTGGATCTGGATGAATTATTGGAGTGATTTTTTTGAAGGAAAGAGGGGAAAGATGTGATTTTGGATGAGGACAAGGCATCATAAATCTAGTTATTGTTACTATTATTATTATTAGATTTTGTTTTGATTGTCAGATGAAGTTGGAGTTTTGGATGGAATGTTTATTTATTAATGGGCTTAATTTTTTTTTTGTATTTAAGCAATGTGAATGATGAACTGAATGAATACCAACTTCTCTTTACTAAAGTTC

>UN00091

GAATCATTCATAGCGATAGATAATGAAGCAGAAAGAATATACATATCGGGTTACCAAGATGGATTGTGTGAGGATATGGAACGCTACATGTCCTATCCTCAAACCTCATGTTTCTGTTAGAAGGGAAGGTACCAGGTACTGAGGTGGATGGCCAAAGTGGTTCAGTATCTGCACTGGAGTTTTGTCCCACAAGTATGAATTTGGCTGTTGGCAACGAGTGTGGTCTGGTTAGAGCAGTTTTTTGTATTCCTTTTCTTTTGAGTTATTTACACTTAAATACCCCAAAGTTTACACCATCTTCAACTTTGATACCCCAAAGTTTTTCTTTGCCCAAAAAGCACCCCAATGTCAAAAAAGTTTATACCTTTGATACCCCTGATAAGTCATTTTGGGGGTACCAAAGTTGAATGTCGTTGAACTTTAGGGCCTTTTTGGGCAAAAGAAACTTTGGGTATCAAAGTTAAAAATAACATAAACTTCGGTG

>UN00092

CAACTAAAATACTCACCGTTTTCCTATCAAAACCCTAAACCCCTAGCTCAAGAACTCCGATCAATGGAATCCGAGGATCTCAAGAACTCCCCTCACAACTCCAACGGCTTTCCTTCAGAGAAGCCCACCGAAACCCTAACCCCAGAGAAAGATTCCGACGAGGATCTCGAGACTCCCGACGGATCCGACGCCTCCGGCGACGATTCCGCCGTCAAGATCCTTCACCAAGAGATCCACGTCCTCCGCCGCGAGAAATCGGAGCTCCAGCGGCGGATCGATGAGCTGACTGCTCAGTTGGAGCAATCGGAGACTGGATCCAAGGCCGTCGCCGATCATGCTTCGTATCTTGAAGGAGAGCTCATCAGGGTTCAGGAGGATCTCGCTATGGCGTCGTCGGTGGTTGATGATG

>UN00093

GTTCCGTCCGTATTTCCTTCCTTCCGGCGTTTCCTCTTCCTTTTCCTTGATCACCCCTTCCTCCGCCGTCTTCCAGGTGGTCATCGGTGGAGGCGGTGGCGGAGCATTTGTAGGGGGCGGTGGTGCCGCTGGCGGAGCCGCTGCTGGTGGTGCTGCCGCTGCTCCGGAGGCAACTCCGGCTGAGGAGAAGAAGGAAGAGAAGGAAGAAAGCGACGATGACATGGGATTCTCTCTTTTCGATTAGGTGTCTTGTATTTTCTTTTGGATGGGTTGAGACGTTTTTGTTACTATTCCTAGATCTTGTTATTTTCGTTCTATTGTAACTTGGTTATTTCCTTGGGGTTACCTTTATTTTATTTGTTTATTTTATTTTTAG

>UN00094

ATAATAAAATACCAAATTAGCTCTACCCATCTAATCGCAACTATCTAAGTCATGGGAAAAAATTATCAAAAAAAAGTCATGAAAAACATAAAATTATGAAATTAAACAAAGATAAACACAAAGAAGACCACAATTTTGACAAAACTACCCTTCCACAATTAAACTCTTTTAATTAATTTTCAATTTTCTCCAACAATTTTGTACACCAAACTACCTCACACCTTTCACCACCGATACTGAGCCCGCCCGTTCCTCGGCGAACTGACACGTGTCCTTTTTGCCGAATCGGACGGCCCCGAGAACCC

>UN00095

TTTAATTAGGTACCTAACCTGATGTTTTGAAGTCTACTTTATTCTGCATTACAGCCGTCAAGTTCGATGGAAGATCTTAGATATCATGATGGTGGCCGTCTTTCAGATCAACAGATGGCACTTCTGCAATATCAACGGGAAAACCTTCACTATTTGAGTGAGGAGGTTCTACGGTTACAAGAATGTTTGAGCAAATACCAGCGATCTGATGATGGCAGCACCCCTCAGGTTGATCTTGCACA

>UN00096

GTTTTCGAAGTTCTGATGGAGGATTTGCAGAGCGCTCTCTCGTCTCGTGGCCTGGCGGTCTCCTCGATCCCCGGCAAAGGCCGCGGTCTCGTCACCACCAAAGACTTTACCCCCGGGGATGTTATTATCTGTCAAGAGCCCTATGCATCTTGTCCAAACAGGTCTTCTGGCGGTTCAATCTGTGATGGATGTTTTGTTTCAAATAACATAAGGAAATGCTCAGCGTGTCGGGTTGCCTGGTATTGCGGGAATAAGTGCCAGAAATCAGAATGGAAATTGCATCAACTAGAATGCCAAGCTCTTGCAGCGCTGACTGAGGACAGGAAAAAAATGCTTACTCCTACTATACGACTAA

>UN00097

GTTCTAGTATTAATTTCGGTTCATTCTATTAAAAAAATCCCAATAGAATACCAAAAGCATTCATTTCACAAAGAGGAGCACAAATAGCCAACTGAATCCGAAAGAATGCTCTAAACTAACATTTGAAGATTTTCACACGAGATACATCAATAAAGAACGCTTTGCTTCTAAAACTGGGTAGCATAAGAAAAGCTAATTACAAGATGAGTGCATAACGAAATTGAAGGTTTCAGGCAGAAATGTCATTTGAATGGAGCTCAGAATGCATCTCCCGGACAAGTATTTCTTCGATTGCATCCATGAATCGAACAAGATGGCCTCGAGTTTGAATGTTCAAAGAATTTGTAAGTTCCTTAACCTTGTAAAGAAAACTCAGGGAAATGCTCTTGGATTGGCTATTGGAGTTTTTTATAGAGACCAACTTCAAACCAGAATCTGATATCGGCCAGCTCTGAGTTACTTTTATGAAAGCATCAATACCCCCAACCAGATGAGCTGTGATAGTCTCATCTCTGTCAGAGTACTCAAAAGAGTGTCTTGATATTTTTGCATCTTTCAGTAACAAACGTCTTAGAGTGCATATGAGAATTCGATGTTGAACATGCCTCACCAAATATTCGAGTGCAGAACAATTCCTTGCAATTGGCATGTTACTTGAGGATTTAATTGTGTAAACAATATCATTGAGGGGTACATCATTAGGGAAAATCTCAACTTTATGTACTTCCAAAGTCTTTTCAACAACTTCTATTAACAACTCATGTTCCACTGTAGATGGCTCTACTTGATAATCCAACTTATGTCTGAGAAGTAAACTTTCTGAGGAAGGGATGGGCGTTTTCAGTGACAGCCTAATGCAGTTATCTTCAAATTCAAGAACCTTGACTTGTGATAGTTTATCTTCAATTTGCCCAATCGCTTCCATCCTTTTGAAAACAAAATCAAGATCTTGCAGAATACTTAAATTATTTTGGTGTCGATCAATATCCTGTTGAAGCTTCAATATCTCATACTGCC

>UN00098

GTTACTTAGTTAAGTACGTAAAGTTATTTAAAAATTAGTAAAGTTGTTTTTCTACTTTCTTCTGCATGCTCTGCAGTTTCACGATTCAAATGTAGCACACTTTTGCCACAGAAATCGAGACTAAGGAGCACAGCCACTTGAAAAAGAGCCTGTACAAATAAATTTCTCCACATGATATTGGTTAACGTAGAGGCTCCCTCCGTCCAACTGGGGGTCTTTTCATAAGCTTGTCAGTTGGTGGCTCGGTTGCCAACGCAAGAGCTCCAAGGGAATCCATGATGAGGTTAATCCACAGAAGCTGGACAGCATTTAAAGGCACATCACCAGATGAAATGGCAGCTACAACATTTATCACAAGTGCTGCAACGTTCACTGTAAGCTGAAACTGGATAAATTTTTGAATATTTGCATACACAGAACGGCCCC

>UN00099

GTTCTAGTTAATTACTAAGGGGGGTTTTTCTTTGTTTGGTCATAAAACATATGCCTCCACAAGAACATGTTTGGCTGGAAAAGAGTTACGTATAAAGGGAAACACAATCTGATGAACAATAATGGTGAAAGAAGTTTATGAATCTGTTCATTACTTTCTCCACCTATTTTTACCATCCAATTACTGTAAAGATACATAAGAATTTTTACACCAAGCAGATGCTGGTATAATGATTTATAATGCTATCATATCCTGATTTTTTTTAACTCTTTATTGTGAGTAA

>UN00100

TTCGTCCCCTACTCCTCTCCTCCCTCTCCCACGACGGCTTCACCCCTCCCTCCATCGAGGAGGTCACCGGCCATCTCCGGCGTCGACCAAAACCGCCTCGTCGTCGCCTGCCGCGTCCGGGACTCCCTCGCCGTCTCCGCCCCCGACCTCCTCCCCGCCTTCGATTCCTCCTCCGCCGCCGACCTGCTCTACGAGCTCCGGCTCCTCAACTCTGCCCAGCGGGCCTCCGCCGCTCGGTACGCCGTCGAACGCGGTTTTGACGGCAAAGCCGCGCAGGAGCTGGCGAGGGCCATCAAGGACTTCCCGCGCCGCCGAGGGGAGCGCGGGTGGG

>UN00101

TAGCATATTATGTAAGGGGTCCAGCACCGCAAGCATTCCTTCAATATTGTGTTCACCAAAATACAAACGGCTTGCTTCCTCCAAAGCCTCATGCCACATTTCATGCCATAATATGGCTACCCTGATCAACTCCTTTGACACTAGTTGAGCCTGCTCAACAAGAGCTCCACTGTGCTGGCGAATTTTATCAACCAACTTCCTGTGCTGCAGCTCTGCGCAATTGGCTTATTGATTTGCATGCCACCAGAAGAGGATACATAAGAGCCTGTGGATGTCCTCTTCCTATCCGCACCAACAACGATTGAATCAGCTCTCGTACAGCTTTATTATTTGAATGGATCCTTGCAATTATTTGAGGTAATACAACCAACCACA

>UN00102

AGTTAGTTCTTACTTATTTGTTTGTCTAACTATTCTGGAGAGGGGTTATACGAGGGATTAGACTGGCTCTCAAACAACATCGCTAACAAGTCATAGTATCTCTCTGTTGATGGTGTTTTGCGTGGAGCTGAGAGTTATTACCAAGGATGTTTTTCTTCAAATCTTGCAGTCATAGATTTCTTCAACTATGTTTCCGGCATTTTGTATTTCGCTGAACGACTCATTCGGATATCAGATGTTAGTATTTAAAGATTGGGTACCCGGCTGCTTATTTTTTGTAATCGAGTTGGTTTTTATCGTCTGTCCTAATTTTTGTATAATTTCTGATAGTCAAACATTTTTATTAATGTGTTGATTGTATTCTTGAATAAATAATTGAGGCTTGTGGCTTCTAGTTTTTGGTGTGCTCGCACTGCAAGAGAGCTTCTAGTCAAAATATCTAAGTTAATTTT

>UN00103

GTTTGCAATCAAATGACACAAATTTGAATTTCAATATGATTTTTGTTTACAAGATAAAACTAAAAAATAAATTAATACCCTGATGAACACATCCAATGGAACAGTGCCAGATAGATTCATATACAAAGAACTACAGATAGGATTACAATTACAACGTTGAGGAAACACCTTCAAGCTTTTCATCCAAACTTCACACATTGGCTCTTCTCCGAAGATACGGAATCACTTTCCAAATCGATGGCTTTACGCTTTTGACCGCTG

>UN00104

TTTTTTTAAAACTTTTAAATTTTTTAATTTCTTTCTTTGGTTTGTATTTAAAATTTCTTCTTCCAAAACATACGCCAAAATTTAACATAGATAACATAAAAAGATATCCTAAGGAAAAGAAACACCATCTTTGACAACAACACCGCATTTCTAGCCCGCTAATACAAAAACTAATTCTTGATCAAATTCACTGATCACCAGGGAGAGTCTTGATGATGTCAGAATCACCTGGATCAATAATGCTGAGGCAGCATACTCGGTAATATTTACCGCATGCTGTTCCCAGAATACAACATTATTTCCTATTTAAAATTGGTTGTAACCCCTAAC

>UN00105

ACCCTGTTGCTGGCCAAGTTGCTGCCCTAGTTCTATGCCATACGAGAGAATTGGCTTACCAGATATGTCATGAGTTTGAGCGGTTCAGCACATACATGCCTGAGATTAAAGTTGCAGTGTTCTATGGAGGTGTTCATATTTCAAAACACAAGGATATTCTAAAAAAACGAGTGCCCTCATATTGTTGTCGGAACTCCAGGAAGGATTCTGGCATTAGCCAGAGATAAGGAGCTCTCACTAAAGAATGTGAGGCATTTTATTCTTGATGAATGTGACAAGATGCTTGAATCGCTTGACATGCGGAAAGATGTGCAGGAGATATTCAAAATGACTCCTCATGATAAGCAAGTCATGATGTTTTCAGCTACTCTCAGCAAGGAA

>UN00106

TAGTCTAAACTATTTCCTTTCTTTTACGGGAAATCCCTGGAGAGCGTCATGCATGGTACTCTTAAGGATAACCCGATCATGCAGCTGAATATAATGATAAACCTAGTTTCGTAGAGAAAGCTGGGGGTGGATGGTTCATGTAACGGGAAGTTCTCGGAAGAATGCTATATGTAATTTTCTTAGGGATAACCCGATCATTATAAACAAAGTGATAAACACTACATCCCCACGGTGATTTCTCGAATACTCTTTTGCTGAGATGAGATTCGATCACTTCGTTGGATCAATCTTCCCTCATAAAATTCTCATTTGGTCAAACCCTTAAACAGTTAGGACCAAAAGGCAAAAATCTCCTCTTAATTCCTTTTATGAACGAGAGGT

>UN00107

ATCAACAATGCCATAAGCTTGGGCTTCTGTTGCTGACATAAAAACATCCCTTTCCATGTCTTCGGATATAACCCATAAAGGGTTGCCCGTTCTTTGTACATAAACCTTTGTGAGGGTTTCGCGAAGTTTCAGTAGTTCTTCTGCTTCCAGGATAAATTCCCCTGCTTGCGCCTCATAAAAAGAACTAGCAGGTTGGTGAATCATAACCCTGATAATATTGATATAACATCATGCATAGAACGGTTCTTCTATTTCGCAGGATTGGGCTAAAGTAAGGGATAAAAAAAACAGAAGAAAAAAAAGAAATAGACTTGAACAGCCGTACAGGCATCCTTTGTGCATTGCATACGGCTCTGCAATGGAA

>UN00108

GGTTTTTTCCGGTTTTTCCTTTTTTCTTTTTTCTTTTAGTTTCTTTGTTCCGTTCCGTACAAGGCTCTTCTCTCTGATCCTGGCTTCCGCCCACTTGTTGAGAAATATGCTGCAGACGAGGATGCTTTCTTTGCTGACTATGCCGAGGCTCATATGAAGCTCTCTGAGCTAGGGTTTGCTGAAGCTTAAGGTAATGAATCCGACATCAGGTGAAGGGTCTTCATGCTTGTTGCTTCTCTCGGCAAATTCTAGGATGTCAGTTTGGGGAATGAAGCAGAATTGGTATCAGAACACTATGTGTAATAATTAAGATCTTATTTTTGCCAGCTAGCCTTCATTCTGTTTTGTTGATACTCTGAAGTCTGTTATTGACCAATTAATACCTAAGGTTAACTAGAACTTAAATAAGTTAAACT

>UN00109

AGTCATAAGATCCATGACAAGCGAATCACACGTAAAAAAGCAGCAAAATAGCAGGTGTCTCATTAAACATCAAGCAAATGTTGAAAAATTCAAAAGATATCTTAAACTTCTAAATTTCCTCAGTCTCCACTCAAAATGTTCACCTAGAGCAAATTAAGATACATAGCTAGAAAGTAAAATGAAACGCTCGTGTCCTTGATCACTTCTTGTCTTCCTTCTCAGCTTCGGCAGCTCTCTTGAGCCTTGCTCCGTGGTGACGTTTGTTTGTCCTCTCCAAGCGAAGCTTCTGGTAAGCTCTGAAAGCCTTCTATTCTTCTTTCTAGTTCTAACCTTAGAAACGAA

>UN00110

AATTATTTTGAAGGGCTATCATGATTGCCCATTGCTTGGCAATTGTAAATTTTGTTCTATTTTTTATTGGGAGGGGGGGGGGGGGGGGGGGGATCACAGTTCATCCTGTTTTCATTGTTTGTGTTACTGCTATTTTGCCAATTTGAGTAAA

>UN00111

CTCACGGCCTACGTCCCCGCCCCTCATCTCGAGGAGATATCCACTATCACAATCTGGTTCGGAAGCCGGAACTTCTACACACTCGGTTTACAAGATCACCGAAAACTCTCACACTACACAATCACTAATTAATCACACTTTACACACTAAGTGTAGGAACCTAGCCCGTGTCCTCCTCCTCAAGTGCTCGGGTGCAACCTCTCCCTAGGTTCGTCAACCGACGCCACTCCTATTGGATTCGACCCCGTCTCTCGCCTCACACTTCTCTCGGTGTACAAGTGTATTACAAGTGATACTCACAAATAAAGATTAAATACAATGGTTTGTTCTCAATAACAAATGAGAACACAAATAAAGCTCACACTCACTAAATTACACACC

>UN00112

GTTAAGGTAGAAGAAACTTACAAGGATCCCCCAACCGCCGGTGCCGTGCTCCGCCTCCGCCTCCGCTCGCCTTTGCTCCGATCCGACTTCCCACCGCCAAATCAGGCAGGCTCCGGCGCTCCGCCAGTCCGCCTCTGCTCCGATCAGCATCCCACCATACCGCGACGCCCCGCCTCCGTCGGACCAGCCACCTCCGCCGGCGCAGCTCCGCCCCGCCAGCCGTGAGCCTCCACCCCACGCCGCATATCTCGCGGGCAGCGGGCTTACCGGCCACCGCCTTCGAATCTGGGTTCAATCTTTCTAACTAAAATCTATTTAATTTTCTTATCATTTATTCATATTAATTTTATGCTTAA

>UN00113

GTCCAGCTTTTGACTCCGCTTCCTCCGCCGAAGGAGGAGAAGAAGGAAGAAGAGAAGGAGGAGGAGAAGCCCAAAGTTGAAGAAAAGAAAGAGGAGCCTGTAGTGATTGCGGTGGTGCTGAAGGTGCACATGCATTGTGAGGCCTGCGCACAAGAGATCAAGAAGAGGATTCTAAAGATGAAAGGTGTTCAGTCTGCCGAACCTGAGCTCAAAGCCTCACAAGTTACAGTGAAAGGCATATTCGACCCCCCAAAACTGGTAGAATACATGTACAAGCGTACAGGGAAGCACGCCATCATTGTCAAGACTGAGCCAGAGAAAAAGCCTGACGAAGAAAAGCCCAAGGATGATGAGAAGCCCAAAGAAGATAAAAAGACTGAAGAAAAGCCCGAAGGTGAGAAAACCGAGAAAGAAGCCGTGGAGAAGAAAGATGCTGTCGCCGGGGATGAGGCAGCAGCATCTGCCCCAGTAGTGACCGGCCCCGGAGGTGCTGTAAAGGTTGAGCAGAAAAGGAATGAATTTTATTATTATTATCCAAGATATCAGCCGATGGAGTATGCATATCCTCCTCAAATGTTTAGTGATGAGAACCCTAATGCGTGTTCTGTAATGTAGAGAAATTTGAGGGGGTTGGTTATAAATATGCTGGAAGGTATTAGGAATAAGTGACTCTTTTTGATGGGGGAAAGAGACGTGGGTGGGATGGGTGAAATTACCCTTTTTTTTATTTTGTTTTCTGGGGTTGTAATTAGAATGAAGAGTCTTTCCCTTGGGTCTTAAGGAGATGAACCGAAGAGGATGTAGAATTTTTTGAGTTTAGGTTTTTATTTTATTTTCTTTTGTAGCTTTTATGCTGTATGTAAAATATAATGTAAACGCTATTATTCTCTATCCTGCTGCTTTGTAGAGGTTTTTTCTATCGTGGTTTTTTGGTTGATTGACT

>UN00114

TGCCCCTTCCTCGCTTGTTCTTAGCTTTCCCGATAACCTGACATTATTCGCAAAAGCATTGAGTAATGTCCAAAATAAAAAGTTCAGCAAGAATGAAATCATCGTATTTTTAAGGGAAAATTTACTCCGATTCCACTGAACTCCTTAATAATCGTTCACTGTAAGCCCTTAAGGTAACACAACAAGTCTATGTGACAATCACGTGACGAAGAAAAAAGAAAAGAAAAAGAAAATACCCCTTGACCCTCGAAAATCCCCTTTATATTCTTTTATTTATTTCTACTGATTTTTTAGTGTTTTCACCTTTTGTCCATCACGTGCTTGGCACAGGACATGTTAGAT

>UN00115

AAGGTTGGTCAGTACTGTAATGCACTGCTTTGATATTATGCTGACACCAAAAGTTTTGCCTGCCTGTCCATGGTGGACTACTTGCTTGTCAAGTGCACTTACAAAACTTCTTTTTCTCCTTAAGGCGTTAAATATCAAATATCAATTGACACTTCAATTTCTAACATTTCTCAGAATTTTCCTTTTCTTTATTAAACATTTTCCTTTCCCGTTATGGCATTATTACAGTTATATAGTTGTTTTATTACTTAAAATTGTGGGATATCATAGTTATGT

>UN00116

TCTCTCTCGTTTCCTAATTTTAAGGCTCTTGTGCTTGATAGTTGTGAAGGGTTCAGCACTGATGGGCTTGCCTCAATTGCGACTCATTGCAGGTACCTTAGGGAGCTGGACTTGCAGGAAAATGAGGTAGAGGATCATGGGCCAAAATGGCTCACTTGCTTTCCTGATTCCTGCACTTCCCTTGTTTCCCTTAACTTTGCCTGCCTAAAAGGGAGTAAATGCTGCATCTCTTGAGAGGCTCATCGCAAGATCCCCCAACCTCAAAAGCTTGAGGCTTAATCGAGCA

>UN00117

TAACTACTTGGTTTTATTGTTGTTGTATGAACTTTTTTCCTCTTATCTTTTAGGTCCTGTTGGGGAAGACATGTTCCATTGGCAAGCGACCATTATGGGACCCACAGATAGCCCCTTTGCTGGTGGCGTTTTTCTGGTGACAATTCACTTTCCACCGGACTATCCCTTCAAGCCCCCAAAGGTCTCTTTCCGCACTAGGGTTTTCCACCCAAACATCAACAGCAACGGCAGCATTTGCCTGGACATTCTCAAAGAGCAATGGAGCCCTGCCCTGACAATCTCCAAAGGTTTTAACTAATACC

>UN00118

GTTTTAAAGTTTTAAAGTTTAAAGTAAAGACTCTGAAGAGGAAAAGAGGCTACTCCAAAGAAGTCTGCTAATGGCAAGAAGAGACCGGCTGAATCAGCTTCAAAGACTCCTATTCCAGAGAAGAAGGCTAAGGTTGTAACCCCAGCTGGACAAGAAAATTCAGGTGGTGATGGAAAGAAAAATGTTCATCTTGCGACTCCTCACCCTGCAAAGCAAGACAAGGAAAACACCAGCAAAGAAGTAGAAAAGGCGAAGCAGC

>UN00119

TTTGTATTATTGTTGTAATACCATTGGCTACATATATGTAAGGCAGGCGGCAAAGGAGCTTGGGAAGAAAGCAATATATTTGGGTGTTCCTTTTATTGCCGAGTGGTTCAGAAACAAAGGGCATACAATTAAATCACAAGTAACTGCTGCAACAGGTGCAATTGCATTGATGCAGCTGCAAGAAGACATGAAGAAACATCTCAGTGCTGAGGGAAATTATACAGAGGAGGAACTTGAAGAGTACATGCAGTCTCATAAGAAAGTGATGATTGATTCCTTGTGGAAGCTTAATGTTGCTGATATTGAGGCTACCCCTTCCCGTGTCTGTCAGATGGT

>UN00120

AACTTAAAGAACAAATTTAATTAACTACCAAATACGTAGTAATAGATTTCTACAACTATGATATCGAAAGGCCAGAATTTGTTCAACTTTTCATCAAATTTACTCAGAACTTGGCTCTCTTTGGTTCACTGCTCTTCTATCTTGGGATGAAGAATTCAATCCCAAAGCGACAACCCAAGAAAAAGGCTCCGAAGTCCAAGACAGTTTAAGTAGGAGACAAGTTGGGGTGTGTTGTACCCCTTGATGCTACATGGCTATCTTGATAGTTTGTGCTTGTTCTTGTTACATGTATTCGATTCATTTTGTATGGGACTATGGGGCACATGTTTAAGAGATATTATCGGTTGATTAGGACTGCTTCCCAAATACTTCATATGTTGGGAAGTTGAGCATAGACACGGCATATTTGTATTTCTAGGTCAGTTCTGTAGGATTCTGTTCTTAGAAAACAAAGTTAATTAGAAATTACCTAAGTAAG

>UN00121

AAGCTCTCCTTCGCCGCCGCTCAGAGATCCCCGCTGCCATCGTTCGGAGATCGTTAATGCCGCTTGGAGATCGCCGCCACCTCCGCCACTCTCTTCCCTCAAAACCCTAGCATTCGCTGCCGTGAACTGCAGCCACGCCGCTCTCTCCCCTCTTTTCCACCGCTTGGAGATCGCCGCTGCTCCTATTTTCCGCCGTGATTTCCTAAGTCCTTGGCGAAGCTTGATTCCTAAGCTACAGATCGGTGTCACTGCAGGTCCACAGGTTGAAGTTTCTTAAATGGCTCTAGCTTTAGTTTCTTACTCAACAAGCCTCACAAGATTGAGCCTAGAATCAGGACCAAAGTTCCCGTTCAACAGATCAAGAATCTTGCCTCAAAGTTACAAAACTTAGGTACCTCAAAATTCGTGCAGTACAAGAAAGTGAAGGGCCACGGAGATTAGTTGACCATTATTCGAATAATCCCCGACATCTCAAAGAACTATTTCAGGAGGCGTTCAAGGAGAGCATTGTTTGGTGGCATCTCATTGCTCGGTGGTTTCTATGTTGCTCAGACAATATCTCTCTCCTTCGGGGCTTTGGGTGTGAACGATGTTATTGCTGCTGTGATTTGTGTTCTACTGACGGAGTATGTTACTAGGTTTTACTATAGTCGGCCGAAGGTAACATTTTCTCTTGCACTTTTAAATAATTTCAAGATGGGATTCACGTATGGTCTCTTTATAGATGCATTCAAGCTTGCAAGTTGAGTGCAGTTGCAGATAAATCAAGTGACAGGTTCTATTTAACTGGATCTTTGGGTGGTTTGCTTAACAGTTTGAAATGATTCTTTTGGAGTTCCATGGGGAAAAAAATGTTTATCTATAGATATAGAATATCTTCAAGGAGGAAGGAGGGGATGACATCTGCCACATCAGTATTAATGCAATCACTAATAATGTAGTCAACTGCCTATTAGTGATTATCAGAACATTCACAGCTGCACTAGTAGACAATAGTGTCTGTACTAATAGACATCAGTGGTATCTCCATCGAGATTTGTAATATGACAGGTGTCAGTGGTATCTCCATCGAGATTTGTAATATGACAGGTGTGAACCTCGCCTTACTCTATATATGTGATCCGTCTTCTAATT

>UN00122

CTATTTTCCCTAATTTATTGAAATCTCATTATTTTTCAAAAGTGTTCAAAACATGAAAAAAGTGATATACATGAAGTTTGATGTTTTGACATAAACAATATTTTGAAACTTTGCACAATGATTATATAGACTTGATAAAATATGTATATGATGATTGTTAAGTCTATAAACTGAGAATACAATATTTGAGCTTAGAAACCAAATACGTGTGTGTATATGATATTGAGCTTAGAGTATCTACCACACATAATATAAAACCTAAACTAGATTACCCAAGCCGATATTGATCTTCAATAAGCTTGACTTGGTACACTTTGCAGATGAAGCTTGAACTTGATCTTTAACTTGAATTGTATATTGCTTCACTCCACAATTCCGCCAACCTCCATTTG

>UN00123

CCTAGTCCAAAAACATTTCACCACAATTCTAGATTAATAAATCCCTCAAGATCACAAAACAATGAGCAAAAAATAACTATCAGCCGCAATGTACAACACCACCAACATGAACAAGGAGATGCCAAGAGAGACACAGAAATGTCAAGAAATCTAACAAACAAATAATAAACTTACCAAAGTACACATAACATTAAAATGGGCAAAAACAGAAAAACAAAAACACAATATAATAACAAGAACGGCGCATTCAAGCCATATCAACTGCGATTATTCTGTAATCCCTGGCGATGTTTTTTAGAGTCCTCACCCCCACCTCATAATCGCCGAACCCCAAGTGAGGCCTGCTCCAAATCCAGCGGTAGGACAAATAGGTAGTAACAAACCTTTAACTT

>UN00124

GGTTCGTTAATTTAATTTTCCCTTATTCTACTATTCCCTAAAACTTCCCCAGAAAAATTCATATCTCTCTCTCCCCCTCCAAGAGCCTTCCTCCTCCGTCGGCCAACTCTGCCGACCAACTTCGTGAGGCAACGCTGCAAGGGGTGATCTCGATAGGTCTAGCTGATTATGTTGGTTTGCCATGTCGAATAGCCTAAGGATGCAAGTACTGCACATTGCCGATCTTCAGGAGACGCTGCCACCAATCTTCAGGAGACGCTGG

>UN00125

TAACCAAGGTTACTAGAATACTACAAGTAAAAGACCTCCATTTGCCCCCTCACAGTAAAGAGCAGAGCATTGAAGGCTGCCACGGTGGCAAGCGGAGCTCCCATCCCTTTGTATAACCCTCTAGGGCCTTCTGCAGCAATTGTCTGCTTCACTGCATCCATGGCACCCGAGTACTTGGGTGGCTGACCTGGAAGTGGTGTAGGTTGGCTTTGAAGCTTGACCTTGATGGTGTCAAAGGGGTGCCCAACTACCAGTTGA

>UN00126

GGTTTTGAATTGGGCTACTCAATGGAGAATCCCAATTCTTTGGTACTTTGGGAAGCTCAATTTGGTGATTTCTCAAATAAGGAGCTCAAGTAATATTTGATCAGTTCTTGAGCAGCGGAGAATCGAAGTGGCTCCGCCAAACTGGGCTTGTTGTATTGCTTCCTCATGGTTATGATGGCCAACGGCCCAGAGCATTCAAGTGCACGATTAGTAACGTTTTCTCCAGATGAGTGATGATAACCCTTATGTTATT

>UN00127

TAGTTTGGTAAGGTCTTAAGTAATTGAGGAAGCACGAACTTTGAATCAATATAAAATTCCAGAGAAAAACGAAAGAAAAAAAGAGAACGGATCGACATGAAGACGATATGAGCATGTAAGAACTTTTGAAAATGTCATGTGAACACAAGAGGTAACAAAGCACGATTCTTCAGATGAAGAGGATGAAAACTTGAGACAAAAGTCAAAGAGAGGAAAACTCACTCTCTGAGCTCAAAAGACTTTATTGTTTCTCTTTAGTTTGAGGCCCGTTTATTTATA

>UN00128

CTTGTAAAGAGAAACTTGTGGTTTGCTAAGCCAGCTGAGCTTTTATACTGGAGTTACTTTCGGCAAATGCTCACATACCGCAGGGTTGTAGACTACTGTTGTTGAGACAGTTCACCGATTACGTCCTTTACAAGAGGTTTCAAAGAAGCATGAGTACAGAAAATGTTGATATTTATCGATCACCGATATACACAACACTCCTGATCAAATAAGCGCAACACGGTGGTGGTAAGTTATGAAGTGAAATGTTACATTGTGATAGCACG

>UN00129

CGTAATAATTGCATTCCTTGATCGCAGATTTGAGCCAAGAACCTAAAACTAAGTTAGTACAAACTGCTTTAATTCCACATTCATCCTCATCCCGATTTGGTAGACACAAACAACCACAGTAAAATCCCATAGCTAATCTCTAAAGCCTAAACAAACCGCTTTAATTCCAAGCACTAAAAACAACACTACTAGGCTTTAACCCAAATTCTTCTAACAGTAAAACCCTCAACCCCCAAATCCATAGGAGGGTTCGGCCCTGTCGCTTGAGGGCGTAAACAACATCCATAGCAGTAACCGTCTTCCTCCGGGCGTGCTCGGTGTAGGTGACGGCATCCCTGATGACGTTCTCGAGGAAGATCTTGAGGACGCCACGTGTCTCCTCGTAGATGAGGCCGCTTATCCTCTTCACCCCTCCTCGTCTCGCCAACCGTCGGATCGCCGGCTTCGTGATCCCCTGGATGTTGTCTCGGAGAACTTTACGGTGACGCTTGGCTCCCCCCTTGCCTAGCCCCTTTCCTCCCTTTCCTCGGACCCGAACAATTTTTAGTTAGGACGAAATAACCTTTTAAGAAATTTAAACCTAAAACGGGAAAAA

>UN00130

TAACAACCAACTACAAATACGAAATAAAAATAATATAACTTAAAAAGTTGTGACAAAAACATTACTCAAACCCACATAAGTTTATTAAATACCACAATTAAAGCACTAAAAGACCCAAGTCCAGCGGCAGCAGATAATTTAGGTAACATAACATTAGACTACAATCTCCTTTTGGAGTTCTGAAAAGAACAGGCTCTAGCACTTGATCTCCTTCAGCCCGTGGGCAAAGTACAAAAAAGGTAGGATCACTGGCGCCTTCCTTGTAGTATGCAAAGACCAAGCTACCGTCATCATGCATGCTCTCACCCACAAAGAATTGCAAATCTTTGAGCTTCCCAAGCAGGAATTTTGTAGCTCCCTCAATGTTTCTTTACTTTAAAATTTACCTTAACCGTAACTTAA

>UN00131

TTACTACAAAGAGGCGGATGATTTAGATAAGCGGCGGGTACGGAGGAGGAAGGAGCGGTGGACGTGGCGGCGGCGGGTACGGTGGCGGTGGCTATGGCAGCGGCGGGGGAGGTGGAGGTGGAGGTGGTTGCTACTCCTGCGGCGAGAGTGGGCACATGGCGAGGGACTGCCCGACCTCCGGTCCAAGTACAACGTCCGCTCGATGCCGATCCGCAAGGACGACGAGGTCCTCGTCGTCCGTGGGACCTACAAGGGCCGGGACGGCAAGGTAGTCCAGGTCTACAGGCGTAAGTGGGTGATCCACGTGGAGCGGA

>UN00132

TTTTTCGTTATTTGACAAATCCAATCCCGACTCCTCCTCTCTCCTATATCTCAGTCCCGATCCGACTCCTCTCTCTCGTTTAGGTGATCTTGAATCTCTCTCAACGCATGTATCTCTCTTCTCTCCCCTTGCTTGTTTTCTCCTCTCTTTCGGTTGCTCTCCTCAAACTTCTGCAGTTTCTGATTGAGATTCAAGGTCACTGGAGAACCCTTGATCCAAAGGAAAGGCGACATAGCTGCAGTTCTCAAGTCAAGACTCGAAGCTTTCCACAGGCAAACAGAACATGTTTGGGCTTATATAGTGATGGCATATGAATTACTC

>UN00133

TTATAGCGCAAGGTTGTTCCTTCTAGGGTGGAAACGAGTCCAATCTGAACGAATAGTGCGTGCCTTATTCGAACCTGAATTCGGCTCAAATAGCACTACTGGTATTCGTGTTCGGATTCGACTCGAATAATATTCAGAATTCGAATACGCTTCGGTTAGTTGTGTGCATGTTCGGTACCTATTCGCGAACATTCAATTAACAATATTTCGCTTGCCACAGAGAAAAACATTGAGCCGGATTAGGTAGTATAGATGAGAGAGTTCACCTCTGACAATTAAATGAAGATTTTTGATGACTCGAGATGGAATACAGTCGGGGAAGAGGGTCGACGAGCTTCGA

>UN00134

GCCCCTGCCTGGCTTCGTGAAGATTTGCTTGGAAATGATCCCAGAAGCTCAAAAGGCTGGCATATTGCATTTGCCTCCTCCAAGCCCTTTCAACGCACGCATTGAGCTTTGGAATAAAGAGATGAAGTAACCTTAGTAGCACAAAGAAGCTGGCCAGTGCCAGATAGGTATCTTGTTGAAGCAACTTCTCAGGTGATCTTGCCCATGAGAATGGGCAGTTCTCCTGATCTGTGGTTGTTCTGATTTCAGGGAGGTTTAGCGAATTTGATGCTTTCTCCATGGATGATGACTCTTCCGAAGCAGGTTTAGTGCCTGTGACATCACTATAGAAAGCAACAAGAGAACTGATGGTTCGTGAGCCATGATAACGCACCCGCATGGTAGAATTTAGGAGAAAGAGGGTTGGGAAACCATGAACACCATACCGTGAAAGGATGCTTGGCCTGACAACTGATTCTTCGAATGCAAAATGACGGATGGTAGGAACCATGGAAGACATGACGTTTAAGTTCGGTCGGCAAATTTTCGAGAAAGGGCACCAAGAGGCATGAAAGAGCACGGCTACATATTGTTCCCTGTTTTTGTACACAAGATTCAATGCTCTCTGCAGTGCAGCTTCATCCCCCTCAACCACACCAACCGGTTCAAAACCACCGGACCCCGATTCCGTCGCCGAGCACGAATCTCGAGGCGGCGGTGGCAGGATGGAGTCGACGGCCGAAACCCTAGCGCACACGGCCGTATCGGCGGAGACTCCAACGGCTAGAGCCAACCGACCGAAGAATACCAACAGGGCGAAACCTCTCCAGAACCTCGCCTCCATAGCCGAGCACAAGGTATTTCTATACAAAGCGGAAGAGAAACCCTAACCCTAGGCGCACGATAGAGGGAGGACGGTCCCGGCGCATCCTAGAGCCCGTCGCCATTCATGGCGGACGATGAGAGAAGAAAGGAGGGAGAGAATCTCTTTTAGCGCCTTTTTCTCCCCCCTTTTTTTCCCCTTCTTTATTATTTTGAAATTGAATTCTCCCTTTCTTTCTCTCTCCTCTGATTTCAAGGCGGCGACAGAGAGGAG

>UN00135

CAAAGCAAGACCTGCAAAGCTTGTTAGAGATGGCGAAATGTCAGCACCATGTGGAACAACAGTTCTTTATCCTACTACTGGTGGTAATATCCACTGCTTTAAAGCTATAACCCCGTGTGCGCTCTTCGACATTTTAACGCCCCCTTATTCATCAGAGGATGGGCGACATTGCTCATATTTCCGGAAGTTACCAAAAAAGGATCTATCTGATGACTATATAGAATACGCAGAAAGTATTTTACCGGATGGGATGAAGGCTTCTGAAGTGGGTTGGTTGGAAGAGTATCAGCCTCCAGATAGTTTTGTGATCAGGAGAGGGTTGTATAAAGGCCCTGCTCTTAAAATTTAGAGATATAAAGTTGGTGTAAACTTCCATTAGTTAGATTGGTTCCCAGTGGTAACATAGAGTTTCCGG

>UN00136

CAGAGTTCATGTGATGCAACACGTATAAGTGATGGCTACAGGTGGCGCAAGTATGGACAGAAATTTGTCAAAGGAAATCCTAATCCCAGGAGCTACTACAGATGCACTCACAATGGATGCCCCGTTCGAAAACATGTCGAGAGAGCTTCAGATGATGCCAAAGCAGTTCTAATAACATACGAGGGAAAACACAACCATGATCAGCCTGCTCTCAAGAGTGGATGCACCGAACAACCAGCAACAGCTCTTCTCATTGCTGCTGCTGCTTCTGCTACTTATTCAGTAACTAAGGATGAAAAATCACATACTTCTGATTCTTCACCTAATAAAGAATTACCAACCCCAACCAATGGAGAATCAGCTGGTGAT

>UN00137

CCCCGAAGCCATTCCTTCTTCCGTTCTTAATTTCCCGTAATCGACTGTTCGTTTCCTTCAATCTCAGCTCTCCTTTACCCCATTTCCCCCCAAACCCTATATTATTCCTTCGTTCTTACTTTCAATCCCAGCTCTTCTGTAGTGTTCCAAGGAATCATGTCCATGGGATAACTTCCGGGTACCCGTAGAAAGGAAGTGAATTCTTGGATCTCAGCAAGAGCATTCTGAGCCCTCTTCTCCGCCATGGACGCCTCGAAATCGAGGTAGTAC

>UN00138

GGAACAATAAATGTGCTTTAAAAAAAAATTGAAACGACGGAATAAGAGGGAAACTAGACTTATAGCTTGTTTTCTGCAGAAAATACGCCGCAGAGAGGAACTGAAAGTGAAGACAAAACATGAAATCAAATAAATGACTTTGATTCAAACGTTTACGATAATATGTGAAAGGTATCAAAATTTCACAACATACGGCTTAATCTCTTTTAAAGCTTTCGGAGTTTCGATCATGGCTGGAGTACTTGTGCTCTGTGCATTCGATTCATAATTCACCAACATCTTAGCCTCAACTTTTAAAGATTTAGTCTTTCCTTAAAACTCCAAATAAAGACTCGAGAGCTTAGGGTAAAAGATCCAGCTCATTGCACAGAAACCGAATTCCACCAAAACTGTCCTTGGAATGAAGTCTCACTTTCATCCTACGAAGAAGCACTCTGATACTGGTGTAACAAATCCGCACAATAAATGCTTGTAGAATATCAGTTCACCTTTGGCTTGGAGAGGCTGCTTAACCATTAATTCACGGAGAAGGCGTCTTAAAGGGAAGAAAGACATTGCCTAGATGAGACTCACAAAGAGACCTTGCTTCTGGCTTCAACACCTTAAGAAATGGCTTTCTAAGGTCAAGTGCAGAGATTACATTGAGTGCCCGTGTACAAAAACGACGGACCTCAGCCCGCCGTGATTGTTACTCTGGCAACTGGC

>UN00139

TTAGGTCTTCTTATGTTGGTGTTTGTACATGGGTCTTTGAACCATTGTATAGCTTTTGCAGCTGCTGAACAGCCCATAGCATGCGCAGAATAAGAGTGACCATGCAAAAGAGCCATGAGCTTCGAGTCGCCTTTGAACACTTCAAAAACAGCTTCTGTTGTTAATGTTACTGACAATGGTATAATGCCTCCAGTCATCAGTTTTGCAAAACACGCGATGTCTGGTAAACAATCTAGCAGTTCAGCAG

>UN00140

TAGACAGAGGCGGAGCAACTAATAATATGCTTACTTCCCCCTTCTTACCATCTTCTTGTCTGTGAAGGGTGTGTGGACGCACACACATGTTCATTCTAAAAGCTTGTGCATTTTTTAGATTCACAAATAATGGCCTCTTTATGTTGCGAACTGTCCCTATTTGCTTCAATTTCTGATGCACTTATCATATCAGCTTATATTCTCAATAATTATAATTCAATGGTTAAATTTCACCTTCACCTCCTCAGCACGTTCTCCCTTACATGGTTTGG

>UN00141

GTTTTTGTTGAAACTAACGTATACTACAAAGGGGTCTCTGCAATCAACCCCATGGAGGTCCTTCTGCATGTTGATTGAGGGTTTGAAGGATTCAGGACAGTGTACAGGTTAAGCTGTATATATGGGAAAAATTGAAAGCAGCAGCTGGGGGTTTAAAACAGCAGAGAACGATCCACTGAGTTCTGGAGTTGGTTACTCATTCGATTATCTGAAATAGGTACTTTCTACCAAGAGCCCAGTGTATTTTCTTGGAGCCGGATATGTCT

>UN00142

GTAAGAATAAATTTGACTTGTTTTCTGCAACAAACGTTTGCATATACAAGTATTATCTGCCTACAATATTTGCTACATTTCAGTTTCATACCACAAGCCAACATATAAAAAGGGAAGAATTTGTCCAATTTGAAACAAGAGAGGAACTAGCTGCTTAATTGTAGAAAACGAAAACAACCCAATAATACAGATAGAAGATTAAAAAAAAATTACACCACTCAAATTTAAAACACAATCAATGAATCAACCATTACTCATCCTGTTCATCTTTCTCATCAGCAGGCATCATCAAGCCCGACTGCCGGATATCCTCAATCCGGAGAACAACCTCTGACATCGACGGCCGCCTGTCCGGATACTGAGCCGCACAGTCGATCGCAAGCTGCAGCAATTGCACCATCTCCTCCTCCACATTCTGATACCTCAAAAGCTCGAGATCGAAAACCTCCGACGTCCACTCCTCTCGGACAACCGACTGGACCCACCTCGGCAAATCGACTCCTTCCTCGTTGAGAGCAGCCTGGGCGGGGGCTTTTCCAGTAAGAAGTTCAAGGAGGAGGACACCGAAGCTGTAGACGTCGGCCTTTTGGGAGACCTTGCGGGCGTCGGTGACCTCTGGGGGGTGCTTTGGCCCATGATTATTGATCATTAAGTAGAACAATGAAGTTTTTGTATGTGCACGATCTGACCACCCTTAGAACTGATATATGGGTATTTAGTACTGCGCC

>UN00143

TTAAAATTTCTTTTATTTTGTCCCTATTTCCTCTTCTTGTCCCCTCATTCTTACTGGAACACCTGTGGATATGGCTGGTAGAGAAGGAAGTGGTCACTCGAGTTTCCCTCATGTTGCACTCAATGAGCGTATCCTTTCATCAATGTCGAGAAAGTCTATTGCTGCTCACCCATGGCATGATTTAGAAATAGGACCTGGAGCACCTGCGGTTTTCAACTGCGTGGTTGAAATAGCAAGGGGCAAGGTTAAGTATGAGTTGG

>UN00144

AACAAATAAATAACCAAACAATAAATACAAATACTAATTATATTTCAATGTCAAATATAATTATATTCAAATAATACATTCACCAAAAAACTAGAAAGATACATAAAATTTTTACATAGATTATAAAGAATACAATTGTTTACATAACTTGATGAACTTTGCATAATTTATAGAATCCAATTGCATTTACTCCTCCAAGTTGTTGAAGCATCATGTTTCCTCCTCCTTGATCAACAACATTGAGAGCAATCCATGTACAGCAACTTTGCTATAGTA

>UN00145

CCTTTTTAACTTTAGTTTTGTTCTTTAGTTTTGTAGTCTACAGCGGCTAAAGCAACCCAAACGAAATTACAAATCACGCCACTGCTGTCTCGTTGCTGCAGATCTTTTGCAGTGGGAGAGAGACAAGGAGTTGAGGAACTCCTGTGAAGTAAATTCCTAGTAAGAAGTAAAGGTGAATCATTTCTTACAGCTCTGAGATCATCCTTAAGCACGTAGATCACCAAAGGTGAGCGAGCTCTCCTCAGTAGGAACATTGAGGG

>UN00146

AGTCCAACAATCAAATCCAATAATATGATTCTTCGGTTCAAATTGTTTTTCTACAGCAGGTTGGGGTTTCCCCCTCAAGGACTTAAAAAAGAGACAACCGGAGATAATTTCGTGGTTAACAGATATAAGACAGAAGTAACCAAATTTAACAAACAAGGCCAAGGAAAGTAGCCCGAAACAACCTCTCGCCCGTAAACTGCTAGCCCATTGCGTACTTCTGGGTCCAGCTCCTTGCAGTAGTTTCATACTTGCTCCTGTCAGTCTTGTACATATGAGCAATCTCTGGAACCAAAGGATCATCTGGGTTGGGATCCGTCAACAAAGAACATATTGACAGAAGCACCTTGGAAATGGTTAATGCTGGGCTCCATTGCTCCTTCAAG

>UN00147

AGTTTCTCTGCATAATACTTATGAAGCTTGAGTATAAACTTGCATTATTACCTCTCTGAGGTGCCTGGTTTCGTGGAACAATTTCGACAAAACAGGTATCTCTAAATGAAGTAAGCAGACAAATCTTTGCCCCAAACTGCAAATAAAATAGTAAAATAAACAATTAATGAGTAGGGAGATATTTTTATTCACATACAGAAGAGGTATCTCTAAACGTAGTAAGCAAACAACTCATTGCTCCAAACGGCAAATAAAATAGAG

>UN00148

TTCGAGTTAGCCGTAACTCGCGCTCTGCAACAATGAGTACGGTCACCGCTGGTGATACTCCCTCCGTCGTTCCCAACATGACCATCTACATCAACAACCTAAACGAGAAGATCAAGATCGAAGAGCTTAAAAAGTCACTCCATGCTGTTTTCTCTCAATTTGGAAAAAATATTGGAAGTATTAGCCTTCAAGACGTTGAAACATAAAGGACAAGCATGGGTTGTGTTTGAGGATGTTTCTTCAGCGACTGAAGCACTTAAGAGG

>UN00149

CTACTTGTAAAGTTCGGAATATTTAATTCTTATATAACTTAATCATCCCTGTGACAAAAACCAGCAATTTATCCTAATGTACGTTAATATCCGAACATAACATTAATAAACATTGGAATCATTAGACATCAAACCTTTAGTGCTTTCATGACAAGCAGTATCCAAAATTTCCAAATAACCAAAACATCAAAGCTTTATCAGCTACCAAGTGCTGCTATTCGATGGGAAGTAATTCTAGACGTATCATTCGTTCGCACTCATGGGAGGATACAAAAGCTATTGTCTCGCTGACTTTTGTTCCTTATGCCAAGAAACAACCTCTTTATAAGACAGATCACCACCAAATATATCGAGAGCGCTTCCTACTGTGACATCCACATTACCCTTGCCCGCCTTCTTTATCCTCTCCATATCAGCCATTGAATTCAACGCCACCAGC

>UN00150

GGTTCGTTCCCTACAGACATCTGAAATGACTACCAGTGAGCTGGAAGCAGAGGATAGCGAAGCTCATTCTCAAAGTGTGTCTGTGGGAGGGCGGCGTAAAAGGCGACAGACGTCAACAACTGGGATGCAGGCCACTGGAGAAAGGCGCTACAATCTTAGGCGTTCTACAGTTGCTGCAAGCTCAGCTGCAGCAACCCAGGCAACATCTGATCAGGCAAAAGGACACAAAGCCGAAAGTCAGCAGCTTTCACATGATAATGAAATCTCAAAGGGTGATCATGTTGGAGAAGGAACTTCCAAGAATGGGGCTGTGATTTCACCTTCATCTGCTTTTGTTGAAGAAAAT

>UN00151

TAAGAGGTCGAAGCCCAGCATTTCAAAAATTTCATCAAGCTGCTTGATTCTGAATCAGCCCAAACATCAAATATCCTCAAAACAAATTCCTAAACCCTCCTGTTTCAATTGCTAATGCCATTGCAAAAACTGCAGCAATTGCAACTGCCAGCGAGGCTGATTCTTCTGAGGCAACTCTCACGAAAGAACAAAAGCTCAAGGCCGAAAGGCT

>UN00152

GCAGTCAACATAGGCCAATTTGATGGACCTTGTGCTTATAAGTTGAATTTCCTTTCTGTTAATCGATTGAAAGAAAGAAGAATCTTCAACTGGCAATTTTGGCATTTGCATTGCTCCGTTCCTTGGTGGCTTTGACAAACGTCTGAAGGAAAATGTCTGAGTATCTAGAGGAACTTAAAGAGTTGGCTGAAACTGAAGGAGTTTCTGATCGTGTTAGGTTTGTTACATCCTGCTCAACTGCAGAAAGAAATGCTCTGCTCTCTGAATGTCTCTGTGTTTTATATACCCCAAAGGATGAACACTTCGGCATTGTTCCTCTAGAAGCAATGGCAGCACAAAAACCTGTTATTGCTTGCAACAGTGGAGGCCCAGTTGAGACGATTATACATGAAACTACAGGATTTCTCTGTGATCCAACGCCTCTAGAATTCTCGAAGCATATGGCTAAACTTATGGCCACTCCTGAAATGCCTATAAAGTTGGGTGAAGAGGCACATCGACATGTCACTGAGAACTTCTCTATCAAGGTATTCGGTGAACAGCTTAACCGTTATGTTCTTGAGACTTTCCATCAAAGAATGGAATAAGATACAAGTTGATACTTTCAAGTTCCCAGTCAAAAGCTCAGATGATTCTGAAATTTATAACCCTCAGTTCTTTGTTTCTCAAGGGTCCGCGATCTTGAACATACTGAAATTGATACATATCTAATGAGTAGTGGACATGCTTCAGTGTATTTATCGAAGGTGTACATACTATTTTCAGGTTAGTGACATTTAGAGGAACTTGTCGCTTGTTTAGTCGATCCATTTGAATTTAAAACC

>UN00153

GTCTCTTCGTTGATCGGCGGAGCTAATCATCGATTTGTTACCGATTGACCACGATGAGCGAGGTTTTTCCAAGGATACGAGCGCCAATACTGCGAGATCTCCGCTTCTCTATCGAGAAAGTGTACATCGGCTGCTCATCTCGATGGTGAGCAAAAGAAGCAGAAGATTACTGAGATTAAATCGGGAGTCGAAGATGCTGAAGGATTGATTAGGAAGATGGATCTTGAAGCTCGAAGTCTGCAGCCAAGTGTGAAGGCTGGATTGCTTGCAAAGCTAAGGGAGTACAAGTCAGATCTGAACAACTTGAAAGCTGAGGTTAAGAGAATTACATCGGCTAATTTGAATAAAGCTACACGAGAGGAGCTACTGGAGTCTGGGATGGCAGACACGCTAGCGGTTT

>UN00154

TCTATTGTATTTGCAATCCGTATTCTCTTTCCTTCTTGATTATTATTTGGTTCTTGAGGTTGTGTTGATTGATGGGATTAACTAAAAAGATTGAGAGTAGTGAGTGAGGTTTGTGCTATTTTTGGAGTGTTGGCTACTGCCATCATCCTATGAAGGAAATCATTGATATGAAAGACCGGTTGGGCCGCTGCCTGCTGTTGTGGGCTCCGTGGGATGCGCTCATTATTGAAATGTAATTAGAGTTATAAAATGTTT

>UN00155

CCCTCATCCCCTCGGGCAAAGGCACAAGCTTCAACCAATCATTCAAATCCCTGAACACATCAGAAGTTATCACGACAAAAAAACCCACAGCCGCCAACAACGCAATCTGAAACGGCTTGTTCTCCGAAATCCCCTGATTAAAAGGCCGTCCCATATAGTTCACAGCAAATGTGGCAACTTGAATCATCATGTTCACCATGTAGGAAACCGTGTTCACGAGATTAGGGTGGAAGTCCGAGTCTGGTTCAATGCATTCCTCCGGCATGTATTTTGATGCTAGGTTAACCGCTGAAATTAGGAAGCATAG

>UN00156

TTTTTTCAGAAGCACAATTTTCTTCTTCTATCACATCATAACGCCTTCGTTGGATAAAACCATCGCCTCGTATGTCGGCTTGACTCTCAAAGATGGGAATCGCCGGAAGCCTCAGCCCCGATCAACTCGAGTTCTTCAATTCCAATGGGTATTTGGTTATTGATTCTTTCTCGAGCTTCGAAGAGATTCAGGAGATGAGGGATAGGATGGACGAACTGCTCAAGGGTTTCGATCCATCCAAGTCCTCAGTCTTTTCACCAAG

>UN00157

CTTGTTCTTACTCGACAGAATAATAAAGTTCTTCTCTATGATATTAGGTTATATAGTCTTCCGAACCTGTTGAATATAGGTGTTGGAGAGAGGGAGGTCTAAGTTCTCTCAACTAAATGATAAAATCTGCAATGGCCTATCAGATATTGCTTTCACTTCAGCAGATAAATCAAGACTACTTGCATCTGGATTGGACGGTGCTATTTATATATGGGATAGGAGGTCGAGTAACTTTCCGTGTGTTGAACTTAAC

>UN00158

AAAGTTAAAAAAAGAAAATTTTTTTTAATATAAAGACCAATCAACTTGAGGTGATAACAACAAAATGACTACGTTCTTTCATCAATCCAACACCTAAAAAAAAAAAAAAACCGTCACAGTCCCTCTCTAGTACTACACATCCATGAAAGAAGAGGAAGAAGGGGAGGCACAACAAGAAAAGAAGCAACCTCTCCCCCCCTCTAATTTATCGAAACGATAGGCTTCGTCGTCAACCCAAACTCAAATGGGAACAAAGGATCATAATGCCCATCCCCAACATTCATGGGCAACTGATCCGCTCTCCTAAACCAAGTCCTCGACAGCTTCCCGCTGAACCCGTAATCTCCAAATATCACGTCAGTAACCCCTTGGCCCTCTGAACCTGGCAGCCACGCAGCGTACTAAGT

>UN00159

AGTGTGGTGATTGTGGTAAAAAGTTTAACATGGGTGGGCCCATATGGTCTGCTCCCATGCATGATCAAGATTGGGTACTTTCTATTCTAGCAAATGTTAAATCCATGAAGGAAAGGTATCCTGCATATGAGAGGATTTCTGCAGTTCTAACAACAATATCTGAGGAATTGCATGATGTCCCTCTCTTTGTCAGCCTCCACAATTTGTGTGGCACTCTGAAATGTACCTCCCCATCTGCTGTTATGTTCCGCTCTGCAGTAATTAATGCTGGATATCGCATCTCAGGCAGCCATGTGAATCCACTTGGCCTTAAATCTGATGCTCCAATGGATGTCATTTGGGACATTATGCGCTGCTGGGTCAAGAATCACCCAGTGAAACCGCAGCCAGCAGATCAGCCGGGAACTGTGATTCTTGCTAAAGAACCAACACTTCAAGCAAATTTTTCAAGAGCCGTCTCTTCCCTCAGCAAGGCACACAGCGAAGAAAGTGGCAAGGTTCCTTCCGAACCCCGAGAGGCATTGGGGTCCAAAGGTCAGGGCTGGGAGGCGAATAACAAGCAAGCATGTCTCATTGTTGGGTGCTGAGGCTCTAAACGGGACTAACAGCCATGGACAGGGCAAGGGAAGTGCAGAAATTGTTGCAGAGGAAACCGATGGGAACGAAAACATAAGCCATGAAGGAGACGAAGGACCTGATACAAAGCGTCAAAAGACTGGGGATGATTAAGAGACTAATCTTCAACATGAAAAGTGTATTATTAGTTTGAGGTTCTTTAACTTTTCTGCTATATGAGTGCTGCGCGAGCGAGTCTAATTTATTTGTTAGACGAGTGTTAATTGTTTAGGGATTAATAAAAAATTCTCAAGTGATTTTGTTTTTGTGAAAGGCAGTTGACGTCGTCGTTTCCC

>UN00160

GTTCCTCCAAATTTAGCAGTGACAGGACAATTCATGAATATGCGAAGGATATCTGGAATATCGAGCCGCTTGTGTTGCCATAAAGAAACAAGATTGGCAACGTTCATTTTTTATATACAGTATGCGCATATTCCTCAAAGAGAGGGAAGGTAGAAAAATAAGCCAATATCTACTGGTTGCAAAAGGTCATCCTTCAAAGTCTGGTCCAATAATAATTTCCTCGTGCCGTTGTCATTGAAGGCTGTTTGCGAACAAGACAAGGTCAGTTTGTGAGATTGTGTCCAGAAATCAGAAAGAATTTTATCTGGCTCGATGTGTCTGCACTAAAATCCCAAGGAATTATGTTGCTGGGAGTAGTTTTGAACTCATTTGAGGCTTTTTAATTGTTAATATTTATCCATAGTTACTCTATGCCCTTTTCGTCTAGTCTTGAATACTCATTTCCTCCCCTAAATTCGACACTCAGTAAAAGTTTCTTAATTGTTACT

>UN00161

TAGAAGCATGTACTCTAATTATAGTTGTATGTTTGTGTTAAATGTGGCTTAGCTGTTGTTCTTGCTTGTGTTGTTCCTATGTGATAATCGTGTTGGTAAATAGAAATGAAAGCAACTATGGGATAAAAATTTTGTTTACCCTCCAATTTGGGGTATTTTCATGTTATCCATGGCTCGAAAAAGTTTTTATGTTGTGAAGAGAGGTCGACGATGTGGTATTTATAATAATTGGGAGGAATGCAAATCTCAGATAGATAAGTTCCCAAATGCCTCCTACAAAGGGTATGCAACATACGAAGAGGCTTTGATTGAATGAGAGAAGCACACACTCGCTCATGATTCAGCATCCAGCTCGAGGCCTGTCCAAACATCTGTTTATGCTCAAGAACAAAGATATCCAATGAGCCATAATCTGGATGTGGACAAGGAAAAACTTGGGGAAACTTCTATTTTCTCTATCTATGTTTTTGTATTAGGTTCATTGTTTACAATATTAGTATTTCTAGTATGTATCGTTGCGGTATTTGTTGGTTTCTTCATTGCAAAAAACATGTGATGTAATTTGTGGCTGAGCTTGTATTTGTTGGATAAACTTTTTGTTTAGCTCATGTTAAATTGATTTTTAATCAGCTAC

>UN00162

TTAGTTTTACTTTATTTTCTTTAAGTTTATTTGTTATTCTACCCCTTATGTCAAGAATTGCAACCTAAGTGAATAGATGGACATAAAAAGGGGACTCGTGGGACCGGCGCAAAGTTGGGAGAAAAGAGCTGGTCGATGTCATTCTTCCTCACTCTATGCTCTGTTGCTGTCATCTTCATCTCTTGATTTGTTTCATTTGTTTCTTCTTCCTCCCTTTTACTGTACTTTCCAATTTATGTTGACGGCTTGTGGATTGCTTACATGTATTAGTTACAGTTACG

>UN00163

CACCAACGCAACTTCGATGAAACATCTTCAAACATGACAAGTTACTAACCAAGACATACAAAAAAAAACCACCCAAAAACAAACGCACGCATAACAACTAGCTTAATCTAATTAAAATCGGTACCCCAACAAATTAAAACAAAAGACTAACAAATAAAGCTTGCTTAATTTATTCTCGAACACCGAAGCCATGACCCGGTTCTAACAAGGTCCAGTGCAGAAACATCGGCGACGGAGTCCGCGGCAATCGCCGCCTGGAAAGCCTTCGGTCTGGCAAACGCTAGCGCAGTTGGATT

>UN00164

TTTATTGTTGTAGAACAAAATCATTTTTTAGGAAAATTATAATAATTTAAAAGGAATTATCTTGGAAAAAGAATCATTTTAGAATAATTATAATAATTTTTAGAAATTATTTTGTGGAAGAAATCATTTTAGAATGACATATTTTAAAAAAAAGAAATTATGTTTGGGAAAATAGACTATTTTGAATTAATAAGAATTTATCTTATAAAAAGATTATTTTTGAATTTATTATTTTAAAAAGATTATTTTTAGAATTAATATAGTGAGAAAATAACTTATTTTGAAAAAGGGATTATTTTGAAGTAAATAACTTTATTATTTCAAAAGATTATTTTAGAACAATTTTA

>UN00165

TTTTTTTCCTTTTTTAATTTTTTAAATTTTGGTTTACCTTTAACTTTTGGTTAAATTTCTAAGTATTAAAAGTAAGGTTAATTGAAGTTGTATAATTCTGACTATTTCCTAGAAGAATACGGATCCATTACACTAGGATTCAAATTTTTAATTCTGATTATATTGTGGAGAAAAAAAGAAACTAACATACAAAAAAAATTTACTCTGACAAACTCATAAGACAAAGTCAAACATAAGCTTATAGTGTCAGTAGGCTACAATATTCTTGACAACAGGCAACTACAACAACAAGGAGCTTTCCCTAGACCTTTCGGTTGGAAGAAGGAA

>UN00166

AACCTTATTTGGTCTAACTTATTCTACTTACGTTATATGTGTGTTGATCACGATGGAAACTCCAATAAAGTCCCCAGGGGAACTTCACATTCTCAGAAATAACCATGGCATCTAGAAAGCTAATGGCTAACGAGAGCACCACTTTCAGAGATAGAACAGATGAGATTCCTCTAGTGTGACTAATCCCAAGATGTCACAGATAGAGCAACCTGAAGATCATTAGATGTGCCACATATATACATTCTATGCGTCTCAATATCTCCGGTGATATCTGATGAATTGCTACTTCCGTAATGATTGTCACTAAGAGTATCATAGACTGAGAATATAATCTCCCTCAAGGCTGGTTCGTGACCATTAAGAAGGTTACCCATGCTCAAGACTTTCCTCCATGATTGGTTAGCTGTTGGAGGTGGAAAAAGACTCGCTACAGCATCTCTCTCACGCAACTTAACTAGTTGCTTGGTGTTGAACTCCTTATCATAGCAAATGCTTGCAGAACTGGTCCCCATTTTAGGAAGATAATAATTGAAATAGTCATTGAAAATTGCTTCAGAAAGACGTGGGGCATCCAACAGCTTACGACGCACTGAAGCTGCAAGGATCAATAGCTTTTCTATGTGTTCGAAAACACGACATAGTTGGAATAGGTCTCCAATCAACTCAGACTTTTCAAGCGAATGATTACCTTGAAAACACTTCAACGTAGAATGCCAGTGTCAGGTATAGCTGGTCAAGTTTTGTTTTCATAAGTTTCCAATCACCA

>UN00167

AATTTTTATTTTTCTTTTTCGTTTGTTCCTTATTTGTCTTGGTTGCTGGTGTGCTCTCCGGTTATGACAAGCTTCAGTTAATCCTCTTGGGAAAGAAGTACGGTTCCGGTGGAGCTTAAGTTGATGGTGATGAATAATATTGAACTTCATTTTCTTTCCTAAGTTTTGTTGGATCTTGATAGGACAATTTCTATTGATCCCCCAATAATTTTACTGCTGTGGTGGGAGATTCTCCCTTAATTTTTTTGCTATATGGAGATATGATACGTTCTCCGTAAACTTGTTATTTTTCCTACTGTAGTGTTTTAACTTGAAGTGTTATGTACTCTAATGCTGCGAAGTTCTATCCAGAACAAAGTAGTAAAGCTTTGGGAGTTATAGATACCTTAATAGTTTAGTTAGTAAC

>UN00168

ATTGGGCTTCTTTGGCTTGATATTGCATTGCTCTTCCCTCAATAACAATGATCCATGCATGGCGGGGTGCCGCAACTGCTGCTATGGATGGGGGCATCCTGGACTGTTTCCCTGCTTCCATGGAAGCTTGCTTTGCCTTGGTAGTTATCTTTGTTGTTGTTTTTGCCATTCTAGGCATTGCATATTGGTTTTCTTGCAGCTTACTATGGCCGTCCA

>UN00169

GTAGGTTCCTATTTTTCTAACTTCGGTAGTCCCCTTTCGATAAACGAAAGAACTCCCTTCTTTTTCTCTTTGGTTAAGCCCTCTCATGGTTATAACCAAAGTTGAAATTTCATTTCGATAAACGAAAAATTCATCTAGATAACCAATCCTATAAAAGGTAAGCTTATTTAGATCTTCCGGATTCTCGGAGTGGAGATCCCACTTCTAGTTTCTTTCTCGCACTGGAAATAGAATATAAAATTCTTCCTTTCCAGAATCAGGGAATAGAATATAAAATTCTTCCTTCCTTGTTTTCCGGGTTTGCCTTCACCCTAAAAGAAGACCGGGAAATAGAATAATAAAATTCTTCCTTTCCCGCACCGATCTTTCCAAAATTTGTCAATATAGGATTCATCCTAGGAAGTTTATTCCGAATCCTAAGCAGGGATAAGTCTGCGCCTAACACGCGCTGCCGGTTTAGTCTTTTTCCGCACAAAAAGACACCAGAGTTCGATTCTGGGGTATTCTATCCTAAAACCTAAGTAAGGA

>UN00170

TTGGTTTCTTGTCCGTCATCGACCGCAAACCCTTTCTAGGGTTTTCAGCGTCCCCCTTTTCTTTAATTTTCTAGGGTTTTCGATTCTTCTTCGTTCTTCACTCTCTCTCTCTCTTTGATTTCAATGGATTCCTTTGGCGCTGGTCGGAAGAGGGGGAGACCTGATGCGGCTAATGGAAATGGTGGATTTGCTGGATCTAAGCGATCTAAAGAAATGGACGCACAATCTGGTTTAGGAAGCAAATCGAAGCCATGCACCAAGTTTTTCAGCACGGCTGGTTGCCCATTTGGCGAAGGCTGCCATTTCCTGCACTTTGTACCTGGTGGATACAATGCCGTTGCCCAAATGACAAACATGGGAAACCCTGCACATGCCCTACCACCCAGAGGCCCTATGGGACCTTCTCCCATTCCTGATGCTCATGGGCCACCGCCTGCTGTCAAAGACTA

>UN00171

TAAGGAAAATAATTCAATCACACACAAATTGTCAACTCCTTATCACCCCCAAACCAGTGGACAAGTAGAGGTCACAAACAGGCAGATCAAACAAATCTTAGAAAAAACAGTTAATCATAACAGAAAAGATTGGTCTGTAAAATTATGTAATGCTCTGTGGGCGTATAGAACAGCTTTTAAGGCCAATTTGGGGATGTCACCATACAGAATGGTATTCGAAAAAAGCTTGTCACCTACCAGTAGAGTTAGAACATCGAGCAATGTGGGCGATAAAATAGTTAAATTTTGATTCAGACTCGGCAGGTAAGAACAGATTGCTCCAAATAAATGAGTTGGAAGAAATTCAGAATGATGCGTACATAAACTACTAAAATTTT

>UN00172

AAGTTCGCAAATGAAACGGTACACTAACTTCAATTTATTCATCTCAAACACACAGACCTTCTCACGCATCGCTATAAATGTAAATCTCGAAACTTAAACAAAAGAAACAAACCGATCTTCCAATAAATCAAGAACGGAAGAGGACGAGGAAACAACTTAGGGAAGAAAATTGTAAAGCAGCAGAAAAAAAAAATGAGGAAATATAACCATGTTCTCCATCACTCCGTTCTTCAGTCTTGATGTGTTGTCGTTGGCAGCATTCTCTGCTTCGTCTCCTAAAAAGCCTAGTACCTGTAAGTAGAAGGAAGGACTTAGTAAGGGAACCCTAAC

>UN00173

CGTGCCGGATGATGTGGTTAATGGTGGGAGCAAGAAGGGGATGATGAAGACGAAGAAGTAGGGATGGGATGGGCTTCTTGGTGAGCAACTGTAAGAGATCTCTTCTGTTCTGAAGTTTCAATTTTGTTATCTGGGTTATGGAAGTAAGCTTGAATTGAGGTTTTATTTTCTGGTAATTGATGTTTAGGTTGTTGTTTTCAGTATAAAAGTTTTCATTTTGTCATGTGATATCAGTGAAATGCTTGATTATTCTAATTATGCCTAGTTCTTATATTGATTAAATAGGAAAACGTTGTTAC

>UN00174

TGACGATCCTGAGCATATGCAGTGGGTTTACTCGGAAGCTCTTAAAGAGCTGAGCTCTTTGGTATTACTGGTGTAACTTATTCTCTTACCCAGGGTGTTGTGAAGAATATTATACCTGCTATAGCTTCTACAAACGCTATTGTGTCTGCTGCCCTGTGCTCTGGAAGCCCTAAAGCTTGTCTCTGGCTGTAGTAGAACATTGTCCAATTATCTCACGTATAATGGTCTGGAAGGAACTCATATTAAAGTAACAGATTTTATCAGGGACAAAGATTGCCTTGTTTGTGGACCTGGAACTCTCATCGAGTTGGATTCTTCTGTTACACTATTACAGTTCATGGAGCTACTTGGGAAACACCCTCAGCTGCGTCTTTCG

>UN00175

CCACGTTCGATCAATCGCCGAAGAACCTCAGATCCCTTTTCTCTTCAACTCTGCAGGTTTAGAGGTTTAAGATGACAACAGCAAAGATCATTGGAGCTCTTGCAGGATCCTTTGTAATCGCATATGTTTGTGATACAACAATTTCTGATAAAAAGATCTTTGGAGGTTCCACTCCGAGCACTATCACTCAAAAAGAATGGTGGGAAGAAACCGATAAAAAATTTCAGGCTTGGCCTCGAACTGCTGGACCTCCAGTCATCATGAATCCTATTAGTCGACAGAACTTCATTGTTAAATCATCTGATGCTTAAGGTGTGAGGGGGTTCTTTTTCTCTGGGGATGTTGCTTTGATAACTTTCAAGTTTCGATCAGAAGAAACATAGTTTTTATTACTTATGTTTGTTTAGTGGGATTACACTGTAATAATATGCAAGTTTTTATTACTTATGTTTGTTTAGTGGGATTACACTGTAATAATATGCAGACATTTTAGTTTTTACATGCAACTATTGCTACAATGAAAATTTTATTTTGTTAAGTACCGCACACATGAATTTTTCGTGCCAGTTGAGCAAAAATGCTTATCTGTTGTG

>UN00176

TATGGATTGAATACATCCATTCGGAAGAATTTTGAGCGATACAATCACATCCTTAAACACATGAGAGATTGAGAGTCCACTTATCCTAAAGTGAGGTCTACCAATTTAACTACCTGAAGGTCTCAATTTTAGTACTTCGGGATTGAAGCTAGTTACATGAATTTCTTTTTCCTACTTTGACTTGAGAATTGGATAGGATGGCTATCCTTGACTTGTGACGCTTGTGATCGTAAATAAAATTTTAAAATGTGATTTTGTTGGGAGTTTTTAAAATTACTTTGTTAAGTCCCGATGAAACGTATTTGTCTTGTGATATGTTTGATATTGGTTAAAGGATTGATGGAGGGGGTGATCTCTACTCTATTTGGCTTCCACTT

>UN00177

TTTCCTTAACCCTAAAATCATGCGTCAAGGAAGCGATTACAGCTCCCAATACTACCCCTACCACCATCCAACCCCTCTCCCGAGCCCTAACCCTAATCCTCCCTCAAACCCCACCGAATTCGCCCCTTCCAATCTGATCCAGCCTCCCTTCGCTTCCGCCCCTCCTTATTCCCAGCATCCCCCATCGGAGTTCTCCTCCTCTACTTATTCCTCTTATCCCCAGAATCCCGATCCTGTCCCCCAATCTCCCCCCCTTCAGCTCCCTCTTACGCTCAACCCCAATTCCCCCAATCTCAGC

>UN00178

TTCGTTATTGGACGAAGTGGACGATTTGGGAGAAAAGGTGTTGCTATCAACTTTGTGACCCGTGAAGACGAGAGGATGCTCTTTGATATTCAGAAGTTTTACAACGTGGTTGTTGAGGAGCTTCCATCTAATGTTGCGGATCTTCTGTGATCGAGTCTAAGGAAGTATTTCTGATACATGTTTGAGTTATTCTAGGTTTTTTTTTCTTGAATTTATGAGTTTCTTTTCTTGCACTCCTGCGGCTTAGTGCATGGTGTATGGTGCCTTGATAACCTTACATGTAGGGTTGTTTTGGGTTTTGGGTCTTGGTTAGGTCTGATATGTGTTACCTTATTTCTTTTCAGGTTATTTATGGACTAGTGTAGAACTGTACGCCACAAATTCTATTGAGCTAATTTTACTTTTTTTTTGTTCGGTATTTCCCTTTCTTTTAAAAAAAA

>UN00179

TAATCTCGCCAGTGAGTTTTAGCTGTAAGACAAACCTGAAGAAACATCTTCTTCCATCATCTTGCGGAATGCATCCCGATTTTTGCGTTCTGCCCGTTTTAATTGCTCCTTCTGCATCTTTTTCTGCTGCTGCTCCTCTTTCTCCAGATCATGAATATACTCCTGAAAAATCTCCAGTCGATCAATTTTCTCAAGTCGAGAACATCTCTCATCATCCTCCAGGCGATCCTGAACTTTCCTCCATTGACTGTTTGCCTTGATGAAGTCGCATGATTCAAGAAAAGTTCTATATTCTGCAATGTTTCGCTTGTGCTCTTCTGCTGCTTTTGACTCTTTCCTTCTTCTGAAGCTCCACCAGATAGTTTTCAAAGAGATCCTTGCGGTCTCCAGGCCGACTAC

>UN00180

ACTTATTTGGTTGTACCCTATTCTAGGTAATTCCTTGGAGTCTAAAGTTAATGAAACCAAAGCAATGGTTAAGTTTCAGCTCAAGAAAGTTCTTTGCATGGGTGTTGCTGTTGGAAACCTTGGAATGGAGGAAAAGCAGATTTTTCAGAATGTGCAACTCAGTGTCAACTTCCTGGTGTCATTGTTGAAGAAGAATTGGCAAAATGTTCGATGCTTGTACCTGAAGAGCACCATGGGCAAGCCCTTTAGGGTGTTCTAAGCAGGATTCAAAAACTTAATCTGGAATGGAGATATCTGAACTTTTCTTGAGAGTAACACCTATCGTTTTACTGCACTTTTGAACTTGATAATTGTGGAAAGGAGATGCCTGTGATGTTTCTGTTAGACTCGGTCTTGTTATTTTCTTAGTAGAGC

>UN00181

ACTTAGTTCTTACTTCATGTAAACTAACACAAAAATGCTGGGGTCTGTACAGTTTTTGTGACCGTTCTGGCAAGTTACTGCTGCTGTTTTTTCTTGGGGGAGCATGGATGAGACTGGTCGATACCGGAAGAAAGAAAGAAAAATGATCTTTCTGAACTTTGAGCTTATCCCTTTTGAGTAAAACTCAGCTCAAGATTTCTGGGGTTATTTTCTTCACCTTTCAGCTATATAGGAAAGCTAGTTTCATCAAACCTTCAAACGAACTAGGATTCTACGGTGAG

>UN00182

ACTACTTTTTACTACTCGTTCGAAGCTCGACGATCGCCAGCGATCTCCGGAGATACTTTGAGGTTCTAAGATTCCAACCGAAGATTCCACCGATTCGTCGAGAATCTCTGCGATCAGATGGCGATTTCGAATCCAGCTCCGGATTCCGCCAATCTCACCGAGGATCGCGATCGCAAGCGGAAGAGGGGTCAAACCCTCAGTAATCCGTCCTCGTCTCGATGGAAGAACGAGAACGAGCAAAAGCTCTACTCGTCGAAGCTTCTAGAAGCCCTCCGCCACGTCCGCCGCTCGAATCCGACGGCCGGGGTACCCCCGCGCAGCCGCCTGGTCCGCGAGGCAGCGGACCGCGCCCTCGCCATCGCCGCCCGCGGCCGCACTCGCTGGTCACGTGCGGTCCTCGCGAACCGGACGCCCAAGCTCAAATCCCGCCGTCCGAAGCGAAGACCGGTACCGGTTAACCGGTTAACCGG

>UN00183

ACCTTACCAAAAACCAATAGCTGCTGGATAAGGACCTGGTCCAAACTGGGGAAAACAGTGTGGGATTCCTCCACTGATAGGTTTATTCCCATTGAAAACAAGCATCTGGTCGAACAAAGAGGAGATCTTTGCCATTGGCAAGCTTCCAAGATGTGATACATCCACCAAATAGATAAATCTCAGCTTCGCTGCCACGAGAAGAGGTCAACGAGACCTTGGGAAGCCCGCCATTGCCCTCGCTGATCTTGACGCCTAAAG

>UN00184

ACAATAAAGACAATAGCCTGCTTTATTAATAAATTGAAATTTTTGAACCCCAACCACAATCACCACTCTTCAGCCACGGATAAGTTGTCGATGCCTCTCACTGCAAATAACAAGTTTAAAAAGTTGATAAACTAGATGCTACACTTGTTAAAACATCAATGCCCATCATTCACAGAGACATGCACCAACTATAATCTACAACAACTATGAAAAATTCATCAACAAAAGGCATAAGATGGAGTACTCATCAATGAAGAATCTGACTGCCGTAATCATCGTTTGTAACTTTCTTCTATAGCGGTGAGCACTGGAGATGGGCTGATCGGCCTTGTGTTCTTCAGGGCTTGCCCTCCTAATAATCTTTGGGTGTACAACCGGAACCAAACTAAGAACCGGTAAACTAACCTAA

>UN00185

TCTATATCTATGTTTTGATCCAAACACTTGGGAAACGGTTCGAAAGGAAAATTTTGTGTTGCATTATGACGCAGTGGAGAAGAAACCTGTAATACCTCAGCATTAGTCAACAGTTTTTTCTAATATAGACTTGCTCTAGTGTAGTAGTAGAAGTTGATGGCCGAGCAAGGGAACCTCAAGCCTCCCATCCTCATAAAATGTTACCTATTTCACTGCAAGTTCTTGATGTACATTGGAGATTGCTTCTTCACTCTTAGATTTTCTTTACAAGTGCGGGAGTTTTGTGTAAATAAGACTCGTCAGCCGTTTTCTTTTTTCACTTTGATTTGATTCAATTCGAATTACGAGTGAAAAATAGGTTTGTGTTTTCCTACACAAACATGAGCTATGACAATACCAGTGAGTTGGAATCCAAAGGCTAGGCTTGGAGCTTTACATGATGCCGATGCTTGAACTTTGTAACTTTGAAACTGGCAAAGAGAAAACTGTACCTTTTCTTGTGCTTGGTTAGCCTACGATGATCATTTTTCGCGCTGATGGTTGA

>UN00186

AATGATATGGAGCCTTCCAGTTCGTGTCCCAGTCCTGGAGAAAAGTTAATAGTACCCAAGTACCTGGAAGAAAAGTTATCTGATGGGGAAGTCCTTCCGTTAGAGCTCACAGATGAATTTTCCTCATTAGATGGTGAAGTAGGCAAAAGATTAAATCATATGGTCCCTGTTCCTCATGTTCCTAGAATCAATCGAGAAATTCCATCTGTTGATGAAGCGACATCTGATCATCAAAGGCTGCTTGATAGGTTG

>UN00187

GTTAGGTTAATTTTTCTTTTCTTAGTCTTTGTATTACCCAAAGGCAGAAGCAAGCTCGCTACCATTCATACAATCAACTCGCTTGCACAGAATATAACCAGTTGTCTAGGGATGCTCAATATGGTGGATCTAATTTACCTGTGGATTCAAGAAACAGGAACCTATTTCCAAGTGAGGACTATCCAGCCTCAACGGCCCCATGTATTTACTTGGTGATCTTCAGCATCAACGACATGATGATTTTGGGAGGGCCTATGGCCG

>UN00188

CGTCGGTACTTGTTCGCTATCGGTCTCTCGCCGCTATTTAGCCTTGGACGGAATTTACCGCCCAATTGGGGCTGCATTCCCAAACAACCCGACTCGCCGACAGCGCCTCGTGGTGCGGCAGGGTCCGGGCCGGACGGGGCTCTCACCCTCCCTGGCGCCCCTTTCCAGAGGACTTGGGCCCGGTCCGTCGCTGAGGACGCTTCTCCAGACTACAATTCGGGTGGCGCGGCCGCCCGATTCCCAAGCTGGGCTATTCCCGGTTCGCTCGCCGTTACTAGGGGAATCCTTGTAAGTTTCTTCTCCTCCGCTTATTGATTATTGTCTTTAAACTTCTAGTCGGGTTTGGTTCCCCGTTCC

>UN00189

GAACATTTCCTGCTTCGTCTTTAATTGGTGCTATAGTTAATAGATTCCAGAAGGGTGTCCCATCCTTCTTATAGTTCAAGATGCGACCACAATAGTTTGTACTGGCTGCCAGTGCTTCTCTTATCTTAGTAATCTCTGCAGTGTCAGTTTCAGGACCTTGAAGAATCCGACAATTCCTCCCAATAACTTCCTTCGCCAAATACCCAGTCATCTTAGAAAAACCCCAGCACTCGCGTACATCACAGGATAATCCGCCCTCCGTCGCATCGCACACAACAATGACTGTTAGAAATGCCGACAAGACCTCCCTCAGCTCCTCCGAAACCCTAGGCATCCCGACCCTCTCCTTCCGTCCTCCTCCTCCGCCTCCGTCGGA

>UN00190

CTGAATGGTAAAACCAGGAATTCTGACAGCACGCAACATCTATATAACTCAGGAGCCAACATAATGAATATGCTTTTGAGATAAGTCTGAAGAAATATTGGCCTAAAAATCGAAAAGAAAAGGGGGGAGAACTGTAACAAGGCAACCAAACACCTGCAAGTTTACTTTTCTGCTTCCGCTGTAATTCCAAAGGCCAACTCAAATATCTGGCTGTAATCATCTACAAAGTGAACCTCAAGTCCTTCCTTCACATTGTCAGCAAGCTCAT

>UN00191

CGAAAAAAGAAAAAGAAACAAAAAGGTAAGAATAGAATAGATCTCCATTACTATTCCTTCTGCTGCTCCTAGGCCAGCCGAGAGCCCATGGGGTAGGTGTATCCAACCCCGTGACAAACTTAATGAGGAAGGCACCAGCGATAGCACCAACGAAGCTGAGCAACCCAGTAGAAGATGCCGGTCAGGACAGTGATCTGCGCCGAGAGCAAGCCCAAACGTCACGGCCGGGTTCACATGTCCGCCGGAGATGTTTGCGCCAACAGACACAGCGACGAAGAGGGCGAGCCCGTGGCAGACCGCCACGGCCACCAGACCGGCAGGGTCAAGGGCAGCGTTGTCCGTCAGCTTGTTGTAAGCAATGGCCGAGCCAACGCCAGCGAAGACAAACAATAGAGTGGAGATAAATTCAGCAAGGTAGGCTTTGAGCGAGCCGACACCGAAGGAATCGTCAAATCTGCCAAAGGCAATTCCGGCCATAGGACTAACCCAAAATTTAAATAAAATAAAACTAAGAAATAAAGAAAAGAAACAAAAAC

>UN00192

ACAAATAACACACAATAACATCTCTCTGATAATACAATATATACAACTAAAAGTCAATAGAACAATGTTCTAACATGAATTACATTTTCATTCATTCGCATCTGTATCAGTAATAATACACTGTCGATAGGAAGTCAACAAGGCAAATAGAAGTAACTGAACAATGTTCTAACATGAATTACATTTTCATTCATTCGCATCTGTATCAGTAATAATACACTGTCGATGGAAGTC

>UN00193

AACGAAATAACAAGTACAACGTAGACAAGAAATCTCATCACCGGGGTCACCAAGGGCTTTCGATACAAGATGAGGTTCGTCTACGCTCACTTTCCGTATCAACGCCAGCATCGGCAACTCCAACAGAAACATCGAGATCAGGAACTTTTTGGGCGAGAAGAAGGTAAGGAAGGTAGATATGCTTCAAGGTGTGACAATTACTCGCTCTGAGAAGGTCAAGGATGAGCTTGTTCTTGATGGAAATGACATCGAGCTTGTTTCCCGTTCTGCTGCCCTCATTAATCAGAAATGCCATGTGAAGAACAAGGATATCCGAAAGTTTCTTAGAATAGGTAATTTAATGTCAGCGAGAAGGGAACCATGGCTGAGGAGTAGTGTGTTG

>UN00194

GTGATCTTGATTTGACCAACTACGTTCCCCACAAGCAAGGCCCCGAGCGTCAACTTTATGAGCTATATGCTTTAAGCAACCACTACGGGAGTATGGCAAGTGGACATTATACTGCACACATCAAGCTTTTAGATGAAAACAATGGTACAACTTTGACGATGGCCATGTTTCTCCAATAAATGAAGACGAAGTAAGGTCATCAGCTGCTTATGTTCTCTTCTACAGGAGAGTTAAGGGGAAGGATGAATCTCCTAGTGGACGGCAGCTATATGCAAATCCGAAGCCCAACTTATACCGAAGATAGATTTCACTAACAAAATTAAACAAAAACCCATGTATCATCCACTATAATTTACAGATAAAGAGTTGTAGCTAGAGAAAGCTCGGTGAAGGGCATTAAAAAAGT

>UN00195

AACTTTTTACTATTGTTATAAATTTGATTCCCTTGCATAACCAACCACATTCGCCTATAAACAAATTTCTGACTCATAATTACAATAACACCCCCCACCCCTCTCTCGCAGCATGTTCCTAAAGCCAAAATTAACTTAAGGAAACAGCACTTCAATATTGCTACATCGTAATTTAGATACAGGTTCCTGTACTCCTGTAGAACATCAGCAGTAACAAAAGGGAATACACAGTTGACGTTTACATTATCAGTAAATAAAAAAATTGAACATTAGTTGCAAATTAATGTATTTACATGACTTCCCAAAATCGATACAAGGATATTTCTACAATAGCATACATTCATTTTGCACATGGCATTTTATTCTTCAAGCAGCTGACTTGGGTATTTTGAACTGCTTTTTAAGAAACTGAGAAGCATCAGAATCATTTCTGTGGCACAAAACCCTCAGAAGAGTTTTTGAATCAGCACCAAGTCTGCTCGTGTTTCCTTGGGTGACCTCTTTCAAATCTTCGATCAGGACACGAGTCTGCATAACATCATGTGATCGATTTTGTAGTTAATCAACATAAACTATAAGGTCATCTCAAAGCTGATCAGTTTTATAATGGGGCGTACCCTTGCCACCAAGTTTTCAACGGTCCCTCGAGATTCAAGCGATTGCAGATGATATAACTAGTAAATATGTTCCTCCTCATGTCAACATATTTTATTGTCTAGGGGGGATCACACTTACTTGTTTTTTAGTCCAAGTAGCTACGGGTTTTGCTATGACTTTTTACTATCGTCCAACCGTTACGGAGGCTTTTTCCTCTGTTCAATACATAATGACGGAGGCCAACTTCGGTTGGTTAATACGATCAGTTCATCGATGGTCAGCAAGTATGATGGTTCTAATGATGATTCTGCACGTATTTCGTGTGTATCTTACAGGTGGGTTTAAAAAACCTCGCGAATTAACTTGGGTTACTGGCGTGGTTCTGGGTGTATTGACTGCATCTTTTGGCGTAACTGGTTATTCCTTACCTTGGGACCAAATTGGTTATTGGGCAGTAAAAATCGTGACAGGCGTACCTGAAGCTATTCCTGTAATAGGATCACCTTTGGTAGAATTATTACGCGGAAGTGCTAGTGTGGGCCAATCCACCTTGACCCGTTTTTATAGTTTACACACTTTTGTATTGCCTCTTCTTACTGCCGTATTTATGTTAATGCACTTCCCAATGATACGTAAGCAAGGTATTTCGGGTCCTTTATAGAGTATAGAAAAGGCAGATCATAGATATTTGTAATTTATCATATTGGGGAGGACCAATAGTATTTCATTGCTACAAATATGTATTATTGAAAAAATAAGACATGTTATTTGGATATTTCTCTTCAACTCCAAAGTATTATATTTTTTATTTGATACGAATAGTTGAAGTAGTTGAAGTGGATTCTCCGAAGAGAGGATGGATTATGGGAGTGTGTGACTTGAACTATTGATTGGGCCGTGCCGATATATGATTTTATCCGCCACGTTGGAATTCACAACCAAATGTGTCTCCGCATCCAACCACCATGTAAATCCCCTTATATAGCATAGGATAGGCCGGTTCGCTTGAGGAGAATCTTTTCTATGATCATACCCGAATTATGTCATACATGAACAGGCTCCGTAAGATCCCGTAGAAAAAAATAAGTGATGTGGCATGATCCAATGTTCTATCTATTCCACTTACTTATTTATAGTCTGGAAATGCATTCATTTCCTCTGCATCGATCCCGATCTATGATACTATCGGAGTGAAATAAGGGATCTAAGGAAGAAGAGAGGCTAGACTTTATTAGTAACAAGTAAATACTTTGTATGTAAGAAAATCGAGATGTTGTGGGGGATAAACACCAAGCAAATCAAAAAGACATGAGACAGTCCAAAAAGCACTTGATTATGATCAAATTTGTAAGCCTACTTGGATATTGAGCATTTACCTGTAAGAACTGAATTCTTTACTGAATTCTTTGCAATGAATAGTTGCAACTACGGAAATGGATCTTTTCTTACATAGAGTCATTATATATGTTATGTGTGGATATGTATGAAATATAGATTTTCTATCGATTTATTTCTGTCATCCTTTTAT

>UN00196

GAGAGCCGACTCTGATATATCAACCAAATTAAACATAAATTAGGCCGTTCAGAAAAAACCAGTCTCATTTTCGTAAATAAAGGAAAAAAGGTAGAATAGCCTAGCCTCTTATTTTTACATGAGAAAACCTTTGTTACACAATGTAATATAAATTATACAGAAAATAAAGAAGAAAATAAAATAACACAGTATTGTACCTATGCCTTGCCTACAAAATCAGTTTACACTCCGTGCCACAAGGAACTTGGACCGTGTATAATAATTTGCTTGCGGAGCTATGATCCGGAGAACTTGCTCCTTGTTAATGAGAGTCGCCTTTTTTGCTCACCAATTGTGTAGTTGCGAAGCAATACTCTTCACCATCGGATGGTACTCTGTGTGCATGAACTTTCTTGCTGTCTCTGAACCAGGCTGCCCGAGCCACTCG

>UN00197

TCCTTCTTGTATTAGTTGGACAGAGTAGAAGAGTTTCGTCAGTGAGGAAGAGACCCTCTGATGGTGAGAGCGCGTCATCCAGAGGCAAGAGTTCAAGTGAGTCCTCAAGCAGTGGTCTCTCTGCTACAAATACGATCTTTAATGCCTCAACAAAATTCAAGAAGGGCTAGAAATCGGGGTTTAAGTAGGGATGGCCCGGTCTCAGTTAGAACCCGGAGAGCTTCTGGTGGGCAACAAAGGGAGAATGTTATTTCGGTATCAGACG

>UN00198

GGTTAAGGTCCAGTGGTTGTCTTGGAGAAGTCTAATGTTGTTCCTGAGCATAATCTGTATTTTCAGGTGTACTATAAGTTCAACAATCTTTCACTGTTGAGGGAGCCGATAATGTTAATATGTGGCTTCTTCTTCCTATTTGTTGCTTGTATTTTATACACACGTACAGACATGTCAATCTCCAAATCTTCTGCTTCCTATCTTGCAAAACTTCAGTTAGAGGAGGTGCGAGAAAGGGTCCAGCAAGTCCAGAACATCATCAGCCGATGTTTAGCAGTTCATGATAAAGTAGAGGCATCGTTACGTGAGCTCTCCAGGACGGGGGATATTCAGTCTTGCAAATTGCTCGTAAACGCTGATGGATTACTAAAAGATTGACGAAGGAACTAAAGCCGTTGTTGACA

>UN00199

TTCTTCTCCTTGAAGAAGGAAAATGATACCCATCTTTATACAAGTTGGGTTCTGACAAGGAAAAGAAGAAAGAAAATGCTGATAGGGATGAGAAGGCCAAGAAGTCTTTGAGCATCAACGAGTTTTTAAAGCCAGCTGAAGGCGAGAGATATTATGGTGGTGGCCGTGGCAGAGGCAGAGGAGCCCGTGGATCCTTCCGAGGTGGTTATGGTGGTGGTGGTGGTTCTGCTCTGGCATCTGCACCCTCAATTGAAGAACCCTAGGAACAAGTTACCTACACTTGGAGGGAAATAGAGCATAAGCACTTGTGCCACCGTCATTAGTATTTACATGTTTCTTAGTTAATAATGAGATTCTCTGATTCTGGTCATTTAAATTTTGTTTTCTTTTTCCTATTTTCAAACTACTGTATATTGGAGTTTTTGCTGGGACAATGTTTAAGAGTCTGGTGAGTTCTCTGTTGGACATGATTCTGGGATGTATTGTCA

>UN00200

TTAAAACGAAAACAAAATAAAACTAAGAACTTAACGAATAGACTAATACGGTAGTAGAAGAATAAATAAGAAGGAACTGAGGAACTCTCCTGGGTTAGAGAGCTTAGGATGCTGCTGCTTTTAATTTTGTTTGCTTTTATGCTTTAGAGGTTATTTTGCTGTCTCAATAAGTTGGGAGGTTGACCTCCAGGATACTGGTTTAGGTGTTTATTATCATCTTTGTTCATGTTGAATTTGGAGGTTTTCTGCCATGAAAAAATGGTGGTATGTATGCGTTTAACCGCCTAGATTTTGTTTAATGTGTATTTTTACAGTCTTTGTTGTTTCATATATTACTTCTTGTTCCGATCTTTATCAGTCTATATTGAGGAAGCAGGCGCTCGAACATTGTCTTACTT

>UN00201

TATGCAGCAACAGATGAAGACTGTTGGGGAGCAGCAGCTGGGCTCTCCTTGGCATTACGTCGAACAGCTTGTGAGCGCTCCTGAAACGCAGGAATCGGGCCTGAATCTTTGGACTGGAGGGTGTCCAATGTGTTTTTGGAGCATTCAACATAAGTGTGACCCTGCATATGGCAACCCATACAGAATTTGGGCACTCTCTCATATACAATCGGTTGCTTAAAGCCCTCAAATAAACCAATCCATATGAACTGCGGCCGCTCCTTCCTAAGATCAATTTCAATACACATTCTCGCTTCACTAGGACGAGCAAGCGTAGCCGTGGCTTTGTCCAAACATAGGAAGCGCCCAATTAAGCTAGCAAATGATTCCAAATATTCAGG

>UN00202

GAGAGAAAGAGCTCAAGTTTATTGCTGCTGGTTGGAAGCGTGATGGCTTTGGAAAGTGGTACAAGGATGAAAATGTTGAATTTGACTCCGATGAGGAAGACCCAAATGAGACACTAAAATAGAAGCAACTTCAGCTTAATAATTGATCATAGTACTAGTGGAAGCACTTAGATTAATGGTGGAAAGGCGAATGGAAAGGACTCAATTGAACCTTTTATGGATTTTTTCTGATGTTTATCTGAGCTGGTAAGTGAAGGTTTTTGAAGTTGGGTTA

>UN00203

GTAACCGAACCGACCTACTTACAACCTTACTACCTACCGAACAAGCCGCCGCCGCCACTGAAAACTCCGGCAACAGACAGATTGTTAGGAGATGGCTTTCTGCAGTAAACTCGGTGGCCTCATCAGGCAAAGTGTCTCCGGGAGCAAGATCGCCAGCGGCTCGACTCCTGCGATGCACTTGCTCAACTCTGCCCGCCACATGTCCTCCTCCAAGCTTCTTTGGTGGGAGGTTCTTTCCTACGGTACCGATGACCTGTCAACTTAAAGAGG

>UN00204

AGAGAATTCTGTGAAGCATTTGGAAGAAATGAGAAGGAATTAGCTGATGCTTGGCGTGCTGTTGCATCTGCTGAATCAAGGGCTGCAGTGGCTGAGGCTCGATGCTCAGAACTTGGTGCCAAAATTAGCCTTACTGAGATGCAATAGGTAACTAGGAAGGATGGTGGAGATGAGCATTCAGCACTCCCTGCAAATGAGGTCAATGGAGAGCTGTGGAAAACAAAAGACGAGTTGGAGAAATTGAAACAGGAAGCACGAGCAAACAAGGAATACATGTTACAGTACAAA

>UN00205

ATTGTAGTAATTATTCATCACCACCACCAGTTTAATTTTTATATCAGTTCTTTAGACTCCGTTCATATAAAGTCTAAAAGCTCTTAACAAGTTTTACAGCATCCATGAATAACGAGTGCAAAAGAACTAGTAACCTCCAACAGATACGAGTGCTACCATTCGAGGGCATTCAGTCCATGCTACGATCAGAATAGTAAGGAGAAGGGCTACGTTTCCTTGAAACCGGGTTTTGGCTCACTCGTTTCTTACGTGGAGGAGAATCATAGGGGGATCTCGACCTGG

>UN00206

GGAGGTTTCTTGGGAGCGCGAGGAGGATGTACATCGCGACTACACACACCTGTTTGATCAAGTAAATTAAATTTATGTATTGACTTATTTTATGGCTTATGGTTACATGTATGGCGATGTTTAATACTTAACACCAAAATGAAGGGTATTAACACTTGTACTCCTAAGTACTTGGAACATATGGTAATAATATATGAGGGGTTCCTTGTTTGGATTTTTCGATCGATGTACTTGTTCCATTTAGTTAGGGGTTAAGTGGGGTTATTTACATTTTAGATGGTT

>UN00207

AAATAAGAACAAAAATTAAACAATAGACAACCAAACCTTACGGAACACCTACACCCATCAGCCACTTTATGGCCATTCGAGACATGGGGAATGGATATCGTCGGTCCTATCTTCCCGCCGTCAATCAAAGGCGACCGTTTTATTTTGGCAATCACCGATTACTTTTTCAAATGGGCTGAAGCAGTACCACTAAGAGAAGTGAAGACTAGCGATGTCATCAAGTTTATTAAACACCATATCATCTACCGATATGGAGTTCCAAGACGAATTATACACG

>UN00208

GTCAAAACAACAAAGCATCTCTTAACATTCAAATCAAACCTCAAACCCCAACGATTAAAAGATTTCAAATACAACCAACAATCTACACTCCACAATCCAAATCTGTTCACAAATCTCCTGACACTGATGATTACGTTTGAAACAATCAACAACAATCATATACCAAACCTTAGCACTCTATTGAACACTAGCTACAATCTCAGTTTCAGTGCCAGCTTCCTCTGAACCTGACAGCCCTTGGTAGCTCTCAGTTTTCTCAGCCTTGAACATATCACCATTCTCCAAACTCAACAACTTCTCCTTAGCCTCTCCTTTGAGCAGCTCCACAACCTCAAGCATTGTGGGTCTCTTCTCCGGTTCATTCTGAGAACAAACAAGGGCAACAATCACCATCCTCT

>UN00209

CAAACATCACATATTTAAAAGAGCTCATTCAACCAGCGAACACAACAAACAGGAGGAACCTTATTTACTATTTTTTTTTTTTTGATTTGAAAAAAGGAACCTTGATCACAACTATACTAGATACCTTATGGATAGTTCGAGAAGAAACATCACTAACAAACTAAACATTACAACATCTGAAAACGAACATGAGCAAGGAAAAGAGCTTAATATTACATAAAATACCCAAACCAAATCCGGACCTTCAGAAGTCCTCATCCATCTTGAACTCGAGGATGGTCCCCCGTCCCATTCCCGTTCAAGCTTGACATCACCGACGCCTTCTGGTAATCCCCTAACCCTACTTACTAACGAAAAGAAAAGTTAACGAAAA

>UN00210

TTTCATTTAACTAACTAAAATTGATATTGATGAAATCATATATATGCATATGAGTATGAAACAAAAAATATGTTTTCTAATTATATTCAATTGTTTCATAGTAAAACTTGAAAACTATCAAACCAAAACATATATGTATATATATAACTATATATATACAAAAACCAAAACGTGTATGTGTGTATATGATTGAGCTTAAAGTACTAACACACATAATATAAATTCCTAAACTAAATTACCCAAGCAATCTTGATCTTCAACGAAGCTCTATCTTGGTACACTTGCGGATGAAGCTAAACTTGGATCTTGAACTTGAATTGTACGATGCTTCACTCCACAATTCCACCAACCTCCTCTTGATATGATCGTTATGGCACAATCCATCCATCCTCCAACAACGTT

>UN00211

TTTAACTTATTTGTTCTTAAGTATTCTTTGGGTTTTTCTTCACCGTCATATATTAGTTGAACCTTTTACAGTAGTTATTCATCCTCCCATTCCTTGAAGCAACATCTTGCAAAATGCTCAGAAATGGAGTTATGCCAGATCCTCCCGCAATTAGAATCAAGCTGTCATACCTATGATACGGAAAATTCATAGGACCGTATGGACCTTCTACTGCAACAGCGAAATTTTTCACATCATCAGATGTTGAATCGCCTATTGTA

>UN00212

CAACCCTGAGTAATGCCAATGTGAATCCTCTTTGGAAAAACAGAAAAAGGAAACTTACAATTGCTTTTGGAGGTAAAGGTGAAGCGCTAACATCCGTCCTTTGGCTACTGGAGCTTCAGTAAATTTGCATAATATGGCACAACCTTTATTATATAGAAATTCTGTAGATGCGTGTGTTGGATTGCGATAGTTGGTTCGTTGTTTGTGATGGCTTGTCCTTGGTATACTAATGGTGTGGTTAGGGCATTGTCAGTGTTTGGCTATGCTTTCTTTTGTGCTCCATTTGTGTGGGCATCAGCGGTGAAGGTCGTTACTTCTTTGCATCTTGTTTTTGAAAGGGAGGGTTTTTTTTCCTAGATTGGGAGAAAGCTTACAGACTCCTCAGTTCAGCAAGCTGTACTGATGTTATTTATTA

>UN00213

CGTTGTTTATCCCGGCCATAGATCCGAGGTCTTTTGTTTTGCAGGTGGTTAGCTTTGAGTCATCATGCTTGCGGATGATTTCGTCCACATCTACGAGGTGTTGACTCGTTCGCCTCCAGATCCGGGCGAATCGGTGGAGATCTGGATGGGTTGTTTCACGTCGGCGATGTCTGTTCGCTTCGGGCATCCGAATCTCGCTGATTTCGCTTTGATGGGGGAGATCTGGCCCAGATCTAACCCCGCGTCTCTCCGATCTGTAGAGATTTGACGATCGTGCGCTGTGTAAGCCATTCTGTTGTAAGAAAATTAAATTGAGAGAGGTGCGATGTACTTGTTTTGGGTACCCTGGGGGTCTGGCTTGCTCGAGAGACGCAGTGATCTGGAAGTTGTACTCGGTATGTGTTTCCTTACGACCAGACCTTTGCCATGTTGGGTGCGCTCGCATGGCAGGTCGCCTTCATTAATAAAATTTATTTTTTTGTGTGTTTTGGGGGGAGATCGTACGGGGTGCTTAAAATTTTTCAACCCCGGGTTCTCTCCCCCTATTGCTGGTGCCTGCTTTCTCCTTCTGGTTTTCTGCTGGTTAGATAATCCGTTCCGCTCTGGAGATCTGCCTGCCTGCAGCCAGGTACATAAAACTTGCACAATCCGTTTTCTGTACTTTTTCGTCGATTCACTGGTAAAGTTTGAATCTTGCGAGTGTCTACTAATATGCTAGAATTGAAATTGCGCTGGAGTACTATGTGACGGCCTAGTTTTCGATATATTTATTACATCTCG

>UN00214

CTCCATCAATGGTTGCTTCCACTGATTCCACACGCTATCAGCATGGAAGCCAGTTTACTATGTTCCTAACAGCTCCGGTTCAGGCTTTTTGTCTTTTAGTTGGTATGTCTGGCATGGACATTGACAAGGATACATATGATAAGGCTGAGAAGCTATTTTCATTGTCATTGAGTGAGTGGGAAAAAGCTTTGGTGTCATCTGATTCTCTCCATCCTGTTTGGGTTGAAGTCTTGGCTGATCCTTTTTTGAGGCGACTTGTTCTTAGGTTTATATTTTGTCGAGCAGTTCTCGCACTCTATTCACCGACCTACAACAGAGAAGAATTCCTTCCCTCTTGCTTGCCCCATTTGCCTGCATCTGTTAGCCCAGATGACCCAATCTCTCAGTCTGCAGTCCTGAGACTCGCTGGCTTCTTTGGTGCTGCCGAACATTTTTCCTTCTCTCAAGGAATTTCTGTAGCCGAGGGCACCAATGTTGAAGTATTACCCGCTGCAACAGTCAGTTCTTCAGATACTCCGGAATCTCACTCATTGGAGGATAAGAATCGCTGAGAGACAGAAACTTGCTGCTGTGGACGTGGGCAAATTTGGAAATACTTGTTGTTTTCTGTGAAATCCACAGCATGATATATACATGTAAACCTAATGGTAATGTTATTGTACCTTGCTACTCTAGACTTAGGAGATGAAATTTGTGATGTCAATTGAATTCTTACAAGAAAGAAGTTTATGTGTGTGCGCGCGGGAATTTTGAAATTGATTTACTGAGCCTCTTAGTATGGTTTAGTTATTCTTTAC

>UN00215

AGTATGAAATCTATGATCTGAAACCATTCTTCTCCAGTTCCCATTTCAAGGACAGCAACTTTGTTCTTGACGAGAGCCGCGGTGTGATTAGACATCCAATTTCACAGTGAAAGAGATGGCTGCCTTTATTTATGACGATCTAAGTATTTATCTTCACTTTGAGAAATTTAGGTTCGGCCATCTATGTGTATTATGCTTGATATTTGGGATTGTCCTATCGACAAGTAACTTTGTGTTTTTCGGTGCAAAGTACGATTCTGACTTACTTAGAAC

>UN00216

TTCTACCGTCCGCGTACGCGGGGGCCACCAGCCGGGTGAACGTCGTGCCGGAGTCGATGACGGTGCCCGCCCCGGTCGACGGGTTGAAGGCGAGGCTGCCGGAGAAGTTGGCGGAGCGGAAGCTCGGGAGGCAGTAGGAGAAGGTGGAGGAGTAGAGGGGCTGGGTCTGGGAGAGGAGGGACAGCGGGCCCCGGCCAGGCCGATGAGGCCTTGAGGGGGGACGGAGGTGCCGGTGGAGGAGGTGACGGCGGCGAAGGCGTAGGACCTGATGGCGTCGGAGGCGAGGGAGAGGGTGTCGGTGGCGAGGGTGGCGGCGACGGCGGAGGTGCCGTAGGTGAAGTTGAAGGAGCAGGCGGGGGAGGGGGAGGGGGAGCAGGGAGGGGGAGGGGAGGAGGCGGCACTGGGCAGGCGTCAGCCCCCATACATGGTCTTACGACTTTGCGGAGACCTGTG

>UN00217

CAACAAGGAGATGCTGCCGCACCTAATCGGGTACAGAAGAAGAAGATATGGGAAACTGTCCAGCCTCACCTAACGACCACTGATTCATGTGTGGCTGTTCTTGGGGAACGTCCTATGCGGACTTCGGCAGGAGTGGTGATGAGCCCAACCCTGACGAATGCAAATATCTCTTGATTAGAGGGCTGGTTCACTTATTTTTGCTTATTTTTTGTGCAGTTTTGCTTAGCCAAGCCCCTAAACTTTTATGGAGAACATTTTGTTATATTTTGTTTCATGATTTTTTTTCCAATGTGTTTGAAGTACTTCCTACATCTCTTCTTCGTAGC

>UN00218

GGCTACCATTGAGGAAACCAGGTGTTACAAATATAAATAACAATAATTAACGACAAAAAGCATCAAGTCAACAAAATCACAGATCTAGGGTACCTAACGAGTTCATTTTAAAAAAATTATCCAGTCCTTTGTCAGTTGAAAAAACAAATCCGCGTGAATAATTATACATAACTAGTTAGAAAGACAAGCAGACATTGGATCCTTCATTGCACCGCACGTGAAGGCAGCTATAGCTCAGGTGAGAGATGTGATTTCAATATTTTTCCTCAACCTCCAGGCAGAGGCATGTTAATGTCAGTATTGGAGTTCCTAGGCTCAGATACTGAGTGTAGGGGAGCAACATACGAGCCGTCTCCTTTAAATGAGGAAGAAGGCACAAGGTTTGGCTTTGGTGTTGAACTCTGCTGCCCTGGACCACTATTGCCATAAGCATCCATCTCATTACTGGTTATTAGGGTTGCAGATTCCGGCATTGAAATTGGTGTCATTTCATATCTTGGCTTCTTTGCCTTCTGCTCAATCTGGAAACTTGGCAGAAAACTTCCCACCACAACCTACACAGGACTTGCAGCAACCAAAAGACCAGCAACCCCTCCACCAACGACCCGACCATCAGGGCTTGCTAGTGAAACGCTCATTCCACCTGATCTGCTTCTTTGCCCTCCGTTTTCAATTGGCTCTCTCTCTCTGGTTTCGATCTCTCTCTCTCTCCCCCCTCCACCCCCCTCACCCCCGCCACCTCCGCTTCAGCCTCCCACCGCCACGCCTCCTGACCCCCGCTCCCACCGCCAGCCTGAGCCACCTACGCTCGGACGCTCCCA

>UN00219

TCGGGGAGCTCTAAAAATTGCAACTTGATATCGTTCAAAGCTACCGACCCTTAGGAATAATACTACCTGGTTCTCTTCTTTGATATTTCTCATGCTGTTGTTGAGGAATGACCAGAAGTTTATCGACTAGACTTCTGAATATATGCATTTCGTCTCTCTGCAAGGCTAAGAAATTGGAGAGGGCTGAGGCTGTTATTATTGACAGTATACGACTTGGTTCTCTTCCCGATGTTGTCACATACAATACTCTTAT

>UN00220

GCTCGATTGAGGGAAATTATTCGAGCAGCGGTCGAGAGCGCCGATCACAGGAACAGGAGAATGTGAAGAGGGTGCAGCTTGCTGACAAGTACATGAGCGAGGCTGCACTTGGAGAGGCCAATGATGATGCAATGAAGACTAGAATACGGGCAAGCAGCTCAGCTAAGGTTAGGGTTCCTGTTCCTGAAGGTTGCACTGATCCATCTGC

>UN00221

TCAGGATGAAACCTTCCAGGGATCGGGCTGCTCTGCTGCTCCCTCATGAGACGCAGCCTCAACTCATAGTTCTCTATCTGAAGCTTTGTTAGCTGCTCATCACGCTGAGATATCATCTGCTTCAACTGCTGCAGCTCTTGACTGCATCTCTCAAACTCTTCCTTTTGCCCGTGCAGGGTTTTGAACCCTCGTTTCCAAATATCATTCTCCTGAAGCAGCTTTTCAACTTGTTCCTTCAGCATCACATTTTCCTTTTGAAAGCTTTGAGCTGCTTCAGCACAAGCGCGTGTAACGATTGATTTTTCCAATCCCTCTAAAACCCTAGATGCGCGAGCCCTTGCGTCATCTATATTTGCAGCAGTGGACATCTCTCTTACAATCAGCTCAACCCATTCAGAGCCTGAGCTTGGGAGATTGCAGGCAGCAGATTGGTTCGGGAAAATATTGTTCTCACCCGTATGATTCACACTGCCTTCAGGTGATTGCCAAACGCTGGCTTCAGTCCCAATACTAGCTTTGTTGTTGTTAGCCAAGTCCACTTCTTCCCTTGATGACACCAGGTGAAGATCATTCAAATTCTTTATTGCAATGTCCAAGTCATTGCCCGATACCTCAAGAGCTTTCTCAAGCAACTCTTGATCCATATCAGGGAACAACGACCTAAGGTGATCGATGGCGAAACTACAACCGGCGGTGACGACACCAAGGGATTGTGCGGGGAGAAGTGAAGAGCGGAGGAGCACCGGATCCTCTTCGACGCTGGGCTGGGCGTGTGGCAGTCGTCCTCGAAGAAGCTCGCCGGCGATCTTTTGCCGCAAAGTAGCACAGACATGATGATGATCGAAGCTCAGAGCTTCAACTTGATTTTGGGAGGGGATCGATGGATTCTAGGGTTTGGGGAGATAGAAGGGGCGCAAATGGGGCC

>UN00222

TTAGGTTTCAGATGGCAGAGAACTTGTTCTCAAGTTTCGATCGCCAGGACGATAAGTACTGAACTTGCAAGTAAACACCGGCTGCCTCATTTTCCCCTGAACCTGAAACACCTGCGGACTACACTCAGGCTCTCCGTCGAACTCGAACACAAACCTCGGATCCGGCTCAGATCTCACAGTCAAGTGCAGCATCGCCGTACGATCCCTTGCTCTCCCTCCCAACATTAACCCACCCATTATGAAACACGCAT

>UN00223

AACGTTTTACGTTTCTTAAACTTTAAAGTACTGAGATATCTCATAAACAGAATTCATTACAGTTCTGATTGTATAAAATTATGAAGGAAAAAAAAAAGTCCACTAACTTACAATTTGATATGAACGCGACTTACAATCGGGTTAGCACTTCATGAATTTGCTATGACGAGTCAAAACTTTTCAGGCTCTCAGGCACTGCCTGATCACATTGTCCGAGTACCTTGATCAAAGGTTGAGCCAAAGAGGCATCTGATTTAGAAAAGAGACAATGCACTGCTCCAGCAATACAGTAGCGTGCCATGTTTTTGAGAATTGCAATGTAGACCTCAGTTGACGGGTCTATTTCTTAGAAAAATGACAATTTCAAATTCCT

>UN00224

AGTTTAAACTAATTAAATTAGAAACAATAATTTATATTATTAATTATAATATATTCTGGATTTTTTTTTACAGATGCAGGTCCATTATCCTCCGCATGGATGCATAAAGTTACAAAGCTGGCATTAACACAGCTTTGCCGATTACAAGATCCTTTTCCAGCATCTTTATGCTCTGATTGAAAAGAAAATGGATAGGTTTAGCCTAAGTACATCTTTAAGAACTTCCTCCAGTTCCTTTTGCTGCTATAGAAGTTAATCTTAACCTGATGAGTGGCTTCTTTGGAGCATTAGATGTTGGAGCAGGAAGCCCACTGTAAACCGGTGCAGATAACTCTAAAGGCTCGAGCTCTTCAATGCCTCCATTACC

>UN00225

AAAACTTTAAAATTTTAACCCTACTTAATTTTGTTATTATTATAACTGGAACCAAATTATAAGAAATTCCAAAAGCAGGTCTATACTAGAGAATACTTCACCACGGATAGTTCGATCATACTATACTAGAAACTAAATAACTGCTAACAAACTGCTTACAAAACTATGGAAAATTGGGGATAATTTTAATCAAACAGACTGAAACCCATATCCTCATCATCACTCTCCTTGGTTTCCTCCTTCTTTTCCTCGGCAGCTGGGGCAGCAGCTAGCAGCAGCATCTGCAGCAGCAGGAGCAGCAACTAGCAGCCGGTGCAAGCACCACCACCAGATCCAACATTGAGGATAAGGTCTTCAAT

>UN00226

GTGATCAAGCATTGGCTCAATGATGAACAGTAAACGAGTCTTGACACTCTATGTGATCAAATATAGTTATGTTTCCCTTGAAGAATTCATGGGGCATAGAGGCTGTGGCGAAGATAGTACCTGGTTCATGTACAAATGAGTCTTCTAGTTAAAGTCTCGATGTGTTGGATAAGTCAAACTATGAAAATGATGGCTGTCATGGCTATATAGATAACTTCTGCTTTATACGTCAATTTGCAACATATTGGAGATTAGATTCCACAGAAATTAAGGATAGTTTTGAAATAAGAATTTTTAATGTTGTAGCTTCATTGGGTTGTTGATTACCGGTACTTTTTTGATTCAGATGTAATGAACAAAAGCTTGTCATTTCAGATTTTGATGATCTCAATTCTACTTGAGGTCTTATTTAGG

>UN00227

AACTTTAAGGGTTAAAAGAAAAGAATCTTCCCAATTCATCAAAAATCCATAAATAAGCCCAGGGACACTTCATCCGCTAAATGATAACGGCCCAACATAGGAATCTAAAGGTTTTCAAGACAACTTTGTCTTCCACAGACACACACATAATAACAAACTACAAAATCAACAGATCAACTAGTCGACAAAACACAGACATTCGCCTTATCATCACAAATACAAACA

>UN00228

TTCGTCAGTTGTTTAGTGAATAGCAAGGTTTGTAGAAGTCTTCAGGAGGACAAGCAGACCCTCAGTGAGTTTATCAACGGCAGTCTCTCTCCAATTCAGGAAGCCTTAACAGCTCTCTGGCGTGCAGCTTTTCCTAAAGTTGAACTCAGAGGTTTGATATCTGAACAATGGAAGGAAATGTGATGGCAAGGAAAAGATCTGTCTACAGATTTCAGTTCATCAAGATTTCCCAAGACTTGGTCGAACTAGAGGACGAAAATTGCAACTTCTCCAGCTCGTAATCAGCGCTGTGCACATGCGTATTCTTCTTTTCTTGCTGAGGTTAAGTGTGTGAACTCACGTGAACGAATTCACGTGAAGACCTGGGAACGCGTGGAGAGATAGAATGCTCACACCCGCACTTTAGCAGGCCCACCCGA

>UN00229

TTTTGTTACTTATTGTCTTGTAATTTTGTAGTAACTTCTTCTAAGTGGCTCGGAGTTTCTCAGTAGGCACTATGGTCTTCATCAATCTCTCTATCGTTTTGGTCTATTTCTTTAAAATCTCTTTGTTGCTTTAGAATTTTGTCAGTATTATCTTAATGCGTACTGAGAGAGACTTTTCGTCTGAATTTTATGTCTTAGGGTTGCTAAAACATTTAGATTTTGTAGTTGTTGCTTGTGTTGCTTCTTTGGATATTCATAGTTATTCTATGACAACTTCGCTTATATATTACAGTAGTGGTTATGTGGTATGTAGTGTATTGTGAAGCCATTTCTGTCAATAATCTCGGTCGACAAACAAAACTTAATAGTAGAAACT

>UN00230

CAAAATTTTTTTAACGAAGACAGTGGCAGAATTTACATTTTGCTGTCATACTTGCTGCTTTGGTTCTGTTTTTTCTTAGGCCTCTTCTATTTGTTTGGATTAAGTTAGAAGCTAATTGTCGAAGGGAGAGTGCCATATGCACTTGCTTCAGTGTATTAGCGGTAAGTTGCATGTTATTTTGCTTCAGACAGGCAGTTGATGGTGCCATTTAGCAGTATTCTATCTGTTGTCATATTCGTTTGTTTCTGAAGAAATAATAGAGTTGTCTTAAATTTTTATTTG

>UN00231

CGTAGTAGACATTGCCAATGAAAAAGGCATAGTAGAGCTGAGAAGATACAGTAAAGATCACCCATTTGCGCAGCTATCCGGATCCGATAACATCATATCATTTACCACCATGAGATACAAGGATCAGCCGTTGATTGTTCGCGGACCTGGTGCAGGTGCTGAGGTCACTGCTGGTGGAGTTTTCAGTGATATTTTGAGGCTAGCATCTTATCTGGGCGCACCATCGTAAATAAAGAAATTGCGACTATTATTGTGCTTTGTGAAGCAACTATGATGGGTTTTTGATCTGTTATTTCATTTTATTATCAGAAACTGAAAATGTTGAGAGTACTGTACGAAAGAGCTTTGTATTGCAACTATTTGTTAATATCAACTAGGGCAAATCTGACGTAAGTTCCGAAGGTTTGTTTTTGCCTTTAAAGATGCCCCTTTCTTCT

>UN00232

TTAAGTTAAATTGCGAAATTATAATAGTGTTTTCAATCAATTGTTAGCTTTTGGTTTCTTATATATATCATTATATCTTTTTATGTAATTGTAGATAGGCGCTATGGACGACATAGAAGGGATTGCAAATGAGGGTATAGCTACCGCCATAGGAGATTTTAGTACACGTATCGATATTGCATCGGACTGTGGCGAGGGTTCGTAGAGCTTTTTCATCAAACTCATGAGGATTCGACGTGAAGGTCGTGAGCAGATGG

>UN00233

AGTAGCAGCTAGATCTAAATTTTCAGGAAAAAGTAAAGCTCACCACTTGTAAGCGAAGCTTCACACGCTGGAAGCAATACGGCTCACTAGTAGTATCAAAATCCTCGTAAATGTTTGCAACCAAAACACAGAAAAGGAGTTAACAGGAGAAAGTTAGGAACTACAACTGAACTTGGCAATAGTTTTTCTAGATTGATGACCACGTACATGGAAAAACATCAATATTGATAACCTCGACGACATAGAACATACAAGTTTCAGTCCATGATTGCATTTGAAAGTTCCACCTTTTGAATCATAAAGTTCCACCTTTTGAATTCACTTGGTTCAAGAAAATCCAGGAATTTCTCCAGGCACAGGAGCCAGTTCATTCCTTCCAAACCTCTCTTCATCATAGAAAGTAGCATGAACCACCCTCCCACCAAAGAAA

>UN00234

GGGCAGAATACAGCTTCACTTTATCGTATACCAAAGAGAGTACTTATTCATAGATACAAGATTAGAATACACCTTTTGCAATGTAGAGGGTAAGTCACTCATTATCCCTAAACTTTTAATTAGTCGCACTTAATATCCCTTGGTAGTCAAACAGAGTAGTCTCTCTTTTACCGTCGTCCATCACGTGACGTCAATATGTTGCAGAGTACCCTCTTCTGTTATACACGTTGAATATACGCATACCTTCGCGATGTTTGTACAGATAACAGAGAGATAACTTCCATTTGAAATCGAAGGGTCTAAATACTAGTAAAAAAGTTTAGGAATAGTAAATAACTAATCCCTGTAATTATATATATATAGTTGTGACTCTAGGCCAAGACCTTCCTATTGAAGATCCAACTGAAAGGGATCATCATATTCTCACCATACTCCTTCATCTTCCTCATCCTCCCCTCCTCCGCCCTCTCCTCCATCCTTCTATACCTCTCCGGTAGTCCGCAGACGTAATCTTGCGCCCGCCTCCCCTCGCTTGACAACCCTGCGCTCACCGTCTCCACCTTCCACCTCTTCGCAAAGAACTCCACGAGGTCCGCATAATCCCTCGCCGTATAGACTCCAACCTTCTGAGCCACTGCGGAGTAGTGGTGGAAGAGGTTGTCGTCATGGCCGTCGAACATGAGAAGGGCGGGCATCGTGATGCGTCGCCGCATCATGTCTGCCAAGGCGAGCATGGTCGTGTCGGGGTCGACCTCAAAGAGCTTCTCGACGATCTTGGTGTAGGCTAGCTCGTGGCGCTTCTCGTCAGAGGCGATGAGGCCGCAGATCTTCGCAAGGGTGTCGTCACCGTGGAGCTTGGCGCTGCGGGCAGTATTGCCG

>UN00235

AACTTTTAGTTTTAAACTTTATTTCGTTAGTATTCTAGGTAACTTTTTTGGGTCGAGAAGAAGGTAAGGAAGGTAGATATGCTTCAAGGTGTGACAATTACTCGCTCTGAGAAGGTCAAGGATGAGCTTGTTCTTGATGGAAATGACATCGAGCTTGTTTCCCGTTCTGCTGCCCTCATTAATCAGAAATGCCATGTGAAGAACAAGGATATCCGAAAGTTTCTTGATGGTATTTATGTCAGCGAGAAGGGAACCATGGCTGAGGAGTAGTGTGTTGTATCAGAGGCTGAGTCTTGATGCTTTCTCTAGTCGATCTAGTGTTTTTTTGGTTTACAAGTTATTTGTGGTTGTTATTCTAGTTTTCAGACGTGGGAGTAAAAAGATTTTGAGATATGGTGATGCTGAATATTTTCTAATTCTTATTCCTTTTTCTTTTTGTACTT

>UN00236

AGGTTCAAAATTCATAAATTAATATATAAGCAGGTCACAGTAAAAACATGATATAATCTTTTCGGCAAGATAATATAATCTTTTCTGCAAGAGCGCCACCTAGGACACACGTAGAAGACTAATAATAATATACACTGAAAAGAGAAGATCACAAAGATCAACATCCCCCATTGATTGTTCTACTCACACTTGATACGCACCCTATACATGAGAATTTCAAAATAAACCTCGTACACCTAAAACCTGCTAGAATTGCCTCCAATGCTCCCCCGAACTCTG

>UN00237

GTCTGAAATTCTTTGGTTCAATGGAAAGTTCAATGTGGCACAATTTCCACCTTCTCCTCAAAATCCTCCAAGAAGATAGGATTCTGACAAGGGATTTTGCTGACGTGACAATAAATTCCGGACTCTTTCGCTAATGCATCTTTGCTCAAAACAAACAAAGACATCGCAAGAGTACTTTTTGGGACTTATACTAAATTCGGTTAATATTAATCTCGTAATGTTTTCTGATATATTTG

>UN00238

TGAGCCACGCTGGGTTATCTCCATGGGAAGCTGTGCAAATGGTGGTGGCTATTATCACTACTCGTACTCTGTTGTTCGTGGCTGTGACCGAATTGTTCCGGTTGACATCTACGTGCCCGGATGCCCTCCTACTGCTGAGGCTCTTCTCTACGGCATTTTGCAGCTGCAGAAGAAGATCAACAGGAGGAAGGACTTCCTACATTGGTGGACCAAGTGAAGGGAGAGGGGGGGAAAGGTACATTGGACTTTTTACCGTATCTCTGCTGTGTTTTGCGTTGTGTTGCAATAAAGAGCAAGTGCTCAACATATCGCATGTTACTGAGTTGTTTGGAATGATTTGGGAGAGCGATTCCCCTTTCTGTTATACTATTGGAGGTAGTACTGTTTGGTGTATTCCAGCTTGCTGCTTTTAGTTTGCACCCTTTGTGGGGGTATTGATTTCCTGTTAACAAGAAGAATCTGTATGTGAAAATGACAAGGATTTTCAAATTAATAGAAATAATTTAA

>UN00239

AGAAATAAGAACAAGAAGAATAAGACAATACTAGAACTCCAGAGACAAGAGATCCAGCAAGGACCCCAGATAGAGTCTCAACACCAAAGAGAGCTCCAACAATGATAGGAGTAAGCATAACAAGAGCACCAGGAGGGATCATCTCCTTGATGGAAGCATCAGTGGAGATCTTCACACAGGTAGCATAATCAGGCTTTGCAGTTCCCTCCATGAGACCAGGAATAGTGTTGAACTGCCTTCTGACTTCCTCCACCATCTTGAGAGCTGCACTGCCGACGCTCTTCATTGTCATGGCTGAGAACCTAGTTAAGGTAAGTACTAATTAAGTTACTAACCCTAACTAAA

>UN00240

GTAAGTTAAAGGAAAAGAAAGACGTTACTACAATACAAGAAGAAAAAACGAAGTAATTACAACAACCTGCTTGTCTCCGATTTTTCCGATACAACACCATCACTTCCTCCTGAGGAGCTACAACAGTTTATGGCCGGTCCACAAATGGTGCATGAGATGCACGCTGACGATATAGGAGCTGCCGCTGGAAGAGTGCCTCCCCCTCGTGAGGTTGAGGGGCGAAATGCAGCCATAGTTTTCTTGGAATCTCTTCTTCCTTGGATCC

>UN00241

ATTCTCTTCATAGTACACAATGGATCCAAATTTGTAACTCTCTCTCTTGCTCTCTTCACGAAAAAGATCCTATACCCCTCTCTCAAGCAAACATCATTAAAAACAAAAAATTTCCAAAAGCCCAATCCAAATAAAAAAAACAAAGAACTATGTACAAGACTGTTGTATATAGGAAATTATGGATACGAGCTGAGATGAGTTCATTTAAAGTTTGTTAAGAGATAAAAATAACCAGGATTATGAAATATGAATCTCAACAATTCATCCTCAAG

>UN00242

CCAATGCTGGTTCATTTCTCTTGGCAGACGGTGACTTTTCCTTCTCCTCATAGAAGTCAAAATCATCTAGTATTGATGTTCCCGATTGGTAAGCCTTAAAAATCTGAAGCATTTTTAGACCTTGAGGAAGTTTTATCTCTTGTGTGTCCCTGCTGAAAGTTACAGCTCTATTGTCATTGTTTTCAAGAGTGATATGCTGAAAATTCCTGTTTGGAATGTCCTTTATTATGTGCCATTTTACTGGAAAAAAGCCATTCCTATTTATCTTGCTGCCAGAAGTCCATGGTCTTCTTGAAATCAACCGGCCCAGTCATCTCCGCCAAGCCTACGAACTGACCACTTGCG

>UN00243

AGTAAAGAAGATGTTCTTTCAGAGATTGCTGCCCAAAAAAAAGTTTAGCAATATACTCATCCAATATCACACCTCACATACTGCACCTTGGACTTGTTTGGCCCAAACATGAGCCTCAATTTATTCTCATTACATAACATAAGCGACAATATTGCACAATATAAACTATGCACGTAACTATCTACTAACAAAAGAAGTTAATATGTGATGAAGCCTGGTTGCTCTCATCCTCATCCCGTGGCGGAGAATGTGCATGTCGCCCTTCGTACGTAGTGATCACCATACGCGCATCCTCAGCCAGCCGCTCCACCCTCTTCTTCACCCTGCAGTTGTCCTGTGTGCACCTATAGTAGCTCCTAGGATGTTGGGTGTTCTTCACAACTTTTTGTCCATACTTCCTCCACTTGTATCCGTCATCCAACACATCAACTTCACTCATGGTCTTGAAGCAAAACCTTGGCTCCCTCACCTTTCTTCTTGCCTTCATCACCTTCTTCATCCTCTTGGCTCCAACTGATGACAAATTCTCGTTATAGCTTCTATTGTTGATGGTACTAATATTGCTGCTCTTGTTGCTAATATTGTGAGCATCCTCCCTGGTTGATGATGACAAAGGATTGATAGT

>UN00244

CTTATAACCTGCAACAGAACTAACATGGCTAGGGATAGAACTAACCTTGTAAGAACCACTTCCAGTTAACGTTATTTTCCTGATCGCATATACAGTACGCCAATGGAAATATCCCCTGATTTCCATCATATGCTACGGTGCTTAGGAGTATGCCTTTGAATCGATCTTTAAGAAAGGTACCATCCAAAAACAAGAGTTGTCTACAGCCGTATAAAAAACCATTTATGCAGGCGGCGTAGCATATGAATAACCTTGTAAAAC

>UN00245

TGAGTCGCACACTGGTTGGAGCTGAATCTCGTTATCCAATGATCGAGAAAGAATGCCTCGCCTTGGTATTCGCAATCCAAAAAATACGACATTAGTTGGTTGGACAGACAATTTATGTCATTTCTAACATAAACCCAATCCGGGTATTCATGACCCAACCAGCCTCCATGAATTGGCGTTTAGCCAGGTGGGCTCTACTATTATCGCAATATGATCTGTACTTCAAGCAACAAAAATCAGTTAAGGGTACGAGCTATTTGCGATCTTTTGGCGAAAAACCCTACGAAAGATGTAGTGTAGTTGTACGACGATCTGCCGAATGAAACTCACGAAGTTAATATCACCTCACATCAGAACGTAGTAA

>UN00246

GAAATAAAAACAAAAATAAAAGAAAAACAAAAGAAAAGAAGAATTAAACTGAAGCCACATAAGCTATTATTGTTGGTTATGCAAGTATTCTAGATTTCAAGTAATACCATACATAAACAATGTCACAACACAAGGAAGCCTTTGAGCACAAATAACCCAATCCATCACCAAACCCCACAAGTCTCAATTGATCACCACAGTTTAAAGAAGGGGGCAGCTTATTCCGCCTTGGTGAAGTGCCTGACGGCGAAGGCCAAGCCCAATATCATGATAGGCACAAGGAATTGCAATATCTTGATGATGAAATCAGAAGATTTGTCAGGGTTGTATGATGGTTGCTGAGGTGGCACATAAGATCTCTTCGCTGGAACAGATGAGGCATCGATGTCTCCGATCAAGTACTTGTCCATCATACTACCCTTAAGAACTTAAAGTAAAAGTTTAAAAGTAAAAC

>UN00247

AGTAACAATCAAACTTTTCAATGTAATTAAGAAAACTTATCTGATAATCTGTTACAATAAATGGCACAACTATTTTTCTAAAGTATGAACCAGCTGCAGGTACCCACCATGCTACATAGACTGATGGATAACAAAATACAATGATTAACTTCTGAACTACATACTATAAGCATCAGGATGTCCACCGTCAAGAATGTCATTGATTGAAATATCTCTAATTATTTTTTCTGATGAGAGAAGGACCAGCTTGCAGCATTTTCTCCTCAGATAGTGTAGTAGTCACAGCAATAGATCTCATTTGTGCAGCTTTGGCCGCCTGAACTCCAGCAAGAGCATCTTCAATCACTAGGCACTCGCTAGGATGTACATCCAACTTCTTTGATGCAGCTAAAAAATATCTGGAGCTAGGTTTTTAGGTTTCTT

>UN00248

TAGCATCATGGTTTCTGGTTACAAGATGGCCCTCCAGAGGAAATGATTCACTTATGCCATAGAAAGATCGAATACTGTTGATAGTTGCTTCATCTTTGAAAAATAAGACAGGATCAACTCCTTTCCACTTGCCTTGTGTCTGTAATCTCCCTTTACTTCCATTCAATTACTGCATGGCCTTTCTCCCCATTGTCACATTCTTCCTTTTCTTTTTTCCTTATTATCTTCTGACCCAAGATCTTTAGATCCTAAATGCTGCTAAATTGGTTCTCGTCCATTTTACTGTCATCAGATGCTTGTTCTGTCGTAACAGGTTCTTGATCATGAGGCACATCTCCAGATAGCTTTGTTCCCGACTGATCTTCGTCCACAAGGTTCTTGCTCATGTTTCTTGAAATCTTGA

>UN00249

GTTTTCGAAACTCTGAGCAATTCGATGGAGTCGAGATCCACTCCGCCGTCACGGCCACCGAACCCCGCGCTTCCCTACCGTGAAGATTGCTGGAGCGAGGGAGAGACCTCCGTCCTCATCGACGACCTGGGGCGATCGCTACGTCGAGCTCAACCGCGGGAATCTCCGTCAAAAACAATGGCAGGAGGTCGCCGACGCCGTCAACTCGCGGCCCTGCGACGCTCGCCGGCCGCCGAGGACCGATGTCCAGTGCAAG

>UN00250

TTTAACCTAAACGTTAAGGAAGACGGAATACAACGAAGGGAAGAAGGTACAACGGTTCCACCGTCAACGTCGGCATCAACCCCTCCAAGGTTGTGATCACGAAGCTGAAGCTGGACAAGGACCGGAAGGCGCTGCTCGACCGCAAGGCCAAGGGGAGGAATGCCGACAAGGCGAAGGGGAAGTTCTCGGCCGAGGAGGTCGCCGCCGCCGCCGCTGGAGGAGCCGCTTCGCTCCAGGAGATCGATTGAAGAAAAAGCTGAGTCTTTTATCTAGTTATTGAATTTGAGGGGTTTTGTTCCGTACTTTTTAGGTGTTTAATGGCGATGATACTGGTAGCACTCGCCGATGAATGTTTTTTCAAGACTTGAGAGAGTTTGGTGAAGGTACTTTTTTGAATCTAGTTTGTGTTAATTAATAGAAGTAAGTAGTAGAAAGAAACCCCAAAATTTTT

>UN00251

AACTTAAAACTAGTCAAAATTATATTTGAGATCTTGCTTATTCTGGACACAAGTTACATACTCCTCTTTACATTCTTAAGAAAACCATCGAAAAAAAGAAAAAAGTTTTAATTCAGGGAACGCAGCTTATTTAACTGCAGGCATGTCTGAGCTCGACAACTCTTATCCGATATTAATAGAAAATTGTGGATCAACAAGAACGCTCCAGTGGGCGATTAACACTCCATCAAGGGGGAAATTAACACTGAATTATTTTAGTTGACGCCGTAGATCCATTTCTCAGCATTGCTGGTATAAGAGACCAAGTCAGTAGGCTCGAACCATAAGGAAATTTCATCCTTGGCGGTTTCAGGCCCATCGCTTCCGTGAATGATGTTTCTTCCAACAACAACAGCTAGGTCTCCACGAATAGTTCCAGGCTCAGATTTCTGGGGATCAGTGGCACCAATAAGTTTTCGACCGTATTTTATAACACCTTCCCCTTCCCACACCATAGCAAGCACAGGACCAGAGCTCAGAAAGTCGCAAAGGCCGTTAAAGAAAGGCCTTTCCTTGAGATCATGGTAGTGTTTCTGAGCAAATTCCTTGGAAGGAACTACCAA

>UN00252

TTAACGAAAAAGGAAATAACTACGGAACGAACTACAACCAAAGCACGAAAAGCCAGGACTCTTTCAGAAAATGGATTCCTATTTGAAGAGTGCATAACCGCATGGATAAGCTCACACTAACCCGTCAATTTTGGATCCAATTCGGTTTTTTCCTTGGGAGGTATCGGGAAGGAATTGGAATGTAACCCTAATATCGATTCATACAGAAGAAAAGGTTCTCTATTGATTCAAACGCTCTACCTATGGGATAGGGATAGAGGAAGAGGAAAAAACCGAAGATTTCACATATTTTGATCGAAAAATCAATCTGATTTATTTCGTACCCCTCGCTCGATGAGAAAATGGGTC

>UN00253

TCTCAAAAAATATCTCCACAAATTCGCTCGAGATAGGAGACATGATAATTTTTCCAGATATTCGCTTTACAAAAGATCTGCTTGTGTCATTGAGGAGTTAGTCTAAAAACTAAGGGAGATTTGCTGCGTATTCCTTGCTATGAACTCATGATTGTAGTGTTGCAGGAAGTACTCGCCAGTCTTATTTTTTCAGTTGGGTCTCAGATCTGGTACCTTGTATAAGCCTATCCAATTGTTGCTAGGAAAAGCTTGTGAATTTCTCTCTCTGCTATGTATGATAATATTACAGTACAATGCAATTGCATTGTTTTATCTATCTATGCGGGGATCAAATCTGGCAATAGAACTGGGAATGTAACTTAATAAAGGGGACGTACAATAACTAAGAAATAA

>UN00254

GGTACGAAAAGATGAAAATGGCACTCTTACCCTGCAAAAGCCCACTTTTGTGGTCATTCGTGCAAAATATAGTTTGCTTTGAGAAGTATCAGCTAATGGGCCAGCCTCGAGAATTAAAACAAAGGACCTTCCATTTCCACCGACAGAGAGAAGCAAGCCATCATATCTGTCTAGAGTGGAACCTAATGGAAGGGAAAGCTTCTTTGAGAGTTCAACATAGCCACCTCTGGTATAAACATACCCTGAGAATACAGCTTC

>UN00255

CGAAAAAGTTTAACAAGAACCTTTAGTAGAAGACTGAAGTGGAGATTTATCACTTGAACTACTTTACTTACAGGACTTCGAAGGCATTAATCTAAACAAATTTTAGATTCCAAGTAATCTTAGTGGATCCAAGTTTCATATCCTTTGGAAAACAACGAGGACTCTCAAGATCGTTTCACCCAGCATTTCTGTTCCATACAAATGGCAACCAATGAGAACCTTCCCCCTAATGTCATTAAACAGCTTGCTAAGGAATTGAAAAATCTTGATGATTCCCCACCAGAAGGCATTAAAGTGGTTGTTAATGATGATGATTTCTCAATTATATATGCTGATATTGAAGGGCCTGCTGGAACTCCATATGAGAATGGTACATTCCGGATGAAACTGTTGTTATCACGTGACTTTCCCCAATCGCCTCCAAGAGGTTATTTTTTGACTAAAATCTTTCATCCGAACATTGCAACAAATGGTGAGA

>UN00256

GTCTAGAACCTCTTAGGTATTACAAAATCTTACTATGGACTCATATTCATACATGAACCAAGACATTCATCTTCTTCTTATACATGGCCAAAAATTTTTCGAGTAGTCATAATACACGTCGAAGAATTATATTATTTATTTTTACAAGATACCGAGCTTGGCTAGCAGTACAGAGTTTGATTTCATATATAAAACCTAAGATGCAAAAGAAATCACATAAAACACGAGAATTTACCTATCTACATACAAGTACAAGCATCAGTATAATTCCACATTCCATTGGTCTCCCTTCTTCAACTCCTTCTTGTAAGTCATCCAGTCAATAACGGCTATAGCCGCCAAGTTCACCCTAATGTCGTCGATAACCTTCTCCATTACTTTCAATCCAACACATATACACGTAGGTGTAGACTTTCTTCAGGAGGTCCCACAAATACTCTGCGTAACTGTGCCATTCGAGTTTGGATGTACCATTTTCTCGCCCTTCTCATAGGATTGCACTCGGGTAACGGCGAAAATCTAGCCTGAATATGTCGGGTGCCTTCTCCTTCATCTTCTCAAATTCCTCCTTGTAGAGCAATGAGCTACTTGTGGGAACTCCCAAGAAGAGCCATGCCAATCCATTGAACTTGTAATCATCATGCTTCTCAAAGAACATATTCCATACGAAACTACGGAAAGGAG

>UN00257

CCAAAACTCACCTATTTATTTCATGTAAACAGCTACAGAAAATTAAATATAATCTCCAGTTACGCTCCATGACCACTAAAACAATATTCCCGCGTGAAAGTAAAGGGCAGTGAGCGCCAGTTAGAACTTTAGACTGCTGCGGTATGGAAAAGTTTTTAAGTAGCTAAAGTACAGGTACAAATGTAAAGTTCAACTAGTATACAACAGGCTGGCGAGTTCAAATCAATTCTGTCTGCAAAGGCCAACATAAGCAGCTTTCTTGAACAGGGGATGAACATTATCAACAAACACCTCCTTAACAAGCATTTCAATATGCGGCCAAACCTCAGTCGCATACTGCCTTGAAAAAGCCTTGACGTAACCCAATGTGGTTAAACACCATTGCTCTTGCTCCCCAATGTTGTAGCCATTCGTTGTAGTTTCACCATTCATCTGACTGGTGTTTTTGCCATTCTCATGGTCAACATCCATTGCTGGCGTCTCCTCGCTGTCCACAACCATGCTTTGCACATTACCAGCCTTTAAGTTTGGTAATTTCCCGTCAGCAGATACAGGAGGCAGTCTTTCCTTGAGCACATCTCCAAAGTTCTTGTACACTGATAAAAACAGTGCCTTGTTTTCTTCAAGAGCTCGAGCAATGAGAGCCTCCTTAGCTTCCAAGGCTTCCCGTAAGGATATCTCTTCATCTTTTGCTTTTGCAGCATACCCTTTAAGCCGTTTTAACCTCCCTAAATTATTAGTATGCACTGGCTGACCATCAACTAGTTCCAGCATCGCTTCAGCAGCCTCAAGCTCTTTCTGAGCTTTGATTGTAGCTTCCTCAGCTAATGAAACACTCTTGTCAAGTGACACAATCTCCTTTCTGAGGTCAGTGATACGATTGTACGTCTTGTTGATCGCATTTCTAAGGATCTCCCATGGTCGATCCGAAAGGTGAAATAGTTCAATATTAGATGGAGAAAAGACCCAAGTGA

>UN00258

AAGTTAAGTTTCTTCGTCATAAAAGAAAGGTAGCGAAGATATATTGCACAAGGGACTTCCAGTAACTTTAATATACATACATTGCTGCTAAACCTTATTAGACCAATACAACAGATCTACGTAGTCATAGAAATTTGACGAAGCACACTAGCTAATTACATACACCATCCAACTTACATGACCATGACCATCTAATTTAGGTGACCATGTCAAATGTACCCATTCACTGAAATCAAGGCTTGTAAATTAAACCTCGATATCATATAGTATGTGCCTGAGAAGCACAGGGATCACAGTATCAGGAGAACTCAGCTGCGGTAAATGTCCATTCGAAGGCATAACCTCAACAATGGACTCACCTCCAAGATTCTTATGCCAATACTCAGACACCACGACTGGCACCGCGAGGTCTTTGGTGCTTTGGAGGATGTGGCAAGGGACAGTGACGAGTCCTAGGATTGTTCTTAAATCACTGGAGAAAATCGTTTGTAGCACGCTTAGGGCGATGTCTGGTCGGATGTTGAAGAGAGTACGA

>UN00259

GACAAGAAATTAATAGTAGTTTAGTAGGGTGTTACTTTCTTGCACTGTCATTTTTCAGATAATAATTGTCCAGTTCCTTGGTGAATTCGCAAGCACTACGCCATTGACATTGTTACAGTGGTTCATCTGTGTGTTTATTGGCTTTCTTGGCATGCCTATTGCTGCTGTTGTGAAGATGATTACCGTTGGATCTAATTGAAGAAGATTTAACGTAGGAGAGTAGAACCGATATTTATGAGATAGATATCGAGCATTCAGTTCTCTACTTCAGTTTACTTTTCTGCTGTCATGAGTCAAAGCATGCGCAGCAATTGTTCACTGAATATGGTTGTATTTATGTATTTTCCTTTGATTATTTTCCATCTGTATTGGCTTGATTGGTTTTGTAATGTAGAAGTTATATGCCGGTAGATTATATTTGTAATAATTAAGATTTAAGTTAACGACTGAGTACTTTATTTGTAATAATT

>UN00260

CTAAATAACAAAAAGACCGACAATAAAAACTTTAATTAAACGAGCCAATTTCCCTAACTACTTTGATTCGAACCACATGGTTGATGCGGCAACTACACAATTCTCGGTTACCAACCATAAACATACATCAAACCATGAAGTACCATCAAACAACTTCATATGAGTCCTGCATTCAAAGCCCCAGGCACTATCCATATCAGTTCTACATTTTTTTAACAAGCCTTCACTAGATTCTGACATGTTGACATGGAGGTGCCAAAACCATACT

>UN00261

TTACTCGAATGCTTGTTTGGTTTCAGAAGGTCCGGTCAAGTTTTCTTCTCTAACTTCTGACATTAAAACAGGAGCACGAATTTCTCTCTTTGATGAAGGAACAGTGCTGGTATTTGAACTTCTAGCTAACATAGAGATTGAATGACAGGTGCATCTTCACCAGGACCAAGCGACCGTCCATCTGGAGTTCCCAATCTCCTCAGTGTACTAATAAATGTTTGACTTCACATATTGCTCACGGAATTCTTTATCGTTATT

>UN00262

CCTTTTATTTTGTTTTTTCTTACTTAAAAAAGGTCTTGTCTATTAGTATTTCAATTTCAGAGCTACATGAGTAAAAAAGAACACAAATGTCAAAAAGCCTCTCTCTTCAACTCACATATCTCTCGAGGAATTGACTCGAACAAAAGTTTCAAATTTCATGGTGCAACGACAATACGACCAGCAACTTGATCAAGATCCCGCCTTCCCTGGGAAGTGATTATCCTTCCACCTCTTGGATCAACTTCAATGATATTCATTGCCTGCAACTGTTGCAAG

>UN00263

ACCGTCTTCAATCTCCAGGTCCACGCTGGTCACGGATCCTCTACGGTTCTTCAATCTGGAACCGTGTTGCCTATCAAGGTGCATAAGAATGAGGAGACAGGGCAGATTCTGAATCTGGTGATGGCATGGGCAGATGAGGGTAAGGACATGACGGTGTTGGTTCCGGTGGTTTATAAAGGGGAGAAGGCTTGCCCCGGGCTCAAGAAAGCAGGGGGTCATCTGCAAAAGATAAGGACCACTCTGAAATACCTATGCCCAACTGAACACATCCCACAGAAGATCGAAATAGATTTGACAAATCTAGATATTGATGATCGAGTTCTTTACAAGATGTCAAGGTTCACGAATCTTGAAGC

>UN00264

AAACTTTAGTTAATTCCGTCTTATTCCCTTTGTATAATTACTCAAGATAAAGTCCACAATCTCTTTTGAATCGTATAACACAAGAATTTATAGGATAAATAAAAACAACGATTTCAAACACATGATGTTGATGCTGCATACCACAAAGGCCTATAAGAATAAAAATAGATTTTCTTCTGCGCGTAAATGATGAAGAATGCCTCAAGCTCTTCCATTCCATTCTTCATAGAATTCTGCCA

>UN00265

CGAGAAAGTATTGAACTACATAGATCTTGGAAAGAGAGAAGGGGCAACATTGCTAACAGGAGGAAAGCGTTGCGGAGAAAAAGGATACTACATTGAGCCTACAATCTTTACCGATGTCAAGGAGGAGATGAGGATAGCGCAGGATGAAATATTTGGCCCTGTTATGTCTCTCATGAAGTTCAAGACAATTGAGGAAGGCAAATCGAAGAAAGCCAACAACACAAGGTACGGATTAGCTGCAGGCGT

>UN00266

GTCCACGTCCAACCTCCCGAGCCTCCAACCTTAGCCCGACCGCGCCGACAACAACGCCGCCCGTCGAGCCTCGTCTCCTCCTCACCGCTATCGTCGCCAGACAACCCCTCGCCGGAGTTTCTTTCGACGCCACGCCCTTCCGTGTCCGATCCAACCCGCACCGACAGCGTCGATTCGTTCGACGAACCCCGAGACTTAGGAGAAGAACATGTCCAGCTTGGAGATCGTCAAGAGCCAGATGATTCTTAGTAGGAGTGCCTA

>UN00267

GTTAAATTGTAAAGTAAGGTTAAAGATTAGTCTCTTGAGATGCAATGTCGTTAATCAAATGATGTATATACAACACTTAACGGACCTACATACAACCTTACAAGTCTCTAAACAATTTCCATGACCATATTCCGGTAGATACGACAAGAAAGAGCCACATCAATAACATTGAGCTGAGAAAGCCCTAAGTTTGAGTTATGACAGTTCCGTCCATCCCAGTCTCTCGTAGTCTTGCTCGCTCCATCTTTCTTATACGCCATTCCTCAAGAGCTCTTTGGCTTTTGTCATCGTTCATCGCCCTATCTTTTAATCTTTTAGAAAGATCAACTGCAGCTGTCTGCTGCTTCGGCGCTTGTTTTATCTCGGGCCCTGGTTCACTATAGACTTCTTGCCCAATTTCTTTAATTCTTTTGATTAATTCAACGGGCTCCATAGCACGCCGTATTTCCTCTGCCTCCAAGAAATATCTACCTTGTGACGGATCGGATCTCGGAGATCGGCCGCCGTTTCTTCGATCGGATCCATGGAGAAGGAAGAAGACGGCAACGAGATTCAAGCAGCAGATGAGTATGGTGGCGTTCTTGTAGGATACCTTCGATCTCCAAGATTGGGGAAATTCCATTAGAAATCAAAAAATTTGCCCCAGATTTGGAAACCCTAGATTTAGCGCGAAGACTAAA

>UN00268

ACTTCTGAAGCAAGTTGTCAACCAAACTACACAAAGACTCTGCTTTTAACTCAGCAAGTGATCTTAGAGGTTATTCACCATAGGAGTTAATGCTTTGTGCGACATAAAGGTATAAACAAAGAATAAATACCATCTTCTGTTTTCAATCGTCATGAAGGATGTTGACCTGGAGAGCAGACTGACATAATCTTGTTCGATCAATTTTTCTTGAAAGCGTCAAAGCACTTGTTGCACGTACTTGATCGGAATTACATCTTAGATGTGCTTCTGGCTGACATACATTGCT

>UN00269

TCTAGAAATTTTCCTTTTTCTTTGCCCCCCCCCCCACCCATTTCCTTTTTCACAGTTAGATAATACAACAAAATCTCTACAAATCATAACAATGGGAATATAATTCCTCACACATGTTTTTGTTCTTGTATGCATACAACAAAAGCCCAAGCAGCGCTAATCTCTACCTCTGCGTTATAACGAAGGACTGATTAGTGATCTCCCGTATGCCATAGGCAGGGTCACTAGCTTCTTGGTTTTGCTTACATACGCCCTTTTCGTTGAAGTTGTCGTAACCGTTTGCTTCAATCTCGTCAAGAATTTCCCGATACAAGTACAGAGAAGCATAAACCGGCCATTTACTAGCCTGACTGAGCTCGCCCACTCCTTTCTCCGCTTCATGAAAGTACATCCTTGCCCTTGTTAAATTTAA

>UN00270

AAAAGTTTCCTTTTCTTTCCTTACTTTGTTCTAGTCTATTGTAACTATTTCCTAGCTGTGGCGCTGAGTGCACGAATGTTTTCCTGAGTGTCGAAGAAAACCCATTTCCTGGAGCTGGGTTAGCTGTGTCGCATACAACTCTTCTGGTGGAACATTGGGAGGGATTGGGGCACACCGAGACCACCAGCACCAAGTCCCCCGAACATGTTCATCAAGAAATCCATGTTTGGCATCCCT

>UN00271

GGCGCCGTACTACCAAGAAGCCATACTCAAGGTCGATTGTTGGCGCTACTCTTGAGGTGAATTCAGAAAGAGGAGAACTGAGAAGGCTGAGGTCCGTGAATGCTGCCAGAGAAGCAGCTTTACGTGAAATCAAGGAGCGAATCAAGAAGACAAAGGACGAGAAGAAAGCGAAGAAGGCTGAGCTTGTGGCTAAGACTCAGAAGACGCAATCCAAAGGGTGCCAGCATGCCCAAGGGAGGCAAAGGCCCTAAACTTGGTGGCGGCGGTGGAAAGCGCTGAAGAGATTTGGCCCATTTTCCTTCTTTTCATTTCTGCCATTTTGTAGTTCATTAACGCTTTTAAGTTCGATGGATCTCTTCTGCATTCAGAGAACATTTTCTAGTAATTTTGTTCAGTGTACTGTCATCATACTGTTTAATAACTTAGAATTAGTTTTAGGTTAGTTAGAAGGTAAG

>UN00272

AAACTTTTAGTTTAAACTTAGGTTTTCTTAAGTTAGTATTTCTAGAGTTGAGAAAACAAATACTAGCCTTAACATAGCACCACGGCCTACGTAAGATCCCAAAACGATAAGATGTGGGCATTGCCACTCTACTCATTACAACCTCGATAAAACAAACTAAGCGACAGAATAAAGGAAATCTAACTCTCAGTCTGCTCCCTCAGCCTCCTGATGGTGCTCCTGACAGTGACAGCACTCGCGGTGTTGAGTGTGTAAGCACCACCTAGTCTTTTCTACTTTTGCCACAGTCCTTGCAGCCCCAGATCCCAACAGC

>UN00273

CTCGTGCCTAACACGGATGAGAAAGATGCCGAGGCTTTAAGTCAAGAAGGGATACAACAGGATAAAGATTCAAGTTGAGCAGAAAAACTATTTATGGGCCTTCATCTCAAAGAAGCATTTCATCTTGGAGCTCATGTAGCCATGGTGGAATCATCCAAAGAAATGTTAAATTATCATGAAAAGGGAAAGATGTCAGTTGTTCACCTATACTAACCCTTTTATCTGGCTGGTGGGGGGGTTAGGACTACACAAGGTATACGCGTCATTGTAAT

>UN00274

AAAACTTACCTACTAATAAATAACTGCACCCAGAATAGATGTGGCAAACTAGACACCAAGATCAGCTGTTAAAATAAAGTACAAGTCTTACGAGGAGACCTAACTCTCCCAAAAACAAATGTTCATTAAGTATCTTTATAGCTCATGAAAGTGGGCTTTCGATGGCGCGCCCTTCAATCTTCAGGGGAAAGGGTCAAAAGTTCAAGAGGGCATGTATGTTGAAGTGTCAATTTGCTCAGGCAACTCTTTGATGTCAACCTCAAACCTTGCTTGAACCTTATTAAGAATATCAACATCAGAAGCAGATGAGACAAATGTGATGGCCAACCCTTTAGTCCCGAAACGAACCAAGACAACGACCCAAACCCTAAGTAAGT

>UN00275

GTATTTTACAACACTGTCACCAGGAACAGGCAAAATTTTGAAACCAAACTCAAAAACAAACAAACAAACAAAATTGATCCATGACTACATTTCTGATATTCATATCAGAATATTTCAAGCATGCACATTGCAGTCACAAAAATTAGTAATGAATAAACCTCTAAAAACTTACAGTTATACTTTCCTTGCTAGCGTCCTCTGCTTGGTTGCATGCTGCACAACCTTTTCCTCCACCGGAAGCCCTTTCCTTCGTCTGACAGCATTCATGAGCTTCCGAGCCATATTTGGAAGG

>UN00276

ACAGAAACGGACGGGAAACCCCAACTCATTTATTCATTTAGTATAGCACACATACACGGTGACATTTTCTATTATAGTCTTAAATGTCACATTACTTCATCGTGTATTTAAACCGTTTATAAGTACAATCACAGACGACTCGAAACCGAGCAAACGCACAATACTCATACAAAAACTGCAGCAGATACTAGGTCGTTGCAACTTAGTTTATACAGAAACTACTGATTACGCCCAGTTCTCCACCACAGGGCTATTATTAGCTCA

>UN00277

TTTTCGTTTCCCCTTTTGTTATTTATTTTTGTTTCCCCTTCTATTTGGTATTGAGGATCAACCCTCTTCCTAAATTCTCCGTCCATGTCTCTCTCTTCCTCCTCCTGTCCTCCCCCAGGCCCTAACTCCGCCGCCTCCTCCTCCGACGCCGCCGCGCAGGTCCGCCTCCGGGAGGCGGAGGAGAGGCTGCGCGAGGCGATCCAGGAGCTCCAGCGCCACCACTCCCGGCGAGGATCCGGGCGATCCACCCCGGCTGCGACCACGCCGACGAGTCCTGCATCGCCCACG

>UN00278

GTTCACCAGAAGTTATACTCGGACTTGGGTGGAACTATTCTTGTGATTTATGGAGTGTGGGTTGCATCCTTGTCGAGCTGTGTTCGGGAGAGGCACTCTTTCAAACTCATGAGAATTTGGAACACTTGGCTATGATGGAGAGGGTGTTAAGGCCCATTGCCGCAGCACATGATGCACAGAGCAGACCGCCGAGCTGAGAAGTATTTCCGGAGAGGTGTACGGGTTGGATTGGCCTGAAGGGGCTACTTCTAGGGAAAGCATGAGAGCAGTTTGGAAATTGCCTCGTCTTCAGAACCTTGTGATGCAACATGTTGATCACTCTGCTGGGGATCTAATTGATCTGCTACAAGGGCTTTTGCGGTATGAACCAG

>UN00279

CAATTAAAACAACTAGAATAATTAGTTTTACAAACCCATAATGATTCAGGTCCTAATAGTTACAAGGAATGAACTCACATCCATCCCAACAAAATGTAAGCATTTCTCAAGCACAAGATTTGAGTTTAAAAGATTCACATTCCTATGAAGCCCACTCCCCTATTTCCTACGATAACCAAAGGGGGCCAAAGAGATGGGAGTATAGCAAAAACTCCCATGTAAATCATCCTCACCGCTTTCCCTACTTTAGTCTTCATCCTCATCGTCGTCCTCCTCACTCCCGGTTCCTTCTTCCTCGTCATCGTTTAACCTTCGGTACTTTGGTACTTTGTTCTTAGTTACTTTACC

>UN00280

TCCTGAACAATCTTGCTCACAGTTTACTTTATTTGGACAATTCTATTTAGTTCTGATGGAGGATACAACTATGTGGTGGATACAACTATGTGGTTAACAACTAAAGTAACTCAGATTCTATCCAAAGTTGAAGTCCTTGAAATCTCATTCATTGTCAATAATTGGATGAGAACATGTTATTTAGTAGTCTTTAATCATAATCAGCGATTTCCATATAAATATTGAAAATATTGATAAC

>UN00281

CTTTCTTATTGTACTTGTATTGTAGTCGTACTCATAAGCTGATGAGTCGACTGAGCAGAAGCTTTTGACAAAACGGCTGGGTTTCGTGAGTCGACTCATGACAGGCATGAGTCGACTCAACCTGATGAGTCGACTCATGTACATGTATGAGTCGACTCACGAGCTGAAATCTGGATTCTTCGAATCTGCTGCTCGCCGTCTCGTTTCACGCTTCCACCTGTGCTCCGCCG

>UN00282

GTTCAACAAAGTAGCTTAACCTACAAATTGGAAAAAACAGTATCTCAAATAGTCGAAACAAAAAAGCACCAAACAGCCAAGCATAGAAATCTAGCGATATCGGCGAAGAGATTTGGTCTGGTTCCTCTTCCAGGTTGCCCTGCGAGATGGCCTGTTCTTGTGGTACAAGTGGCCTTTGCCTCTGAGACCACGGTACTTCTTTCCTGCCGAAGTAAGCCCACGAAGCTCGGGACGGTGCTTGTGCACACCCTTACATATCCAGTTGATCCTAGGATTCGTTGTCGGTATTGGTCGTTTGTTGAGCTGCATACAAAAC

>UN00283

CGAAACGAAATAACTTTACTACTCTCATCATCAGCATAATCCAAAACCCTTAACCCCTAATTTCTTCTTCCTCTATCGAGGAAAGAGAAAAAGGGAGGGAGATGAGCGAGAGAGGAGAAGGGGAGCTGGTTCTCGTAGCTCGAAAGGGTGGTTTCGGGCTGCCGACAGCTTGCCCTAATTGCCTCCCAGTCTATCTCTACCTCAGATTCGCCAACGCTTCGTTCAGTCTCGATTTCGATGTCGCCAACCCCGATTCTGATCATGT

>UN00284

CTCCGCCCCTTCCTCTTCTCCCTCTTTCTTCTCCCTAATAAAAAGGGGGGAAAAAGGGAAAAAGAGAAATTAGAGAAAATTAAAAGAGAAATAGTGAGGGATGAAGACGAGGTCAAAATCAAGAGAGTCCAAGGCAAGCGACAACTCCAGTGCTCTCGCTATCATCAGCGGCAACAGCAACAAGCAGCCGAAGAAGCCCAGGCCCAGGAAGAGACCCACTGCTGCTGATGACCCGAAGAAACCCAAGAAGCCTCCCACTGCTTTCTTCTACTATATGGAAGATTTCAGGAAGATATATCAAGCAGAACATCCAGAGGTGAAATCAATGCGTGAGATTGGGAAGGCATGTGGTGACAAGTGGAAAATCATGAATTTCGAGGAAAAGGTCCAGTACTATGATATAGCTACAGAGAAAAGAGCAGGATATGAGAAGGCCCTGGCAGCCTACAATAAGAGAAAGGAATCTGGGGAGCTAACAGAGGAATCTGATGACGAGTACAAGTAATTTGGATAGATGAAGAGATGCTAACGAGAAATTAAACAATGTGATTTGAAGGAACTCTCATGTATATTAGAACCCTGACAATTTTCTCTCCTAATGAATGACCGATGATTGTTATTTATCTTGTTACAATGGCTTGTTTTATTGAGACCTATCTACCTTAACT

>UN00285

TTTTACTAAAATAGTAAATTTAATTTACTAAGAAGGACTAGAATTAATTTGGCTGGTCCCCTTGATTAAAAATATTAAGATCAGTAGTCAAATTTCTAAAGGACAGCTTCATTTGACCAGTTCTACAGTTGATGAGAATATTAAAAGTGGCTAAGAAATGTTGTCCTAAGATGACAGGTGTCTGTCCTTTGGGATTGGAAACAGGCTGTGTTTCCAAGACTACAAAATCTACTGGAAAAATAAAATCTCCAACCTTAATCAGAACGTCCTCTAACTCATCCATAGGGACCTTGACAGATCTATCAGCCAACTGTAAGGTGCATTTGGTTGGGTTCAGTACACCTAGACCCAATTGCTGATAGACTGAATATGGAAGCAGATTGACACTGGCCCCTAAGTCTAAAAACGCCTTATCAATGTGAGTATTA

>UN00286

GTCACGTGGGTGGGGGCCGAACGTCCGCTCACATTTTCTGCATTTGAACTTCACTAGATTCGAACCGAAGATATTCAGTTTCGTTGAGCAGGGAAGAGGAAGCCGGAGCCCGGCCCTCTTTCTCTTCCTAACATTCTCAGCCCAGTTGTGCAATGTTGATCTCAGCTGATCGGCAAATATTGCTTGTTTAAACGTGTCCCCCATCTGGGTGACAATAGCATAAAGAGGAAGAGTGCTGTAACTGCAGAGAACCTGAACAATCACACCGATAACAAGTCTCGGTATGACAAATCCTACTTTATCCATGATGCAGGATTTGAAGCCATAGGTGCTCCATATCCAGA

>UN00287

TTTGCAAAGCAATAGCTCATTACATTCGAGAAGTCCTTTTAGAGCATACAAAACAACTGGAGTGCCGGAAAGGCGTTTAATACACAGTCTATTTCAGCGAATGCCTATATGAGTACTTTGATTCACTCCCGAAGAATTTGAGTTCAAATGCGTAAACGGGCACTGTCACTAAAATTATATTCTATGAAGGATTAGCCTGTGGCTTTAAACTGAAGACATAAATAGCAAAGAAAACAGAAGAGATGTCTTACGTGATGCTTAAATGCTGTCACTTTGATATGAACTTCATCAGAATGATTTGTTTATATGATTTTAATTAAAGGTCGTGGATAGATTTTGGAATGCTTGAAACCTGAGTTTGCGTCAGTGTACTTGATCCAGGCTAGAGGGTTCAAGGGTATAACCTATTTATGAGTATATTTTGTAGTTCTCAAAATGTTGGCAAATATTAAAAGTTCGAAGTGTTTGTACG

>UN00288

TCTTGGGTAGGGTCCGGAGGAACTATGAGAAGCACTCGAGCATGCAGCATTACTTGCTCATCTTCATGGCTGCTGCTCAACGTTCCCCCTCTTGTTCTGGATTGGGAATTAATCTCGGCCCTTGAATCCTCCTTGTGGTTCTTCTCCATTTCTAGCCAATTTCTCAATGAAATGACATTTTTTTTATTCTCTAGGATTCTTATGGAGTCCTTTTTTTTTCGCTCCAACAAATCATTTGGAGCTCGATTTACTTTTATATTTGTTCTGTTTAGCTCTCCAAAGGAGAAATGATTATCAGTATCATTGTTTTCTTTGTAATTAAAAAAGAACTATTTTTGTCGATCTACTTAGATAGTTTATAATTCTTAAGGCTAG

>UN00289

AGTAAATTTAATTTAGGACTCAAGGGGGTAAAGATTGAACTTGGTGGTAAAAGACAATTTTTTTTATTTTGGAAAAAAGAGAAGTTAATTTCCCTTCTATAAACGGAACAGATTTAAAACTTTGGTAGCAAGGAAAGTTTGCAATTGTAGCAAAGCAGGTCGCGGAAATTAACCAGATCACAATTTGAAATTGCAAACTAAAAACAGTAAAAAAAAAGAGAGAGCCCAATTTCAAATTATACCACAGTAAAAAACATATATAAATTGAGAAGGAAAACACAATTAACAGGGTATGAGGTCGCGTGGCAAAGGAAACCTGGGGATTTCAAACAGCAATAAAAATTAAACGATTGAGATGGCAAAAAGAAAC

>UN00290

GGATTCCCGTAACAATGGCGAAGTCTATGTATTCGACATCATAGCACGAAGTCTAAGGGAGTTCATTCCCTTAGCACAACGTTACATGCGGAACGGATTCCGATAATCCGCATGCTAGCCCGACTCAAACGGTAGTCGGTAAAGGCTAATCAAACCCGTTGAAGGCTGGGGGTTAGCCAAAAATCCTAAAGTCAACACCTAACACGTGTCGCCGGGTAGCCTATTCCCGTAACAATGGCGAAGTCTATGTATTCGACATCATAGCACGAAGTCTAAGGGAGTTCATTCCCTTAGCACAACGTTACATGCGGAACGGATTCCGATAATCCGCATGCTAGCCCGACTCAAACGGTAGTCGGTAAAGGCTAATCAAACCCGTTGAAGGCTGGGGGTTTGCCAAAAAATCCTATGAGTATTATGGTCAATCATTTCAAACGTGATGAGGAATCATAATAAAGATAAAGATAAAAACCAATTCCACTAAAGAGAATCAAATGTCAAGGTTTTCAGCAAATAAAATCATACATCAAGCCCATGGCTAAGAATCTGCTGACTGAATTCAGATAACTTCTCGTCATAAGAGCGTCTAAGCTCCTCCTGCAACTCAGCTCTAACAGACTCTTCGTTCGCAGCTAAAGTCCTCACAGCATCCTCCTCTGTCTCAGCCAGTCTCTGCTTCAAGGACTTCAGCTCATAAGATAAAGCAGCATGTGCTTGCTGATTCTGCATCTTTGTGTCTTCTTTCTCACGGAGTTCTCGTCGGAGCTGATCTATCTGAGAATCAAGCTCCTCGAGGAAAGAAGATCTAGTGGCGAAATCAGCCTCAACTCGATCTATGCGGTCCCGCTGCACCTGAATCTCGAGCTCAGCAACCATCCGAGTACTGCGCCATGTCTGTAAGCGAGCATCCAACTCGGCAGCAACGAATGAAGAGGCACCTGTATCCCGGAG

>UN00291

GAACCTTACCCCAAAACTAAGTACGAATAGTAATTAACAATACAATAACTGGTTGGCATGGCAGTTTGAGATCGCCCCTATAATCCTATAATTAAGTATTAAGAATCGTCTAGGCTACAAGTGGCAATTGTACATATATTACTATTACCAATTTGGTATTTTCTGAACGGAGCCTGGATATTTTATTAGTCCAAGTCAACCATAAATTCTTCTAATTGATAATATTGATCCTCAACAGAAATTGATCTAATTTCACTTTACGCCCCGAATGATTGAAGAACCAATCAATACAATATTTCTTGGGCGAAACAGAGGATATCTCGATACGGGGGAAAGAAAAAACGGGGAAAAAAT

>UN00292

GGTTACAATAAGAGGAATTTCTATTGGTCTACTCATCATATGCATTTGCTTTTAAAGTTTTAACTGTTTGCATAACTGATTAGGTGATCTATATTCTATATTAACATATCTTTTTTTACTTCACAGTTGACCTTTTCACTTTCTAGATATGAAGCTCTTATTGATGCTTACTTGGTTGATCCATATTTTTGAAGTTCTGTCTGTTTGCATTACTGATTTGTTGATCTATATTCTATACTAACATAACTTTTTT

>UN00293

GTTCTTACTTCTAAAGGTAGTACGAGGAGACTGTCAAATGGGCAAAGTCGAGGGTCAGTTCACCGGAAGAGATTGAAACATTGGATGATGATGATGAAGTTGCACAGCCTGATTCTAACCTTCGTTCCCATCTTAACCTCGCACTTCTCGATGTTGAAGATGATTCAACCTCGGTCAGTAGCACAGAGCAAACCCTGGATTTCGTATCAAGTAACACTTCCTTGGATGATTACTGCAAGGGAGATGGAGTCGGT

>UN00294

ATGATTTACGACGTGAATTCCCCCCTCTTCCGCTCGTTCCTCAGAACCAAAGAAAAGGGACGGGACCCTACCGAACAAAGAAGGAAAACTGAAGAAAAGGCCAAAGGAGCAGAAGCCAAAAGCAAGTGAGAACAAACCCGTGATGAATGAATGAGACTATTTTGAGAAATGCTTGAGTAAGGCTTTGAATTTCCCGCAGACTTGCGAGTAATCTGTTTGGGTGATGAACTATAGATTTGTTAACCGTTTGCTGTAACTTCTGTTAACGCTTTATGCTGTTGTTTTAGTTGAAACCAATGGACCACCCTATGATGCTTTAGGGCATTGTTTCAGCGCTTTAATGTCGTCTGTTAGCTATAAGAATAATTTAGTTAAAAAAAAGGGTTAAAATTACTAAACC

>UN00295

GTTTGGTCGGTTGTATGAGGATGATGAGGACAGCGATTAGAGGGGACCGAGAGGGCAAAAGAGGTAATTTTGGTTAAAAGATTTAGCGGTAATTTGTATTGAATTAGGGGGTGTAATTGGTGCTTCTGGATGAAATGTAGATGAATGCTGAGTGAGTAGGCTTGAATGCATCTGTGCATCTTCTTTGTTTTCAATTGCCTAGTATTTTGTGTGAAGTATTGGCGGAAGTGTTGAATGCTACTTTTGATGATAGACTTGGTTGTTATTTGGAATTAGCTTTCGGATTTC

>UN00296

TTCGTTACGGTCGGAGATCGTGGGGGCCAACATCAATTTTTCAAGTCGGGGAGCCTAGCCGCAAACGGCGTCGATAGTCATCGGCTCAACAGAAGGAAGTGGAGAGTCGACGCGGAGCTAGGGTTTTTCTTTGACGCATTTTGTCGAACACTTGGTGGGTTTGTACGAGGGAGTGCGAGATATCGGTTTCAGATCGTGATTTGTGCTCTGCTTTCCACGGGACTTCCACTTGGCGAGGGAAACATGATCAGGACATCATGTGCCACTATGTTGAGTCATCTTGGGAAATGCGGTCCAGTACAAGGAGGTAAAAAAGTAGGTGAAAGGTCTCTACTTATAGTGCTGACACTATGATATCTTCTA

>UN00297

AAGTAACTACACCTTTTTTATTATTAAGCCTCCAATTTACGGTCTCCTTTTGATTCCAACAACTCAAAACATGTCTCTGTGACATTAGCACAACTTAGCAGTTAAATGCAACTGTAACAAATAAGGAGCTCTGATAATAATAATAATAATAATAATAATCTAAGGAGCCACTGGTGGGCCCAAGAGCCATGTTTCTCCTCCAACATAATACGTTCCGAGGAAAGGTTGGGCTTCCTCATCGGTCAGAAGATGAGCCCAATGGACCCTTCCCGTCCAATTTGCACCCGGCCCACTACATTGATACTCACCATAATACACGCCACTCTCCTCAGGCAGTTGGATATGCCAATTGCTCCATCCTCCGGAGATGACAACCTTCTCCATGAAAGTGTAGGAGAACACTACCCTCGAGTGATCTCCCCATGCTCTCCCCAAATAGACTAAGCCACTTCCATTAACGGTGCTACTTTAGTAGAAAGGAAGAAATACCAAA

>UN00298

TCGTTAACTTCCTTTGATCCCTGACCCCTTCAATCGGGCCGTAATTGGGCCGGCCCAGGCCCACCACATGGCTGCAGAGGAGAAGGCAATCGCCGAGAAGGGGTTCTACCTGCATCCGTCGCCGAGGAGTGCGGAGCCGCCGAGGCTGCTGCCTCTGTTTCCGGTGACGTCGCCGAGGAGCCAGAGCTCTTCGCCTTAAAGTTGACTGCGCTCTGCGTCAGATCTCTGTGAGAGGTTTATATCTGAATTGTTAGATTGTGATGTCGATTACTGTGTTGTTCTGATTGGAGCCAAGCGCATTCATCGTTGTCAGTGTGTTTTAAAACATCAATGCTAATTTTGGTGTATAATGCATCCACTGTGGTTATTGTTGAAAACGTTTTCACTCATTGGGTGAGTGCCCTGTTTCTTTAGTATAGCATCAGAAGCTACTAGAAAGACCCAACTTTAGAAA

>UN00299

GGTTCTTACCGTAAAAGTAAGGTCTTTTATTTGGGATATTTGGGTTTCCATAACATCAATTTCGTTGATCAACGAGCATACATAGCAACACCTGCCTAAAAATGAATAAGGCGGTTATCACAGAAAATAGCAGTAAGATACAGCACACGGACATCACTGGGACGCTACATAGATGCTACAGTGGTACCTAACAAATATTTCCTAATATAAGAACAAACATATTAAGCTAGAAAGTGGACAAAAAGCCATCCAAAAGGTTCATGAGCTTAAATAAATCCTGCATAACGATAAAAACAAGATTTGCTCTCCAGGCTATCTACCCGGAGGCAAAATTAGAGCAACAATAACAAAATAAGGAACCAAAAACCCAAATAACAAAAATAAAAAACCAAAAAACCCGTAAAACAAAAATAAAACTAAAACTAAAAC

>UN00300

ACAGAGTGGATTACCTAGATCCTTTTGGGCCGAGACTTTAGTTTATGCGAGTCATCTCGTCAATAGGTTTGTAGGCTGTCTAAAAATCACAATAGATAGCTGATCCCCATCAAAACACTGATGATGGAAGTTACAATCTGACGACTTCCAGATGGCTACAATTTGAAGAACTTTCACGTAGATCGCTAAAGGTTACAATCTGACGACCTTCACGACAACTGATGGAAGCTCTAATCAAACAGCCTTCATAATCAACATCAGGAGTTACAATCTGACGACTCTCATATATCAGAGAGCTAAAGTAAAGATTACAAAATGGCGATCTTTGCAACGCTTCGACAGAAAGAGACTACAAATCGACGGTCTCTTCAAATCAAGACG

>UN00301

CGTTCAAGCATCAAGCATGAGATTCTTGATTCTCGATCTCGCCATTGTAGAGATCTGAGCTCTTCGATCGCTTGAGGTAATTGCAGTTATAAATCATGGCTACGGGAAACCTTTTCTCGAAAACTACGCAAGCATTGTTCTATAATTACAAGCAACTCCCTATACAGCGGATGCTTGATTTTGATTTCCTTTGTGGAAGGGAAACGCCTTCTGTAGCTGGAATCATTAATCCTGGCTCTGAAGGGTTTCAAAAACTATTCTTTGGTCAGCAGGAAATTGCCATCCCAGTTCATCCAAGCATTGAAGCAGCTTGTACTGCACATCCAACTGCTGATGTTTTTATCAACTTTGCATCGTTTAGAAGTGCAGCTGCCTCCTCCTTGTCTGCTCTAAAACAACCCACAATTCGGGTTGTAGCTATTATAGCTGAAGGTGTGCCAGAAGCCGACACAAAGCAGCTCATTTCCTATGCACGAGCTAACAACAAGGTTGATTAAGGTCCTGCAACTGTTGGAGGCATTCAGGCAGGAGCTTTTAAGATTGGAGACACTGCAGGAACTATT

>UN00302

TTCTTAAAACTTAAAGTTCGTTGGTAGTCTTGCAGCACTTGCAAGGTCTCAAAACCTATGAAGGAGTCCCACCTCCATATGACAAAGTGAAGAGGATGGTCATTCCTGATGCTCTCAAGGTTCTGAGGCTTCAGGCTGGTCACAAGTACTGCCTTTTGGGTAATCTATCCAAGGAGGTTGGATGGAACCACTATGATACCATCAAGGAATTGGAGGAGAAGAGAAAGCAGAGGGCACAAGTTGCCTATGAGAGGAGGAAGCAGCTGGCCAAGCTTCGTGTGAAGGCAGAAAGGTTGCAGAAGAAAGCTCGGTCCTCAGCTTGAGATTCT

>UN00303

AAATCAAAACCGTTACCGAGACTTTGCTTCAACAATGGCCTCTGCTGCAGTCACTGTCTGCAGTACTACTAGTTCCCTCCTCAAAGTCAGCTTCTCTCGTTCTCCCCGCAAGAACCTCCATCTCTTCGACTGACAGGATCACTTTCAACAAGACAAACTTGTACTCAAAAAATGTGTCAACAAATGCAAAGTCAGTCTCTATAAGAGCCCAGGTCACCACTGAGGCTCCTGCTAAAGCTGTAAAAATCTCAAAAG

>UN00304

GTAAAAGGGCTGTGGTGGCCCTTTATTTTCCCTTTTTTGTTCAGATATTTGTATTTGAGACTTGTTGAGTATTCCATTTTATCTACACAGACCTTGACTATGTACTTCAGTATGTTGGATGATGAGATATTTATGTGATTTCATTTCATCAGTATTTCTTGTGTTATCTTTCACTTGCTTTTGTACCTACACCTGTTGTTGATTCTACTAGATTAATGTTAATTGTGATGGTGAAACATGTTCTTGCATGGTGTGG

>UN00305

AAATCAAAATGGTTCTCTCATTACCATTGTCGTCGATAAAACTGTGTCACGTCTCCCTCGAAAGAAATAACACTAATCAGTTTCAACATTGCTTGTGCACGTAAGTAAGAAACTTCAACCAGATGCAAATAATTGGTGCTTACTCCAAAATTTTAAGCAACGATACATAACAATGAGTTAGAACTTAGAAGGCAGAGCCTCCACCTTTGGTCTTTCCAAGCATAATGTCCTTGGCTTCGTTAATCTTCGAAGCTAAGTAATCGCTGCCTCCTGCATCTGGATGATTAGCCACCATCACCTTCTTGTGTGCTTCTTTAACTTTATCGGCAGGGACGCTCTCCCGAACACCGAGAATGAGAGCAGCTTCTCTTCTCGTTATAGTTACTTTGAAATTCCTTCCTT

>UN00306

GCAGAAAAATGAGGCGTACCTATTGTGGCTATTATAATTGTTCTAACAAAACCAAACATAGATCTCAACCGACACAAATTGAAACTAAAGACGGGTGGTTGAGATCTAAACCCTTTAAATTTGGAAACAGATGCTCATGTAAACTTTTGACCATTTAACAAAAATTAATAATAATTGAAAAGAGAAGGAAGAGACGTTGCTGGCGTCGCCTTTGTTCTTTTTTTAAGTCCGCTTGGTTCCTAGCTGTCAATTAGGTTGTGAAGTGAGCATCTC

>UN00307

CATTAATCAGCAAGGACCAATTGTAAGTATGAAGCTTAGTATTGCGGCGACAAATATTATAATGGCTGAGGTGGTGTTTAAGTTAGTCATTATACAGTGTACGAGGAGAAACTCTCAGCAGGGCGCTGATTGAAGAGAATGTTTTGGAGTTTGGTGGGTTTGAGCTTATTATTAATTCAAGGTGAACGTGCCTGCACTTTTGCTTAAGCAAGCATGGTTATTTTCAAGGTATGAAGGGTTTTGCAAGTTATTATTATGGTGTAATTGACCTGGCAGTCTGTCATCATTGCTTGTGTTGTGAGCTTCAGACGATTGACAATTTCAGTGTATTCAATATGATATATTTTCCTCAAACAAAAAAAGGGGGAAGAAAAGATAGAAATTGAATTACGAGATAAATGAATTTTTAAGG

>UN00308

ATATTAACCAAATGTTTACTGTACTCACAAATTTGAATTGCCATGTGGTTAGCTTATCTGCAGCTGATGTCACCTTCTCAAATTTCTTCACATACTTGCATTATGTCCTATAAGGCTATAACAATGCACGTGTATGTATTATGTGCGCCCAAGTAGCTCAAATTTATCTCCGACGGGTCTTGTTCATCGTCTAGTAACAGATTATGCAGGTTTTTGACCCTCCAATTGGAGGAGTCAAACGGTCGCAGTTTGGCTGAGAATTGATATGGAGAATCCTATTACCTAA

>UN00309

GTTTGGTATTCTTCTAGTCTACTTTACTAAGGGGTAAGGGTTCCTTTTTGTATTGTTGAATGTTGCATCTCAATGCGGGTTGACTAACTCTAACTATACAGAGCTGAGCCAACTGTATGAGAAATAACAAGGATCAAGGTTTCGAGATTTTGGCTTTCCCTTGCAACCAGTTTGGAGCACAGGAACCAGGTAGTAACGAGGAGATTGTGGAATTTGCTTGCACACGCTTCAAGGCTGAGTATCCCATCTTTGACAAGGTTGATGTGAACGGGGACAAAGCTGCACCAATCTACAAGTTCTTGAAGTCCAGC

>UN00310

AGGTAAGAATCACTAACTATTACAGGGACTCATCTTCATGATGAAGATACAAGCCAAAACCAAAGCGAGCACAGACTCGACAAAAGGCTAGTTCTTCTGCTGCTGCAACAGGATCCTCGAACCGAATATCATTTAAAGACACTGTACCTGTAGACTCTCTGTGTGCCTCTCCATCAGATCCTCGTATTGTAAGACGATAAAGCACTGTCACACTTCCATTGTCTGTGTACATAACACTCCTTATCTCCCCACACCAACCGGGGGCGTAGAAGCTCAGCATTCTGTTAGCATGGTACCAAGGGATGGACTTTTCCTGGTGATTGATGATGTTATCAGGGACTCTCTTGTTGAGATCCCTGAGAATCTCAGCCAAAGGGCGAGTGAGGCCAGAGGTGGCCTTGTCCAAAGGAACCACGTACTTTGAATTATTAGGAACCTCCTTTTTCCCTTCCGTTGCTAGA

>UN00311

AAGTAATTAATCTTCCTTCCTGTGTTGGCCTACTTTTCTTGGAACTAGCGATTAAAAGTAGTAAACAAAGGAGTGATCTTTTAATAATATATTGCTCCGAGCTCAAACTATAGAAAACACAAAATATGAAACGTAAGCAAAGTAGAACACAGCTAAACCATGGACTTTCCTTTCACGACAAGGAAAGAAATCTATCTAATTGCACCTAATGAACCGGAGGCGAATCCAGGATTTCGTAGCATCACATAACAAGCTCCATAGGTTCCAACGGTGGACCGATGCCCTGTTAACACTGGACACTGTATATATAGATGCGCC

>UN00312

ATCTCGTCTCCCCTTAAATTGGCGTTTTTTTGGTTTACCATTTTTATTCACTTTTTAAAAAAAAATTAGGGAAGCCATGGGAGTGGAATCGAACTCCAGCGAAACTCGGCAAGCAGTTCCCACGGCGACGACCGCCGACCAAATCCAAGAGCTGCTCGAGGCAGCTAGGTATGGTGACATTGACGATGTCATAAGCCTATTCTCCATGGGTGTTTCTCTTGATTCTAAAGATTCCCAAGGCCGAACAGCACTTCATATGGCTTCTGCTAATGGGCACCTTGACATTGTGCAATATCTCATCCAG

>UN00313

CGAAGTTTGAAAGCAAAAGCGGAGAGTGTTCGATACTCATAAAAACATGGTGGTGGTCGGAGACGTGATTGTGACCAAGATCATCGAGAAGTTGGTGGAGATCGGTTTCAACTATGCAGCAGACCGGTATTTTGCTCGGGATGCTAGGATGAGAGTTGAGCTTGAAAGGCTCAATGATGCTCTTCCTCGCATCCAAGCTGTTGTGGAGATGGCTGAGAGCGGGCAGCAAGAGATCAATGGAGGGCTCAAAGAATGGCTGTGGCAACTAAAGGATGCGTTGGATGAGGCTGACAACGTACTTGATGAGCTTGATTACCTTAAGCTACAGAAACAAGTTGAGGGGAAAGCCTCAGGCAGCAAAGAACATGGTCATAGTGGTAAGTGATGGCTTTTTAAATGT

>UN00314

TAATTTACCTAAACCCCTTACAAAACCCCGAACTACCTACTTTTTACTCCTCTCTCATGCTCGTGTCAAAGTCCCGACTCGATCTCGATCTCGATCTCGATCTCAAAGCTCGATCTCGATCTCGATCTCGATCCACCCGCGTTTCTTTCCAAGCTCCTCACGCTCATCCCTACTCTCCTCCGCCGAAACCCTAGCTCCGGTCCTCCGCCGAAACCCTAGCTCCGGTCCTCCGCCGAAGCCCTAGCCCCTGAAGATCATCTGTCGGCTAGCACGAGGAGGAGGCCGAAGCCCTGAGCAGCAGAAGTTCTTTCCAACTCAGAAGTAGAGGCATTGG

>UN00315

AAACCTTTGTTCTTTTTTAAAATTTTTCTTAAAATTTCCCTATTGAACAAGCAAATTCATCATCCTGCTTCGCAACTCCAAACCCTAACTTCAAATAACTCGTACAATAAGGCAGACAAACATAAAACATATTCAAGGAGTACTCTTTAAACCTAAAAGTAACAAAACAAGGACAAAATGGCATCACAGCCGAAATACAAACTAAATCAGCAATAGATAACGATGTGTCTTCTAGGCGGCGGTCTGCTTCTTGAGTTTTCCAAGCTCTTGCCTGACAGTCTAGTCTTTCCCCTTTCTTTTGTTATTTTCTTTTGGCCAACATGACCTTGAACCTGTCAAAGTCGTTACAAAAGGAATAGCTACTTACTACTTA

>UN00316

TAAAATTGTTACCTACAAGCGAGGCCTGTGGGCGATCAAGGCCAAAAAACGGCGGGTCTTTCCCCAAGCACGACAAGAGCCCGACCCGCTAGCCCGCCGAGAAGCCGCCCAAGTTTTACCCCGCTGACGACGTGAAGACTCCCATCCCCAACCGCCGCAAGCCCAAGCCCACCAAGCTCAGGCCGAGTATTACGCCCGGGACGGTGTTGATCTTGCTCGCTGGGAGGTTTATGGGGGAAGAGGGTTGTGTTCTTGAAGCAGCTGCCTTCAGGATTGCTTCTCGTCACTGGGCCCTTCAAGGTTAACGGAGTTCCCCTCCGCCGTGTAAACCAGTCCTATGTCATTGGGACATCTACCAAGGTTGATATTTCTGGTGTTAATGTTGACAAGTTTGATGACAAGTACTTTGCCAAGGAATCCAAGAATAAGGTAGAAGAAAGAACCGAAGGGAAGTAAAGTTTAACTTTTAAGTAAA

>UN00317

TATTCGGGCGATGATGGCGATAAAGGATGTGGATGGGATGTTCTCGACCTTGGATCCGGAGTACTATGACATTCTCATGAACTTTAGGTTTGGCAGACCAACCAACTTGTTTCACATGACGTTGAGGTGGATTCTACAGGACAGGTTGCTACTGCTGATGATTGGGTGGAGGAGGAGGATCAGCTTGCTGGAGTTGATGGTGATCAGCATAGTCTTCAATGTAACTATGCATGTACTTTTGTTTTGAGCTCTCTTGTTTGGATATTTGCTCAGGTCTAGTTGCATACTTTATCCTTCTTCTTGTGTGGCTGTATATTTTTTCCCTATTTTTTCTGGTTGTTTGTTCATTTCTTTTTATTTAAAAAAGGAAAAGAAAACCACTTGTACCTTCCATGTGCCTACATGTTTTGAGATAGTCCTCTGCATATATATTGTTGAATGGTCTATTGGATTGCTTATTTCTTGCTAGTCT

>UN00318

CATAGAATAAGATAATACATATCACAAATGAATATTCATCCCAGACATAAAATACGGGTCAAACACGCTCTTATACAAACTATCTGAATGAAATACTTGAGAATATAAAAATACACTGATCAAAAGGAAATGAAAGAACTACAAAAGTAAACTGGGACCATCAAAGAATAGGCTTCGACTCCTTCTTCTGCTCCGTCTACATGGCACTTCTGTTGAGACTCCTCTGGCTCCTGCTGAACCTGTTCTTCTCCGAAGTCTAACGTACACCAGAGAAAACAAGTGTACGTCAGGTGCATGGTGCACGGCAAGACCCGACCCCACAAAGAAAATAGAACGAACGGAAATCCAATGCGAAAAACGATACGATAACAGAAACAACAAAAGAAAGTACAACTAATATCCACAAATCAACATGAAATACTACT

>UN00319

TCTTCTATCGCGTACCGCTCCATTAGTTCCCTTTAGAGGATAGCCCAAGCAAATAACTGCCGAAACATGGATCTCTTCAGTGTTGGCCACCATGCAGCTGACTCTTGAACCCATGGATTTTCCTGCTAAAACAAGAGGATGCCCAGGATGCTTTGCTATGGCATTTCTTACAACATCCAGGTGATGATCAACAATTTTCAGCTTTAGGAGGAGCTTTACGTTTCCCACCAGAAATATATGGGTAATCAAAGGTGTTTACTTCAACAGCACCCAGTGCTTCCTTGAGCATTTCCTTCCATCTGATCATCCATTCTGAGGTGGACGGAGCCCCAGCGCCATGAGCGAACACAACGAGAGGCGCCGCCGACGATTCATTGTCGTTCTTCGGAGAGGCTTCGTCGCCGTCGCTCTTCTTGCGTCGCTTCGAGGGAGGGTCGGAGTCTAGAAGACCAATAA

>UN00320

CGGTTATTACTAATTTAACTATTTATTGCTCAACTAATTTTGGGAGATACAAAACAAAATTCAACTTCAAGACCAACATTTCCTCTCATACAAAACAAAATTCAACTTCAAGACCAACATTTCCTCTCATAAACCCTTTTTACAATTGCAAAACCAAATTCTTTCATACAAAACTATCAATAAGTGATTTCCTCATGTCATCAACTATGGAGCTTGGCTGAGCTTCTCTAAGCTTGAACACACTCTCAATCACGGACAGCTCGGTGGCTGTAGTGTCCGATCCACAAAAACCTGTCCAATCGTTGACGGTCATCCCTGCAGAC

>UN00321

TGAGGGAGTTGATTGAGAAAGGCTTGAAGCTAGAAGCTGATCTAAGATCTGCTGAACCATTAAGGAATGATGTGATACAACTTCGTGCAGAAGTTCAGAAGCTGAATTCTCTGCGGCAGGAACTTGCTGGCCAGGTTCAGAATCTCACACAAGAACTGAAAAGGGCACAAGCTGATAACAAACAGATCCCTGCTATGAGGACTGAGATTGATGGCCTGAAGCAGGAGCTTGTGCGTGCAAGGGCTGCTTATGAGTATGAGAAAAGGGGGAATGTTGAGCTTCTGGAACAAAGGCAGGCAATGGAGAAAAACTTGGTTTCCATGGCGCGTGAAGTTGAAAAGTTGCGTGCAGAATTTGCAAACAATGATGGGAGATCATGGGGTGTGGGACCTGTGGGTGGAGCTTACGGGATGCAAGTGGCCAGCTCTGATGCACCTTTCCCTCCTTCATACGGTGATGGATTCGGCATTCATTCTGGCATT

>UN00322

TCTAAGTAAACTAGTCTTATTGTATTTTATTATGTACATATAACGTTTACCCTTTTTTTAAGTCGCCAAAAGAAAGAGCTTCATGAAAAGGTACATCTTTTCCTTTCCATTCTTTCAGCATGAAGCAGAAACTTACAATTTCATTGATATTAATATTATTAACAAACTAAAGCAGACAAGACATGCTGCTGATGCCTCCCTTAAAAACCTGAAACCCTACTAAATTGACAGACGCTTCTCTTATATCCCACCAAAAGACGCCGCGAGCATCAAAAGACACATAAATATGCATGGACTGATGTTATTAGCCCTAGATGTGGTTTTCACAAGCTCCACATGTATCAGTAGTCTTAGGATTGACAAATAGTAGACAAGTAGAACTAACAACCTACCAAGA

>UN00323

CTTATCCTAATAAATAACCATTGTCTCGCAATATTAAGCAAGCAAGCATCAAAATTAATACATCTGAGCTTTTCCTTCTTCCACATTTTTCCCTCGAGAGTAGACCGTAGCCTGTCAGGTTCTTAGTACATGATACAAAGGGCAAAGGCACCAAGGACATATGAAAGACATTACAGATGATAGATGCACATTGAATTAATTCACATTACACTAGAATAGGTTGCTGGAGTGAAGATGTACATCTTGCAGTCATGAGAATCAAGTTGAGCTCCAAAGGATCCCGCTACGTTCTCTGCTAGGTTTTCATGCTTCCAGAGATCTCTTACAAGAAACAACTAGGTAGGAATTAACGTAAACAAAAAAGGAAAAACCGAAAAAAGAAAAA

>UN00324

AGTCCAAACTTTTTGCTCAATCAGAAGAACTCAAAGTACGAACCCTAGAGAGAGAATTAAGTACCCAAACAGCAGAAACAGCTAGTAAACAACACTTGGAGAGCATTAAAAAGGTGGCAAAGCTTGAAACCGAGTGTCGTAAGTTGAGAGCCATAGTGAAGAAAACATCATATGTTAATGATCACAAGAGCATCTCTAGCTCTGCTTGTGCAGAATCTCTTACAGATAGCCAATCTGAT

>UN00325

TTCTGTTCCTAATGTTGATGAAGGAAACTTGAAAGGTCAAACTCTGGAGATCACAGTCCAATCCTTATCTGAAACTATTGGAAGTCTTAAAGAGAAGATTGCTGGTGAGGTCCAGCTTCCTGCCAATAAACAAAAGCTCAGTGGCCGTGCAGGTTTTCTCAAGGATAATCTGTCACTTGCTTATTACAACATTGTCCCGGGTGAAACACTGACTCTTGCCTTGAGGGAGCGTGGTGGAAGAAAGAGATGAAATCTCATTTGAAAGGTCTCCAAGGATCATTTTGCAGCAAGTTGTGGTTCTGATGAGAACATTATTCTAGAAATTATGTTGGTCAATGCGTTGGCGCAAAATATTTGGACTTGTATAATGAGGATCTCTGAACTTATATTTTTATGTTTTGAACTGATAGAGATTTATAGGTTACTTAATGCAGATTTGCAATTTGGGTCCATTTCGGAAGCAGGTTGACAATGGCTGATCTGTTT

>UN00326

TTCTTTGACCAGTGCCCTTAAACCTGTCCTGGATGAACTCAAATTGCTGGACATGGGTGACCTTTCCTGGAACATTTTCCATATCATTTTTGTGGATTGAGTTCTCCCAGTGCTTCCATTGCCTTATCTTTGCCCCTCCTAGAATCACAGTGAGTACACTAAAGACCACATGAGTGAGTGCCAGAACGAAAATAAAGATATGCAACTGATGCAAAGCCTCGATAGACAATAATGGCACTTTCCCCTTTCTGCTGCAATGTGTTGTTGCTGATCCCTCTTCAGACAGAAGCCTGCGCAGAACTCCCGAAACCAAATGCTCGGTCATGGCGGACCTCAGATTGTTCATCTTCCTTCTTGCACGGGAGCATGTGATTAGTCCACCCCTCAGGGATGCAGATC

>UN00327

TTTTAAGGTTACTTACCGGTTTTTCTTAACTTCGGTCCCTACTAGGGTACTAGTTACCGTATACCACCGATCTTGTACACATCCTGGAGGGGGAGACGGAGGGGCTTGTCAGAGGGCCTCTTGGGCTCATTGACCTGGTCCAGAGCTTCGAGGAGGGTTGGGCCCTTGTACCAGTCGAGGTTTGTGGATCTCTCAATCATGTTATCACCTTCGAAACCAGAGATGGGAACAAAGGGGATCTTGTCAGGGTTGTAACCGACCTTCTTGAGATACGAGGAGACTTCTTTGACAATTTCTTCGTACCTGGCCTTGGAGTACTTGGGTGTGGTGGCATCCATCTTGTTACAGCAACAAATCATTTGCTTCACACCAAGGGTGAAAGCAAGAAGAGCATGCTCACGGGTCTGTCCATCCTTAGAGATACCGGCTTCAAAACCACCAGTTGTGGAGTCAATGATGAGGACAGCACAGTCAGCCTGTGAGGTACCCGTAATCATGTTCTTGATAAAGTCACGATGTCCAGGAGCATCAATAACCGTGCAGTAGTACTTGGTGGTTTCAAACTTCCAGAGAGCAATATCAATGGTAATACCACGCTCACGCTCTGCCTTCAGCTTGTCAAGAACCCAGGCGTACTTGAATGACCTCTTGTTCATTTCAGCAGCCTCCTTCTCAAACCTTTCAATCACACGCTTGTCGATACCACCTAGCTTGTAGATGAGATGGCCAGTGGTGGTCGACTTACCAGAGTCGACATGTCCAATGACCACAATGCTGATATGATCTTCTCCTTACCATGGTGAATTGAACGATCAAACCCTGCTCGAGGCGCACCTGCAGCCCGGGGTCCACTAGTTC

>UN00328

GTTAAACAAAACAAAGAAAAGAACAACGAATAGACTTAAAAGTAAAGTACTTACAAATAATACGGCCACAACATTCCTACGTAACAAGACCACTCGGTAACGAAACAAAATCTCCTAATCAAAGAGAGACAACATCCTACAGGATGGAAGTTTATAGGTTTTCCAAAACTCGAATAACACGTTACTAATGTCGAGCAGTAAATCTGCTCATCACTCATCTTCCTGGCTCCTGAGCCTGGCAAGCTTGTTGGTGACAACAATATCGAGTTGAGCAGCCCTCTCCACTATCTCCTTCCTCTTCCTTGTAGAGACATTGTGTGCAATCTCAGCACAGTAAGTCCTGTTATGCATCATAAGCAACTCCAATTCAGAGACATTGTGGACAAGGAACTTCTTGAATCCATTTGGCAGTATTAGTTGTTACGGGTTTTTCTTTTGTTTCTTTAGTTTAACCTTTTA

>UN00329

TTACGTTAATTTAGGCTTGTTCGCTTCGGCCCCCGAAGCCCTCATCTACCGATTCCTCCCCAAATCCGAACCTAGGGTTTCGTCGGATCCCATCGAGTACAAGGAAATTTTCTGTAGATTATACTTTGAGTTGTGAAGATGTTTGGCCGTGCTGCTCCGAGGAAGAGTAACAACACTAAGTATTACGAAGTTCTTAAAGTTTCGAAAAGCGCCGGGCCTGATGAGTTGAAGAAGGCGTATAAGATTGCTGCTTTGAAGAATCATCCTGATAAGGGTGGGGATCCTG

>UN00330

AAAACTTAAAACTAACAAATTAATTAAACCAATCATACATAATATAGTTGAAACATTACATGCAAGCACATGATCAACTCCAGGAAAAAGGGACTAAGCAACTGAACATCAACCTCACGACTCTCTCAAATTACTACAAATTAAACCAATACTACTAATTACTCCTTATTACCTCATAATAATGATGATGATGATGAT

>UN00331

GTTTCTTAATTTATTTTATTCTAAGTAACTACTATATACAATAAGAGGCTTCATCAACTCTAAAGCAATATCAAGACTCAAGAAATGTATTTCTATGTGAGAGATATTTGCTATTTTATTTTCTCTCTAGTACTTAAATGTAGGCAAGTAGTTATCGATTTCATTTGAAGCTAATTGGGATATCACACACACTTGCGCACTTTCAAAACTCGTATTTTGGTAGTTTTGTTAAAATGATGATTTTCACAAATA

>UN00332

TTATCAATGATCCTACAAACCCAAAACACTATTGGCGAATTACCGAAGTAACAATAGTAACTATAGAGACACTGTTAAAAGATGAGGATCTAAAAATGACCATAAAAGATCTTGTGGCGACTAGCGGGAGATCATATCCTGGGATTGACATGCAAAAAGAAGTTTTTAGCAAAACGACTCAGAACACAATGAAAGAGCAGATTGCAAACAGGAAGGAAAAGGCAAATTCCGCCCATTTAAATGGTAACCACTAAATCTAGTGAGCTTGCTGTCTCGCTATTGTCATATGATATTTCTTTTCCTATCCTACTATCCTGGCATTCTCGGCTAAAGAATGAGTGTCACTGAAACTACTTGATAAGCATAAAATAATCTTACCTTATCAGTGACCAATTTTGTCCTGTATACTGCTCATATATTGGTGCACTCAATAAAACCAATTTTAG

>UN00333

GTTCGGCTCTAACAAGCGGAGAGAGGAGGAGGAGAAGAGCAATGACGGCGGATAAAGATGTTACGATCCGAACCCGAAAGTTCATGACCAACCGCCTCCTCTACTTCGACAAAGACAAATTACGTTAATTAGATGTTATTCATCCAGGGAGAGCGAATGTCCCAAAGGCGGAGTTGAAGCAGAGGCTGGCTGCGTTGTATGACGTGAAAGATCCGAACTCGGTCTTTGTTTTCAAGTTTAGGACTGCTTTTGGAGGAGGAAAGTCTACTGGTTTTGGTTTGATTTATGATTCTGTCG

>UN00334

GTTTTAATTTGTTTAAGGTTTACGAGTTAATTGTTAATGTTACGGGTTAATTGTTAATGTTACGGGTTAATTGTTAAGGTTATGAACCCCCTCTCTCTCTCTCTCTTTCTTTTTATTATTATATGATGATGATTATTTTATTTATTTTCATTTTCTCTCTCTCTCTCTCTTTATCATGATGTACTAAGATGATAGTTGAAATGATTGATGAGGATTATTTTATTATTTTCTATTTTT

>UN00335

TGACTCGCAGTTATAGAATTTTTGGCAAATCCTCAGCCGTCAACGGATTTGATGAGCCTTTACCGACTACCGTTTGAGTCGGGCTAGTATGCGGATTATCTAAATCCGTTCCGCATATGACGTCGTGCTAAGAGAGTTAACGCTCTTAGTGCTACGATGTTGAATACGTAGGCTTCGTCTTTCTCACGGGAATAGACTACCCGGCGACGCGTGTTAGGCGTTGACTTTAGGATTTTTGGCTAATCCCCAGCCTTCAACGGATTTGATTAGCCTTTAACCGACTACCGTTTGAGTCGGGCTAGTATGCGGTTTGTTTAATTCCGCTCTTATGTGACGTCAAGCTAAAAGAGTTAACCCTTTTAGTGCTACGATATTGAATAACGTAGACTTCGTCTTTCTCCTATGAAAAGACTAAACAACGACGTGTATTAGACGTTGACTTAGGATTTTTTGGCAAATCCTCAGCCCTCAACGGATTTGATGTGCTCTTACCGATTACCGTTTGAATCGGGCTAGCAAGCGGATTGCTTAAATTCGTTCCAAATGTGACGTTGTGCTA

>UN00336

CTTAAGAACAAAGGACTACAAAAACCTAATAGAAAGGAAGTACCCAACCTCCAATATGACAAAGTGAAGAGGATGGTCATTCCTGATGCTCTCAAGGTTCTGAGGCTTCAGGCTGGTCACAAGTACTGCCTTTTGGGTAATCTATCCAAGGAGGTTGGATGGAACCACTATGATACCATCAAGGAATTGGAGGAGAAGAGAAAGCAGAGGGCACAAGTTGCCTATGAGAGGAGGAAGCAGCTGGCCAAGCTTCGTGTGAAGGCAGAAAAGGTTGCAGAAGAAAAGCTCGGTCCTCAGCTTGAGATTCTAGAACCGTTGAAAGTAAATTAAAAGAAAAAGAAAACGTTTTAAAAAGT

>UN00337

TTACCGTTTGCTACCAACGCTTTGCAATCCGTACAATGCAGATGCAACTGATCATGCTTGTCGTTCTGGAGAGGTTGATTTCAATAATGCAACACAGGTATGGAAAAATTACGTATGCAATGCAACCACCACCTCCGGATGAGATATGCACCACCGTGGGCCGCCTCACTCCGACCATCAACCGTCAAATGACAGCCGCAGTCACTGTCAGCCATGGGCTCCACCATTACGGCCCGTTCTTGGCCCAATTAGAAGACTGCACTTTTGTTCGACAAACTTTCAGCTCGATCAGCCAAAACAACTGCCCTGGCCTCAGAAAATATAGTAGGTGGGTTTATATTGGGCTTGCGATGGTTTCGACTGCTGTGATGCTCTCGTTGATATTTTGGGTTATTTATGCGAGAGAGCGACGACATCGAAAGTATAGTAAAGAGGCAATGTTGAGAAGACAGCAGAAGCAAGGTGTGGTTGGGGGTATGTGAGGTTATGGGTAGGGCAGTGTACATTGGTAGGTTTTGTTTATATGAATTTTCTTTTCTTTTCTTCTCTTTTCTTTTCTTTTGGGGGGTTTTGGTGACATGGTTGTTGTGTGTTGGTTGATTATGTAGAATGTTATGTATTGATTTATTGTTTGTGGTGATATGTTATACTGCAGTGTAAGTGGTTTTTATCAGATTTGAGATTTTTGAAC

>UN00338

GGTTATTTAAGTTCCTTCGTACCCTACCGTCGAGGGTGACAGCTACCGAAAGAGGGCGAAACCCAGAATCCTCGCTCTCGAGTCCAAATGCAGGCCCTGCTACTGCCGTCGTCGTCGCCGCTGCAGGCCGACGCGGTTCGCTGCAGCGGAGCTCCGCCTCGCTCTCGTTATCCGCGAACCCTAACCCTACCCTTCCCTCAAAAACCCCCCTTCTCTGCTCCCAAGATCTCCCCTTCAATCTTCTCCCTTCTCATTCGGCGGAGAAATCTCGAGAATCGCGGAG

>UN00339

TTTGTTCCAAACCATATAATCATTCTAGCACCCAGTAAACATGATTTGGGAATAAACACCCAATAAACCCGGAGGGTTATATAAACACAAACCTCTCAACGAAAATCAAAACTAGTAATCATGAAAACGAAGGAAATGATCAATTAAAAATTGTTTTCATATCCAAAAAAAGATTTAAACACATAAATTTCAAAACCAAGAAAAAATAAAAACACTAAGTTCTTGAAAAAATTCATTTTGACACCCAAGCATTCTTGGTGCCGGATTGTTTAGGAACATGACCCCTATCAATAAAGCTCAAAGCCCTAGGGTTATGAACCCTCTTAGGCATATGTGGTGCACTAATATGGTCGAAGCAATATTTACTCAAATGACCCTTCCTATTGCAATTTGTGCAATGAAAGAGATCCATTTGTTTTTGTGGACCATTAGCTCGTGCACGGGAAACATTAAAAACATTTGCAAACATAGATGGTCTTTTAAAATTATTTTGCAAATGAGAAGCCTTATCAAAAC

>UN00340

GGTTAACACATCAGCATTTTCAGCAGCCTGAATAACTTCTAAGAAACTGGGTTGTGTCATCTGCACGAATAAAAGACTCAATTGCATCACTAACTAGCCCTTCCCGCAGCTGGGCCTTCGCCACCTGACTCCAAACTGCTTCTTCTTCAACACGGAAAGCAAACTCCACCGCCCGATCTATGTTCTGAATGTTATCCAGAAGAACATTGACTAGCTTGGACATTCAAATTGAACTTCTTAAAGATAGCGAA

>UN00341

CTTTTATTTTTCTTAAACCCGTTTCTATTATACTTCATACAAACAGAGTACTCATTCATCGCAATATTTAGGACCATAGTCTGCTGTTGAAATTCTGTAGTGTATTCTTGAACAGTTTGATTGAATATTTAGCGCAAGTCGTATCACTTGTACCAACGCTCTTCAAGGAATCTCACTGGATAAAACTATTTCCTAAGCAGATCCTTAAACTTTTTTCCATAACACCGATTTCTTCTCCTGCTTGTAGTAGGAGTTCCACC

>UN00342

TTTCTTCCTCTCAAGGGCTAAAAGGGGTTCGTAGGTGCTATTGGCTCAAGGATGGATGAACCTGGTCCCTATCCTTCCATAGATAGCGATGCTGGAAAAACGTTGGTTCATCCCGAGACGAGAGAGAAAGAAGAGTGGGAACATCGAATTCTTGGTCCAGAGAAGCCATCATACATAAAACTTCAAGAGTCTTCAGATGATGAAGTTCATTATACAGGTAGCTCGAGGAAAAAAC

>UN00343

TTAACTACATTTTAATTTTTATCCATAGAATCTAATCATTAAATAATATCTTTCCATAAAATTCTTCTAAGATTCATTTATTTCAGCAATCCCCCTCAATTTCGTAGCTGACAGTAATTAAGATTAATAACATATCATGATTTCAGAGAAGGTATCTTCTAATTTGAACCTTCCCTATAGTTATCTAAATTTATCACCACAGGCACAAAGTGAAACTAAGTCTTGAACTTGAACCTTTAATGTAGTGATATTCATTGGTAACAACACGATATAGG

>UN00344

AACTTACCTAAAATTCCTAGTAAAATTATCTCAGAACTTGGGACAAAGTTTTCCGTCTAATATAGAAAAAAAATCGATTTCTTGACAGTTTCCCTTATGTTCTCTCTTTCCTGATCTCTATCAAAAAAATACCAAACAAACAGATTTTGAAATTTCAATGCCTCCCCAACAACAAACATGGGGTATACTAAGCTCAAAGCAAAAATTAGTGCACGATGGACTTAATCATGCAACACTTCACCATAAGCCAATAAACTTCCACCAGAAACCCCCAATGCCAAGCCAAATGATAATATTGACGACGGAGATGAGGAATCCATAACCCCACCATTTCGCCAATGGAACGTAATTAGCACCGTAAAAGACGGGGGCAGACCCGATACCATTAGGTTGGGGTTTAGGGTCCCGTCCCCTATTGTAGGTTTGGTAGTAGGTAAGGTAGTAGGTAAC

>UN00345

TAAAGAACCAATAAATAAACTAAAGTACACATACAAGATATACATCTTATTTATAGGCAAATGATCAAATTGTAGAAACATGACCTAAAGTACAATTCCTTGAACGAGCTAAACCGCTGCAATGATTCAAAACTCCAAACACAAATGGACTAAGAATAGGCTCAGCTCACAAAAAAGAGAAAAGATCGAATGCTAGAGCCGAATAAAAATCCACCTAGACAAAAAAAAAATACAACTTAATCTCAGATTGTTGGAAGACAAATTGAATCAACAAAATTCACCCCGAGCACGCAAAAGAAGCATATGTGCAATACTGCAATGATTAGATAAGACAATTGCAATAAAGAGAGAAATCCTTGAGACTTAGGAAAGGAAAATGAAGAGATAGGGGCAATCTTGTCTAAGCAAGTGCTTCAAGAGAGAAGTGCTT

>UN00346

ATTTATGTAGTTATAGATTGTGTTAAGACATGTTGTGAATTTTGAATTCTACCTATATGTAATATATTTGTTATTTTTATTGTATAGTGAATGAATATTGTTTGAGAAC

>UN00347

GAGTTCTGGTCTTCAACAGAAGAGGCAGCAACAACAGCAACAGCAGCAGCTTAGCAAGCCACCACCAGCGGCGATGAAAGCTGGTGGATGGCACAGCCAGACCGATTACCTTGGGCGGTCTTATGAAATCCCGTCTGCTAGGCGCTATTCACGGAAGGTTTTGGGTTGAGGAAAAAGATCTATGGGAGGCATTTATACGTTGCATGCATCCATATTCGATGTTTTATGTTTCCGATGGTTTGATTTTTTTTTTTAATGTGTGCTAATCCGATACCGGTGGTGTTTAAATAGCTAGATTCGGTTTGCATTGTGTGCG

>UN00348

TACTTTAGAAGTTAATTAGAAATGAATGAAGAAATGGGACAAATAACGGTCTTTTGGATAAACAGGTACATTTTTACTAACTTTCTAGTAGTTAATATAATGATGATATTACTAACTTACTAATCATGCTTTTTTTGTTGAGTATTTTCATATTAGAGACTTGAAAGTTGAAGAAATGGGACAAATAACGGTCTTTTGGATAAACAGCGCGCAATAGCCTTAGCGGTACCTCGCCGCGACGCGACGCGATCCGCTATCAATAACCTTGCCCTAGATCTCACCCAGCGCGATGGCGGCGATGCTCCAGCCTCAGATCATACTTCTAAAGGAAGGCACCGACACGTCGCAGGGCAAAGCGCAAGTAGTGAGCAACATCAACGCCTGCACCGCCG

>UN00349

TCCCCAGTAGAACTATGACTTCTAGTATCACTAGTGTCAAATGCTTCAAAGAATTATTTTTTAAATGTTCCAAGCATTTTTGCTAAACTTCTGTATCAGAGGCCTGGGGATATTTTCGCTTGATCATCTTGGCTATTTTAATCATAGGATTTTCTTTCTGTATTTCTTGAGTCAGAGAAGTCTCAAGAATAGTATTATTCTTTTTAATAATTTCCCCAAATTGCTTTTTTTTTTTACTCTATTAGAATGTCAGTCCATCATCTACAGTAG

>UN00350

TTCCCTTATTTAAATTCTTCTACTTCTTGAAAAGTGAAAAACCCTAAACCCTAATCATTAAAACCCCAAAATGGCGGCCATCGCCTCTGTATCCTTCGCCTATCTCGGGCACTCATCTGATCGGAGATTCTCTTCCTCTGCTGCTCGATTCGTGGGGCCCACTGGATTCGGTGGCTTCAGGACCAAAGCTCGTAATTTGAGTGCTCGACTGATCGTTCGCTGTTCTGTAGGCACAAGTGATGTCCCGCCTGCAGTGTCCGAG

>UN00351

TTTTTCCTTTTGTTCTTATTTTTGGTCCTATTGTTAAGCTCCCTAGTTCTTGTTTACTGCTCTATTTATATATTGTGTAGGAATTACTGGAGTTTAGCATGGATTCGGAGTCAAGAAGGAGTCAAGGAATGCATGAAAAGTGGCTACACGGTCACTCAGTGTTCAACAACACAGGCGTGTTGGATTACAGAAGATAAGCCCAGAAAGCAGAGTGAAACAGAATTAAACACAGCC

>UN00352

CCACTGACATATTGCCGATCATTTGAATAATAACACAGAGCAACAATATCAGTACAACGAGTACTTATAAATTTTAAATATAAAACTACATCATTCAGTATTTTTATTCAAGACCTCAACATTTTTTTCCAGCCTTTCCAGCCTTTTTGTGATGTTCTTGGTATTCACCCACCCATTCGAAGAAGTTACAACCCTTGCAATTAGTACCCTTGTATCTCGGGCATCCATAAAATTCTAAGCCCCTATTATGTCCTTGCTTGCACATCGATATTTTGCAAATTAACCCACAATAACATTTTTTTTGAAGTAAGCCCTCATCTTCTTCTCCACTGAACTCTTCCGAAAATTTCTTCATCTTTTATAAGAAGAGGATGAGATGGAAACCTTGCTGGGAACCGAACAACGGGGCTGAAGTGGTGAATCCGAGCACTCGAGGGATTTTCGAGGGTATTTTTTTTGAAAATTAAATGAGGAACTGAACTTGGGAGGAGATAAGGGG

>UN00353

GCAAATTCACTGTAACAACTAAACGAAAAAACATTAGTCACTGACCACTATACAAATTCAACATGAGCAAATTACTCAAGCAAAGACCAACAGCATAGTTCCAGTTAAAGATGATCAAAAAGTGTAAGAAAGCTCAATTGTATAGCCATACATCATCGAAACCATTTCATTTTAGAAATTAAAGTTTTCAGAATCGACCTATTGAGCCGCATCTTCGGAGAACCTAGCGTCAAATGGAGCTCCGCCAGCAGCAGCGGAGGCAGACG

>UN00354

CATTACCACTCCCCCATCTACTGCATCCTTCAACAATGGATCCAATATCGCCATCACATTCGCTTCATTATAGTTCTTGAAAGCCCATCGGACAGTAATCCTTTCATCAGTACTCCTCTTCATCTCTACAGGGCGGCGTCCAGAAAGAATTTCAATAAGCAAAATACCATAGGAGTACACATCACTTTTGGGTGTCAGCTTAAAAGTCTTGAGGTATTCTGGATCAAGGTACCCGGCTGTTCCTTTCACTTTGGTCAAGATATGGGTTTGCTCTGATTCTGTTGGACCTATTCTAGCAAAACCGAAGTCAGATACTTTGGCTCGAAAGCTTTCTGTTAGCATGATGTTCGATGACTTCACGTCTCTGTGGATTATTGGCTTTTCTGCATATAGATGGAGATAAGTTAGAGCATGAGCAATGTCTATAGCTATTTCCAGTCGTTGATTAAAATCCA

>UN00355

GGGTCTCCTGGCTCGTAACTTTGAAACTTGTTGATGGCATCGAATTTATCGGATTGCTCCCTTGCAGGCTTAGAACACATGCCGTCACTGAACCATTCATCCTAGCAACAAACAGGCACCTGAGCGCACTTCACCCAATCAACAGGCTTTTGGTCCCTCATTTCCGTGACACGATGAACATAAATGCCTTTGCTCGCCAAGCCCTCATCAGTGCTGGCGGTATCCTAGAATCCATTGTGTTTCCGAACAATTTGCAATGCAAATGTCTGCAGAGAT

>UN00356

ATTAAAGTCCAGGCAACATCAACCGAAATCTGTTTTCTCTAATAAATCGATAGAAACGAAAAACACGACAGTTTGCCTTTGACCTGATTCGCCAACCATGCTACTAAGAAAACATAAACTTTGAGGTGTCCCTTTTCAATCACCCAAATTATTCACATCTACTGTTGAGTCTCAGCTTCTGCTGTTCCTTCTTCAGTCTGGCGGCCGACACCTGATTCGTGTAACTAGTCTCTCCA

>UN00357

CATCATGCTTCCAAAAGGCTGAGTGAAGAAAGGATCATCAAAAGGGTCCCTCCCACCGAAAAAATTTGATATTAGGCTTTGAGGTCTTCCCCCAAAGCCAGGAAACCCTGCAAACAGGGTAAGGACAATCTACTTAATTACTTGATAAACTAGAAGAGTAGTAAGTTCGTCTATAAACAAAATAGTAGCACAACCATAGTCTACTTATTTCCCATCTTTCAAATGTTCAGGCATGAACAGGATCATGACAGAAGAAAGAAAAGACCAAGAAACAAAGATCAAAAGGGAGGGATTTTACGTAGAAAGTAAATCTTAGCCCCCAAATTGACAATGCTGCGACTGACAATAATCATAACGCAAGGAAACAATATAAACATATTACAGAATCAACCAAAATCAACCAAAATCACAGCTTCAGCAGCAGCAGCAACAACAGCTTCGGCAGCAGCAGCTTCAGCAACTTCAGCAGCAGCAGCTTCAGCAACTTCAGCAGCAGCAGCAGCGCTTGCTTTCTCGTCAGAATTCGTCTGTGCCATTTATTGAACAAATTCAAGGTTCTCAAGAGAGCTCGCGGACGAGGGCGGCTAGGGCTTCGGGATTGACTCCGCGATCGCAGAGGGAGATGATGAGGGAGAGGGTGATGCCGGGTCGGAGGCGGCGTCGGAGGCGGCGTCGGAGGCCGAGGAGGAGGCGGAGGAGGAGGGATCAATGAGAACTCAGCCGCCAGAGAGAAAGAGGAAGAGATTGACTCCGCGATCGCAGAGGGAGATGATGAGGGAGAGGGTGATGCCGGGTCGGAGGCGGCGTCGGAGGCAGAGGAGGAGGGATCAATGAGAACTCAGCCGCC

>UN00358

TTATTTATTTGGGTTGTATGTGATCCAATAATACAATGACAACTCATCTCATCTCATTTCATTTCTTATTTTTTAGAATGAACCGAACAGAAGAAAACAACAAACTACAGGAAATGAATTATAAGAAAACTATTTCTTGTAATAGGTTGTTTTCTTTTGTTTGGTTATTTTTTAAAAAATAAGAAAATAGATGAGATGAGATGAGTTGTCATTATATTATTGGATGATGTGACCCACACTTAGGGCCCGTTTGGTTGTATGTTTTTACATGTAAAAGGATGG

>UN00359

CTCCTCTCTGTTCTCTAATTCAGCTTTTGCATCATTGATGATGAGGCAGAGCTTCTCATTAGCGAAGGCCTCATGGTTCGCTGTAAGGTGCTTCCTGCAAAAGTCCACCACTTCCTCATCAATGTCGCACATGATGATGCGCTCTATGGTGTTGTGCTTGAGTATTTCTCTGGCAGCAGAACCTTCACCGCCTCCCATAATGAACACTGTCTTCGGATTTGGATGGCAAAGGAGGGGAGGGTGGATCAAGCATTCATGGTAAATGAACTCATCCGACTCGGCACTCTGCATCTTCCCATCAATCACCAAAGCCTTCCCAAAATGCTTGGTG

>UN00360

CTCCAACCAAAAGAAAGGTCAGCCCCCTCTTTCTCCTCAGATCTCCACTAGGCTTCTAGGGTTTCTCTAATTCCTCCCCGTTCTAGTCGAATTCATAGCTAAGGAAGAAGATGGCAGGTAAAGGAGGGAAGGGGCTTCTGGCTGCGAAAACGACGGCGGCCAACAAGGACAAGGACAAGAAGAAGCCCGTCTCTCGCTCTTCTCGTGCTGGCCTCCAGTTCCCCGTCGGTCGAATCCACCGCCAGCTGAAGTCGAGGATCGCCGCCCACGGCCGCGTCGGGGCAACGGCGGCGGTGTACTCTGCCGCGATTCTCGAGTATTTGACTGCTGAGGTGCTGGAGCTCGCCGG

>UN00361

CCAAAGCTTCCCAGTGACCAGGGCGGTGATGATGGAGATTCTGACATAGACCTGGATGACTTTTCTGATGCAGAAGATGGAGAGGAGGATGAATATGATCAGCTTCCTCCTTTTAAGCCTCTTAAGAAATCCCTAGCTTGCCAAACTTACCAAGGAGCAAAAAAGGGCCTACTTTGATGAATATGATTATCGAGTTAAACTTCTTCAGAAGAAACAGTTTAAGGAGGAGATTAGGAGGTTGAAGGAGATGAAGAAGAGAGGGAAAAGCGGCAGGGAAGAGTCACCCTATGGGGACATGGGTGAAGATTTTGATCAGGATGGTGCACCAGCTGCAGTGCCTGTT

>UN00362

TAACTTTAAAGTAGTTACTACAAGACACATACAATTACCATGGTTAAGGAGTCACAAAAATAGAGAAAAAATAAACATATAAATGAGCTTTTCAAGAAACTCGTTTTTGGTCGTTTTTGGGACAAAAACAATACAATCAAGTTACAAAAATACTATAAAATGCCTTGGATTTGCTGTTTCCGTGGATCTCATTCTGCCGTCTCAGTCAACTTGAGCAGCCCCGACATTATCCCTTCACTTCTAGTTTCATCATTCTGCTCTTTGCTCAAAACTCGTGTTCAAAATTTGAAGTTAATATGGCAGCTTTGGTAAGCGCTGCTGATATGGCACTAACATGTGATTAAATTATAGCTCAGTTATTGTATTGGGCTCCCTTGCTCTTATTCTTCTTTATCTTTTTAGTTCGCCCTTCTCCTTCTAGACAAGGTAGGTTTTACTTACTTTTACAAAAGAACCTTAACAAAAGTTTAAAATAAACAAAAAGTAAAAG

>UN00363

ACAGAATATCTCAGATTCAAGCATTTACCGCCAATCAATGCAGTTCAGAACCAACTTTCATCAAAGGCCACTGAGAGGATGAGACCAAAACATAAAAGACAACCCAACAAATTGATTTCAAATGGTCCAAGCATCGAATAGAAACTAGCCAGATTTTTTTCTGAGCGCCAGGAAGCTTTTGGTAAATGCATGAAAAGAAATCACAGGCTGAAGGAACATAAAATTACATCTAGTCTTCAAAGCACATCGTCTCTGAATTGAGCATTATTTTTTCATCACTCATACAAAATCCGTATCATCCAAATCAACGCAAGGAGTTCTCAGCATTTCAAAGTTTGGCT

>UN00364

CACACAAAGCAAGAGACCGCAAAACAGGATCTTGCGATCTGTTCGACAAAAGAGAAAGAGTTGTAATTTTCTTTATTTCTTGTGTTATTTGATTTTCGTTGTAACCGGATCTAAAAAAGACCGGATCTTTGTAATCGATAAACTATTACAAGAATTTGCAATGGAATCAATGATGAAGAAAACAATGAATTTCCCCGCGAACAGATACCAATCGAAGGCGGCTCCGCGAAGAGTGCCGACGAATGACCGCACCATCAGAGGCTCGTTCCCAGAGATAGCACCCAGGTGCGATTTGAAATAAATCAAA

>UN00365

AGCTACCCTACAGCAGTCTCAACCTTATGGCGGGAAAGCAGAACACGACGGAGGATGAAGACCCGAGGGAGCAGCTCTTTATTTTGAAATTGTAGATCAAGACCAGTTTAGTTGATGTCATAGTGGATCCAGGGAGCCAGAAGAATTTGATATCTGAGGCCTTGGTTCAGAAGGTAGGATTCAAGATGGTTAAACATCCGAAACCCTTTCCTTAGGGTGGATTCAGAAGGAGGCGGGATTGAGTGTGATCAACCAATGCACTTTCAGATTTGCACTCCACGAGTCGTATATTGACGAGGTCACATGCGACGTCGTTTCGTTGGATGTTTGTCAAGTAATACTGGGCAATCCTTATTTATAAGATAGGTTTGCGGTCTACGATAGGCGAGCCTAGAAATATACGTTTACCAAAGATGGCGAGCAATTTATCTTTCGTTCGGCATTTCTTTCACAGGAGACGAG

>UN00366

TGATTTATGGATACGCTGCAGAATGGCAGAAATACATGAACATAAAGAGTTATACATTAGCTTACATAATATACCAGCAACCCACTTGGGGAACTAGACCTTCAACTTCTTTCTGAAGACAGCAGGAAAGAAAGAACCAAGAAGGGGCCAGGTTGTTACTATAGCAATATAAGCAAGCAACCAGATTGATCTCCCACTCTGCCTTGTGAGCTGATTCATCGATCTCTTCACTTGAACTGTCA

>UN00367

GGGTCGTTATGGTCATCATTCTATCTGATGGCAGGTGCTCTGCACGTTGGTTACACAATATGACCAAAGTTATACAATCCTTCATTTTGGAAACAAGATTAAAAAAAAACATGATCACACATCACATAAAATCTTTAATTTCTCACGAAATTAAGACAATCATTTCATACGTCCCAACAACGAGCTCTAGCCCCGCTTGACCGGATCGAAGTTCGATACCCCGACGACCACTCCGATGATAACAGCCACCACCACGCCGCCGGATACAATGCTGAGCAAGAAGTTCTTAAGGGACGGCGAAACCCCGGGCCCCGCCGCCTCCGCTATCTACCGA

>UN00368

CTTACGGGGGTCTTTCTAGATAGGCCCTCTGGCAGTAAGAGAGAGAGGCCGGTTGAGTTTGATAAGCAGGAGGAGAATGATCCATTCGGTTTAGAGCAGTTCTTGACAGAGGTGAAGAGAGGAAAGAAGGCTATGGATAAGATTGGTGGTGGAGGAACCATGAAAGCTAGCGGAGGGTCTTCAAGAGATGACTATGAAGGAGGTGGCTCGGGAAGGACCAGAATTGCCTTTGATAAAGGGCGTTAGAGGATGGACCAAGGAAGGACTCCTAGTTTATCACCTGGATTTTATTTTAAAACTGCTGTGGCAAAGGTCAGCTTCTAATTATATGTTCGTTCCAGTGTTGAATGTTTCTTCAAGGTGCTTTTATTTGCTATCTTATTAGAGACACGCTGTTTGTAAAGTAACTTTTTCGATGTTTGCATTTGGATGTGTTGGATTATTATGTGTTGGTATAATCATGACTCTAG

>UN00369

CAAAGAAAACCTAACTAAGTAGGACAACTTTACTACCAACCAAGACCTTTAGTAGACATCGAGTATCGCTTTTTCTTCTTCTTGAAGAAGGATGTTCTCTTTAAAGGTAGAACAAGATTCCTTCTTTGACAAGTCTCCAGCAGAATCTGACTCCAGTTTTGCAAGCTTCAGTTGGGAAGCCAACTTAGAGAGAAGCCATTGCATAAATCCAGGAATAGGACTAAGAGTTGCAAAGACCTATAGAAACTATTGTATTACTGT

>UN00370

TAAGTTAACTAAGAAAGGAAATAAGGTTTAGAATAAGAAAAAGAAACTGAAAAATAGAATACGTTGGAAGGGACTTGATGATTTAATGCCAGGAGGTGCGGATGCTGATATTTCAAATTTGCAGTTGGAAGTTGAAAGTCTTACTTTGAAAGAGCAAAGCTTGGATGCACATATAAGCAAATTGCGTGAAAAATTAAGTGATCTCAGTGAAGATGAATACAGTCAGAAGTGGCTTTATGTGACAGAAGATGACATCAAAAGG

>UN00371

AATACTAATTTAACGTTCTATATTGGGCTCAAACTGATAATACTAATGCAAAATCAATATTCATATAAGAAGAGATTACATTTGTGCTACAATTCCCATCAAAGAAAAATACTTCTCCTTGAATTTTTTGAAGCGAATTCTTCAATTAAGTCATCATACTCAAGTAGTGATTTTGCCATTTCATCTTGACGTTGCTTGACTCTTCTATTATGAGCTCCAGAGTTTTGTTTTCTAATCCTTACTCGGTTTTTGTCATCTCCCGTCATATTGTACTGTAGTCTGATGGGGTGAAGCCTGAAGGACGGGTCACGGGTCTGACGGCGGGAGCAGAGTAAGGTCGTCGGAGGATCGCCGGCTCGGAAGGAGGAGAAGGAAGACCGACCGGACTACGGGAAAGGAAGGAAACTAAGGAAAGGAAAGAAAATAAACTAAAG

>UN00372

AGTACGCTCAAGAAAATTAATTTTCAATTATTATGTAATTAACAGTACATTTCAAGACATCAGAACAAAATTAGCTCTCATTGAGACTTTGCAAACGTTTGATAATTCAGAAATTTTATCAACCACCAACAAGATAAAAAGCACGTAGCATATATACCTTCACATGCATATATAGTTTGAAAGTGTCGAGGGGGAAGCGATGGTAGATCCCAGCCGGTCAACAAGATAAAAAGCATGAGCGATCGAGGTTCTGTTACCGCCGGTCGTCATCGATCTCCGTCGTCATCGTACATCGATGATCATCGCCGGTGATATTGCCGATTCCGGTAGCCGACGCGGACAAACCACCCCAACTCAATTTCTCCCTCCCTCTCTCTTGAGTCTTGACTTCAGCGTGAAATCAAGCTGTG

>UN00373

AGGTTTTCTTTTAAACTTTAAAGTCTACTATTTAGCATAGAATTTTTAAATATAAACCGTAAGGCACAATGTAATGTGAATAAACTCAGTCCAAACCAAAACTTAATTAAACAAAACTATGATAGAACATCATGATCATGATAGAGCCAATGATTCCTCATTTCTTCTTTCCACCCTTTGCAGCATCTCCGGCCTTTGTCTTCTTCACACCACGGATCTTCTTTGCTCTGTTCTTTCTTTCCTTCATCTGCTTCCTAGACTTCTCAACCTTTGTAGCCAATCCATTCCTAATGAGACGATACTTGGGCTCGAACTTCTTCGCACTCTCAACAGAATCGTAAATCAATCCAAACCCAGTTGACTTCCCGCCTCCAAAAGCAGTCCTGAACTTGAACACGAAGAATACGAAGTTACGGAATACCTTAAACAATAACGAA

>UN00374

TACGAATACAACCAACCTACGGACCGACCAACGTCATCCCTCTCCGACCGCCACGCGGGCCCCACGTCCCGCTTCTACGCCGTCTACCGCATCTCCCCCCTCGACCGCCGCCCGGCCCACCACCTGCACCGCCGCCCCCTGCCCGTCTCCCGCCCCGTGGTCCCATCCGACGACCGCCTAGGGCTACGTCTAGGGTTTGGCGGGGTCACCTCCTCCTCCCTCCAGGATCGCGCCAAGGACATCCTCATCGTCGTCGCTGGCCTCCTGTTCGGCCTCGGCTGCGGCGCCGTCACCGCCTCCACCATGTACCTCCTCTGGTCCTTGCTCTCCGATCACCGCCGCATCGGATCTGACGAGGGCGACGACGGCGGAC

>UN00375

AGTTAAGAGGAGAAAACTCCAACAACCCACCGACGCTGAATAAAAAACAAACCACAGCCAAAGCTGGGAACACTTAATCCTTCATCACTCTCTCCCCCCTTGCCTCCTCTTTCTTAAAAAAATAAAAATAAAAAAAAAATGAAAAAAAAAGGAAACAACTTCATCTGAGCATGGAGGTGAGAGATCTGAAGACGACCTCCTCACAAGGAATAGTCAATCCCATATCATGATCGAACCCAAACTCCTCCTCGGCTTGCCTCAACAGAATTTGGAACTCAGGATGGTTAAGAAAAAG

>UN00376

CCCATAACACTATAGCGAAACTAAAAACATCAGCCTTGTGATCATAAGGCTTGTGTTCAATGACCTCCGGAGCCATCCAACGGTATGTTCCTGTTTCTGCAGTCATCACTCCAGTTTGAGCTTTAACACGTGCAACACCAAAATCCGCAACTTTGACAACCTCATTTTCATCCATCAGAAGATTGGCAGCCTTCAGGTCTCTATGGATAATATTGTTTTGGTGCAAGTAGTTCATACCCTTGGAAACGTCAGTTGCTACTCTGAGCAATGCTGGAAGCTTGAAAACACCTTTCTGTTTATGAAGAAAGTCGTATACACTTCCACCCGACATGAATTCTGTCACGATACATAGGCTAGGGAGGGCGGGTGC

>UN00377

TATGCATAGGTCGCCGACTGCTGCATTAGGAGTTATTGGATCTGGTGGAGCTGCTTCAGATTTTGGGTTGTGTCGGATATTTGGATACCCAGCATTTGGTTACATCGTGGATGTTGAGATTAGATCCGATTGCGAGTTTGGGGTTCGGTAGGCTCAGTTGATTATCGTTTGGGAGCTTCTGATTTTGTTCTGAGCAGGTATGTTACTTTTGACTCTCGGCAGGTGGAGAGTGTTGATCAGGT

>UN00378

AAGTTCTAGAAAGCTATGTTGCCCGCCAAAAACTAGGATATAATCTAGTAACAAAATTTGGCCAAGGATTGTGTAAGACACAAAAGTTCACCACAAATACACAACTAATAAACATGAAAATACTAATATCATACAGCTTACAGCATCTTTCCATACAAGGAAAGAGACTGCGTTCTAACCTAACAATGCGTTCGCATTGCTGATTGTCCTGACATACAATTATAATAATAAGATGTATTAAATTACAATAGATTTACAACAGACAAATAAACATCTGCTGAGGCTTATCTTAATCCTTGTTGCGAGATGAGTGTTCTTTTCTTTGCCCACCAAGAATACGAGAAATCTGCAGGCCTCTACTGAAAGCTTGGTTGAAGAGCATCCTGGTTTGGAATTCAGAAACAAATGGGAACCACGCCAAAAATGCAATCGGAGTGAAAAGGAGTAATCCTATTATGATTTCATATCCACGTGCAAGTGCACGTATCGATCCCCATAACCCAGCTCTGCGAACAACTCGCTTAAGAGCCTGTGCAATCAAGAGTATACCCCAACCAGTCGGCATGAATGCAAGGATGCATACAATTATGTCTTGAAGTGTCATACCAGGGATGGCTATCAAAGTAATCAAAATGGACGCAAATGTCACAAAGATCAGAAATTTGATCAGCCGAAAAACAAGCTGAAAATTTGCGCTGAATCTCCTCCTTCCTACAGATACCGTCTTCATGACAAGCAGTACAGCAAATATCACAAGCCAAGAAATTCCATAAACCAGGATGCTTTTAGTCTTTCTTGTTATGTTCAAGTGATAGACAAGCCCATACTGAT

>UN00379

TAAGACCCTATACAATGTTGATTGTGTTCAAAGAATCCTTGAGCGCTTCTTGGCTATGGATCAGGCGACAGGTGGGGCTTCACCTTGTTTGGTTGAAGATGAACAGATGATGGCATCGCCGTCTTTGACGCCGCTGACTACTGTTGCCAAGCTTATTGATGGATATCTTGCTGAGGTTGCTTCGGATGTTAATCTTAAGTTGTACAAAGTTTCAGGCCCTGGCTGCTGCGGTTCCTGATTATGCTCGACCACTGGATGATGGACTGTATCGTGCAATCGATATATATTTGAAGGCACACCCGTGGCTATCAGAATCCGAGAGAGAACAGCTCTGCCGACTTATGGACTGCCAAAAGCTCTCTCTCGAGGCCTGCACCCACGCCGCTCAAAACGAGAGGCTCCCTCTCAGAATCATAGTCCAAGTCCTTTTC

>UN00380

AGGGTTTACGTAAAGAATTAGGTTATGGGTAAGTTTCAGGCTCCCGCAGAGGGAAGCTACAATCTGAGTTCTTTCTGTTTGTGTGATGCTTGGATTGGTTGTGATCGGAAAACGAATGTAAAACTTAAAGTTTTGAAAAGGAGTAGGGCGGGAACTAGAGGACATGTGGCTGAAGAAGGGCCGCTGGCAGAGGAGGGTATCGAAGAGGAAGAAGACGAGGAAGAAGAGGAATATGATGATTATGAGAGTGAGTATAGTGATGATGAGGAGGAAGATAAAAAGAAGAAGGGTAAGGTCGCCAATGGTGTTGGTCACGAGGAAGGAGATTCTAGTGATGAAGGATCTGAAAGTGATGAGTGAATGCATTGGCTGTTGGGAGAAATTTGTGACTTGTTTTCTCTGGTTGGAGGTCCACCCACCTGGAATTTCCGTTTTAGAAAACTGTATCGTTGAGGTACTGGATCAAATTTTGTGGGCTGGGTTGGAATTGAATTATACAAAACATGTAATCGTAAGCGTTTTAGACAGAGAGATGGAGAGACCCATAATGAGTTTATATCGGCCT

>UN00381

AATGTAGATGCATGTCTCTATATAAAAAAATAATTAACAAATTTAAAATCATGTAGGGGAGCATGCCCCCACTCTTCTCTTAATGGATCCGTCCTTATGTTAGATGGATCTTCAAGATAACCTCACTTACACCGTGCATGTCTAATTGGATGTCCTCTTCCACCATCATCAAGGCTCGATCATGGTATCCATGAGGCTCTCAAACTTGCAGTTACATTCAAATTAAGGCTCCAGTGCT

>UN00382

TTTTTATTTTACTTTTTAAGGTTTAAATAGACCAGTCCTGTATCTCATTATATTTTTTGGTTGCTCATGCCAGGTACATTTTCCTTTATGAAACAATCACAAATTCAAAGTTTGAGACACCAAGCATAGAGGTAGAAATTTGTGACCTACTTCAGCATTCCAGTAATCTTATCAATACAACTAACTTAATTGTTTCATTTTACAGGAGCCTATACATGATCGCATATCTCGAAATATCTCACAGGCCTTGTCAAACTTGTAGATTAGACG

>UN00383

ATCGTACAGAAAGTTTGCCAAACTTCCAGGAGAAATATAATCTGACAAAATAAGCTTTTCATGCTGCGTTGGGCCCCAATAATATCCTCTCAACCCCACAACATTTGGATGTCTAATATTCGCAAATTTCTTCGCCTCTTTCGCAAATTCCTTCTTCGGCTTTGCCACTCCTTCTCTTAGCCACTTCACAGTCAGGAAAAACCCCCGTTATCCCAAAGTAGCTCGATATGATGTTCCATGACTACTCCTCCCTAAGACCTCAGCTGGTGCCCTAGATAACTCCTCTGGAGTCAAAGTAATCGTCTCATCCAAGAAATATAATTCCCCCGCTAAACTATCTGGAGATCTAACATCTAATTTAGATAGGTTTTCTTGTGG

>UN00384

ACGAATTTTTTTAGGATTTTTTGGGGGGTTTATTTAGGGTTATAGTGGAGGCCTTGAAGATGAATAGAGTGCAGAAGTTGTTGACAGCTCTGGAGCCCTTGGTTCGAAGAGTTCTAAAAATGCAAGGAGGACAATTTTTCGATGATACTTTTTGATGCCTAAAGGCTTCGATATCTCTTATGAAGGTGACCGAAGAGATGAAGAATGGGCAATAGCACTTGAAAAGTATGTTAAAGACTATCCAGATGTAAAAAATTCAGGTAATATTTTAATGTCTGATCTGTAACATTTTGTTCCATATCATTTTAGGGATTCAAATTGGAATATTTTGATATTTTGTATATAGCGAAGGGCCATGTGTTTATTTTTCGCACAGGGCCCCTAAAATCATAGAACCGGCCCCGGTAACCACTTCGATGATGAGCAGCTGCCTGGTGGAGGCGACTTATAGCCGTCTGGTTTGGGTTTGACAGAAGCATA

>UN00385

AAGAAATACAAGTTAGACAAAAGGACTTTAGCGTCCAGGTGGGGTTGTATGCACTCAGGCAGAGAGCATATGGCTTCACATGCACATCATCGAAGATATCGTTTCCGTCTGTCGCCAGGTCTTCAAAGGATCTGTGAACTATGCATGGACCACTGTACCAACATATCCCAGTGGAGTTATTGGCTTCATGCTTTAGCTCAACTGAGGGGCCAGCAGTGGACTTTAAGCATCCTGTGTACCGCATTGACGAGAACTCTAAAACGAAAGGGCCACTCAAATTTTACAATCCAGAGATCCATTCTGCTTCATTTTGCTTGCCTTCGTTTGCCAAGAAAGTTATTGATTCTAAGTCCAGCTGATAAACTTCTGAAGAATGAGAAGAGGGCAGTTTAGTTCTCAAGTGCGGAGGAGGCCAAAGCTGCGGATGAAATTAAAATTCAATCAATTTTCATCCATTTCCCCCTCTCATGCGTGGGAGGAGTCCAATTTGGAGCATGTAGGAAGAGTAAAAATAAACAATGCCGATGGGTTTGGCTTCTAGATATTTGGTATTATCTGTGTGCGTGGAGAGCCTGCAGTGAACTCTTGTGTCATTTAAGGAACAAATTAAAATTAAAATAAACAATAAGAAATTTAACGAAAGTAAACGT

>UN00386

TTGTAGTGGATGTGCTGTGAATAAATAACTAAGAATAGGACAATACAGTGTTGTTGTTGTTGAAGGTGGTAAGAAATCTGTTAAGAGGTATGGAAAGCTGATGCTGAAGAGGATAAACTGGGCAGCTGCTGTGAGCAATGATGATGATGGAGATGAGAATGCTGATTCTGTAGTCAATAGTTGCTCTTTGGTTTGGCAAGGGAGTGTTGCGAAGCCTAGCTTTCATAGATTTCTTGTTCATCAGTGTAGAAGTGAAGCTGCTGCTAGGAAAATTTTTGCAGATGCTGGAGTTCCACACTATTGGGATCTTGCAATTAATTTTCTGATGATCTTGACGCTTAAAAGCTTGCAATATTG

>UN00387

CACAAACACACACTGTAGCAATAGCTAGCATTGAGTGGATATATGGCCGGTGAAGAAATCCCCCATTACGGTGTCTTCGCTTGAACCTATGGGCATCTAAGCGAGCACACTGAGCACAGGATCTCATTGTTGTGATCCTGAAGCTAAGATGATGATCCTCCAGTGATATTGATATCTGGCCACTTTTCTTGTCTGCAAGCTTTCTGGCCACCAATTCATTGTATGAATGTTTGTTTCTCGCGGATGCATACATGTAAAGGGGAATTAAGAAACC

>UN00388

CTTATTGTAAAATTAACTCCGATAACAATCAACAATGTTCAGAAGTAAACAACAAAACAGATCGAAAACAACAACAATAGTAATAGTAATAAAGAAGAAAGCACTAAACTAAGCTTCATAAGAAGCTGCACCAGGTTCAAAGGATATATAGACCCCGCAGAACTCATTCCACCCAGCGTTCTTGATACCCAGATATCAACGCCATACAGAAGCTCAGAGCAAATGTAGGGAGATGCACATGATTATAGAACCAGACAATAACTTAGTTAAGAAAAATAAATAAAGAAAAGTAAAAACCAAAAAAATTAAAAAATAAAAACAAAAAAAC

>UN00389

AACAAAAAATAGCCCCAAAGCATGCTGTCATAAAAATAAAATAAAGAAGTCTTTTTTAATATCAAGTACAAGACAAAAGGATCAGAGGATGACAATATATGGCCCCAAAATATATAATCTTAAATATTAATGGGCGTTAACAAGCCAACATCCTTCATGGTCTTGTTTCCGTACAGTCTATGCTAGTCTTCAATCATATTGGCGGTTTTTATTCTTCTCAGATCACACAAGGCTGAGCCAATAAACTTTTATGACACTGCATAGATGTTTGAACATCTCCAGCTTGCTCAAAGTTGAATGCGAAAACCTCGCTGGTAGGACAATTGACATACATTAAATGTCAACAAATTTTGTCGTAACGGCTCAATAATGATTAACGAATCGGAAACAAAAGGAATAGAAATTTAAGGAAAAAATAACAAAAATAAAAACGAAAA

>UN00390

GTTAAGTCTACACCAAAAGTCTATTGATGTTGTATCAAATTTTTATTTTTTTGCAATATTTTTTAAAAAAATGCACTTGTCTTCAATACATAGCATATATTACAACCAAACTCACGTCATCAGGATCAAGTAATAATGTTTCTCACAATAATCCAGTCATTCCTCTCATTCCACCTATAACCTATCAAAATTCAAAGATTTGAGATCAATTAACGTTATCCGCACACTATCGGCTATGTGCTAGTAATAGGTGTGTTGCCCACATAGCCACATGGGAAACATAAAATTGACATATCCATAATTTGTTAAGATGAGCACCATATCCACAGTGATGTTGCCAAGTTGAACTTTTCCATGTTTTGATTTTAGA

>UN00391

GGAGTCTAGACAATGCGTGCGAGCCAACGGGCGTTGAAACCCGGAAGGCGCAAGGAAGCTGATGTGCGGGATCCCCCTCGCGGGGTGCACCGCCGACCGACCCCGATCTTCTGTGAAGGGTTCGAGTGGGAGCATGCCTGTCGGGACCCGAAGATTGGTGAACTATGCCTGAGCGGGGCGAAGCCAGAGGAAACTCTGGTGGAGGCCCGCAGCGATTACTGACGTGCAAACCCCCACACCCAGCGGTATCAATTCCTCCT

>UN00392

TTTCTTTGTTCTAACTTTTGTATTTCGAATTATTCGTCAAATATTCGATATGTATTCGCTAATAATTCAAAAATCATAAAAAAATAAATATAATCAGTAAACAAAAAGGTTTTTTTACCTTTCCAGTGACAAAAAGCAGAGAATCACGGTACGAAATCTCCAAGATCTGCAGAGAACGAGGGAAGTGGGCGTCGTCGCGGGGAGGAGGTCGTCGGAGAGAGAGGCGCGCAGGTCGTCGGAGAGGGA

>UN00393

CTAGGTTTTTTACGTTGGAATGGTGCGACTGTGGAATTGGAAGGCATCAGTGACGTTGAGTATGTTGCTGATGAGACTCCTATGGTTAGTTATGTAAATGTTCATGCGATACTCGATCCGCGAAGGTCCAAGGCTAAGGCTGCAGCAAGCAGCGATGTGGGTAATTCACAGGGTCCAAGGGTGATTGTTGTAGGGCCGACTGATTCAGGAAAGAGTAGCCTGTGTAGGATGCTCTTGAGCTGGGCTAGTAAGTAGGGTTGGAAACCAACATTCGTGGATTTGGATATTGGTCAGGGGTCGATCACTATTCCTGGATGTATAGCTGCTACTCCCATCGAAATGCCTATAGACGCAGTCGAAGGGA

>UN00394

GTACTAATTGGTGATCAGAGTCAGAAAAATGAGAACTCTGCCGCCTATAAAATCAAGCCGAAGTTAGTAGCTGATCTCTATGGGAAGTGGATTATGCCACTGACAAAGGAAGTGCAGGTTGCATATTTGCTAAGAAGATTGGATTGAAAAATTATGCAGATATATTGGTGAATGCAATGCATTTATAAGATTGGGGATGGTTATGTTGCTCTCATATGTTCATACACTTGGTTAAAGTTAGGATCTGAGTCAAGATTAGATTGATCTTGGGCTGTTGTTGGATCCGGCATATCTGACAATTTCATGTTAAAAGAAGAAAAGGATAATGAAGGTGTGAATTGAAGCCAATACTGGATTAGCTATGAAAATGAATGGAAATAGTTTTTTTTTCCTGTCTATGTATGAGATTTCACGTGTATACGGGATGATTATTACATAATTCACAAGTCTACGTATGAGATTTCAACGTAAGT

>UN00395

TTATTCTGTACTAGAAGCTTAGGACTTAGACTAAGCTCTGAGAGAGCGCAGAATGAAGGTGACTCATGTGGATTTCAAGAAGGCGAAAGAGAAAGTCATGTTTAAGAAGAAGGAAGGGGTGCCTGAGGGACTTTATATGTAAGAAAGAGCAGCCTCTATAAGGAACAGGTGCTTAAACTGAAGACTTTGAGGCGCACATACTACATACATGCGTATGTTTTTACTCAGGGACCAGTATTAATTGTTAAAGTTGGCATGTAAGAATCGACTTGCTCTGCTGGGTCACTTTCCTTAGTGTGGTTGCCTTTCTTTTCCTGTACCGCTACGAGAGAATGAAAACAGGGATGTTGATTTAAATGCTTTATCATTATTCTGCTGTCATTAGAAGACCTTTTGCTTGAC

>UN00396

GAATACTAGAAGACTCCGACCATGACACTGCCTTCACCCCGCTCGAGCCGCCGACGTCGCACCAAATCCACCCTCCAGCAGATCCGTCATGACTCGACCGTTCAGATCCGTCGTACCGCACCGCATCGGCGTCGTCGCATCGTCGACAGGCCCCATTGGCCTCACAGATTTAGAGGAAGAACAGGTTCAGCTGGGAGATCATCAGGAGCCAGAGGATTCTCAACATGAGTACCATGTAGA

>UN00397

CAATGGTGGTGCTTATTCTTCTTCTTTCTTCTTCTGATTTGAGGAATGAGAACCCAAGGGTTAGGCACCAGCATAGTCAGTCGATGGATGGGTCGACGGAGTTTCTTGGGGCGAATTCGGAAGGGCCAACCTCTTTGGCAGAGGCAAAGAAAGACTATGTCCGCAGCGAAGCCTTGCAGAACTCGCGCTTGTGGATCCAAAAAAGGCCAAGAGAATTTGGGCAAACAGGCAGTCGGCTACAAGGTCAAAAGAAAGGAAGATGCGATACATTGCAGAGCTCGAGCGGAAGTTGCGAACACTGCAAATCGAAGCAACGACTTTTTCGGCTCAGTTGTCAATGTTACAGAGAGATACTACTGCTCTGACTGCTGAAATACGAGTT

>UN00398

GAAGGAGAACACACAGGTCCCATCAACATAGGGAACCCAGGTGAATTTACGATGATGGAACTCGCGGAAACTGTGAAAGAGTTGATCAACCCTGGGATTACTTTGTAAAGAAGTGGAAAATACACCCGATGATCCTCGCCAGAGGAAACCAGACATCACAAAAGCCAAGGAATTGCTCGGTTGGGAGCCAAAGGTGACTTTGCGCGAGGGTTTGCCTCTCATGGAGGAGGATTTCCGTCAGAGGCTTGGTGTTGCCAAGAAAGCATGATGAACATTTTGAAGGATAATTATACATCAATTTGAAGATTTGGTTTTTGGGATTTATATAATAAGTAGTACATCTTTTTACAAGTTCATGCCCTGAAAAGTTCCGGAATAAATTGTTGTTTTAGAATGAGTGGGGGCAAATTGCAAGGTCTCGTTTGTTTCAAGTCATCAAATATGATGAACAATAGGATAGTGCAAGTTTTGTTTTAATGTCTAAAGGCATTGATTATTGATAAGAGATTGCTATTTTTCTTGATATAAGAGATAATTTCTTTTTCCTTTTACT

>UN00399

CACTGTTATCTCTCCAACTGCGCTGACGGGCTCATCGACGCGGTCATCGCGTCGACGGCGATCACCATATCGAACAATTACTTTACTCATCATAATGAGGTGATGCTCTTGGGGCACAGTGATTCTTATGTGAGGGATAAGGCGATGCAAGTGACAATAGCTTTTTAACCATTTTGGTGTGGGCCTGATCCAGAGAATGCCGAGATGCCGCCATGGATATTTTCATGTCGTTAACAATGACTACACCCACTGGGAGATGTACGCGATCGGAGGCAGTGCTAACCCGACGATCAACAGTCAGGGTAACCGATACCTTGCTCCGGCGAA

>UN00400

GAAGAAGAAATAGTCAATATCTACTTTAAGTTTTCATAATTTAATATTTCATGATCTTTGAACTGCAGTCCTCCATGTTAGGATCACTTGTGGGTACATGCTTCCCTAGTGTTCATACATAGAAGCAAAAAAAAGTGCCCCGGTTACGCATTGGACTAAAAAAGACTGGCAGCACTCCCTGCTCATAGTTGGGGGCCCCTATGAGGCTTCAATAATACTAGGAAATTTCAATGCAGAAACAGATAGCTGCGAAAAAAATCTTCGCTCAGAACTCCTGCAACCGG

>UN00401

TCCAACGCCCACAACATCACCAAGATCCTCGCCAGCCACCTCCGACTTCTCCACCTTCAACCACTACCTCACCGAGACCCGGCTCGCGGCGGAGATCAACCGGCGCCTGACCATCACCGTCCTGGCCCTCGACAACTCCGCCATGTCGTCCCTCCTCGACCACCACTACTCCCTCCCCACCCTCCGCAACATCCTCTCCCTCCACGTCCTCGTCGACTACTACGGCGCCAAGAAGCTCCACCAGCTCACCAGCGGCACCGCCCTCTCCTCCTCCTTCTTCCAGTCCACCGGCGCGGCCCCGGCACCACCGGATTCGTCAACATCACCGACCACTCCAAGGGCCACGTCACCTTCTCCGCCGAAGACTCCTCCGACGACTCCGCCTCCTCCCCCGCCGCCTCCTTCGTCAAGTCCGTCAAGGAGATGCCCTACAACATCTCCATCGTCCAG

>UN00402

GTACAACAAACTTAATATTTTCAAGAATAAAATGTCAAATGCAATATAGTTATCCAATACAAGTTGCCTGACATATCACAAGTTTTCATTTCAAGTGATCAAGATATTAAACATATGAATAACTTTGTTATGTCAACTGGTCAAGTACTTTTTTTATGTCACGTCATTGAAAACCATCATTTCAATTGACGGAAAGCATTAGAAGTGATTAATGATCCAATTAGTATTTAATTACATGATTAGTTATCCAAGCAA

>UN00403

GTTAAAGTATTTCTATTAGTGAGTACTCTATTATCGATCTTTCCTTCTTCTTCTTTTTTTTTTTTTGTTTGTATCCTATCAGGAGAGGCAAAAGCCATCCTTGAAAAGTCACAAGCAACTGCAGAAGGACTAAAAAAATTGTCTGAGGCCATGAAAACAAATGGAGGTTCTGAGGCAGCAAGTTTGACCGTTGCTCAGGAATACATCAAAGCATTTGGACACATTGCTAAAGAGGGAACTACACTATTGCTACCGAGTGGTTCTGAAAATTCTTCTTCCATTATAGCTCAGGCTCTATCCATATTTAGGACGCTCAGTGGCAACATGTCTTTTGGTGGCCCTAGTGCAAATCCTCAATCAGCGTTGGGCAATAATAACAAAACTAATGCTTCCGAACGTCTGAAAGAACTTGAGTCTCCAACTAAAGTTGGTGATCTGGATCGACAGGAAGAGCTTGAAGTTCCGGACTCTGAGATATTATCTGACTCGAGTGTTAGTTCTGATCCAAATGTAAAAGTGTTTTCTCTCCAGAGTCAAGAAAAGTAACAAAAGTAGAATCCAACTTGCATGTACACTTACAGAAAGGTAGTACACATTCATTTTCCCGAAAATTTGATCATACCAGACTTGCAAGAATGCGTACTGCATTTGTCTCAAGGGGACTTGAAAGATTTTTGACATTTTTATATGCTTCAGTTGTTAAGAAGATGAAGCATTCATAGAGATTCTTTCACGTTTGGCTTCCCTTGTTAAACGTCTGAGGCTACATTCACCCATATCTCAGCCCTTTTTAAGCCTCGTTCTATAATCTGAGATGAATTCTGTATTTTCTGTTCTTTAAATTTGAGTTTTTTTTCTATCTTTCGAAGACATATCTGAAACAGCTTCAAAATGAGTTGCTTCTGAAACCATCTTTGTTCAGCTTTACAGTATTAATCTTTGTATAAGCTGATTGATCGCTTGTCTTATTGTTCGCT

>UN00404

TCTCTCTGCTAAAGGAGTGAGCCTAAAGAAGAAAAGAGAGGAAACAAGTAGTTGAGATGGGCATTAATTTGTCGTACATCAGTTCTTTCTGTGATGCAGGAAATTTGGTGATTTGACAATGTATAGCGTGTAACTTGAGTTTTCGGACACAGTTTTTTGGGCTTTGCATGTTTCTCAGAGAAATTTCCTACAATATTTTTGTAGCTGTGGTCTTCAAGAGGCTTCACATATTTATCATACTTTTGCCATGCATCATCTTACTGGGTGCTGAGAGTGGCTAGAGAGATCTTTGTATGAGAAGAGATTTGAAATATAGATTATTTATACTATGAATCGCATTTTGTCTGAACC

>UN00405

CTGGAAAAAAGGGCCTAAGAAGCATCGCCATTTATCTCATATTGACAGAAGATGAATATGGGATACATTCAGCAGGTCAATACCTTGAATTGGCTGATTATGTTGTAATAAATCATATTTCTGTCTTTTTAACTAGAAAATTATTGTGTTCATTGAGTGTTCTGTGTAGCACTATGTTCATTGAGTGTTCTGTGTAGCTCTATGTTCATATATATAATTCAAATGCATTTTAGTTTACAAAAAAAAA

>UN00406

GTTAAATTAGTAAGTATTTCGTACTAAGGTAACTTGCAAGCATGTGGAGGAAAAGGCAAAGGGAACAGAATATATAATCGTTTCAGATAGTGAAGAAAGCGATGAAGGTGAGAGACCTTTCAGGTCAAGGTTGTCTCTCATGAGAAAGCCAACGGTGAAAAGTCTGCACAAACCGTGAAAGAAAATCTGTAGCTAAAGCGCAATCTCCTTATTGCATGAATTTACATATGTAATTTGAATATGCAGCTCTTAACTGGACAACAAATCTGCATTTTACGGGGCTTTTCTCCTGAAAACGTTTATGACCTCTGATGATATTTTCCGGCTAGGCTGGAGTGCCATGATTTGATGGTGTTTGAATTCATTCTTTG

>UN00407

CTACGATCTCTACTGTGACCACGGCCATTACCTTCATCGTCGGTAGACAGTGACTGAAAATGCTAAGGGTGGTGTTGATTTGTAAATAGTGAAAACGGGTAGGATTTGAAGAGCTACTGATTAATTATTATGTGGCACTTTGGGGATGGAGCAATTTGCATCGATCAATTGCGATGGGCATTTAAATTTAGGACGGGGAGTGGCTACTGACCAATCGAGTTGTTGTTGGTTTTAAAATGATGAGAGTTTATGTTTATTTATTTTAGATATATATATGACTGTTAACTTTTTTTTTTTTTGGGAATCTCTTTTTTATTAGACTTACAAAATTT

>UN00408

TTCAGGAGGTTCAGGGATATTAGACTCCACGGGGAGTACTATTACGATGACAGAGCAGATACGACATTTTAGAACAATACAATCAAATCTTTCCATGAGAATGACAATTGATGCAAAAAACCTTATGCTCTCTAAATCCATATTTCTCATTAGCACTGGAGGCAATGACATATTTGGCTACTTCTCACAAAACAAGTTTCCTAATGCTACTCAAAAACAGCTATTCATAGCTACTCTTGTCTCAGACTTGTCAGGGCCATCTGGAGGAAATGTACAACCTTGGTGCAAGGAAATTCGCGATAGTTGATGTCCCACCTATTGGATGTTGTCCGTATCCGAGAAGCCTTAACCCTACAGGTGGTTGCATCGAGGCATTAAATGATTTGGCTCTAGGATTCAATAAGGCTATAAAAGTTCTCTTACAGAACCTGAGTTTGAACCTTAGAGGGATGAAGTATTCAATTGGAAGTTCTTATGAGGTCGTTTCGAACATAATTAAGAAGCCATATGCACTTGGTTTCAAGGAGGTGAAGACTGCATGCTGTGGATCTGGGAAATTTAACGGCGAGGGTGCATGCATGCCCAATGCTACTTATTGTTCTGACCGCCGAGGTTACCTATTTTGGGACATGCTACATCCAACACATGCTACATCCAAGTTAGCAGGCTTTGCGATCTACCATGGGTCACTGCAGTTTGCTGCACCTATTAATATTAGGCAGTTGGTTGAGGCTAGTAACTGAAGAAGGTAGCTCATGTACTTAAACATGTACTTAGTGCTGTTTGTGGATTTTGGGTGAGGTAGTGCTTGTTGTATTTCTGTAAAAAAAGATTTTAGAGATTACTTATTGTATTTTAGACTTTATTGTTGTTGATATGTATGTGTTATTTTTTTCATTTCTTGCTGAAGTAAGACC

>UN00409

AGTTTTAAAATTTTTTTGGTTCTTATTTCTTACTATTTTCTATTAAATTTTAATTACAAAGTCGATGGATTTAAGTTACTCCACTCTCCAAACACATATCACGGCCATCGGCTCTTTTCTCTTGCAAAGATCCGTGCAGAACTTTGGTCGATAAGGTATAAGGTCTCGTATACAATTGAACTAGCAAGAAAAGGTTCAGTACATAGCGAGTTCAGAAAGAATATATCGCGAAACATTTAGATATCTCTACAAACAATATTACACGTTATTCTCATCCCGCTATAGTATGTACATGGTTCTTCGACTTGGTAGCAGCAATGCCATCCTCAAGCATTGAGCGTTTCTTTTCATCGGTTCTACTTCAGAACAGTTCCTACCTCTACTGCGTACCAGCATAATCAATCATTATACACCTCAGACAGTATTTAGAGTATCAGCAAGACATCGTAGTATGCATCCCAATCCTGCTCTCTCCGTCAACTTCTCATGAATCCTCAAGCACTGATCGCA

>UN00410

CTAAGATCAGTCAATCCTTCAACAAGCAAAGTAAACCCAGCAGAAAAATATATCCAGATTGTGTCTGTTGACAACCATGAGTTCTGGTTCATGGGTTTTGTGAACTACGACAGTGCCGTGAAGAGCCTACAGGAAGTCTTGCCTGGTGCTCTCGACTTGCAACCTTAGAATTGGTGTGGCATGTGAATTCGCTGCTGCTGCTGCTGCTGTTTCCTTAAGTCTCATTATTCACTTGCTTGGTTACCTAATTGTGCATTATGTTATGTGAGTGATGTTGATGATGGAAACATTCTTCTTTGTATGAGTTGATGATCCTTCTCTCATACTCGTAAAATTGGTGAAGTCCTGAAAATCAAATCTGTGTAATACTGGGCGTGAAATTGCTCTCAAATTTGTTTTTATATGCTGCAGTCCTTTTAATGACCGTATTCACTTTTGGCAAACGATGCATCACTTGATCCTGATTGTCGGCCTACTCTTTTTGATGATGGGGAGGACAGATGTTTGGAACC

>UN00411

AAAATAAAACAAAAGAAAAAACAAAATTTTAAATAAAAATTAACTAAGAATAAAATAATACCTAAACCAAAAGTTACTAGACCGAACAATTACAAGGTTGTTGAAACTAACACCACAACAATAAAGAGTGAAACCAAAGATACAGCACGGAATGGAAGTAGAGAGTATAAGAGAGACCATCCAAGTCCGCATTAGCCACATGCCATTATCATACTTAACATATGTCGATATGTCCATAACATTCTCATCATCTATACTAGGATGAGTACTACTTGGCCACAACGCATGCATAGAGATAAAGCAGTTCTCCATTTAGCTCTCCAACTCCTTGAGGAAGTCTGCGTGGTCCACCAACACGATTTTCCAGCCATCAGGATCGAGAAATGAGGTAATCTTGGTATTGATTCCTGGAATAGGTCCTGGCTGGCGTGTGATCTTTCCTTCCTAAGTCTTCTTTGGTTGTACCTAGTAA

>UN00412

TAGTCTACGTATTAATACCAACAGAATTTACACCAAATGATGAACAACTATTATTAGTTTCCATGGTTGAAGTACTAGGGGCGCAAGAAAAGAAATGCAGAGGGTGGAACTGTGCACTGTCAGCAATGAACTTATGATAATAAGCTTCTGCCCCCACATCCACCACTCGTTGTTCTATCAATCTAGGGATTTCTATTCTTCTTCCTTCTTCGGTAACTGATGTATCAAGTATATCTCTAACAAATTTCTTGGCTTCCACTACATCTTGATTAGAGAACATCGGATTTGCGGATCCTGGTAGGAGAGCATTTTCAGAAATGTAGGTGTTGTTGGCAAAGAAC

>UN00413

CAGAATTCACAGATATCAACCGTGGAATGGAGAAGACTGGTACCTTCACTATGAAAAGGAAAAAACTCTTTCAGTTAGTGGGCAAGGCAAACTCAAATCTTGCAGATGTCATTCTTAAACTCGGGCTCTTTGAGAGATCTGACATCGCTTGGAAAAACGCAAACTATGCTCAAATCTGGGAGTAATCTTCGGGAATGAATATGAACTAACTCAAAGATTTGGCAGTCTTGATTTTAAGTTGAAGTTCGTTGAGCATAATATTAGATTCCTCCAGGAGATTCTCCAAAATAGAAAATCTGACTTTTTGGAATGGCTTATTATTATTTTGATAAGCGTGGAGATCCTGATCTCTGTTTTTAACATTGTTCGTGAATCATCAATCACTTCCTTATAGTATGCAGA

>UN00414

TTGATCAGGCAACAGAGGAAGAAGTATTCAATGGTTCTAGAAGACGACAATTTACTAGAGGTACAAAGTGCACCTGAACTGCCTGATTCCATCAAAGGGCGGGTATCAGTGCATGATAGGGTGAGAGTACCGGTCTCCTATGATGACTTGTTTGAAGAGGGTGAGATACATGAATGAATTTTATCCTTTGAAATCAATGTTTTGAGCTGCCAAAATATTGGTGTCAAGCAATCATTGGTCCAGATGAAAGTTCTTTTTTGAGAGCGATGTAAAAAAAAAATCGACTGTCTTATGTATAGATGGTTTTGTCATACCCTGAAGTTGAGTTTTATTATTAGACTGCAAGAAAGGAGAATGAATTGAGTTTTTTACCCCTTTCTATATTTGCTTATTTCCCTCCTGTAGGATGCAAAAAATTCACATTTGA

>UN00415

GAGGTGATAATAATTATAAAAGCTACGAGGAAACCTTTCACAAATCAGGAGAACAAGATAACTAGAAGCCATCACTAGGGATTAGGTTTAACAAAGCGCAACATTCTTTAACAATGTAAAACACAAAATATTACCAAATGATTGTCTTACTAACAAAAACTTCTTAATTGTCTATAGAATCCTACAGGCAAAAGTCTCCCAACAAGATCAAACCCCGGTAGGATATTAAACCATCAATGGTGTGGAGAGTTTTTGAAGCAAAGAGTTCATCATTTCTTCTTGTCGGACACACCAGCAGACCTCATCTCCTCTCTCTTCTTCTTGGCCCTCTTGTGAGTTCCCAACTTTCTCTTTGACAAACTTTACAAGTAGAACAAACGAACTTAAATAACCTTTAAACCAAAAACCTTAAACAAAAAATAAAAAACTAAAAAC

>UN00416

ATTTTTTTTTAAGGTTTTTTTCGTTTCGTCAAATTTTACGAAGTCCTTGCAAAGATTTCTGTATTAAGTTCTTTACAGGGCCCCGTAAGACGGCACCTAATACTTCACACTATCTGAAGGAATTCACCAATGATTAACATTTAACTGCAAGATTACATAACACAACTTTAAAAAAAATTATCGCACAAGAATGATGCGCAATATCAAATTCAACAACTTAAGAGCAACAAATAAACACTTGTTTCAATTAAGAGGCCTGGAATAAGAAGCTACACTGAGGAAAGCTGTTGACAAATCCTGTGACGATATTAGGCGGGAGCACTTTATCCCTCAAAAGGATTTGATGGTGCCTACTTATGAAATCTAATATGGTTGTTTGTATGTCGATTTCCTCAAATGGACCTAAATCAAG

>UN00417

GGTTTGGCTTGGGATGAGACCAAGGCTGCAGGGCGCTCAGTTTGGAAAGAGAATCATGGGTTCTCTACAAACTATCAAAAAGATCCAACCTTTGGATCCCGTTCAAAGGGATGGTGAGGATTGTTCATCTGGGTCTTTGAGGAATTTGATGGCCCTCGGCGGTTTTAAGCGTTTTATGTCCTTTGGAGCTGGAGAGAGTGTCGCAGTTCAGTATGATAAGCCCAGTGACCAGGTTTTAGATTTCCCTGGAGGGAAAGTTATGTTCGACTCTGAGATGAGATTCCTCGCTGAGTCGCCGACTGAACGAATCCGCTGCTATAGAGTTCTTGACGACGATGGAGGAACAATTTCTGGCAGTGTTTTTCAAGAGGTAAACAAGGAGCTTGCAGTAAAAATGTACACTCAGATGGTCACCCTCCAAATCATGGATACTATCTTCTATGAAGCTCAGAGACAAGGAAGAATTTCTTTCTATATGGCCTGCAATGGAGAAGAAGCCCTTAACATTGGAACTGCAGCTGCATTAACCGACGACGATATCGTCTTTCCACAGTATAGGGAACCTGGTGGCCTTTTATGGCGAGGTTTTACTCTGCAAGAGTTTGCAGACCGCAATTTCGGCAATAAAGCAGACTATGGAAAAGGTCGGCAGATGCCAGTTCATTATGGAAGCAATAGACTCAATTACTTCACGATATCGTCCCCTATAGCTACACAGCTCCCTCAAGCTGTTGGAGCTGCTTATTCTCTGAAGATGGACAAGAAAAATGCCTGCAGTGTCACTTACTTTGGTGATGGAGCCACAAGTGAGGGAGACTTCCATGCTGCAATGAACTTCGCTGCTGTGATGGAAGTCCCCATTATATTCTTCTGTCGAAACATGGTTGGGCCATCAGCACTCCTACTTCAGAGCAGTTTCGAAGTGACGGTGTTGTTATCAAAGGTCAAGCTTATGGAATTCGAAGCATTCGTGTCGATGGCAATGATGCTCTTGCTGTTT

>UN00418

GGCATTAATTAATCAAATAACACAAATTATAAGAACATTAACTTACTGCAAAACAACATATTCAGAGAATTATAAAATCTCTTGTATATCCTGATATTAATTTGTCCAGAACAGTATGATCAAAATTATCCGTAAAGCATCCAGAATATAAACTGTAAATACTCATAAAATTGGAATTTAACTGAAAATTGGTTCTATCAAGTCTCCTGACTGAATTTGATGTCTAGCGAAAGAAACCCCTCACCTGGTAACTCTGTATCCCTTTACAGTTTTCAACAATTCTTTTGCAGTGGGGTCGTTTTTGAAGTGATCACGAACAGGCTGTAGTATCTGATTTAGCGCTTTGGAGAGAGCAGGCTTCACATCAGCTGGATGCAATGCACCACTCTCATAGTCTACAACTAGCTCCTCCATACTGGTAAATTTTTTGTTACCCCCATTTTCTGCTTTTCGTACTACCTCGAAGCTTCCAAACCATGGAAAGATGATATATTTGATATATTCTAGACAAGGGTTTCCCTCAACAATATTAGGCGGGCAGTAAGCTTTCTTTATTTTCGAATTCACATCACCCTCTTCGTCCTCCATGAAGATAGAAGAAGATGGATCACTTTTTGACATTTTCTCCTGTCCCTGTTGTAACCCAGGAAGCATATGGTGTGAAAGAATGATTGGTTTGTTTTTCCTTTTAATATCGGTACAATATTCCCTTGCAAGGACGTTGACTTTTCGCTGATCCATGCCTAATTGGCAGATGTCAGCCTTCAAGAAGAAAATGTCAGCACATTGCATGCATGGATAGAAAATTTGGGCAGCAGTCAGTTCATCTGTCTCATTACGACCCATGATCTGGCTACACCTTATTATTCTAGGAAGTGTGTTTCTTCGAGCAATATCCATCACAAGCGGCCAGTACTCATGTGCCCGACCATTAATTTCCTCTGAGGACCATAAAAATTCACCTTATCCAGTTTCATTCCCACGGCTTTCCATATC

>UN00419

GTTTCTTAGTTTTCCTTTTCCGTTCTTGTTCTAGTGTTGTTTGTATGGCGCTTGGAGGAAGCGCGCTCGCTGTTTTCTGGAGGAACAAGCCTATTCCAATCGATGACAAGGATGATTTTAAAGAGTGAACCAAGAATTCATTAACTTTTATTTACTTTTAGCTCGTTTGCTTTGGTGTTGAGCAAACCTTAGTGTTGAATTTCTATTTAGTTTGCATTCTAGTAGTATCTTCTATTTGCTGGTTTGAGGACGCTAATGAACTTAGTTCTTAGCTTATGGAGTTGTACTTCATTAGCTAATTAGGGGGTCCTAGGATTGGAAAAAATAAATCCCTGCGATTTTATTTAGCAGGGCTTTTTAGACCTTTTTATGCAGAATTGATTAGAGAAATATGGGGATTTAAGAAAAAGTTTAATAAATTAAAAGAAACGTAAAGT

>UN00420

CTTGGTCTAATTTATTCCACCAAAGATTTTTCATACAGATCCTGTAAATTATTGCTTCGTTGTCCATACATAGTATACTATGTTTCATATTTATACATAAAAGAAGAGAAAAAGGAAAACATAAAGAAAATGGAAGGCAGACTGATGATGGGGAAAATGAAAGAAAAAGGGGGGGAAACAGAAGAATCACCATTCTGACTCCTGATTTGAGTTGGTATTGCCTTCAGTTCTAACCTCTTGTGGTTCGATACCGGGCACTGGAGATACTTTCCGAGATGATGCTGGGACTGCAGCTGGAGCTACAGAAGGGTTGAAATCCATTGGATGATTATATTTGCAGGAAAGACCATACTTGCAGATCCGATGTCGATTATAAATAGTTAGTTAACTAATTAATTAAGGTTTAAGTAA

>UN00421

TCGTAGTATTTAGGGTTTTTTTCTTTCTTTTTTCTTTTCGTAGAGAATCATCATCCGGTCCGGAGGTTAAAGCGGCGGGATCGCTCCCCGTCCACCTTGACAAAGATCCCCTTCGTCTTGGCCCTCGCGTCGAGCTGCCGCCTACGCCGCCGCCGCCGTCGTTAGGGTTGTTGTACATCTCAGGTACTTAAGAGAGAGAGAAATGGCCATCAAGGTGACTAAGGCCGAGAAGAAACTGGCGTACGACAAGAGGCTATGCAGCCTCTTGGATGAGTACAGCCAGGTTCTGATCGCCGCCGCCGACAACGTGGGGTCCAACCAGCTCCAGAACATCAGGAAGGGGGCTCCGTGGGGATTCAGTGATCCTCATGGGGAAGAACACCCTCATCAGGCGTTGCATCAAGTCTCACGCTGAGAGCACCGGACAAACAAAGAAACTAACCTTAAACCTAACCTAACCCCTTAAGAACTAACGAAA

>UN00422

TAATAATGAGCTAATGACAATGGCGACAAGAAATATAAACACAAGTATAAATAAACGTCAAAGCACACTCAAACACAAGATATGATCACGGAGTTCGGACCTTCCGTCCTACGTCTCCGCTCCCCACTCCTGGGGAGAAATCCACTATCACAACCGAGTTTTCTACGCTCACCCGAATAACATTTACAACCACACTATGTCGGTTTTCAACGATCACTGAACAATGTTCACTCCTCACACTCGGTTTTCTAGTTCACCGAAAAACTTCACAACACACTCGGTTTTCTAGCTCACCTAAAACCTTACACCACTCACAATGTAACAAATATAAATTTGTTCTCTACAATAGATGAGAACACAATTAAGCACAACTCAATGTTACAATCTCTCACAACCCTAATATACTATATATGCAAATACAATAAGCTCAAGTGGGTGTATGTAACAATGAGATAATATAATCAAATCTAAA

>UN00423

GTTCTTATAGCTGTCTTTTTTTTATTTATTATGACTTATCCCTGCCTTCTTTTTATAATAGCATCGAATTCTCCCATTGATGCTGTGCCTGCTGCCGCCGCCTCCTCCGCCTTCTCTTGTCAAGATAATTTCCGCTTCAAAGGGTACTTTTAATCTTCTTGACTGAGCATTAGAATGTCAAGTGGTCAAAGCACACACTGGTGCCATAGATGCATGAGAGCAGTCCATCTCTGCCACCAGGACACTCTTTGTTCGCCC

>UN00424

TTTTTTTCCTTTCTTTCTTAATTGCAGAGTATATAAATTAACAGGACAATCAATGCGAGAACAACAGCGCATGCAAGGCCTATAGCAATTTTTACACCTGTGGATAACCCTTTGCTCCAGAAAAAGGGACATGGTGGCAGAGTTGGTACACCACACAAGCCTTTATTACCTGAAAGACTATACAAAAAAGGTAATAAAACAGAAAAAGAATCACCCAATTTCACTAGCAAATGCAATTATCCAGATATTGATTTCGCACACAATGATTCCATATTTACAGATT

>UN00425

TTAGTCCGTAATTCTAAATTATACTTTTAGTTAAATTCTATTTATTTATAAGCTGAATGCTAAAAAATATTCATTTAGTATTAAATAAACTACCAGATCGAATATGACCGAATATCTAAACGAATATTAACCGAATTGTTTATTAAATATTTGGTTTCGCTTCCACCCCTAGAAACAAGCACTTCATTTTCTTTCCTACCCTATAATAAAGCCTACTACCTACCCTCTTCACGTGATACATCTCATCCACATGTGACTCTATTGTTAACTTCCTTTTTCATTTTGAGGATTTTTTTTTTCAAAAC

>UN00426

TCTGAACTACAGAAAACATATAATTATCATGAAAAAGTGAACTAGTAAATAGAATAAGCATGAAGGCTAGTGAATCACGAAATGTAGAACACTGCATATAAATACAAAAGATGGTGATATGACGAGCTTAGAGAAGAAGTTCACAACGATAGCAGGCATATGATGTCAATAACTCTAATCTACATAGGAAGGAGCAGCAGCAGATGAGACTTCTCGCGATCTGGTTGCTGTACTTTCTACAAATATTTC

>UN00427

GAAAACCAAAACCAAACCTAACCCCTACGACGAATTTACTAAAAAACAAGAAAAATAGTTAGGTACAAGCGCTTCTCTCTCCTCATCATCCCTCTTTCTCTCTCTCCCCAAATCTAGGGCTACAAAGCTAGGGTTTCTCCAATCGAGCTCTCGTCAGCTTGAGATCGCGAACGTTCCCTCGCTCTTCTATGGATCGTTCATCGCCGATCGCGTAACACGGGGGAAGATCCGAGGATGCCGCTACTCCCAAGCAACGCTCAGGCTCAAAATCAGAATAAAGGTGGCAACTTTACTTCCGGTGATCATAGAACCCAAAATTTACTTGCCAGGAAGCAGTGGATTGCACAGCAGAGGAAGTCTTTGCCCATTGCAGCAGTA

>UN00428

TTCAAAAGACCCAGGTCCAATCTCAACAGACCGACGATGTCAGGCCGAGGAAAGGGCGGCAAGGGCCGTGGGGCAAGGGCGGAGCAAAGCGCCACCGCAAAGTCCTCCGCGACAACATCCAGGGCATCACCAAGCCGGCGATCCGCCGCCTCGCCCGCCGAGGCGGAGTCAAGAGGATCAGCGGGTTGATTTACGAGGAAACGAGGGGGGGTTTTTGAAAAATTTTTCCCTCGAGAACGTTGATCCGCGACGCCGTGAAACGTACACCGAGCACGCCAGGAGG

>UN00429

TTTTTTTTAGCAGTTACAGCACAGGAATTCAATAACTTGTTTCAAGAACAGAATCCTACAGAACTGACCTAGAAATACAAATATGCCGTGTCTATGCTCAACTTCCCAACATATGAAGTATTTGGGAAGCAGTCCTAATCAACCGATAATATCTCTTAAACATGTGCCCCATAGTCCCATACAAAATGAATCGAATACATGTAACAAGAACAAGCACAAACTATCAAGATAGCCATGTAGCATCAAGGGGTACAACACACCCCAACTTGTCTCCTACTTTAAACTTGTTCTTTGGTACTTTCGGTAGTCCTTTTTTCTTTGTA

>UN00430

AAATTTAACTAAATCAATTAACTGCACCAAGTTTGATCCAATAATGTTGAGCACAAACAGATACATTTCAGCTCATACGAGAGAGGCCTCACAATATTGCAAATGCCTACAAAGGTTCTCTCTCATGGCTTGGCCAGAAGAATCAACTTGCTGACACATTTGCTCCTTGTAACCAGCATCATGGTGATTTGTGAGGGCAACATCATGGTTCAATTTGTCTCCATTATCGAT

>UN00431

AAAACTATGAATTCTTTTCCAAAATTGGCAGCCAGCTTTTCTACAGTTCATAACCCCGCGCGTAACTTTTACAAAGCTTCAATAAAACATAAACATCGATACTTCTTGTATTATAAAACATTCCAAGTTGTGTGATATAAAGAATAGAACCAAACGAGGAGAGGAGTGGAGATGAACTCATAAAGCTGATGTTCTCCTGTGATCCTAAAATAAGTAAATACTGATACTTTTAACATTCGAACAATGCTCCTAGAAAGTTCGCAGCCTGTGAACCATCAGGTGCTCTAGGCTCTTCAAGAATATCGGCACCAGCAGCTTCTCTTTTGTTTTTCCTCTCCTCGATGACAGATTCGATCGATTCAACGCAGTAATTTCTCTTCACTTGCTTCACTGGCTTCTCTGCCACAGTGAACTTGTTACCCTCCCGGCATCTTTCAGCAAATCCTGCCCAAACTCCAAAATGATGCAGAACAATCGACGCAGCCACAGTCACGTTCAGCGATGCCGTCCCACCCCCATACTGAGGAATATACACGAAGAAGTCACACATTTCGTACTACCTTACTAACCG

>UN00432

CTTAGGTTAGGTAGTAGACGAGTGGAGGAAGCACCCATCGCTCACCAGCAACCTCCGCCATGCGACGCCGGGGCTCGGCATCGCCCTCGTCGCCTTCGGCCTCTACCTCGTCGGCGAGGCCGCCTACAACCGCATGAACCCCTCAGATCCCCGTTCCAACCACTGAGGATCGAAATCCCTCGGTAAAGCTTCGAGCTTGACCTCTAAATCTGCTAATTTCTCTTGGGATTAGGGTTTTTAGTGGTTTGGGGGGGGGGGGGGGTTATTTGTGGGTTTTGGGCTTCGTTGAAGAACTA

>UN00433

GTTTGTATTAGGAGCACAGAAGCCAGTGTATTCTGCTGCAGGTGTATGTTAACTAATCAGACTCTGGAGGTGGCTTTGTTGCGCTATGGGTTCGTGCAAAAGCTTGTTCTCTCTTTATGTCTGGAAGCTAGCGCTTGATTCGTGCTTCTTGTGTCATTATCAGGACTTCTTTTTCTTAGTTAGGCTGATAAAGAAATCTTCTTGTGGACAGCTGATGATCAGTAGCCTTGTAAAAATGTAGCAGTGACATTCATATTTGAGGATGTTGTTATCTCATTATGACAAGGTGTTAGATAGTTTCATATCTAATTTCAGGTGAATACTGATGTATCTGGGCTTGCTTGTTACGCTGCCTTCGGTGCTTTTGAATTTTAGTTTGATACTAGTTGACGAAGTGCTTGCTGTTAG

>UN00434

CCTGGTTTATGACCCTTGAAGGAACAGACTGACATTGCATGGTGAAAAGGTTAAAGGAAATGAAAGAACTAGACGGGGGTTACAACATAGTTGGGCTTTCTCAGGGGAACTTAATAGCCCGTGGGATTATCGAGTTCTGTGAAGGCGGCCCCTAGTTAGAATTTCTAGCTATGTCATGTTTTCTTCTGTTTTCCTTATTACTATCTTTTTTTAGCATCCTTTTCAATGTGGTGTGTCTAGGATGCTAAAGTTGGAAAAGTGTTGTGATGCAGTCTTTTGTGGATTGATAATCAACAGTCGAGCATTTACAAATTTTATGAAAAGATATAATCTTTTGATACTTCATCGCAGATCTACAAATATGAAATCTCTTGAACCATTAGTCATATCATTCATTTGGAAACAAGGCAACCTTGATATAAAATTAACTGAGCATAGTCTATCCATAAAAAAACCTAGTACAAAGAACAATAACTAATTAGTAACAAACGGGTAAATAAAGTAAGAAAGAAAAGAAACC

>UN00435

TTTATTTCCTTTTTTAATTATAATTTTTTCTATCTATTTTTGCAGAAGTTGTTTAGAGCTCCCCGACGCCGTACGAGGAGGCGCTGGACTGCTTATCCTTCCTGGTTGCATCAGCGTTGGCCTAATGTTTGATTAGTTAAAAGATATTGGATCTTGAAGAACCCATTTCGCAGTTGAATGTGATTCCTGTATCTGGTACTAGAGGGAAGAGTTCTACTTGCACTATTCCGGAGTCAATTTTGAGAAATTGCGGGTTTCGCGCTGGATTG

>UN00436

TTTAGGTTAAAAGTTAAAAATTGTAAGTATTAACCTAAGAAAATAAGGGCGATAAATGATTGGTGCTTTGATTCTGCTCGTGCCTGCTTGTTCTCCATGGTGAGATTTGGTGCTCAATACTCATTATGTATTTCATGAATATTTCTACATTTGAGCTGGATCTCAGTTGGAGTAGTGATATTTCTGTTCTGAATGAATATTAGAAAAGATTTCTATTAGCTGTATTTCTTCCGTGATATTCTCCATCCCAATCATTTCATGATTCTACCACTGTCATACTGAGGCCTATACTCCATCTGT

>UN00437

TTGGAATCAATAATGATATGGAGTTTGCCAAGGAGTTGATGAGAGAAGAATCTGTTCTGGTTCTACCAGGTGCTGTAATTGGTTTGAAGAACTGGGTGCGGATATTCTTCGGAGCTCCAAGTAACCTCTTAGCAGAAGCTTGTGACAGGATCAAGTCATTCTGTGAGAGGAGATGGTCACGAAGTTGATCTCTTAGTTAAACCGATAATCATACAAATCATATGTTCATGGATTTTCAATCCAATAATATGTTATAATTCCTCGATAAAAGGCTGAAGTTTCTTTACTCAAATCATACTTGGAGTTTTAAAGAGGAATGTACGTCTAGTAATTTGTCGATCTCTTTAG

>UN00438

CTAGAGTCACGAGAGTAGGATCGATGCCTCCTTTCATAGTATGGAGAGTATGATCGGGATCTGTGCCTTCTTTCGTAACATGGTGAGTATGATCGAGATCTATGCCTCCTGTAGTAAGGCGAGTATGACCGTGATCTATGCCTTCTTCCATAATATGGAGAGTATGATCGGGATCTGTGCCTCCTATAGTAAGGAGAGTATGATCGGGATCTATGCCTCGATCTATAGTAAGGAGAGTATGATCGGGATCTATG

>UN00439

ATCCCTTTCTCTGTCTCTGTCTCTATCCCTGTCCTTCCCATTCCTCTCCTCTCTACCTCTATCTTTATCTCTCCTATGCCGATCCTCCCTCGATCGAGACCGATGCCGAGACGAGCTCCTCTCACGATCGTCTCTAGATTTCTCGGATCGGTGGCGGTCGGATTCGCGCTCAGACTCTTCGTCTTTGTGCCTCGACCTCCGAGTCCGTTCCTTCTCCGGTTTAACGCCGTTCCCGTTCGCAGAGTCTTTCTCCTTCGACGGGAG

>UN00440

GGTTAGATCAAAGGACAAGCATGAGCGAAGTGAGAACCGCGATCGTCATAGCTCCGATTTGAAACGCCACCATAGGTCGAAAAGACGATGACATTTTCATCGCATGATTTTTGTTGTTCGAACCATATTTATGTCTGTATATTATCGAGTTCTTGAACACGCCGTGTTCCCAACTGGTTTAATTGTAAACTTATTTTGCAGAATGATTGTTCAATCAGACATTTGTAGTTGCTCTTGTGTATGTTTGTACCAACGGAAATGTGACATAAAGAGACTGGAAATTTACCCTAGACTAATATAGCAGCTACTTTAGAAAGTTTAAATAAAC

>UN00441

GGGAGTTACATGCATGTGGGATGAGATAGGGAAAGTTTCAGGTAAAGCACAGAAATCTAACCAGAACAGAATTTTCAGGTTTGCGGGATTGCGTGGTGTGTTTGCTGTAAGTATGTGCACTTGGAAGAGGCTAACCATGCCTGTGTGTTCATCTTGTGTATGAATAAGCATCCCTGTTGAATTTATTTTATTTAACCCATGTCATCCAATCATTGATTAGGGTTTCTATATCGCTCATGCGTTT

>UN00442

GTAAGCATGAGAGCCAAATCAAAAATGCAAGCCCCCAAAAGGATCTTTCAATTCATTGACATAGAACTTATCAACAAGAACAAACAAGGGGAAAAGCATGCGAAGCAAATGCTAATAATAATAATACACTTCTCTCCCCCATCAACAAGAACAAGAAAACACATAAGCTCTAGTACCATAAAAATGGAAGTGCCGAAAGCCATCTATATCTAAATTTGTTTCGCCTTCCAGCAACTTCTATAGGAGGTGTACCCAGTTCAAGCGGCAGCAACTGTTTTACCACTGCCAGAGGCTTTAGACCTGGCTTTTGGTTTGGAAGCATTGGATTTCCTCAACATCTGCACGCATTTCTCCTTCAAAGCATCCATCTTTATCTGCTTCTCACGGCACTCCAAGCTGCAGAAGGCTGCATCTCCCCTGTAAATGAACGTATCGATCCCAGGTCCAAGATTCCGCTTACAAATCCCGCAAGCCCTAAGGAACTCCGCCGCCTCCAGCGCCGCCGGAGGAGGATCCACTGCCGGCCGCTGACGCTCCCTAGGGTTTGGATCTGCAGCGTTGTTGAGATCCGATGCGATGACGGCGGAGGCGAAGGCGGTTACGCTGGTGGTCCGCTTCATCGCCGGCGGCCGCTGCCTCTTTCCCAGCGTTCAT

>UN00443

GGTTCCTATTAATTTGTAAATTAGTAACTAGTAAATGCAAAAAATCATACAAAATCTCAAGAGTTCTGCTCATAACTATTCACCACCCTCGGTAAGATCTACATCAACATAGGGGAAAACTAGAATTTGTAAATAATTACATCCGCCATGAATCTCTTAGGCTCATGCCAAGAATGCTGAAACAGCAATCCAAGTATCACTTGTTAGAGAAATGATACAGTCTTGTGATTTTTCTGCTTTCTTTCCTCTGTAGTGAGATAGTAGGGGTTACGTTTGCATACTCCTTGCATCTTCTATCATGCGAGTAAGATCTTGGACAACCTCGGACATTGGAGGTCTGAACTCCGGCTGTTGCTGAACACAACGACTTATAATATCTGCAAACCTTGACAATGATTTCATAGGAAGTTTTCCATCTATGGAAGA

>UN00444

ACTAAAAATCAACACATTCCTTCTTTTTTTCCCTGTTATGAACAAGCTTGTCAGATGTCAGAAACATTCATACTACATCAACTCACTAAATCAACGTGCAATTAGAAATGCTCGAGTTAACAAAGGAATGGTTAGGGCAGTAAAGATTGCAGACCACTTTCAGCCCCTCTCACTGAATAATGCAATTTTACATATCCTGCTTAGAGGATACTGACAATGATACAACTTGCTATAAAGTAGAGGGATGAATATCATGGAACAAATTTATGTGGATGAAACTGAGACAATATAACGACCAAGAAGAATAAGTAATTACTAACTAATTAAGTAACTAAACTTAAAGTTTAAAAAAACTTTAACC

>UN00445

TACAATACAAATATATATTATATTATTTATATAATATAAAATAATATTAATATAAAATAATATATAAAAAATATATGTATGTCACATATCTAACACACTAGGGAAGAAAATAGAAGAAAGGAGGGGAAAATTTGAAAATGAAAAGGGATTTTTATTACTATTAGTTGTAATTGAATAAGCTCATAGTGCCGTGTCTATAATTTACGTTCAAACTTCAACTACAACTAGTAGTTAATATCTTAAACAAGACATTCCATTACCTAAGAAAGGGAACTTCCATTTTTAGTTATTTTTATTTTATTTTAATTCTATTCTATATACAAAGTAAGAAGTATTATAAGATAAAGATTTCGGATACTTTCTTATTGGATTGGAATCCAATCAATTAGTTCCGCAGTAATTACTACAAATAAATTCACTAGAATAACTAGATCTTTTTTCATTTATTGATGCATCCATATTCTACTG

>UN00446

TATTCTAGTCTAGACACCATCTTTTTCAGAAAATAGCTACTCAAAGTATTGATAGCCTTGTCACTTTCGGGATGATTTTTGAGAAAATTTGCTAAACTATACACTTCGTTTTCACGATCTTCACGGAAATTCTCTGCATTGCCACGGAGATCAAGTTCGGCTTCAACGATTAAGGTAAGAAGCTCATAATCGTTTTCTGCCAATTGAACTAGCAAAAGTTTTCGAGTAGTATCTTGTAGCGTTCGAATATTTCTTGATGTTGCTGCGAATAACGAATCAAATGCATCCTCCAAGCTAGATAATCGACTCCATACACCGAAATACGTATTTACCATTTCGCGGGACAACCCTTGTGCAATATATTGCAACTTTAAAGCCAATGAACGTTGAAGATTTTGGTTTTTGCGTATTTTTTTCATTTCTCTTGCACGATAGGCTCTTACAT

>UN00447

GTAGCATATCTCACGAAACAGACCAAATAAAAAAGGACCCAAGTCAGAAGATACAAGAACAGAGCATGCAACTAACTCGTAACTCGTAGGACTTAAAAAAGAATAGAGACATTACATGTCATTGATGTCAATGATTGAAAAAGAATCATTAAATATAAAGCAATAGATAACACAAATTGATCAATGGAATCCTCTTACCCTGTATGAAGCGTGAAAACTAAAGTCCCACATTGACCGACTATAATTATCATCCTTAA

>UN00448

GAAACAGTCTTGTATGTTCATTTAAAGCCTACCCATGGGCAACTATTGACAAATACCATAGTTTGGCAAGCTTTAGATCCCACTGAAGTATTAAAGACAATGATGCCACTTATTTTGCAATGAATCAGATCGATGAGGAGCTCGTTTTCACTGAACACTGCATTGCCAAGTCTTCTCCATTTTTTCCATCATCAGATTACAAAAAATTCTACAAATACAATTAGACTAAAGAAACAATAATAATACC

>UN00449

GGTTCGTAAACTTAGTTGGTCGGTGCTAAGAGAAAGAGGACGAGGAAACGCAAGAGAATTCAAAAGGATAACAATCTGGGAGAGTCTGGGTGTTAGTCGCTGTTGTTGCATTTCTTACTAAATTTTGCAACGGCATATTCTTCTAAGCCCAATTTCCTAGCTTGTGAGGACCATTGTGAGGCGAAGATGAATCTGAAGAAATAGCCACCATTTTTGAAATAGGCAGGTGGATGAGGCCCGTTTTATGGCAGGGGAAGGCTTTTAGTTTGATTTTTGTCATGAAATGTTTTTTGAGGAGATAATGGTTTATGTACGCAGACGCGTTACTATGTTTGTACTCGTTTCTGAGAGAAAGACAAGTTTCTTGTTTCTTTTTGTGAGATTATCTACAGTGTTCTTTTTTTTCTTAAAGATATTAGAAATCTATTTAGTGGTGAGACCACGTTGTAG

>UN00450

CTCGATCACAGAGTAAAGAAACCCTAGCGGCACGATGAAGTTCAACCCAAGGGTGTCCAGCTCCCGCCGCAAGAGCCGCCGGGCCCACTTCACCGCCGCTGGAGGAGCCGCTTCGCTCCAGGAGATCGATTGAAGAAAAAGCTGAGTCTTTTATCTAGTTATTGAATTTTGAGGGGTTTTGTTCCGTACTTTTTAGGTGTTTAATGGCGATGATACTGGTAGCACTCGCCGATGAATGTTTTTTCAAGACTTGAGAGAGTTTGGTGAAGGTACTTTTTTTGAATCTAGTTTGTGTTATTATGAGTAGTGTGTAAGTACCCC

>UN00451

ATGCAATGGGAAGATTTCATTCTTTCATGAAAATACTGTACAACACAACATGAATTGAACTACTTGTGCTTTGATGATATTACAACAAACTGATAAAAACAAATCAAAAATACACTGATTTCTACGTCAACTACTCTTGGCGCTTCTTAAGTCCATGTTCAATGACGAATCTATCTTCTTAGTATTCTCCTTGTTGATAAGTGAGAGCAACTCCATTGATGATCTTATCTTTTGCAT

>UN00452

GCAAGTTATAGAATGATGGACTGTTTCAGTGTTAGATTCAATTGAATGCAAGGGGTCTCCTTTATTTGAGGTGGAAGACCACCTGATTTCAAGCTGTCAAATTATATACGTTGGTCAAAGACCAAAAATTATCGGGCTTGCAGGCCAAAAGAAAACTGTGTTTGTGTCTCGTTGATGGAAGTATCGATTGAGTGAAATTGGACCAGAAAAAAAGGAGGAAAAAATAATCTGCCTTTTATTCATATTTTTGTTAGATTAGATCATGTAAATTTAGGAAACTTTAGCTGCGTCGTGGTTCATGAGAGCATGTCCAATGTGGGGTGGGGTGGCGTTGGGGATGACATTCCTGAAGTTGAAGTTTTGTTTCTCTTCCGTAGTGATATATACAGCAGCCAAGGGCTGATGCTTCTTATATTTTGTTAAATTAAACTAATCCTTTTCTGCAATATGGAGTACAGCTTTGCATCTG

>UN00453

ATTTCATTCATCAGTAATTTTCCAAACTGATTACAGGAGAGTTTAAATAAAGCAAATTACAGTTTTGGAAAATCCTCTAAATCCCTCAAATCTCTCTATAACCAAAATACAACCACTTAACTAATTAGCTGCCAAATGAAATTCGAAATTCAAAAATAAATTCAAATTTCAAACTAATTATTACAATTGAAATCAAATCCATAGTGCTGCAACCTGGCGTCTCCTTCGCTGATAAACTCGGCGAAAATCCCGTGTATGTGTCCGGATGTCCCCATCATTCTCCCCGGGTAGGAGAGAACTCGACCCCGTCGAGTGTAAATCAGAACGGCTGTTATACTGCTCAAGAAGATCTGGATCCAGCCTTTGCAAGTCATCCCTATCGAGCCAAGTGTCATCTGACTCAGGTCGCCCTTTCCAACGTACCAAATACCTGCGGATACCACCATTGCTAGTAGAAACAAT

>UN00454

CGCCTCGCGAGGATGGGCGGGGTGAAGAGGATCAGCAGACTGATCTTTGCCGGCAAACAGCTTGAGGACGGCCGTACCCTCGCTGATTACAATATCCAAAAGGAGTCCACACTCCACCTCGTCCTCCGTCTTCGTGGTGGGATGCAGATTTTCGTCAAGACCCTCACTGGGAAGACTATTACTCTCGAGGTTGAGAGCTCCGATACCATTGACAATGTAAAAGCCAAGATCCAGGACAAGGAGGGAATCCCCCCAGACCAGCAGAGGCTTATCTTTGCTGGCAAGCAGCTTGAGGATGGGCGAACGTTGGCAGATTATAACATTCAGAAGGAGTCCACCCTCCACCTTGTCCTCCGTCTCCGTGGTGGTATGCA

>UN00455

TATTTCTTGCGAGCTCATCGATCTAAGAACACTACTACCCTGGGATAAGGAGACAATAGAGGCCTCTGTCAAAAAGACTGGAAGGCTCCTGGTTAGTCATGAAGCACCACTTACTGGAGGGTTTGGTTCTGAGATATCTGCTTCAATTATGGAATGTTGCTTCCTGAGGCTTGAAGCTCCGGTAGCAAGAGTATGTGGTCTCGACACCCCCTTTCCACTTGTTTTTGAACCATTCTATATGCCAACCAAGAACAAGATACTGGATGCCATCAAAGCTACAGTTAATTACTAAGAGGTGGATTTACAGACAGACATGGAATCCATACGTCACAGTCAAAGCCTCGGACCCACGGTAGACCTGGCTTTGTAATATAAAGCCATGCAAATATGTATTTGATTTGAGCAAATTACTCTGGTCGTTACGATCTGGAGAAAATTGCTTGCAATTCGTACCTGTTCAATGTTGTGCAATTAGCGTCTGTAAGAAGCCCAACTCTCTGATTATATTTATAATCGAATTTACGTGCCCTTGT

>UN00456

GTTTAAGTTTATTTAGTTCCTTGTCGTAGGGTTGTTTGTGTCGAAAGTGCACCGGCAACAGCCGGCGATCCAAAAGATGAAGCACTTCGCCCTCGTCGCCGCTGCCTCCGCCGCTGTCACTGCCTCCTCCTCCGTTGCTCTCTCCGCTGCCGCCGATTGTAGCGTTTCTAATAAGACTAATACCCATAAGGGTTCGATTTCGTCTGGATCTATAAGGGGCGTCCAAAAAAAGGGGATAATTTTTCCGAGGTTAGATGGGCTCAGATTCATAGAAACGCTTGTGACGGCTCACAGATAGATCATCTTTGTTTTAGTGTTCGGTAGAGATGTGATTTGAGAGAGGAAAGTTTATCAGTTTTCTGAGGCTGTTGTTGTAATCTATTTTGTTATTTTTTGTCTAAGTAAGTTTATTGGTTTTAGTACT

>UN00457

AAGTTGCATATTATGATAAACAAAGAATTTGTCTCCCACTGCAAAATAGTTACATTCATTTCCATTTTTAAGCCAACACTCAAAGACTTGTTTACATATTCCAGTTTGCTTGTAATCTATGAAGGAAAAAAAGAAACACAATGGAAAGAGAGGAAAATGTGAAAGAAAAGAAAGAATGAAACAACTGTTTAAATATTCTTGTTTGATTGTAACCTCATGACAAGAAAGAAGGGAACAACAGGTTTTGTCAGTGCACTATCAGAACTTGCAAGCATCCATGGTATCACTAGCAG

>UN00458

TAACCGAAAATAAGTAACTAATTTACAAAGAAAACTTACAATACGGTAAGACAAAGACTTGGTACATTGATGAAGTTTCTTGAAATAGTAGAGAGGTTTTAGAGTACTTGTATTTTGGTTATGTTACCATGTGTGGGTTATGGGTCAGTGTATCTCCTGTTTTGTTGGATGCTATGTGGATTGATCTGAAATTTTGTACTGAACGTAAATGCTGTTTCGATATTGCACCTTGTTTTCTGCTTAGTTTGAAAACGGCAACTCGTCTGAGCCTTCAACGACGAGGGGTCCTCTTGACTTCTGACCGCCCTCCTGGTGGGTATTTGTGTTGGATATGGATTTGGTCGGAAGTAAGTTAGGAAATAAATAAACTAAACGTTT

>UN00459

ACTATCAACTATATATTAATTTATAAACCATAAATTATATCTGACCCATATAAATACAGTCTTCCCTGTCAAAACCAGCATTTTAGAAGCTTTAAACATAACTATGAACATTACTTCTATTATATCCATGCATCGTCAAATTGTTGTTTTAAAAAAAGGAATCACCAAAAAATTGTATTGAAGCCATTGACATACTCTAGATAACCATGTCCACAGCTCATTGCTTGCCTGTGAGAAGGCGAACAAGGGACTTGCTACCTTCAGATCTTCCTCTTCCCACTGCACTTAGTTTTATTTCTAAC

>UN00460

TTCCATTATTTCTCAGTTCAAGAAAATAACGTTAGATAATAGTCTCGTACAAAATTACACACACAACTCTAATTTAGTCAAATTCGTAGGGGGGGAGGGAAAGGGGGCGGGGGGAGGAAACAAACGCGCACACACACCCCAAATAATTATACATAAATCAATCACGAGACCAAATCCAACCTATCTCTCTCCCAATCCCGATCCCGATCCCGATCCTTTCTAATCCCGCAAAATTCCGTTCATAAAGTCCCAATCGACAGCAGCGCCATCGACAG

>UN00461

AATTATCAAATAGTAGTTAACCCTACCTACAAACTTATCAAAATCCCGCAAATACATAATACAAAAAACAACAGCAATGTCTTGTATCCTGTACAATGTTTTTTATTCACAGATTTGATCGGTTCTTTAAATTTCTGAAATTTTACACAAGCAGATCCCACCAGCGAACCAATCAATCAAAGGAAGTCGAGCAGAAACTCAAGAAATCATCATCGTGCTCAACAAACCTTGATCGGTTTGGCCAGAGACTTCTCCTCCTCCCTTTGATAATTCGGCGGCGTATAGGGAAAGCACCGAGACATCTTTCCTCGCAATCTTCAGTCCCCAACTCTCCGAAATATCGCCGGATAAAAAAACAGGAATCGAGGAGGAGACGAAGGGATCGGAGATTATGATCGATCTCTAGGGTTCAGAAGGAAAAAAAAGCGCGGGAAATCGATGAGCAGAGCGCGCTTTGCGACGCTGGAGGCGGAGGCTAGGGTTTCGAA

>UN00462

ATGGAGAATAAACTTCTCACTCAAATTCAATTTAGTTCAAGCATTGCAGCCGCTTAACTGGTCTAAACAAGACTTGCGACACTCAAGAATTCTCAATGGAAAATACTATGAACAGAGAGAATAACGTCTTCCACTTTACATTAATGATCCAGGGAATATTCTATTACACACAGTTGATGGCAACCAATGCGAGTCCAATCGGAATTTCTAAACAATATGCATATTTGCAACACCCATAAGATACAAACAGTGTACATATTCTAAGCTTTAACAGTTCTTCGTGAGAATAGATTCACAACTGCAGCAACTATCATCTGAAGGTGAAGAATAAAGCTGTCCTTTGATACAATCAACAAGAACAACAGTATTCTGGTTATATATTAGGGGTCGCCTTCTCGAATTCTCTGAAGGCCACAACTTGTCTCCTGATTTACGAGGAATGATGTGGAAATGAGTGTGAAATATTACTTGTCCTGCAGCTGATCCACTGTTAACCAGCAAGTTGAATGAATCACTTTGAGTAGCTTTCATAATTGCATTGCTGAAGAAAAAGGTAACTTTAGGAAGAC

>UN00463

AGTAATAATAATAGCCAAGAAAGAATGGATTGCCTCTGCCCCCGTGTTTTGCTCGTCTTCCTACAGAGCTTATATTTAGGGTCTTGGAGCTTCTCCCTGGTGTTGATATTGCTAGGTTTGGATGCACTGGTTCTGAAATGAGGTATCTCACTTCAAATGAATCACTCTGGAAGATGAAATTCATGCAGGAGTTTGGGCAAGGGAAGGAGAGTGAAATTGCATCATTAAGCTCTTGGAAAGCTAAGTTCAACGATGTTGGGTCAATAAACGAGATGGTGGTAAAGCTGGGAGGGCTCTGAATGGATTACATACATTTCCCGAGAATCTCAATGCCAAGGTATCCTCCATTTGCACCACAGGGGGTTTTCCGGTTGTTGGTGGAGATTATGACCGGTTTCC

>UN00464

TTTTATTTCGGTTTTATTTGGGTTACATACCCGACTTTGACTTGAACCCGAAAGCAAATTTAAGCATCTAGCGGCTAGGGTTTCCACGTTTCGTAACTTTCGTCGCTCCTTCTCCAACTATTTTCTTCCAAAAGCGAAAAATAAGTTGCGGTCTCCACTCAGCAGGGCGACAGTGACGGGCGACAGGCCACGACGAGCAGGTACTTCTCTTTTTTTCCAGTTTTCTTTTATTTTTCAATTTCTTTCTATTTCCTTTTTGTTTTCTAGTTTCATTTTGTTTTGAACCCTTCTTTGCGTATCCTCAACGATATTTCTCAAATT

>UN00465

ACAAGGTGCCCGGTAGTGCCTTGTGGAAACAATATAGCTATGATCTTTGTTAGCATAAGTTGAGCTACCGAAATCAATAACCTTGATAGCGCTTGATTTGGGCAACCTCTTGGAGGAGGATGCATTCTTAGAAGATCGAGATGAACCCTTGTAATCAGGTATTTCTATATACTCTGAAGAAACAAGAAAGTAATAGTTACTACAAGGCTTCAAATCAGTATGAATGAGGCGTAAATCATGCATAAATG

>UN00466

TTCTGCCTTTCCGATTTTTCTTGTACAACTCCATCAGATTTGGATCATGGAATGGGAGGTAACCAGCCAAGAGAACAAATAAAATCACACCACAAGACCAAATATCTGCTTTAGCACCATCATATCCTTTTCTGTTGATGACTTCAGGTGCAACATAAGCTGGAGTGCCACATGTTGTGTGTAGTAAGCCATCCTGTTGCTTAGAATCAGCAAGAGCACTCAAACCAAAATCCGAGACCTTTAGGTTTTCATTATCGTCCAAGAGGAGGTTCTCTGGTTTCAGATCAACGGTGATAAACACCTCGGCTATGACAAAAGTCCACCGCACTGATAAGCTGCTGGAAGTATTTTCTAGCAATGTCCTCCTTGAGCTTG

>UN00467

TATTCTAGTATTATATATATTCCCCCCCCAAGTATAATATAATTGTTTTCTTCGTCTTTGAGAAGTTATTTCGTCTAAACCCTAGGTTTAGATTTTCGAGGGTTAGCGTTTTTGATTGGGATAATGTCGGGAGCAGGGGCGGAGGAAGACAAGAAGCCGGCCGATCAAGGGGCCCACATCAATCTGAAGGTTAAGGGTCAGGATGGGAATGAGGTTTTTTTTCCGCATCAAACGTAGTACACAGCTGCGGAAGCTCATGAATGCCTACTGTGACAGGCAGTCTGTTGATTTCAACTCCATTGCCTTCCTTTTTGATGGTCGCAGGCTGCGAGGAGAGCAGACCCCTG

>UN00468

CCTAAGAACAAGTAAGGCACCAAAAATCCAGAGGCTTGTGACTCCCTTGACCTTGCAAAGGAAGCGTGCCAGAATTGCAGAGAAGAAGAAGAGAGTTGCCAAGGCCAAATCTGAAGCTGCAGAGTACCAGAAGCTCCTTGCCTTCGAGGCTCTAAGGAGCAGGAGGGACCGACGCAGTGAGAGCTTGGACAAAGAAGAGGTCCAGGTCTTTCTGCTGCAGCTGCCAAGCTGCTTCA

>UN00469

TTACTTACCTTACTACTACCTCTCTCTCCTTCTTCAGATCTGCTCGGGTTTTCACTGTTCTCCCTCGAAGCTGGTCGTTCACTCGTCCCTCGAATCTCGTCGCCGACTCCTCCCTCGAAGCTCGTCATCCCTTGAAGCTCGTCGTCCCTCGAAGATCGTAGTTTACTCCTCCCTCGACTGTCCCTCTCGAGTCATCAAAATATAATTTTGCACGAGATCTGATCTCATCCCTTGCGGATCTGTAATTTCAATCGGCTTCT

>UN00470

ACTCTCTACCCCTATTTATAATAAACATCAGTGAATGAAAGCCGTTGGATCGAAATCTTATCCAAAAACATCAAAAATCAGCCATAGATTCATAACTAACTGTACATCGTATCGATGCTGGCATCGATGCCCCCATCGATGCTGGCATCGATGTCAAGTTGTCCCTGGTTGTTGTTCGTAGATTCCTCTTGGGTTTGCTCTTGGGCTTGGGCCTTGGACAATTTCTTGATTTCTTCATGCATTTGTTTGTGCTTTGGCATTTCTTAGGCTTTAATTCTTCTTCTTAGCTTGGGCTTTTGGATAATTTGGAAATTGATTTTCTAATGGGCTTCTTTGTTTTATTTCTTCAT

>UN00471

GATTTTGTGATGAAGCGCTTTGGGGGAAAGCAGTTTGCCTTTTTTTTAAATTTTAATTACAGGTTGCAGTAATTTACTTTAATGAAAGTAAAAGTATTCAAGTAGATGCATGTTTTGCCCTTGTTATTTTTCGAAATGTTGATGAGTCGCCCTGTGGCTTCATTCTCT

>UN00472

TTAGTTGAGACTCTGCTTGTACATAAGAGAACTGATTTGAGACTGGTGGAGGTGGTGGCTGCAAGGTATAAGCCTTATTTGGCAAGGGTGTGCTGATACTAGGCTGAAAATTATTTCCAGAATGCATTGCTGTCTGAGAATTGTAAGGGCCAGAGGAACCATGAGGACACACAGCTGGTGGCATCTGTTTAGGGAAACCACCATATCCATGAGTATAATACGGTGCCGACTCCGAAGAAATCAACTTGCCTTGAAAACTATTTTGTTGTTGACTAATTGAATGTTGTTGATGGTTCTGATTATTGTGGTTATTGGAAAGGTGGAAACCATTAGCATTACTGTTAGCATGAGATCCTCCGGATTGTTGTTGGGGAACAAGAGAGATTGGGGTGGAGCAAGATGGAGCCATAGGTGGAGGAGAAGAAGGCAAAGGGGGTGGAGATGGTGGCAAGTCCTCAGGAAGTGGAGGAGCACAGGGCAAGGTATGATGTTGCTCATGCTGATAATGAGCATCAGGTACAGTTCCAGTGCCTGAAACATGACAACTTGAACTTGCTTCTCCTCCACAAGGAGGGGCGACATCCTCCATCTCAAGTTCACCATCAACATCTTCCAAGATATGACGATGCTTCCCGCTGGGAGTTTGAGTCACTCCCTTATCGTCCTCAACCTCTGCATTCCGTTCAGGGGTAACAGCTTCAAAACTTTTCTCGTCATCCCCACTTCCTTCATCGTCTTCAAGCAAGGGAGGATGAATGAAATGTTGCAGCTGAAAACTTGTGTTGCTTCCATACTCATCAACAAACATTCCCTCCATCTCACGGATAGGATCATTAAGGGAGCGCTCTGTTCTTGACGGACGTCGAGAATAGGAACTAGCTAATGATATTTCATTTACAGATTCAAGCC

>UN00473

TGGGAGGGTGGGAGGGAGGGAGAGGAAAGTTCTGGTGGACAAATCAAATTTGAAAGAAAATGATGGACCAGCAAAAAGCAGCAATGAGAAGTTCACAGTACTAGAAAAGAAAGTAGAAGAAGCATGCCCCCTACTCCTCAGAGGATCCCCTCCCCTTCAAACCGCCGCCCTCGCAAAGCCGGAACTAATCCTGCAAGCTCTCCTCTGAGTAAAGCTCTCAAACCTAAAGAGAGGGGGATACTGAAGGAGCTGGATCAGAGGAAAGAGGGGGAGAAGAAGCCATTGGTTTCTAAAGATGAAGAATGCAACAGGGCTACTGATGTTGGTCATGAGGGAGGTGAAGGTAGTTCGATGGATATGTTTTGGTTCTTCAAGCCTTGCACTTATTTGGTCAAGTGAGTGAGTTTTGTGTTCTCATGAATTGATATGATACAGTGGTAAATTACTGAGAGACTGGGGGATACATGTTCAAAATAATATATAGGTGATTCATTA

>UN00474

GTAAAAGTAAAATTTTTAAAGAAAAGGTAAGACAATAGCTAGCAACAAGAAATTCGATGGTGATGTTACTCTTGGTGTCATTGCCATGAGGACCCCTGGTTTTAGTGGAGCAGACCTGGCTAATCTTTTGAATGAAGCAGCTATACTCGCTGGGCGTCGTGGAAAGACAGCCATTTCATCTAAAGAAATAGACGACTCAATCGACAGAATTGTGGCTGGAATGGAAGGAACAGTGATGACTGATGGGAAGAGCAAAAGCCG

>UN00475

AGTCTACTATAAGATTCATTTGTAAATGTTCTGCTCTTTCCTCTACATCTCATTTCTGTAAAAACACAATAGCTGATTAAAAATCAATTTAACATGAGCTAAACAAAAGTTATCCAACCAATGCAAGCTCAGCCACAAATTACATCACATGTTTTTTGCAATGGAGAAACCAACAAATACCGCAACGATACATACTAGAAATACTAATATTGTAAACAATGAACCTAATACAAAAGCATAGATAGAGAAAATAGAAGTTTCCCCATGTTTTTCCTTGTCCACATCCAGATTATGGCTCACTGGATATCTTTGTTCTTGAGCATAAACAATGTTTGGACAGGCCTCGAGCTGGATGCTGAATCACGAGCGAGTGTGTGCTTCTCCCATTCAATCAAAACCTCTTCGTATGTTGCATACCCTTTGTAGGAGGCATTTGGGAACTTATCTACCTGAGATTTGCATTCCTCCCAATTATTAAAACTACCACATCGTCGACCTCTCTTCACAACATAAAAACTTTTCCTAGCCATGGATAAAC

>UN00476

ATAAAACATTTAAGAAGAAATCCTTTCATCAAAATAAGAACCCTATCCACTTTCAAAAGAAGGAAGGAGCTGAACGTCGAGCATGCTTCATTTGTGGAAGAACCAACCATCTTGCTAAGGACTGCTTTTTCAAGAAAACTGAACCACCCAAGCCTAGGAAGGGAGGACCTCCACCTAATAAGTCATAAGTAAACATGGTGACTTCCAGTTCAGCTGAGGCTGCTTTCAGGTCTGTTCTCTTCACTCCTGAAGTAAACATGACCTTTCAAGCAAATGATTGGTGGATTGACACTGGAGCTAATATTCACGTTTGCTCTAATCAATCTTTGTTTTCCACCTATCAGGTCTCAAGTAGAGGAACTGTGACAATGGGAAATAATAATGTGGCTCCTGTATCCGGAAATAAGGTAC

>UN00477

TTAGGGTAGTTTAAGTAAGCAAAAAGTGCTGGGAGATCAAGAACAGAATTCGCAGGATAGACTTAATGAAGCAAGCCCTTCAGCGGGTACTGGAGGATGTAGAGAACATGGCGGATTGTATCTGGGGAGTCTGATGAACCTCGTATTCTTACAAAAGATCCAGAAGGGCTTTCATATTTTCTTCTCCATCAAGGAATTACACGGACAGGGTACCGAGTTGCATGGCAAGATGTTCTTGGTTGTCGCCTGCTTGTGGATGAACTAGTGTAGGTTTGTTCGTATAGAACCTCTAAATATGCACTTCCTTGTAGGATTTTG

>UN00478

AGATGGCTTCACTCGATCCTTTGAAATAGTCATGATTGTACCATCATTAAGGATTGCAATGATCAAAACCATGAAAGGGGAGAAATCAAATTTCCATATCAATGCAATAAGCATGAATCCTAGCTGCAATAGTACCGTAAATTACCCTTGCCCTAAAAAAACCCAGCCAGCCCCTAACCCCGGCTCTCGATCTCGAAAAATGGCGCCCAAGGCGGAGAAGAAGCCAGCGGCCGAGAAGAAGCCCGCGGCGGAGAAGCCGACGGAGGAGGAGGAGCCCAAGAAGGCGGAGAAGGCCCCCGCGGGGAAGAAGCCCAAGGCCGAGAAGAGGCTGCCGGCGTCGAAGGAAGGGGGAGGGGACAAGAGCGGCAAGGCGAAGAAGAAGAGGAAGGCGAAGAGCGTGGAGACGTACAAGATCTACATCTTCAAGGTCCTGAAGCAGGTCCACCCCGACATCGGGATCTCCAGCAAGGCGATGGGCATCATGAACTCCTTCATCAACGACATCTTCGAGAAGCTGGCCCAGGAGGCCTCACGCCTGGCCCGCTACAACAAGAAGCCCACCATTACCTCCCGCGAGATCCAGACCTCCGTCCGCCTCGTCCTCCCCGGAGAGCTCGCCAAGCACGCCGTCTCCGAGGGCACCAAGGCCGTCACCAAGTTCACCAGCTCCTAGGCCTAGCGCGGCTCAAGGGGTTTTCCTCTTTTGATTAGTATTTTTCTTGTTTTTTTTGTCGCTGTTTAGTTGCTATCTCTAGTGTAATTATTTCCTTTTATGCCGTGGATCAAGGAAAAAGCGCGCTTTTTTGTTTCTCTCGATGCCTGATTAGTGAGTGATTTCTGTGGATTAGAAGTAATTTATTGTGTTGGTTATCCTTCAAAATCGAAGGACGTGACCTTTATGTTTCGTTTGTAGACTAATTGGTTACGTCTGAAATTGCTCTCA

>UN00479

TTTTAATCATAAGTACATACTCTATAACCTTCTACATTTCACCCAATAGACGATGCAGACAGATACCTCGTTATAAAAATAACATAGCATCCTTCCATAATTCAACACCAAAATCAATTTGGTCGTTGCAAACTAATAAGATTACAACACCGTGGCTAGATCATTTTCCCACCTTACCTTAGGATAACATATCAACTACATCCTTGATGCCCACATTTCTTCTTTGCAAAAAGTTGGGAGGGCGCAATGACATATTGATCTATAACAAAAGAGAGAGAAAAAATGGCACACGTGCTAATATAGTGTGTACCAATTTATGACAGTTGAAAGCTGTCATGATTTGCTCTTGAACTTCCTCTGTGCTTTCAGATCAATCACAACTACACTTATGTTATCCTTGCTTCCTTTCTGAAGAGCAAGTTTTGATAAATACTCAGCAGCAGCTTGAGCAGCAGGATCAGCCTCTTCACCCTTTCGCAAACTAGAATCTGATGCGATAATACCGTTCTTTTTGTAGACCAAAAGTAAGAACAAAATTAAACTTAAACTTAAACTTAAAAGAAAAAC

>UN00480

ACCGCCCCCCCAAGCACCACCGCCTACTCACGGCCGGCGAGCGCTACGCCGCCCTCAAGACCAACGTCGACTCCCTCAACCTGATCCAGGTCGGCCTCACCCTCTCCGACTCCGCCGGCAATCTCCCGACCCTAGATTCCGAATCCGACACCCGCTACATCTGGGAATTCAACTTTCAGGGACTTCGACATCGGTCGCGATCTCTACTCTCGGGAATCGATTCAGCTGCTGAAATCGAACGGGATCGATTTTGATGAGAACAAGGTTTAACGGAATCGATTCGAGTGACTTCGCTGGATTGCTGATGTCATCAGGATTAGGTTTTGAACGAATCGGTGAATTGGGTTACTTTCCACAGCGCTTATGACTTCGCTTACCTGATCAAAATCCTGACTTGCAGCGAACTGCCGAAGAATTTGGGTGAATTTATGGGGCTTGTTAGGGTTTATTTTGGGGGAAAGGGTTTTTTTGATATGAAGCATATGATGAGGTACTGCGATGGATT

>UN00481

AAGTTGGTAGGTAAACATCATTGACAAGAAAATAGTGAAATAGTCACTCCACATGTGAAGGGTAAATAAATATTAGAGAATATTACAACACAACATTTGTTATTAATTTTAGATGCTAAAACTGCTTTAACATGGAACAAATTATATGTTGCAAGATTCACTGAAATTATTGATCGTCAAGGCTGCTTGAGAGTTTATAATTCCTACTGTAGCCATTAGCCCTTGAAAAGATTCGGC

>UN00482

CCTAAGTCCTACTAGTTTAACTAAACAATCCGCGGCCAAACATATTGAAGAGGTGATTATTGAAGACGCATAAATTACCACGCGACAAGTTCCACAGGTTCACTGTGTTTTTCTGATACTCAAAAGACCAAAAAAAATTCAGTTATGGCACTTAATTAAAAGGCGAGATCCATGTAAGTTCTCTGGATAATGCAGCAGCAGCAGCAGCA

>UN00483

GTTAACAACAGTTTCAGGTCTAGATTGCCCACTACAGCTACTACTCAAATGGAGGCAGAATGGATAGAACAATATGAACCTGGTATATACATCACTCTTATGGCACTGCATGATAGGACCAGGGTAACTGAAGAGGGTTAGATTCAGTCGGAGGCGATTTGCGGAGCAGCAAGCTGAAACTTGGTGGACTGAGAACCGTGAGAAGGTGTATGAGAAGTACAATGTTCGAGTGTCTGAAAGAACAAACTCATCTGGAGTTCCCAGATCACCATGATAGTTTGCAACAGGGAGAGAATTTTTTGCCATTTTTCACCCAGTCTCTATTGGCGCTGCCAAGATGAGTTCTATAGAAACATGATATCTTAAAGTC

>UN00484

TACCTCCTATGATGATGATGATGATGATGATGGCCATGGCCATAGCCCGTGCCCAAGCTGCTCCATCCTGATCTCCTTTCCCCTCAGCTGCTTCCTCATATCATACGCATCCTCCCCATACCGCATAATACTTCGCCTCCACATCATTGATCTTGTAACCAAGCGTATTAGCATACAAATTGAATGCCGCCCTGTTGCTCCTCCTCACGTGAAGACGAACAACAAATAAACTAAACAAAAGAAACAAAAC

>UN00485

TTCGACAGGAAACTCGAAACTTGCTGAATTATCCTCGAGCTTCAGAAACTGGTGATACCCTTTCACCTCCTCATCCTCCTCAATATATGAGATATCGGACTGATCAGGCTCGAAAGCATTAGAGGAATCGGTGTGAGCTCCATCCATGCAATGAGGGCTTTCAGAATCTGAGACCGCACTATCGCTTGAACTGTGATCCTCTTGCTTTACTCCTACACGTGGGGTTTCAGAATTTTTGAGCTCAATTGTGATGTTATTATCTGTTGTCCTGAGTTCTAATGGTTCTGTGACACCTTTGTGGTTTTCTTTGAGGAGGAGCTTGTCCGTGAGATGAACTACCTCTGCTTTAAGCTTCTCCTTCTCCTTAAGAAGATCATCATAATCCCCCTTAAGAACATCAAAGCTCTTCTTCAACACCTCATAATCCTTCTC

>UN00486

AAGTTATCATAATCATAAACTAGAAATTGAAATAGATCAGAAAGATAAACCGTAGTGTCGAAGTTGCACAAAATTCTCCACAAACAGCAGATTCCAAAGCAAAAGAAAAACCTAATCTAAGAGTTTTTAGAAGAAGAAAGAAAGAAAAAGAAGAAGAAGGAAAAATCAAGATCTCCAGCCTCCAGGTCCTCCATGATGCTTCAGGACGTCTCCTTTTTA

>UN00487

GTTTTCCTAAATTGACTTTAACGTAAACAAGTTGTAAACATTAAAATAATATTACACTTGTAGGTAACATGAAAGACAACTATGAGACTTCTCTTCTCCATATATTCTTTACTAAGAATCGGATATGAAACTCTAGCTAGAAAGTAGAATTTTACTAGTTAGAAAAGATAAACTGGAATCTCAGAATATTGTCTCCCCATTGCGTGCTCCAACTGTTCATTATTTTTCCAATAATTGACCCTGTCTTTTACACTATACCCAACGACAACAATAACGTGCAACCCCCCTCCAGTACTATTGCCAGGAGACCTGTAAACTTTTCCGACCTGAATAGATAGAACGAAACAAATTACACTAATTAATATCCGAACCAATTATTCAATTTTGAATTTATGTAAAAAACATAAGGATTTTCACAGTCATGACTTTTGACACATATTTTAATAAGGATTTT

>UN00488

CTAACCCTGGGCCTCCCATGGTATAGCAGGGAGAAGACCGAGTTCTTCCCGGTAGAGGAGGGAGAGAGACGAGTAGTTTTGGGGGACGCGAGCAGTGGAGATCGTGGGGAGGAACAACTCTCCTGGGCTTTTATGGAAGAAGATAAAGCTGACCACCACCCGCGGGGACAACGCCAAGAAGCGCCTCCGCCGTGTTCGGCAGAATGAGGCAGTTTTGAGGGCTTGTGCGGAACCACCACCACCGAAAACTTCGTTAAACAATGCTCCAGCATCTTGAATTGAACGGAAGGGGATTGATAGTTAGCAAACCAAAAGGTTGCTGGATTACTTGAACAATGGTCTCCTGGG

>UN00489

TCTTTCTCGTCTCTTCGTTGATCGGCGGAGCTAATCATCGGAATTATAATTAAGAGAATTAATAATTCGTAGGATTTGTACCGATTGACCACGATGAGCGAGGTTTTCCAAGGATACGAGCGCCAATACTGCGAGATCTCCGCTTCTCTATCGAGAAACGTGTACATCGGCTGCTCATCTCGATGGTGAGCAAAAGAAGCAGAAGATTACTGAGATTAAATCGGGAGTCGAAGATGCTGAAGGATTGATTAGGAAGATGGATCTTGAAGCTCGAAAGTCTGCAGCCAAGTGTGAAGGCTGGTATTTGTCTTTGTCTAAAG

>UN00490

CCTTAACTTACGTTTCTTATTAAATTTCTAAACTAATTATTCCTAACAAGTAAACAAGATGCCACGATTCAAGTCAAAATACAAAGCATTCTAGCAATTTACTCTCCACGCAAGATCTAAGATAAAATTCGGAAGGCTCTCAAGGTCCAACAACTATCTCTCAGTAGAATAAGGCAAAGCAGTGACATAGTAAACCCTAGCTCTAAGAGGGTGAAAGGGGAAAAGTAAAAACCCTAGGGGCGTCCTCAAAACCCTGCTT

>UN00491

CTTTTTAAATTTAACTAAACAATAGACAAAGGTAACTTAGTTTACCAAAGAAACTTAGAAAAATAAGATCTTCAAGATTAATCGAAGCTGCTGCGGAACCATCACTCTCGATCTCAGCCTCAGACCGAAACGCAGACAGCGTCCTGAGAAACCCACTGCTCTCGAGGAAGCCCGCAATCGAACTGAGAAGCAACTTCCGATCCTTTCTCCTGCCCCCATCGCCGTCTACTTCTAGGGTTTCGGGCGCACCTGAACCATTTTCTAGGGTTTCTGACGCCTTGCCCCTTCGATTCCTTCTTCTTCTTCTTGTTCTCCTTCTCCATTTCTTTGTTCTTGAGTTTGCTGTCGATTGAGGTCGAGGATTTGGAGTCGAGCATGACCTGACGAGGGACGAAGGGGATTAGGGCTGACGTGGCGAGCGGCTTTGGCGCCCGCAGCATGAGCAGTTGCGAGGGCGAAGGGAGAATGCGAGACAACAGTAGGGTTTAGAAGAAGCTATGAGAACTGGGT

>UN00492

TTGTTAGGATCATATATGGAGGTTCTGTAAATGGTGCAAACTGCAAGGAACTAGCAGGACCAAGGACTAGAATAGTTGATGGATTTCTTGTTGGTGGGCTTACTTTGAAGCCTGAATTGTGGACATCATCAATGCTGCCACAGTGAAGTCTGCTTAAGCGATGGCCTGTTTAGAAAGTTTTGTAAATCGTCGTGTGGTCTTTAATTCTGCAGTTTTTCATTTTGAAGTTACATACTGTAGTATTTCGCCTGAAACTACGAGTTAAAGCGTACAGAAACAGGCTGCAAGTATCAATAAGGGTGTTTTTCTGTAAACATGCTATTCCCGTGTGAAATTCTTCTATCGTAAGG

>UN00493

TATGCCCAAGCTCAGATGGGCAGGAATCGTACAAGACTTCCATTGATACGAGTGAAAAAAACAGCGCGAAGGATGGTGCTTCTCGAGTTGCCATTTTCAGAGATAGGGAAAAGGATATGACTGATCCAGATTATGACCGTAGCTACACCAGGTATAATAAGGGTATCACACCTTCCCATAGTTTTAACTTGGCTCCGTGCAATGTTCTTCAGCCTTCACCTGTGCAATTTCAGGAGTGCATTTCACACTTGGGTCAGTTACCTGCAACTCAACCTTTGGTGAGTTACAGACTGTCAGACCCGCCTATGAGCCCCTATGTTCCAGTTGGTCGCAATCAAACTCCAGT

>UN00494

TTGGGTGGTGAAAAGGTGATATTTGGACGAAGACATGTTGGTATCATGTCAGCAACACCAATAGGAAACTATGGAGTCAGAATAGATTTTGATGACTTGCACAAGACTTGGGATTTACACATGGGACTACTTCTACAATCTGGGCTCCAACAAGTTTACCCTGATGAGAAACTATATCAAAACCCTGAGGAAACATGGCCTTAGCCGAGACCCTCAGAGAAGGAAACCAAATGAGAGCAAAGGCGGAGAAAAAAGTGTTTAAATGCTTTTAGAAGTTTACTCGGAGATCGTCACAGGCATGAATTTTATGCTGCGAAACTTGGTGAACAGAATGCAGATGGAATAATTTTCAGGCCCAATGTATGTCATCGTGTTC

>UN00495

AATCTGGGACTACTAACAAAATTAATCTCCAAAATCATGAATTACATCACATCCACTGAGGTATCATAAACAGTTGCTAACCTGCGCCTCTTTCGTCGTCGGCAGCGTCCCGCCCTCGAGAAACCGATCAACCGGGATATAAGGCCGCCGCAGAAAGTAAGAATTGCCCTTCTTCCCAACACTAATAACAGTATAGTCCAGCCCCAAGCTCTTCAACTCGGCGATCCTCGACTCGGCCTTCTTGATGATCGCGTTATTGAAACCCCCACCAGAGCCCTCGATCCCCAGTGACGACAACGAGCGCGATCTTCTTCACGGGCCTCACCTTCGTGAGGGGAACGTCGATGTCATCGGTCTGCATCTGCTCGTTGATGTTGTAGAGGACCTCGACCAGGGTTTCGGAGAAGGGGCGGCCGCTGACGACGGCCTCCTGGGCTCGGCGGAACCTTAGGACGGACGAA

>UN00496

TATTGAAACGAAAGATTGAAAATTGAAATTATCAAGAGATGAAGAACGCCGGCGTGATCGCTGCCTCCGTCGCCGCCTTTTCTCGGTGGCGATCAGTTCCAAGTCCGATCTCTACGTAAAGCTCCCTTCCAATTCTAAAGAGGGTTCGGGATCGAGAAGGGGAGTTGAAAGCGAGAAATTTGCTCCCAAACTTGATGGGTTGAGATTCATCGAGACGCTGGTGACGACGCACAGATGAGGATGACGTACGTCGGAATTCGCCGTTAAGTTTGACTTTTCTTCGTTTTCGAGGGAGATGAGTTGGCGTTATGTTGGATTTTTATTTTTATTTTCCTTCTTTTTTGAAAATCGAAAAGGAAATTGAGAGATGGTGATTCGCGGTGGTGAGAGCATCTCCAACAGCTTCCCCATTTTATTGGTAATTC

>UN00497

AACCTTTAAAGTACTTCTTCTTCTTCTTCTTTACTTTAACAAACATAAACTGATGTTATTAGAGATTCCTCTGCTTTCCTCTATCCGCAGGCTAACATCACTCAAAGAAAAGTAAACCATAAACAGGAACAAATTGCCAAATCTAGCTTTAATTAAAGTTTACTACTGAAAAAATAAAAAGAAAAAGGAAAATATGAGATCAACTGACTCCTTAAAAACTGCTCTCGTAGCTGCTTATGGAGTTGATATCATGCAAGAC

>UN00498

GTTCTTAATTTGGGTCTATTATTGCGACCTCAGAACAAAGAACTGGATCTGCTTGTTGTGATATTGCCTGATAATAATGGCTCTCTTTACGGGGATCTAAAACGTATTTGTGAAACTGACCTTGGATTAGTCTCTCAATGCTGTCTAACGAAGCATGTATTTAGGATGAGTAAGCAGTATCTTGCAAATGTTGCTCTGAAGATAAACGTAAAGGTCGGTGGTAGAAACACGGTTCTTGTGGATGCTATTTCTAGGCGCATACCTCTGGTTAGCGACAGACCTACGATTATTTTTGGTGCAGATGTCACACATCCTCATCCTGGAGAAGACTCTAGCCCCTCCATTGCAGCGGTTGTTGCCTCTCAAGACTGGCCAGAGATCACAAAGTATGCAGGATTGGTTTGTGCTCAGGCACATCGCCAGGAGTTGATTCAGGATCTGTTTAAAATTTGGCAAGATCCTCAGAGAGGGTCCTGTCTCTGGTGGAATGATAAAGGAACTACTTATTTCCTTCAAGAAAGCAACTGGGCAGAAGCCTCAAAGAATTATATTTTACAGGGATGGGGTTAGCGAGGGACAATTCTATCAAGTCTTACTGTATGAACTTGATGCTATAAGAAAGGCATGTGCATCTCTGGAGCCTAATTATCAGCCGCCAGTGACTTTTGTGGTGGTTCAGAAACGCCACCACACTAGGCTGTTTGCAGAAAACCATGCAAATCAGCATTCAGTCGATAGGAGTGGAAACATTTTGCCAGGCACTGTTGTTGATTCCAAAATCTGTCATCCGACTGAATTTGACTTTTACCTATGTAGTCATGCTGGTATCCAGGGCACAAGCCGTCCTGCGCATTATCATGTATTGTGGGATGAAAACAAGTTCACTGCAGATGGTTTGCAATCACTTACAAACAACTTATGCTACACATATGCAAGGTGCACAAGATCTGTATCTATTGTGCCACCTGCATATTATGCTCATCTTGCTGCTTTCCGAGCTCGTTTCTACATGGAGCCTGAAACATCCGACAGTGATTCCATGGCAACTCCAGCAGCAGGAGGCCGTGGCCCGCCACTTCAGCAGCAACAACGGCCCACACGGGTCCCAGGGGCCGGTAGTATTAGGCCTCTTCCAGCACTGAAAGACAATGTCAAGAAGGTTATGTTCTACTGCTGAGCCCTGCGAACGTCTGATGGCTGCTATTCTCCTGTTTGGTTGTTGGTTTGTTCATCTTTTGGATGTTTACGAGCCTAGTTATGACTTACTTTCTGTTTGACTACCAAAACAGTATATGTTTTTTCTGTACCTCTATGTTACGATAAAGCGGTGAAGTTGGTATATTTATGGATTATAATCTTTTTGCTTTACAACTAATGTGTTTGAGATGT

>UN00499

TTCTTTGTAACGCGGACGAAGCCCTCCCCTTCTCCTGGATCGCCTCCATCTCCCTCTCTCTCTCTCTATATCGTAACGTAACAGCTTCGCGGCGGTCTCTCTCTCCCGCGGAAAATCACTCTGGAGAGATCGCCTCCATCTTCCTTCACTCCCAAATCTTGATCTCATCCTCTCCATCGGAAAACCAGTCGCTCGCAAGAATACCACCAGCTGTTTTCCGGCCATCAGGAGACACGAGATCATGGCGGCGATCTACAGTCTCTTCATCATCAACAAATCCGGTGGCCTCATATTTTACAAGGATTACGGATCGACGGGGAGAATGGACACAAACGATAGCTTGAGGCTTGCGAGCTTGTGGCATTCGATGCACGCTATCTCGCAGCAGCTGTCGCCTGTCGTCGGATGCAATGGGGTTGAGCTCCTCCAGGCTGATAATTTCGATCTCCACTGCTTCCAGTCCCTCACAGGAACAAAGTTTTTTGTTGTTTGTGAGCCTGGGATGCTTCACATGGAAGCTCTGCTCAAAGTAATTTACGAGCTGTACACTGATTATGTTCTGAAGAATCCTTTCTATGAAATGGAAATGCCAATCCGGTGTGAACTCTTCGATCTGAATCTTACTCAAGTAATCCAAAAGGATCGAGTCTCGTTGTTAGGGCGGTAAGTTATTACTGCTGTCTTATTATCTTAATGTTAATTTCAATTTTCCAAATTGGCTTGTACTTCAGACTTATGGGTAACTGATGTTAAAAGGAGGATGCTCGCTTACTAGTCTGTTACATTTTTCTCATTCTTCCAAGAATTATCTGTTTTCATGTTTATTTCCTGAGAAATTTGTACCTTGAGC

>UN00500

TTTTTATTTTACAAACAATACTCTTCTTCACTCACCAAAAAGCTTTACAATGTAATTTACACACAATACAGTAAATAGACTCCGAACCGCCAAAGCAAATTACCTTCGAATAGACAGGGAATACTCTATTATATCTGATGAAAACCAAACACCCAAAAGAAATTTCTAAAACAACTCAAGACCCAGAAACCCTCACATGAGAAACAATTCCTTTTTTCTTTTTTGATGAGAAACAATTCCCTTTTTCTTTTTCTTTTTTATTAACTTTTTAGTTTTTACTTATTCAGAATCACTTGAACTAGAACTCGAGCTTGAATCTGAACCAGACTTGCTGGCTTTCTTCTCTCAGCTTCTTCTTTCTTGATGCACTTTCATTTGTCGCTTCAATC

>UN00501

CTTTCCGGATGAATCTCGCCTGAAGATATCGAAACGCCAGAAATCAAGAGGATGGGATTTCTATTTGAGGTGGGGCATCAAGGGATTGATTCATCAACATTTAGTTTATAACGCGATAGCGACTTCCTTTTGGAGCTTCAAGAGCTTGGTTCAAGTTAAACAGTAATGACAAATTGCCTAGGGGTACATTCTCACATAGTACTCTTTTAACTTCGATGATATTCTGAGTCTTTGGTGGCGAAGATGAGGCTATGCGTTTGCATTCTTACCTAAGAACTTCTCAGAGAGATACCTGCCAGTATGAAAGCCAGCCATATACCAAGCATTCAGAACTGCAGTAAGATCTGTATCTGAGCTGATACTTTGCGATGCATTA

>UN00502

AATAATATGTTCTGTCCCTTTTAGTTTCTTCAACTTTGTATAAAGAATACCATGCAATCAACGTCCATCTTCCATCAACATCATCTAAATAAACACCGCTGCCAAAAAAAATTACATATTCCTTCCATTTCGCGAGCAACCAAAACAATGTACAATTGAATTTCTCTTCTTTCTTTCTTTTTAGTTTTCTCGTGTCATCTAGGCCCTGAAAGCTCGATAGCTTCGACATCAAGGGATGACTCCTCTACGAAGTCTAAATAAGTTGGAGACATTGGTTGCGGCTCTAAAGATGAGGACCGAGGAGGGAATTTGGGAGTGTCCGCCTTGTTTTGGGATGAGGCTTCCCACTTGTCGGACAAAGTACCAATACTAACCTTAACTAAAACAAATTAAACTAAAG

>UN00503

CTTGGGCGAAAAGAAAGCTTGGGAAGTCGCTCAAGCCCCTTTCAAGAACTTGATGATGATGGGCTTCATGATGTGGATGGCTGGAAGCACAGTGCACTTGTTTAGTATTGGTATCACATTCTTCAGCTCTTTGGCAGCCAATAAGTGCCCTTCAAAGTGTGGGGAAAGGTGTGCTTTTGTTCTGTATATTTATTTTAGGTTTATAGGACTATGAATCCATAGTACACAATTACATTTGTATTAGTTATGCCTTTGTTACATGTTTCTTACTGGTGCATACTAAAAATGCAAAGTTGTCATCAATCAAGGTTTGTTAATAGTTGAGGGGTCTTTTTGGTTACTTACAGAAACAGTCATAAGTGAAGGAGGTTG

>UN00504

TCTTTTGGTTTCGGTTTTGTTATTTCTTCGTCTAAAGGTACAACGTGCTGTCTGCATGATCAGCAACAACACCGCGGTAGCCGAAGTGTTCTCACGTATCGACCACAAGTTCGACCTCATGTACGCAAAGCGCGCCTTTGTGCACTGGTACGTGGGTGAAGGTATGGAAGAAGGGGAGTTCTCAGAGGCAAGAGAAGATCTGGCTGCCCTTGAGAAGGACTATGAAGAAGTTGGAGCAGAGGGTGCTGATGATGATGGTGAAGAGGGAGAAGATTACTAGATTGTTGGGAGTTTTTCAGTTTGGGATTTGCTTGTGGTCTCATATTATTTTCCTCCTTTTTGTTGTTGATCTGCAATTTTGTTATAGTTCATTGTTAACTTTTGTAATGTTCTCGTGTAATTTCTAGTATTGTTTTGTTTCTTCCGTAAGTTTTCTTTGTTATTTT

>UN00505

ATTTTCCTCTGAAATCGGACCCTTTTTCTTCTCTCAAAATCCCTATTCTCTCTCTCTCAAAAATATCTCTTTTTCTTCTCTCTCTGAAGCTATTCATCCAAAATGGGCCAACGCAGCGTCAGGAATGGCCGTGGATGACGACTGCAAGCTGAAGTTCTTGGAGCTGAAGGCCAAAAGACTCACCGTTTCATCGTCTTCAAGATCGATGAGAAG

>UN00506

AGTGAAGTGGAGCCATGTTGTATTTGTCAGGAAGAATATGTGGAAGAGGACAATGTTGGAAGACTGGACTGTGGGCATGATTTCCACACTGCTTGCATCAAACAATGGCTAACAATGAAGAACTTGTGTCCAATTTGCAAAACTACTGGCATGAACACATCAAAAAGAAAGATAGATGAGAGGCACATTGCTCCCATCTAACCTCAGATTGAAAGAGAAATGGAAGGTTGAGGCATTAACGAGTGATGGCTTTCTTTGCCCCCCAATAGATTATAGAGAAGCCCATACTGGTTGGATAATACATTCTTGCTTTATTTTTATTTAATATTTGTT

>UN00507

TTTTGGTTTACTTTATTTGTTAAGTTAAGGTTGGGTCTAATTGTACAGAAGCGGTGAGGAGAAGGCCTTACAGTGTTGTGCTATTTGATGAAGTGGAGAAGGCCCATGTTGCTGTGTTCAATACTCTACTCCAAGTTCTTGATGATGGAAGACTCACCGATGGCCAAGGAAGGACTGTGGACTTCACTAACACTGTGATTATCATGACTTCAAATCTTGGAGCCGAGCATCTTCTTGCGGGGATGGTGGGCAAGTCCTCAATGCAAATTGCAAGAGATCGAGTTATGCAAGAGGTGAGACGGCATTTTAGGCCTGAGCTACTCAACAGACTGGATGAGGTAGTTATCTTCGACCCTCTCTCGCACGAGCAACTGAGGAAAGTTGCCAGGCTTCAGATGAAGATGTGGCCGTTCGCCTTGCAGAGAGGGGATCGCTTTGGCTGTGT

>UN00508

TTTGGGTTAGTTCTTTGTCCAACATCGGTGAAGCGAAAGACTAAGACTTTGTCTCTCGCGACCTTCTCAGTGAGTCCACCTCGCTCCATCTTGGCCAGGGTTTTCTCCTTGCTCTGAGCTTGGCGAGCCAGCTTAGCAGAACCGTGACCAAAACGAGCGATGTACTCCTTCATTGAAGCAATCTGCTCCTGCTCCCATCTGTACTGCTTCATTTGATTCTCCTCCAGCTCAGCACGAGTCTGAACATACTGATCATAGTTACCGGTGTACATCTTCAGCTTCTTGCTTTGCATGTGTAATTGGTTGTTTGGTTAGTACTAAGTAAAC

>UN00509

ATTCGGTTTAAATTTTCTAGTTATTCTAATTGGTCGACGAAGAAGAGCGTTGGATCTCTGAAGGCGGCCGATCTCAAGGGGAAGAGGGTCTTCGTTCGCGTGGATCTGAATGTCCCACTCGATGACAACCTCAACATCACCGATGATACCAGGGTTAGGGCTGCAGTTCCCACGATCAAGTACCTCATGGATCATGGCGCCAAGATCATTCTCTCTAGCCATCTCGGACGTCCAAAGGGTGTAACCCCGAAGTACAGCTTGAAGCCCCTTGTACCTAGGTTGTCTGAACTTCTTGGTGTCAATGTTGGGATGGCCAATGACTGTATTGGGGAGAAAGTCGAGAAAATGGTAGCTGCACTACCAGAAGGAGGTGTTCTACTTCTTCGTAAAACGTTAGGTTCTACAAGGAAGAAGAGAAGAATGATACCAAGAATTTAGACAAAAGAAAGAACTAAAGAAC

>UN00510

AAGTTTAAGGAACAATACTACTTAATTACTTGATAAACTAGAAGAGTAGTAAGTTCGTCTATAAACAAAATAGTAGCACAACCATAGTCTACTTATTTCCCATCTTTCAAATGTTCAGGCATGAACAGGATCATGACAGAAGAAAGAAAAGACCAGGAAACAAAGATCAAAAGGGAGGGATTTTACGAGAAAGTAAATCTTAGCCCCCAAATTGACAATGCTACGACTGACAATAATCACAACGCAAGGAAACAGTATAAACATATTACAGAATCAACCAAAATCACAGCTTCAGCAGCAGCAGCAGCAGCAACAACTGCTTCGGCAGCAGCAGCTTCAGCAACTTCAGCAGCAGCAGCAGCAGCAGCAACAACAACAGCTTCAGCAACTTCAGCAACTTCAGCAACAGCAACAACAGCTTCAGCAGCAGCATCAACAGTCAGTTAACCAGTATATGCCAGAGTTGGAGCAATTTTTGAAACTTCAGTTAGAGGAGCAACATCATCTTCAACAGCATCTTCTTGAATAATCTCATAGTAAGCACAATGGTATCCCGAAACCAGCTACTATCTTTTGCCCTTAAGAGATACTCATCACCATTAGCACATTGAATTGAACTCCATGGGATGGCCATAAGGAATTGTAATCTCTTAATTGCCAAAACAGCTTGCTCCCATATGTCTGGATATTTAGCAGATAAATGAACTTCATAAGATATGTGTCCAATGGAAAGGGTATTCTCGATCTGCTTCTGCATATCAGGATCTTTGAGTTAGGGCACAGGAGATTGCAGGCTTTGCGCCTCGCCAAGCTGGCCATTGCCATCGCCTAGGGTTTCTCTCTCCTCAATTTGGGGGAAGAAGAGGACGACGATGTGTTTGCC

>UN00511

TCTTAACTTTCTTTGTTATTTGTTAGGTTGTCTAATTCTTGTTGGTTGAGACCGATCACGAAGGAGGTGAAGAGGAGGACTTTGAAGAAGAACCCCCTCAAGAATCTGAATGCCATGCTGAGGTTGAACCCGTATGCTAAGACTGCAAGGAGAATGGCGTTGCTTGCTGAGGAGCAGAGGGTTAAGGCGAAGGAGGAGAAGCTGGATAGAAAGAGGAGCAAGCTGCCCTTAAGGAGGAAGCTGCTAAGATCAAGGCTGCAGGCAAAGCATGGTACAAGACAATGATCACCGACAGTGACTATGCTGAATTTGAGAACTTCTCTAAGTGGCTCGGAGTTTCTACAGTAGGCACTATGGTCTTCATCAATCTCTCTATCGTTTTGGTCTATTTCTTTAAAATCTTCTTTTGTTGCTTTTAAGAATTTTTGTTCTAGTTATTATCTTAATGCGTACTGAGAGAGACTTTTCGTCTGAATTTTATGTCTTAGGGTTGCTAAAAACATTTAGATTTTGTAGTTGTTGCTTGTGTTGCTTCTTTGGATATTCATAGTTATTCTATGACAACTTCGAC

>UN00512

TTCTTCCCCTAAAAAAATCTCCTCAACGTTTCCCTCCATTTTTCCTAGAACCCTAACCCTAGAAGACTCCAATTCCAGCTCCATGGCGAAAGACGAGGACGAATTCAGAGGCGAGGTCGAGGAGCGCCTCATCAACGAAGAGTACAAGATCTGGAAGAAGAACACGCCGTTCCTCTACGATCTCGTGATCACCCACGCCCTCGAGTGGCCGTCGTTGACCGTACAATGGATGCCGGATCGAGACGAGCCAGCTGGAAAGGACTACTCAGTTCAAAAGATGATTCTTGGGACCCACACCTCAGATGATGAGCCCAACTATCTGATGCTCGCTCAGGTTCAGCTCCCGATCGATGACGCGGAGAACGACGCGAGGCAGTATGATGATGATCGGGGTGAGATTGGAGGGTTTGGATGACGCTAGTGGCAAGGTGCAAATCATTCAGCAGATAAATCACGATGGAGAAGTTAATCGAGCTCGATACATGCCTCAGAATCCTTTTATTATTGCAACAAAGACTGTCAGTGCAGAGGTGTATGTGTATGATTACAGCAAGCATCCATCTAAGCCTCCTCTAGATGGTGCATGCAATCCTGATATGAGGCTGAAGGGCCACAACTCTGAAGGATATGG

>UN00513

GGGAAAAACCCTAGAAAAAACCTTGAGGGGGATCGTCGCCTCCCTCCCCTCAAACCTCAACCATGGGTGGATCTCGAAGAAAACTCAAAAGAAATCGGGCCAAGGTCAAAGTCGCTCTCCCGAAGAAAAAGCCCGGCGTCTTCAAGCCCGCGTTCACGATCCCCGAGCCCCTTCTGTCCTCCTCCGACGAGAAGCGAGAATGGGACGAAGCCGGCAGCGTCATCAGAAACTACCGATCCTTCGGCGTCGTTTCGAACCCTAATCTCCTCGGTGTTCGCGCTCGGACTCCTCAGATTGTCCAGTGCTCTTCGCTTCAGGTCCCGAATCACGACGAAGATCTCGGTTCCATCGATAGCGGCAGCGATCTCGAGAGTGATGATCTGAAAGCTGCACTTGGGAAGAAAAGGAGGGATGGGAAAGCTGCACCTTTGCGACCACTGACAACTATTCAACGTGTTCACATTGGCAGATTGATTGAGAAATATGGTGATAATTACCAGGCAATGTTCAAGGATATAAAGCTAAATGCGATGCAGCACTCCGCAGTGACTCTGAAAAAACTCTGCCAGCGATATTATGCGCGTGGGAAATGCTATGTAAACGTAAAGTAACCTCTCACTGACCCAGGGGTTAAGATTATTATAGGTAGTGCCCAATACTTCATTTCTTTTTTGTATGAGATGATATCAATGTTTTCATTTCGTAAAGCACTGCATCTATTATTGCATTTTAAGATCATCAGCAGTGTAATTTGATGTTCAACTTCTCGGTAATACTTGTAGTAGTATGGTTTTGCTTTGAAAGGACTTTTAAGTAACTTTGTGC

>UN00514

TGAGATGTTCAACAAGTTTCTGTTCTCTTTCTCTTTGCAATTCCTTAATCTTCTCTTGAACTCTTAGCCTCCTTACTTCTGGTGCTAGTGATTCTTCCTCAATTTCTACGGATGCCATGCTTGACTAAAGACCAATTGACCGACATAATCTTCAAAGAATTCACTTCCGAAAAGCATTCCAAATACAGCTGAAGGATCCACCATGGAGTCCTGAGAAAGGCCTTCCTTTCCATGCTTATCATATGCCTCACGTTTCCCAGGATCACTTAAGACCTGATAAGCCTCCCCAAGCACCTGGAATCTATGAGCGGCTTCTGGATCTCCAGGATTTTTTATCTGGGTGCACCAACCTCGCCTTAATGTAATATGCCTTCTTTATGTCGGCAGCAGAAGCATCAACAGTCACACCCAAGATATCATAGTACTCAGTGTCCTTCACCATCGCTCGAAACCCTAGCTAGCCAGAATCGCAGATAGAGAAACGCCAAGATTTAGTCAGTGAGAGGTCTCAAATA

>UN00515

TTTCTCTTATAGCCAAGTGACTTCCAAAATAAACTTAAATCAATTTCAGGGGCTTGTTTCTTGCTCGAGGTCAAAATTGTATCGGAATCCAAAGACTCCTTCAACTATAGAAGACTTCTACCATATCTTTCGGATCTAGCCAAACATGAATCAAGTGGCTTGGAAATTATAGATTCCAAAAATACAAAAGTTGAAAATAGCATTGAGGAAAGGTCTCCAATGTCAAATGACACCCCTTTACCGAGAAAGCATAACAGTCCTGTACTCCTGGTAGA

>UN00516

TTACTAGGAAGTAACAATAGGTTAGGGAACTACCAAGAAGTTGCCAGGTTTTTGCCGAAGCAGTTGTCATGTGTGTTAATAGCTCCATACGCTGTAATATTGACATCGTCTTGGGAAGGTATTTTCATGGTCTTTTTTGGGATATTTACTTGGTACGGTCTGTTTATGTTTGGGTAGTGTAGCCGTTACTGAGATTGTGGCGTCCCATCCGAAGGTTTTCAGTGTGTGGTTATTGATGTATTTATTTCTAATATATTAGTTGATTAGAAACGCATGAATGCGGTTTATTG

>UN00517

TTAATTTTAATGGGTGGCCCAGACAAAACCCAGTAGCCACCAGGTCGAAGAACTCGATCGATTTCCATTAGATATCTCCCACCATAACCAGCCCATTTGATGAGACATCGAGCACAGTGACCCATGTCAAAGGACCTAGATGGATATGGTAACCTTGTGGTACTAAGAACTCCCAGCATAGCTGGCAATCCACGTTCAAGGGCGAACTGTACTTGGGCTTCATGTGTATCTCTTGGTGCCACAGACATAGT

>UN00518

GTTTACCGTTTCTAACTAGGGTCGTATTGTATTCGTTGTACGGCGAGCCAGCTGGCGACGTACGACCAGGTGAAGGAGGGGATAATCGGCAGCGGGGCGATGGCGGACGGGCTGGGGACGCACGTGACGGCGAGCTTCGCCGCGGGGTTCGTGGCGGCGGTGGCGTCGAACCCGGTGGATGTGGTGAAGACGAGGGTGATGAACATGAAGGTCGGCGGCGGGGCGAAGCCGGCGTACGACGGGGCGTTGGACTGCGCGGTGAAGACGGTGAGGGCGGAGGGGCCGAGGGCCCTGTACAAGGGGTTCATCCCGACGATCTCGAGGCAGGGGCCGTTCACCGTCGTGCTGTTTGTGACGCTGGAGCAGGTTAGGAAACTGCTCAAGGATTTCTGATCGCGTGGGAGGGGGTTGGGGTTAATGGATGTACGGTGATACTGGCGACGACGGCGATGATGATGACGAACAAGCTTTATTAGATGCTTACTAGTATTATGTTTTTCTTGAGTTACAACAATTAGGAAAGGAAAGGAAAGGTAAGAC

>UN00519

AATTTCCTACAAATCCAGCAGATAATTTCAAATAATTGTCAAATATAGTTTTCTTAAGCATCCATCATCTAGCTAACATCAGTTCATTTAAGCAGCTAATACAAATTAGTTACATATGGCTCATTACCCGAATTCCAAAACTCTTCTGAACAGTATCAGCATTAAGAATTCCTATTACATGGCTTCCTCCAAGTTACCGCGCTCTGCAGCTCTGAAGACATCGCCTAAATTGACATTTAAACTGTTTCTCATCGTTCTTCTTCCTACTGCTCCATTGCCTGCAGTTCCCTTTCTCATCATAGCTGCGAACTCACTGTAGTCTATTTGCCCATCATTGTTCTGGTCGATTTCTTTGATCATTTCATCAAGATGAACATCACCAAGGCCAAAGTCTTTGCATGCTTGAGAGAGTTCATCAATTGTAATGTAACCACTACCGTCTTTGTCAAAGAAGGAGAAAGCATTTATCAAGTTCTCCTCTCTCTCCAATTTATTCATGTGCACTGTGGCAGCAAGAAATTCACCATAATCTATGGTGCCATTGTTGTCTATATCAGCCGCATCCATAAGAGCTTGGATCTCAGACTCCATGAGGTCTGAGCCCACTTTTTTCAAGCCCTCTTTTAGTTCATCATATGTTATTGTTCCGCTGTTGTCTGTGTCAATCATTTTGAACAATCTCTTTAGGCCCCCTATCTCTTCTTCTGATAACCTTTCTGCTATAACCCGCAAAGCCATCTTCTTTAGTTTGTTCATTGCTGAGAACTGTTTCAGGCGTGACAAAACAGCAGAATCCAAAGGTTTGTCTGGGGCCACTCCAGCATCAACAATCCATGGGTGACAAAGAACTTGATGAGCTGTATATCTTTTCTTGGATCACGATTTAGCATATTGCGTATGAGC

>UN00520

CAAAAAATAAAAACAAAAATAAAACTAAAGAAAAAAGTAACCTTAAGAAAGACCGACTACTAGAATTTTACTACGTTCTGATTTGGTAACATAAAGAGTCGTGAGGCCTCTTCATTAATCTCTAGCAAGATATTCCTAAACGCAGTCCATCCTTCATCACCAGAGCTTCCTGCAGGGACAATTATTGTACTGCGGTTTCTGCTAAGAGATGCTTCTGAAACCTTCAAAAAGCGCCCCCTTTTGTTCTCTCCAACATCAAAATAAAACACCTTAGTATCGAGCGGCAGCTCCTTATTGAACACCTCCTGCTCGTCCGCGTTCACATAGTAATTAAACAAATTGAGGAA

>UN00521

TTTAAGTAAATTACAAATTAATAATAAATAACAATGTTTTAATTTAAAAGACTTGCATACACTTCTTCAACGTATATCCCTACCCTTCGCACCATAGTTGCATGGAACCTGGTTCGTTCCGGTTCACGGGACGGGTATGGGAACGTGGTACGTCACTCATTAGGAAGCGGGTACGAGGGGGTAGGCTCATACGATACGTTAGGGAATGTGGTACGCTTTAGGTTCTATTCTGAAATGCTTGTTGATATCAGTAATGGTAAAAGAACCTTAAAAATCATGTTGCAATTAAGAT

>UN00522

GTGCAGGCGCCCAGCCTACATTGGTGACCTTCAAATTCAATCCCACATCAAAGAAAAATTAGGGCACGAGATGAACGATAAACAATTTACAGGGTTTATTATGCTTGTATTGACATGGATTGCTCTAACATACGCGCTCCGGGTACCAAGAAAGAATTGATACATACAAAATTTAAGGAGCCCCCAGACGGAACATAAAAACAAAACACCGTCAATCAAATTGAATGGATTAATTAAAACTAATTGATACAACTAAACAAATGAGCTTTCTACTCCTGTAAGCTTATACAGCGCGGGGATCTGCCTCGACAATTAGTAGCACCAGAGCAAGACCTTTTTCTGCCAACTGTCAGAGATTTGTTAGCGAAGGAGGGAAAGCATTAGGAAAATGCCTGATAATTCTCTCGCAAACTCACATATGTGCAACCAT

>UN00523

GTTCGTAAATTCAAAGAGAAACCGCTCTCCGGGAAGGAAAAAAAACACCTCAAAAGCTCGGATTTTTTTGTCCTTTTCGATCGAAACCCTAACCCTAGATTTCGTGTCCGATGGCGGGAAAGGGCGAGGGGCCGGCGATCGGCATCGATCTTGGGACGACGTACTCGTGCGTGGGGGTGTGGCAGCACGATCGGGTGGAGATCATCGCCAATGATCAGGGGAACAGGACGACGCCGTCCTACGTGGCGTTCACCGACACCGAGAGGCTGATCGGCGACGCGGCGAAGAACCAGGTCGCCATGAACCCCACCAACACCGTTTTCGATGCGAAGCGCTTGATTGGCCGGCGCTTCAGCGACCCATCAGTGCAGGGAGACATGAAGCTCTGGCCGTTACAAAGGTACAATACCCTAGGAACCCCGGAACGAAAC

>UN00524

ACAACCAAAGTAGGGGAAAAAGTACTAGTAACGTAGAACTTAGTTTCAAATAGATGATTGGCTGAGGGGAGAATTTATCACAACTGTTCAACAACGTGGTGCTGCAATCATCAAAGCTCGCAAACTGTCAAGTGCATTATCTGCAGCCAGCTCTGCTTGTGATCACATCCGTGATTGGGTCCTTGGAACCCCAGAGGGAACTTATGTTTCCATGGGTGTCTACTCTGATGGGTCTTACAATGTCCCAGCTGGGCTGATCTACTCATTCCCGGTGACATGCCGCAATGGCGAGTGGACTATTGTCCAAGGGCTTCCTATCGACGAATTTTCAAGGAAGAAGATGGATGCAACTGCTGAGGAGCTAACCGAAGAGAAGGCCCTAGCATACTCATGCCTCTCTTAAACGCCTCTGCGCTAGTGGATTTTAAATGAAATTGAAGTTTTGTCGCTATGAATGTGACAGCAGGATGCAACTGCATTATTTTCAATAATTTGACGCTAAGAGTATTCCTCAGATTCTGTAATAATTCTTAAATTTTCGTGTGCATTCGGATTCGTGTATTATCCTTGTTTTTCTGCTCAGTGATTGTGTAACAACTTTTAAAAAAGATGGGAGTCAATCAGGGATGGTTCCAATTAATACAATAAAGAAATTAAAATTAAAATAAAAACCTTAAACC

>UN00525

AGTTAAACTAAGACTATCCTTCCATCGTGCTTATTAAAGAATCTTGCATTTTTACATAGGACTAATAGCATAGTCACTACACACAGCTAAACAATATCTACAGATTTGAACAGAAAACAGGCCATCAAAGGCATGAATCGCTAAAGAAAACGCTACGTACAAAAAGGTCCGAAGAAGTCCAAAGATGAATTCAATCCTCGAACCATCTGCTTTAAATTCCCCACCAAATAGTATGTGATTGTAACTCTCAAGTGCCACTTCCTGCCCACATGATAGCAGCA

>UN00526

AATGAGGACGCACAGCTTGTAGCCTACAACTATCTTACTGAGGATGCGGCTCGAGGAAGGAACTTCATTGGGTTACCAATTCATCGGAGGAAAATATGGTTGGAGATTAAGCTTGGATGGGAATCACACAATGTTGGTATCAGTACTAGGAGGAGATGAAGGATGCGAGATTGATGTTTTGATGGGCTCACTAACGTCAAAAACCAAAAACCAGGACTTTGGCATATTTCTCTCTCTCTCTCTCTCTCTCTCTCTC

>UN00527

ATTATTGCTCCTCTGGTTCATTTATTGCAAACTGCTGAATTTGACATCAAGAAAGAAGCAGCGTGGGCCATCTCAAACGCTACATCTGGTGGTACCCATGAACAAATAAAGTTTCTTGTGAGCCAAGGCTGCATCAAACCATTGTGCGATCTTCTTGTTTGCCCAGACCCTAGAATAGTCACTGTTTGTCTCGAAGGACTTGAAAACATTCTGGAGGGGTTGGTGAAGCTGAGAAGAACCAGGGAACTACTGGGAGCGTGAACCTCTATGCCCAGCTGATTGATGAAGCCGAGGGTTTGGAGAAGATTGAGAATCTCCAGAGCCATGATAACACTGAAATCTATGAGAAGGCTGTGAAGATCCTCGAAACTTATTGGCTGGAGGAGGAAGAAGAAGCAATGCCTCAAGACGCTGCTCAAACTGGATTCCGCTTTGGTGGGAATGAGCAGCTTACAGTTCCATCTGGCGGGTTTAACTTTGGTTGAATGGGTCTTTGTTGCTTGAGGTGAGGAAGGAAGCGGGGTCAGTGTCGGGTCAGTTGGGGAAGAGTCAAGAAGAGTCGTGTCATGTTGGTTTGGGTCCAGTTGGTCCAGTCTCAGGTGTGGGTCGAGGTCCGGAGTTGGGGTCAGGAGTCGGGTTTAAGGGGGGGTTGGTACCAATAAAAAAATGAGGATCAATGGTGCTGACGTCTTTTGATTCAAGTTGCAGCAGTGCATGTTCTGCTTCCTAGATCATCGACAGTCGGTCATTTATGCTGAAGGAGGGGAAGCAACATGCTGTGATAAATTTGAGGTAAAATATGTTGCAGGTGATTCTAAATCATTACAGGTTTTTTTCTCCTGTATAGCGTCGAAGTTATTTTTTTAATGTGGGGTTGGTTTATCAATGGGGTTTTGTTTTCGTAATGCTAAAGATACCATATGAGAGTGTATGGTGGTGTATTATGCAAGTTTGGATACACGGTTTTAAGCTTTCGAACAAAGGAAATGAAGTACTTATTTTAGTGTCACC

>UN00528

ATCTTATATAATTCAGTGACTCACATTTCTCTAACTCCTCCTTAATCAAACGTTCATCGTTTTATAGTTACTTACATGAAAATTTCTTAGTACACTAGTTAAAAATACATCATTTTTGTAGGATCATAAATAGTCTAACTGAAAGATATATTGTTTCTATACTACGAAATATAACTATGAGGTCAAAATTTATACAAGCTAAGCCATCCTACAAAAATATAACTTATGTAATGACCGTAGGCTTCTCCCTGCCAGTGAACTCCTTCAGTTTTGATATTGATAGTGCCACATCTATCAATGGCTTCAAAGCTATTTGTGCATCAATGTCATGTTTGGCAAC

>UN00529

TTTTGTTGGTAGTTAATTCTACCTTTAAATTGTAGCCATACGAAACCTTGGTGCCTACATCACTTTATCTTGCTCATGAGATTGAACATTTGGTGATTCCAACAAGAGACTACTGCTTTGCACCATCACTGGCAGATATATGTAAAGCTGTAGACTTCATCCACAATAATGGATCGTCTGGGAAAACAACGTATGTTCACTGCAAAGCTGGGCGAGGAAGAAGCACAACCATTGTTCTATGTT

>UN00530

CTCAACGGACCGAATCGCTCTCCAAACTTCCGGAGTAAATTTTGGAGCATTCTGGGACTTACAGATAATCTCGATGTCAAAAAAATACGTCACAAACGACATCCATGCAGTTCTCAACACCTATGGAGTAGAAGCAGCTAGGCAAACCATCATCAATGAAGTCAAAGGAGTGTTTGATCCATACGGCATTCGCGTAAATACACGGCACTTGAGTTTGATTGCAGATTTTATGTCTTCTAACGGAGGGTATCGGCCAATGAATAGGCTTGGAATGATGCAGTTCAATACTTCGCCTTTTGGTAAAATGAC

>UN00531

ATTCTTGTAGGCACCATTGACAAATGCTCAGCAGGATGAGGAGGGATCTAACAGTGAAGATGGAAATCTGGACAGCCATTCAGTAACAATTGAAGAGATTTAAATAATAGAAGATCTTTAGGAATTTTATGCCTTGATGCAAATCATTGGGACTGCCAGTATAAATTTGTTAGATCTTTTCTGGGAATTTGAAGCATTTGCGCATAGCTTAGGCAATAACAGCTATATGCCATC

>UN00532

CTTAGTTCTAAGCCTTCGCAGCAGCCTCGCGTAGAAGAGACGGCGCCTGGCCGCTTCGCCCTCAGGACACGTGAAGCGCACAGGACTGATACCATAAGAACCAGCCAGAAGAACAAAGAAAACCCAAAAGAATCGCGCGAGAGAGAGAGGGGGAGCGCAGAGGATGAAGATCGTCCACATCCCTTGCTTAGAAGACAACTACGCCTACTTGATCGTGGATGAGAGCACGAGGGAAGCAGCGGTGGTGGACCCCGTGGAGCCGGCGAAGATCATCGCGGCGGCGAACGAGATCGGGGCCGATCTCAAGCTCGTTCTCACCACTCATCACCACTGGGATCATGCCGGTGGAAATGAGAAGCTGAAGCAGTTGGTGCCAGAAATTAAGGTCTATGGCGGTTCAATAGATAATGTTAAAGGATGCACAAATAAGTTGGAGAATGGGGATAGGCTTAGTTTTGGGGCAGATATCAAGATCCTCGCTCTGCATACACCATGCCACACCAAAGGCCATTATTAA

>UN00533

ACGACGTTACCCTAGAAAGTACAATACTAGTAGGACCTTAGGAAGGTAGAGTATGCTTCTATGGTTGCATACATCACATATAAGCAGTAAATAATCTGGTTGACTTGTGATTTGTGATAAAGCAAGAGCAAATAGCTATTGAACTTGTAGAGATTTTTTGTTCCATATTCCACTTTGTAGGCATATTTCTTATTTAGGTAGAATCCCACTATCTGCAACATGTTTTATCTGAAAGAGTTAAATTTTTTTTACATTTCCACTACTGT

>UN00534

GGGTTCGATAAAGAAAGAGAAGAGGGCGGGGAGGAGTTTTCTTATTCGAGTGCGTACAGAGGACGCGAGGACGAGAAGCAGTACGATAGGGATCCAGAGTTCGCTGAGATTCTCGGGAGCTGTCTCGATGATCCCCAAAAGGCCCAATCTAAAATTGAGGAGAGGATAAGGAGGAAGAGGAATAATATATTGCATACCAAGACTGGGTCTGCGACGCCAGTTAAAGTTATCTTCAACAAGTTTGACTTTTCAAACTCCTATATATGGTTTGAATTCTACAATGCGCCATTACCAAAAGATGTAACCTTAATTTGTGATACCATTCGATCTTGGCATATTATTGGACGACTTGGTGGCTGCAATTCTATGAATATGCAGCTGTCACAGTTACCTTTGGACTCCAAAAGACCAAAATATGATGCTATTCAAGGAGCAAATGTCACACCAACTACGTTTTATAATATCGGTGACCTTGAGATTCAAGACAACTTAGCGCGCGTTTGGGTGGATATTGGAACTAGTGAACCGTTGCTTCTAGACGTATTAGTAAATGCCTTGACCTGCATAAGCTCTGACCCGTTCACGCAACAGCTATGTAGGGATCAAGCAGTTGGTTTTTGGCGGCTCAGAGTTTGAGAACTGGAAGGAGAACTTGACCTCAGAGGATGCTGGTTACAGCAGCCACAAGATCTAGCTTATTTCCGTCGAAAGTCCCATCCTGGTCCTAAGAGCAGCTTGAGATTACAGGCTGATGCACTCGCGATCTGAGAGGAGAGCCTTCATATATTGACGCCGCGAACAACACTGGGGAGAGAGTTCAAGTTTTTGGCAGAAAATAGTGGTGCTGGGATTGCTGTTTTTGTGCTAGCTATCCGTATTCATTCTCCTGCAGTTTCGAGGCCTTCATGATTTTCGGCCTGAAAGGGGATTCAACAGTTGTGTATTGGTTTCATATAGGCACGGAGCCCTCTATTGAGCTATAGAGCTTAGTCATGAGCTCTTAATGATCATGTTGCTTAATCTTAATCAGAAATGTAACACTTTCTCAATCTTCATCATGAGTTTGACATCTAGATATACAGCGTGCTTTTATCC

>UN00535

TTTTTGTTTTTCTTTTTGGTTTATTTTTGGTTTAGGTTTCTTTCCTTATTTCCTTTTGTTCTTTCCCTTCTAGTCTACCTTTCCTAGCAGATGTGGATATCCAAGGCAGAGTACGATGAATCTGGTCCTTCAATCGTTCACCGCAAGTGCTTCTAAGTTTTTCAATCATGGCGACGAGTCGTATCTAGCTGTCTTGAGGTGGTTTTTGAGCTTGTTTAATTGAGATTGTTGTGGAGTTTGTTTGAGTTGTATTTGTACTCTATGATCCTTTGGTATGCGTAGACTCTAAAAGGGATTTGAGGGCTGGTATTTTGCCTTCAAATCCTTTGTTTCAATTAGGCTTGTCAAGTTTGGTAGTGTTTTAGTAGAGAGCTTGTTTCTTTTCCTTCGCTATGAGCAGTTGTGATGTTTGTATTCTTGAATTCTAAATTTCTTTGTTTGTATTATTATTAATTAACTACTTATTTATTTCTTTAGTTACC

>UN00536

GGTATTAAGTAAGGTAAAACTAGTACCCTAGTAACCTCTTTATGTATCCTGAACAACCGAGTACCCCCAGCAGAACAATGGAAGTTGCTGATGTTTACTCATTTCAACACCAACGAATCAAACTATGCCTAAGAAATGAGGCCTTGCAAGCTAACTCAGGAACTGTTCTTTGTCGTATAAAAGACTTTGTCTTTTTCTTTTTCTTTTTTTTTTAGCTCAATAAGGTTTGTGCAGAATAATTGATGTATTCTTTCTAGATGCAGTGATGAGATAACATTATATGGATTACTTTGACTT

>UN00537

GGAAATATAAAATGCAGGCATCAATGTTGGAAATATATAATGAAACAATACGTGATTTATTATCATCTAATCGCTCAGGAGGCCTTGATGTCAAGCATGATACAAATGGGAACACATGTGTGCCTGGCCTTACTGTTGTTGATGTCTGCAGCATTAATGAAGTTTCCTATCTCTTACAACAGGCTGCACAAAGCAGATCTGTTGGCAAAACCCATATGAATGAAGAGTCATCAAGAAGTCACTTCGTGTTCA

>UN00538

TTTAGAAGAATGTTGTAAGTCACAATGTTGGGTTTGCACCTTTCAATTCCTTTCATTTTCTCAAAATATCCTGAGGCTTTTAAAAGAGCCTTGGATTTGTCACGAGAATGGAGGTGAGCAGTTATTAGTGCATTGTAAACTGAAGTATCAGGTCTACATCCACTACTTCTCATCTCTGTGAAAAGCCACATGGCCATCCTCGTTTGGCCTTTCTTGCCCATTACCGAAATTAATTTTGAGTATATGCCATTGTCTGCAA

>UN00539

TCTAAGTAAACTAAAGAGACGTCAAGCGTCACAGACGAAAAACAACTGAGAAAGAAATGAAAAGCCCCTTGCCCCCAACTCCAGCTGCTAATTCTGAGAAAAGCTAGTAACGTTTCTGAGGGTTGAAGGACAAGAGTAAATATAAATTGAAGGCTGCTTTTTGTGCGGCAAGGTGCAGGTGAGCTCCATCAACCTGCCTTTGCGGTTTGACCTTTAGCATAGGTTGAGATGAATGATTGAGATGCCATCATCGTAAAAACAGTTTTTGACGTAAACTGGATTTGGCAATGTCAGTAGTAGATTAGTGGATGTGTAAATGCTTCGCCAGCCTATATTTTCCACTATTAGGGTAAGATCTATCTTATCCTACCTCATCAATCATGTAAATATCATCTCTGCTCAATATAATGGATATGTAGCCTGTGGCTCTTGTGATTGACGACGTTAC

>UN00540

TAGGATCGAACTGAGAGGGCTTGGAGGACGCCAGAAGCCAGTGATGAGACCCCACCAACAAGGGTTGAATCTGTGGAGGAGCAATTGCCTGAAAACTGAGATCCCTACAAATGACAGACGAACAATGAGCCTCAGTGAAAGCAATTCAAGCAAATGATGGAAGATTCTATTAATTATGGTTTTGATTACTCCAATGTCAATTTTGTGCTGGTGTTCTCACAGGATATCTGTGGTTTACTTATTGAGTTAGTTTTGTTAGATTAATTTCATAATTTGTAGACATAACAAAAGCAACTTGCACTTTTTTCCCCATGTGTTTTGACATTTTGTTCCGTCATTTTGTACTACAACATTTTTTTAATTTCTGTGATAATTATTTGGAACAAATTTGTGCTGATAATATTTCTCAAATATTGTTTAATTTAAATGTAATTTTATAC

>UN00541

AGTATTTGGTTGTCCTGGGAGACGATCCGAAGCCGAAGCCAAAACCAAGGACGAGAGCGAAAAGGCTTCCTCATGAAAGACCCAAACCCTTCTATAACCACTGTAAGCTCCTCCTCTCTGTCGAATTTGTCGTTGGCGCAGCCCAAAAAAATCGTCGAAACGTGTCTGCTGTTGTTGTCCGTTCTGCTCGTCTCTCGTCTTTGTTATTTGCCGACGATGAGGGTTTCACGATTTCAGATGAAAACGGACGTGGATGGTGGGACGAGGATCGGGAAGGCGTCG

>UN00542

TACTTAGGGTTTCTTCGTTCTAACATGATTATCCCGAAGAAGAATCGCCAGGAGATCTGCAAGTACCTCTTCCAAGAGGGGGTATTGTACGCAAAGAAGGACTTTAACCTGCCAAAGCATCCACTTATTGATGTGCCGAACCTTCAAGTCATAAAGTTGATGCAAAGTTTCAAATCTCGGGAATTTGTGAGGGAGACCTTCGGCTGGGAGCATTACTACTGGTTCCTCACTAATGATGGCATCGAACACCTCCGCTCTTATCTGAACCTTCCATCTGAGATTGTTCCTGCTACCCTTAAGAAGGCTGCAAAGGGACCTCCCCGCCCGTTTGGTTCTGGACCGCCTGGTGAATACGACCCTAAGGGGAACCTTACCTAACGTA

>UN00543

GGTTTACCTTTAATTTGTAACTTAAATTTGTAAAGTGACATGGTAAGTTGTTAATTCTGCAGTGAGAAAACTTAGATCGATTCGCCCACAAAGTGACAAGAACGAGAGACGTGCAGCTTTTGCACTTTGTGGAGCAGATGATATTGCTAAACTTATTAAAGCTTATGAGTTTGAGATCGTAACTCTTGCTTCTCTATCTTCATTAAAGGTTTTAATCCAGGGATGATGATGTTCCGCGTGGGTGTGCTTTTGATGTCGTAATGAAAGTCTCTCTATGTACCTACAGCTCC

>UN00544

TAACCAAAGAACAAGACGAAGACAATACGACTACCCCCTAAACCCGAAACAAATTACAAAAGTAAGAGGGGGGAAAAACTGCAATCTTTCAATTCAAAGCCCTAGAATTCAAGCAAAGATGTCAGATCACGCACAAGAGCAGGAGATGGAGATCGAAGCACTGCAAGCAATTTTGATGGATGATATCGAAGAGATCGAATCTAGTGATAGCGGGCTTGGTACAAAGAATCGCTGCTTCCAGATTACTCTATCGCCTCAAGATGACGATGTGGATGAGGC

>UN00545

CTCACTCCCGATCCCCGATCTCCGATCCCCGATCGCGACTCCTCTCTCTCATTTCTCAGTCTCGATCTCGACTCTCAAGTTCTCTCTCAACGCAGCAGCTTGTTTCCTCCTCTCTTTTCAGTTTCTGATTGGGATTCAAGGCTTAATATGTCAGCAAAAGTATCAACAGTGACGATCGCTGCAGCTATTGCAGGATCTTTCGTGATTGCATATGCTTGTGACACAATAGTCTCTGATAAAAAGATCTTTGGAGGTTCCACCCCCAGCAAC

>UN00546

ACTTCTGAAAGTCGTCTTGAGCAGTTTTCTCCAAGCTTTCCACACCAAACACTCGCGGGAAGAAGTTAGTTTTATATGCAACCCAGAAGAAAATAACTGTCATCACTGCCAAATAACCACCAAGAATGACCCCAGTTGCAAAAATTTCTGAAAGCTTCCAGCTGTCAGGTAACGGAGATGGCTTCACCCTATCCTTTGATATCGTCATGATAGTACCATCATTGAGGATGGCGATAATTAGGACCATGAAGGGTGGAAAGTCAAACTTCCATATGAGTGCAAGAAGCATGAACCCAAGCACTATACGGATGGTAATGGAAACTGCATAGATCGTATAATTTTTCATCCTCTGGAAAATTGCACGACTGGTCAGGACAGCACTTATTATGACACTTAGACCCGGTTCTGTGAGAACAATATCAGAAGCACTTCGAGCAGCATCTGTAGCAT

>UN00547

GATACATAGTTTAATGAAATGAATAATACGGAATACAACGAACGTTTCATGATAATCAACGCTTTCAAGATTTTCATGACAAAATAAACAAATTACATAACAAGATCTGACAACAAAACAACACACGTTTTCCTTGGCCTAAAGCACAACAAAAGAACCGACGATATCACCACCGCAACAACGTACAATCCACTCTCAAGTTCCAACTCAGTGATTAATCCTCCTGCAAATCTTACCTAATCTCTCCCCTCTTCCCAGTCAACGGGTCAACGTTCCCCATCTTAACCATACTCTTTGCAAACTGCTCGAAGAAGGCGCCGTTATCCTCTGCGTACTT

>UN00548

TTTTTTAAAATTACTAAAATTTAACCATCACGTTACTTTCTTTAATTCTAGTTCTAGCAACAAAGGAAATCAAAAGTGTTGCTATTACAGAGCGAAGCTTGAAAGATTCAGCTAATAGAAGTGAGTTTGAATGATTGTGATACAATTTGATCATAAATTCTGACACAACGAGAAGTTTGGATGCAACAGAAGGAAAAGGGGTCCTCGGCCATTTTCTCAGATTGATGATGAACCAGGGCTCTTCACCTTCTCCTTCAGGAACTGCTGAATATGTTCACGCTTCCAGTTGTCAATTCTAATTGATTCAATGTCTTGGCCGTCTTCGTCCAGCAATACAAGCTTGGGTGGGGAAGCATAAGCATAACGAACTTCGACTGGGAATTTCTCTCTGTCTTCTTCAATAAATGCTACAATTTCAGGATAGAACACAAGCTTTCTCATACACACCTCTAAAATTGCACCAGAGAATGTAACCTTACTCATGGAATCATCAGAATCATCAGTGCAACACTTGAGACAATCTGACACCAACTCTTGATCCTTGACGTACTCAGAAAAGGACTTGCAATCGGAGCAGAGAGCGAGACCTGTAAACCCTAGATTCTCGCAGTCCTTGGCGCTCAGCTGATCGCCGAGCACTGAAGAGGAGAAATCAGAGAGCAGCAATAATAATCC

>UN00549

CAATTGGGTTCTCCACAGGGATTTGAAGACATCTAACCTTTTATTGAATAATCAAGTGACTTTGTGGTACAGATCTCCCGAACTTTTATTAGGAGCCAAGGAGTATTCAACTGCTATTGATATGTGGTCATTGGGATGTATAATGGCAGAACTATTAGCGAAAGAACCATTATTTAGTGGGAAGACCGAGTTTGACCAGCTAGATAAGATATTTAGAATGCTTGGCACGCCAAATGAGAAGATTTGGCCTGGATTTGCAAAGTTACCTGGAGTCAAAGTTAACTTTGTCAGGCAACCGCCTCCAGCTATGGGTGATCTTGCTCAGGCTTCCTGGCTTTTCTTGGTGGCACTTTCAGTTAGGTGATGCATT

>UN00550

TGAGCGCAACCCCAGTGTGAGGGCATGCAAACATACCGGTCTGATCAGCTTGAGCTGTTGCATTCATCAACTCCTCCTCCGTAGCCTCCTCAACAATTCCATTGGTAGCCTTGAGGGCATGCACTGCCCGATCAATTGACACAGGGTCACCAATTTGAATAGCAGAGGCAAAGGTTTGGCTGGCAATAACAGGCTTGAAGTCCTTCCATTCAGACTTGTAATAGAGATAGAAGTGGATTAGCATTTGCAGCTTGTGCACACACCAGCCGGGGCACTTTATCAACTAGGCCAAGTTCTCGGCATAGCTCAAACCCTTTGTA

>UN00551

TTGGTTTAAATTAATTACTATATTATTGGTGACATAAATCTTCAAGTACCTTCCTATCCATAGTACTGTTACATCAAAGTACCCAACCAAATAAGCCTATTGCTTATTTGTCAATTGAACTCCATATTACAAACTGAACTCTAGCATATCTTCTCTCAGAATATTTATTTACATTTTATTCGCATTTTGCCTCCCAGTTAAAGGATAGTACATCTCAGCATCAACCCCTTCTGCTCCATCCTCTGGTCATCACTACCACCTTCTTGAGGCTGGATCAGTTTTCGGTTTTGGGGAGAAGCCATTTCTTTCTTCAAACTGATCAAGATATCTTCCATTGTGTATTCCCTATGCCAATTAGCAAGCATTGGGAATAGGCTAGGTTCAACCATTCCAGTTTCTCGGTTCACACATGTCATGTTTATTCGTGATTGGAAACGAACTGCTGGGGGGTTTTGGGATAGTCTTCATCGCAGAAGATCTTCAACTGATATATTCGCCCCTGTGAACAGTATTATGAGGACCAATGATAGTCCCAGTCCATGAACACATCAGTATGTCATCCGAATCGTCCATTCCATAGCTGACAGTTCCATCACCTATACCCTTCTCTCCTCTCTCAAGCTCCTCCAACAATCTGAAACTTCTTGGAATGACAACGCGAGATCCTTCGGCCTCGGAGCCCATTGCGGTGAGAGGAGGTAACCGTCGCCGGGCGGTGACGGGATTCGAAA

>UN00552

ACTTTTTGTTTCTTATTTCTTAAATTTACTTGTTTCTACTAAACTAGAAAGAGAGACTATTTTTGAGGCACTGCGACCGCATTTTCTTACTCTTTCACGGAAGAAATATGCAGTTCATCTTGTAAACAAACTGTTAGATACTGCCACAAAAAAACAATTGGAAGCTTTCATCTCATCTCTTCACGGTCAGGTTTCTTCTCTTCTTCGACATACAGTTGGATCTGCAGTTGTTGATCATGCATACCAGTTGGCAAAAGGGTCTCAGAAACAAAGGCTTTTAATGGAAATGTACTCTACCGAGCTTCAATTGTTCAAGGACTTAACTTTAACAAATT

>UN00553

TACTCTCTGGGATCTTACTTGTCACTATTATACTTCCTTGGTCCAGACTTCTCCTCAAGCGAAAAGGAAGAGGCAGGTCAGCTTGAAGCAGTTTGCTAGCTATTTGTTCCAGAATTTGGAGGGTAATTCCAGCGAGCCCATTCGTGAATTAGATGTGTTGACCCCTACCACCGTTAATGAGTTGGTGGAGAAGGCTGAGGCTCGTTCCTACACCATTGCCAGTGCTGAGGGAGCTTTTCCTGCGGGAACCAAGTTTCGTTCTTTTCTCTGCCGTGCAACAGAAGTCCATTAGGCCCCGGACTCTCCTAGCAGGCTACCTTGCTCTTTGGTTGAAGAAGTGTGTTGTCCCGTACCAGCCTTCAGACGCTCTACCCATCGAGGTGCTCTTCCCTGCAGTTCAGCTAGCTTACAGGAGAGAGCTTTCCCTGCTCCCAGCGA

>UN00554

GTTTTAAATTTTTTTAGTTTAAGGGTTCCCTAAGAGTTGAGAGAGAAGATGGGAAGCATCAAGGTGTTTGGATTGCCAACATCAACTGATGTAGCTAGAGTTCTAACTTGCCTCTTTGAGAAGGAACTTGAGTTCCAACTCATTCGAATCGACACCTACAAAGGAAAGCGTAAAGTTCCTGAGTTTACTACAGGCTACAGGATATCTCTGGTCAAGTGACGTTCAAAGACGAGGAGAAAATGCTCCTCGATTCCAGGGAAAT

>UN00555

CGAGCAAAGGAAACGAAACTAACAAAGAAATAACTACGGGAAGTCTCAGTTCTCTTCACTCAATCAAAATGGGGAAGACTCGTGGTATGGGAGCTGGGCGCAAGCTGAAGACCCACCGAAGGAACCAAAGGTGGGCTGATAAGGCATATAAGAAAAGTCACCTTGGTAATGAGTGGAAGAAACCATTTGCTGGTTCTTCTCATGCTAAGGGCATCGTCTTGGAAAAAATTGGTATTGAGGCAAAACAGCCAAACTCTGCCATCCGTAAGTGTGCCAGAGTTCAGTTGATCAAGAACGGAAAGAAGATTGCTGCTTTCGTGCCAAATGATGGTTGCTTAAACTACATCGAAGGAAAAATAGAATAGAAAGTTTTAGAATTAGAACCGGAAATTTTAAGGTTAACGTTAAAAGT

>UN00556

TACTTAGAAAAAGGAATAACCTAATGTAACTGTGCCACCATGGTGTAAGATCACTACAGGTAGCAAAATGGTTGCAATCTCAGGGATTCAAGAAGGTGTTCAATGTGGCTGGAGGAATTCATGCGTATTCTGTCAATGTTGACCCTTCAGTTCCAACATACTAAGAAGGGCAAATTCACAATAACCCTTATTCTTGTGTGCAAATAAACGTCCCCCTCTCCTTTTTTTTTTTTTTTTGCCAGTAATGCCTGTGGCCTAAAGGGTTATTTCTGTTTGAGTTGGTAGTAAGAGGAGTTTCTTAGTTTTGAAGGAAAATTTGTTAGAAAATGTTGAGAAGTTTCAATATTTAACGATATGTTATATAATCATTGCTTGTGATATTTGTCTCATTAATGGATTCATTCTATTAATTTGAGGTTCAGGATTT

>UN00557

TTTAAAGTAAAAATAAAGAAACAAAACGAAAAGGAACAAAGAACAAAAGGACGACTACGTAAACCTAGGACTTTTTCCACTTTGCAATCAAGTTCTTGTCAGCATATCCCTGATCCAAACAGAATTCATACAGCTCCTTCGAGATTTCCTTCCTCCGATAGTATAAATCATATATGTATCGGCTCTTCTGATGTGAAATTTGAAATATCGGCCACAGCGCTTCGCACTTTCTCTTCCCATCATGTGGATCATTTTCAGCTTCTCTCATCTTTGCCTCCAGCTCTCGAAGTGTAGGCTCGATGAGCTCCCATCCCTCAGGAAATTTGACCCGGCTCGTCTTGATCTTAGGCATGATGATCAATCCTTCAGTGCTTCAACGAAATAGAGAAAGGGCGATGAAGCCGTCTACGGCGTCGGTGATGAACTCCTCTTCGGCGATTGTCTTTACGTTACGGTACGTAA

>UN00558

GTTCCTTAAGTTAAAGTTAATTCTTCTATTCTTCCCAAACCAGTCATATCAAATTCTTTAACCATAGAACTCTAAAATTCATCAAACATAAATTCATCATTGCCAGTAAATATTAAATCATCAACATATAAACTGACAATGAAAATTTTACCTTTAACCTTTGTCTTGATGAACAGAGTTTGTTCGCTGTAGCATTTTTCAAATCTCTGACTCACAAAATGTGCCTCAATACGACTGAACCAAGCTCGTGGAGCTTGTTTAAATCCA

>UN00559

TTTTCTTTGGTTCCTAAGTAGGTCTAGTTAGTCTTTTCTTATAGTACTTGTCCTTGTAATCTTTTTGGATAACGACATCATGCAAAGGATTAAGATCCAAGACGACTATGCTTAGGCTAGCAGTCGTAAAAAAGTTGGCGCAAAATATTGGTAGATCGTATTCTGGTTCAGGTAAGACTGCAAAATCAAGAACCTGCATTGCCGGACTTCCTTCGATCAACAAACTTCGAAGGAGTCTAATTTTGGGCGCCTGAAAAGAAAGTGCATGAAGCAGAGTCCTTCCATCTAGAGCCTTCATGAATTTGAATTTCTCCTGAGAGGGCAAAGGAGTGAGAGTGGTCTTTAGTTGGGCCTGTTGCAAAGCAAAATCCACGAACTTCTTGTAGGAAGAAAGGACCGAAGAAAGAACTAA

>UN00560

AACACCAGGATGATCAGTTGTATATAGGTTTTGTATTGAAAATAATACTGGTCTATCATTTAAAATAAGCTCTTAACAGTAATAACGTAGGCCATCACAATGTGATATGCACTCAAACAATTCTGCTCTCGTAGGTTTAAAAAAAGGGAATTTTTTATGCTTCATTTGAACCTCCTGCACACTTGAATACTTCATGGACTGCCGAAAAATCCAAGTCTCCAAGCCCTAGGCTTCTGGCTTTCTTAAATGCCTCATTGGATGCAGCAGCAATAGGCATTGATA

>UN00561

CTTTGTGCATTGGGTCACGAACGGAGAGACACTTTGTTTTTAATTTCCGGAAATTAGAGAACACACAATTTTTCCAGGCAAAATGATTCGGATTCCCAAGATTCCACCTCAAAAAGGAAAACAGAGAGTGGGATTCAGGCCTTCATCTGTAAGATATCCCTTGACTTTATTCCTTCTGAGCCATTTGTACAATTTGGTTTAATTTGGTTACGGTTCTATCTTATTCCTTGTTGTAGTCTGATAGAAAGTACTATGAGTAGTTCAAGGTATAATTCTTTTGGAGCACTGTTCCGTTGTTTGTGAAATATTACAGTTTCTATCATGTAAATATTTAATATACATATTGTTTGAGCTAC

>UN00562

TAATCATCGTTTTCGAAATCAATCCCATTTTTTGTAAGAATCGCTGCGAAAACCCCTTTTACCCTCCCGAAGGAAAGACAGAAGCATTGGAGCCGTGAGCGGAGTCGACGAAAGAGCTCGAGAACGGCGGCGAACGAGCTCGAGAATGGCAATGGCGGAGGGGGCGAACGAGCTTGGAGGATGGCGCGGTTGGGGTCAATGTCTGGCCACGGTCGGAGGTGGAGCTCGGCGGCCGATTTGGCGTCGAATCTTCGATGGGGGCGTCGGATCTCAAAGGGGGTT

>UN00563

GTAAGTAACTTTGTCTTAGCAAATTCTCAAAAATTAAATTTCATAGATCTCTTTTGAACAACACCATTTCCATTTAACCGTCATAATCACATCAAAGCACTACTGGACCACATTAGGAGCTGGAGACCTGCACACTGTTAGCTTGTTGAGGAGTTGCCGGTTAGGAGTGTAGCATACAATATAACATTGAAAATTCAAGTCATCATTCATGAAACTCATTCACGGAGTGGCCACATTTTCCTTTGCAAGGGGAGCAATTTCCTCGCCATCAACTCCTGGCTCGCTGCCAGCTACATCTAGGCTCAGAAGCATTTGCTCCCATTCCTTTGCAGGACCCTTCCATGAAAGATCTTGGGCCATGCAGCTCTGAATCATCTCTCTGAAGAGAGGTGTAGCGTAGTACTTTAA

>UN00564

GGCACAAACCTTAATAGTTCAAGTTGCAAGCATTTCTTATGGCACTTTCAAAGTAAAAATACACCCCTGAACCATAAACAATGGTTAGAGGAAAAAAAATTCAATGTACTTCTCGGCATTCATGGGTTTAAATACACAGGATGAAACTCCATTTTTATCTTGGTTTCCTTAGTACAGAAACCAAACCAACAATCATGAAACGTTCCTCGCTGTCGTCCTCTGATTCCATCCTCTCTTATGCAGCATCTCTCTTTCTGCTGCCCTCGCGCCATCTCTAACCATCACTCTCCGAGCCATCCTCCCGCTCCCGCCGATCGAAACCCTGACATTCTCCAACCCCGCGACTGTTCTCCTCTTCACAGTCTGCGGCAACGGACGACCTCACCCCACACTTCTTCTTCTGCAGGCTTCTCCTCAAAACGCCTTTCAATTTCTTACTACTACTAGTAGTTTGCATCTGACTGCTATTTACATCCTCTCCTTCACGTGCACCATCTCCTTTCGTGTTGATATCTGGCGAAGGTATCAGCTGCAAAACATTGATGGTCGCATTGCCGGGTATAGGAATCAGTGAGTTGCGACGTGATGATGGCATGTTCACGCTCGGATGAGTTTCTGCAGGCCCGA

>UN00565

GTAAGACACAGAACTGAATCACACTGATGGAGCAAAGAAGGATGAGTCCCACATTAAAAAGGAAAGAATTCATCAAAGTTGCTCCCCACTTCATCGGGTGAATGGTTATAAACACTAGTCTTAGACCAAGCATCATAGCTCCGGCAATTACTGCAATCAAAAGATAGAAACAGAAAAATGCAAAATGCTGCAGTACCTAGCAGACCCCAGACATCATCCAATTTTGATAAAAAACTTCATTTTAAGAAAGGGGAGAGCGGAGGGTTGATCAATGAGTATATAATAATATGTGCAACCCAAGCCACTGAAACAATTAACCCCAAAATTCCCAATATAAGCTTTCCCAGATACCCCAGTACAGTTAATGCCCAAGTAGTCTCTGCCTTTTCTCCTTGAGGATACATCTCTTCCAAAGCCTTCATATC

>UN00566

TTCTAGTTACAATAATTTGACTGCAATTTTGGCTAGCAAGCCTCCTAATGAATCTAAGCTACGCTGCACTAAAAAATAAACAAACCTAAAAATAACAAATAAAAGTTAAGGTAGAATAACCCCAGCACCTTCCTAAAGTACAAAAATGCGTGTAAAATTTTCAAATATAGCACTATGTTTATCCGGTTAATCATCTTTGCGCTTTGAAGAATGTTTGTCCACACTTGCAGAGCTCGTGCACCGTACCGACGAACTTAAG

>UN00567

GTAGTAGAGTACACCGTCAACCTAACCAACAAAGAAGAACTACCAATGGATGTACTTTCAAGAAGATGGCTCCCAAGGCCATCAAAGAAATCAGGAAATTCGCTCAGAAGGCAATGGGTCACAACTGACGTGAGGGTGGACGTGAAGCTGAACAAGCACATTTGGAGCCGCGGGATCCGTAGTGTTCCAAGGAGAGTGCGCGTGAGGATTGCCCGCAAGAGAAACGAAGAAGAGGATGCCAAGGAGGAGCTCTACTCGCTCGTCACAGTTGCCGAGGTACCTCCTGAAGGGCTCAAGGGATTGGGAACAAAGGTCATCGACGAAGCAGACTAAATTAGAGAAAGTGGTTGCCAGTCTTTTAGCTCCCTGTTCCTTTTGATAATTTTAATTAAGTAGAAAAGAAGAACTTAATAAGTTTAAC

>UN00568

TTCTTTCTAACATCCCCTCAGCCGCATCCCGGCTGATCCGCATACCCAAAGAAATACTGCTTCGCTACACCCAGATCCTTCATGGCAAACTCCTTTGCTAACTGCTTCTTCAGCTTGTTTATCTCCTGTAAACACAGTCCTGCAACTAACATGTCATCGACATACATCAATAACACGATATAGCTGGACTCGAACCTCTTGAAGTAACAGCAATGATCTGAATTGCA

>UN00569

GGTTCTTAAGGTTAGTTCTTCTTGTCTAAGTAGTCTATCTAACCCTGATGAAGCCGTTGCCTATGGTGCCGCCGTCCAAGCCGCCATCTTGAGTGGCGAAGGTAACGAGAAGGTCCAAGATTGTTGCTTTTGGATGTTACTCCTTTGTCTCAAGGTTTGGAGACCGCTGGAGGAGTGATGACCGTGTTGATCCCAAGAAACACAACCATTCCGACGAAGAAGGAGCAAGTGTTCTCCACGTACTCGGACAACCAGCCCGGGGTCCGTTGATCCAGGTCTACGAGGGAGAGAGGGCGAGGACAAAAGACAACAACTTGCTCGGCAAGTTTGAGCTATCTGGCATCCCTCCCGCACCTCGTGGAGTCCCTCAGATCACAGTGTGCTTTGACATTGATGCCAATGGTATCCTCAACGTCTCGGCTGAGGACAAGACCACCGGACAGAA

>UN00570

CTAACACCACAAAACCTCAAAATCTCGTCGCGTTTTTGCCCCAGTTTACTTCGATTAGGCTAGATTTTTCAGATGGCGGGAAAGGGCGAGGGTCCGGCCATAGGCATCGACCTCGGCACCACTTACTCGTGCGTCGGAGTATGGCAGCACGATCGGGTGGAGATCATCGCCAACGATCAGGGGAACAGAACGACGCCGTCCTACGTGGCGTTCACCGACTCCGAGAGGCTGATCGGAGATGCGGCCAAGAATCAGGTCGCCA

>UN00571

GATACTGAAAACAAGTTTGTCTCTACTGGGGTTTGCAAAGGAGATTGCAAGAGTAGCTCAAGGGAAATATTATTACTTGCCAAATGCTTCTGATGCTGTCATCTCTGCTGCCACAAAAGAGGCATTATCAGCCTTGAAAAGTTCATGATTTTGAAATTAGTATAGGAAAGGTAGGTAGTGTTTGTATTCTTTGTTAATAACCTTGTTGTTGTATATTTTTGGTCAATCTTCATCACAAGCCTTTGTACAAGCCCTTTTTCCCCCTTATTGCTTGTTTGATGAATAATTGTCAAGTGTTGCGTCTTTTGTCTAGGGTAAGTTGACCTGAAAAACAACAGGAACATTATGAGAAATACAAATTGATCTCCTGGTTTCATCGGAC

>UN00572

GTTCAATTAAAAGAAAACTAAAAGAAAAATAAAACAAAAACGAAACGTAAAACCCTAAAGGTAACAAAACGACCAAGTACAAACAACAATTGCGATGAAAAGTTAACACCGGGCGATCCGAAACAATATCGTTCAACACGTAGACCTAAGCCAATATATTATGGCAGAGGTAGTCAGTTCAAAGTCAACTCGACAGCACAAGCCAAAATCAACTCACCAGTTTTTGATTTGAAACTTTGACGACGGCCAGACGATAAACGCAGCGCCGGAAGAACTTCAGCTATTCATCAAAATAGACTG

>UN00573

AAAATAAACTTTTAAAATTTAAACAAAACAAATAAGAACAAAAGGTAACTTAGTTTACCAAGAACTTGAAAAATAGATCTTCAAGATTAATCGAAGCTGCTGCGGAACCATCACTCTCGATCTCAGCCTCAGACCGAAACGCAGACAGCGTCCTGAGAAACCCACTGCTCTCGAGGAAGCCCGCAATCGAACTGAGAAGCAACTTCCGATCCTTTCTCCTGCCCCCATCGCCGTCTACTTCTAGGGTTTCGGGCGCACCTGAACCATTTTCTAGGGTTTCTGACGCCTTGCCCCTCGATTCCTTCTTCTTCTTCTTGTTCTCCTTCTCCATTTCTTTGTTCTTGAGTTTGCTGTCGATTGAGGTCGAGGTATTTTGGTAGTTCGTAGTCTATTGTACCTGACGAGGGACGAAGGGGATTAGGGCTGACGTGGCGAGCGGCTTTGGCGCCCGCAGCATGAGAA

>UN00574

GTCTTCTACATTATGCGAGATCGGGGGTAGATACCAATTGGATGCAGAGGGATAGTATGCCTCTAAGCTGTTCTGTCAGTGCAAAGATTCAGGATGGCGAGGCAAATAATGGAGATTGTTCATGCTCAAATGAAAATATTCCGTTTATGGATAAATCAATGGGGAACAGAAAATACCGCTACGCAAATCAACCAATTGATTGGCATCAAAAATTTTGGACAGACAGTTTTCCTTGCACAATCACAGGTACACGCCTCGTTTTATTTGCGACGGTTAGTGTGGTTATGTGCTTGAGTGCATGTGTTATGCTTCTCCATCCTATAAAACCTGGAGACATTGCTGTCTCTATTAGAAGGTGCTTGTTCAGGTACCCCTTTCCTTAGAAAATGAGCAGGTGATGTTTTTTGTTTTCTTATTGTTTGTATCTTATGTACATTTTTGTTGGTGTATTATAGCATTTGGTTGTGAGAATAGTTGATCGAGTTTTTAAGTAATAATATTCCTTTTCCTT

>UN00575

GGGAGAGAGATAAATAAAAAAGAAATAAAAGGAAAAAAGGGGAAAAAGCAGAAAAATGGAGGGAGGCGAAGATGTTAGCATGGAGGAGCTCGCCTCGAATCTCTCAACCTACAAAGATCAGCTTCATCAGGTTAGAAAACTTTTGGTTGATGAGCCGGGAAACTCAGAGTATGCAGACATGGAAAAGGAGCTTGAAGAGGTAATTGGTCTTACGGAGGAACTCTTAACCACTGCAAAGCAAAATGAACAATCTGGGCTGATATCTCATCAAGATGCCAATATATCTCCAGGACAGGAGCCGTTAACTGGAACTTCACAGTTTAATACAGGATCAAGTAATCATGAAGGGTTTCCTGTTGGCACAAAAGTTCAAGCTGTTTGGAGCGAAGATGGGGAGTGGTATGATGCGACCATTGAGGCTATTACTCCTAATGGATACTATGTCCGCTACAACGAATGGGGAAACAAAGAGGAGGTGGATCCTGGAAATGTGAGGCAAATTCAAGATGGCACAATTAATCCCTTGCTAGAAGCTGAAAAGGAAGCTGAAGCTACCAGGCAAGCCATTAAACGAAAGATTGCACAAGCAGCAGTTTCTGACTTCCAGTCACGAAGCTTGCCTGCAAAACTTCGCATAGATCCTAATGATCCTGAAGATGTGAAAGCTGCTAAACGTAAGAAGATACATGCATTTAAGTCAAAAGCTCGTTTTGAGCAACTTGAAGTTGTGCAAAACAAACGGCAAAACGCTTGGCAACAGTTTCAGACGACAAAGGGAAAAACAAAGAAGATTGGTTTCTTCTCAGGGCGCAAACGAGACAGCATCTTCAAGTCTCCAGACGATCCAAAGGGTAAAGTTGGAGTAACCAA

>UN00576

AGAGGATGTTTCTGTTATTGCATGGGGCCTTTCCCTAGAGAGGCCAACAATGATTCTGTACGGGATCAATAACATTCGGGACCTCTTTGGACCCAAGGTTGATTTCAATATTATAAAGAACAGCAAGATATGCCGTTTAGGTTGGCAGTAATCTTCTCAGGTGGAGAGAGAGAGAGATCACCTGCGATGAAAATGCCAGGGCCCTGGTCGCTTCAAGTTAGTTCAAGTGAATTTGTTACCTTCACAAATTTTGGAATGTCATGGTACACTTTGAATCCAGGATTTTGTTTGTTCCATTCTCATTGAAATACAATGTAAAATTAAGTATAGGGAGACAGCGACAAACTGAATAGAATAGTGCTTTGTAATATTTATTTAGTTAAATGAAAATCCTCTTAAACTAGAAG

>UN00577

TTTTAAAAAGAAAAAAGAAAAAATAAAACAAAAAAAGAAACTAAAGAACGGTAAGAAAAGAAAAGAAATAAGTAAATAAGAATAATACCAAGAACTAAAGAAAAGTAGAACAACCCTACATCAGGCCTGATGGTACTAAGAAGGCGTATGTGAGGCTCACTCCGGATTACGATGCTCTTGATGTTGCCAACAAGATTGGCATCATATAATTGGAAGAGGGTTTCTAGTTGAGTTTGTTCTCAAATTTACATTTTGTAGTGGTGTTAAAGTTACTGAGGTTGACACTGTTTCATTTTTAAGCTTAAATGCATTTAGTTTCGGTTAATTTGAACTACCTGGACTTGGGCAATACATGTCTTTGTTGGATTGAATCTGTTTCATATTGCTGTATTGTTGATTGCGTTGGTGGCCCTGTTGCTACTGAAGTTTGATATGTATTTTTCTTCTGCTATTTTAACCCTTTAATTTATTGTTTTTAATTTATTTTTTTGTT

>UN00578

TAACCGACCCCGACCGAACAACCCGACAACCGGTAGGACGGACCAACTCCACCACCGGCATTGTCTCCTGTTCCCACTCCGTATCGTTGCCGCTCCGGTGAGTTCTCCGCCGATGCCAACAGCCGAGGCGCCGGCGCCTGCTGCGGGCAAGGCGAAGAAGAGGGCCGATGCTCCGGCCCCTGCGACGAGCGCGGTGGCGGCTGCTCCCGGCATGGCGCCGTCGGGACCCGGCGCTCTCTCGCCTGGGCCTAGCGGCGCCTCCGATGATACGGGCGACGCATCACCTACGAGGAGGACCACCGTTGGGATGATCACAGGGCTTTTCTTGGGCGCTGCTGCCCTATTGGCTGTCTAGACTCTCTGTTATAGACTGAGAACAGGATTTATTCATCTTTGTTTTGTCTCTATTTTGTTCTCTATTATGACTCAGCTCTGGCTTATTAGAACAATAATTTAGAACAAATTAAGTTAAAATAAAGTAAAAG

>UN00579

CTTGGAGGACTGCTTATGTTTATGCTCATCATGGTATATACATAAGCGACGACAAGGTCATACATTTTACACGAGGCCGAGGCCAAGAAGTCGGTACAGGCACCGCCCTTGATTTCCTCCTTGTAAGTTCCGCCCTAAACAACCCGATACCCCCTGCCCTATCTGCACCACCCAACTGTCCGACTCCCACGGTGTAGTCTCCTCCTGCCTCGACTGTTTCCTTGGTGGTGGCGTCCTCTACCGCTTTGAGTACTCCGTCACCCCTGCCCTCTTTTTGGCCAAAGCCCGTGGAGGCACGTGCACTCTTGCCCCCTCGGACACCGACGATGCCGTAATCAAGCGCGCCAATCATTTACTTGTCAACGGCTTCAGATGCTATAATGTCTTCAAAAACAACTGCGAGGATTTCGCTATCTACTGTAAAACGGGTCTTCTTGTGGCCGAAAATGGGTTTCCTTGGGCAGAGCGGGCAGGCGATATCGATCGTGGTGGGAACTTTGCAGTGGTTACGTCGA

>UN00580

GTACCTACCCTACCAACTACAAATTCGTCAGATCCCGACGGTCCCGAGACTCCGGAGTCCCGACCAAAGACTCCCGACCCGTCGCCGGTGTCATATTCGGACTAGTGAGCAGTGAGTGGTAAAGGTGAAGAGCATAACTTATTTTTATAATCGGTCAGTTATTTGCTCAGCCCTTCTATC

>UN00581

AATAATCATTAATTACATTGAGACTGATACACACGAAGCACGTTATTTGAGGATGACATAGTTACAGTGCGTGGGGGGGGGGGGGGGGGGGGGGGTTTGGTCAGGAAGCACAACAAACATAACTATAATCTTCCTCTCAGAGGATGGTACACCACAATCATTCTCTGTCAGCAGTCCCTGTTTTGGCATTGATCCCCGCGCTTCTTGAAGTTCCCACTTGGACATTTTCCTGTTGCGGCACTCTCAATGATCTTAGCTTTGCAGCAGATGACCTAAATGAGTGAATAAGTGGCTGGTTCTCTTCATCATCATTCTCTTCCTGTTCAATCTGCTCTGATGCATTGATAGAATCTTCGTCACTGGAACTTTGTGCTTCAAACAGTTTTTTACCTGGTAAATTAGTCGTCTTTCTTGGAACGGACCTACTTACGTAC

>UN00582

TAGCTTCTACTTTGTACACAACATCCATTCCAGAGATCACTTTGCCAAAGACAACATGCCTTCCATCCAACCAACTAGTAGCTACAGTAGTAATGAAGAACTGCGATCCATTTGTATCTGGACCAGCATTTTGCCATTGATAGAAACCCTGGGTCAGTGTGTTTGACTTTAAAGTTCTCATCGGCGAATTTTGTTACCATAAATAGACTCTCCTCCTCTTCCATCGCCGAGAGTGAAATCACCT

>UN00583

AAACCATCTATTACATAATATTAAAAGTTAAAGAAAAAAACAAAATTAAACAAATAAGACAAAGACAACAATGATTCAACTTCCTAGGAAAAAAAGGACTAAGCAACTGAAACATCAACCTCACGCTCTCTCAAATTACTACAAATAAACCAATACTACTAATTACTCCTTATTACCTCATAATAATGATGATGATGATGATAGTAACAGTAGTAGTACGTAGTAGTAGTACTAATAATAATTTAAAACACAACAGACATTACGTACTAAAGACAATACAATTAACTTAAGGAACTTAACC

>UN00584

TTAGTTCGGGGTAGGCCATGGGAGTTCTTAGAGATGCTGCAACTCAGCTTGGCTCCCTATCATTGAATGATTGAGACCCATATTTATCTGTAATTTGTGGTTAGTTCTTCACATGACTTCTGCCGTTTGTTTTGGGGGACTGTTGCACGAGTGAGTGATATTTTTTGGACAGTTGCTGTTTAAGGAAGGCATCCGAGCCTGCCTGTTGCCAAAGTGTTTTTTTGTGAGATTTGGCATGTTATAGAGTTTTAGAGCATAAATGTTTCGGGTTTCTTTTACGTCTAATTTGGGTCTTGTCTAATTATCATGAGTGTACCAGAAGATATTTCAATTATATGCGTGGTTGCTTAAAAGTTTACCTAGTTTGGGTTACCTGCAGACGCATGCGTACTACAATAAATTAGTCACTT

>UN00585

GTCAACAGACCATAAACTATTTCAACTCATTGACTGATGATACAGGAAGTAACACCAATGTACAAAAGTTGCCCCAAAAAACATATTACAAGCCGCAAAATCTCAACAAACATCTCTTTCGTTCTCAGGTATAAAAACAGGAAGAAAAAAAAAGGGTCATAAGCTATAGAAATAACAACAAAACTAATATACCACTCAAAAAAACTCTGCCATCGGAGAAACTCTACTGAAATGCATTTATTGGTGGAAGGAAGATTTCTACAAACAGTGTACAATAGTATGGGAAAAGGGATAAAAACCTCAATTTTTACACCTGCGCTACAGTTTAACTGCTCTCTTTCGCGTATATCCTCGTCTCTTCAGTGACCTGTGCCCATATGCTTGCATTCTTCTCCGCGTATCCAACCTGGTTTATTACAAATCCTTTAAGCATACCCAGGAAATCATCATGTCTTTCTCTATCAAATCTCTCAAGTTCATT

>UN00586

AATTTTTTACTAAAAAGACCTAAAGAATTTACAAAAACCAAGTATGAACTCGTCTCCTCATTTTGAGAGCTATTCATGAAAATACCATCAACAAGAATCATTTCTCTCCTTCTCTTCTTAGCGGCCGCGGCCACAGGCGACCCGACGCCAACCCTGTGGCCGGAGCGCTTCCACGCGCTCCTCTACATGAACCTGACCGATGCGAAACTACAGATTACGGACCTATGGTACGACTGGCCGAGGGGGAGGGAACCTGAACCTGATTCGGAAGCAGCTGGGGGACCGGCTGTACGATGTGGAGTGGGACAACGGCACGTCCTACTACTTCACGGTGGGACCCGGCGGGGCGTGCCGGACTTTGCGCTTTGAGGTGGGGATCCTGCGGCCGGACTGGCTTGCGGGGTCCACGTACCTCGGCCGGAGGCACACGGACGGGTTCCTGTGTGATGTGTGGACCAAGGTGGACTTCATTTGGTACTACGAGGACGTTCGCACGCGTAGGCCGGTG

>UN00587

CACCATTATCAAACCTAACATTTAGTTAATATTTATGATGTGTTCTAAATAAAAAAAATGGAATGAGTGGATATCTCTAGAGTAAGTCATAACCCTTAGGCTATTTACACAGCTCGACAGGAACCTCTTTAAATTCATTTACATTCACAAGTCTATACAAAACCAACAATAACTTATAGTGTCATCTTTTCCTTTGTTTTCAATTCCATGAACTTCCCCATCATGAGCACAACTTTGTGATTCAGTAATGTCCACATAACTGCCTCGGCTGATCTCTGATTGGAGAATGAACCGTTCAATCCAGCCACCCTCAGTGCCCAGAATAGCAAAATTACTACAAGCACTAATTGTGCAAGCCTTAACATGTGTTTGCTTGTCGGTGGAAGGTGTGGGAATATGTTCTACCTAAGGTACTAAAGTTTCTTTGTTAAGTTACGTA

>UN00588

GTTAGGTTAAACTTAGTCTTACCTTTGTCTAGGTAGTACTAACTTCACGCTTGCTGATCTATCTCACTTGCCAAATGCTCATCGAATCATGACGAAGCTACCACAGCATGCTAAGCTGTTCATGGGGAGGAAGCGGGTGAGCAAGTGGTGGGAGAAAATATCAGGCCGGAAATCTTGGAAGAAGGTGGTTGAGATGCAGAAGGAGTCACCTGTAGAAGCCTAAGGATGATGATGAGGAATAAGGAAACTTGGTTTAGTTGCTCAGTTTGATTTCAGTATTTTGTTTCAGTTTGATGTTGGAAGTAATGCTGGTTATACAGTTAGAACAATGTATTAACTTATTACTTTATTTGGGTTTTTCTACGCTACTTCCACTTTACAAGAGAGTAAAGTGTGGACACCTTGATGAG

>UN00589

TTCTAACCGATGAACATTGAGGCAAGAAAGATGGACACCTGGCTGCTCGAGGTATCTGTCAACGTATACATGTGCAAGGATAAAGCATGAAGGGCTACACTTTGAGTACTTGAATATGCGCTCAGTGTAACTTTGTATACTAAGATCTGGTGCTCTGAGACCATGGAAAATGGTGTCATTAGCTTTCCTCGTCGTAGAATCCATTGAACTCTCATTCTTTTGAATGGTCCTCTTGAGAACTGAGGATAACAGTGACAGAACCTGTGGAAATTCAGAATCTCCTTTCCCTGATTCACTAAGACCCT

>UN00590

CTTTTTAAATTTTTCTTTTTGGTTTCTTTAGTTTAGGTTTGGTTTTCTTAAGTATTTCTAGGTAAGCATATCTTATTTTCCAAGATTTTTCTGAGAAGTATCAGATGACTGGGATGGTTCTTAATGGAAAGGCTGTCTGCTGTATGCACATGGGGCATTTTGATGAAGCTGAATCTTTGTTGCTTGAAGCACTGAACAAGGATGCAAAGGATGCGGAAACTCTGGCCAACTTAGTTGTGTGCAGTCTTCATGTAGGCAAACCTCCAG

>UN00591

CCATGCTATGTCGCCTCGCACGCTCGGACTGCACGCTCATGCTATCTACATGGGCCCAACCTTTTCTTGGGCTGGCTGACCTAATTTTTCTGGCCATGGAGGGCTTTTTGGTGGGAGGAGTCGAGGGAGATTTTCCTTGTTCAGTAGTGTTTAAACTTTCCCATGGCCGAGCAGCCATCCAGCGCTCCATCCAGCTCCAGCCCCATTCAGGGTTGTTCGGATCCAAGAATGTAGGATTTGCTG

>UN00592

TAAACGGTTTAACTAATTCTTATTAGTTTCTATTCACCCAAACAGATTACTCGCAAGTCTGCGGGAAATTCAAAGCCTTACTCCAAGCATTTCTCAAAATAGTCTCATTCATTCATCACGGGTTTGTTCTCAACTTGCTTTTAGGCTTCTGCTCCTTTGGCCTTTGTTCTTCAGTTTTCCTCTTGTCGGAGGGCCCGCCCTTCTGGCTGAGGAACGAGCGGAAGAGGGGGAATTCACGTCGTAAATACAATAGGACGGACGAACGTTAACTAAAGGGTTTTAAAA

>UN00593

AATTTAGGTTAGTAGTCTAGTCATCCAATATTAGCAAATCAGTCATGCTGTGAAGCTTAAATAAGTACCAAGAGTTCTCAGCTGAAACATAAAAAAGAGGAAACACAAATTACTAGCTGTCACTGAAAAAGTATGAAGCCCCAAGATACATGGCAGATATGGTTAGCACAGACCCAAATAATAATTTAAATAAGAATATACTGCTATAGTAAGGCTGCGCTTTAGGCTGCAATTCAATTCAATTCCAAGCTAAATCAAAAAATAACGGTAGCAAAGACTTCTCCAGGCTTCCACAGATTTATGGTTATATTCAAATTTTGAATCCCACAACTCAAGCTTAAAAGATTGGCGGAACCCAAAAAG

>UN00594

GTTATTCTAATTTTATTGCATCTTTATTTCCCGTTTTTACTGTCTTTTACCAGATCACAGCCACAAGATACATGTATGCATCCGTTACTATATGGATACTAAATACCTCTAAATAATAAAATATAGATACCCATATAAATATATTTTGCTAATTTTTCGAAATAAGAATCTAGACCTGTATCTCGAATATCTAAACGCACGTATCAATTAGATTACAACCACACCACTTTTCCAATATAGAAACTCATTGGCAAGTCATTTGCAGCAACTAATTTTACCGCTCAATTCAAAGAGTTTTAAAGCCATCATACATCATGTAACCAAGAAGTCCATGAATCTGTCCCTCCAGAGCTCTCACCAAAGAAAAAATAAAAAAGTTGCAGCAGAAAATACAACCCCGAACGTT

>UN00595

TCATTCTCTTCGGCGTCCCCAGCGCCTTCACCCCTACCAGCAGTATGCAGCACGTGCCGGGTTTCATCTCTAAACCAGAGGAGCTTAAATCCAAGGGCGTTGATGAGTCCTCCTTAGTTAAGACGAATATTTTGTAAGATATTTGTGGAGGGGAAAGGCTTAATCACATATGTACCAAGATATCACCACCTTTTGGATTGCTGCACTATAGCTGAGTATTGGTGTTGATATGCTGCAT

>UN00596

GTTCCCTTAACTTTTTTAGGTTATCAACTCATTTCCTACCTCAACAAAGACAACCACCACCACCACCACCACGGGAGAACAATTTGAACAATATACTAATAAAAGGATCACGCGACTCAGGACTACATCATAAAACAAAAATACATGTCTTTTTCATGTCCCCACATTAACTACCATCCAAGCTTTACACAGGCCCTCAGATGTTACCAGTAAGAAATTTATACAGCTGCTCCCCTGACGCCTTAATATACCAGACCACCACCTGTTTGGTGCAGCATAGCATCAATCTCATCCCCATCCTCCATTTCAAGCTGTAAC

>UN00597

TCTCGCTTTCATTGGGATTGAGCTCGGGTTCAAGATCCTGGTGGTGAAGGGGAGGGTGGCCCTGGGCTTGGGGTTTGGGGGGAGGTTTGGGTGCGGGTTGTTGTTCGCGGTGATGCTGATGGGGGTTGGTTTTTGTCTTGGGCTGGTTGCGCAGACCGTGGTCTACTTTGTTTGCAAGAGCTATCACCATGAGAGCATTGATAAGTCGAGCTTGGCGGATCACTTGGAGGTGTATCTGGGGGAGTATGTGCCGCTTAAAGGGCGGGACGTGCAGATGGAGCAGTACTATGCTTGAGGCTTCTCCGGGGTTGTTCTCCGGTGATGGGTCTCTGTTTGATACGGGGAAGGATTCCACTGCAGACCTCTTAAAGAATGGGCGAGTTAATGGAAATAATGGTGCTCCATGACTTAGGAGGACGGTTTGGTGATAGGCATCTCGTTTGGGTCTTTCTATTTTTGTTCTCTTTGTTCATCTCACTATGTCTGTAATTGTAAAACAGAATATACATGTGTATGTGCTTCATTAATGCATCCTTTTTCTTT

>UN00598

GGTTCCCTTATTTGGGTAAAAAAATTTCACCGTGACCAAAATTCTTATTTTCATAAGTTATAACTCATGATTACTGATCATTTTGACTTGGTGGCAAATAATTTTACGGTGGCCAAAATTAATATTTTTATGAGCTATAACCCATAATTTTTTGTCCACTTTTATTTAAAATGACCAAAGGAATTAAAATTCACGATTTAATTAGCTACAATCCATGATTATTTGTAATTTTTAGTAGTTTTGACACATTAGAAAAATAGTTTTGTTGAGGTCAAAATTCACGACTTCATAAGCTATAACT

>UN00599

TTAGACTTGGAGGTGGTTGATTGCAACAAAAACCAACTTTAAATGTGGTTTATGTATAAATTGACAATGAAAAATAAGTTTTCATATAATTAACCCTATTTTTAAGGTGATATCTAGGAAAAAATCCCTAAAATAAAGTACTGAATCAATACAAACGATATAAATTTCAATACAACAGTGAATTTTAAACATCCTACAAATTATAAGAATTGATTTTTATTTCCCGTATGGTATTTTTAGATGATCCTCTACATGATTTTTTTAGGGTATTTCCAATATTAGTCCCTAAAT

>UN00600

CACAACCCAGATCATGCCCACAAAAGGAACCATGTTTGCCGTCCAACCAGAGGCAACCCCTTTCCTTAGCTGTCGAACCGCTGCCCCCTCTGTTTTGCTGGTAACTTCGGAAGCCTGTGCCCTGCTTAACTTTGCTGATATTTTGTGTGGTCTCAAGATGTAGCTGTGAATGATGTTTGACGTATTATTTATCATCCTATTTTGATATGTGTATATTGCCTTTTTGGCCCCATTTGTTATGTAAGAGTGACATATCATGATGGCTTACAGCAATTGGCTTTTTAGAATAGCGGCCAAGGTTACTTGCATCCCCTTCAGTTTTCTTGGTATTTTGGAAATCATCATAATGTACACTGATGCTTTGGGCATACGTACCTTTTTGAGTAAGAACAGGAATAACTTTCTTC

>UN00601

AATTTTTTTTTACCTATATCTATCACCCAAAAAAAAGCATCATTAAACAAACAGTATAGATATTGATATATAAGAATAATAGTGACGATGATCACGGCCACAAATTAAATCCCTCCGCTGAGGAGGGGAAAAAAGGAGCGAGCAACAAATGAAGGAAGGGTCTCAATGGCATAATGGTAATTACGAGCAAGGTACAGGGGCATATACGTAACTTTGGCTCCCCTCTCTAGCTCCGGCACCGCGTGATCCGCGAGCAGCCGCGGGAGTA

>UN00602

TTTTAGGTAACCCAACGGGGTTAGAACTAACTAGAGGTTAAGTCCGTTGAGATGCATCATGCGGCTCTGCAGGAGGCGCTCCCTGGTGACAATGTTGGGTTTAATGTTAAGAATGTTGCTGTTAAGGATCTTAAGAGAGGGTATGTGGCTTCTAACTCGAAGGATGACCCGGCTAAGGAAGCCGCTAACTTCACTTCTCAGGTCATTATCATGAACCACCCTGGTCAGATTGGTAACGGCTATGCCCCCGTCCTCGACTGCCACACCTCCCACATCGCCGTCAAGTTTGCTGAGATCCTCACCAAGATTGATAGGCGATCTGGTAAGGAGCTGGAGAAGGAGCCCAAGTTCCTCAAGAATGGCGACGCTGGTTTTGTCAAGATGATTCCAACCAAGCCGATGGTGGTCGAGACTTTCTCAGAGTACCCTCCTCTTGGTCGTTTCGCCGTTAGGGACAATAGAAGGACAAGAACGGTTAAGAACTAAAGTT

>UN00603

GGTTTACGTAAAGTTTTAACAATACCAACTAGAAGAGGCCTCTAACGCTATCAGCGGTCTTGATGGGAAGGAACTCCACGGGCGCATGGTGAAAGTAAATTATGCTACTGAGCGGACTGGTGGTTATCGTGGTGGGGGCGGCGGCTACAATGGTGGCTACAGTGGTGGTGGTGTTTACAGTGGTGGCGGCGGCTATGGCGGCGGTGGCGGCTATGGAGGAGATAGCTACAACGCTGGAGGAGGTGGTGACTATGGTAGTAATGCTGATGGTGGTATGAACGACCAGTTCAAGGGTGATAATGAGTATGCAAGCAGGCAATAATTGCACCATACAAAACTTAGTATGAAGTGTTTATGACTGGAACTGCAATATAGAGCATGAGTTAAGTTTTTAATTTGGTAGTAGCGTGGGTTTATAATAGTTAGTAATTAGTACCTTAGTTTTTTAAATTAACAAAGTAAATTTTAAGAAC

>UN00604

GAACCGTTAAGTTAAACGGTTACCAAGACTTAACTTACAAACGAATTACACAACGTCAGGCCACCAAAGATGCTGGTGTCATTGCAGGGCTTAACGTTATGAGGATTATCAATGAACCGACTGCTGCTGCTATTGCCTACGGTCTTGACAAGAAGGCAACCAGCGTCGGTGAGAAGAACGTCCTCATCTTTGACCTCGGTGGTGGTACTTTTGATGTTTACCCTTCTTACCATTGAGGAGGGTATCTTTGAGGTCAAGGCCACTGCTGGTGACACCCATCTTGGAGGAGAGGACTTTGATAACCGTATGGTGAATCATTTTGTCCAAGAGTTCAAGAGAAAGAGCAAGAAGGACATCACTGGCAACCCCAGGGCTCTTAGGAGGTTGAGAACAGCTTGTGAGAGGGCGAAGAGGACTCTTTCCTCCACCGCTCAGACCACAATTGAGATTGATTCCCTTTACGAGGGTATCGACTTCTACTCAACCATCACCAGGGCCAGATTCGAGGAGCTGAACATGGATCTCTTCAGGAAGTGTATGGAACCCGTGGAGAAGTGTTTGAGGGATGCCAAGATGGACAAGAGCACCGTCCACGATTGTCGTGCTCGTTGGTGGGTCTACCAGAATCCCCAAGGTGCAGCAGTTGCTTCAGGACTTTTTCAATGGCAAGGAGTCTCTGCAAGAGCATTAACCCTGATGAGGCTGTTGCCTATGGTGCTGCAGTGCTAAGTCTTGCTTATTCCTTTTAGTTTGGTTTGTTAA

>UN00605

TCCGTCTGCCTAAAATCGCCGTGGCCAAGATCTCATCTCCAATCTCATCAATGAACTCGTCGCCGTTTTCATCTCCGATCTCATCTCCAATCTCATCTTCGAGGCCAAGATCTCATCTCCAATCTCATCACTGAATCTCGTTGGTTTCAGGGTTTGCTCCACCTATCTCTTCTCACGATATCTGATCATATAAGGAGGTTCCATCGTTCGTACTGGAGATTGAAAGACCCCTCCCTTGATTTCAGGTACAGAAATGATGAAAGCCAAGATTGAGATTGATACTGACAAGTTCCTAGTGAGTATTTGACATTGAGGAACAAGTTTTTGGAAACAAATAAAGAATATAGTCTTTGAACGAGAAGTTTTATGTTTTGTAATCAAATGTTGATGTACATTGTTTGAATGTTAGTTCGGTACGATATTTTCTGAGTTGAAATGTTGGTAGTGTGGATTTAATACTTTGTAGTTTTTATACTATACTGATGTATGTTGGCACTTATCATTATGGTTGATTATGCTCAACATAATCACTTTTATATTGTGTATTAAAATTTGACT

>UN00606

AGCAGATGTTGCTATGTGTGCAATGCTGGAGATAGATAGGAACCCTCGTTTGAAGGAGCTTGGATGGAAGTTACTGCTTCAGGTACATGATGAAGTTATACTCGAAGGACCCAGTGAGTCTGCTGAGGTTGCTAAGGCAATTGTTGTCAGGTGCATGTCCAAACCCTTTGATGGCAAAAACTTCCTCAGGGTTGGCCTTTCTGTAGATGCTAAGTGCGCACAAAGCTGGTATGCTGCCAAGTAAGCAAGTGTGGTTCATAATCCAAAGATCCAAACTCGGATCACATCCTTGTGCTCTCAA

>UN00607

ATAAGATCATAATCATAAACTGGAAATTGAGATAGATCAGAAAGATAAACCGTAGTGTTGAAGTTGCACACAATTCTCCACAAACAGCAGGTTCCAAATCAAAAGAAAACCTAATCTAAGAGTTTTTAGAGAGAAGAAGAGAAGAAGAAGAAAGAAAAATCAAGATCTCCAGCCTTTGGTCTCTGGTCAGGTGCTTCAGGGCGTCTCCCTTTTAATTCTGGAAAAAACGTGTTGAAAGATAACTTCAAATATTGCACCAGATCGCATGATAACTAACTGCTGTCATCCAACACGGCTGTGCAGATCGACGCGACCAGGCCGTGTTTAATTCTGTTTCGCTCTGCTTTACTGGGCTTTTCTTCTGTAATCAACACGCCTTGTTGTTTGTTTGA

>UN00608

TTTTCAATCCGTTAATTTACTAAGAAAAGTAAGCAAAAGTATGTTTTTAAAACAATAAGAATTAATAGAATAAAAAAACTCAAAAACCTGTTAAAGTGAAGTTTTGAAATGGTTATGTCTAAGATAGCAATGCCAAAATTCTGTACACTATGTCAAATCAATCATCTAATTAGTGTCCATATGTAGACGTAAAAAAGAAATATTTAACATTACTAAGAATAGACAATTTCATTTTAATTTTAGCTTAATCTAAATTGCTTGATGGATAAATGGGTTGACTAACAAAGATCTA

>UN00609

TTTTTTTTAAGCTCAGCAACAGAAACATTTTTCTTTCATTATTATGAAGCGCTGATCAGTGCCATTCCTACACAGAAACTATAATACTCAACGTTCACAAACACAGCACCCATAAAAAGTATCATACGACCAAAAACAAGCACATCAACCAACAAGCACAAAAACCAACCCTCCCCTCCATCCTCTCACTGGATCTCAACATCAATAACCTTGCGATCAACCTTGGTCTTGGGCACACTCACCAGCAGCACCCCATTCTTGAGCTCAGCCTTAACCTTATCCTTCTCGCAGTTATCCGGCAGCACCATCCTCATGTCGTAATTG

>UN00610

TCTTAACTATTTACTTTTCTTTAAGTAGCTTGTTTTGGTTATCACATAACCCTAGTCCAAATAGCCCCATCACTTGATTTTATGATGCTAATTTGTCTTCTTGTCAATTTATGTAGCATATGTGAGTACTTCAAAACTCAAAATTACACAAACCTCCAATAGACAAGCCATACCAGAACACTACCCCACTGTTACTTTTCTTTTTGAGGAATAGCACATGATGCCTGGGAAATCCTAGATTCAAATTGCTAAAATACCTACATCGTATTATAAG

>UN00611

TCGAGAATTCTTGCAGCATTCTCTCTTTCTTCTTTTAAGCCACTAAGATAGCGGCCATCGAGAGATGCTGCGATTGTTGGGGCAGCGATTGAGAGCTCTGCTGCTTGTTGGACTGTCCATTTTCCAGTCCCCTTCATTCCGGTCTTGTCGAGGATCTTATCCACCAACTCACCTTCACCATGCTCATCCCTAACCTTGAAAATATCAGCTGTAATCTCGATCAAGAAGCTTTCAAGCTC

>UN00612

GTCACAAAAGAGTGAGCGAGCAGCCAGCGCTCTCTGTTGAAAAAAAAAAAAACTTTAGAAGACAATGAGATCCAAGTGTTTGGTTGCAGTTGTCGTGTTGAGTGCATTAGCTAGCGCCGGAGCTCAAAGTCTAGAGCTCGGTTTTTACGACAAGAGCTGCCCAAAGGCAGAGAAGCTCATCTCGGACTTTGTTAAGGAGCACATCCGAAACGCTCCCTCCCTGGCCTCTCCGCTCCTCAGAATGAACTTTCACGATTGCTTTGTGAGGGGCTGCGACGCCTCGGTCTTGATCAACTCATCCACGGGCGCAAACCAGACCGAGAAGGCCGGTGTACCGAACAGAACCCTTCGCGGGTTCGACTTCATCGACCGCGTGAAAGCGCTCGTGGAAGCAGAATGCCCCGGTGTCGTCTCCTGTGCGGATATCATAGCTCTCACCGCCAGAGATGCGGTTGGTGTCATAGGAGGACCCTTCTGGAATGTTCCTACCGGCCGAAGAGACGGCTTGATTTCCCTTGACTTCAGAGGCTAATTCACAGATCCCTGCCCCTTCGTTCAACTTCAGCTCTCTTCAGACGTCTTTTGCTAACAAGGGACTGAACATAACTGATCTAGTTTTCCTAAGCGGAGCGCACACGATAGGAGTTGCGCACTGCTCGTCCTTACAAGACAA

>UN00613

AATGGAGTAACAAACAACTTCAAACAAGGCTCACAGTTTCAAGAGGGTTACAAGAGCAAAGGTAATGTAGCTTACGAACAAAATTGATTCATCACTCAGTTGTTCACCACTGATACGAACCAGAGAGATCTTTTTTCAAAACGAGAAAAACCATTGGAACATATCTAAATCTGGATATGATTATATCGTGGTCATTAAAAAATGGACTCCATCTACTATACACTCAGGCAAATATCGACGTGCATAAGCTGCTGTTGCCATCAGCAACAATGTCATGACTGGACCAAAAAGAACTGCCCGCTTGAAATGCCACTATAGTAGTTTAC

>UN00614

TAGTTACCACGCGCCAAACCCAGTACAAAACTAAGTAACAAAACTAAGGTACATTGAAGAATCATACACGAATCACATCACATATTTCCCCCAATAGAAACCAACAAACACTGCAACAATACATACTAGAAATATTAAAATTGTTAAACACTGAACCTAATAGAAAAAATAGATGGAGGAAATAACAGTTTCCCCATGTTTCCCCTTGTCCTCAAAGAAGATTATGCCCCTCTCGAAATATTTGTTCTTGAACACAAACATATGGCTGCACATGGCTTGAGCTGGATGCTGAATCACGCGCGCTTAAATGCTTCTCCCATTCACTCAAAGCCTCTTCATACGTTGCATACCCTTTGTAGGAGGCTTTTGGGAATCTATCAACTTGAGTATTTTGTAC

>UN00615

AAGGGTTAAGCCGTTGGCCAAAGAAAGTTAAAATTATGTCTACGCAAGCTATAAAAAAATTTAAAAGTTCTAATCATGGAAATAAGAAACTAATAACACAACTAATGCCAATATTACATTTTTTCCCTTCTCACAGGAATCTTATGAACCCAGAAGTTACTAAATTACTATTATTTTTGTAATGAAGTTTTTCTTTTTGAAAACTAATAAGATCAAATAACAGTTCTTCAGCCAGTTTTTTAAGCTTCTGAATTCTATGGATCGCATCCCCGCAATTTCACACGGAATTCTGACTAAATTTTGACACCGACAGAGATTAAGAACGCAACGAAACCCTAAAAATACCATCTTTATAAGAGAAAGGGCCCGATGAAGCTGATCCTTGTAGGTTGAGAGATTCGAAGCGAGCTCCTCGATGCTAACATCTTCGCCTCCCTCCTATTTTTCCCCTTTTTCCCCTTCTTATTTTCTTCTTTCTTTTTTTTTTAC

>UN00616

GTTTTCGTTAGTTCTTTCTTTGTTCTTTCTAGTTTCACTGCGCCGTTCTCTCGTCCCCCGAGCTCCAAAAACCCTAAGCATGGCGGAACAGACCGAGAAGGCGTTCTTGAAGCAGCCAAAGGTGTTCCTTTGTTCGAAGAAATCTGGGAAGGGGAAGAGGCCTGGAAAGGGCGGGAACCGATTTTGGAAGAGCGTTGGGCTTGGATTTAAGGTCCCGAGAGAAGCAATCGAAGGGACATACATTGATAAGAAGTGCCCATTTACTGGTGATGTTTCTATTAGGGGTCTATCCTGGCA

>UN00617

GTTATTTCGTTAGTTTTTGTAGTAAGGTCGTCTTTCGTCAAGAAGTTCGCTCGCAAAGACGAGGAGTATGATTCAATGCTCGGAGTAGTTCGTGAAGTCCCACCAGAGCTCATACTTCCTGATAACATACTACCAGGCAAAGCTCTGAAGCCATCACAATGAATCAATCCATTGGACTGATTTCTTGTATGTTTGTTGTAGTTTGATTTGCATCTTTGAAGAGGACGGTGGTTTTATTTCTTTCCACCCCACAATAATTTTTAAGATCAACCCCAGCAGCTATTGAGCTCGTGAGATTCTTGATCCTTGTTGTTACATTGAAATCGAATATTTCTTGATTA

>UN00618

CCTCCAGCATGCTAGCAGATCATTCACTTTCTGACAGTGAACACAATAATAACTGCCATCTAGTTATATGCACTTCCCACTCTTCATAACACCAGCTCGATCATGATTCAGAACACACTCGATGTGGTAAGATATGCCGCAGGAATCACCCTGATATGGATAATCCGAATTGCAAACCAACCAAAGGCTTGGGTCCTTATTCTCATCGTACTTGTAACATATACCACATGCACACCGTTTGCAAAAAGGACTATCTATGCTTAAAGTGGCTTTGCAAACTAAATTTTCACAGAGACGGACATTTCTTAGAAGCTTCACCGCCATTATCTACTGAATTATTGCTTGTATCAATTGGGAGACGAGAAGGGTGATCTGTCTTTCTCTGTCTTTTGGAAGGGGGTTGATTGTTGGTTGGAGGCAACTGAGGAATTGGGTCAATGTCTTCAGTAAGTTTTCCAGATTTCTTGTCAGACACAACCTTAAAAAGATGTTCTATTAACTTTTGCTTTGTTACTCCGGTGTACTTTCTCTCCTTTCCCATTTCTGCACAAAGGATTTCTATAAGGTCGCGGCGGCTCCATGTTTGTAGCTTTTCAGGTGCAGTTTCTGGCCATTTAGATAATTCGTGAACCAGTTCCCTCTTCTCGTTCATGCTTAGCTTACAGCATTTGGATGGGTCAAGTACAAATCCGGAGAAAGGAGGATCCATAAGCGAGATCTGGTCACCGGCGCGGTCGCATTACCGCTTGCTCAGCAGCCGCCGAGAAAGCAAGGAATCGAGAGGCGACTTCGTCTCTGAGTCCCCCCTCTCTCTCCCCCTTCCTTCTCTGGTCGTTGGGGCTTTTGTTTTTGTAGAGAGGAGGATAATAGCGTAACAGTAAGCC

>UN00619

AAACTTAAAATTTAAAAAGAAAAGAAAATAACCAATAAAGACCCAAAGTTAAAATAATAACTACTAATTTTTCGATACGAGTTCGATCTACAAAATGGAACTTCAAATTCACTCTAAAGATTAAAACATGAAATTCTAAAGAACCCTCAACTGGTTAAATTACTAATCTCAGAATGATCGTTGAGCCGAAATATTGAAAAAATCTCCAGTCCAAATTGGAACCAAATCATCAAATAATCTCTTCATTTAGGTGAGCCATTACAATCTAAAGGCTCAGACCGCTGCCTTTTAGGCTCTCGATCATCCCCTAACCTCAAAGAAACACAGAGGTCTTCTTCATCATCTGAGTTCCTTAATGTTGAGAGAGACTTAAGCTCTTTGTTCTCTTCTTGACTTTTATGAACTGGGGACATGCTAATAAAACTATGTTCTTCTACTTTATTATAGACATCCATTGCAACTTGGAACTCCAAATTTGTGAAATTTTGGGGGGCTGATTTTGAGCCAGTAGCTTCATCACGAGGGAGTTCCTCGATATTGATGCCCAAAGGCTGGTAGATTTCTTTGGTATTTTCCTTCTGCAGAGTGCATAGTTTAGGGGATGATTCTCCGACAGTGGAATCAGCTGCCGCTAAAGCCATCCGCTGCTCTTGAATTGCCTCTTGAAGTCCTTCATTCGTTGTGATGAGATCAAGCTCCAAAAGATCAAGAAG

>UN00620

GGCTCCTTTCTTCTTCTCGAGTTCACTTCATTTCTCGCTCGAAGATGTTGCGCTACACTGCGCCGCTGCTGCTACTGTGCGCCGCGGTACACGTTGCCGTAGCCATCACCGATGGACTACTGCCTAACGGTAACTTCGAGCAAGGCCCCAGCCCGGGCAAACTCAAAGGCACAGAGGTGAAATCCCACGACGCAATCCCAAGCTGGGAGATTTCTGGCTTCGTCGAGTACATAAAATCCGGCCAGAAGCAGGGCGACATGCTCCTCCTCGTCCCCCGAGGGAGCCTACGCCATCCGCTTGGGCAACGAGGCGTCCATCAAACAGAAACTCAAAGTAACAAAGGGGATGTACTACTCCGTCACGTTCAGCGCAGCGCGAACCTGCGCGCAGGAGGAGCGCCTCAATGTGTCTGTCACGCCAGAGTTTGGCGTGCTCCCGATGCAGACCATGTACAGCAGCAACGGGTGGGATTCGTACGCGTGGGGGTTCAGGGCGATGTTCGACGAGGTGGATTTGGTGATTCATAACCCCGGGGTTGAGGAGGATCCGGCCTGTGGACCGTTGATCGATTCGGTTGCCATCAGGACTTTGTATCCTCCGAGAATTACAAACACCAACCTCCTAAAGAACTCCGACTTCGAAGAAGGCCCCTACATATTCCCCAACACCTCATGGGGAGTCCTCATCCCCCCAAACATCGAAGACACACACTCCCCTCTCCCCGGCTGGATGGTCGAGTCTCTCAAAGCTGTCAAATACATCGACTCCGCCCACTTCTCGGTCCCGAGGGGCAAGCGCGCAGTCGAGCTCGTCGCAGGCAAAGAGAGCGCACTCGCCCAAGTCGTCCGCACTGTCCCTGGGAAAACCTACTCCTTGACTTTCTCGGTTGGCGACGCAGCCAATTCCTGCGAAGGTTCGATGCTTATTGAGGCGTACGCTGAA

>UN00621

TCAAACTTGGTCATAGCTTTGCTGGTTGAATATCAAAAACATAGGAAAGCTCCAGACTTCCCTCATATATTTCATGGAACTTATTCAAATATGTGACATTAACATTTTGAAGTATGTCGTCTAAGAATACAACAGACTTCCCCACTTCAATACATTATATTTCAAATTTCAAAACACCAGGTGAGATTATTCAATATAGCTAAGTGATATGAAATTTGAAACATATGAACCCATTTAAGAAT

>UN00622

GAAAATAGGGAAGGGGAATACAATGGAGGAGGAGGAGGAGATTCAGGGCGAAGGTGTGGAGCATGACGGGTGGGCCCTACTGCAGGCCCAAGCACTGGAAGCGCAACACCGCCATCGCCATGTTCGGCGTCTTCCTCGTCTGCATCCCCATCGCCATGAAGTCCGCCGAGCTCGAGCAACGCCCACACCATCCAGTTCGTCCAATCCCTTCACAGCTATGGTGCAAAAACTTTGGCAACAAAGAATACTGAGCACAATGTTTCGCATTACAAAAGGAAAGGGTATCCTTGCCATGTAATCTTGTCTCGTCGGGTGCTTTCTGCACTCCTGTGTGTTGGTTGAGGATTAATACTCTTGTCATGTATGTGTGCGCTTTGGAACACTTATTGTGCTTCATGTGATTTTGAACTTGATTATAAATAATCAGTCTTCCTCTAGAATAATAGGAAAAGGAAAGTAAGAAAAAGGGACAACC

>UN00623

GGGAAAACCATCATATCCATTAATCCCGTCGCAAACGCCTTCTTCCTGTACTTTTGACATATACACCCCTAATTATCCACAGCTCTACCACCAATAAAATAAAACAAAATATAAAAAAAACAAAAGCAGCACCCCCTAAAACTGCGAAACAAAGGGGGGGAAAAAGGGGAAATTTTCACAAAAAAGCCCCCGGAACAAATCCCCAGCTACGAGATGCACCGCAGCGAGTACATGGAGGAGGCGGTGTCCATCGTCGGGGCCTCGTACGTGCGGTGGTGGTCGCTCTCGAGCCCGTAGTCCATCAGCGGGCGGCGCTCGAGCTCGTCGTCGTGGTCGTGGACGAACTCCGGGACCTCGACCTCCCCCCTCCGCACGTGCTCCCACAGGAACTCCAGCCTGTTCACGTACCTCATCTTCCCGTACAGCCCTGCGCTGAAGAGGAACCTTCCAAAAGTGGCAAAGAAAAGGCGAGCCTGGAGCCCAGGATGGACGAAGAAGACGGCCTGGATCCCATCGCGGACAGCCGGCGGCAGCGCCTCGTAGATCGATCGTAGCGCCGAAACGCCGGGGAAGTTATCGGACCGGTTCACGTAAGTGTGAACGTAAACCACCGTGAACGGCCGCCCGCCGATCGCCGGAAAAATCCTCTTCTCTAAGTACCCCTTCAGCGCCTGCTCTCCTCCGGCGGCGCTCAAAACCCTAGCTGGAAAGCATTTTCCGATGATCCTCACGATCCTGCGCCCGCGCTTGTCCGATCCTTGGATCGTGATCGCCTCGAGTTTCTCGAAGAGGAGTTCTTGATCCGACAATGGCAGGCTCGTAATTTTATCGCTGCTCATAGCTTGAATTTCTAAAAGAAGTGATCCGTGGACCGAAATCGGTCCACGGAGTGCATTAAGCC

>UN00624

TTCGGTTAAATTTTAAAAGTTCTATTGGTTTAGTCTACTTGGGTCTATAAAGACGAAGAAGAAAGGCGGAGGAAAGAAGAAGAAAGGTAAGAAAGCTGAGGCCACAGAAGCGGAGCCAATGGATGCAACGAAGGAATGATGTACTACTGAGTGTTGTCAAAGTTCACAGTTCTCTGAGGATTCTCTGGCGCATGAATTTCATACTGAGCCAAAGATGCGAGCGAAGTTATGATAATATTCGACAAAATAGTCGCCAAAGCTTCTGGCAGTAGAAGGTTTGCGCCTTAAAAGTTCAAATGTTTAAAGAGTCACGATGACGAAAAGACTAGAAATTTAGTAATACTAAAATACGTTTAAGAATAAGG

>UN00625

TCCGTCCCGTAGTTTTGAAATTAGGATACCTCTCTGTTCTCTTCTCCTCTCTCTCAAATCACAAATATCCTAAGAAAAAAAAGGAAAAAAAAAGAAAAAGAATCCAAAAAATGCTAAAGAGCAACAGAGGAAAATCGACGGCGAGAAAACCTCTCATCGACATATCAAATGGCGGAAAACCCTCGAGGATTCTCAAGAAGAAGAGTCCTGTCGATGGATTTGATGGTGGTGCTCTCGATCGTCTTCTCCTTGTTCGATC

>UN00626

ACGGTTTAATTTTGTTCTTTATTGTTACCGTCTTTGTTCTATATATAACACAGTACAAATCCGATAACATCACGAGGAGAACAAATAATCGCCAAGTAGTAGGATCACAGGGACCCTTATTACACCGCAAACCAATTATTCAATCTACATAGAACCTAAGCTAAATAAACCAAAGTAAATATGAATCTAGTCCATTTATTCTGCATTAATCATCGGACATTTTTCAGGGGCATTTGGGTCTTTTGCCGTGGGTCCTGAGTCTAGCATAGCAAGGGCAAGAGTTCTTATTGCCGTAAGTCCCTGCAGGAACACAGTGGCACCTCAAACAGCACGTGCCACATGCACGGTGGCAAACGTTCTTCCTCGATGATTTCCTGCATCTCCTCGCGCACGACAAAGACTAGTACTAAATTAACTTAAGTAACTAAAA

>UN00627

AATCAAAGTGAAATTAAGAAATAAATTTTAGTAAAAAAATAAGTCAAAAATACTTAATCACAATATTTAATTTAATCACGTGCATTGCAGGTGCATAAATCCTAGTAGGAATATAAACTTGGAAGTTTCAGCTTTCCCATAATCATGATGGCGAGATAAAAATCAGAAACTGTCATCAACTCATGCCCTTCCCCATCCATAGAAAACGATTAACTTAGTTGTACACCATATATTTCGGAGTCGCGCTAAACTTCTGATAGCCTTTTATTACTTTGTTGACAGCATCGAGAAAATCCTTCTCAGTGACCGTCTTTCTTCGTGCACGGATCGCATACATCCCAGCTTCAGTGCATACGCTCCTAATGTCAGCTCCAGTGGAGTTGGGACACAGCCGAGCAAGCAGCTCAAATCGAATATCCCTTTCACAGTTCATCGTCCTTGTATGGATCTTGAAAATTTGTGTCCGACTCTCCAAATCAGGAAGTCCAAACTCAACTTTACGGTCCAACCGCCCAGGACGTAATAATGCTGGATCTAACGTGTCAGGTCTATTTGTTGCCATAAGGACTTTAATATTTCCTCGGGCATCAAACCCATCTAGTTGATTAACAATTTCAAGCATAGTGCGCTGAACTTCATTGTCGCCTCCCACACCGTCATCGAATCGAGCACCACCAATTGCATCAACTTCGTCAAAGAACACAATGCAAGCCTTTTTGGAACGAGCCATCTGAAAGAGTTCACGGACCATTCGAGCACCTTCACCAACATATTTTTGAACTAGCTCACTTCCAATAACACGAATGAAGCATGCATCCGTTCTGTTGGCTACAGCTCTAGCTAAGAGTGTTTTACCGGTTCCTGGTGGACCATAGCAGAGAACACCCTTGGGTGGGTCAATACCAAGCTTAACAAATTTCTCCGGATGCAGCATGGGAAGTTCCACAACTTCTCGCATCTTCTCTATTTGCTCCTTGCATCCACCAACATCATTGTATGTAACATCAGGCTTCTCTTCTACAGTCATCATTGTAACACTGGGATCAATCTTAGGCGGCAAAGGAATTTGAATTTGATACTTATTCCGATCAACTCCAACACGCATCCCTTCTTCAATATCAGTGGGAGAAACTTTGTCACCTAGTCCAACAACAAACTTGGCGATTTGCTTAACATTTATCACATATTTGGCATCTTCAGTGTTTGGGTTAATAAA

>UN00628

GTAACATCGAACATCAAGTCTCATGAAAAGTTGAAACATGGAAGAACTACAGCATTGAACATTTTGTTATACATGCAGACAATAAAACTGAAGATAAATACTCCTTAATATAATTTACAGATCAAAGAAACCCTCTAAATAGGAGAAAGAAAAACTATTCTACAGACAGACTTGACTTAAAAACAAAGACCATCCCCGCCATCATCTTCGTCATCTATCATATTGTTTATAACTCCTTCGATTATGTTCTTCACTTTAGCCTATCTATAAGGTCAGAACAATAACCAGTTCGAGTATGCTTATCAAGCTTTCTTTCACTTTTGCCTATTTTTCCCTGCAACGCCGATTCAATCTCGTGGCCGCGGTGTCGTTCGGTGGGGGACGGATGAGGAAGAACGCACTTGCAGCGCCGATTCAAGATTTTTGGGGCTTGATGGAGATCGATAGCTCGAGGGAGATCGAGAGCGGTCGGCGAAGATGGGGGATGTCGGCGGAGGAGGAGGAGTTGGGA

>UN00629

GGGTTTTGTTTTCTTAATTTAATTTCTTTGTTAACAAAGTAAGATATGTATTATTAGTGTCATGCATTTTGTATTTTTTAATGAACTCAGACTCTTGTACTGTGAAGTTTTGTTTTCTAACAACCAATTCTTCTGTAGAATTCTAAAAATTTGGTTGTTGCTTATTCTCCAAGAATCTAAGTTCATCAAAAAAGGTTCTTCAAAATTTAATTCAAAGCAGCAAATGTTTAACACAGTTTCATTATTCTCAAGGACCTGATCTGCTTCTTCTAAAAACTCTAACTCTTCATCAAAAATTTCCTCAAAACTCAAATCACACTCTTCTTCTTCTTCTTCTTCTGATCTTTCCTGAATGTAGTTTATTTCTAGAGGCTGATTATTTGGTTGGTCCCCTGGATTAAAAATGTTGAGATCAGCAGTCAAATTCCCAAAGGACGGTTTTATTTGACCAGTTCTACAGTTAATGAGCATTGTTGGCCTTCTTGTTTTGATGATGATTTACAAACAATTATTACTAATGTTGTTTGTCTAGTGTTGTGCATTCCTATGTTTTATAGACAAACAACGTAGTTGGAGGATGGATGGATTGCACCATAACGAT

>UN00630

TAGAACTATATATCTAGATATATAGTTCTTTCTACCACACCAGATTAAATACTTAATAAGATACTTTTGATTCCAGCCCAATCATTTCATTTAAGACTTAGAGTTTGAATCCCTTCTTTCGTTTCTTGAATGATTGATAAGAACTAAGAATTCAAGTTTCATTCAAATTAATCATTTTGGCTGACCGTTTTTACATAGATGATAAGTAAAAAAGCCGTAGGAACTAGAATGAACAGCGCAGTAGCAATAAGTGCGAGAATATTGACTTCCATAATATAATCTTCCTTTTTTTTGTTTCGCAATAACTCGGGATCTAATCCCATAGAGATGATAAATTTGACTCCTGTAAATTCAATGGGATGAATTACATCCTAATGATACTGAA

>UN00631

TAAACTAACTTTACTTACCAAGAACAAATGCACTTATTTACCATCCTTTGCACAAAAAGTCTCAAATGGTTTTTCCTCATTTCTTATCATTTCACTTAATTGGGTAACCACTCATCCATCTCCTTGACTCTCTATTTCTGACACCCAATCTGAAAATAGCTTTAATCTGGAAGTGTGAACCAAGAAAAATCAAATGTTGATGCAAAAGCTTTTAATTTTGCGTATATTTTGATTTGGATGTTTATCTTCCACAGTTACTGGGCTACCCAAGCAAACCATT

>UN00632

TAGTAGGTCTATTAATTATTTTCTCGGCTGAACATTTTTTTTTGTTTGAAGGATATGAAATATGAGTAGGAGCAACAACAACATCAACAACAAATGGTGTCTGACTTAGAGTCACAAGTGAAAGCTATGCAGGAACTTTTTTTAGGTAGCCATCTTCATTGACTCCTCAAACTCGAGAACATGTGCAGGACCAGGCTATCCATGCAAGATTAGGAGAGGGGGTAGCTCGAGTCTTCTTTTGACGATGATTAGAGATGAAACGATGTGCCTTTCCTAGACTTCTATTTTAATTTGTTTTAGATAGTTTCACGTATTTTCCTTTAGAACAATTGGGATATTAACACTTGTTATTAGTGTTGAATATTTGAGGATGGTGGGAATTTTAATTTAATTAAGAAATAAAGTTAAAAATT

>UN00633

CTTGGTTGGTGGTCCTGGGAATTCGAATGAACTGGGAGGGGGAGGGGGGAAATGCTCAAGTCACCATACACCACACCGAGGCTCTCATAGGCTAAAAGCAGGATAGTCCTCCATGAATCCTTCTTTGGATATTTTGCACTTCCGAATTCAGGATCCATTATATTTACTTAAACCCTTATCTTCACTATATCTAGCACAAATCTCACGTTTTCCAAGCTACAATGGCTGCTTCTTGATTATGAGCCACAAGCT

>UN00634

GTTACAACTCTGATGAAGGATCCTGTCATTTTGCCCTCATCAAGGATGTCGGTTGATAGACCAGTAATCCAAAGACATCTGCTCAGTGACAATACTGATCCTTTTAATAGATCACATCTTACTCCAGACATGCTGATACCTGATGTCGAGCTCAAGGCAAAAATTGAAGAGTTTATCAGGTCCCAAGCTTCAAAGAGGACAAAATCCATGACCAATGCTGTGGAAGCAGGAGATTCTTCCAATGATGTGGTCGCTATGGTTACTGAACCCAGTGAATGAAATTCCTATGCTAAAGCTTCCTCTCATTAAGGTATAGCATGATGGTTCCCTGCATTTTAATGTTTTTGTATCCTTTAAAAAGGTGACCTCCCACCAAGGATGGATGTACTTGTACATATTATTTGCCTTTTGGGTTTCCCTTCTGAGAGTATGGGTGGGTTGGCTGATAGGTCTTCAGTAAAGGTGTCTAATTTGTTTTGTATAAACTTGATCCTAGTTGGGAAAGTTCGATGTGTCCATAGCACATAGCAATCTTATCTTTTAATTGTAAGATTGTCCAAGAGTAAATATGCATTTGACGTTTTTTTC

>UN00635

CGGAGAGGAGCTTCCTACTACAACTGCCGCCCTGGGGGCCGCTGCGAATCCGTACACTCGCGGGTGCAGCGCCATTACTCGATGCAGAAGTTAGGTTTTCGAACTCTACTTCTTGTTTGATTTGGGGGTTCTGTGATGTGTTGGTTTTTATGCTGGTTTGGGATTTTGATTCGGTAGAAGTATGTGCTTGTTGTTAGATGATGGTTAGAAGGATGTACGCTGAATAATTTTTTTATTCCATTGAAGGGTTCGTCTAGATTTGACAGGGGATTCATGGGCTGTCGTCAAAATCTCATCTTTCGTGTTGAAGTTGGCTTTTCTCAAAGAAAAAAATTTATTTAAAGCCCAATAGTA

>UN00636

CTAGTTTGGAGGGTAATGTGGTGATCATCACCGGCGGCAACGGCGTTGGCGGCGAACAGTCGGGCCGCGGCCTCACCGATGCCGCTGGCGGCGCCGGTGATGATCACCACCTTGCCCTCCCAACCCAAACTGAGAGAATAAAGAAAGCAAAAAAAACAATGATTAATGCATTCAAATTACGAAACTAGAGGGAAGCTATTACCTTAGCTTTGGCATGGTCCTGTATGTATGTAGTTATCTTCTCATTCACGTCATGTGTTGCTCCTCCGTTGAAATGAAGGGTATAATGTGGAAGCACAGGAAAGTAGCAAACATGTATATGAGGCTTAAGGTTTATATATATTGTTTAATTCCTATTAC

>UN00637

GTTTAAGTTCTTACCTATTTAGTTCTAGGTCTTCTTTATTCTTTGTGACAAGATCTCGTAGCTGGTCGTTTTATCGAACGGCCCGGTCTCTTGCTTGTAGCAGCTTTCTTCATTGCTCAACTTGTTGCTACATTAATAGCTGTCTATGCTGATTGGGGGGTTTGCTGCCATCAAAGGAATTGGATGGGGCTGGGCTGGGGGTTGTTTGGCTTTACAATCTCATCTTCTACTTCCCACTCGATATCATCAAATTCTTAACCCGATATGCTCTTAAGT

>UN00638

ACTTAGTTAGTAACCTAATTACTTTTGGGTTGGTTCGTGGGCTAGCAGGCCCCTGGGGCAAAAGCGTCAACGTGGATGGATACAAGAGTATTGATACAGTTGAGCAAGAGCTTGCTGGTGGTGGTCTGCTGATTTATCAGAGCTTCTCTCAATCGGTGGCAATGGTGGGTTCTCCGAGGTCCACCCTTCACTGCTGGGATGGAGAGGAGTCTGAAGAGGATAAGGAGAAGGATTTGAAGGAGAAGGGAGGATTCTGTGATGATGGTTTCTAAGCTTTATTTAAGGTTTGTGTCGGTTCTCTCGTGTTAGTTTTTGGCTATTGGAGCAGCAAAGCCAGTTTGTTTTTTAGTTCTATGGTGTGTTTGTGT

>UN00639

TTACCTTTACCTTAGTACTTGTCCTAAGTAAGTAGGGTCGTAAAGGTACCAAGGTTGTCCCTGTGACTGGAAAGAGAATCCAAGGGATTCCTCACTTGAGACCTACTGAGTACAAGAGGTCTAGGCTTGCGAGGAACAGGAGAACTGTGAATCGTGCTTATGGCGGTGTTCTCTCTGGTCAGCGCCGTTAAGGAGAGAATCATTCGGGCCTTTTTGGTAGAAGAGCAGAAGATTGTGAAGAAAGTTTTGAAGATTCAGAAGGCCAAGGAAAAGCAAGCCGGCAAGAGTTAAACGAGTAATTAAGAAGGAATTTTGACTTTCATTATGATATGTATGAGTGGCTATCAAGCAGTTGCCATTTCTCTTTAGATAATGTTTCATTAGTTACTATGAACCAATATCTCTTTAAGAAGTTGCGACTTTTTTTGTTTAAGTATTTCTGCTTGGCCGTTCATTACTTGTATTTTGCTTGGATGCGTACTTGAATCGATTCAGAAATCTATTTGGTTGATCTTTACCTATTTTAATTTACAAAAAAA

>UN00640

TCTATGCCTTGAACTTGAATCCTCTCTCCTAGTGTAGCTGCTTCTTGATCTAGAAGACTCTTCCCTCTCCTCAGATTTTCTGTGCCTTGAAAGCGGGTATTCACTGGTTATGTGACTGCTTCTTCGTCTTGGAGGCTCTTCCCTGTCCTCAGATTTTCTATGCCTTGATGATGAACCCTCTCTGCTTAGATTACTGCTTTTCCGTCTTGGGGGTGATGGAATCTGGAAGCGTGACATATATCTTCCCTCAGGAGGA

>UN00641

TTGTAAAGATGTATCGATCCTGAGAGAAGCACATCCTGGAATCTTGTTCCTGCGGAAGTTTCTCGAGGATTCGGCGAGCGACGGCGCCGGTAGTTAGCCGGTAGACGGCGGAGAATTCCGCGAGGACGACGGTCCCCCTCGCCACCAAGGCGTAGAGGATCGCCATTGGAGATCCGTCGATGGATCGATCGAAGAGATCGGACGAGATTTTCTAGGGTTTTGGGGGCAGAAAAT

>UN00642

GGTATCTGTTGCCTTTGATCTCTGGAAAACCTAACATCAACCCACAGTCGAGGGACTTGGCTCTGCAGTTGGTTGAGAGAATTTTGTCAGGACAAAAAGCTCGGTCTATCTTGCTGAATGGTGCCGTAAGAAAAGGGGAGCGCTTGGTGCCTCCTTCTGCACTTGATTTGTTGATGAGAGCAACATTTCCTGCTCAATCTGCTCGAGTCAAGGCTACAGAGAGGTTTGAGGCGATATATCCGACTCTCAAGGAGCTGGCCCTTGCTGGTTCTCCAGGTACCAAAACTACAAAACAGGCATCACAGCAGCTTTTGCCTTCAGCTGTGAAAGCAATGCAGGAAAACAATCTTGAGTTAGCGAAAGAAGCTGCTGATGTATTCATCTGGTGTTTGGTTCAAAATCCAGATTGCTACAAGCAATGGGAAAAACTTTACTTGGATAATGTGGATGCAAGTGTTGCAGTTCTCCGCAAATTATCTGATGAGTGGAAGGAATATTCAATCAAATTCTCTCCTTTTGTTGCTCTGAAGCAGACACTTAAGAACCTAAGGACAGAGAATGAGAAGGCCCTATCGGGGGCTGTTGATGCCAGAAGCCAAGCATCCATTAAAGAAGCCGACAAGCTCTGCAAGGTGATTTTGGGTAGAGTATCCCGTGGCTCTCGATGCATTAAGAATGGATTACTTCTGCTCACGGTTGCAGTTGCTATTGGATTTGCTGCATCCTCAAATTTGGAGTCATGGGACTTGAAGAAGCTCCAGGCTATGGTTAGCAGTTCCCTTCAATCCTTTTGAAAACATGGCTTAAAAAAGATGGCATAAAAAAGAGTAAGAGAGTAGCCAAACTTATGTTCAAAAGTCGGTCTCTAAGAAAGAGTCCATGATAGTTCAGCTCCCTGTAGAGAAAATTAGTTTAAGGAACTGTCTGAATTGTGCAAGTTTGTTGAGAAATAGTTAGGCTGCATAATATGTTGTTAGTTTGTTTTCGGGGATTTTCAAAGTTTATATACCCTAGTTGTTGTGTTGCTTCTTTTCTCTAACTCTGAGATGTAAGTTTTTCCTTAATTTAGTTGTTCCTGCGGTGTGAAAGATTCAATAAAATTTCTTTTCGAATT

>UN00643

CGCAGCCAGGCGCAGGCTCTGGAAGATGCTCTTGATGTTGAGGATCTTGAAGCCGATAAGAGACCAGAAGACCTGATGCTGAGTTATGTGAGCGGGGAGAAAGGGAAGGATAGATCTGACAGAGAGCTCGTGACTCCGTGGTTCAAGTTTCTTTGGGAAACATATAGGACTGTGCTTGAGATTTTGCGCAACAACTCTAAGTTGGAAGCGTTATATGCTATGACAGCACATCGAGCATTTCAATTCTGTAAGCAGTACAAGAGGACCACTGAGTTCCGAAGATTATGCGAAATTATAAGAAACCATCTTGCAAATTTAAATAAGTACCGGGATCAAAGAGATAGGCCTGATCTGACAGCTCCAGAAAGTTTGCAGCTTTACTTAGATACAAGAGTAGAGCAACTCAAGATAGCGACAGATTTGTCTCTATGGCAGGAAGCTTTCCGCTCTGTGGAGGATATTCATGGTTTGATGACCATGGTGAAGAAAACCCCCAAGCCTTCCTTGATGGTTGTATACTATGCTAAGCTAACAGAGATATTTTGGGTTGCGGATAATCACCTTTACCATGCCTATGCATGGCTGAAGCTCTTCACTTTGCAGAAAAGCTACAACAAAAATTTGACACAAAAAGACTTGCAACTGATAGCATCATCAGTTTTATTAGCTGCACTTTCCGTGACTCCATATGACCATAAGCATGGAGCTTCACATTTGGCACTTGAAAATGAGAAAGAGCGCAATTTTCGGATGTCTAGTTTGATTGGATTTAATCCAGATCCTAAAAGAGAGAGTAGAGAACTTCTTTCACGCTCATCGCTTCTCAACGAATTGGCGTCCAAAGGAGTAATGACATGCGTCTCACAAGAAGTGAAAGATCTCTACAATCTCTTGGAACACGAATTCCTTCCATTAGATCTTGCATCAAAAGCACAGCCTTTGCTTACCAAGATTTCCAAGCTTGGCGGCAAACTCTCCTCTGCCTCCTCTGTTCCAGAAGTGCAACTAGCACAATATGTTCCTGCCCTAGAAAAACTGACAACTTTGAGAGTATTACAACAGGTCTCGGAGGTTTATCAGTCGGTGAAGGTTGAGGTGCT

>UN00644

CAAATATATGACCAGTTTGCCTTAACCAAGCCGGCCATGGGAGATGGTTAATCGTCAACTAGGTAACCAAGGGCACCTCTCATCCATCATAAATGCTCCCAGTCCTTCATACAACCACCACCAGATTCATGCCCTCGAAGGATATGACTCAGAGGGCTCTTTTGAGACTGATTCACAACCAAACTCCCCTTTGGAGAGCCCAAAGGAAGATGCCAACAAATGCCCGTTCCTTCCTCAGTCCTGCGCTTCTGAAGGAGAACAACCTACTGAGGACCACCCCGGGTTTCAAGGCCATATCTCCGATGTGGAATTCTACCATCGACCTATGTCTTCAAAAAAGAATAAGAGGCAACCCGACGGACCCCTTAGAGTCCAACTTAGGACCCGAAAAGGGCCTTCAGTGCACAAATGATCTATATTTTCAGCCGGTCATGGTCATGGTCAATGTATTGTTTAGTCTCTAGTGTCCTCTAGTTTTTGTAAGAGTGAGGGCTGTCTCACTCATGAACAGGTCGCACTCAAGAGGAGTGGCGAAATCAGTGTACTTTTTCCTCAATTAATGACATTTTA

>UN00645

GGAAAAAAAAGAAAAAATAGGGAAAGAGCTCACCGACGATCAAGTCGCCTCGATGCGCGAGGCCTTCTCCCTCTTCGACACCGACGGCGACGGCAAAAGAAATAACGAACCCCCTAACCGAAGACTACGGGAATACCTAGATGCGATCCCTCGGCGGTAACCCTACCCAAGCTCAGCTCAAGGAGATCGTGACAACGGAGAGCCTGACCTCGCCCTTCGATTTCCCGCGCTTTCTGGACCTGATGAAGAAGCACATGAAGCCCGAGCCGTTCGATCGCCAGCTCAGGGACGCGTTCAAGGTCCTCGACAAGGACGCGACGGGGGTCGTGTCGGTCTCGGATCTGCGACACGTGCTGACGAGCATCGGGGAGAAGCTGGATCCGGCGGAGTTCGATGAGTGGATCAGGGAGGTCGACGTGGCGGCGGACGGGACGATCAAGTACGAGGATTTCATTGTGAGGATGGTGGCTAAGTGATGAAACCCTAGGATCTGAGATGAGATCGAATCAAGATTGAAGAATGTGACGGTTCCTAATCTTCTTCAGAATTGGTGTTGGTGTGCTTAGGAGTTGATCTGCTTGTTTTATCGTCAAAACGTTTTTGACCTTTGCTTTTTCTATTTAATGTTGAAACCATCTGTAGCCTATTGGTACATTTGTTTTCTTTTGCCTCTTTTGAATTTATGGTGCATTATGGTTGTACCTTTCTCTAATCCTTGGGGGTGTGAGCTAATGTATGAATCTATTGTTGTCTATATTTCTATTTTGAAGAAATGATACT

>UN00646

CTAGTTCTAAGTCGTCCTATCTTTATTAAATATAATACACAAATAACCCTTAATAGTAATATCTGCAATTAGCCTCATAATAAAAGAGATACCTTGTCAACACTTGCGTTCTGCCTGCAAACAATATTGGCACTCCCAATATCTCCTCTTGTCGAAAACTTAACACCTTCTTTAGTCACCGAAATAACAACTGCACCCCAGATTAAAGGAGTTTCAAACAAAATGAAGTTTGAATTTTTCTTCTTCTTCTTTCTTTTGAAACCTGGATTAACTCAGCACT

>UN00647

CGGGCCCCCCCTTTTTTACCAAACCTCACCCACATTTACAAGCAAGAGCATCACAACACACGTGATCACACATCCCTTGATGGCTCGTCTCATCCGCACACAACAAAAAATACAGCTTCACATGATCAATGCAATCCAACGCTATGAACTGAAAGGTATCAGCCCGCAAGCAAGCAACATTTTGATACCTATCTTAAAAAACATGAGACAAAGCATGACTCCAACGTTGTCCTATAATAGTTTGTACCATATTTACCCAAAATGAAATAAAAGGAACAAAAGGATATGAAAGATGTGAAGCCACCCGCGGGCTCATTTGCCTTCTTTCGCGCCACCATGACCAAAGCCTAACCAAATGATACCAAACAAAAAGAGCATGGCAAGCCATAACATTTGCCATATACGGTGACCCGTCATTGATCACAAGCTGACGATAGCTAGCTAGCTGGAGATTTCGATGAAAACGACTAAGAACCTATTTGATGCATATCAACGAGTCAGCATTCAGCAGTCCCTGTCAGATGAAACAGACACCTGGTCCTCATGGGCGTTTCTCGTGACATCGACCTCAGCATTCTTACATACCCCTTCGCCTTTGAGTATTGGCAGCTTTGATTTGGGGGACAGACGCTTGGCTTCCTCGCAAGTGTAGATGTAGATTTTTCTAACCATGGTGCAGAACTCGTGCCAAGGGTCGTCTCCAACCAGCATCATGTCATCTTCATCATCGGTGTAGACAACCTGCCACTTTTTAACATCACTGGTTAGCTCCCCTTCAATGCTGAACATCTCTTCCAGCTTGTGGAGGAGGTCCTCGTACCCATCAAATCTTGTCAAGTCCACTGCTCTCCCAACTGCCATTCCATGCATGTGAACTTTGGTGCAGCTTCGTGATTGCCTGCTCTGAGTTTCGTGA

>UN00648

CCAAACCAAGACGACGGACGACAACAAGTCTCTATTCAGCCAAAACCAAGTATTGATCTCTCAAATACCATGCAAATCAAAGCATGACGTATGTACATAACATTAGTATAGACCGAAGTATCCATAAGGTAGTAAGGCAGCTACCGATAATTTATATATACATCGCAGACTCGCCATATACATGGACAAAATCTCACCCACAAAACGGGGCATTGTCTTGGTATCTGTACAGTGATAGTACACCCACAACCACTTGCCATGAGAAAAGATCAATATACAACAAAAAC

>UN00649

AAGTAGGCAAAGCTAGGCTTATTCATTAACATAGAGTAAGATACAAACACGGCTAGCATACTTGCGAACTAAGGTTTACAGCTCTTACGGGAACATCATATGATCATAACTCAAGCACAAAGAATAAACATTGATGAAGGATGATTTTAGACCAAATTCCTTTGGATAGGACTGAGAGAGACCTCTCTCATTTCCTCGACGAAGGAGACAGCACTCTCGTGCGGTACTCTCCGAGAGCCCAGATCGGAAAGATGTTCCTATATGCGGAATAGCTGATCATGCAATTTCTATTGAAAAACCCCCATTATTTCTTGTTGAGGGAATTCACCATTCTCCATCTGCATATTTATCAGCGCTTTTGCTGCAC

>UN00650

ACGATAAATAATCGATGTTTCCTGTGTAATTGTCTGCAACTGTTCGTTCAGATGAATGAGTTGATAACTGACGGTCAAATGTGATCTTTACAAGAGTACTATCCCGAAATAGTTAAATCCTGGAATGTTTCGGTAGATGGAAACAGGAAACTCTATTTTTCTTATCAAGTGAGAAGGGCAGAAGAATCAGAGAA

>UN00651

CTCAAGATGAAGCGGCGGCGGAGGATGGAGGGTGTGGTGATGCTGCGGCGGCGGCGGCGGCGGATGGTGGTGGTGGTGGTAAGAGGGAGGGGCGCCCACGCCGGGGATCGGGGGAGGAGGAGGGTAGTAATCCGTGGACCGAATTCGGTCCACGGCTCCATACAAGTATTAAGCAAAAAGGATAGAGAAGATGGCCGGAGCCAAGTCCCTTTTTCTTCTGTGTTTCCTCCTCGCAATTGCATCTTCCCAAGCTGAACAACCTTGTAGCTCCGATCCTTGCTTCCGGCGCTGCCGTCTTTTTAAAGAGGATTGTGCGGAATTTTGCCACTGCAGACTCGTAACATTTTTACCTAATGAGCTGGCAACAGAGGTAGAGCTGGGCCGGAAGGCGAAGATGATGGAAGCGCTGGCGGCAACACAGATGGAGGTCGCCCACAATCGTGTTGAAGCTGAAGCTGAACTGAAGGAGATGATGGAACAACTTATCGGCTTGAACCACAAGTTGACGGGCAGCTGTAAGTATAATCCTTGCTACCGTTTGTGCCGCGCCGCGGGCGGCAGCAAAGCAGCCTGTGGGACTAAGTGCAATTGTACCCAGGTCACGGCTGAGCTGGCAGCAAGCACTGATGACGAAGCATCCAAAGCCATTGTAGTTAATCCTTGCTAGCGTGACGCCGTGTCGTTCCACTCGGCGCTCCCGTGTTTATGTTGCATCGTGTGTTACTTACTTAATTTGCAGGTTGCGGTTCGCTTTGTCTGTTCGAGTCTGTGATGTTGTCGAGTCAGGGTCTGTTTGTTTTGAGTCAGCGCAACTTCTGTATGGTTGTCGATCGGGTCGTACGATGAACTTATCATCTTATCTCTGTATCCTGCTAAATAAATGGAACCGTATGCGTAAAAATAATTAAAAAA

>UN00652

TTGTGGGCTCTCCTTACTTGTGTTCCTCTACCGATGGTGATTGGGAGATTCTGAAGGCTGTGACAGTTGCTGTTGCCTCTATAGGTTTGTGTCTCATGTCGATCATTAATTTCGCTACAGCACAGATTGGATCCATGCTTCTTGTTCCGATGTGTCTAATGGTTCGACCTTTTAAGAGAAGGTCTCAGGTGGCTCTGAGTTTGAGATTCATAGTTCTTGCTTTTAACCTAGCATTTGGGTTATTAGGATTTCCGCCTGTTGGGTTGATGATGTGGAAAGGGCTGTTTGAGGGATTTGCAAAGGTTAGCGTTGGAGATTTCTGGGATCAGGCAGAGTTTCTTTGG

>UN00653

AAAAAAGTTACTGAAATCAAAACTTCGCCTTGATCAACAAGTCACGAGTTGTTCCAAATTATACTATTAACGACTCTAAACATTTGATAAGACATTCTCATCTACCGAACCAAACTAAAACAAATAGAAGGATCTCATCTACTGAACCAAAACAAAACCAGATAAAAGAGAAGACGCAGCACAGCAGCAAGCAAAGAGTACTTCACTTCTTCTTTCCACCCTTGGCAGCATCGCCAGCTTTTGTCTTCTTAACTCCGCGGATTTTCTTTGCTCTGTTCTTCCTTTCTTTCATCTGCTTCCTTGATTTCTCAACCTTGGTAGCGAGTCCATTCCTTAATTGTAGTACGTATTACTTTGGGTTCTTTCTTTAAACTTTTTCTTTTA

>UN00654

GTAACTAACAAAAACTAAGACGGGAAAATAGACTAGACCCCAAAGGAACAAAGGACTAATCAAGAGGTTCCTTGTGAGGAACATTGTGGAGCAGGCTGCTGTGAGGGATGTGCAGGAGGCCTGTGTCTATGATGGATACACTCTTCCCAAGCTGTATGCTAAGATGCAATACTGCGTCTCTTGTGCCATCCACTCCCATGTGGTGAGGGTGAGGTCCCGCACTAACAGGAGAAACCGTGAGCCTCCTCAGCGCTTCAGACGCAGGGATGACGCAGCAAAGCCTGGTCAAGCTCCCCGACCCGCTGGAGCTCCTGTCCCAGCTCGCCCATGATGTTTGTTATTGTTACTGGATTCCTAGTACTCAGATAACCCCTTTAGTTTGGAGATAATTTTGCTTGAGACACAGACTGCTGGGTTGCTACAAGAATGTTTTGTTTTTACCCGTTGCCTTAGGTTTTATGTACTTCAGATTTTGCATGTGGTAGCTCTTCACTCTCCATTGAGTTACTTTGTATTCAGTGTATTTGGGATTTCATCAGTATGTCACTTATTTGTCTTTTATTCTATTATTTTTGTTTTTTCTTTGG

>UN00655

GTTTGTACGAGGATTTAATGCCGGTGATGGCGGATAAACTGGAGGCCGACGCGTTCGTGGAGGAGCTCTGCGGTGGGTTCAGGTTGCTCGTCGACGAGGGGGAGGGGAAGATCACGGCGGGGAGCTTGAGGAGGAACGCGGGGGCGGTGGGGATGGGGGGGATGACGAAGGAGGAGGCGGAGGCGATGGTGAGAGAAGGGGACCTCGACGGTGACGGTGGGTTGAGCGAGATGGAGTTTTGTATATTGATGGTGAGGCTGAGTCCTGGGATGATGGAGGACGCCGAAGCCTGGCTCGACAAGGCCATTGCGAACGAGCTCGCGTGATCGAGAGAAATTAATTCATCAGCTAAGTGAACACCACCACTGAGCTCGCAGTGGACGTTCCAATTGTAATCTGTGTTTTCATAGCAGTGAATAATATAAATAATGACAAATTTTAG

>UN00656

ATGTGACATCTCATCTCATCTCCCATTCTTTGATTTCCTTCTTTTTTTAGCCAACCAAACAATCTCAAAACAATTTTTCTTAGAAATAGGTTTCCAATTTTTTGATTTCCCTCTTTTTTTAGGCAACAAACAGACCCTAACAAGAACACTACAGATATCTTACTTCATTGCCCCATGTTATTTTCTATGGTTTATATACGCAATGCAAGCTAGATAAAACTGTGCTAATTGTGCTCCTTGTTTTACTTTTTTATGCGAGAGAAAATGCATTTAATAATTTCGTTATAATGCTTATTTATGGTTTACAACTGTCCTTAATGGGTAGTATTCTAATGCTCTTTTTTCGCTCCGCTGCAGCTTATCACGCTGGTGAAGTGCGCTTTGGCT

>UN00657

TTGTTGGTAAAACATGCATGCCATTTTCAAACGGAGCAGTATAACCAGAGTGATTATAAAGAGTTAGTTTCAAGGGCATATTATCGTCCTGCGGCACAATTTGACCACTACCCCTGTTAAATGCATCAACTAGTATGCCCTTTGACTTAATAACCTCAGTCATAACAGTGCTCATATCAGATTCACCCAATATATGGTCAATAACAGTGCGAGCTCCAAACTGTTCAGCCTCTTTTTTGCTCCTTCCAGGACCACCTACATACATTTTCCCGTCAAAGACAACAGAAGAAATAAATGTCGGAAACAGCCCCGCTAACTGGGAAGTTG

>UN00658

TTTGCTTGGTGTATGTGTTATCGGTTATGTAACAGAAATCTTCCACATGTGGAGGAGTTATCTCTTCATACTTCGATGCAATTAGCATAGCAGAAACACCAAGAAGCTGCAACTTCTGCCTGTTGAGCACATTCGACGACAGATACCGGTCGATATACGAAATTGTGAGATAGAGGGTATCGGAAGCAAGCTTATACTCCTCGGCAACCTCAATCAGCCAATCCACCAAGA

>UN00659

TTCTAAAGTTATTGCAGGTGAAAGAGAGATGGCTCTTCTTCGTTGGAATGGTTGTGTTCCTGTTGATAAGCCGGGTGAAGATCCCTCCAAGAGTTTCTAGCCCTAGGGAGAGCGGGGTGACATCAAGAAGTAGAAGCTCCTTAACGTCGCCTCTCAAGATTCCACCTTGAATTGCAGCACCCATGGCCACAGCCTCATCTGGATTCACTCCCTTGCTCGGTGCTTTTCCAAAGATACTGGCAACTACTTCTTGAACCTTTGGAACCCTGGTCATTCCACCAACCAAGAGAACTTCATCAACTTCTTTGGCAGCGATCCCAGCGTCTTTAAGGCAATTCTTGCACGGGGCTCTAGTCCTCTCAATAAGATTATTGACAAGTGCTTCAAATTTGGAGCGAGTTAGGGTGACGTTGAAGTGCTTAGGACCACTTGAGTCAGCTGAGATGAAAAGGGAGGTTTATCTCGGTCTGCATTGTGGATGAGAGCTCGACCTGCTTTTCTTGCTGCCTCTCTAAGCCTCTGCACTGCAACTTGTCTTTAGATAGGTCGA

>UN00660

GCTTATTCCCCTTACATTGGCATTAAGTGTTCTTTTGGGTTGGCATATCTATCTGATCTTGCATAACAAGACAACTATAGAGTATCATGAAGGAGTGAGGGCCATGTGGTTGGCAGAAAAGTTGGAAATATTTATAGACATCCATATGACCTTGGTGTCTATGGATAACCTAGTTTCGGTTCTCGGTCCAAACATATTCTGTTGGCTTTGCCCCATAACAAGACATAACGGTTCTGGTGTCCGTTTCAGGACTTCTTATGACCTAACATTTCCCGCGACGCCAAGGTAATTGATCCAGCAGTGGGAAAATTTACTCTGCAGTTCATCTAACTAGTCACCCATCTGCATCTGCATATACAAAAGGGAAAAAAAAGAGAAGAAAAAAAAAAGTTTACCATACAAGCACAAAGTGGGGAGTTACTCTGTTTCCATCTACCCACCGTGTAAATTTCCGAGACGT

>UN00661

AAAGTAGTGAATGAATGATACTACACTACCACCAAAATGACTTGCTCTTGGGGGGGGTTAAGGTACAACCTCTAGAGGCTTCATCTGTATTACCACTAAAAAGAAAAATACAGTAACAGGATCAAGAATTGAACTACAACTAATATACACCGGCCAGAACACTGAAGTTCTTTTACATTAAAAAAAAGAAAAATCATTGTCTCATGCAGATAAATACTACAAGCATCGCCTTTGACTAACAATTTAATCTGATAAACAGATCTTATGTTGCAAATGGCATTCTCTCTCAAGGTGGAAGCAGCACTTTAGATTGCCAAGAAAGAACACGAGAATGCTGAGGCGCCTCTCTTAATCTTCTCCTGTAATGCTCCAAAGTTTGCGTGTTTTCGTCCATGGTATCGCTAGATATTGTATCTTCTGTCAATTTCACGGCCGCCTCTTGTGACATTGAATGCATCCGCTACA

>UN00662

TTTCGGCATGTAGATATAAAGATGGAACCTCCAAGCTCTTTTTTCCCAATCAGTTCAGAAAACATCCTCAGGTACTTTGCTGAAGCAACAGGTGAAATTGAAAGATCGCAGGGAGAAAATATGTTTGCAAAGATTCTGAATGAATCAACTGCATTTCATTTCTGGAATGGCTTGACGACGGCACTAGTTCCTGAACCCAACAGTCTCGTGGACAGGCTTTTAAACCACAATTGTCTCTACTGCCATG

>UN00663

GGTTACGTAACAAACTATGCTATTGCGTGTCAATGATCACCCCATCGCTGTTGTGATAAAAGTAGCACCTACATTTAAGAAGCGCAAGACCGAAAAAGTTTACAGGTCTCCCGACAATAGTAGTGGAGGGGGTTGCTCGCTATTGTTGTTGTTGGATATGGTGTAAAAGATGAGATTAATTATATTGGAAAATAATGAACAGTTGGGGCACGCAATGTGGAGACGATGGCTACGGTCTAACATGCCGAGATTCCAGTTTACCTCCCAACAGAAAATAACTTCTAGCTAGAGTTTCCTATCCGATTCATAGTTGCTGTTGGTGTATAACGGCATGCACTTGCTGTGAAGCAAAAGAAAAAACCACTCATATGAATCGACAATTGATCCAATTTGCACCCCAAGACATTATCAACTGCTACGCACATCATTATTCGTCTCAGTGTAAAATCGACACTGCTGGATGCTACA

>UN00664

AAAGTAACAAAATAGACAACTACAAAGGGAACTTTGTATTTCAGAGTAGAAAGTTTATGTATTACAAATGGTTATTACACAAAGTGAATTTAGTGTTGTCTATTAAAAATGAAGGTAATACAACAAGTACAATGTTTCGCAAAGAATCACCACCCACCAAAACATCAAGCACTACAAAGATGATCGATGTCCATAGTCGAAAATTAGGGGAAGGAACATATGATGCAAGGAGTTATGTTTCATTCAAATCCCACAGAAGTTTTCGATTAGATTGAGCTGGTGGCGCAAGTCCAGGTATATCACGGATCGCCTTGTTATGAGGGTTAACAATCTTCACAATGATAATTGCTATTACACCAATTACAATCAGGACGAGCATGGCCATTATACAACGATCAGTTGCAACCTGTCTGCCAATTTCTTTAACCAGTTTAGTTGCTTTCTTGATCGAGAAATGAATTGAGTCAAG

>UN00665

CTCTCACTTTTACTTTCAACCCCCCTCACGCGCTCTTCCGCCACCTCAAATCTCTCGGTCGAGCTCTCTTCTCCCAAACCCCTAAACGATTTTGAAGTCGACAATCTCCTTAACGGCGTCCAAGATGAATTTTTTCCACCCGTCGCAGTCGACGCCACATCTGCAACCCCCAGGAAAACAGCTGCCAGTGCCGATACAGTCACACTAATGACCTCTCTAATTACTCCGGCCAGCTCAGCCTCCTCAACTCCGTTGGTGAATCTCAAACCAACTTGAGATTTAGAGATCTCTTTAAGCGACGAGGCGATCTTGACGAGCTCTTTCTCGCTCTTCCTGATCGATCTCGTGCACAAAGAAATATTGAGTTTATCTTGAATTCTGATGGCGACTTGAGCGTCAGATTGCTGCTGGGCAATCGTAATGAGCTCGGATCTGAAGCATCCGTGCGCGTCAGCGAGGCGCAGGAAGTCATTGAGGATGCGGTCGGTCCATTCGGACCCGGATCCGCGGCGGAGGGAGTCTTGGCTCTGGGGGAGGTTGAGGAGGTCGTTGAGGGAGGTGTGGAGGAGGTCGAGGCGGGAGAGGCCGTCGCAGAGGTAGGATGCGGACACGGGTGCGGATGAGACCCAGTTGGTGACGGATCGGATGTCGTCCTCGAGTTGGGAGATGAGGGGGTGGGAGCGACAGGGGAGACTGATGGATCGGACGTGGTAGGACTTTTTCGGGTCGGGTTTCGGACGTGGAGACCCGGGAAGGGAGAAGGAGCGATTGAACACGGCCACCATTTTGATTTTGATTTCCGCCCCCCTCGATCTTTCTGTTTTGTTTGTTCTTGGTTTGCGTTTGCGCC

>UN00666

GTTAAGTTACTTACTAGTATTAGTCTATTCCTTCCTCCCTCTCTCGCAGCGGCTAGGGTTTTGTTCTTTTCTCGCAGTAGCAATGGCTGAGGTTGAGACCGATGTTGCTACAGGCCAGCCGAAGAGGAGAACGTTCAGGAAGTTCGCCTTCAGAGGCGTTGATCTCGACCAGCTCCTCGATATGTCGACTGATGAGCTTGTCAAGCTCTTCACTGCTCGCGCTCGTAGAAGGTTTCAGAGAGGACTGAAGAGGAAGGCCTATGGCTTTGATCAAGAAGCTGAGGAAGGCGAAAAAGGAAGCACCCAAGGTGAAAACCGGAGCCTGTGAGGACTCACCTCCGCAACATGATTATTGTCCCTGAGATGATCGGTAGCATCATCGGGGTCTACAATGGCAAGACCTTCAACCAGGTCGAGATCAAGCCTGAAATGATTGGTCATTATCTTGCTGAGTTCTCAATCTCATACAAGCCTGTCAAGCACGGTAGGCCCGGTATTGGTAGTCTTACCCTTACTTTCTTTTCCTTAGGTTACAATAACCCTAACTAAAC

>UN00667

AAATTACAAAACCTAACAGCATCTTATTAGTTGCGTCCAGAATAATAAGAAATAAACCAATTGCCATCTGATATAATGAGAAACTTGATACAAAAGCATGGCGACCTTCCCACATTCAAACATATATTTAATAATGATAATAATAATACCAAAAAGGAGGGGAACCAGGAAGGATAACCATGCTCAAAAGTTCAATCACATCATATTCATGTTTAACATCCGAATGAGGATCGAACTCGATATGATCTCCAACAACTTGGCCGGCACAAGCTGGATTGAACCCGTAACCATCATAAAACTTGTCATCAGACGCCATCGGGGAAACCCAAGAGTTCGGAGGATCAAGACCCGAGGGCTCCTGGTTCATGCCCGGGAAAGATGCAGCACTTGGATTCTGAGGGGAGTTTCCAGAAACCCTCCGGCTGTTGGAGCTCCCCGGGGTGATCAAGGACATAGG

>UN00668

AGTCTAATTATCCACCAACAGCAAGTCTCACTGGTAAACAAGGCAAACATCAGTGACCAGAAATATAGTACAAGAGTACTTTTATTTTAGGAACATCATCTCACATCACATTACACGGAAATTATAAGCAAGTATCACAAAACCATGAACATTTTTTCCCCCCAGCAATACAGTCTACTTAGTACTTCAATGAGCTGTAATGGCAGGATTGCCAGCTTCTTCCTTCTTCACAAAAGCATCAATGTACTCCTTCCTTGCAGGAAGATGATCATGGGGACGGGTTATAGGGAGTCCACACTGGCTCGCCCACAAAAAGGCGAAGTGCCTTGATGTAGTTATCTGTGTCCAGAGTAAACCTATGATAAATACCAGCAGGTAGCACAATCATACCCCCTTTCTTCACAGCCACTCGGATCCAACAGTCATCTTGGTCCCTCACATCAAAGTAACCACTTCCTTCAAGGCAATAACGGATCTCTTCATCAATGTGCAGGTGCTCCTCAAAAAAGCTCTTTATTTTGGCTTCATAGTTTGGCATCTTTTCTGGGCAGATTTCACAAATATCCACGTAAGTGTAGCCCCTTGCTTCACGAATTTTCTTCAGGTTTTCATCAATCTTCATGGTTATCACCACCCTTCAAGCGCCAGCTAATAACACCAAGTTCTGAAAGTTTGTTCAATGGGATAAATTCCTTGGGCTCACGGTG

>UN00669

GTACTGGGGTGAACCGGGTTCTTGGCTGCGTAAATGAACATTTGAATGCCAAGGATGTTTATATATCGCATCTCTCGTTGGCACATATATTTGATCGTGTGATTGAGGAATGTTTTATTTTCCATGGTGCTTCTATTGGATTTTGGAGTGGGGTAAGCAATTCAGAACCTTAGACTGCGGGATCTTTATGATTGAATTCATTAATAGTATTGTCGAAAAGCGATCGATAGCAACCTCACAAGCAGAGTGTGCAAAGTACAGAGCAAAGTTTTGTGCAGCTCTCTTTTATGCACGTGACTCACCATATTTGTGACAGATTGTAATTCACGTGTTTTTGGTATTGGTGGAGGTGGGTTCAGAGGTTGTGATGGTGTTGCATTTGTTGCGAGTATTGTGGTAATTGGTAGTGTTGTGATTGTGGTTGTGGTAGTGTTGTGACATTTATTGTTTATGTAAGGTGGTTCATCCCAATTGCATAGTAGACCTAGTAAGGTAGAAT

>UN00670

ACATTTAACGACCAAAACTCGATCTAAAGCACGGCCGGACATATATTAGAAAACAAAATATGCCCACATAGGTGATATCTTGGCATGTATTTTAAATAAAATCTAGTAAAAAAATTCTTGAAAAATTAGAAAATACTGATTTTTGGCTTCCGAATCCGTATAGGTGTTTGGATGTGGTTCTGAGGGGCACTTACAAGTCACGTAGATGAAATCTCCATGAAGCGTACGAAATCGTACGAAGTGTGTATGATTTCGTACATTTGCATTTT

>UN00671

TCTTAGGTCCAGACCCTTTGGTTCAAATCATGATAACATTGTTTTCACATCATATTTCCATGTATTTGTCCTCTGTACTCTTCAGAGTACTAACAATGTCAAAATAAAATCAAAATGATCAGATAAAACAGAATCACACTGACATTATTAAGTCACTTCAAAATCGAACGGTATGAATCACCTAATAATTTTACAAGAGCCTGTGATATCTACATAAATGAACCCTTCGAAGTCATCAACATGATGCTTTCCTGGTTGCTTATCAATACATCTATTCTTGATTATCACCACTGGACACGACACGAACGCCAGAATCAATGTGAATTCACAGAATAAGCTTACACAAAGTCATCAAATCATTACCATGGTATTCAAGAACTACGGATTTTAGGATTGAACAGAATAGAGCAATGCGCCCTTTTTGTTTACTTCTTAGAAGAACCCGCACAGATCAAACCTTTATGGGCTCAATATTTTTCACCCATAGCAGCACCCTTCTGCTTCTGGAAATGAATGTATCT

>UN00672

CTTTTTGAAGTTTCTTGGATCATCTGGATCTTCAAAGGGGTAAGCTCCAACAAGCATTACATATAATGTTACCCCACAAGACCAAACATCTGCAATCTTTCCATCATATTCCTTTCTACATAAAACCTCAGGAGCAATGTAAGCCGGTGTTCCCACAGTTGACTTTGGCTGTGAATGCAACACAGAGGACTTCGAATAGCCAAAATCACAAATTTTAAGGCGAGGAGCTGTGTTTCCATCCAAGAGGGTATTTTCTAGCTTCAAATCTCTATGACATATTTGCATGGAGTGACAATAACTGACCCCCGAAATCAATTGTTGGAAGAAGAACCTCGCCTCA

>UN00673

CAGAATTTTAAAAAATCATGACATCACCAGAAACATCATCACGAATCGCAAACATTTCTCCTTGTGACTGTTTTCTTCAAGTCCACAATTTATTGTATTTCAACACGACATCAATCGGTTACTGATTTATCAGAACATTCTATTCTGTAAAAGTTTTGGTTTGTCCGGAACAAATAGAACTTCTTAACGCTTATCACAACATCAAACTCGACTTCATCGATTAATACAACCTTTCATTGCCTCCAGCAAAATGAAGTGAGAAGACTCCTGCTATCTGTATTCTTGGAAATTCACATGGCCAGTAGCTTGTGCATGTTCCACTGCTTCCTTTGGCCAATTAAACCAGCTTGGCATACCCCACAACGCAAGGTGAAATTTGCAGTATCGGTGTAACTCCTCTTTCTGTTAGCATCTTTAACAAGATTTAGAGCAAGATTTTCTACAGCTCCAATTGTTCGATCTTTATTGAACAAGGAAAAAAATTAAGTTTAAGT

>UN00674

GTTGACCGAAGCAAACACTGGGGAGATTTGGAGGAAGAAGAGGAGGAGGAGGAGGAAGAAGAGGAAGAACAAATGGAAGAAGAGGAAATGGAGGAAGGCATTCAATCAGTTGATAGCCTTTCAAGCACCCCCACTGGTGTTGAAACTCCTGATGTTATTGACCTACGAAAGCAACAGAGAAAGGAGCCAGAGAGACCTCTTTATCAGGTTCTGGAAGAGAAGGAAGAGAGAGTTGCTCCTGGGACTCTGCTTGGAACAACTCACACTTATCTGCTCACTGATAAAGACAAAGACAAAGCAGCTGCTGCTAAAAGGGTTGATCTTCTTCGGGGTCCAAAATCAGACAAGGTGGAAGTCACCATCCAACCAGAGGAGTTGGAGGCTATGGATGATGTTTTGGCTGCCAAGTACGAAGAAGCACGTGAGGAGGAGAAGCTGAGGAACCAGAAGGAAGACTTCAGCGACATGGTTGCAGAGGCCGAGAAGAAGCGAAAACGTAAGATGCAGGATAAGGAAGGCAAATCGAAGAAGAAAGATTTCAAGTTTTAGTTTATATATCGAGCCGAGATGGCGGCATGGAAGTTTCCAACTGTTGATAGTAACCTTTAGTAACTTACGAAAAGTTACAAGTAACGTTTAAACTAGTAAAATCAACTTAGCTCTCTCAGTAAACCTAGTTTTGCGTGTCGAAGATTGAGAAGTTGCTACGATTGTGGTGCTTG

>UN00675

GGTCTGCTAGGAAGTATATGCATTATCCTTCACGCAGAAGCTATGCACCCTCCATTACTTCACAACTGGTTCTGTAACTAACCAAAGATACAACACGCTACTATAAGCTTAATAATAACGTCCTGTTACACCGAACCTCTTCTAATAAATATGGAGAATTAAACAAAGAGGGGGATGCAAAAGCGATAGTAAACTAGGCATACAATTTGTAGAATCTCAACGCCAGCTTCTGAGCATCAGCAAAATGTGATTACCTCAAACTAATCAGAATCAGATTCATTAGAAATTTCGGATTGTCGAACGCTTCGTTTGCTTGTGTCTGAGTTGATTTTGCTTATGGAGGATTCACCATCGTATGAAATAACTTGACACTTTGCGAGCCTCTCCTCCAGATCTTCAGTGCTGAATTCATCAGTACCTCCAAGTTCATCAAATCCCACCACATAATCGTCTACTTTGGCATTCTTTACGAGAGCAAGGGTCGGTAGTACAACTATCCTCAGCTTCTCAGTAAGGAATGGACTTTTCTCAGCATGAACTTTTATAAAGCGTGTCTCTATATGTTGCTTTGCTAGAATACTCAAATGCTTGTCAATTACCTTGACAAGGCCAATTCTCGCGAAAGAAGTGGCAGACAACGCGATCGCTAGCCTTAACAGCAGCAAAGAAATCCTTCTCAGAGATCTCAGTGTACTCGCCATGGCCAAGAGCAAGCCACTTGCTTCTCTTCTCCGCCATCTTCTTCATCTGCTGAATCCTCCTCTCTCTCAACACTTCGATGTCGTCGAGATCGAGGCGATCTAGGGCGGAGATCTCCTCGTCGAGCTTGTCCTCCACTGCCTTGGCTACAGTTAGTACTTGATTCTCGATAATCTGCTTCACTAGAGATTCGCCCATGGCTGAAGCTCGATCTGATCTCAGACTCCAAGGGAGAGAGTTTGATGGAGGGCTTTTGTATTCACCC

>UN00676

GTTCTTATTTTGATTTTCTATATTGCAGATGAAGGCCCACAGCATGGATGGTGTCAAAGGATTAAGACTGAATTCACTTTTGAATACAATTATCTTGAGGATGAGCTTGTGGTGTAGATATGAAGCGCATGACACTTGCGAATCGACGAAGCTCCAGCCTGTGACTGAGGAGGAGACGAGGCAAATCGGCGTGCAAATGGTTATTCGGGTTATGGAAACTGTAAAACAGAGTAGTGTTGTGATTGAAAGTTATTCATTTCGAGTTTTGCAACACTTCGCCATTTGTAAGTTAAAAATTGTTGTATAATATTGTGGGATTGATATGGGCCAGCTCCAATATTGTGGGATAAAGGCCTTGTATAATTGTTTATGTTCCCTGTGTGGGTACGGGCATGTGAGATGACCAAAGCAAGCGATAATGGAAGAATGGCAATATTTGAATTGTCCTTAGGAATTACAAGCTCTTCCCTATGTGTTGAGTTCATGTACTACTCTACAGCAGAGAGTAACATTTGTACCGGCCATGTTTTAGTTGCTACGGCTTAAATAGAGTAAGAAAAAGAAAGAGTAAATTTATTCATTGAAATTTAAATGTAC

>UN00677

CTGCAAAAGAAGGGAACTGAAATAAAAACCGGGAACCTCATTTCACACTCATAAATCCGCATTTCTGCACAATTTGATGCTTGTAACAAGCTGTCGGAGGAAATCAAAGAGCTGGATATTGCATCTGATATCATTACTTTCATAACCCTTCTATAATTGAATATCATGTACAGATTTTCTAACTATTGGCTTATCATTCAGGCACCATTAGACAAATAGAAAAAGTTTAATGAACATCAACGCGGACCAAAGAGGTTAAGGCTCTACACTTCTCTATCATCAGGTACCATTAGAGTGAGCTCCGGAAGATCGACCGCAAGCCTCTGCCAATTCCTTTAGAAACATGACAACATCATCGGATGAGTTAAGGAGATACCTTGCATTTGAGCGCTTCCTACCAACAGCACAAGAGAAGTAGTTGTCGCCTTTGAGATCAAGAACTGAGGATCCCTCATGCCAGGATATGATCTCTTGTGGGGTCCGCCACCCGCTTGAGTGGTTATGGTGATTGTTTACCAATGACGATGATGTTCTCTTGTCCGAGGATCCAAATGGAGTAGATTTGTGAGTTTTGTTGTGGAATGATTTTGAACTGTTCTTGTTGTTGCTTGTAGGTTTTCCCATGTACTTCTTGTCTGCAGATGCTTTGACCGCATCAGCTATCTTTGATCTCATGGAAGTTACAGGTTCTGATGGGAGCTCTGGCTCAAAGAATGTGTATATGTCCTCATCCTTTCCAAGGAAGTGCCCGACACATAAAACATAGTCTATTGGAGTAACCATGCTTTTACTGTGAACTATTTCACCCAATATTCGATCAATTGTCTAGTCTACCCTTTTGTTCTACTTCCAACAGATCGGACCTCAACTGATCGACTTCCCTGGACAACATCAACTGCTGCATTAGAAATTGGGCCTGTCCATAGATAGACTAGACAAGAA

>UN00678

TTATTTAAAAAGTTCTTACTTAGTCCTTGTATTCTTGCGCTAGCAAAACTTCAATAACTCGAGGTGCTCTGTTGGATTCTTGCGTGGGAAGAAGCATTATGATAATAATCAGCTACTACTGCAATGTATTTGTATCACTATTTTACATTATTATTTTTGTTACTGTACGGATTGATTAGAGGACAATAACGTAAACCTGACTGTAGTGTTTCTGCTGGCAAGACAAGCAGGATACTTATTTGTATCCTGAGATCTAGAAAATGTAGGTGAAGGATAAGTTTTGGCTTTGATGTATTATTTCATATTCGAGAAAATTCATCTTTTTCCTTTTCTGGAACATCCTTATGTTTTTGGATATGCAATCTTTATACCTTAATAGACTAACCGTTTAAG

>UN00679

AAGTCGTATAATTTACAGTCTTGTATTGAACTAATAATAAATACAACTTACATAGCAAAAAGCAGTTCATAACTTCAAAAAAAAAAAAGCACAATACCACATACAGAAAAAATGGGTCTCCATCCAAAATATGGGTAGGCCTTATTGGGGACTAATTGCAGACTTTTATAGGATTATTTCCTCCTGTACACTGACATTTAGGGTTTTATTAGGGAGGACGATGCTGTTGATCACCACCACTTCATCTTCTACACCAACAGCTTCTCCTAAATAGTAATCCCAAGCTTGGCGTTATAGTCCCCTTCACCCTGAACACGTGACCTATTTTTCCGATTGAAGACTTCCACCCTACTATAGAATGAATAGAAACAAAAACT

>UN00680

TCATACACCCACCGTTCATTCAAATAAAAGAAAAGATAAACATATAAAATTAAAATTCCAACACCGACCGAGTACAAAAAAGAATAAAAATAAAAAATTGATGGCTGTGTCAGTTATAGCAGACATCCACGGGCTACAGTGGATAAACTCAGATTTCGCGATCCTGACCTACCTGACATTTTTCTGTTCATTGTGATGCATAGCCATCACGTCGAATTCAGCTTCTGTAACTGGGCTTTAGCCGCATCGAGTTGTTCTTGCTCATTTGTTTTTGCGGTCTCCACGTCTTTGCGGAAAAAGATGTTGCCAGCTTTCTGATACACACCTTTGGATGGCGGGAGAGCGTTCAATTCCACGATCGCGATCTCGTGGCAACGTATCTCTTTTTGGAGGCTGAGGCTAGGGTTTACAGAGGGCAGGGGGGGTGGACGAATCGGAGGTGAAGAAGAGGGAGAAGCCATCGGGGTTGGAATCTGTACTCGAGACTAATGACCGTAAATTTTTTACGGGAC

>UN00681

TTAGTAGAATAATTTAGCTCCATTCGGAGTATTGGGTGATTCAGTAGGGTTCACTTCAAGAGGTACATATGTTTTCTTTTTGCGCTTTCCTTCAGGTATGGACGCTTTTAAGTATAGCTATATGTTTGGCTTACTTTTGGTGGGAAAGCAAGAAGAATATCTGAGACATCGGATGCACCCATATTTTCCACCTTAAGAGTCAGGAAAACCCTGATAATGGAAGAGGTCAAATCAATCCTTCTCTCGGCGCTGAGGATCCGAACCTCTTGCGATGAAGAAGAGGATCCGGGAGAAGATAGAGAGAGAACGACGATGAGAGAGAGAATTAGGGTTTTTCGAGAGATTTGGAACCCAAATCCTTCCCCCATTTTTTCTTGATTCTTCTCCCTCTACGAC

>UN00682

TGTTTGCATAAGCAAAATAAATATCAAGACTATGGAGGAAAACAAAAGCAACAATGACCCAAATTTGTTGCCGTTATGTTAACAGAAAATGTGCTGGAGGCAGCTCCGGTAACAAAAGTAAGAAGGGGAAAAAATAGTGATAAAGGTTTGCTGGTGGAGGTCGTCATATTTTTGTAATTTCATCCTTTTTTGCTTAGAAAATAAGAAAATTGATGTAAAATAGTAGCAGGGAACAATTAAATAAGCAAGAAAAGACTACAAAGAGATGACGACGGTTCAATGTAGAGTACAACAGGATTTTGAAGATGTAAACAGCAACATCAAATACTGGTTTACTCATCCCCATTCATTCCTTTGCAGCAAAAAGTGCACAAATGACTGCCGAACTGCGATATGACATCTACCAAGCCCCCATAGCCACCAACAGCAGTAACCCGTGAGGG

>UN00683

TGCTGCTATCAAGTCTGCTCTGATGACTACAGCTCATCGGATGAATGCAACTTCGAACGAGGAAATGGAGTTTGCATACGGTGCAGGACAAGTAAACCCCGTTGCAGCTAGCGAACCCGGGGTTAGTTTACGATGCAGACGAAGCAGATTATGTGAGAATGCTTAACGAGTCATTCACTGAAACTTTTGTGGCAAAGATGAAATCAGTCGCTGGTAAAATTGTTGCCATGAACTGTATAGCAGCATACAGAAGTGGTTTGGACATTGATACAAATGTCAGTAAGAAGGCTGCCGAAGAAGGTTTGCTTGAAGATTTACATGGTAGTGCAAAGAAAGACTGTACGAGACACTTGATTGGTGAATACGTCGTATGGTTATGTATTTATTGCTTTTTGAAGTACATGATGCTTATGTTGCAAATTTTATTTTCTACAGCTGGGAAGCGTATGTAATGCACCAAAGTTAGTTTTTGTTGTTCTGTGTACTATGGTTAATTTTTTGTACTCCATGTTGGATTATCCAAAGAACTAAGTAATTTAGTTTTTCAGAC

>UN00684

ATATTCTCAGCTCCTCCAAGTGACTGGCGACGAGAAGAACCATTACTCATACTCTTCGCATCTATAGTGGAGCGATTTGAACCACTTGAAGACATCCTCAGGCCATCTTCAAGGACTTTAAGTCGTAACTGAAATTTATCCTTTAACTGGGCCTCCGATCTTGCAGTACGTTCCGCAACAGCAAGTTTATCTCGTAGTTGTTGCATTTCTCCCTGCATGAATCTCCGCTCTTCAAGCCACTGTTTAACAGGCATTACTTTGTCATTAGCATCTTTCCATTCATTTGCCACTACAACTGCGACTCTGTTAGCTGAAACCTTTGCACGGGCTAGTTCTCGGTCAAGAGTTTTTCTCTCTTCATTCATCTCTTG

>UN00685

GTAAGTATAACACGTTTTTCCGTTCATGCAATGTTTACTTCCCGCATAACTAAAGTGAAAATTACATCGAACGCAATTTATTCAGAGGGGGGTTACAGTGACATCTCCTCCCCCAGAAATAAAACCCCTAAGCAAGGCCAAGGGATACACTCAATAAAATGAGACAAAGTTTATCAACAACAAATGTCGATATCACCTTCAGTTCGGTTGATGATCAAGCGGAAGACACTGCACTCTGTACAGCAACTGCAGAGTTGGCAAGCAAAAGCTCAGAAGAGTATTTCAAAGAGAAGCCTCAAGCTGTTGTGGGAATAACTTTTCCAGCATGCCATTTGAGAGAATCCTGTTCGGGTCGAGCTCTCTTCGAGCTTTATTGTAAGCATCGACAGGAAACCGCTTCCTTAGCCTTGCTTGAAGTTCTGCAAGTTCTTCTTTGTTCTTCGGCACCTCAATTTTAGCCCAATGCTCATAGGCAGAATATTCTATTCCCTAGTAGTCTATTGTCTTTTGGGTTCAGACGTCGATAATCAAAAAATACTTCCGTAGATAC

>UN00686

TTGGGTATTGGGTTAGGGGCTCTTCTGTCAGGTGCTCGTCTTATTTCAGATGGGATGCTTCAAGCAGCAGCAGAATGCCTTGCCTCTTATATTACAGATGAAGATATCAGAAAGGGGATTCTCTTTCCTTCTATCTCCAGTATCAGGCATATCACTACCAAAGTTGGAGCATCTGTTGTGTGCACTGCTGTTGCTGAAGAGCTAGCTGAAGGACATGATGATATGGATGTCAAACAGCTGAAGCAGATGTCAAAGGAAGAGACAGAAGATTATGTAGCTCGTAATATGTGGTACCCTGTATATAGCCCTCTTGTTCATGAGAAATAAGTCAACATCCCAGCTTTCCCTCATGCGGCTCTTATATCAGTCTGGTTTTTTTTCCCTTTGCTTTTACTGGATATCTTACAGTCACCCGGTGGAATACTAGTTTTCTCCTGAGAGGCTGGACGTGATATTTACAGAGCTTGTTATCGAACTTTTAAAGCTTTGGATGCAAATAAGCAAATTTTGACTGTTCATGTATGATGTTAATCAAAAACAAAAGGTTGAAGGAGACAAATCACCATTAAGTGGCTACTTAAAATGTGAAACACTTTGTAGTATTGCTTGCAAGAATATATTATATTTCATTTTGCACGTTATCCTTGAGTTCCAAGCTACAAATATTGTTCTTGTAAGGTCTCAACCAAGGCTTGGAAGATTATTGTCCTCAGGAAGCCGTTTTAGGTAAAGTGTGGGAAGTGTAAACAGACGGTAACTGTCATCTTCTTTATCAGCAATGCATTTGTTTTCAGTTTTAGGT

>UN00687

ACGAAAAGTAGAAAATTGCTGAACGACCAAGATTTCTCAGTGGACGACCTTGAGATTTAACCCGTGAATACATGGAGTAGCACATCTCAGTTGGGAGAACCTTGATTCTTCTAAAGATCACACCAACCTGCACAGAGCAGCACCATGTTCTTCATGGTGAGCTTACAGTTGTATGCAGATAAAAAGTTTCAATGTTTTGAATTTCTTCTGGGATGGTGCTTCTTTTTGAAAGATGTAATTAGTTACTACATTATTCAGATTGTATATAGTGTCTCATTGCAATTTATGGTACGTGACCGGCTTGCATTCAGCAGTTAAGTTGTAAAAGGTCAAATTTGATTGCGCTGAAATTTTGAACCATGTATGTTTGATGGGAGCATGTGGAACTGGAGCATGTCGATCTCTTCTGGCTTGTCTTTTTAGAATGTTATTTTGCCTATTTTCTTGT

>UN00688

GGTTCTAAATTTTGTATGAGGAGACTCTTGTAAATGTGAACAACATTAAGAAGCAAAGGACATCTAGCCAAAGATCCAAAAGTTTTAGCAATGAATACATTGTGATTACAATTCTAGTGGCTGCGGAGGGAGTGCACAAACTACCTGTCATTAATAGCAGTGCAGACTTAAAGGAAGCACTACAAAAGCTGGGTTCTATGCCAAGCAACAAGACTCTGGCTGTTGAAGTGCTGTGGACTCCTCAGAATGAAAACGACACTCTTTCAGAGAGGGAGCTTCTTGAAGACTATCCACTTTTAAGGCCATTGTAAGATTAAAACCCGAAAGTTTTTCTGTATACCGTTGCTTCTGTAAAAAATGAAATGTGTATATAAAGTAGATTTACAGAAGTTTTACATTATTGATTTTGTTACAAAGAGGGCTTGTAATTATAACAGAAGGCTTCTGTTTTCTTTCTTTGTACTT

>UN00689

CTTCTGCATTTGCATCACTAATTTCATTAGTCATAAGTATACCACTTGTAGGCTGCCATAGCTGGGGTGCCACCTGAGCAGTTGCCTTGCCAGTGGCATTTCTATCAGTCTTCTGCCATTTCCATAGCTTGTGAACAGCATTACATGTCAAAGCCAAGATTGCATTTCCTGAATTGGTATAAATCAACCTAGCAACCTTCACCGGTAACAAGTTGTCAGAAAGTTTCAATGAACAACACTGGGATGGTTCACTAACTTCAGCCAACTTCCATATCTTAGATTTCTCCATTGACTCGTCAATGATTCTAGGTTTAAACATCTCCAAGACTTCGATTGTCACCATTCTGCAATCCGACCAATAGATCCCATGGGCGCACTTCGATCTGGAAGTGCAGCACTAGATCCAGCCATAGCAGTGACAGTACCCAAGGGTAGTGATACCAGGTGTCTTGGTGATCGCCTCAGAATTTGATCTAGAAGCATACCGGCTTTCAACAGTACGAAGCATTTGAAACCCATCAGCATTTGCCAAAATCTTTATACAGTTATCAACCGTTGATACGGCTAAAAGTGAACCAAGTTTGTTGAATCGAATGCGGGGAGAGGCAGGTAATTCGCCCCCTGCATCTGTGGTTGTCAAGAGGTTAATATTGTCCATGTCCCAGAATTTGACCATAAACTCATCTCCAGCGGCTAAAAATCGATTTTTTGTAGTGTCAAATTGTACCACACCCAGGGATCTTTTCCGAAAACCAACATATGTTCGTTTTACAGAACCTTCACTCTCATTCCATTCCACAATGTATGAATCCCCATCCTTACTGGTACCGCATGAGAAAAGCCTTGCTCCGTCAGCACTATAAGCCATAGTAGTACACCAAGGACCTGGTGCTGTATAATCAACCCTTGAACCCCCATTGTCATACAGCCATCC

>UN00690

AAAACGTTTAGTAAAAATTAATTGGTTAATTACTTCATGCGCAGTAGAAAGAAACGAAAAAACATACAATAAAGATGTTGCTAGCTAATAGAACCCTTCATCACCAGGAACATTACATGATCGGTCGTGAGAATAACTTGATTTAGCCCGAACACGATAGGTTACAGCATAACAAAGTGCCCGAGGCAACGAAGATAAGGTTGCTATGTCCGCTATTACAATTCTAGTTTAATTGCTATGCTCATAATTAACATCCACCTCAGTTTCAGAACAAAGAAGATGCTAGCTCTTTTACATCCGACACCACTTGATCATACTCAGATTGCAGCTTCTCCTGCACCTCCGGCCCTAGTTCTCCCTTTGTCGGTTTTGTTGTCACTAGAACACAACAAGTTGGTCTCTTGGTGACTCCCGCAGTTGCAAGCTCCTCCTTAGAAGAGACATATATGTAAGGAATATCAGCTTCTTCACATAAGATAGGGACATGTGTTATCACATCAATGGGCGAAATATTTCCTGCGATCACACACAACCCTTTGTGACCTCGACGAATGCTCTTAACAACTTCCTTCACACCTCTTTTAAGGCATTTTAGATTCAGCAGCTTTTCTGACGAGCTTGAGGATCTTTTTGGAGAGTTTCTTGCCAGCGAGGGGTTTGGTGATGGGAGAGAGACCCATCAGCTTCTTCCTCTCCTTCGTCGATTTCTCTGTCTCTCCATCGCTCCCCATTTTTCCTCTCTCTCTCTCTCTCTCTCTCTCTGCCACGCAATGCTCATGAGTTATTCAACCATCGACATGCATCACTTAGAAATTGTATTGAACGTATTTTTGGCATTTTTAAGTCTAGGTTTACTATTTTTAAGTCTCAACCTCCAGGTTTTCCATATGAGACTCAAAAAAGTATGATCTGGGCATGTATCGGTTTG

>UN00691

TTCTTATTGCTGATTCATGCAAGAAAGTTGTGGCAGTTGGAATCAATTGCACACCTCCCAGATTTATTCACAACCTAGTCCTCTCAATTAAAAGGGTGACAAAGAAGCCAATTTTAATTTATCCTAACAGTGGGGAAACATATGATGGTGCTAAGAAGGAATGGGTGGCATCTACTGGAGTCTCAGATGAAGATTTTATTTCATATGTGTGCAAATGGCTTGATGCAGGGGCCTCCCTTATTGGAGGATGTTGCAGGACAACTCCAAATACCATCAGAGCCATATCCAGGGTTCTAAATTAGGATTTTTGATAGCTAAATATCCCATAAAAGACTAGTTTTTTGCAATGTTGGAATGCTAGTTTATACATGTATCTGTGAGCACCGGTAGATGAACTATGTATTAGAAATTACTTACAAGAGAATAATTTTTAAGCGCAGTTTACAGTGTTGTAGAGAAGAGTGGTTTGACAAATAAAAAGAAAAGTTAAATTAAACC

>UN00692

GTAACGGAAGAAAACCGCTGGTTTCTGAGAGGCTCGGTCGACGCAAGAGCTTCAAGGCCAGTCCTGAAGCTGGGAGGGCGCTTGGCGTCGCCCGCTCGAAGCGGAGCGACACGCTGGGGATCACGTGGAGGTCGATAACGGACGGCCGCGCCGTCCCGCTCGCGCGCCACCTCAAGAAGTCCGACACGTGGGACGCCACGCGCGCCCCGCGTGGCAACCCCGTCCCGGGAGGGCCCGAGGCGGCCCCGAGCGAAGCCCAGGGGGCTGCGGCGTCGCCGACGGTGAAGAAAGACACGTCGTTGGGGAGGGAGGAGCTGAACCGGAGGGTGGAGGCCGTTCATCAAGAAGTTCAATTGAGGAGATGAGGCAAGAATCGTTCAAGAG

>UN00693

GTTTTACGTTTAAGTTTAATTTTAGTTCCTTAGGTCTACTTCTATGGCGTTTATAAGAGCTAATACATAATCGAGACCAGAATAGAAGAGCTAATACAGAATCGAGATCAGAATAGAAACGGCGATTATTTTTTTGCCCTTGTTTTACCATTATTAAGGTTCATATGCTTATAAGCGGTTTCTACACTCAAAATAATTTGCCTAGTTTAGTGGTTTTGTGAATCGCATTGTGTAACAGAGGAAAATTTTGTGTAATCTAATGTGTGTTATGTTGGTAAAGATGCTGACTGAGTAGCTGAGTGATGAGAAATTGCATTTATTGAACTTCTTGTTTAGTGACATGTTGGATGAATCTCCTTTTGTGGTTGTTTGTTTTTATATCCTAAACTATGAAATTTGTTTGGTTCT

>UN00694

GAAAATCAAATAACTCTCACCAACAAAAACCAAATACAACAAGAAATATTAGTAAGCAAGTTCAATATAAGTTCCAGAGAAAGTAAATATAAGGGAACCAACACACAATGTCTACGTGCCAGCTGAATAACATGTGAAATGTCAAGTTGTCTTGTCCCAGCTGTCTCTGCAATTGACATAACACTACAGCAAGGGTTAAGATGTGAGTCCCAAACATCCAACTAGTTGTAGTCATAGCAAGAGGAAATAAATCAGGCTCATAATAGCTCCATGGAAACCTTAAGCCCATCAATGCAGAAACATCACAGCATCATAATCAGACTCACTTCATCTCAGACATAGAAACTTAACTAAACAAAAATAAAAACTAAAACGGAAAAA

>UN00695

CTCGGTGTTGTACTTGGCCAAGTATTCGGAAAAGGTTGGTGGTCCCGACTGCGGTTGTGCAGTTCGGACCCTACCGGTGGGTGAGGCACCCAATTTATGCATCGACCATGCTGCTGTTTGCTCTGCATTGTATCACGTTGCGGGCTCCGTTGAGCTTGGGGTTCATTGTTGCGGTGTGTGTTTGGTATTACGGGAGGAAGGCGGAGTTGGAGGAGGAGCTGTTGCTGGAGAGTTTTGGGGAGAGGTATAAAGAGTACATGGCCAAGGTCAGGTATAGATTGATCCCTCTGTTGTATTGATTAGCATTGATATGCATGTAAGCTAGTGTTCATGTTTTTTATGTTGAGGAATTAAAGTTTTGCTGTGAGAGAGATGGCGTTTGTTAGCATGATTTTGGGTTTTGAGTTTGACTGCTTGGAGAATGTACATCATGACGGTTTGAAGTTTATGTTATTTATTGTACTCTTGAGTGCGATTGGATACTAATTTAGGTATTTAATATATAAACCCTTTACTTGACTT

>UN00696

CTAATGACGTCTACTTAAGGAGGGGAAAAACTTTACATAAAACTTTTTTCTTTACCACAAAACTGATATTAATGACAAAAAAAAGCACAAATTTAAAAGAAAAACACTTCCAGTTTCTATTCAGTATCTTGGAACTTCTCTAAGAGGAAGCATGGGCCTGGTTGATATTGGTGAACCGGAGGGAGAGGAGGCAATGTGGAGCACAGCAGAAGGATAATAGCCGGCTTCGTCAATGCCTTCTGATGTTGAAGGCTCATCTCTAATCATGGTTCGAAAAGATATTGAGCCACGCTTTGTAGAGGCATACGGAAGTTTACTTAAAC

>UN00697

ACTTACCTTATTACAAGGATCCGAGTAAGCTCTCGGCGTATAGAGACCGTAGGTTCCCTGGAAACCAAGAGGAGTATGAATATGCACTGCAGACATCGACCACTGTTTATATAGGGAACATGTCGTTCTATACGACAGAGGAGCAGGTCTACGAGCTCTTCTCGAGGGCTGGAGAGATCAAGAAAATCATCATGGGACTTGACAAGAACACCAAAACTCCATGTGGCTTCTGCTTTGTCTTGTACTATTCCAGGGAGGATACTGAGGATGCAGTCAAATATATTAGTGGTACAATTCTTGATGACCGGCCAATTCGTGTAGATTTTGACTGGGGGTTTGAAGATGGAAGGCAATGGGGTCGTGGTCGAAGTGGTGGACAAGTTAGTTCATGGATCTTTTCAGCTATCTACCACCTGTGCTTTCTGGTTCTTTAAGGAACTTTTACTCGCTGTAATGTGGTACCTTTTTCACTTTTCACAGGTGAGGGACGAGTATCGGACTGATTATGATCCTGGTAGAGGTGGCTATGGAAAGTTGGTCCAGAAGGAGCTGGAAGCACAAAGGGAGTTAGTTGATTATGGTACAGGTTCGTTAGGAGATTTCCAACCTAACATGCCGTCTCAGTATGGTCGACAAGGTGGTAACCGTGGATACAGGAATCCTTACAAATATGAACGCAACCCACGTGATGATCGAGGTCGTGGAGATCGTGACTATAATAGAAAGCGGTACAGGGATGATGATCGTGCACCTGAGATGTCAAAGAGGGCTTCTGACTATGAATCTCGAAAGAACTCTGATTATGATTCTAGACCAGAGAGAAACCCACGCTTTCGAGAGAGTGGAGATTCAGATGAAGAAGAAGATGATGATAGGAAACGTCGTCGCTAGATGTGTAATGTCATGTTTACTGTTTGTGTAATGTTGAGTTTGGAGTTTTGCCATATGTTTCACCAAAAATTGTATGTTGAACTGTTTTGAAGTTTGGAACTCATTTGGATAATCAGACGATTTTCGATCAGAATGACATGGGAAATGGAGTTGGTAAATT

>UN00698

GGCTACTGCTTATTATCGCACGGACCTCTATCAGCGATCCCTAAATGAGGTGCCCGTGACAAACTTTTTTGGCTCAGTTATGAAGACAATTCACACGGACTCAGCATACACTGGCTTCCAGGCTCCACCTGAAATTGAAGGAGAAACTTTTCTAAAAGACCGTGATGAGGGAAGAAGAGCATTGCAGAGTGATGCTGATGCAACGAGAACAAATGAGGACAAGAAGGAGCCTGGGTGCCCTTTCCATGCATGGATGGATATTATGCAAGAGAAATTGGAGAATGAGGATACTGATGCTTTAGTGAAGTATGGTCTGGGATCCATGATATTATTGCTAGGCATTTCCACTTGGCTATCTAGATAAGTACTTGATACAACTTGAATGTATAAAGTTGTGTTTATTTTGAATTTTGAACAAGTGATGTACTCTAGCTTCTTCCTTATTTCAATATTACACTTTTGAGATACACTTGTACCTTTCCCTGAACATTCATCTTTTTTATGACAGAATTTTTAGAACGGTTATGCTTTACATTAGGCTTGAAAACTCGAACTCGACTAACACGAGCTTCTACTGCATACTGAAGCTCACCTACAACCTTGATCGAATCGTCCAACGAAAGCAAACTCGGTGTGAGAACCGAGCGAGGTGTCCTATCGCTACTAAAGTCTCCACGTTAAACAAGGAGGGCATTCCCTCCACTTCTGTTGTAATTTTTGATCTATTAGTTTGTAATAAGCTATTCTTATCAATACAAACGAGGTCTCTATTTCGAGAAATTAGATTTTTACACCTATAGAATATGGCAACCATAGTGGGAGAAACTATTTCCCTCACCGAAATGGTAGTGGCGTAGTTCGACGAGGTTGCTGTTGGGACAACCGTGGAGTC

>UN00699

CTAAACAAACATTGTATTTCACTTGCGTGCTGACATGGATGTAAAAGTCATTTGCAAAAAAAGTGACTTACATATTTTAAATTCACTTTCAGATTGCCAAACACCCCCTAAGCGTAGCAAATTTAGAGAGAAAACCATCTCTTAGCAAAAGTCCACACATATTTATAGGGTGCAAGTGTAGACAATCATAAGTGAATGCATTAGCAAATAAGTCTTGGATTAGAACAATAATATGGAGTGGCTGTTACAAATAAAAAATTATGTGTAATCCCAGTCCTCGAACCCATCGGCAGACAGCACATCGTTCAGTAGTCCATTTAGCATCTACAGGCTCGTATGCTTCTTGTAAAAGAGCGAACAACTTTCATTTTCATACCTTCAGAGAAGGTCGTTTTCCATTATTGCCCGAAACTGTATTGTAATGATCTAGCAACAAGAATACAATTTCACAAAAGCCAGAATGTACCACCCGAACATGCCTTAAAGCTGGGACGCATTAGTCCATCATCTGTACTACTTCATACAAAGGGTGCAACACTTGGAAAAAATTCAGGCTTTCTTGCTGAGACAATGACCTGTGAAATGTCTCTTTTTGGTTCAAAAGAGCCAAAGGGAGCTCTGTGTCAGGCTTGACAAACCATTCTGGTTCAGCCATTATCTCACTCTTAAGTTGGCCTTCAACATGTGCTCTGAAAAGGGCTTTAGAAACAGCCTTGAAAAGCCCTTTATAGTCAAATGGTCCAAGTTCAGATGATATAGCTCCTTGATCCAATCTGTCAACCATCTGCATATACATTACAGCTTCTAACACAGAGAAGAGAGTATTCAGAAATTCCCATCGACTTTCCTTCCGAAGTTCAACCAGTTCTCCCCCATAAATCTTGCTTATCCAGTAAAGATGTAGAATTTTCAACCACTTTCAAAGATCAACCGAGACAACAAGGCCCCCAAATCACAGGATACTAGAGGGGATGGCCTTTAAATCGACGAACTTGTCGGACGAGAGGAAGTGAAGGAGAGGCGCGGCGGACGGGGGAGTCGACGTGAGGAGGAAACGAAGTCGAGGTGGTCAGCGGACGCGCGGGAGTGCAGGATAGCGAGGAAACCCTAGAACTCAGTGAGAAGTCTTCAGGGGGAT

>UN00700

GTTTTAATTTCGTTAAGTTTCTTTGGTAATTGAAGCTGTCCGTAAATCACTCCTTGATGAGTTGCGGGTGGTCATATCTTTTGATGGGTCTTACGTGAACTATCGTCACTTGGCTATTTTGTGTGACACTATGACTTATCGAGGTCACTTAATGGCCATCACCAGGCACGGTATTAATCGCAATGACACTGGCCCAATGATGAGATGTTCATTTGAGGAAACTGTGGATATTCTCCTGGATGCAGCTGTTTA

>UN00701

ACATCTGCTATAATAAATGCAAAAGCCTGATATTTCAGTACAAACAGATGTAAACATGTACAACCACTAAAGTTGTACGTAACTGATACTTACATTGCAATTACGACCTGAAATGAACCAGACAGAACGTAGGAGAGAGATGAATGAATTCAATGTTTCTAAATTTGTCTCCATTACATCAGTACACAAGTAAATCAAAGGGTGCACAGTTATACAACATAAATTTCCCGGAACACAGAATATAGTGTTCTTTATCAGTTTCAATTGTGGAAAAGCTCCACAATGAAACTTAAAATTCCACCCACAGTTACTCAACTCTTCTCAACTTCATTTTGAGGGGCACTTCCTTGTAAGAATTCACCTTCTCAGCTGCCCTTCTCATTGGCCTCCCTATCAACGATCTTCTTCTCGACCCCTGATTTTGATTTCCAGGAGAAACACTTCGATCCTTCTCGGTTGAATCCTGAGTGCTGTCGAAGCATCGATCTTCCTGTGAGCGTAGTGCAAATTTTATGTCTTCTATCTCAAACAAGCTCTCACTTTGTTCAGAAAGCTCAGTTTTCATGTTACAAGCCCTCCTCAAAGATTTCCTTCTGCCTTCATCCTTCTCCTTTGTCGTTTCTTGACGAGTTATACAGGTCGGCCCTAGAGATCGACTTCTCAGAGTGCGTTTTCTATCTATATCAGCTGGTTTTTTGCAGTAAGCTGGAGGAGGTGGATCTATCACAATTTCATTTGGCTTCAAGAGATTCTCCTCAGTGCCAATCTTTTCACCAAGCTTTTTGTATTGCTCGCTCAATTTCTTCTCCTCCTGTAGTTCTGAAGTTCTAGCTCTGAGGACTGCAACTGTAGTTCCAAGTTCATGCTGCATTGCTTTTAGTCTGTCTTTACCCAAATTGAGCTCCGCTAACATGTGAGAATTAGCCTGAGCAAGCTGCCAGTTCTGCTGATTGGCCTTCTGTAAACTTATCTTAAGCTTTTGCAACTCCGTCCCAGTAATTTCTACGATTTTATTTCTCTCATCAAGAAGCCTCAGAAGCGCATTATTTTCCTTTAAAAGCATCTCAATGTAATCCTTTGCCC

>UN00702

CTATTGGTCCATACTCTGAATCAATTATAAGGATACGCTCATTTCTTAAAACAATAAGGTACCATAATAAGAGAGAATTTTATCAAAGACGATACGACTTAGCAAACTCAGCCCTCGATCAAAAAGAGAAACACAAACTTTCTTTCTGCAAAATCATCACTTCTGTGAAGTCACTAGTATCCCATGATAAACGTAACATATTTGCTTAAGCTATGGTTGCAAATGGTTATATCAGAACTGCACCGGCTTATATAAGAATCTGCATCTTATTTGTTTCCACCCTTGGAATAGGAGTCGCGGAGAGTAACGGTTCTATTGAAGACGAGTTTCTCGGGGGTAGAATCTGGATCAACAGCAAAATACCCAAGTCTCTCAAACTGAAATCTGTCGCCAGTAGTAGCACTACCAAGAGAAGGCACAGCATATGCTTCTGATATCACCTCCTTCGATCTCGGATTAAGATCAGCAAGCCAGTCTTCTAGCTCAGCAGGATTCTCAGAAAGGAATAAGTTCTCAAACAATCTTACTTCCACCTTCAGAGGATCAACCCCAGGTGAAGGTTGAGCAACCCAATGAAGCACACCCTTGGGCTTAGATTTCTTTGAAGAATCATACTCTGCCCTTATCTCAACTACAGTACTACCTTAAGTAAAC

>UN00703

TATCCAGTTTGCATCACTAGAAGATCATCAAGTTACATTTGGGCAAGGATTGTCTAAGTTGCAAGAACATGCATCACAGTCAAACTAACTTGCCCAACGTCCTCGAAACAAATAAACAGGCATCGACGCGTGATTTTTTACTTCATCAGGCGCCTGAGAAGTGCTTGAGGTAGGCATCCGCAAGCTTCTGACCCAAACGGACCTGAGCCTGAGTGGAGAGGTGGATGTTATCTTCGTTGAGCGGCGACCCCTTCGCGTCGACGCACACCACATTTGGCAGGTCTATCCCCAGCTGAGCCGCCCTTACCTTCTCTACCAAGCGCTCTTCCCCTGATGCGATAGCAACCTGAATGAAAGGTAGGGAAGGCAAACTGAGTTCAGCCCTCACGTCTCCGATCAGCC

>UN00704

CCACAGGCATGCATTTTCCCAGGGACTTTACTTTTGTGCTCCCATTCCACATTCTCCTGCATCGGGAGAGCATCCATGTCACCCCTCAAAGCTACAAACGGAGCTTCTCCGGTCCCAACGAATCCGACAACGCCCGTTAACCGCAACCGGATGCTCATATCGAATCCCCAGAGAATCCAATTCCTTTCTGATGAGCTTGCTCGTCTCGAATTCTTCGTATCCGAGCTCAGGATTCTCGTGTATCCTCCTCCTTATTCCAACCATCCAATCTACGAACTCCGGCTCCTTCGCTCGCCGGAGAAATCTTCCGGTGTCGGATCCTGATAAAGATCCGAACCCGATCGAGCAGAGATTGACGAGGAGGAAGAGGATTAGAGCTGAGCACTTGGAGAACTCCATGATTGAGATTCTCAGCTTTTTGGGGTTTAA

>UN00705

CTTATTTGGGTTAAAAAAGTTTCTTTGGTAATGGCTCCTGGCTTAGAGAAGCTCAACATATTGCCTTTCAGAGTTGCAGCATTATGATACAGTAGCAAAGAAAATGGCATTTTTCGACCCTTCTCGAGCTAAAGATTTTCTCTTCATCTCTGGAACCAAGATGAGAACATATGCAAGGACAGGAGAGAGCCCTCCTGATGGTTTCATGTGTCCTGGTGGCTGGGAAGTTTTGGTTAATTATTACAAAGCTTGCAAACTGAAGAAGCTAACCAACAGCCAGCTACTGTATGATGATACGCTTTTTGAGAAGAGAGGAGCTAAATTAAATTGGAAGCTTTTGGTGCAGAATTTTTATTCGCTTTGAACTCAAAATTTAGTTATTCTGATGTTTGATTTGTGATTGTTTTCATTTTCCTATCATTTATTTTATACGCAAGTGTTTAATGATTTTGGTGAGAAACAAAGTGAAGCAAAATAAGATGGAATTGCGAAATGACAAGCAATCGCTTGTCAAAAATTTGT

>UN00706

AGATCTTCAAGAACAGCTGCAGTGCTAGCATCTTCAGGTGTGGCTTTCTGTTGAAGCGTTCTAGGCCAGTGAGATTTTTCAAGCAAAAACAGAATCTCTTCAATGTGCTCCCTATTCCTCCACATATTTTCATCCATTTTATGAGGAGGTTGAAGATCTGCTTCTTGGGCCAATGACTCCTCACCGGCCAAAAGAGGAGATTCCTTCACGAGATCACCCGCGATCCCTCGCAATTCGCCGGTGAGTCGAGCAACGTCATCAGAAATAGGATCGAGGGGGTACTTATCGTAGTAGCGAGCCAAGAAAGCCCTAGTGATCGGCACCAAACCCTCGGTAGAATTCGCCATTGACGTCGATCGACCGATCGAAGGAAAGGGGTTCACTAACAAAACCGCTCTCTTTTAGCGAATTGGGAGCAGGGCATTGATGGAATAACGAAAAGAA

>UN00707

ACCTAGTTAAATTCAGATGACTGAATCATCACTTACCCTGTAAGCAAGATATCAACAATTTGAAGATTAAAATCTGCTAACTGCAACAGCTTTGCACCAACTTTTCACATGACTTAATGCTTCTACTAATGAGAGACAAAGAGGATACCAGCATTGCTGACCAATGAAGGACACTTTGCAAATCAGACAAGAAAGGGACTGAAGATAAAGGTTATTTTGCTGGTTACGTCGAAGTCATCTGCGCATGTTAACAAGCGAAACGTTATGACAGTAAGACACATTTGAGTTCGGGGAACTCATTTATGGCCTCCTTAAAGTTTCCGAAATATCCATCAAGAACTCAATCAAATTCACATATCTATGTGTCAGCAATTGGAGCTTCTGTC

>UN00708

CAATCAATACATCGATAATAATAATAAGAAAGGGAATTCCTTAGTACGTACAGATCCTAACAAATTATATATCGAACAAGATACACAGAAAGAAGCAAGGAATGATTTCACCGATCTAAACCATGTTACATGATAGATTTTACTTCTTCAGCCTTTACTATTTTGATTAGCTGATTGAGGATCTCGTTGAATTGCTTACAATAAAAATTAGCCTGGAGTAAAAATGGCCATGATACAGAGATTTGATATACAGCTGAATGAGACATGAGCGACTCAGGATGTTGCGTTTGTGCGGTATAGAGATCTCTACATCACTGTACCGTTCTCTTCCTCTTTGATCTCTTGCCTTTTCTCGGTCCAGTATATTGTGGAAGCTGCTTGCTGTTACTAGAAGCATAGGCTTCTTCTAGTTCCTCCTTTGACTCTCCATCTGAATTATCATCAGAATCTGCTGTAGAAGCCAAAACCTCAACCTCTGCTGGCAAGGCTTTGTTCACCTTTATCTTATTTTCTTCTGCTTTCAATTGCTTAAACCTTTCACCACTTCGTGCTGGCTGAGAATTAGACCGTTTTGCTTGACCAGCACTAATGATTCCACCTCCATCTGCACTAATAGCAGGCTTTGCACCACCTAATTTCCTTAACCATATTTGTTGTGCTGTACTGAGAATGTTGTTTGTAAACCAGTAAATTGATAATCCTGACGGGACTGATAATGAAAAGTAACCAATCATTAGAGGAAGAAACTTGAATATGAGTTGAGAGTTCTTTGCAGCTGGATCATCACTCTGAGGTGGCTTCATGATCTCCATTGAAACGAACTGTGAAACAACAAGTAGTACAGGTAGAACCAGGTATGCTGCTGTATCGGACCATCCTAAAGGTGGATGTCCATCCACAAAAGGGAATAGCCAGGAAATGCCAGACCCAGTTTGTCGAGCAGCAATTG

>UN00709

GTTACTAAAATTACTTTCTACGAAGTCCAGGCAGGGGCTAAACAGGCTCAATCCATGTAAGAGGAGCAATCTTCAAGAAGGGGCAATGAATGCAAAGGCATGGGCGTCGAAGTCGTTGGCAACAGTATCGATTGGCGAACCGAGCACTTCAAGAGCACAATCAGCAATTTAAGGGTTAAATATTTACTCCCGTAACTACCTGTTGCCTTGTTTTGTAACAGTTGTCTGTATAGAGAGAAGAAAAAAAAGTTTGTTAAACTTGTGATGGGTTAGTGGCCATAATTCTTTTGCTGCAGACTCATTTAGGAGAGGAGATGATTTTAGTATTTCAACTATTGCTTACCCTTTCAGTAAGCTCCTCTTTGTAAGCTGTTGTTGTATTGTTCTGGTATTTATTAATTTAAATCAATTGCCCTACC

>UN00710

TTTAGAAGAACCCAAATTACGAACTTTACTGAAGAGATGTGGGTTTAATAAGGGGCAGCTTGCCATGATTGTTACTGGGTATCCTCTTGTGTTGATAAAGAGTGTGAAGAATTGCTTGGAGCCAAGAATTAAGTTTTTGGTGCAGATAATGGGGAGAGATATTGGGGAGATTACTGAGTATCCCGAGTTTTTTCGACATGGGTTAAAGAAGAACTTGGAGTTTCGGTATAAGCTTCTTAAAGAGAAGAATGCAAGCTGTAGTTTGGTTGAGATGCTTGATTGTAACAAGAGGAAATTCTTCCTTAAATATGGGGTTTGTTGATTGTCAATTGTTTTTTGAATTCTGTAGTAGATTTGAACATTTTGATGAGTAAGAAGAGTTTGTCAATTTGAGCATGTTTCCTTTGTGTCAGGAACAAAGAGTTGAATCATATTTCTGTGTGTTATCATGTACAAAGTTTTAGCATTTACAGATTGCAATGCTTCTTTTATAAATTACAACTGCAGTTGCAGTTTTTAA

>UN00711

ACGACAGTTCCCTCTAAAAAGATAATATGCTAAATTATTTGCATAGGACATATAGAACACAAAAAATACGAGAAAAAAACACAATTATGTGCAAAAAACAAATGGAGAAAGCCATAAAAGAAAACTATTGATTAAAAGGTTCTACATGGTGAACCCTTGTACAATATTTATACAGTAGTTAATAAACTTAAGCTGGACTAAAAAACACACTTTATGGAAAGATCAGTGTGTTGTCCCAGCTCCACCAACAAGCAGTTAAGCCCCAACTGCCAGGACAGCAGATTTGCAAATACTCATTCTTTGTCTACAAAAATCTCAACATCAGGCAACGGGTCATATAGTTTCTTCTCTCTAGTGTCGAATATGGTAAT

>UN00712

TACAATATTCCCTTCGCATCAACCGCACACAGCATTTCCAACAAAAAACCCCAAATATATACACAAGGGAAAAAGAAAGAAAAAAAATCAAAGTCGACGAGTTTTTGGAAACCCTGGGGCGCTAGTAGATCTAAGATCCACGACCAAGATCCGAAGCAAGACGAGATGCAATTGCTGAATGATACATGACATCATGGAAATTCTCCATCTCCACCGTCTTCATCCTCAGCTCCCTCGCCTTCTCCTCCGTCAGCACCGAGAACCTCCTCCGCCTGCTCTCCGCCACCTCCTCCGGCGGGGACGCAGCCGCTTCCACGTAGTCTGGCGACGCCGGCCACCCGAACAGCGGCGTCCACCAGTTCCCGCCGCCGCCGCCTCTCCGCCCTTCCGACCTCCGATCTGCTTTGGAATTCGCGCTCGCTATGACCGGAGACGGCCGAAACGGGATCAAACTTGAACAGGCCATTGGTTTTTTCTTAAGAG

>UN00713

TTTGTTCCTTTCTTGATTGGGTTCGCAGAAGGATCTGATGCCGATTGCTTCGGGGTTTCTGATCGGAATGCAATGCGCGCTGGCTGGCGCCTTCTTTTCCACCTGTACGTACCTTGCTGCAGAGATACCGACCGATCGGTGAGGGTTGAGAAGAGGCAACAAGGGAAGAAAGCAGAGGGGGCTGAACTTTTTTTTGCATCTAATTCATATATAGCCGTGATCAATGTAGAGTTGTGATTAATAGGGCCAAATGAACCTTTTCAATTAGTAATTGTACAATTTCCAAAATTTTTGTATCTTA

>UN00714

AACTTCAAAATGTTATGGACAATAGAAATAACTGCGTGATGTTCCATGGAAAATTAAATTACAATAAAGCGCATCAACTCCTTTGTGCAAAAATATATAATTAAAAAAAAATCAGGCAAACATACAATTTCAATTGAGTTGGGCAAGAAAAGGAACAAAAATTTGGCCGCGCTAAACTAGCTTATGTATAAATAATATACACGTACGTGTTACAATTAACTCAGGACTTACAAGGGCTTGAACACCATCGAAAAGACGAAATTGTCCCATATCACGATCATTCTTCAGACATTGCAATACCCCGCAACACTGGGTAACACCGGCTTACCGTCTTGAAGAAAAAAAAAATCAGTTGAAAAAAAGCACAACTTCTGCAGTGGAACCTCGCCACTCATTCAAAAACCGAGCATTGGTTTGATACTGCTTCATCATCTTTTGCTCTCACGTATAACTAATTCAGGGTTGGTGTATATCCTTCTGAAGATTCCATCTCTAAAAGCATCAATGATTTCTGTCTCTGATACCGGTTTCTTTTAGCAGCAATGGATATTAAGTCTTGCGGGAGTCCGAAATCATAGTCATTTTTTCCTCTTCTAATGCAGACGATATAAATTAACAGGACAATCAATGCGAGAACAACAGCGCATGCAAGGCCTATAGCAATTTTTACACCTGTGGATAACCCTTTGCTCCAGAAAAAGGGACATGGTGGCAGAGTTGGTACACCACACAAGCCTTTATTACCTGAAAGATCTATAACTCCGCCATGTACGCCAATTGTGTAAAGTTTATCGGGAACTTGTCCATCCAATTCGTTATTATTCAACAACACAAGTTGGAGTTTGGATGAGTCTAAGCTGTCGGGAATACTACCAGAAACT

>UN00715

CAAGCTATTAGCTAGCTACCTCAAGGGTGCATGATTTCTATCTTTACAAACAGACCATCCACCTCCCAACAAAAATATTAATTAATAGAGGGGGAGAGAAGAAACATATCAAAGTAAAAACCCACAAAATTAAAATTACTCTCACAAACCAATCAATCGTTCCCATTGATCTCCTCAATACTCTCAAGGTGGGGCCTCCAAACTCCGACCCTCCCTCTCCTCCGATCCCTCTGCGTCGAGACCTTCTCCGACAAAATCTCCGACAAATACCTCTCCGACATCAGAAGCCTCGTCCGATCCTCCGCCAGCTCCCCATTACCCTCCTCCGCCCTCTTCTTCCTCCTCCTCCTCGCCGGCGGCGACGCCCCGCCGCCGGCCTTCCCGCCTTCTCGGAATTCACCGGCATGAGGAAGTAAATCTTGCCCCTCTGCAGCTCGGCACTGGGGGGGAGAACGAGGGACTTTGGGCTCTCGGCGGCGGCGCCGAACTCGTCGAAGGAGGAGGAGGAGGGGGGCTTGCGGAGCACGTGCTTGGGGTAGGCCTCCATGATGTCACGCGCGGAGACGGGGTGGCTGATTTCCTCGACGCGGCCGCTGGCGTGGACGATGCGGATGACGTCGAGGGCGCCGCAGGGGAGGATGCAGGAGATGCAGCATCTGATGCTCTCCTTCATTTTTTTTTCCTCTTTTTTTTTTCCTTTTCGTTGAGACTCTCGAGTTTCACTTTTGTTACTATTATTGATATGAGTTTTGTTAGAGAGAGAGAGAGAGAGAGAGAGAGAGAAGGGGGGGTTTTTGGCGAGCGGGGGTTTGGTTGTGGGACTCAATGCGTTTGATTGCGAGAAGTTTAAGGGGAGTGGGAGGTTGAGGGTGGATTTGCTGCCGATCGGAGGGGGGCTTAGGGTTTGCGGAGTGCCGAAGAGGGAGAGACTGAGGAGTCGGTGGGTGGTGAGGGTCGATGAGTTCACCAGAGAGGAGCATGTTTATAGGATCGCACCACCAGTGCAGAGGAAGATGATTAGAGCTTAGAGCCTTTTTAATAGTGGCTCTTCTTGTTGTAGCTAATAGGGGGAATGTAGAGCCTTTGTGTGATTCTAGAGTTGTTGCAAGATGTATATATGCTTTCTTTCTTTCTTTCATCGAACCTAGCCCTCTGGGAGCAACACGGCTTTGACATGTGACCTGTTGGGCCGGCCTTGATCGTTTTTCTTGCTTTTGTTATTGACGTTTGTAGATGTAAATGTTGATATGAAGAAGATTTTTAAGTCC

>UN00716

AGAAGGCCCACCAAAGGTAATACCCTTGTATCCAATAATTATTATTTCTTCACTTAGAGATATTTGGCCATATTATATAGCCGTTCAGAACATACAAATACATACGTAACAACACTGATCTCCCGCTCGATTAACATGAGCCAATACAGAGTAAGCAAAGGAAAGAAAAACACGAATCAAACTTTTAACGAACGATACATTTGAACGGGCGATGCGAAACACTAATGGATTCAAGCAGCAGCAACGTTAAGAAACTGCTCATCCCTCTCCAGATTCTTCATTGCCTCAGGGTCCAGAGTCAGCTCTGCATCAATACCCCCATCCCTCCCCGGGTACAGGAACATGACCCCATCAAACTTATTGTTCAATCCGCTCCGCACCCGCTCCGGACGCCCGAACCCGAACTCCACATCGTACACCCTGAACCTCGGCGAGCTTCCCACCGGCACCGTGTTGACCCCTGCGTCGGTGTAGTGGAACATTTTGGGCTTTGCCTCGTACTTCATCCAACCTCGCGTTGACCGCCTTCGTGTCGTGGGACTCAATAACCTTCTGCAGTAGCCCAGTACCGAATTCGGGCGGGCTCGCCTGCAGCAAGCCCGACGCAGTGCCAGTGAAGATGGCCTGGATGAGGTTCCCAAAGTAGGACTCGGGCATTGGAGGGTCGATGCGAGAGCGACAATTCATGAAGATGGCGAAGACGGTGATATCCTCGGGCTTGAGTGCACGGGCACGGCAGACAGAGCGCCATACGTGGGCCCCGAGGGACTGGAAGGTTGAGAAGGGTTTGGATTCAGGAGGGAGGTTGGCGTTGGCCTGGGATTTGAGTTGGGAGAGCGCGGGTTCGGAGAAAGAGAAGACTTTGGCGACCAAATGTTTGGGTGGGCCGTTTGGATCGGCGAGTTCGTGGGCTTTTGGGGAGGAAGGGAGGTCCACTTTGACGCGGGTGGATCGGGCTTTGGTACGGTCGTGGAAGGGAGGAACGGATATAGTTGCAGATGGGCCGCGAGTGAGGTCGGCCCATGATGTCATAAAGTGCCAGGTGGAGTGTCCGTCTACAATAGCATGGTTAAAGGCGCATCCCAGTGCCAAGCCGTTCTTGAGCTTTGTGAACTGAACGGCGAGCAGAGGACGGTGAAGGCCCTCGAGGTTCATGATTCCGGTGTAAGGAACCACCTCCTGAAGCAATTCCGTAGTCTCGCCCTCGGCGAGCTCCTCCACCGACACATCCTCTGCGGACGCATCAATAACCTCAGCGCCGACCAAGCTCTCCCCGCTGCAGTCCACGATCAGAGACCCGTCCTCATCCTTGGCAATTTTTCCCGCCAACGGATAGAAGTAGTCGAGAGCGGCCGCAAGACCCTCTTTCATCCTCTCCACGGCGCCCCTGAACTCCTCGTCGTCGGTTGATGTTTTGTACAGCAATAGCTTCTGATTGTAGTAGAAGGTAATGTACGGCACGTCGAAAGTGATCAACGGGCATGAAGCGGGTCTCTCCGTCTCCGTCCTATTGCTCGGGGCAACCATGCTCGTGTTGACCACCTTCACCTTCACTGGCGCTGTAGTAGTCTTCCTTCGTTCCGCATTGTGACCGTCGTCAGCTGCCATTGGTGAAAGGTTTTGGGAGCGACAGAGATCAA

>UN00717

ACTTCTAAAAAAAATAGATCTTTTATATAATTGATATAGGATAGTAGATGAGAGTGATATGTTACAAGAGATAATTCACCTATCACATCATGATACAATAGTATGCTGGTATTACGTTTCTATCTGGATCATTGCTCTTTCCTTATCTGATTGCTGATTCTCCGAACCCTGTAAATTGCGGGCTGGAAAGCAAAAGAATTACAATGCGGAGATGTGAATATAAAAGGAAATTGTTAGGCTAGACTCTTCTGTGATGCTTGTTTTTGCTCTTTTTAAGCAGCATGGACATTGCATCAATGCCTCTAGATCCTGATGAGTTTCCGCCCATTAACCATTCCAATGCCTCGGCTGCAGCATCCTTTTCTGCAAGTTTCTTATTGCTGCAAGGTTGTCCGACAAATTGCATCCCATTGAACTCAACAGTGGCTCTGAACTGATTGTTCTTGAGCTGCTTTGTTTTGTAAATGGGGTTATCATGTCCTGCTCTTGTTAGAAGAGTCTGGAGCTGGCTCTTGGAGTTGTCGCCTCCACCGCCGCCGCTGGATTTGGCTGAGAGCAAGGATTTAGGTTTTCTTAGGGTAGTTAAGTTAGTTAAGAACCG

>UN00718

GTTAACCCCTTTTTAGTTATTAAACGTATTAAAATAGTAATCACATTAAATTCTCCTGGTTTTATTCTCAAATTGTCAGGAGAAAATATAAGAGCCCCGAAAGAAAGGAAAGAAAAAAAAGAGAGAAGGGAGGAAAAAAATTACATAAACAAAAGTTCCAACTGTTAAGCCCGCTCGCAGGGCTCACTCGACAGAGAGCTCGCTATCCAACCCCGCAATTTATACATTTTGTGACCCCAAGACTGATGATTTCCTTCTCAATCTGCTTCCATTATACTGGCTGCTACCTCGCAGGTATTAAATTAAGCATATCTTCTGAAAAATGTAAGAAGGTTACAGGAAACGAACAAAACATAGAAAATTTCCCAAACTTAGGATTCATCAATGCCGAGATATTGAGAGTGAG

>UN00719

GTTATCGACTATAGATTGCTTATTAATATGAATATGGTGTGTCTCTGCGACAAAAGTGGATGCCGGAGCTTAGACATTATGCCGAGTCAGTCCCCGTTGTGCTTGTGGGCACCAAACTAGATTTGAGAGATGACCCACAATTTCTGACGGACCACCCTGGTGCATCTACTATCTCAACAGCGCAGGGTGAGGAACTAAAGAAGCAAATAGGAGCATCGGTATACGTTGAGTGTAGTTCTAAGACTCAGCAGAATGTGAAGGCTGTTTTCGATTCTGCGATCAAAATCGTACTTCAACCTCCAAGACCCATAAAACAGAAGAAAAAGAAAAAGTTGTGCCCGATCCTTTAGTTTCTGCATCCAGTCAGAAAATATACTAATGGAGCTGCTGCCTATTTAATTATTAGTGACAGCTTAATTTGCAGTTTACATTTGTGAATAAAAATAGTTTCAATGTCTAAATACCTATT

>UN00720

GGTCTTTCACAATCTCTCCGTGTACTGTCTCAAATTTCCAGTGGCAAAATGGTAATTAACCAAAGACGACAAGGCTATATATATACTACCAGTGGCAAAACCGTAATTACTGCGGAGTAGGTAACAGGAGCGAGGGGATCTGCTCACCTCCGGTAAGAAACGGGGACAATACCAGCGCGGTACTGGGCGATCTTGAGAAACACGGGCATCGCGAGGTCGAAATGGGGCCGCGGGGGGTTACACCACCCGCCGTTATCCGACGGCAGAGCGAAGTTGGGCGGGCAGAAATTAGTCGCCGTTATAAAAATGGACGGGCTGCCAGCGTGGCACCACCTGGGGTCGTTATCACACTTGATCTCGAAGCAGGCCCCACAGCTCAGCCCCTCGTTGAATAACGCCGTGCTCAACGCCGCCGTCTCGACGCCGTAGCCTTGGCTGTAGAGGTTCCCGTACCCACAAGCCCCTCCCCATTGTGCCGGAGGCGTCGCTGCCGCCGTAGAAGGTGGCGTGGGCGGACTGCCAGGGGCCGGGCGGAGTAGACGCCGGGGACGCGGGCGGAGGAGAGAGAGAACAAGGAGAATGCGAGGAGAGAGAAAACGAAGGGTCAGTGAGAGAGAAGTCATTTTGGGTAGGAGAGAGAGAGAGAGA

>UN00721

ATAACAAAGGGATCACAGTAATATGATTCAATGATGAGTTCTAGAGAGGTTCAAAAACAATGAGCTTATCATCATCTGAGATCCACAAACATCAAATTTTATACAAAATTATGGGTTGAAAAACTGACCAGCGACCACTATTGAAATACCCACAACATTCCATTATCAGATGTACAACAAAAGAAGTGATTCAATAGTGAGTTCATAATGACGTTTCAATACAATGAGCATTACCATCATCATCTGAGATCTACAACATCAAATTTGAGTTTAGGATTCATGTATACATCGGAACAGCGGTCATAGATTGGACCGCCATCCGATGGCTCTGGATGAGGATGGAAACCGCGCTGCTGGCATTGCCTTATTACCGACATGCCACCAGGAGCTGTAAGCCTAAATATACCATGTGTCCTTGAACTATCTCTTGGTGCCATAACAATTGCAATGGCTTCAGGTAACATAATCTGATAAGAATAATGGGTGTGAACATCGATCGACGACCTGAAACACGACTGTGTAGGATGTGTGTGAATCCACCCAAGGGTGAAAAGAGATTGCTTATCCTGAAACTCAAATATTTCCTCCTCATTTGTCGTTGAACAAGAATCAGAGGTTGACTCCTGTTTTGGGATGATAAGAGCTGTTACAAAAATTTTCTATTTTTAAGTGAACCTGCAAGAACTCCACAAGTTTCTAAGTTTCTATCAGTATTTGACTTAGCAACCCTCATAAAGCTGTCCATCAACACCGCAGAAATGTGAACTTGCAAAGGAGATTCTGTTGTCGGTACTATTCCTTCGAGAGAATTTGTCTGTCCAGATGTAGGGTACAAAATTTCAGATGTGATAGCAGCCAAATCTTGTACTTCTGCACGAACAGGCGGAGGAGAAGA

>UN00722

TTTAAAAGTTAAAAGTTAAAGGTAAAAACCTAAAGTACCAAAAAAGAAGTCCGTGGCAGGACATAAGGGTGTTGACGATGGAGCAGAACGGCCTCCTGAGTGCAAGCAGCAATGAGTTGTTGTGGAAGAAACATTTTGGACTGTCAAGCATTATTGAGTAGTTTTGATGGTGTTGTCTGAAGGGGATTGAGTTGAGAGTGATCTTCTCTTATCAAATCCTGATCCTGTTGTAGTTTGAAAAGTTTGAGGTTGGTTTGGGGAACTATAGATTGGTTGTTGGTGTGTTTTTAGACTATCATTCGTAACGTAGTAGGTATGCAGCCCCTTCTATTAATTCATTCAAAACCCGTGTTTATTCTTTTTTCTCAACCCATTTCGTTTTGCCAGTTTTAAGGTTTCAGTGTTGATCTTGGGAATATCGTCTATCCAGAGTTTGAATGTGTGGCAACAGTTTGAAACGATACTTAATTAATAAATAACTAAAAGTAAAATAAATTAAATTAAGG

>UN00723

TCATTCCCCCAATTCTTGGACAGGGTGGGTTAGATCCTTATTCAGCAGATCTTGAGCAAAAACGCCTGATGCTGCAGAGGTTCCAAGAAGAGAATCCAGGGTTTGATTTCTCTCAGGCCCAGTTTTCAGGGACCTGCCCAGATCCTCAGACATTCATGGGTGGTATTCGCTCCGACTGAACAGCACTTATCCCCGAAACGCCACTAGCTTCTGATATCAAGCATGTTAGTGGATTAAAAATCTTATTTATATCCTCTCTAATGTCTGTACATGTGTTATTGTAAACTATTCATACACTTGTCTGTTGATTCTGATACATTGTGACAATTTCTTGTAGTCGCTTGCTCATTTGATAATGTGCTGAAAAGTCCACCG

>UN00724

TTCGTGAGAACAGCAATTTCTTCAGGTCAGAACTTCAGAAGATGGGTTTTGAGGTTCTCGGGGACAATGATTCTCCAGTCATGCCAATAATGCTTTACAATCCGGCAAAAATCCCAGCCTTTTCACGAGAATGTCTGAGGCAGAAGGTTGCGGTTGTAACTGTTGCTTTCCCTGCAACACCCCTTCTCCTCGCCAGGGCTCGCATTTGCATTTCTGCTTCCCACTCAAGAGAAGACCTTGTTAAAGCTTTAGAGGTCATCAGCAACGTTGGTGATCTCATCGGCATCAAGTACTTCCCAGTTCGAACCTCACAAGACCCATGCTGAAGAGAGGAGGAAGAAACTAGAGTAGGGAATTCTCAACATG

>UN00725

TTTTCCTTTTTCTTTTTCTTTTTCCTTTTCGTTTAAGTTCCTTTACTTTTAGTTAAAAACCTTTGTTTACGTCAAAGTACGATAGCCTCGCTTATTTTGACCGGGTGGTGACCGGAAACTTGAGCTACGGGGTTTTAAAAGGAGTCGATGGGTTCAAACAGGAGGAGCTCTTTGTTTGGTTGCCCGTTAAGGGGATTTTTAATAAGAATCCTGAGTCCGGAGTGATTTTGTTTGACATCGGCCTGGCTCATAAGCAGTTATCGATGAGCTTGTTTGAGACTCCGCCTGAGTGTAGGCCTGATGGAGTGCTGGGGGATAGCGTCGGCGGCTCTCTTGGCAGGAGAGGAGGAATCCATGAGCAAAGATGATGATAATATACAAAAATGTAAAACAAAGAGCTCTCAGTTTTTCCTATTTTGATCTTTATGTCTTCTGTTTTGTTTAGCTTTTTAGAGTTTTTTGGGAGAATTTCAAGGTTTGTTATGGACTCTGCTGTAAATGGAAATTTGATAATCAAATGTCTCTGGGGAAGTTAGAAAG

>UN00726

AACAAATAACCGAAGGAACCGACCGGACTAGGGACCTAGGGCCTGTCCCTGATGGTAGTGGCCCATGGGCTGCTGTTGCATCTGCTGCATCGCCGGCTGCATCGCCGGCGGCGGGATCTGAGACGGCTGCTGGTTCCACATCGCCGGAGGCGGCTGCGCCACCGCGTACTGCTGCTGGTACTGCTGCGGCGGCGGTGGGGCCATCATCGGCCACTGCTGAGGCGGCGGTGGGCCCATCTGATACTGCTGCGGCGGCTGCGGCTGCTGCTGCTGCTGAGGAACCGCTGGAGGTTGTTGCATCATCTTGAAACCCTAATTCGAAATTAATTGAAGAGGAAAAAAAGGGTGGGTTTGGAG

>UN00727

TCCATTCTGCAATCTTCAAGCCTCCGAACGTCAGAGCTATCGACAACCCAAACTAATCCATCAGTCTGCTCAAAGTAATTCCTCCAGTATGACCTAATGGTCTTCTGCCCACCAACATCCCATATGTTCAATGAATACTTTTGGTACTTGATGGTTTTGATGTTGAATCCAAGGGTCGGGCTGATGATGCTGGTGTCCTCACCATTGATTCTGAGAACTATCGTCGTCTTCCCCGAGTTATCGAGCCCCACCATTAGGATTCGCATCTCCTTCTCCTTCCTCTTTATTTTCCTTATGATGCTCAGAAGTCCCATGATCGCTGTATTATTGTAGTAGGGTAGTTAGTTAGTTTTGTTAGGTTTTCTTTTAGTTTA

>UN00728

ACTCCCAGCTTCCAGCGCTCCCAAACTCCTACGCCTACAGCACGATCTCGTCGGCCCCGCCGAAGAAGCGAGCGGGTCGGACCAAGTTCCGGGAGACCCGGCACCCGATCTTCAGAGGGGTGCGGAGGCGGGGCGGCGGCGGCCGATGGGTCTGCGAGGTGCGGGACCCCAACGTCAACAAGTCCAGGATCTGGCTCGGGACCTTCCCCACCGCCGAAATGGCTGCCCGGGCCCACGACGTCGCCGCGATGGCGGTGCGGGGCCGCAAGGCCTGCCTGAACTTCGCCGACTCGGCGTGGCGCCTCGCCGCGCCGTCGTCGAGCAGTCATAAGGATATACGGGCGGCGGCGGCCGCGGCCGCTGAGATGTTCAGGCCGAAGATGCAGCTGGAGCAGGGCTCGCCCCGGG

>UN00729

GTCAATATCCAAGCTATTCAAATTAGCCCCAGAACGTTCTCATAGCAAATCAACTGATTTACTTTCCTCCATAATTTGTGTTACCAAGAAACAACAAATATGACATATATCCATGCTCTATAGACAACCAACATACCCACACTAAGAGTTACATACTTTTAGACTGAAAACACAGTGAATAAACCACAAGCCTTATGAGACAATGTTCAACATCGCGAAAAAGACCAAAATGAGATCCTAGATTTTCACAAATCCTACTTGGTCTCAGAACAAGTACTCACGAGCTCCTGGACAAACAATCTGTCATTTACTTGTGAATAAACCAAGACAAG

>UN00730

AGGTAAAACTAAAGTCAAAATGGCCCAGACGTATTTTGTAAGAGATGGTTCAGAATAGAGAAATTGGATATCCAATCGCGATAGTAAGACCCCCTAAGATTAAGCAGGCAAATCTGGTCGATAACCAGGATGATATGCTTAAGTGGGATATACTTAGGAGGGATGAAAAAGGGGGTCACCCTCCGAGGGCTAGTATTTACAGAGGCCACCCACTGATCAGAAACCCATGTTCAGAAGCTGGGAAGTTGATAAGGTTGCCAAGCACAATGGATGAACTCAAGAAAACTATAGGAGATAAGTTCAGAGTTGATGG

>UN00731

AAGCATAATTATGCACAAAAATAATAAATATAGCATACAAATAAAACCAGTTCTAAAATAAAACATCACCACCTCATCATGTGAATTACATCGCACCAATGGCCCCCATACATTACAAACACATCAACACACCCGTACAATACAAATACATCAACACAGCGATCCAAGATAATTCAGTGAAGAATACAACATCATCATTCATTGCAAACGTATATCAAGAGAAAAGAAAGCCCAGAACGAGTGCAGACACAAGACCAATCTGAGACAATACAACATGACTAAATGGAGCAGCGCTAGCATCAGCTTTCTCAGAAGCCTGAGGTGAATCAGCAGGGCACGGAAGCCCTTGCTTATCTCCCTTGCACTCGCTGGCAAAGAGACCTGGAGGGTACTTGCCATAAAGGTTTATGTAACTGAACATGGTCGAGGCACAGTCATTTGTGGCGTCGTTGAGCTCGTCCGCATAAGGGCATGCAAACTCTACGAGAGCACTACAGCAGAGCTTAGCAGGGTATTGGGGTCCCTTGCACTGGCTTGTGATGATCGTGTAGTTCATGAACTCGAAGTTTACGGGGCAAGACTTTCTCGTCTGGAGCAGATTCCGACCAGTAGATCTCTGAGCTTCGATCACAGGATCTGAGATGAAGGTGGCGGAGGCGGCAAGGCCGACGGTGAGCGCGACGAGAAGCGCCTGGAACAAGAAGATCCGGTCGGGATCCATGGATGGCTGTTCCTAGGGTTTCTCTCTCTCTCCCCGTGGACCGAATTCGGTCCACGGTAAAGTTTTAAACAATCAAACGTGCAATATTTGAGAAGACATTTATTCATATTTAGCCTGTAGCATAGATTATTTTCTGTTCTCATTTTTTTCCCCCTCTTTTACTACTACTCATAGCATGCTTGTGTCTTTG

>UN00732

CTCCTACATGAAATGTTCTATGGATATACCCCATTCAGAGGGAAAACAAGGCAAAAGACCTTTGCCAACATTCTTCATAAAGACCTCAAATTTCCTGGAAGCATTTTGGTTAGCGACCAAGCAAGGCATTTAATGCATCGTTTGTTGCATAGAGACCCTAAAAATAGGCTGGGGTTCCTCTGAAGGTGCTCATGAGATTAAGAAGCATCCATTTTTTTAATGGTATCAATTGGGCTCTTGTACGGCGCACGACCCCTCCTAAGCTCGACGCACCATTATCTTTCGATATTGACATTGAGAAGGAAGTTAAGCCTGCTGATCCTCAACTTGTGGATCTCCAAGAAAATATATTCTGAAAGATGTGACAGGTTTTTACTACTGCTTCTCCCAATAAGGGACATTGAAGAAATATTCAAGGATCAAATTTTGTATTCGTGTAACTTTAGAGCATTTGTTAGCAGAGACCAGAAATAATTTATGTATTGTCAATTTAATTTCTTTCCAGTGATAGTGCTTACTTCTGTATTAGATCGTATCAAGCAAATAATTGAGATGCAATTACTTTAAATTTTTAAGGTTT

>UN00733

GTCATGCAAAACATTTGAAATTTTCATATAAAGTAAGCAAAGAAAGAAAGTTTCTTTCCTCACAAGTCTTCATTGTATTATATATTCACAGTACTGTGCTAATCATTAAGATCCCAACCATATTTATCCACCAAGAAAAGAAGAAAACTTCCTTCAAACTTCCACCATAGCATCCCAGAAAAGTTAGCAAAGAGTTGAAGAAGGGTTCATCCCCAAAAATAGCAAAGCTTCCAGCAAAATTTACTTTCAGCATAGAATCAGCGAAGCATTCATCCTAAAACAAAAAATTTCAGAAGCCTCTTCAGCATTAAGTAATTTCAGACTTGCATACTGGACACCATGTCTTCCTCTTGAGCCAAGTATCAATGCATTCTTTATGGAATTTATGAAAACACGGCAAATGACGGATAGTATCTCCAGCAGTGGGGTTTTCCAGGCAGACAGCGCATGCTTCTCCAACGTTATCACTCTTAATTATTGATTGTGGCAAGTTGTTGATCTCACTTTCAGAAGCACCAAGAATAGTTAGAATAA

>UN00734

GGTTGAAACTTGATGTCCTCCTCAACGGCCAACCCGTTGATGCATTGGCATCTATTGTCCACAATTTGAACGCACAAAGGATTGGACGCCAGTTGGTTGAAAAGCTAAAAGAGTTCATAGACAGGCAAATGTTTGAGATAACGATACAAGCTGCAATTGGTTCGAAGGTTATTGCAAGGGAGAGTATTTCAGCAATGAGAAAAAATGTCCTGGCAAAATGTTATGGGGGGGATGTTACTAGAAAGAAAAAACTTTTAGAGAAGCAGAAGGAAGGAAAGAAACGCATGAAGCGCGTCGGATCTGTTGATATACCACAAGAAGCATTTCATCAGATTCTGAAAATCTCGTAAGGGTATATGGATACGGAGGAGTCGATTTCTTTCGGTCCAGTGATCAGCAGATTATGTTACCCAGAAGAAGCATATGATGACCCATAACAAGAGGAGTGAAACCATCTACAAATACTTTGAAGAATTATAAAAAAGAAAAGAAAAAGATTTCTGCTAACTTTTTGCTTCCAATTCGGAGGATATGGAGGAAACTGGTTACGGAGCCCCATAACGGTTATGCATACATGCAGGCGCATTGCAGCATGTAACATTTTTGTACCTGTTCGCCAAAAGATGTAATGTTTGTATTACTGCGTATCATTTTCTTGAGCAAGCAGGGCTGTTTGCAAAAACCTCCGTCAAATAAAGCGCCACATACATAGTTT

>UN00735

TTACTAAACCGTAGTCGAGAAAGAGGAGGTAGGAGGAGGAGGAGGAGACGAAGAGGATGACGAGGGGAAAGCAGAAGATAGAGGCACAGAGGAAGAACGCAGAGAAGAATCAGAAGCAAAAGGGATCTCAATTCGAGGCCAGGGCCGTCGCCCTCAAGGTCACGTGCCCAATCTGCAAGGTACAGCTAGCAAATCAAAACCAGCTTGGCGATCACTACAGTTCGAAACACCCCAAAGAGAAACCTCCAAGTGAGTCTGGATGACATCTCTGCAAGCATTTGGAAGGGGCTTGGGATGTATTAACTTCTAAGTGTATCGATCTTATTTGTTCAACTCCTACCTCTATCGTCTTTTGGATCATGTATGAAACGTTATGAGGTTTTGCGATGCTTATTATATGAATAACATGCACCGGATGTAATCGATGTTGCGTCTTTCGATTGTATCTGTACTTTGATGTGTATATGGAATTTTAGTAAGG

>UN00736

TCTAATGCTACAAGAAGAAGGAGCCAATGGTATTCTCAGGCTACACGCCGAGAGTTCCCGCACAACTGGTGGTGCTACGAGAAGTAGTGAAGAAGATGAGCTTTCCTGTGTACCTTCAAGAAATCACGAGCATGTCTGAGCTTCGAAGGGATGGGCATCCATCGGTGTTTAGGATTCCTGGCGATACCCAGAGGAGGATGGAGTCGGTAACGCGCACTTCAGACTGTAGTCACTGGTGTTTGCCTGGAGTGCCGGATTCGTGGAATGAGATGTTGTTTGCTCTTCTGTAATGGAACTTGGAAGGGCTCATGTAATCAGGCTGGAGGTTTATTTAAAATAATGCAGCTGCTTGTTTGA

>UN00737

TGCATGGTTGTACAAGATCGGATTGGAGGGTGAGCATAAAGAAATCTACACCGAAGACGGTATGAAAAATGTTAAGTGGTTACAAAACTCAGAACCACCAAAGAATCAACCCCTCACATGGTACAAGGCAGTTGTAGATCCACCAAAAGGAGATGAACCAGTTGGACTTGACATGAAGTACATGGGAAAAGGCCAGGCTTGGTTAAATGGAAAACCCGTTGGAAGATACTGGCCACGAACAAGCTCTATACATGGTAACTGCAGTTCTGTTTGTAAGTACAGAGGCAAATTTTTCCCAGACAAATGTCTCACAGGATGTGGAGAACCAACACAGAGATGGTACCATGTCCCTCTCTCTTGGTTTCAACCATCAGGAAACATTCTAGTGATCTTCG

>UN00738

CGAAAATCCAAAAGGGATCATGTGGATAGTTTGCCTCCTCTTAAAACTGATTTCCCAACAGACATACTGAACAAGTACAGAAAAAATATGTCAGATGTTTAAATAACTCTCCATTGATTAGAGATCCCAATAAGCAAACTCTACCCAGTCATATATTGAGATGTTTAAGAGATACAGAAGGAAACTAATAAAATCATGCAGATTTTAGGGCAACGGATGCACAATAGCAACTTCCCAGTAGAAAGCAGTGTTGGGTGCTACTGTCATCTCTGTTCACAATAGCAACTTTCCAGTAGAAACCAGTTTTATATATTCTGGTAGAACTCTCAACATCTATATCATGTGCATGGCACATCTTGTTTCAGCAAGTACGACATCAGCGGCCTGATCATGCTTCCACCCCCG

>UN00739

CCAAAACCTGATCTCGTTTCTTTCGCCGACATCTCCCTTCTTGTTGAACTGTAGGGGGGAGGTAATGGGAGATCGACGGATCCAAGGTGGAGATCTCGTTCGCTGGGAGGTTTGCAAGCAGCGCCATCGCTGCTTGTTTCGCTGAGATCTGCACTATTCCCTTGGACACTGCCAAAGTCAGGCTTCAGCTGCAGAAGAAAGCAGTTGCGGAAGATGCAGCAGCTTTACCTAAATATAGGGGAATGTTGGGAACTGTCGCCACTGTTGCTAGAGAAGAAGGTTTGGCTGCACTGTGGAAAGGCATCATACCTGGATTGCATCGTCAGTGCATTTACGGAGGGTTGAGAATTGGATTGTATGACCCGGTTAAAGCCTTCTACGTTGGAGATAATTTTGTTGGAGATATCCCTTTGACCAAGAAAATACTTGCTGGGCTTACGACTGGTGCACTAGCAATTGTTATAGCGAATCCAACCGATCTTGTAGAAAGTACGACTTCAGGCTGAAGGGACTTCCACCTGGTATTCCAAGACGCTATTCAGGAGCTTTAAGGCTTAT

>UN00740

TTAAGGTTTACGTTTAAGGTTTGGTTAGTAGGTAGTTACGTTGGTAGTACTGGGTATTACGAGGATCTCAACTGCGTCGACAAGCAACATCACACGACCGGAACTGGGTTCATCAAAGTGGAAAAACCCGCAGACGGTGGTTTCGGTCTGAAGAAGCCAGCTGTTTCCAGCGCAGCTGTTTACCAACCTGCTCACAGAGGGAATCCGGCCACAAACGAATGGATCCCATCCGCGGACCCTGTGATTCCGGTTTCGAGCAAGCCGAGCAGGAGTGATATCTGAAGCTTCTTTTGGTACGCGTGCGTGCCATCTCAGCGCCGTATGTAATATGTTGTGGTCTCTTAGCATCGTGTGCGCGATTGGTGTGCGAGTCGTAGCTTCTCTGAGATGTACGATGAGGTTGAATAATCGTCTGTTGTAGCTTCTTCTGAGTTCCTTCTGTGCAGCTTAATAAGAATTCCTTTGGTGTGACCACCTGTTATTTTAC

>UN00741

CTCGACGAGGAGAGGCGGATAGAGTCATTCCTAACTTGAAGCCAAAGCATCTGTTTTCTAGGAAAGAGGTCAATCGGAAAGACTCAGAGACGTTAAGGAAGCAATTTCGTCAAAGAACAAGAGATTCGTTTACTTCTTTGGAATTTTTGTTTGAGAGCACTTTTGTTTAGTCTATGTTGATTGTTGTTTTTTATATTCCCCTTATCACTCATGTAGCTTATTTAAGGTACCTACTATGGAGGTTTTTCTTATTGAAGGATTGTTTCGGTAAGATAAAATGATAGTATTTGAATGGGTTTTTTTTTTTCCCTTTCAGCTTTGTTAAAGTTGGAACTTTGTGATATGTGTAATGACTTTGTTCAGGGACTTGTATCCTGTTACGTGTGATCATGTCTATGAAGACGTCCATCCATTGTTGAAGGTTTGAACCAAGTCGCCGAATGATGAGATTTCCATGCAGATAGCATCTTGGATTGTATTTTATTTACCGAAGTTTCCTCTTTGTCCTGGATAAAGCATTGGTTGTACTCTGTTGAAGCATTTCCATAAGACGTCCTTATTCATTAACTGATGCACTTGCTTTGGGTTTCCAGCTTTACATTCTTTTATTATAAAATTGTTCTTAGAGTCTGTTC

>UN00742

CATTCTTGTCATATAATTGCAACACAAATAATAAGAATATTACACATGAAAATAAATTTTTTCCGTAGGTGTCTCTTCTTCCAATCAGGCCGCAAATAAATACAATCTACAACTAGGATTTAAAAAAAAAAACCAATCAAAGAAAGCTCGAGACCAACTCAAGCCTACGGGCTTGGAAGAATTTGCAGCGACCTTTGCCGCTGGATTCAATATCCTCGGTTTTCCTCTTGTACCCGGTGAACCCTACATTTGACAGTTTCAGCTGCCGTTTGCCATTCCCATTTCTTGACCCTTCTTCATCCTCTTTATCAGCCTCCCAAAGAACCTCAACAGAATCTTTGAAATAGGGGGTGCATGTACCCCAACTGAAACGCACCAATGTGGGAAATCCAAACAAAGAATGCTTGTGATGCTCCAACCAAGCTTTAGTCTCGGGATCTCCTGGATATCCAGAACCAAAGTTCG

>UN00743

GGTTCTTTGTAAATGCTCCCTGGGTATGGTTACTCAATTGCACGGATGAAAAGTTTGAAGAACGAATGAGCTATGATTCGATAGGGATTGAGGAAATGGAAGCAGAACCATCATTTGACATGAATACATTTTTAGAACCAAGGGAAGAAGATGAATCTGAAGATGAGTATTCTGAGTATGATAAGGACAGTGAGGATGAGGATATGTGAACTCCATTTGTGCAGTTCTCACTGTAGGTATTTGTTGAATACACTTTTCCATATCAAATGACATCAGAGCGCCTTAGCTGAATCCTTAAGAGATTTTGACTGTGAAAGCTAAG

>UN00744

GGATGCTATAAAAAAGATCGACAAGGACAAGGTGAAAGCTGCACTGGAGAAACGAAGGAAGGAACGAAGTGAAGTTGCAAGGAAAATGGACGTCATGGATGATGATGACCTTATTGAGAGAGAGCTCGAAAGTGGTATTGAGTTGGCAACTGAGGAAGATAAGGTAAAACAAGAGAGGAGGCAAAGCTGGTCGAAATACAAGCAAGAACATAAGAACCAAGACCATGGTATTGAGAATGGAGAGCTTGTGACTGAGAAAGAAATTGAGAATGCTGAAGAGGGGGAATTCCATTCTCCAGAGCCTTCTAGCAGAAAGAGAAAGGATTATGATTATCAGAATCAATATCCGCCTTCAAAATACCGGGATTCTGATGATTCACGTGCAATGGGAAGGCTAGAGAGAGCTGAGAGGGATCATAAAAGGATCAGGCAAGAAAATCATGTGTGATGGGTTTTTTTTTTTAATCATGATTTTATTTATCTTTTGAGTGTAAAATGACTGGTAATTAAACTATTGGGCTCAGGGAGTTCAGATTGTAAAGTCTGATGCGTTTGTTGAGTTCGGCGTTGTTTGAGGCCCTTTGAGGAACTTGGCAGTGTTGCTGCATCACATCAAGTTCTCTTTCTATGCGCAACATCAGTACGTTGGTGCCTCTGAATTCATGAGCATAAAACTGTATAATCTCCCTCAAGCATTTATATCCAATGAGGGATAAAACTGTAATAAACCATTTATATATTTTTGCTGTTGATCTTTTTTAAATTTATATTAATTTGTAAACTTTC

>UN00745

AAGATTGGTAAGACTATGTAGCTTTTATCTCCAAGGGAGAAGAACTGATCGTAGGATGAGAAAAGGAGACGACCTAGTCCATGTGTTGTACTGTTACTATTATTACTTTCTTTCATGTAACTTCGCTGCGCTCATATGAAGAAAGATGGGGAAAAAAAAATCTTATACGTCTTACCCTGTAATAATTGGTTTGAGAAATATGAAGCTGTTCATAGGCTGCATACTGTTTCATAATTGGCCTGAGTTTGGATTTCATAAATTTAGTTGAGGTTAGAGTCTGATCTTGATGTTCTGTTTCTGGTGCCAAACTTCTGACTGAATGAGAAGGAAAGACCACATTTCCCCCTTCTTTTCCTTTATGTTATTGTCACTGATTTTGTCTTTGTTTATATCATTGACTGGTAGTTGATGCCTGACGATCTTCCTTTAATTTGGGATAGCATAAAACTTTTCTGTAATATTCAACTGGCGACAAATTCAAGCTTAGCTGTACTATGGGGCTAATCCATCATCGTAATCTACCCTACACCACTGATTTTTCTTTTTCTTTATGGGTTAATTTCACAAAACCCCCCTAACTTCGGGGGTGTTGTTATCTCACATAACACCCCCAACACCCCACACACAAAAAAAAAAAAAAAACGAAATCTTCGGGGTGTCTCACGAGACCTAAAAATTGACTTTTCTCATAGGACCTTCCCCAACACCCCCACCCCCAAAAAACCCTCCTCCCATGCGTGCGCACAAAAAAAAA

>UN00746

GTTCTTAAAAAGTTAAAGTAAGTCCCTTAATTCGATCTCCTCGCTGTAATCTCTGTTCTCTAATTTCGCAATGGCAGCCATGATCGCTAGCAGGAGGAGCGCTGCGATCGCGAAGCTTCTCGATAAGATGCTGGTCAATCCATCGCGAGCTCTGCGGGCGTCCTCTCAGCCTGCGGCTCGATCGTTCAACACCAACGCTCAGATGAGGGAGGTTGATGACGGCGAGACGAGCCTCG

>UN00747

CCAATTATTACCAGAAATAAAACAGGTCGGTTTGGTACTTATCATGCCGCGAATGTCTGAAAAGAACATACAAATATACCTCTGCATTGAAGAACAGACGTAACGATTCCCATAGCTGAAGGGATACTTTTGAACCAAATGGATGTAAAATTATCAAATATCAATAACTGAAGAATAAATTATCAGAATAAATTATCTTTCAATCGGAAATGAACTCTCAAATAGCATCGACCACTGAGATCGTCCTTGAAATTGATTAGATCGGCCGCTGCTTTGCAATTGGAACAGCAGCGAGAATGGAAGGGTTATTCTGGCATCAACTTTCAGTTCTTAGCATCTTGGAATGGAGGCAAGCCCCAGTTGCTCGGGTTGAAATCCAATAAGGAAGTGAGGACTTCATCT

>UN00748

GTAGTTAAGTAAATCTGCACGCAATACCAAGACAAAACAGAATACTAGTACATACAATTTCTACCAATGCATTTGTATAAAACACACGACGTACAAGCAGCAATATGAGAAATCATATCTTTCATTTAGCCGGCACTCTTGTACTCTTGCAGCTACTAATATACACAAAGGTTGTTAAATATCCAAATCATCGTCTGGATCCTCATCAGAATCAGGGGCCTCATCATCTGAATCACGAATATAATGTATTTCACCCGTCCCCTTGGTCTCATTGTTTCTCTCTGCAGACAATGCAGAGAGATGCATTGAGTGAGGATCC

>UN00749

AGCAGATTGCTTGTGGAGATAGTCACTGTTTGGCTGTGACTATGAATGGGGAGGTGCGGAGTTGGGGGCGGGAACCAGAATGGGCAGCTTGGTCTTGGGACCACTGAGGATTCTCTTATACCACAAAAGATTCAAGCATTTGAGGGAATTCCTGTGAAAATGATCGCTGCGGGTGCTGAACATACTGCTGCGGTTACAGAAGATGGCGACCTATATGGATGGGGCTGGGGCCAGTATGGTAATTTGGGCCTAGGTGACCGAAATGACCGTCTAATCCCAGAAAAGGTTCTTCAGACAAATGGGCAAAAGATGGTATCCATAGCATGTGGGTGGCGTCATACAATAACTGTTTCCTCCTCTGGTACCTTATACACTTATGGATGGAGCAAATATGGTCAACTAGGGCATGGAGACTTCAAAGATCATCTCATTCCTCATCTGGTGGAAGTGTTAAAAGATAGATGCATATCTCAGATATCGGGTGGTTGGAGGCACACTATGGCTCTCACATACA

>UN00750

TCTTGGATATCATTTATAGGGAGAAAATAGTGATTCCAAAATCAATGTCCTTCATCACTTTCTTGGGCCGTTCCGATAGCCCAGCAGTTATAACAGGTAACGACACTGCAGCAACAATTGGACCCGATGGAAAGCCCATGAATACCTTCCACAGCCCAACTGTGGCCATCAATTCCAACTATTTTGTTGCCGCCAACATCAGATTTGAGAACACAGCTCCATACCCTGACGTCGGGCAGGCCGGAGGGCAGGCGGTGGCGCTGCGGATCTCCGGCGACAAGGCGGCCTTCTACAACTGCAGCTTCTATGGGACCCAGGACACTCTCTATGACCACAAGGGACTCCACTACTTCAAAAACTGTTTCATCCAAGGATCTGTGGACTTTATTTTTGGATATGGGAGGTCTCTTTATGAAAACTGCTATCTGAACTCCATAGCGAAGAGGGTAGCATCACTCACGGCACAGAAGAGAACGATAGCAACGATGGAGAGTGGATTCTCCTTCA

>UN00751

CCTGTCATGCAAGAGCTGGGCTTGATAGGGTTACGCATCCAGAGAATGCCAAGTGAATCTGGTGTAGAGTTTGGCATTCCGTCTAAATACGACTATATGACGGTATGTGCGCCATCATGCCATGATTGTTCGACCATGAGAGCATGGTGGGAAGAAGATGAGGAGAGAAGATGGCGTTTCTATAAGAGTGTGGTTGGATGCAATGACGAACCCCCTTCTCGCTGCATTCCCGAGGTTGCACACTTCATTCTTCAACAACATTTCCACGCTCCATCAATGTGGGCAATTTTCCCACTTCAGGATTTGCTTGCTCTGAAAGAGGAATACACAACTCGACCTGCAGCAGAGGAGACTATCAATGATCCTACAAACCCAAAACACTATTGGCGATTCCGAGTACATGTAACTATAGAGTACACTGTTAAAAGATGAGGATCTAAAAATGACCATAAAAGATCTTGTGGTCGACTAGCGGGAGATCATATCCTGGGATTGACATGCAAAAAGAACGTTTTTAGCAAAACGACT

>UN00752

GGGATTCATCGGCACTCGGTCTGAGCCCATCCAACTCCCAGTCCGCACCAATCAGATCCCAAGAGAAATTCCAGCTCGGAAATATCCTTCTTGGCTTCTCGTTCTTGGAGCTGGAACTCTCCCCTTCGGAACTCTCTTCATCGAACTCTTCTTCATTCTGTCGAGTATTTGGCTGGGGAGGTTCTACTACGTGTTCGGCTTCCTCTTTGTGGTCCTTATGTTGCTCATCATTGTTTGTGCTGAAGTGTCTGTGGTACTGACTTACATGCATCTGTGTGTGGAGGACTGGAGGTGGTGGTGGAAGGCTTTCTTTGCTTCAGGCTCAGTAGCTCTGTACGTGTTCCTTTACTCCATCAATTACTTGGTATTTGATCTCAGGAGCTTGAGTGGGCCTGTATCGGCTGCTCTCTACCTAGGATATTCCCTCATCATGGCGTTCGCTATCATGTTATCCACTGGCACCATCGGGTTCTTGATTTCGTTCTACTTCGTTCACTACCTCTTCTCATCTGTCAAGATCGACTAGAAGATTGTTTTTTCATTCCGCTGCAGTGGCTTCATAGATATCTTTGGCTCACAAATTTCATTCTAAAGTTTCTGGTTGCAGATAAACGACACTGTGGTATGACAAAGTTTAACCTGTCTTATTCTCATGTTCTTCAAGAACATGCTTTTAATATGCCTTTTAAGCTTTATTAGATCTAAGCTGGTAGCACTTTTTGTTTCAGTTACTTTGTTGTCTTTCTTTTTATTGAGACCTCTATCAGGCTCGCGGCAGCTTTACTTCATAGCTTCTTTATTAATGTTTGTAATGGATGATCTATCAGTTGGATTTCACTTTAAATGTTTAAATTTCAGAGATTCTCACGCTACTTATTTGAACACTGTTTTGAAT

>UN00753

GTAAAGATTAAGATCAAATTGTAGCTGTTGGAGCAAATTGACTGTATTTCCAGTTTGATCTTATAACAATTGACAACAATCCTGTAGACGACAACTTGTGGAGCTTTACATTTCTCCAACACAATAGAATAAAAACAAGAGGATAGAGTAGAAGCAATATCACTACAAATCCATACCGTAAGAAACTCCTCAGGAGTCGGCCCAAAATCGCTAATCCTTCGGTGGTAACTTATAGTGATTTAAAAAACCATTAATCTTCAAGCAATGGAATTGTAAGGAAACCCATGAATACGCGGGGAAAAAATGAGACGGTTCAACCCAACTTGAACCCACCCTGGGGAGGCATTTGACCTCCAAATTCAAATACCTGCTGCATATTACCCACACTTTGCATATTCTGCTCTTCATCTTCATCATCAACCCAATACCGCTCAAGAATCTTCACGCTCTTCTCATATATCTCATTGTTGTCGTGCCCTTGCAAGCTCTCGATTTTATA

>UN00754

CTTGGTACTAACGCCCTGGATTTGATCCACACTGGTATATTCTTTGATGGTTGGGTGGATATGCAATTGCTAATTTTTTTTTTCCTATTTGATTAGGATCCGGGCCTCTTTGGATGATCATATATATACATATTTTTTTATTATGATTTTGAATTTAGAGTTTACAACAGATTGGGTCTAAGCATTATATAACAGGCTTTAGTTTTATATTTGCTGATTTTTTTAAAAAATAATAGAAGAAGGATGCGAAGGTAATTTATCCTTAAAAGCCGTCATAGATTTG

>UN00755

TCTCAAATCAATCTCTAGCATAATCAAATATCAAAATATATTATTTTCACTATAAAACATATTTATTATTATTTTTTACATAATATTCAAATTTTACGTGGTTTTACAGTTGTTAGTGGATGTTACTGTTTACCCATGAGCAGTAAACCCATTTTGGGGTATTAGTTTTTTTTTCCTTTTGGTTTTTCTTGACCAAAGATCCTTTTGAAGAGATTGATTGAAAAGGGCATTTGAAAAGAGAAAGAGGGTTTTGATAGTAAGTAGGCTTCAAGCCTAGGGGCCTCACACCCAAGGACTTCACG

>UN00756

TATTGTTTGTTGGTGGAGATGGGTTTCCTAAAAAAGTGTTTTTAATGGGGAGGAGTGCTCGTTGCCCGATAATTTTCCGGCGAGTTGGGGTTTTAGGAATAATGCCGGTGGGTTATTTGGTTTGGTTCTTGTTGTGGTCGCCACTCTGATCTTGATGTCGGAGATGTAGAGGATTGATGCTTGTTGATGCTGATTCTTTTGATTCTTTGCGATGGATTGGATTGATGGGCAAGAGCTAAACTGTTTGTTTGGCAATTTTGGTGGAGGTAGAAGCTGCTGCTTGATTTATGACTGGGTTGTGTGATGAAGAGATGAATTGGGTAATGTTTGTCTTGATCCTCTGTTGTTGATGTTGTTTCTCATACTCATAGAAAGTATGTTAACTTTTTTAGGATAATCTGTTTGCAGATAAGCCTCGAATTACCAGCTTCTTTCATTTACTGTGGATATCATGCGGATGCTAATTTTGCTTGAAATGCTATGATATATTGCCAGATTTATTGACT

>UN00757

TTTCTCAGGCTTATATAACAAGGCAGTTTCTTTTCAACCTTAAACAGGACGGAAAAGCAACTACCCTAGAATAGGTCCAGTTGTCATATCTCCTGATGGACTCTTTCCATCACTTTATAGAGAAGTTATCAGAAGAGCTCCTCATGAATTCAAGATCTCATGTTTGGAGGACATAAAAGCATTATTTCCTCCAGAATGCAATCCGTTTTATGCTGGTTTTGGCAATAGAGATACTGATGAGATTAGCTACCTTAAGGTTGGCGTTCCTAAAGGAAAGATTTTCATTGTTAATCCAAAGGGAGAAGTTGCCGTACATCACCGAGTTGATACAAAATCGTATACCTCACTTCATGCATTAGTGAATGGAATGTTTCCGCCCATGTCTACAGCTGAACAGGAGGACTTCAATTCATGGAATTACTGGAGATTACCTCTTCCTGATATCAATATTTAAGCCTGAGCACATTACAGTCTCACTCGTGAGTTGTCAGTACTGACATAGGAAGCGGGGAAGGAGCTTATTGACATCCATCACTTGTATT

>UN00758

GATCATTTTGATTTTCCTGAACCCTCGACCTATTAATTCCCTTGAATGCTGAGAGCGAGGACAAAATTTTGGGAAATCTCCTCCTCAAAAACCCTAATAAAACCCTACCACACGCACCCAAGAGGAGCTCCCCTCCCTCGCTCCCTCCTCAAGCTCGCGGCCATGGCGTCTGCTCTTCTCTCGGACTGCGCTGCTTCTTCTTGCTCGACACGCCTTCATCCGTTCAAGGGAAAAGGCGGGAACTTTTCTCGCCCCTTTCATTGCCCTGGGTCGGTCTCTTTTCCGTTGAAAACCCTAACATGTCAAGCTCGTGGATTTGGCTCGAAATCCACGGATTGGAGTTTGTCAGCCGGGTCCCAAGTAACAATGACTGCAAACACATTATCTACTGCTAAGGTCATTGATGGAAAATCAGTAGCAAAACAAATCCGAGAAGAAGTTGCTTCTGAAGTTGCAAAGCTGAAGGATACATTAGGTATTACCCCGGGACTAGCAGTCATTCTAGTAGGTTCTAGAAAAGATTCTCAAACATATGTGAGAAACAAGAAGAAGGCTTGTGAAGCTGTGGGTATCAAATCTTATGAAGTTAATTTACCTGAGGACTGCACAGAAGAAGATGTGCTAAAGCATATTTCAGGCTTTAATGATGATCCCTCTGTTCATGGAATCTTGGTTCAGCTTCCCCTTCCTCGGCATATGGATGAGCAAAGTATTCTTAATGCTGTCAGTATCGAAAAAGATGTGGATGGTTTTAATCCACTGAATATTGGTTGCCTTGCCCTTCAAGGCAGAGAACCGTTATTTGTTCCTTGTACTCCCAAAGGATGTATGGAACTGTTGCGCAGGTATGGGATCGAAATTAAAGGAAAAAGAGCAGTTGTAATTGGACGGAGCAATATTGTTGGAATGCCTGCTGCATTGTTGTTGCAGAGAGAGAATGCAACGGTCACTGTGGTACACTCTAGGACCAACAATCCTGAGGAAATCATACGACAGGCTGATATAGTCATCTCAGCTTGTGGAGTTGCACACTTTGTAAGTGGCAGCTGGTTAAAGCCTGGTGTGGCTGTGATAGACGTTGGAATCAATCCAGTTGAGGACCGAGAAAGCCCTCGAGGTTATCGACTGGTCGGTGATGTTTGCTATGATGAAGCATGCAGTGTAGCTTCTGCTATCACTCCAGTGCCTGGTGGAGTTGGTCCCATGACCATAGCCATGCTGTTATCCAACACTCTCGTATCCGCGAAAAGGATACACAACATTAAGTGATGAGAGAGTTTTTCCGACTGTTTTGGTGCTTGTTTGCTCAAACTTGTGATTTAGGGGCTCTATATTTTTCTGCCCTCAAATTTTTTTTGTTATAAGATATGAGGCTGCCCAAAATGTGAAATCAAACAAATATCAGCCATGCTATGAAGGGTTCTTTGTTAACAGGGTTTGGTGCTTTGCTCAAAATGTGAAATAAAACAACAGGATACCACAGCATCAAGCGATGGAGTTGATCAAGGGAAATGCTATGAGACTCCTTAGGACGAAGTAGTTTTTAAGGT

>UN00759

TTATTTGCTACTCGCTCCTCAAACTCCCTACCAAACCAAGCCCATTTAACCTCATTGCTACTCGCTCCTCAAACTCCTTATCAAGCCAAGCCCTCGAATGAATTTTTTTAACTATTTAATGAGAGGAAATTTTTTTTTGACAAATAGTACATTAAAAGAGTTCTAAAGAATTACAAGTGGGTATGTACACAATTAGGTGATATTACGAGGGGTCTATTCGCAACAATAGAGCTAAGGGGGTCTATTTACAATTGGGCATTAGTATAGAGGGTCTATCCGTAATTAACCGTAGAGATATTGATGATTGCTTATTGGGCATTGAGGGGTAGAGCCCTTTCTTAATTGAATGTTATTCTTATTGTATGTGCTCCTGTATTGCGTATGGATCCTATGTAATTCGAGATCTTGCCTATTTTTATTTGAACGAAGAGTATAAACGT

>UN00760

GCTACATTCCAGCCACAATGTCTTTCCTCAACATTCTCCCTGCTCCAAGATCTTTCCCTTTTATAGTCCCATATCTCCTCTTTGAAAACACAATGTCTGTTACAAAATTCAACGCAATGATTTCTGGTTTATTTCAACTCGGCAGTGCCTATGAATGGGTCGTCACCAAGAAATCCGGTCGTTCCTCTGAGGGCGATCTCCTCTCATTGATCGAGAAAGAACCTCTCAAGAATTATCGAGAAAATTCGTTGCCCAATCTTGATGCCATCGTAAAGGAGAAAGAGGATGTACAGAAAGAGAAAAAATTGATTAACGAGAAGAAGAAGCATAATAGGATATATAGGAAAGAGCTAGCACTTGCCTTTCTTCTATTGACTGCCTCGGCAAGGAGTTTGCTGTCTGCTCAGGGTATACATTTCTATTTCTTGCTCTTTCAAGGGGTTTCTTTCTTGTTGGTGGGTCTTGATTTGATCGGCGAGCAGATTGAGTGAAATTGAGGGGAAATGCATATATTGATGAATCCCACGGGGAGATGCTGAAATCATTTTAAGGCTAGAAGTTGGTAACTTTGGGAGGTGTAGATAGAGGTTTTCTCTGCCTGTTGGATATAAATGCTTCATTCTTTCAGGTTTACCT

>UN00761

TAACCAATCATGTTTGATGATCTTAAAAGGAATTTCGTAATGAATCCACAAAATGGGCTCACGATTAGACCTTTCAGGAAAGCCCATTTGAATCGTAACAGTGATCAGGAGCTGGTCAAACTCACTCGGTATTTGCTAGCTATTGCTGAGCTCCCTGATCTGAGCTCACTTGATCACAGGATCTGGGAACGCTTTATAGAAGATAGCACTAAGAGACGCAGACACAGTTAAAAACTGTGTGGTTATAGGATATCTCAGCGCTTATGCATCAAACGCTGATAAATTATTGATCACTCATCACAGGTTACTTCCTCTATTTAAATTCTCTAGTCCTGGATTTATATTTCCGTCTATGTATCTCCTTGGATCAAGATATTGTTTTTCCTACTCTGAGATACACCTCTCTTCCTTTGAATTATTATGTAGTACTTTTTAACCTTTTTTTACCCAAGGCAAGTTATATTGTTTGCATATAGTCTGCATTCTTTGTATCTTTTTTGCTATGAGTGAGAGTTTTTTTGGATATAAGTAGAATTTTAATATAGATAATGGAAGTTCTTTAAACGG

>UN00762

GTATACAGGAAAAAATTATTCTTTGCTAACGTCAGATACAACTTCAGGGCTACACATATAAACATAAGCAAATGACATATAAAATTTGATATATACATTGTATTTCCCTGAGGTCTAAAGAGGCATTAAAATGACATGAACTCCTAAACCACAAGTACAACTGTTTTTTATCAATGAAAAAAATGAAACATCATGTGTATTGTTAATTTTCACCCAAATATAGTATTATGAGCAGAAGAAATACACATGGTGTACAGGACAGATGTAGAATAGAATGATTACCAATCTAGAGATGCA

>UN00763

GTAAGCAAAATAATCATCTTATGTAGAATTTTTAGAATGCAAGTTCCCAAATATTGGACAACCCAAGAATAAGTTCTTTACAAATGTTCCGATCTTTTACATTACCCAGCTCTTCCTAATCAGTAAGTAGATGACTAGCTCACAGAATCACAATGGCGAACTTTCCGTAAAAGGTCTTTTTGAGTATATAATAGATGCTACTGAACAGTAAATAATCATAACAAAATTGGATGTACATTCAAGTGTAGAGCTAAAACAAGCACTAGTGATACCATTTAGGTCTCAATTCTTCCT

>UN00764

TGATGAACGTAGAAATTCCACAAAGCTAGAATTATATGCATATATTCAGTCACCATTGATAAAATAATAATCAAATTTTTGGGAGAGCAGCTGCCACAATAACAACAGTAACAACATTGTTACTGTTAAAACATGTACATTTAAAATAGATCGGGGGCGTTGAAAAACAAAAGAAAAAAGAAACTGGAAAAAAACTAAACACAAAAAGAAAACTAAAAAAGGAGAAGAAGACAAAACAAAAACAAAAGAAAACTGGAAAACAAAAAGAACACTAAAAAAACTGGAAAAAAGTGGGAAGAAGGAGAAGAAGATAGAGAAAAAAAGAGGAGAAGAAGACAACGAAATGAAGAACAGAGCAACAGTAACAACATTTTAACACTAAATTTCCGTGAGAACAGTAACAACATGTTAAAATAACGTACAGTAAAAAGAAAAGAAAATATTTGAGAAATATCGATGAGGATACGCAAAGAAGGGTTC

>UN00765

TTGCGGTGAGCACACCAAGCTCTGGAGGAAGCAACAATTGACGGGGAAGTTTAAGATTGTGGCGAGGGAGGACTCCAAGAAGAGAAAGGGCCGGAGTTGAGATTGAGAAATGGTGGATAGATGAAAGTTTATGAAATTTTTTTTTTGTCATTAGAATTGTATTATGTTAATTTTAAATTAGGTTTGTGGAATGTGTAGTTGAAGTAATTTATTTTCTCGCTTATTAGTTTGATATGAAGTAAATTGGGTGATGGTCTTGATAGG

>UN00766

CGCCATGGGCTGTCGCGCCACCTTGTGTTGCCTGATGACGTGTAAATTGTTCTCCCATATTGTTTTGAGTGGTATTTGGATGCCTGCAAGTATTTTTCGATGCCTACACACACAGGATGCTATTAACTGCGTTCACAGTGCAAATAAAAGAATTAGCTCCATGTTCCTTTTGTCTTTGACATCAAATGATAATAACACAACAAGAGTTATCAAGATATAACAGGAAAGATAATGCTTTTCATGTAAAAGAGTTAAAACATGAACACCAGATGCACACAAGTGGAAAAATAATGCAGTTTAGAAGAACCAGCTTCATTTGAGTTTTGCCCTTTGCAGAAATATTTACAACCACAAGCCTTTCTCTATATTTCTTTGCAAG

>UN00767

TTCCAACAACTGGAAACAGCAAAGGTAGAATACATTCGTGCAACCGTCGGTATAAAAAACGAAGACAAAATCCTCCTGCCAAAAATCGTAGACTACTTATTCGAAGGACATAAACCTGAGTTCTCAAGGAATCCCGGACATGATCAAGCGCCATCTCCCTGAGACTCTGAAAATGGCCATAAAGAGATGTCAACAAGGGAAATCTCACAAAATTATTGAGTGGGTTCCCTATAATTTCACATTCCGATATTTGCTCTCAAGAGATTTGGCAAGTCCTCAGATACTATGATGATATAGACGATCATAACTGTTTCCAATAGATTGGGGGGAGTAGGAGATAAGTTACCGATTATTCTTTTGGTTTTTAACTTTTGTTTTGTACAAAATGAGCTGGTAATTAGCATATAAAGGCTGACTACTTCACTGTATTTTATGTAGATCATCCGAGTGGTTTGCTAATTTTTGGTGTAATTATGGTGTGAATAATCGAATCTTGTTCAATTGCTTAATAATAGAAATAAGTTAAAAATTTTAAAAAACCGGG

>UN00768

GCTTTGCTCAATTGAAAGTAGGTTTGAACAATTTAAAACGTATGAAAATTTTTTGGTTTTTTGTTTGATTTGAAAAAATTAAAAGCTTTAGATGATGTTGAATTGAAAGAATATTGTCTTAATCTCGAAAATGCACTAAAATATGATGAACATTTAGATATTAATGGACTTGATTTGTTTTCAGAATTGAGAGTGCTTAGGAAAAATTTAAAAGTAGAGATTAATAGTCCAATTGAAGTGTTGAACTATATAAAGTGGTTAGATTCTTTTCCAAATACTTGTATTGCTTATCGTGTACTATTGACTATACCCGTAACAGTTGCTTCCGCTGAAAGAAGTTTTTCAAAATTAAAATTACTAAAATCTTATTTAAGATCGACCATGTTACAAGATAGAATGAATGATTTAGCAATATTATCTATTGAAAATAATATGTTAGAAAATATTGAATATAAAACTTTAATTAATTTTGCAGCTCAAATGGTAAGAAGAATGAGATAAAATCTATTATGAGTTGTATCATTTAGGTCTCATCTAATATTTCGCTTTGGGCCCCAAAATTCCTAGGTACGGCCCTGAGTTCATCGCCGCCTTCGTCGAAGTTCACCGCCGCCTCCGTCGGATCTCGCCGCCTCTCCTCATCCTCCTCCCGCTCAACGAGGAGGATCATGAGATCGAG

>UN00769

CTAAAATGTTGGTTCTCAATGGAATTGTACAGATAATATTAAGTTCTTGTGATAAATTAGAGAATCCGTAGCTTCTTAGTTGTAACTCAAGACAGCAGTCACACTAATGATAACTCCCCGGCAGGAATAGATCCTTTGACTAACTTAGCCCATGCTTCATTGGTCTCCATGATGACCTTCAGTGCATACTCCTTGTCAGCTGCCTTGTTGCCAAGACCAAATTTGTTGGCAGGCTTTCCATCTGGAATCTTGTAATCTCTAAACCAGTCCCTAATTGCAGTAAGAGTGCCCGGAAAATGCTTCTCAACATCATTCACATCATTTACGAGGGAAGCCTTAGGGTCATCCAATGAAATTGCAACAATCTTCCAGTCTAGCTCCCCTTCATCAATCATGGCCAGAGCTCCCAAAGGCTTGACCTTGAGAACATCACCAATCTTCGCTCGAGCTCCACCTATCTCAACAACATCAACCGGATCATTGTCACCAAATGCTCCATCAACTTCAGCATTTGCAAAAGATGGGTCTTCCCATGTTTGTGGAAGCAATCCATAATTCCAATTTATGTTGTACGGATAGTATCTCAGCTTCCCCTTCTTCGTATCCTGCTTAATTGGTGTATGAGCCTCATCCGTCGCCACCTCCATCTTCGCACTACTCTCCTTTGGGATCTCCACCACAAAATT

>UN00770

AAACTAGCACAAAGCGACCACTGGATTAAAAATATTACCAAAATTTATGGTGTTGAAAATGATTTAGAATACATCAATCCATCTGTAAACAGATGCCAATCTCAGACAGATTAGTTCAAATGACTTACTGATTGCATAAAACCAAATGACCACATAATATAATGGACAGAACTAGACATGGAGTTAGAAAGAGCCTCTACCATCCCTCTATATACTTTGATGAACTCAACGAGAAGCTGAATGAGCCCTCCTTTATTTCATCTGGCAGCTTGAGTGGAGAAACAAGGAGTGAGATATGCAGATACTTCGACCAACCTCAGGGAGGACAATGATCTATGTGCAGATGAAAGAAATATCACAGTATCTTCAGTTTTAGAAGACTTTACTACCAACCTTAGGAACCTTAAGAAATAACTTAACTAA

>UN00771

ACTAGAAAACAATGCTAATATATTAGAAGGAGATGGGCAAAGCAAATTAAGGGTTTAACTTCATAAAATTTCCTCCGAAATACTTCACAAAGTGAAATCAGCGGCGAAGTAAAAGAAAGGAAATGCCAATCCAATAATCACAAATATAAAGGATAAAACCTCCGCCATGGTATCAGATGGTGATCAGAATAATTTATTGGTGGAGATTGACAGAAACATTAACTGATGAGGGCACCAGCACCAGGGGGAAGATGATTTAGAGGGGTCCCGGGAAATTTCCGTTCTCCCTCTGCTCGTTCCATCTCTCCACCCATTCCCCTGGACAGAGGGATCGATAGTACTTGGCAAATTTTTGACAGTCCTCAGACTCTCCTTTGGCTGACAAACAACTTGTGAAACTCAATGTAGCGAGTGAAACAGTGTCTTGTCTGATTGGTAGTAGGGAAGCGGAAATCAGCTGGTGCAGTTCTTAATTCAATCCCCGCCATTATTGCAGAACTCTGATCGAAGAAAACGATAAAAAAAGCTTAGAAATAAAATAAAATCAGCCTTCCGCGATCCCTT

>UN00772

TTTAATTTTGGTTTATTTGTTTTCCTTAGTTCGGGTAAGTTTTCCCTAATTTCAACTTTGTTGGTCGTTTTATTAGGGCCACTGGGGAATTCCTTGAGAAGAGTTGAAGCTACAACTCTGTGCAGAGTTTACATACGAGGTCGTGGCTCAGTAAAAAGATTCCGTAAAGGAGGAAAAGCTAAGAGATAAACCTTGGATATGAACACTTAAGTGAACCATTGCATGTTCTTTTGGAGGCTGAGCTTCCAGCGGACATCATTGATACACGGTTAAGACAAGCTATGAATATCTTAGAAGATCTTTTGAAGCCAGTGGACGAGTCGATGGACTATTACAAGAAAAATCAGTTGAGAGAGCTTGCGATACTGAATGGCACATTGCGAGAAGAAAGCCCTCAGATGAGCCCCAGTGTGTCCCCATTCAATTCCACCAGTATGAAACGAGCAAAAACAGGAATATAGTTCCAACATCTTATTTCTGACCCTCAGAGATTTACTGCTTTTGTCGTATGGAAATATTCGGTATAACAGTTACTCTTGATATATGTGTGACAAAGCTCCATATTTATTTTTCTGAATTACAACAAACTACAATAAAAGAAATTTTAAAATAAAAATAAACGGTAAAAAATTTAAAACTTTT

>UN00773

TTGATAGAAGCTGGAATTTGGGGTTTCTGGAAGGGTGTGATCCCCACACTTATAATGGTTAGCAATCCTTCTATCCAATTCATGTTGTACGAAACTCTCTTAAAGAAACTGAAGAAAAGACGTTCCTCAAATGCGAAGGGAGCTGATGGATTTACTGCTTTTGAGATTTTTCTTCTCGGAGCTGTTGCGAAACTAGGAGCTACTCTTGTGACATATCCACTTCTTGTTGTCAAGGCTAGGCTTCAGGCAAAACAAGGAGTTGATGTCGATAAGAGGCACCAATATAAAGGAACGTGTGATGCAATCGCGAAGATGATTCGATATGAAGGCTTCCCCGGATTTTACAAAGGAATGGGCACAAAGATAGTGCAGAGCGTCTTTGCAGCGGCAGTTTTGTTCATGATTAAAGAGGAGCTTGTGAAGGCGACTCGGTTG

>UN00774

ATTTTCTTAAAGTTTCCTTAATTACCTACGTAGTACTCCAACACATTCAAAATCCACTAATAGAAGAAGTCTACAAAGCACCACCAAGATCAACAAAGAAAAGGCAAAGGTAGTATATGTATCCAGTACAGAGAAACTAGGAAGACAACTCCAAATAAGGGCACAAACCATAACACGACAAAAACCCGAAATCAGGACATAAAACATGCAAGGGAACACCCGTTTTCCTTCACATAGTCTTGCTTCATCAAAGTCCAACACTACGAGCTCCAACAAGATTCAAAATCCACCACACCTCTAAACCATATGAATAATCACGAACTCAGCGTGAAACTAAGATTGTCCATATCCACCCATCTGCCCAACCGGCGGCCGCACCATTGCCCCGCCCTGATAAGAACCTGCAGCAGCACCAGCACCAGCTTGTTGAGCTCCCATCTGAGTAGCATTCTGAAACCCAGGAGGGCCTTGAAACCCTGGTGGTCCCTGAATCCCCTGCTGACCCTGAACCCTCCTACACTCTGTCTTGGTTACCCTAACTACTTACCCTTAATTT

>UN00775

TTGTTACGTTGTATTTTGTTTCTACATTTATATTGCTTTCTGAAGTATCTGTTTCCTAAACTGAGCCCAGGAGGATCTAGAGACGGATGGATCCGTTTAATTACGGCGATGTTGCTGTTGGGAGTTTTTCAGGATGGGTTGCTTTTGTTGTTCCACACCCAAGTTTTATGTATTAACAGGAAACCGAGGTTCTTGATTTATTATTTTTCCTGTGGGATTTTGACTTGAAGATTTCAATTTTTAATCCACACCATGTAACCTCTTTGAACTCCTTTAAGTATGCGCGGAGCTTTGTATAGAGAGTATTAAGCCCCCGAGTTGGGTTATCTCTAAAATCTATGTTTTCGTGTCGTTAGTTAACTAAAC

>UN00776

GTCTATTGGGGAGGCAGTTGCTTATGTTCTATCTGGGATGTATGGCAGTGTTAGCCAACTTGGTGCTGGCAATGCTATTCTTATAATCCTCCAACTTTGCTTTGCGGGTATCATTGTCATATGCTTAGATGAGTTGTTGCAGAAAGGATATGGACTTGGTTCTGGCATTTCTCTCTTCATCGCCACCAACATCTGTGAAAACATCATCTGGAAGGCTTTTAGCCCTACTACTATTAACAGCGGCCGAGGTGCTGAATTTGAAGGTGCTGTCATTGCCTTGTTCCACTTGTTGATAACTCGAACAGACAAAGTCAGAGCTCTCCGTGAAGCCTTTTACCGTCAGAATCTTCCGAATGTGACGAATCTGCTTGCTACAGTCTTGGTTTTTCTCATTGTCATTTACTTTCAAGGTTTCCGGGTTGTTCTTCCAGTTAGATCGAAAATGCTACGTGGACAGCAGGGTTCCTATCCAATCAAGCTATTTACACCTCTAATATGCCCATAACTTACAGTCTGCCCTT

>UN00777

CAAAAAAGTCTGTCAAATCTGCAGAGGATATATGCAGAGCAAAGGAGAAAGAGATAGGTGGGAAATGAGCTGCAGTTGGGACTTGTTTCTAATGTGATGTGTATTGTAGAATGCATCATTTACAGCTATATTATCGGATCGGCCTGAGTTAGACAGTTTCCAGGTTGGTTCACTTATGAGCAGAACCTGGTAAATGATTCATGTTGTGCTGGAATAAATAGCTTTATCCTAGTTTTTTGATACGAATCATTGAGTACTGCGAATTCG

>UN00778

TACTAAACACCCTACCATTTTTGGTTGGACTCTTGACTACTGGAATTCTGGCATTTTCTGGAACGTGTTACACAGTGGCATATCTTGAAGACAGAAAATACTCTTCTCTAGCACCCTTTGGTGGCTTTGCATTTATTGCTGCTTGGGGAAGCTTACTCTTCTGAGGAGCAGATACAGTAAGATCCTTATATGTGTCATGTGTCGTACAATGGGGTTCATTATTTTTAGCAAAGTGCAATGCTACCGATCATGATGTTTGAAATTCTAGATTGGTCAACAATATTTGTTTTAAGCCTCATGTAATTTGACATTGTGGAAATGCTTGTGAAGCTATTCTAATTGGAATTGTATTTG

>UN00779

CCCTCTTACACAAGATCGGCCTCTCCTCCGGCGTCAATGACCCTAGCCCTAGCCCTAGCTCCGCCGACCCCGCCACGGTCGTCCTCGACTCCAGCGAGTCCCCTCCGGCGGCGGCGGAGGCGGCTTCTAAGAGGAACAGGACGGCCGAGCTGGCGGCGTCGAGCGGTGGGGCCCTTGCTCTCGCCTTGCTCTGCAACAAGGCGTTGTTTCCGGTTAGGGTTCCGATCACTATTGCACTCACGCCGCCGATCGCGAGATTCCTCAGGAGGAGGAATCTGATCAAGGGTTTTTGAAGTTTTTGGTTGCTAACGAGTGAGCCTCCCTCTTTATCTTTGCTATTGAAACTGCTTGAATGGGAGGGGGGATTTGTTGTCTACATCTCTGGGATGTTTACTCCGAATACTAGATTTATGAAAAGTTTGAATTGAATAAACGTTTTAATGTTGGGAAAATGGGAGAAAATGTTGTAGCTTGATATTGTTTATTGCTTCAGCTCCCATGATTTCCTCTGGGTCGTTGCATATTACACTTGTATTCCACTTTGTTTCTTTTGAGAAGAAGTT

>UN00780

GTTTCAGATTATGGGTTACCTGAAAACAAGTTTCTTTTTGCATGCTTCAACCAGCTTTACAAGATGGATCCTGATATATTTAACACTTGGTGCAATATTCTGAAGCGTGTCCCTAATAGTGCTCTTTGGCTGCTGCGATTTCCTGCAGCTGGTGAGAGGAGACTGCGTGATCATGCTAGGAGACAAGGGGTGAGGGATGATCAAATCGTATTCACAGACGTCGCCATGAAGAATGAACATATCAGGCGTGGTGCTTTGGCTGATCTTTTTCTTGATACGCCTCTGTGCAACGCCCACACTACGGGTACAGATATATTGTGGGCTGGTCTGCCAATGATAACGCTTCCATTGGAGAAAATGGCTACAAGAGTGGCTGGTTCTTTATGTCTCGCCACTGGTGTTGGAGAGGAGATGATCGTTGG

>UN00781

TTTTTTTTACCTTCACAAAAATCGAAACATTTTCGGACCATCTACTCATAATTGCGCACAAGATCGAGAGCGAACTCTACAGATAAATCAACAACGCACCAAGAAATTATTCAAGAAAAAATACCGACGTAACATACGAAACGTACAGTAATCAAACAAAAATCAAACATTTCATCAGATACCTTACACGCGTTCCTTCCTTCAATCCGATTCCTGCAACCCCGCGAGCACAATCGACCCGACGAACAAT

>UN00782

CTTCTGAGGAATACAATGGCAAACATGACTCTAAATATGAAGAGGTGTCAACTTTCCAACAACTCGATGCATGGAATGCATATATTGTTGCCGTAGCCAACTTGTAGTATTTGGCATAGTTTCGCAATTCCCTCCAGAAGTATTATTCAACTATTTGACATAGTTTTGCTATTCCCCTCATATAGATTATTGTACCCTTTATTTTGCTTATAAACGAAAGCTGTCGTTTAAAGCATTTTGTCCTTCCAGTCATTTGAATTACTAAGTAGTTTGACCCTGCTGAATCTTATACAAGGAAATTATCAAAAGGAGCCACGGCGTGTGTACTTGCCTGATATTACTTGTATCCTTTATTATTTTCTGTAAAGCAAATGTATAAATACATAAGCTCCTTTTCCAGTTCATTCTATCACAATTAGCAGGCTTTATGGGAGCTGG

>UN00783

GTTTCTTTGTTGTATTCTAACATGGCTCTCAGGCGAGATTTCTATTGGTGAAGTTGAACCCATCGGCTAACTACAACTCAGCGCATGAGATGTCTCCAGGTGCAGATGTAATCTTCACAGATGATGTGAGCCTTCAAGTATTCTGCGAGCATCTCCGTAGGCTGGCCGTGCAATCGTGAGGAACGCATCAAAACTGTTAAAAATCTTCTTTTCTCCTTCTAAGTTTGTTTCTCCTTTTCTTTTACCTCAGCTTAGGTCGGAATGTTGATATTTTTAGGAGTTCTTAGAGTCTGTGTTGCCTCTCCTCGCTCTCCCCTTCACATATTTCTTGTACCAAACCAGTCAGTTCTATGACAAATAAGATTATACGATGTTAAGTTCTTTTTTGGATATCCTCAAATAGGCATTAGATCCATGTTATTTGTAATTTTTTAGTTTTAAATTACCTTCCTTAGTACTTGGGTTAATTTTAGTTAAACGTT

>UN00784

GATGTTTTTAATGAAGTTTGGTATTGTGTCCTGCTGAGCTGTGGGCATACCTTAAAAGCAATTGAAGAGATTGAGAAAGCACTTAAAACGAGAGAGGGGGCTCCTTTCTTATGGCGTTTGTTGGAAAGGGCATACGCTTTGGAGGGTGTTGAGAAGGCAAATGTTGTGGCTGAGAAGGCAAGGACTCTAGAGACGGCTTATTTCAAATAAATTGATGTAAAGCAAACAAACTATGTAAAAATATATTGAACTAGCTTGCTTCTTGAGCATCAGTATGTTTATATAAGTAATAAACGGGAGAGCGTTGAAAGCTTGTGCTGCTTTTGCCCATAC

>UN00785

AGCCTGCAAAGATTTGTCTTTAACAATGTCTGAGGTAACTTTGCATATTATTTGGAAGTGCTGTAGCATGACTTGTGTCTACGGTCAAAATGGGACTTCATCTAACATAGTGGTCAGGAGATTGTTTATACTTATCGCCTAATTGATAAAAAAAATTTACCAGGCGGAAAAGTCCTTCTCCTTCACCAAGATGGCGTAAAAGCCATTCTCCAACTCCTAGACGAAGAAAAAGTCGTTCACCATCTCCAAGGCGGTACAAAAGACAAAGGAGTAGGAGCACCACAAAGTCACCTGTCAGAAAGTCTCGGAGCCCAAGTCTTGGATCAGTAGAACGCAAAAGTGCTGCTGAGAAATTACGGAAAGAAGAAGAAGAAAAGAAAAGGCGTCAAAAAGAAGCAGAGTTGAAACTATTAGAAGAGGAAACTGCTAGAAGAATTGAAGAAGCAATTAGGAAAAAAGTAGAGGAGAGTTTGAACTCTGAGGAGATCAAGCTAGAAATACAACGTCGGATAGAAGAGGGACGAAAGAAGTTAGTTCATGAGGTTGCCATTCAACTTGAGAAGGAAAAGGAGTCAGCACTTGCTGAAGCAAGGCAGAAAGACGG

>UN00786

GGCTTTCCATGAAGTTTATGATGTCGTGCTCTACCTTGGGATCATTGATATATTGCAGGCGTATGACATGAACAAGAAAATAGAGCATGCATACAAATCTATTCAGTTTGATTCTCTCTCCATCTCAGTAGTGGATCCGGAGTTTTACTCCAAACGCTTCTTGGATTTTATCCAAACCGTGTTTCCTGAAAATCCTTAGGAAGTTCACACCCTCATCAGTAGTCTCATGAGAATCAACTTCTTGATAATGGATTGTGACTTCAGATATCAGCTTCTGAAGATTTTGTTGTATAAAAAGTGATTCTTGGCCCCACCAAAAAAGAACGAAAGAATTATAGTCTCAAACTTGAATCCAAAGTGTTGTATTTTTCTTTCTTTTTTTTCCCCCTGTATTCCCCCCCATTTGATGTTTCTATGAACACTAATCTGTAATATCAGTTGAGACGATTACTGCTGGAGCCAATAAACATTGTACACTGGAATAATTTGAATAGAATCTTAATGGCGTAGTGAA

>UN00787

TACTTGGGTTCAAGTCCGGTCAAGTCACTTGTCGATGCATGGACTTGTATCACTGACTGAACTCTTGCCAACTGTGTCGCCAAGAGCATTCTCAGCGCGCAAACAACGCTTTAGCCGAACTTGAATCCGCCAGAAGGAACAGAAACCTGCTCGCCAAACTGAAATCCAGTTTGAGCACCAGCATCGCCTGTTGGCATAGCCTCATCATCTTCCTCCAACCAATAAGTTTCAAGAATCTTCACAGCTTTCTCATAGATTTCAGTATTGTCATGGCTCTGGAGATTCTCAATTTTTTTCAAACCTTCAGCTTCATCAATCATCTGAGCAT

>UN00788

CAGTCGAATATGGGTGCGAAAAACCATGCAATCATCATGCCTGATGCAAGCGCTGAAGCTACTCTAAATGCTCTAGTTGCTGCTGGTTTTGGTGCTGCAGGACAAAGGTGTATGGCAATTAGCACAGCTGTTTTTGTTGGTGGTTCAAAACCATGGGAGGAAGAATTAACTAAACGTGCCAGTGCCCTGAAAGTAAATGCTGGTGTTGAGCCTGGGACAGACCTTGGTCCAGTTATTAGCAAACAGGCAAAAGATCACATTTGCAGTTTGATACAAAGTGGCATTGAAAGTGGTGCTAGGATTGTGCTTGATGGGAGAAACATTGTGGTTCCCGGTTACGAGGATGGGAATTTTGTTGGTCCGACCATTTTATCTGATGCGACGGGTGACATGGAGTGTTACAAGGAGGAAATTTTTGGTCCAGTTCTCCTCTGCATACAGGCTGAAAGTCTAGAAGAGGCTATCCAGATTGTTAACAACAATAGGTATGGCAACGGAGCATCTTATATTTACAACGTCAGGCATATCCGCCAGGAAATTCCAAACAGAGATAGAAGCTGGCCAGGTTGGGATTAATGTGCCAATTCCAGTTCCATTGCCATTCTTCTCATTCACTGGTTCGAAGGCATCTTTCGCTGGTGACCTCAACTTTTACGGCAAAGCTGGCGTACAATTTTACACCCAGATTAAGACGGTAACACAACAATGGAAAGACTTATCAAGTCAGGGTGTTTCCCTAGCAATGCCCACATCACAGAAGTCGTAGATTCCCAAAATTATTTTACAAAATGTTCATGCA

>UN00789

GGCTCCCCCTCATTATTAGCGATTCAAAAATTGGACCCTAATCCTAACACTTACCCTAAAAAAAACCCTAATTGAGTGCAGAAATTGAATGGAAGGGGGAGCAGGGGCCTATAATCCGAGGACGGCGGAGGAAGTGTTTAGGGATTTTAGGGGGCGGCGAGCTGGAATGATCAAAGCTCTCACTACCGACGTGGAGAAGTTCTATCAGCTCTGTGATCCCGACAAAGAGAACTTATGTTTGTATGGGCTTCCTAATGAGACTTGGGAAGTTAACCTGCCAGCGGAGGAAGTTCCTCCTGAACTTCCTGAGCCCGCATTGGGAATTAATTTTGCTAGAGACGGGATGGATGAAAAGGATTGGCTGTCACTAGTTGCAGTACACAGTGATGCATGGCTATTGGCTGTTGCCTTCTATTTTGGTGCACGTTTTGGCTTTGACAAGGATGCCAGGAGGCGGCTCTTTCAAATGATTAATGGCCTTCCTACCATATTTGAAGTTGTGACTGGAACTGCCAAGAAGCAAACAAAAGAGAGGACACCCAACAGCAGCAAGAGCAACAAGTCAAATTCAAAGCCATCACGTCAGTCTGAGATAAAAACCTCCAAAATGGCTCCTCCAAAAGAGGATGATGATAGCGAAGCAGGAGGAGAAGAGGAAGAAGAAGATGAGGATGAGCACGGGAACACTTTATGTGGTGCTTGTGGTGAGAATTATGCAAATGATGAGTTCTGGATATGCTGTGATATCTGCGAGAGATGGTTCCATGGAAAATGTGTGAGGATTACTCCAGCTAGAGCTGAGCACATTAAGCAGTACAAATGCCCAGCTTGCAGCAACAAGAGAGCCCGTGCTTGAACTTTTTTGGAATTATGGATTCAGTTTTAGACAGTGTTCTGATGTTGTGCATGTCAGTTTGTTATT

>UN00790

TATCTTATTTTTTCTTAAATAAGAATAAAGTAACCATTATACATACACAGTACAAACCAAAATCTTTATGTGTCGACACAAATTACAGGATACAACTGATATCATACAAAATGTAACAAAATGTAGGAGTGCTTTCACTGAGGAACCGGACTGAGAGGAAGAGCCCTTTCATTCAGTTAGCAGCTGGATGGGCACATCCACTTTGGAAACCAGCTTCCTTAGCGAATAAATAGCCAACCATCATCAAGGAACAAGTTTCCAACCCTGACGCATGACATCGTCATTTATATATGGTCCGAAAAA

>UN00791

AAAAACCCTTCCTTGTCGTCTTTTCTTACTTACCATAACAGGTGCCAAATTAATCTATAGCAAGCTACAATGAAAACGTTTCACCACCAATTACAACAATGAGGGGGGAAAAAACCCCAAAAGACGATAACAAGCAACGAATCTTTTACAGCTGAAACTAAAACAAAGATCCTTCAATTTTCTCATCAAGAGTTCTCGCAGACAGCAGCGATCCTCAGCTGCTGCGTCGGCGGTGGCGGCCGCTCCTGCTCTCCATTCCTCCTCCAAAACGCCCTCCTCCACCAAACCTCAAATCCCCTTTGAATATTTGGACAGTCGTCTCCGCAGTTCAGGACCGA

>UN00792

GGTTTACTTAAAATTTACGTTAAAGTAAAGAAAGATTTCAAGTTTAGTTTATATATCGAGCCGAGATGGCGGCATGGAAGTTTCCAACTGTTGATGTACCTTGTACTCTTGAGTACGTAACTTGTAAATCACTTAGCGCTCTCAGTAAACCTAGTTTTGCGTATGAAGATTGAGAAGTTGCTACGATTGTGGTGCTCGAGGAAACTGACATGTTACCTTATTTTATGGCTTTACTTCCGTAGGCAGATCATGCCATTTTATGTTTTAGATGTCAAATTGATGTTCATCAATCATAGAATGTGAATCAAGCAATTCTACTTGTATGGTAGTAATTGCCCTAAGAGCATGTATGGCTACTATTTATATCTTTGAACCCTTATAAGTTAAGTTACTTATTCTCAAAACTCAATTGTAAGGGTGT

>UN00793

GGTTTATTCCCCGCATATAATGAAGAGCTTAGGCTTCCTGAAGCTCTGGAAGAGGCATTGAACTATCTCCAGGAACGCTCAGCTGCTGACCAGACCTTTACTTATGAGGTGTTGATAGTTGATGATGGCAGTAAGGATAGAACATCAAAAGTAGCATTCAACTTTGTTAGGAAGTACAAGATTGATAATGTAAGAGTCATCCTACTGGAAAGAAATCATGGAAAAGGAGAAGCTATAAGAAAGCATTGTTAAACAGGGAATGCTTCATTCGCGTGGTGAACTCCTTTTGATGCTTGATGCTGATGGGGCAACTAAGGTGACTGACCTAGAAAAGCTTGAAAGCCAGATACATGCGCTAGCACAGAAGAAAGAGAAACTTAGTTCACAGGCTACAACACCAATTGACTCAAGACATAAGCTATCTGATATGGAAATTGCTGTATTTGGTTCCCGTGCTCATCTTGAAAAAGAGGCTCTTGCAACGCGGAAGTGGTACCGTAATTTTCTTATGAAGGGTTTCCATCTTGTTGTTCTAATGGCTGCTGGTCCAGGAATTCGTGATACACAGTGTGGCTTTAAGATGTTTACGAGGGCTGCTGCCTGCAAGCTTTTCACAAACATCAGATTGAAGAGGTGGTGTTTTGATGTTGAGATTGTCTATTTATGCAAACATCTAAAAATCCCAATGATTGAGGTTTCTGTGAACTGGTCTGAGATACCTGGTTCAAAAGTGAGGCTAACTAGCATTGTGCACATGCTCTTTGAGCTTGTTCTCATTCGTTTGGGCTATGGGCTTGGCATTTGGAAAATTTATACTTAATTGACATTTATCAGAGATCAGAGAGAAGTTTCGATCCTTACTTTAAGAAACAGCAATTGCATACACAGGAACAAGAACAAGGTGCTCAAGATATATCGGGAGGCAAAAGGTTATTCGTTCAGTCCTCTTTGTAAAAGTAAATTCCTCCCCTTTTAGTATGCATTGGCCACGAATGAGGGGATCTTTTTCGGGATGTCTAAGTTATCGATATTATGAAAGGGAATTATAAACTTTGTTTTTCTGATTATACATTGTATTGGGTGATGGATGTACTGAGACAACGCCTGATCCAGAAAAGGTACATTGAGTTAGATTTTAGAAATCATACTGTTTACATTGTACTGTTATCAGAAACACTTGTAGTTTGGAGCTTCTCTTTGGAATGCATGATG

>UN00794

CGCCCTTTGAACTTGTAGACCTCTCGAATTGTCACGGCCTTAATAAGATGACAGCGGGGGCGATTTGCAATTAAATAGAGATCAAACCCAATGCTTGCCTCCGGGGGACTGCGCCGCTTCTCGTCGATGGAGAAATCAAAATCTTCCAGAGACTCTACTACTGCCTTATACGCTGTACAATGCGGCAAATGTTTCAAATGGAGGCTGATTCCAACAAAAGAAGAATACGATGCCATAAGAGAGAGCTTCATTGAAGATCCATGGTTCTGCGAAAAAAACCCTGAAGTTTCCTGTGATGATCCTGCAGATATAGAGTATGATACTAGTCGATTATGGGTCATTGACAAGCCCAACATCCCCAAAACGCCCCCTAATACTGAGAGAAACCTGCATCTGAGAAAAGATTTTTCCAAGTTTGATATAACCTACATTATGCCCAATGGAAAGAGAGTGAGGAGCTCGACGGAGGTGGAGAAGTTTCTGGAAGCTCATCCGGGGTACAAAGATCAGTTTTCTGTGTCGGACTTTAGCTTTACTTCGCCAAAGATTATGGAGGAGATGGTGCCTAAAAGTTCTAGGGGGAAAGATTCGGTTGGTCGTAAAAGGAAAAATGAGGATGGATGAAGGTTGAATCTCTGTATGAGACTGTCGGATAAATTGCGATGGTAATTGTTATGTTTTATTGACATTTGTGGAGCTGAGTGCCTCCTTGTATAGTTGTTATTGGAATTTTCTGGTAGAAGCTATGCAGTTTACAATTTAATACATACGCTTGTTCCTGAATTCAGATTTGAGCATGCAATCAAAACCGTGAGACTCAGAGCTTTTTGC

>UN00795

TAACTAAGTAACGAATAGGTAGGACCAACGACAACCCCTCAGCCAGTCCCACTCTGATGAATCATAGTGTGGCAAAGCAGCGTGAGTGTGCTGTAAATATGTGATCAGCACCAACCAACCATTGACAACTAGCAAAGGCACGCCATAGATTCTAATCACCCAGCCCAACGAGAACGCAACGGTCAGCTTATACAGCGCATAAAGACTGCAATAACACCAGCATCGGAGATGAAAATCTGAGCACGCTCGCGATCGGAGTAGATCGGGCCATAGGGATCATAGTGGCATGCAAATCGTGGATAAGGGCGGCCAGAGACATTGAATGCCAAGTACAGAGGCCAGCCCATGGTGAGGCTGA

>UN00796

TTGTCTTTTGTACTAATTAGTCATGTAATATCTTGTATCTTTGTGAATAACCCCCAGGTTTTCATTTGGTGTTTGCTTTTCTCATGTCGTTGCAATGAGAAATTGTGCATAAAAGTCACATTCTTGGTTGACCCAAGAATTCTGTAAATACCCAATAATCGTGGAAATGATATTTATGTTTCTATC

>UN00797

TTTCCTTATTTTTCTTTGTTTTGTTCTTGTTATTCCTTTGGTCCGTTATTCCCTTGGGGCCTGCGAGCTGAATGCCAGGCTAGGAGTCTCATATGCCTGTGTTCCAAAATCTTTGGACGGAAAGCCTTCAATTCCAGAATTCATCCGGGAATTGACAGCTTCAAGCTTCATTGAGAGAAACTCAACTTGACGCTGCAGCGCTTGGATGTAATTGATAATCTCATCAAGAACTGATGCTTTTCCAATAACCTTGTTGCATCCAGGAACCAGGTCTTGAAGAATCTTCATCCGCTCACTTATCTTTTCTCTCCTAGCTCTCTCAGCGAGGCTATGACTGTCAGTTGCTTGACCCCTTCTTGCTCTCACATGGATGTAATCTTGCTTAGGTGCTTCTGATGGCTGGGTATTTTGGTCAAGTATTACTTGTTACATAATCCAGAACTTGCCTCTGCCTCAGTTTTTGATTCACCATTTTCATCTCTAGGTTTCATTCCCCTGATGCGCTTGGCTTCACTGTCTGTCGCGTCATTGCCGCTGCTAGTGGAGACGAGCTTAGATGACTCGTCCTCTGATGGCGG

>UN00798

AACCTAACTCGTCCAAAAGGCAGGAGTTATTATAATACTCGTAACATAATTTTTCTCGAGTACATACCGAATTTAAAAGCCTAGTGACATATCCCATATATTAAGGAATATACATAAGAGTCTCTATAGACTTCAAAAATACATATACACATCATCCACCTTTCAATGACCATTACACAAAGACAACATTCCAATCTTTTTATACCCTTCGTAAGAGGGGAACTCCAGCGCTCCCTCAAAAAAAAAGTAGTAACAAAACTAGAACCCCCTTATTCCAGTATACTTCAAAGAACACCTGTCCAGTTTCCTATATGATCCCACCACCATTGTTCATCTCCCTTTCAAGCATTAGTTTCGGCAAATATGTCGAGAAGCGCCAGTGCCTCAGGAACACTGAGCTCAAGCCTGAACTTTGGGCCATCATAATGGTGAAGAATGGGTCGGAATGAATCCTCCTCCAAAGGTTCGCAGATCTTCCTTGTCAAAACTTTCACCTGTGCTGGGAATCGAGACTCTCCATGTGATTTCTTATCCTCCCAAGCCGTTGGATCTATTA

>UN00799

TTCATAGACACATAACCGTCCATCAGACCATCACCAAATAAAAAAGTTTACACACTAGACAGAACATGCAAACCTTTTCTACCAATCAACCAGATAACGAGTTCTTCGAGACTGCAAAGATGCCCTAAGGCATCATCGTCATGCCTCCAGCCATTTATGTGGATAAAGGACACCAATGAAAACAAGCAGCCAACAGAGAAAGAAAGGGAATAGATAGAAAAGGGAGAGTTGAATCATGACTTCGGTATCTTCTCTTTTTCATCTCTAGATCTTGTCAAGCTTCTCAGCCTTGACAACAGGGTTGGCCTTGGCGATTGCATCTCTAGCAGCCGTCCGGTAATCAAATGGGCAGTTATGCTTTTCCGAATAGCGATGCATCGCGCAAAATACATTTCCACACCGGCAATTGAAGCCAGTCAAGCCTACCCTTTTCTTGCAGGCATTGCACCTGGTTGGACCCTCCTTCGCCTTTGGTTCGCTGCTCTCGCTAGAGACAGAAGCATCAGAATGGCTGTGCTGAAATGATCTTTGGCTCCACTGAAGCAACAGCGACATCAACACTGGCCGAAACAAGTGGCTCTTTTCCACTGGTACTCCCACTTCCATTCACAATGCTGTCGATGGATGATGCCGCCAGCTTAGCATGTTCCTGCTTCAATACAAGATCCTTATGGCACTTGGAGCACATATTCATGGTGGCTGCACTGCCAAAGAAGCCGCAGTTGTTGATGCAGAGGATTGGACCCTCCGGAGCTTGGCATCCAGTCTCATTGTGGTGCTCCATTGTTTCAGTTTTCGATTAGACGACGCCCTTCGCTGGCGCAAAGATCTCCCCCAATCTACAAGGTTGCTTACGACGATCCCCTAGCGTTCTGAACTCGCCTCGGAATCGTGGAAATTAGGGCTCGTAATTTGGGTGGAG

>UN00800

CTTCATTGTGTTGATTGGAACCGCCATGATGAAAATCTTATTTTAACAGGATCTGCTGACACTACTGTTCGTTTGTTCGACCGTCGTAAACTCACTTCTGGTGCTGTTGGGTCACCTTTGCATATATTTGAAGGTCACAAAGATGCTGTTTTGTGCGTTCAGTGGTGCCCTAACAAAGCATCAGTTTTTGGGAGTGCTGCAGAGGATTCTTTTCTAAATGTTTGGGATTACGAAAAGGTAAGTAAGAAGGTAGAGGATGCTGGAATAAAGACACTGAAGTCATCTGGTTTATTCTTTCAGCATGCAGGACACAGAGATAAGGTCGTTGATTTCAACTGGAATACAGAAGATGAATGGACCATTGTTAGTGTATCTGATGACTGTGCAAGCACTAATGGTGGTGGGACTCTGCAGATATGGCGCATGAATGACCTAATCTACAGGCCGGAGGGGGAAGTTTTGAGCGAGTTAAACACCTACAAAGAGCACATACTATCATGTGCACCACTTCGAGCCTGAAAGCTTCGATAGAGAGCTCAACTCCTTGCTCTAGGAGTTGACTGTACTGCTTTTGAAAGATATTCCTATGTAAACAGAGGTTATGCTTGTAACTGTTAATCATAGATTCAACTATTTTCTAGCTGGTCTGTAGATTGATTTGCTTGTGGAACTGTTAACTATATCGGCAACTTGGGAGTTGTAACTAGTGTTGTAAACTTAGTCCTAAGTACTTTCGATTATTTTTTGGGGT

>UN00801

GTCTAACAAATTAAAGGATGTCGCATTGCGTAGGAAAATTATGACCGTCTCAGGATGAGAAAGATGGTTTATCGTCATGGTACAATTGGGATGTGCTGCAACAATTTTGCTCGTCGATGCTTATAGTGTTGGTGGTTGTGGTGGCGGTGTCGAGTGTTTTTAGATTGGGCGGGGATCTGCTCTATGCACTTACTTTTATTGTAAGGTGGTCCTCTCTGTCTGTAATCCTTTTGCCCTGTTCTTATAACAGATTATTGTAGAATGCTTGTAGTGTATCATACAGTCGGTGTTACTACCCCCATGATTAATGTTTTATCATATAGGAGATGTTATTATCCTCATAACTAATGTTTTATCGTGGAGCTTAGTATTCCTATGATTGATGTTTAGTAAATGGCGAACATTTGCAAGG

>UN00802

AGTCATGCAGAACTATTTTCACTGCATAAGATTTGAGCAAACGCTCGTGTGCAATGTTTGCAATGAGGGATACAGTTTACGGATCGACTGTCAAAAGAGGAATCAAAATAGGAGTTGAGATTGCCTATACACCAACATCTAAAAAGCCAACGCCAAAACCACATGATGGATGAAATTTATAGATTGACTATCAAAAGAGTTGAGACGCGTATATACCAACATCTTAAAAGCCAATGCCAAAACCACCGCCATGCCTGTGGCACCCTGTGAAAAATAGATCAAATAAGAATGCCCCACATGCGAGCAAAGTGTTCTGAGCCATAACTATAAAGAGATGGTGGAAAACTCTAGATTTCTCCCTCTTTATGGTTCATAACACTTCACTCTCACGCAGTCTTCTGATAGAATTTGTACTCACCATCGTGGGCCTCCACCGACGCCACAACATGACTGGCACCATAAGCTCCGGCCTCTGAAGTTTAGAGGGCTCTTGGAGAAGAACATATTGTCAGATTTAAAGAGGACTAGGTGACAAAGTATGAACAAATTAGTTA

>UN00803

GTTTTTTTTGGTTTTCGTTTTAGGTTTACGTTTCTTTCTTTAGTTGAATGTGAGCATCGAGAAGCAGATCGATGGAAAGCTCAGCGGTTATATCAGAATTAGGAGCAAGACTCAAGGGATAGCCCTCAGCTTAGGAGACAAGATCACCCTCAAACAGAAAGGGGGCAGTTAAAGAGTACAGATGAACCTTTCCATTTTCTTAGTGTTACTTCTCTGCTCTAGTTTGTTATCTCATATTTTTGCCCGCATTTTGTTATTGAAAGTGGAACTGATTATTTGTTAATTATTTGTTATTTAGGTTTCGAATGCGTAGTCGATCCAGATTATATGTTTGTGACGTTGAGGTCTCGATCTTTATTTAAGGCTGTGTTTGAATTGCCAAATTCATGTTAATGACATCTATGTTGTGTGAATAGTAAAGGTCTTAG

>UN00804

AGTTTTTTTATTTTTTTATTTTTTAGTTTTCTTTTGTTTACTTAGTTCCTAACGTTAAGTTTGTATTATCCAATGAATAAACACAGATATTTGATTCCAACAGTCTAATACTAGATCTTTAACATTTCGTTATTTATATAAATGAACATTGGCGAATCTTGCCCTATAAATTAGCTACATATATTAACAATCCCATACATCATGAATATAGATACATTTCGCTAGCGTACGGATTGCTGGATGCTCGGTACTATGGATTTCACAAAGACTTGTATTGTTCGATCAAGCGCGAAGCATGAAGACGTGAGGTTGGAGTGATGGTGCACCCCAATTTCTGTAGGTAGGCAATCTGCTTAGGCGTAGCTTCAGATGATCCCATTGAGTTTTCAATTCTTTGTTTTTGGAAATGTTCCTGTTTTTCTCTCATCAAATGAGCACGTGTGTCTACTGGAAGACTAGA

>UN00805

GTTCAATCAAAGAATCTCAAGGGTTTATTTTGCACAATAAACAATCATTACAGTACACGGTCTGCTGTCAGTTCAGTCGGTTCACTTTCACAAGCAAAAGCCTCAGGCACTGGGGAACAAGGTAACCAGAAAACAAAGCGACGAAAGCAAAATATTACAGACGAGACACGACTATAATAGGTAATATATATATATAAGGATAAATACTATTTAACAAGGTCCGGGATCTGGGCACAAGAATAAAGGAGAAACACAACCAGAGCGCTGCAGAAATGAAACAGGCTTCATCGTCGACACTCAGGCAGCAAGCACTATGATAACGATAAGTAGGATGACACCAAAT

>UN00806

GCCTCCTTGCACTTCCATAACTCCTCTTCAAGCACTTCAGAGTAACGTTAAAAACACCAAATTTGTGCAAGGGTGTGAAAATGTGGCTTGTGCATCAGGCTCAATAGAAGAAGCTGTAAAGTTAGCCAAGTCAGTAGACCAAATTGTTATGTTCATGGGGTTAGATCTCACTCAAGAGAGGGAGGAACTCGATAGAGTGGATTTAGTGTTGCCCGGGATGCAGCAAACACTAATTACAGAGGTGGCCAAAGTAGCGAAGAAGCCGGTGATCTTGGTTCTACTGTGTGGTGGTCCTGTGGATGTTAGTTTTGCGAAGGATGATAAGAATATTGGGGGAATATTGTGGGCTGGTTATCCTGGTGAGGCTGGTGGACTGGCTATTGCTGATGTTATCTTTGGCAAACACAATCCAGGGGGGAGATTACCAGTTACATGGTACCCGCAGGACTTCACAAAGGTCCCAATGACCGATATGAGGATGAGAGCTGACCCTGCCTCCGGATATCCAGGCAGAACCTATCGTTTCTTTACAGGCACTCCGGTCTTCAAATTTGGCTATGGCCTCAGCTACTCAACTTACTCTTACGAATTCACCTCAAGAACTCAGAGTTCCCTCTACATGAACAAAACAATCAGTCTCCAATCTGTCAAGGACTCACAAAACACCATCAGCTATGATATATCAGACTTGGGCACTGATGAATGTCAACAGCTTGCATTTCCTGCAGCATTGAGAGTAAGAAATCACGGGCCAATGCCCGGAAAGCATTCGGTGCTTCTTTTCTTAAAAAGAGCTAATGTTCATGGTGGAAGGCCAATGAAACAATTGATTGGGTTCGAGAGTGTAGACCTTGCTGCTGGAGAGAATACACATATCGAGTTTGCTTTGAGACCTTGTGAGCATTTGAGCAGGATTGGGGAAGATGGCAAGATGTTGATAGATGCAGAGTCTCATTTCTTGGTGGCGGGGGACGAAGAATTTGAATTTAACATATTGGGCTGAAGTTTGTTGGTTCAAAGTTTGAACATTTTGGCTCAAGGAGATGCACTTGGATTTTTCTTGAATAATGCAATAACAAAAAGAAAGAAAATTAAAAATAAAAGTTTAAAAAC

>UN00807

ACGTTAAAGGTTAAGGTTTACTTAGTTTAATTATATGATGAAGAGAGTATTAATAACCAGATCAGTGACAAGTACTTCCTCACAAAACAATCTCAGCTAACCACCTAAGACAACCACGAACACCATTCTGAATATACATCCTACAAAACAGTTTTGGTTCAAGCACATTTTCCACTCCAGAGTTTCTGAAGTTCAGGAAATGAAAATATTCTCTTACAATTTCAAAAACAACACTAACATAAATATATATAATTCTACTCGTCTTTCTTGAATCTAGAGGGCACAACTTTGGCAGGAGTCGAGGAGCGCTCAGACTGAGTGAATAGAATGAAGAAGTGCATAACACTAGTTACCAACAATAAACCCAGCGACGAC

>UN00808

GCAATGGCTATAGGTAATCGGGAAGGGAAAATTTTTGTTTGGGAGCTCCAGTCAAGCCCTCCTGTTCTGATAGGACGGCTATCTCATGTTCAATGCAAGTCTGCCATTAGACAGACTGCCATGTCCTTTGATGGAAGTACCATACTTAGCTGCTGTGAGGATGGTTCAATATGGCGGTGGGATGCAGTGCCATGAAGATTCTTGGTATCAGCATTTTGCTTTTTTCATATTTTAGCCTGCCTTGCACCATCTGTTGCTGTCTGTCTTCATTTTTACTTGATGCTCGCCTCACGTTATTGATCTCTGTTGTGTACATGAAGAGATTGGATCGTGAGCTCAAAGCAGACAGAGGAGAATGCAAATACTCAGATGAATGGATCTATCCTGTATCTTCATGGTGTCTGTACGTTCAATCTGGTATCTGCACCGTGCATGATTGATGTAAAGATAGCTGATTCTTCCAACTTGACAAGGCTTTTACTAAAATGTCGCAATATTACTGTTGATGAAAAGTTGCATGTTAG

>UN00809

TAATTAACCTACAAGATAGAACCTTGGAATATGAGACAGGCTCACCAAAATGTTTAGAAAACAAAATGGAAGCTGCTGATAGCGACGACGAGGAGCCTGAACGGCCCAAAGGTAGTAGCTCTGCAAGCTCAAGTCATAGGAGCCAGTTAGCAGGATCTCAGTTGGTTCAAGAGCTTGGTACTGCAATCGACTCTGCTGTGAATTTACAACTGCTTGATCTGAGCAGAAACAAGCTCTCATCAGAAGTTATTGAGGTTTTCTACGGTTCGTGGTGTTTATCGAGCACAAGAAATTGTGGTTCAGCATATAGGCATGTTTCTATGGATGGAGGGATTGTTCATTTTTCCATGAAGGGGAAGAGATGTTGTGGGATTAAACCTTGCTGTAAAAGGGACTAGGCCATGTTTTGGTTTTGTTGTATATCACATTATTTATCTATATTAAGAAGATAATTATAGTCACATTTTTGGGTGTATGTAAAGAAGCTAAACATATCTAAACCATGTGATTGGAAATTTCTACTCCAAATTAAAAAATTCTATTCTTTTTTAGTTT

>UN00810

TGCAACGTAAATCCGTAGATTCTATAGCACTGGATGTTTCTGCTTGCTTATTGGAAAGGAGTTTGATGAAAAGAATCTTGTTTGCTATCATACCATGGGCAGGAAAATTCTTGAAAAATAAAGTTAGAAAGAGAGCTCAGGGAAAATGTTGGACGGGACCCCAACATGGTTTTGGTTATGAAAGGAATGCAGCACGGTATAGTTTTCATTTTGATCTGGTCAGCGGGAGTTTGTACAACAGAGTTGGAAGATGGAGGTGGTAGGAGGTAATTTTGTAAATTTCTGTGTGCAAGTCAGTATGGATGTTAGGTAGTATTCATTTTTGCGATATATTTGGGTAATTGTGATTCCATTGATGTCATACAGTCATGGAACAATGTAGGCTGAATATTAATTTATTAATAAATAATAGAATTTTTTTAACCCCCT

>UN00811

TGAGGTGTTCCTAAAGCGTGTTGAGGTTGCTTTTAACAATTTGCTGAGACCACTGAACGAGCATGCCGTGACAAATGCATGGAAGATTTGGTGTGCACGACTCGCTATGTTACCTGGTCCCTCCATAACAAGAATCGTGCTGGTCTCCGCCAATTCCTTGACTCTTTTGCTAGCATGGACCACTCTTCTTTTGGTGGAAATTCTTCTTGTTCAGCACCTACTCAGCAAAACTCATCCTTGGGGCCTGTTAACAATGACACAAAACTTGATAACAAGAAGACAGATACAAACTCTTGTAGTCATACAACAGAGACGAGACTTGTTGATCTTTTGGATAGTACATTATGGAATCGGAGGCTTGCACCGTCTTCTGAAAGGATTGTGTATGCATTAGTGCATCAGATCTTCCATGGCATTAAAGAACAGTTTTTGGTGTCAACTGAATTAAAGTTTAATTGTTTTCTTCTCATGCCGGTGGTTGACAAGTTACCTGCACTTTTGAGGGAAGACCTGGAGTCTGCATTCGAAGACGACTTGGATAGTATTTTTGATATCACTCAGCTGCGGAGCTCTCTTGGACAGCACAAAAGAGACTTGGAGATTGAACTGAAAAGGATAGATAGATTGAAGGAGAAGTTTAGGGAGATACATGGGAAGCTGAACTCAAATTGTGTTCATCCGAAGAACTTCTCAGCTTTGGCATAATCTCGCTAATGCAACTATGTAGAGTAGTGCGCATCGCAGTTTTGATTTTTGTTTCCAGTGGAAATAGGTTGTTGAAATCAGTTTTCTGTTTAATTTTTTTGGGGGGCAAAGAGAGTCGAAAATGAGGAATTCTTTATGCAGTGTTTTGTAATCTATAACATCTTTATACATGTAAGCCTGGCCCTTGTCAGTTCAGTGATCATTTTGTTGAAGTTGGGTTACTATCTGTAC

>UN00812

CACAAATTAATGTCTTCAAGTACCACAAGGAGCAAAATGTCTACTAAATCCACAGCCACCACAAAACAGAAGGAGGGAGAGGCAAGTTGCAGAGCTCGTAGGAGGATTATAGAAGCTCAAGAAGGCGAAGGGCCGACGGCGGCGAAGGACATCAACGAGTGTGCTGATGATTTCATAAAGCGGTTCAGGCAGCAGCTCAGGTTGCAGCGCATTGAGTCGTTGGAGAATTACCGGCAGATGCTACAGAGGGGACTCTGACTTTTTTATATGTTTGATGAATATAAATGGCGCGTCTGAGATTGTGTTTTTGTGCAATTTGAGTTCATTGGCATTGTTTTATTTTTTTTTCTTCCTTCCTTTTTGGGAAATGTGGTCAGTTTAGGGATTAAATGGTGTAGTGTCTGAGATTAAATTATAATATTTGATTCTCATGATGATTTCAGATTTTTAATTTGTACATTATATATCGGGGTATTTCCCCACG

>UN00813

TCCTTACGTTGTTATTGTCTTTTTGTCGGAGCATGGAAATGATCTGGGGAGTTCAAAGGATGTGAAGGGAAACCCCACCGAATTCTTCAGGCGAGCTGGTCATGGCTCATCCTATGGTGTCGGTGCAAAGCTTGGTTTAGTTAGAATGGAATATGCTGTTGACCACAATGCCGGCACTGGATCCATATTTTGTAGGTTTGGCGAGAGATTTTGAGATAGAGAGGGCGGGAGCGAGGGAGCGAGCATCTAGATATGGTATTCATTTTGTGTCATTTCTACTTGTGAAGCCAGGAAGAGAATTAG

>UN00814

ATTTTCTTTGGTTCCGTTCTTACGTTATTGTATTCTTCTAAGTACTGCCCAGGATAACATCGATTTGCAAACTACAATTCTTTCTAGATTTGATTTAATCTTCATTGTGAAAGATATGCGATTGTACGATCAGGATAAGAGAATTGCTAGCCATATTATAAAAGTCCATGCCAGTGGAGCTGCAGCTTCCAAAAACTCAGATGGTATGGAAAGTGAAAACTGGCTCAAAAGATACATCAAATACTGCCGGGGTAGCTGTCACCCAAGGCTATCTGAAAAGGCTGCTGAAATGCTGCAGAATAAATATGTGGAGATCAGACAGAGAATGAGGCAGCAGGCTCACGAAACTGGAAGATCTGCTGCAGTTCCTATTACGGTGAGGCAGCTCGAAGCTATAATAAGGCTGAGTGAGTCTCTTGCAAAGATGAGGCTGACTCTTGTTGCTACTCAAGAACATGTTGAAGAGGCATTCAGATTGTTCAATGTTTCCACATTGGATGCTGCTCGATCTGGAATCAATGAGCATCTGAACTTGACTCCTGAGATTGCAAATGAGATTAAGCAAGCTGAAACACACATAAAAAGAAGAATGGGCATCGGTAGTCACATATCAGAACGTCGTCTAATTGATGACCTTAGCAGAATGGGAATAAATGAGTCTATTATAAGGAGAGCTCTACTTATTATGCATCAGAGAGATGAAGTGGAGTACAAGCGAGAAAGGCACGTCATTGTTCGCAAAGCTTGATGTGCTATGTTATGACTTGCGACCCTAAAGCGAAACATTAGGATGATAAGGGATCACGTTGCCATATTTAGATCAGCATTTCTCCATTGTGAGGTTCAAATGTTTTCCATTATCCGCAGGGATAGAATTACAAGTAAAAGAAAGACTTAAATAACTAAAAGAAACGT

>UN00815

TCCGAGTTAAGTTCCTAACCCTTTTTCTCTCCTTGGCGGCTGCTAGTTCTTGCGATTTCAATTAGTTAAAGATTTTATTTGGACTTTAGGCTTCGGTGAATATGGCGGATGATGCACCAAGTGAGATCCCCGAGCTTACACCCTTCGATCCAACAAAAAAGAAGAAAAAGAAGAAAGTAGTAGTTCAAGATCCATCTGAAGAGATAGATAAGCTGGCTGAGAAAGCGGAAGATCTGTCAGTTGCTGATGTTGAACCTAGTTTTGTTGGAACGAAGAAGAAGAAGAAAAAGCAGGTGGAAACTGACTTCCTTGAAGATGAAAATGGTGATGGCGGTGAGGATGGCAATGGGGATCATTTTGGAGAGGAAGAGCAAGGAGAGGGCATTGAATTGGGAGGTACCCGGTATCCTTGGGAAGGCACTGACCGAGACTATCATTATGAAGAGCTTCTGGGCAGAGTGTTCAATATATTGCGTGAGAATAATCCAGATCTTGCTGGAGATAGGCGAAGGACAATTATGAGGCCTCCGCAAGTTCTTAGAGAAGGAACTAAGAAGACTGTCTTTGCAAATTTCATGGATTTATGCAAGACGATGCATAGGCAGCCGGAACATGTCATGACATTCTTGCTGGCTGAGCTGGGTACAAATGGATCGCTTGATGGACAGCAGAGGTTGGTTGTTAAGGGAAGATTTGCTCCTAAAAATTTTGAGAGTATTCTGAGAAAATACATCAATGAATATGTCATATGCAATGGCTGCAAAAGTGCGGATACTATACTTTCCAAGGAGAATCGGCTGTTCTTTCTTCGGTGTGAGCAGTGTGGTTCTTCACGGTCCGTTGCGCAGATCAAAACAGGTTTCATGGCTCGTGTTGGTCGTCGCAAGGCTTGAGCATCATATCTTCTCCCACATCGGTGCTCGTTTTCGAGAAGCGGAGAAAAGCTCTTTTTTGCCTTGCTTATTTATAACCAGAGAACTTTCTAGCGCTTTTCACACATTTGGATCTGTTGCAATAATTTGAATCTTGATTAGCTTGCTCTCCCCCCCCCCCCCCCTTTTTTTTTGAGTCTTTTTATTAGTTTTGGATCTGTTGCGATAATTATTCTATTTATCTATTGGATG

>UN00816

AACCCAAAACAAGGAATCATAACATTTCCTAGAAAATCACAATATATACATCAGATGGAACTCTTGCTAACGTATGTGACATGGAGAAAAACAGGAATTTCCTACATATATTACACATATTTTGCAATAACGGGAGGGGAAAAAAATCATACCCATTAAATGGTAATGAAAGAAGAAGCAGGCGCTTTTCAATCTTTTTCTTCTTTCCCTTCCTTCAAACTCATATCTGAAGAGTTAAAACTCCCATCTGTCACTGAAACCGCAACGTCAGAGACCGACGAACATACTGTCGATCCTGCTTGAATCTCCTTAAACATTGCCATCACCTTCACCATTGTAGGCCGTCTCATTGGCCTTTCATCCAGGCAAGCACAGGCAATCTTCAGATGTTCAAGAAGCTCGAGTTCCAGAGAGGGATCCTCTTTAATAAGCTCCCGATCAAACACATCACTAATCTTAAGCTTCGAATGCAGCTTCACCCAGCCAACCAAGTTGTTGTCACCGAAATCCGAAGAGTCCGTTGGTAATCTCCCTGTGAGCAACTCGAGCAAAACAACTCCATAACTATATACATCGCCTCTTGTAGTGCACCGAAAACTCTGATAGTACTCTGGAGGGACATAACCAGGGGTGCCCGCCAGTGTTGAAACACTCAAATGTGTGTCCATTGCACTCATCATCCTTGCCATCCCAAAATCAGAAACCCTAGCTTCCAAGTTCTCATCCAAGAGGACATTGCTCGATTTCATGTCTCTATGGATTATATGAGGAATGCAATTGTGGTGCAGAATGCTAAGCCTCTGGCAGCTCCCACTGCAATCTTTCTCCTCGCCGCCCAATTGAGCTTGATCCCTGCCTTCTTTCGATCGTGGAGGACATCTTCCAGACTTCCATACTTCATGTA

>UN00817

AGACGGTCCGCTTCGTTCGTCCTCGACAAATCCCTAACCTGAGACCTAGCATCTCCGCCTACTACCAGACCCGATCCGAGCACCACGCTGTTGTTACCAGCGAGTGGCTCGCCCAGGCGTCGTCGGCCGCTGTCAATTCGTCCACTCCGTCGCCGCCGTCGGAGGAGGGCTCCGCTGGGGGAGGTGGCGGGAGGGGTTTTAGTGTGATTAATGAGTTCAATTTCTGGAGGAAGAAGCCGGATTTGGCTGAGGTTGTGGCGGCGATCATGGCGCTTGCGGCGGTGATTCGGTCGAAGTAGAAGGACGAACGACGATGATGGAGCTCGAGATCGAGCTGAAGAAGGCGTCTAATGCGCTAAAGTTGCATTTGGAGCTGAAGTTGCTGTTGTTGCTGCTGCTGCTGAAGTTGCTGAAGCTGCTGCTGCCGAAGCAGTTGTTGCTGCTGCTGCTGCTGCTGAAGCTGTGTATTTTGGTTGATTCTGTAATATGTTTATACTGTTTCCTTGCGTTGTGATT

>UN00818

GGTTTTTGGTTTAGTTAGTTTACCTTAAGGTTGTCTATTACAAGATCTCCAAGGGGTTGCTTGATAAATATGGTCCTGAAAGAGTTGTTGACACACCAATCACTGAGGCTGGATTTACCGGAATTGGAGTGGGATCAGCTTATTATGGTCTTCGACCTGTCATTGAATTTATGACTTTTAACTTCTCTATGCAGGCAATTGATCATATTATCAATTCTGCTGCAAAATCGAACTACATGTCAGCAGGTCAACTATCTGTTCCTATTGTTTTCAGAGGACCCAATGGTGCTGCTGCTGGTGTTGGAGCTCAACATTCACAGTGTTATGCAGCTTGGTATGCATCTTGCCCTGGACTGAAAGTACTAAGCCCTTATTCATCAGAAGATGCTCGGGGCTTACTCAAATCTGCCATTCGGGACCCTGATCCTGTTGTTTTTCTTGAAAATGAACTTCTATACGGAGAATCTTTTCCTGTGTCAGCAGAAGTTCTTGATTCCAGCTTCTGTCTTCCAATAGGAAAAGCTAAGATAGAGCGTGAAGGGAAGGACGTTACAATCACTGCTTTCTCAAGGATGGTTGGGTTTGCTCTCCAGGCTGCAGATATACTTCTAAAGGAAGG

>UN00819

ACATACATCGGAGGCGTGTCTTGCCCATACATATGCGGGAGACATTTGGACCACGGTGTTCTTCTTGTGGGTTATGGGTCTGCAGGATGGGCCCCGATCCGCCTCAAGAATAAACCCTACTGGATTATAAAGAATTCTTGGGGTGAGAACTGGGGAGAGAATGGTTACTACAAGATTTGCAGAGGCCACAATGTCTGTGGTGTTGACTCCACGGTCTCTACAGTAAGTGCTCTACATGCCACACCCCAGGGTGAGGTGACTCTAGCCGAACGCAAGAACATGTGAATAAAATTCTAGTGGTTCTCAATCTAAACTTTAGGCTTTATATTATATAAATCATCTGTAATATCTGATAACTTGATACTAGGCTTCTCTGCAACTTTACGTCTCTGAACATTATGAGGCTAGTGTGTATTTATCTTCACTTGTGCCTTTCTGTACAGAAAATGCTTTTAATGATTTGGATGTTTC

>UN00820

TTTTGTCGTTGTTCTCAGGACATTATTTGTCATAAATTTTGATCCGATTAATACCAGGATGAGAGACTTGGAAAGGCATTTTGAACCGTATGGAAAAGTTTTAAATGTCAGGATCAGAAGGAATTTTGCTTTCATTCAGTTTGAGTCACAAGATGATGCCACCAAAGCATTAGAAGCCACCAACATGAGCAAGTTGATGGATCGGATTATAGCAGTGGAATATGCAGTTCGGGATGGATGATAAGAGAGAAAAAAATGGATCCAGCCCAGATAGGAGAGGAAGAGGATTAGGTATCCAGAAAGGAGCAGTCGTGGTCGTGAGCGTTCAGCTAGTCCTTATGGCAGAGGCGTGGAGAGGGCTAGCCCTGATTATGGTCGTGGTTCCAGTCCTTACAGCAAGCCTGAACAGAGGGGGAGCCCAAATTATGGAAGCGGCGAAAGTCCTGCTAATGAAAGACATCACAGCCGCAGTCGCTCACCAGCAAGGCAAGAGAGAAGTCGCTCACCAGCAAGGCAAGAGAGAAGTCGCTCACCAGCAAGGCGAAGAAAGATCATAAGATGAATTTTCAGTTCTATCCACCTGCATATCTTAATATAGCGCAGGTAATTCTTGTTATTGTATTATTGGTTTAAAATTGTTTACCTTTTAAGGTTACTTAAC

>UN00821

ATTTTTAACTTATTTTTTGTTCTAAATTAAATTGTCTTTGTCTTATTTCATCTGTATATATCTTTACAAGTATTTTAAAAAGTGAACCAGACTGCAACCTTCCATTTAGCTACACATTTTGCATATAAATTAACAAGATACAACATTGTTGGTAACGCAGTCTAAAAAACCCTCTTCCATTATTTTGAATGATCGATCTGCATTCCAAACATAGCAGCCGCATAAGCTGAAATCATGCTATTACAATTCTTGGGCAGGTTCAAAGCGTACTAAACTACTAGTTCATATATGTTCCCATTAACAAGCTTTTCTGCCAACCTCTGATTCTTCACCATTGCAAGCTTCAAGGCATACGACAATTTAGA

>UN00822

GTCCTAAGGTAGGTTGTCCTTGACCTTAGCAAGTGTATTGCTCTATCCTAAAACCTTGGGTGGTGGTGCAGTTTTTAGTGGATGGGTTCCCTTTTAATTCATCAATCATAGATCGCATTTCTCCTGAAGCCAGAAAGACCCCGATTTTGTGGTCTCATGGAATGGCTGACAGGATGGTATTGTTTGAGGCTGGTCAAGCTGGTCCCCTGTTTCTTGAGCGGGCTGGCATGAAATGCGAATTTAAGGCTTATCCCAACCTCGCCCATTCGATAAGTAATGAAGAACTGAAATCACTGGAGTCTTGGATTAAAGCTCGTCTGAACTCTTCTTCTTCTTCATGAAGATCGCATTTTATGTGGTTGTTTCGGCTTATCCTAATCCATCTCGTGTTTATTACCGATTTGCTATATTTAGAGAATATTGCCTGGAAAATGCTGGGAAAAAATGATGTATTCTAATTTATTTCAGATAATTGAGCTGTTAGCTGCTGAAATGTAGTTTTCAATTGGATATACCCTCAAGTTCTCATTGGTGGGAAACTCTACTCAAATTCTAATATATAATTCGTATCAATTTTGTTTTAC

>UN00823

TTTGGTTTCTGGGATTCAGTATTTAATAGGAGCACACAGAACAAGCCACAGACTAATCTTCTGCTGTTTGAAAGAAGGCTTCAACCTCTGCAATGCTGGCTTCAGCTTCATTTATCTCTGTGCCTTCTTGGTTTGACTCCTTAACCTCTACCAGTGTTGCCTTCAAGTCTGCAAGGGAGGACTCCAGTCTTTTGCGGCAATCGGGGACCATCATTCTTGACTCTGCCAGCACATTCTCCTGCTGCTTGAGATCGTAGGGATCGGCGCCTTTCTCCTTCATGTCGGCGGTCTTCGCCGCTTCTCTCTCGACCTCCTTCTCGTAGTAATGGAGCTCCTTGAGCACGCGCTTGCACGTGCTCGTCTTGATCTTCAAATTTACGAAGACGTTAGACCAATTAGAATAACAAAACAAAAACAAAAATAAACTAAAACTAAAACGAAAAA

>UN00824

TTGTCCTGTTCGCCACCCTCCACAACCCCTCAACCCTGCCTCTCACTGTCGGATCACTCGTCTCCGACCCAATACAAGTTACTAGAACCCCAAAAAATAAGGATGGCACAAAGCAACGCCTGCGAGCTTGGCTTCCCTTCTGAACAGCAGCGCATCGCAACGCTGTGTGCGATGTTCGCGCCTGCGCTCTCGCCGACTACGAACACGCGGGAGAAGTCCGCGTGTTGGGCGAGCCAGGGCTCAGACCCAGAAAGGGCCCAGTTGAGGGCGGCCCAGCAGTCGTCGTAGGCGGCAGGGAGAGGGTGTTCGGGGGCGAGGCGGTAGTCGACGGATACAGCGAGGGATTGGGATTCAGCGACGAGGGAGGTGAGGAAGGGGTTGGGGTTGAAGGCCGAGCCGATGCAGAAGCCGCCGCCGTGAATGTAGATGACGACAGGGAGTTTGGCGGAGCCGGTGAGTCCTGGCGGGAGGTAGAGGCGCGCGGAGATGTCGGAGTCGATGGGGACGTCTTTGGTGGTGACGCCGGAGGTGGGGTCCAGACCGGCAGGGACGGTATCGGTGCCGAGTAGGCGCTCGACACGGCCGCTTTTGTATTGGCGGAACATTGGGGAGCAGTCTATGAGAATTTCGGTGTCCGGATCCATTTCCGCGGCCATTCGGAAACCAATGAGAGGAGGAGGAGGAAGAAGAATAATAACCGTAAGT

>UN00825

GTAGCACTATTGGAAGAATACTTGATCAGGGACGACTGTGGTTCTTGTTGGGCATTTGGTGCAGTGGAATCATTGTCAGATCGTTTCTGTATCCAATTTAACATGAACATCTCTCTTTCTGTTAATGACCTCTTGTCCTGCTGTGGTTTTATGTGTGGGGATGGTTGCGACGGAGGGTACCCTATACGTGCATGGCACTACTTTGTGCAGAGTGGTGTTGTTACTGATGAGTGTGACCCATTATTTTGATGAAGCTGGATGTGTCCATCCAGGCTGTGAACCTGTCTATCCGACACCACAGTGCGAAAAGAAGTGCAAAGTGAAGAATCTTTTTTGGGAAGAAACAAAGCACTTCAGTGTTAATGCTTACAGAATAAATTCTGATCCTCGGGATATTATGGCAGAGGTCTACAAGAATGGTCCTGTTGAAGTTTCCTTCACAGTTTATGAGGATTTTGCTCATTATAAGTCAGGGATCTACAAACATATTACAGGCGATGTCATGGGTGGACACGCAGTTAAGTTGATTGGTTGGGGAACCAGTGATGATGGTGAAAAATATTGGACTTACTAAGAACAAAA

>UN00826

AGTTTATTTCTTAGTTAGTAGGTAGGTAGTAAATTATTTGAGAACTGAAGGCGATGGCGGCTGCTCGTAGGATCACTTCCAGTATTATCTCTCGCTGCTTATCCGCTTCTCCTCGTTCTATTCTCCTCAGAGGAGGTGCCTCATCCGGAATTCAAAGGTATGGCACTGCTGCTGCAGTTGACGAGCAACCGATCACACCACCCGTCAAAGTAGAGTACACAAAGCTTTTAATAAATGGAAATTTGTGGATTCGGCTTCAGGAAAGACTTTTCCGACCTTGGATCCTAGAACCGGGGAAGTTATTGCTCATGTTGCAGAAGGAGATGCTGAAGATGTCAACCATGCTGTTGCTGCTGCTCGCAACGCATTCGATGAAGGACCCTGGCCAAAGATGCCCGCTTATGAAAGGTCACGCATATTGCTAAGGTTTGCGGATTTGATTGAGAAGCACAATGATGAAATTGCAGCGCTAGAAACTTGGGACAACGGGAAGCCTTATGAACAGGCTGCCCAGGTCGAAATA

>UN00827

GGCCTAGAAATTCCCAATTCCAAAAAAAGATTTAGAGGAAAATGGGGAGCTCAGATTTCTCATGGAAGCTTGCCGATCATCCTAAGCTTCCAAAGGGGAAGACGGTGGCCGTTGTTGTTTTGGATGGATGGGGCGAGGCTAATCCTGATAAGTATAACTGTGTTCATGTTGCCGAGACGCCAACTATGGATTCGCTCAAAAATGGAGCTCCTGAAAAATGGAGATTGGTAAAAGCTCATGGCACAGCAGTAGGACTTCCTACCGAGGATGACATGGGGAATAGTGAAGTAGGTCACAATGCATTGGGTGCTGGCCGGATATACGCTCAAGGAGCTAAGCTAGTTGATCTGGCTCTTGCCTCCGGGAAGATATATGATGGAGAAGGCTTTAAGTACATTAAGGAATGCTTTGATCATGGTACTCTACATCTTATAGGTTTACTGAGTGATGGAGGTGTGCACTCTCGACTTGATCAGTTACAGTTGTTGCTGAAAGGTGCTGCTGAGCGAGGTGCCAAAAGGATCCGAGTGCATATCCTTACTGATGGTCGTGATGTTTTGGAATAGGTTACAAAGTAGTAAGGAACTTAACGTAAGGAAAAACAAACTTAA

>UN00828

CTAAATACTCCCATATGATGCTAACATGTTAATTTTATTCCTTTCAATATAAACAAGAACAAGGGATTAATCTCATTCATGATAAACAAGAACGGAAAAAAAAAAATTGACCATGCTCTTGCTCACGCGTTAAACCATGAGTAATTATAAGTTGCACACCAGTTATTTGAAGCAAATAAACAACAAGAATGTCATTTACTTCCTAGAACATTTATTCATCACTTCCAAGCAAAGGTGGCTCCCTCAAATTCATGGTTTCTTTTTACTGCCTCATGGAAAATTTAAATAGAAAATAAAAATAATAATAACCACACCAGGAAATCTCTATGCAGCCTGAGCACCGAGTTCCAGGAGGAGAGTGTGGTTGAGATCATTGGAACCGGGGCCAATAAACTGAATGGGGCCTGGGCTGATGTATCTATTCTTGACGGCCCATTCTTCACGCATGGATGCGAACTTCCTGAATGGTGCACCTTCAAGCTCGAACCAATAAGACCTTACTTAGAATAACAAAC

>UN00829

CTTAGTCTGAACAGTAATGCTTGATATCAATATTTAATGTTTTAACATTGACAGTTCTAAAGCTAGCAAGTTTATGTAGTGTCATTAACCATGCTACATATTTCAATCTCACACAATCCCATATGAAAATATTTGTTGAATAAAAAGAATCAGTAACTGGAGCCTGAATAGTATATATGCCGTAAAACTCAAAATTATTTCTTCCGGACATTGAATAATCTAATAATAGCCAGATGGAGAGTAGCCAGATTGAGGTTGAGAGTCCTGAGGTTGAGACGGTGGAGGTCTTGGCCGGGCATACTCTGCAAAGATGACCCATCCATCGAGATACTGGCCATGCATTCCCTTTATGCCTTCAGCGGCCTGTTCTAAGTGGCATATCTCACAAAACCAAACCCCTTTGAGTATCCAGAAACGCGGTCTGTTACAACTCTAGCGTGGAGAACTTCACCAAATTTTGCAAAAGCTTCTCTTAACCCCTCAGATGTAGTCCTCTTGCTAAGACCTGATACGAAGAGGTTGGTTGAGGGCTCCGCCGCAGGCTGGGCGGCGGCAGGCGGCGGCGGAGTGAATCCAGGAATCGAAGAGATCATTCGCCGGAGTCCAAAACGGCTCGCTGCAATCGCCATCTCTCTCTCTCTCTCTAAAA

>UN00830

TTATCTTCATTCATGCGTTTGATGGAATTTACAAAAGAAAATGCTTCCGGAGAAAATGGAAATATTCTGCCTTTTCTTCTTCAAATATCAGTTGGATATAAAGAAAGTTGACAGTAACTTAGAATGAGATTTCAAGTAAATAATATGTTATTTTGTTAGCATAGCAGCATTTTCAAACATCAGATACAGACAATCTTCCGCCCTAACAAACAATGTGCAAATTTACTTAGTTTCAACTGTATGGTAAAGTATTAGAGGAAAGACACGAAATCCACTCAAAAGTGAGAAATAACTTATTGCGAGCCTGCCCCCGATAAAAAAAAACTATTGCCGAGCCTCTTCCGTTCAGGAAATTGGATTCTGGACATGAACAAACAACAACAGACTTTTAACAAAAGGAATCATGAAAGCATCCTCTTCTTGGCCACAAAAGAGTACAATCAATCCCACCAAAACCTTTTGTTATCTAATCATTTTGCGGGAAAATGTGTACTTAAGAAAAATTCCCCCGAGTATCACTTTTA

>UN00831

ATCAGAACAAAAAAGAAACCCCGAAAAAATTAACCCCAAAAAAAGGAAAAATACCCCAAAAAGAATGAGACTTTAACCACAACATGCTAGCAAGCAACATCAAGGGCGTAACCAGCGGCTACCCCCTCAGGATCGAGGCCCATCAGTCCGCCGCCAAGGAAGCCGACTTCAACGCCGACTTCCTCCGCAACGTCTTCCCCAAGATCGACTGGCCCGCCCTCGTCTCCGCCGCCAAAACCCTGGGCTACGGCGATCTCCCCGAGGAGGCCGAGCCCTCGATGCTGGAGTCCGATGAGTTTCTTCGCCGATTTCACCACGCGCTGCTCGAGGTTCACGTCGAGGAGGGGGCGCTGGTTTGCCCCGAGACCGGGAGGAGGTTTCCTGTGAACAAGGGGATCCCTAACATGCTGCTTCACGAGGACGAGGTCTGATTTTTGGGGGAGGGTTTGGGTCTCTTTAGGGTCTTGATTGTGGGTTTGATGATTTAGATGGGATTTTGATGTTGCTGGTGTTTTTTTTTTTTTACTTTGTTTTCGGTGACGAGGTGTTATTAATATAGATTGTTCCTAGTTTTCTTGTTGTAGAAAAAAAAA

>UN00832

ACGGGGGGTTCTATTTAGTATTTTTTTTTTAGGTATTTCGGGGTTTTTGGTTACCTCGTAGGCCTCGGAGATCTGCTTGAACTTGGCCTCGGCCTCCTTCTTGTTGTTGGGGTTTTTATCGGGGTGCCACTTCATGGCGAGCTTTCGATAGGCTTTTTTGAGGTCGTCGTCTTTGGCGTTCTTGTCGACTTGCAGGATCTTGTAGTAGTCTACGCCCATGGCCGCTGATGCTAAGAAATCTGGATCGGAAACCAACGGAAGATTTCTTTTTTGGTTTTTTTTTTTTTTTTCTAAACAGTTTTATCGTTTGAAGAGAGAGAG

>UN00833

TAACCTAAAACTAAGGAAAATAATACAATAATACAATGACAGCAGCATCTTAACAAGATATTGAACAGTGACAAGCAAAAAAAATGCTCCTGAAGCATGATTAAGTATTTCAAACTGATAAAAGTTACCTACCAAAATCGGGTAAAAATTAACAACCATAACATGAAGCAAACAATTCCCATGTATGACAACTACATCAAATTTGTAAAAGCACCACCGACATTTTTTGCTAAATTCACTGGTCACCAGGGAGGGTCTTGATGATGTCTGAATCGCCAGGATCGATAATGCTGAGGCAGCACACTCGGTAATATTTGCCGCAGGCCGTTCCAAGATCAACATTATCTTTAGAGCTCCTGAGGGATCTGAGGACGGTCTTGAACCCTAGAGTGTACTTGCCACTCTTCATCACCAGAGCCAGCCTGTTGTTTATGCTCTCCGTCGTCTTCTTCGTTTTCTTCGTGGGCGCCATTGAAGCGGAGCGGAGAAACCCTTGAAGACTAAAACTAACGAA

>UN00834

GTAAGTAAGGTTGGTTCTTTTCAACGACAACATGGATAGGTGGAAAGAACGACTTCCTCGGCATTGCTTATCTTAGTGTTGGTGGACTTTGTTTTTTTCTGGCTCTATCCTTTACCATGATATATTTGCTATACCCAAGGCAACTTGGGGATCCATCATATTTGTCATGGAACAGAAATCCAGGAGGGCACTAAATTGTTATTATCGCCTTCATCGGAAGCATTTTGTAGAAACCAAACATGTTCCAATGTCTGTAGTATAGTTAAGTTAAAAAAGAAAAACCCATGCATTTATTTTTCTTTCATGCGTCTTATTCTACCTTTGAAGCCTTACTGTTTGGTTACTCACGTATGATTTGCTTTTGTTTGAAACTTTGTGGTATCTGTCTTTATTATAAAAATAAAAGCTAGTTAGGT

>UN00835

TTACTTTAAGTTCCTTTTATTGTTCCGTTTCTAAAGGGGTAGGTAAGTTTTGAAAAGGCTAGGGGTAGGAGGAACAGCAGGGGCTTTAGGAACTGAGTTATGTTTGTTATTATGCCGATTGCTTGTTTTGTGGAGAACTATGAAATATTCTAGGCTTATCTTCGTTATTTCAGTTGATTCAGGAGAGAACATCAGAGCTCTCTTTTGTTTGTTGCAGCTCGCTTATAGGATTTTGAAATCTATTGCGTGGGACACTGGCTAGGCCGCTTGTTTTTTCAGTTGATTCAGTAAAAGACTATCAGAGTTCTCTTTTGTTTGTTGCAGCTTTTC

>UN00836

ATTTATTGTTAGTTTGTAATTCTTTCTAGGGGTTTTTTACAACTGTGGATAAGGCATCAGCTCACTTGAAGGGTGGTGCAAAGAAGGTGGTCATATCAGCTCCATCGGCTGATGCTCCAATGTTTGTTGTTGGAGTTAATGAGAATAAGTATCAACCTAACATGAATGTTGTTTCAAATGCAAGTTGTACAACTAATTGTCTTGCTCCTCTAGCCAAGGTAGTCCATGAGGAATTTGGTATCGTTGAAGGTCTTATGACAACAGTCCATGCAACAACAGCAACCCAGAAGACTGTTGATGGTCCCTCAATGAAAGACTGGAGAGGTGGCCGTGGGGCTAGTCAAAATATTATTCCCAGCTCAACTGGTGCAGCGAAGGCTGTTGGCAAAGTTCTACCAGAATTAAATGGGAAACTTACAGGGATGGCTTTCCGTGTTCCTACACCTAATGTTTCTGTTGTTGACTTAACTTGTCGACTTGAGAAAAGTGCTTCTTATGATGATGTTAAGGCAGCTATCAAATTTGCATCAGAAGGGCCACTGCTAGGCGTACTTGGCTAC

>UN00837

TTTAAGGTTTTCTTTGTTAGGTTCTTTCTAGGTCTTGGTTCACAAGTACTGCCTTTTGGGTAATCTATCCAAGGAGGTTGGATGGAACCACTATGATACCATCAAGGAATTGGAGGAGAAGAGAAAGCAGAGGGCACAAGTTGCCTATGAGAGGAGGAAGCAGCTGGCCAAGCTTCGTGTGAAGGCAGAAAAGGTTGCAGAAGAAAAGCTCGGTCCTCAGCTCGAGATTCTAGCCCCGTTGAAGTATTAGAAGCGTTTTGTCTGAAACTTCAGTTTTGTTGCCTTGTCTTATCTGAAAACTTCGTTTGTGGACTTTAGGATTATTACCCTAGCTCATTATATTGCATAGGCCTTGTTATTTTGAGCCTGTTAATCAGATTCTGTCTATTGTTCGGTATTCTAAAATTGTTAGTTTTTGTTTTTGTTTCTTTTGTTTAAACTTTT

>UN00838

CTTAAAAATTAAGTAATTAATTTTAATTTATTTTAGTATTAGAAAATAGCATGGGACAATTAATACGTACGTACATACAACAAATACAAGAATTCATACTCGAAGGCAAGACAACAGGAAAAGAAGCAAAGAATTCATCTAAAAGGTCCTCGCACAAGCTTTCCACTCATCTTAGAGCTAAACAAAACTATGAGGAGATGCAGGGCTGCATTCCTCGGGTCTTTTATGTGATTGTTATTATTGTTCCAAGAATTCCTCACTCTGACATGAAAAGCTTGATACAAGAACCTCGCCCTCCTCCAAGCCTCCTTTATTCGACTAACACTGAGACCTGGTTGATAGGAGAGGGGTCGTAAGTAGTTTTATTGTCTTTAGTTAGTTGGTTAGGGGTTTGTTA

>UN00839

GTTACCGAGTGCCGCTCACGCCCGGTCGTACTCATAACCGCATCAGGTCTACCAAAGGTAGAAACAAGCCTACTGGCCAATGGAACAATGTAGGCAAGGGAAGTCGGCAAAACGGATCCGTAACTTCGGGAAAAGGATTGGCTCTGAGGGCTGGGCACGGGGGTCCCAGCCCCGAACCCGTCGGCTGTCGGCGGACTGCTCGAGCCGCACCCGTGGCGAGAGCGGGTCGCCGCGTGCCGGCCGGGGGACGACTGGGAACGGCCCTTCCTCACGGGGAGGGCCTTCCCCGGGCGTTCGTAACTAGTCCGTACTTCTAGTAACTTGGTACGGACAAGGGGAAATACCGAACTAAGTTT

>UN00840

GTTTTTTTTGTTTCCCGGTTCGGGGTTCTTTGGTTCCGTTCTAGGGTACGGTAGGTAAAACGGGTGGTGGCGTGCAAGAGCGCGTGCTCGGCGTTTGGATCGCCGAGGTACTGCTGCACCGGGAGCTTCGGCAGCCCGCAGCAGTGCAAGCCCACCGCCTACTCCAAGATCTTCAAGTCTGCGTGCCCCAAAGCGTATTCGTACGCGTACGACGATCCTACAAGTATTCTTACCTGTAGCGGGGCGAGCTATCTCATCACTTTCTGCCCTCATCACTGATTAATGATATTTGGTTGGGTGTTATTAGCTAGTGAACCATGTGTCAGTTTGATCAGGGTGACTAAGTACGACTTAATAAAAATAACTAACCTAACTAAGAAC

>UN00841

CGTATTTCGAAATCCTTTTATTTTTTGTTTGGTTCAAGTGCTCGCTAAACAAACAAACCTTGGGACTACCAAAGTTTAAAACGATTACCCATCAACGAGGCTGACGTCGTAGAAGTCCTGCTGGTTCGGGCCCTGCCCAGCAGTGATCTCGGCCAGGGTCGCCGGTGGGGTCCCCCCGACGCCGTTGCAGTACAGGGCCCCCCCGCAGTCGCCGGTGTCGCACCGTCCCCGCCCGGCGCCGTCGAACTTGCACCCCTGCCGGCCCCACACCCGACCCGACCACCCTTCGGGCAGCCGGAGCGAGCTCGCCTGGTTCGGGAGCAGCCGGAACCCTCCGCGGGCGACGATGGGCTTCCCGGCGCTGGGCTGGATCCCCGGCCACACTGCCTCCGGGCATTTGTTGTAGAGCGCGAGGGTGGTGGCGTCGGCGGTGAGGGTCAGGAGAGGGAGGAGGAGGAGGAGGAGGAGGGAGGGATTCATTGTCTCCGAGCGCGGTGGGGTTCGACGGAGGAGCTGAGTGATCGAGGGTAACTTTATAGGTCGGAACCTTCCGGACACTAA

>UN00842

GGTTTATTTGTTTGTTAGGTTGTTTGTCTATTCTCAGCGGAGCTCTTGGAGACGCTTGATCCAGAAAAGGTGAGGCCTCTGGAGAGGGAGTTTAAGAAGCAAAAAGTGCTGGAGATCAAGAACAGAATTCGCAGGATAGACTTAATGAAGCAAGCCCTTCAGCGGGTACTGGAGGATGTAGAGAACATGGCGGATTGTATCTGGGGAGTCTGATGGAATTACACGGACAGGGTACCGAGTTGCATGGCAAGATGTTCTTGGTTGTCGCCTGCTTGTGGATGAACTAGTGTAGGTTTGTTCGTATAGAACCTCTAAATATGCACTTCCTTGTAGGATTTTGTTCAGTGGTGTTGCTAGTTCTGGATTTTTTTACCATTTTTTAAAAATATTTCGAACTCCTAACTTTTTAATGTTTACTCATTTTTATATTTCTGATTCCCAACTTTTGTGGAATTTTGTTCAGTGGTGTTACTGATTGTTTTGTTCAGTGGTGTTACTAGTTTTTGTATAACATTACCAAAATGTCAAATCATGG

>UN00843

GTTCCTACAGAAGTGGTGAAACTGAAGACACCTTCATTGCTGATCTCTCTGTTGGTTTGTCAACAGGCCAGATCAAGACTGGAGCTCCATGCAGATCTGAACGACTCGCCAAGTACAATCAGCTGCTGAGGATCGAGGAAGAGCTTGGTGCTTCTGCAGTGTATGCTGGAGCCAAGTTCAGAGCTCCCGTTGAGCCATACTAGACCTTGATATAGAGCACAGATGCACCCCTGTGCCATAAGTTGCATAATTTTGGTTCCCTGGCTGTTTGCTTCGTTTTTCTCTGTCTCGAAACTGTGAGCCTCTTTGCTGTTGTGGGCGAAGAATAGAAGAAATAAAATAAATAAGGTTTTTAAGTTTTAAAGAAACT

>UN00844

AGTTCTTTAACTCCTTTTAAACTTAGTAGGCGTTACTTCAAAATTAATATGTATGCGTTTCTGTCTGTCTCTGACAAAGCTTTATTAACTCGCCATGCACAAATAAATAGCTGAGATACAATAGATAGCTCTCAACCCACACCGAAGTCATGTTTTCATTGATATTACAAAACTTTACTGCACGCTTAAGACCGATTTATTTCTATACAAATCAGTAGGATTACATTTTCGGTAATATAGTGGAATCAGCTACAACGCCAGATTCAGCCTCAAGATATCTGGTTAACAGCGTGGTAGTGCTGAAATTAAAACCTTGAACAGGCTCTTTCTTAGTGCGAGATAAGTTCAGTTGCTTTCTAACTTGGGAGGCTAATGACTTGCCCAACTCGACACCCCACTGATCGAAAGAATTGATCCCCCATATGAAACCCTCAACAGCAATTCTGTGACTCATAAATTGCTAACAGCTGTCCAATGTTATAAGCACTTAAAGCAGGCAGTAGAAGGCTCAAGGATGGCCGATTACCAGAAAAGGTCTTGTGAGGAATAAGGTGGTTCGGAACTTTCTCACTTAGCAATTGTTCAGGAGTCTTCCCATAAGCAAGAGCATCTGGCTGTGCAAAGAAGTTTGACATGAGTTCATCATGGTTGCTCACAACTTCCCCTTTCAAGTACACGGGTTGCTGAGTTTTCATGACACCAATAAAATCACAAGGAATAATCCTTCCTTGATGAATTAGCTGGTAGAAACTATGCTGGCCATTTGTTCCCGGTTCTCCAAAATCTATTTCACCAGCCTCAAAAGGAAGTGGAACGCCATCAATGGATACACCCTTTCCATTACTCTCCATGCTAACCTGTTGGATGTGCGGAGCAAACTTCTCAAGAGCCTGGGAGTACGGTAGTATGGCTCTTGCAGGATATCCAAAAAAGGAAACATTCCACACGCTCAACAGGCCAAGAAGTACAGGAATGTTTTTCTCAAATGGAGTCGACTTAAAATGATTGTACAA

>UN00845

TAGTTACTACTTTGTAGGTTTGTAGGGTACCTAGTCTACCGAATTATAATCCAGTAAAGGTTGTGGCTAAAACCCTCGTTGAGCTGCTGGAATATCTTGGCATACTGCAGAGGGATCCGAGTACTATCGTTGCTCCTAAGGCACAGGCTGTGGCTGAGTGATGTCCTGTGGGTTTTAATAATAATGCTATATATATATGGAATAAATGTGAGCCTGCTAAGGTTTCCATGGGTTCGGAGTGATGTTGTGCTCCGGGTGAAGTCTGAAGTCTTTTTCTGAGTTACGTACTGTAATGGGCAACTACTCTTTTGAATGAATAAATAAAAGTTAATAGACTTAGACAAGGTAACTTAAAATTTA

>UN00846

GGATTACGAGCCCTAAACTTCCACGATTCCAAGGCGATTTCAGAACGCTAGGGGATCGTCTATCAGACGCTCGTAAATTTGGGGGAGATTCGTGAATAGAAATCTGAAAGCATGGAGCACCACGAGGAGACAGGGTGCCAAGCTCCTCCAGAGGGCCCAATCCTCTGCATCAATAACTGTGGCTTCTTTGGGAGTGCAGCTACAATGAATATGTGCTCCAAGTGCCACAAGGATCTTGTCTTGAAACAAGAACAGGCTAAGCTGGCGGCAGCGTCCATTGGCAGCATTGTGAATGGAAGTGGAAGCACCAGTGGCAAAGAACCTGTCATTTCCAGCAACGCCGATGTAGCAGTTGCTGCAGTGGAGCCAAAAATCATTTCACAGCCATCTGATGCCTCTGTGTCAAGTGAAAGCAATGAACCAAAGGCAAACAAAGGTCCTACTAGATGCAACACCTGTAACAAAAGAGTAGGCCTGACCGGCTTCAATTGCCGGTGTGGAAACATCTTTTGTGCGAGCCATCGATACTCAGACAAGCATAACTGCCCATTTGATTACCAGACAGCTGCTAGGGATGCGATTGCTAAGGCCAACCCTGTTGTCAAAGCCGAGAAGCTTGACAAGATATAGAGAAGATTATTAAGAAATGTCTGGAGGCATGATGATGATGCTTGAAAGGCATCTTTGCAGCCTCAAAGAACTCTTTATCTTGTTGATTGGGAAAAAAGGTTTGCATGTCCTGTCTAGTGTGTAAACTTTTAATTTGGTGATGGTTTGATCGACACTTATGTGCTTATGATTTGGTTAGCTTTGTAATCTACTATCTGTCATAGTTCATCTTTTACATCTTATCTGTTGTTTTGTAGCATTAGCATATGAGATGGTGTGAAAACTGTGCATCTCATCAGTTATCATGTTAATTTAGACTTTGTTGCTTACT

>UN00847

CAAAAAGTCTGGAAAAATGTGAACAAGGAACATCCTAGGAGAGGGTTAATTAAAATCAACATTCATAGTACGGCAAAAACACTGAAATAACTTATCATATCTTTTCCGAACCAAGGTCGTTACACACTAAACCGCGTTCAACCGGAATTGGTAACACAACATTACATTACTACCGGTGTAGTAAACACCGTTGGCAGACTGCGGCCAAAGCAAAGAGTTCCAAGACACGCATTTATATGTTGTGCTTCCTCCTAACCTTCCCTTTTCCTCTTTTGCGACGCTTTTCTTTCTTCAGCTGCAGTTTCATCTTCAATTTCTTCTTCCCAATACCACCTACAGGCTTCAATAATGATTTGGATTGATCGTTTCCTCCATCGGCCATTTCTACATCCATTGAGTTGGACGAGGTTTCGTTGGTGATGAGCTCCATGGAATCAGAGCTGGGCTTGGGCATGCGAACGGGGAGCTTTGGGGCGTTGAGGGCGGCCTCCTGGGCCTTGAGCTTGGCATCCTCCTTCTTGTCGTAGAAGGGCTCTGCGATCTCCCTCCTCAGGGTTCGAAGCCTCTTCTTCCTCTTCGATCTCATCGATTTCGCCATTAGGGTTTCTTAGGGTTTTTTCGTTTTTTTTTTTTTTTTTGGGGGGGGGTTTCTAGGGTTTTGTTAAC

>UN00848

TAAGAACTTTTAAAAATTTAACAAAAATTACCCAATTAGAAACAAAGACAAAATTCAATCATCCTGCTTCGCAACTCCAAACCCTAACTTCAAATAACTCGTACAATAAGGCAGACAAACATAAAACATATTCAAGGAGTACTCTTTAAACCTAAAAGTAACAAAACAAGGACAAAATGGCATCACAGCCGAAATACAAACTAAATCAGCAATAGATAACGATGTGTCTTCTAGGCGGCGGTCTGCTTCTTGAGTTTTCCAAGCTCTTGCCTGACAGCAGCTCCCCTCTGTCAAAGTCGTTCAAGGATGCTCTTCTCTTCTGCACAATCAACTTCCTTCCCCACGAGCTGTTTTCCCACTTGTTCTTCACATCAGCAGCTTCCATTG

>UN00849

AAAAACCTTATTAGGTTTTCTAAACGTTCTATTCAAGGAAACAGCAGATGATAATCTTTAGCCCATATAATGGGCCACACATAAATTCCCATAAACTAACCAAATATCAAAATACTACAAAACTGTTCAAAATAACTAGGACACAAGCACCGCTGAGAAATAAATGGGACTCCTTGGAAAACGGATGGAGGAAACTACAGCCTCCTTCCTCTTCTCCCACCCTTTCTCCTGGTGCTGTCAGTGGGAATTGGCGTCACATCCTCAATGCGACCAATTTTCATCCCAGAACGAGCAAGTGCCCTAGAAGTAGAACAAAGAAAATTAAAGGGAAAAC

>UN00850

GCCGTGGATGTAGACGAGGATCAGTAGCTTCTGGGTGGTTGTCGCGGTTTTGGGGAGGTAGAGGCGGGCGGAGACGTCGGAGTTGATGGCGACGTCTTTGGAGGTGACCCCGGTGGAGGGGTCGAGGCCGGCCGGGAGGGTGTCGGTGCCCACGAGGCGGTGGACGTGGCCGTCCTTGTAGACGCGGAGGAACGGGAGGAACTCGGTGAGGATTTCAGAGTTGTCATCCATTTTTGGGCTGCAACGGTGGAGGAGGAGGGAGAGCGGAGTGGAGATATTTGGGCGACCGCCCCCACCGCGACCGAGTCGGTCCAGTGCTTCGGCCGCAAGAAGAACGCGGTGGCCGTGACCTACTGCAAGCGCGGGCGCGGCCTGATCAAGGTGAACGGGTCCCCGATCGAGCTGGTGAAGCCGGAGATCCTCCGGTACAAGGCGTTCGAGCCGATCCTCCTCCTCGGCCGCCACCGCTTCGCCGGCGTGGACATGCGCATCCGCGTCCGGGGCGGCGGACACACCTCCCAGATCTACGCCATCCGCCAGAGCATCGCCAAGGCCCTCGTCGCCTTCTACCAGAAGTACGTCGACGAGCAGGCCAAGAAGGAGATCAAGGACATCCTCGTCGGCTACGACCGGACCCTGCTTGTCGCTGATCCGAGGCGGTGCGAGCCCAAGAAGTTTGGCGGCCGCGGAGCTCGCGCCAGGTTCCAGAAGTCCTACCGTTGAGATTATTTGGGGTCGGTATCGTTCCCGCCTCCTTATCGTCTCTTTCGAGCTTTTGGGATTATATATGAGACTGTTATGATCGTTTGGTTTAAGACTGTTATAGGCATGTTAGGGTTTGTTTTGCTATTAAGACTGCTGGTGTCATTTCCGGTTCTTTGAAGGTTGTCGTCTTAATTTTATGCGTTTCCTTTTTAATATCTGACAGCCGTCGTTGTCTTAATTTTAATAGACTTTTTAGAATTACTACTTAGTTTAAAGTTAAAT

>UN00851

GGTACCAAAAAAATTACTTTTACTCTCATAAATGGTGCATAGTGAAATATACATCATACTCATGCAGGCTAGCATTGTTCAATGTCATATATAAAGAAAACAAAATTATATTATCCAAGGAGTACACTCAGAAATGACAGAAGACATCGAGATTAAAGACCATGAGGAAGGTGCACAAATTCAACATCCAAGTTGGTGAACAATAATTTAACAGAAGAAGAGAAGAAGGCATCCATTACTGATTCAGTGCCTCATCAACGAGTCTTCTTCTTGCCAGCAACAGTGACCTTTTTGCCCTTCAGAGTCCTGCAGTTGTTCTTTGTGCGCTGACCACGACATGGTAGACCCATTTCATGCCTAATACCCCCGAAATAAAGAAACAAAATAAAAGTAAAATAAAACTAAAACC

>UN00852

AGTTACGGTACCTAACACCTTCAATACATTAACTAATTTGCAAACAAATTGTGCCAATCAATGACAATATTATAGCTAGCTCATGAATACGAAAGTTCAAGATTATTTTATTAAACCGGTGGATCGACGAAACTGATAAAATTTCAGAGAGCTTGGGAGCCAGGGTAGCCATGGGAGCGGTTGGATGTAGGGCTCATTTGCCATATCTTCACACTTTTGGCGATCACAGTGGCTCCGGTCGCGTTATTGAAGAGAAAGACCTTGGCATCCTCGTAGATTGCTTGCGTCGGATAAACACGAGATGTGGTGCTCGCTCTCCCCCTTGAGCATAGCTCTCAACAATAGGAAAGTAAGAAAATAAA

>UN00853

AGTAAGAAATTTATTAGCAGCTTGAGCTGTGCCTAATTTGAAAGAGGGAGCTAATTTTTTAGGCAGCTCAAGGCCACTAGAAGCTACATCAAAGAAAAGATGGAACAAAGAAAAAATCCCCTCTCAAGCACACAACAGCTTGAAAACAAACAATCTTTTCGCTGGATATAGACTCTGCATTTCTCTGGTACACCAGTTACACTCCATTTACTTAATTTGCAGCAATCAACTCCAACTAATACAACATGAGCTACAACAACAAGCACTGCTGTTTCAGTACTTAGTGGTCTCATAACAAAGAGCAACTACAGTACGCTCCAAATTTGCAGCATTCTTATCCGAATGAAGAAAAACTAGTGACGCTTCTTTCCCGTTGCGATCTCCCAAGCTTGTAGGATATGATTCACAATCTCCTCTACCCCAACTCCATGCTTCACCTGAGCAAACACAAAAGGCCCTCCATCCCGCATCCGAAGAGCATCACGTTCCATGACAGCCAAGTCAGCTCCAACAGCTGGTGCCAGGTCTGTCTTATTTATTACCAGGAGATCTGCTTGGGTAATTCCAGGACCTCCTTTTCTTGGTATTTTGTCGCCACCAGAAACATCAATTATGTAAATTATATAATCTGCCAACTCTCTGCTGAAATTAGCAGCTAAGTTATCTCCTCCAGATTCACAAAGCAGTAGATCTGCTTTGTACAAGTTAGACAGCTCTTCAAGAGGACCCAGGTTAATACTTATATCTTCACGTATAGCTGCATGTGGACACCCTCCTGTTTCAACAGCACGGATTCTTTCTTCAGGAAGTGCTCCATGCTTCACTAAGAATTCACCATCCTCCTTCGTAAATATGTCATTTGTTACCGCCGCGAGACTGTACTTATCTCGCAAGAACCGGCACAACGCCAGCATCAGAGCGGTCTTCCCAGTGCCGACCGGGCCGCCGATGCCGACGGTGAAGGCCCGCTCACGGAAGTCTCTAGAAAGGATAGCTTGGGCCCGGGACGGGAAGCTGCCCCTCCGGCGAGCCCCCGTCCTACCTCACTGGCGAGTTC

>UN00854

AAGTTTAGTTTCCTTTGTTCGGTTCTAGTCTTGTAAGTCTTGAGCAAGTCCCAACTGATCAAGAACCAGCAGCAGCAGCAGAAGAGACCGCCGCCAAGACCGAAGAAGTGCCCGCAGCAGCAGCGGAAGCAGCAGTAGAAGAAGAACCGAAGGCCGTCGAGGAGCCCAAAGAAGAAGCTCCAGCTGCAGCAGAAGTCGCTGCTGAACCTGCAGCGGAGGCTGTTGCTGCACCTGAGGTGGAGGAAGCCAAGAATGATGCTTAGACGAATGTCTGTTGTGTTTTAAATTATTATTAGTACTACTACTACGTACTACTACTGTTACTATCATCATCATCATCATTATTATGAGGTAATAAGGAGTAATTAGTAGTATTGGTTTATTTGTAGTAATTTGAGAGAGCGTGAGGTTGAATTAGTTTTACTAAGTTTAGTAACTTTAAAGTTAACCTTTTTTT

>UN00855

AAAAGTTTTCACTTGCTCTTTAGAGCATAAAATCTAATAAAAGTTTACAACCTAACAATAAGAGCTTATAAAATTTGAAGTACCGAATGCAAAAGCATTTCCACAGCAAAATATATGACCAATATAAGATGGCCAGAACTAGAACAAAATGTAACAATCCCAATTTTAAGTTCATGATATAACTTCAGTAGAACCCAGCAAATTAGAGAAAACTTAGTCGACCTCCTCGATCTTAGGCCCAGCACCGCTGCCACCAGCAGGAGCAGGACCATCCTCATCCATACCAGCCCCCATATCAGCACCAGCTCCCTGATACATCTTGGCGATAATGGGGTTGCAGATTCCCTCGAGCTCCTTCATCTTGTCCTCGAACTCGTCAGCCTCTCCCAACTGATTCGCGTCAAGCCAATTGATGGCCTCGTCGATCGAATCCTCAATCCTCTTCTTATCGTCCGCAGCCAACTTGGAAGCGATCTTCTCATCCTTGATTGTGTTCCTCATGTTATAAGCGTAGTTCTCAAGTGCATTCTTGGCCTCCACCTTCTTCTTGTGCTCCTCGTCCTCGGACTTGTACTTCTCCGCCTCTTGACAACCAATTTTACTACAAATTTACAATAAC

>UN00856

CGATTTTATTTTTTTACCGGTAATCTTGTAAGTACCGTCTCTTAGGAAACATACACTCGCACAAACTACCTGTTATTGTTTTATTTAATTTTCAATGTACGTTTCAAATCTTCTGCTGTTTTACCAATCCTTGATGCAACTTATATTATACAGGTGAGGCTGGGGAAAAATGGAGTTGAGGAGGTACTCGGGTTGGGCCCCCTGTCAGACTTTGAGAAGCAGGGTTTGGAAAACCTCAAGCCTGAGCTCAAATCCTCTATCGAGAAGGGCATCAAGTTTGCCAAGGAGAACTGATCAAATTAGCTAAACCAATTAGGTTCCTTGAAAATCTTAGTTTTGCATTTGTATGTTTTGAGGGGTGATTCACCCATCACTTGTCTGTTAAAACTACAGAGATTTTTGCAGTCCGGCTGTTTGGTTCCAGAACTGTATCAATAAAATGTATGCAATTTGTTTAAGAAGGAAACAACTAAGAAACGTTTAACTAGGGTAG

>UN00857

TACTTAATACCCCTACTTACCTACTACGTCCTTTTAGGGTTTCCGATCCGTTTCGTTCCTTTCTCCGATGGCGGCTTCCGATGTCGAGTACCGCTGCTTCGTCGGCGGCCTGGCTTGGGCCACTGATGACCAGTCACTCGAGAAGGCCTTCAGCCAGTACGGAGAGATCGTCGAATCCAAGATCATTAACGATCGTGAAACTGGGAGATCTAGAGGATTCGGATTCGTTACCTTCAGCAACGAGCAGGCGATGAGGGACGCGATCGAGGGGATGAACGGACAGAACCTTGACGGTAGGAGCATCACCGTCAACGAGGCGGCTATAACCGTGGAGGAGGCGGCCGTTACGGCGGTGGGTCTGTAAGGGTTAGGTTAGGTAA

>UN00858

AATTATATAACTAAGTCAAAATTCAGAATATCGTTGAGGATGTCAATAACAGTCTTCTGTTTATCACCAAAAGCAGGCCATACTAACGACGACAAGGAAATAACAGAGTTGGATGCAAATAAAGCAAATAAAACCAGTTCATGAAACCCCATAGCAAAAGAACTAAACTCTATGACATCTTCACTCATCATCTTCATCCTCATCCTCATCATCATCATCACCACCAGCACCAGAGTTGAGAGCATTCAATCGTTCAATGAGCTTAGCCTTTCTCTCCTCGTATGTAAGCTTCTTTAAGTTGTTATTCTTCTTTAGTTAGTAACTTTAAACCTTTTTAAAGTTAAA

>UN00859

AGTTCTTACTTAAAACTAAAGTTCGTAGTTTTAGTTTCCGTTCAAGAAAGAGGTAACACAATCCTTGCAAGCGTATCTGATGTATACAAGAGCTTGTTACACAGATCGGAAACATTTTATTTAGCTTTGTAGCTTCTTCCACCCTACATCCTCTCCCTCCTTATTTATAATTAAAACAGGGGCTACACCGCCGAAACGTAAATTAAAGGCACATAAGGCCACACAGTTTCTCAACTAAGAGTGGCTAAACTCTTCAAATCAAGCACAAAACAAGAAATTCACCCGAAAAATCAAGTTCAGACTCTAGCAATAAACTTAACCAGTGTTCTGCATACCTGCAGCAATACCCTTCATGGTCAAAATGAGAGTATCCTCAAGCCCAGGAGCATATTCACTGGTGGGGTTCAGCTTAACCAATTCAGCAGCTGGCTTGCTTGAAACAGAGTACTCCCTTGATAAATGGGGTCTCGGTTTCACTTGGAAACTCGGGTCTCTGATCCTCTTCAGCGTGTAAGCTTGACAGACGTTGAGTGTTGTTATAGTAAAGAAGTACAACGAAAAGAACTTAAAGAAAAG

>UN00860

ACTAAAACTTAGTAAAAAATAATTATTTAACCCCGCCCTCACGGTCAACGGAGCCCACCTTTCTCCGTGACCCTTGATCCTTCTGTCCACAGTTTGGTACAGTGCAAGGAGCGTGCTTTAAGATACAAGAATGATAAGAACAAAGCAGAAATAGATTTGAGAACTTCAATGGTCTAAATTAGCTTCCACAATTTCACCTCTCAATTCTCCACACGGCACTTGCATGCTATCATGCCTCTTCGAAAATGAAAAAGTTAAAAGAGGGAAAAGAACCAACACAAACAAACCACATGAAAAGATGCAAAGAGCTACGTCAAAAGAGACCACAACGACGACAGACACCAACATAGCCAAAAACCCTTCATGGCTTGATCCCCATTTGCCAAGACATGTGGAATTTCTTCCCCAGTGGCAACTCAAGCCCTCCAACCTCCACCACCATCCCCTTCCTCTTATTCCCTCCCTCCAACTTCTCCTCATCCTTCACGCTCTCCCCCTCACCAACATAAACCCTACTCATCTCCTTCATCACCTCCCCATCCACTTCCATTGTCAGTCCACTTCCCACTCCCCCCAACCCCAAAACCCTCACCTTCTCGATCACCAAACCCTTCTACCAATAACTAGAAACTTAA

>UN00861

GTATAATAGCTTCAACTTATAATAAGCACAAATCCAATAATATCAAACTGATAACATTATTATTTACAAAGTACTAACTGATAACAAATCCAATAATACGTTCTCACACCCTACTTAGCACAAATCCAATAAAAATACCAACTAAAAGAACAAATATAATAAGCTTCAACAAATCCATGATATCAATCTCAATCTTTCTCTTTTTCTGCACTTCTCCACCTTGACACGGCTCTTGAAAATTGCGGCGTCGCGCTCCAATGCTTCATCTGACCTTCAAACTCCTCTTCATCTACAAAGTACATGACGATTGCTTGAAGATGACTCCATGCTACACGGCTACGTCGGCGTCGAGAGAGAGAGAGGAGGAAGCGTCTTCGACGCGGCTTCGGCGTCGAAGACTCGAGAACTCGTGAGCGGATTTGGGGAAGCGGCGGCTCTGTTGCAGGAGGAGGGCTGATGTCGTGGTGTCTTTGGGGAACGGCGGAGGAGAGCTGATCTCGTCGGAGGAGAAAGCAGCGGCGTCGAAGGCTCGAGATCTCGTGGTGTCGAGGGCTGATGTAGTCGGAGGAGAGCTGATCTCGTGCGCTGAGGAGAAAGCAGCGGCGTCGAAGGCTCGAGATCTCGTGGTGTCGAGGGCTGATGTCGTCGGAGGAGAGCTGATCTCGTCGGAGGAGAAAGCAGCGGACTTGGGGATCTCGTCGGAGCTGATCTCGTCGGAGGAGAAAGAAGCTGATCTCGTCGGAATTGGAAATGTAGGTGAGAGAGCGGATTCGGGATTTGGGGAAATTTTTGAAGAGGAGATTTCTCTATTTTGGGGGGGAGAGAGAGAGGTCACGGGGCAATAAATTTCA

>UN00862

AGGGTTCCCTTATTCTATTAAAGTATTTTATTTATTTCCTAATTAATAGGAATGGAGTTGAGTATTGCATTAAAAGTATGCATAAAGCACTGGCATCTCACAAAATGGTGGGTCATCACGCAAAGAAAGAACGTCAGAAATGCAGACAAAGTAGATCAGCATTACACCAAACTCAACTAAACTATTGTCCATACCAACAGTAGGTGAACAATATACTATAATCTAACAAAACCCATAAAGCAGTCTCCCAAATATTCTCAATCATCAGCATACATATATAAGCCAATAACAAACGATGAAAACAAGCCGACGCCTCGGCATCAGATTTAGATGATACCGATTTTGTTGGCGACATCAAGAGCATCGTAATCTGGTGTCAGCCTCACATATGCCTTCTTAGTACCATCAGGCCTGATCAGTGTATTAACCTTCTTAGTCTGGATATCATACATTTTACTTCAACTAGACCGACCTTAGAAATAAACTTTTTAAACTTAAAA

>UN00863

TGGTGTTTGTGCGTGTATACCATATATATGGCTCTAAGAGGATGATGAGGAAGAAGGTGAAGAAGGAGAAGAAGAGGGCTTGAGTAAATGTATAGGGAATGAGTTGAAGAAGCTTCTGCAGTGTCATAGTCATTGTAAATAATTTATGTAAAAGTTTATAAATAATTTATGTATCTCTCTACTATTTTGGTGAATGTAGAATGTATTATTTGAATATAATGAGATTTGATATTGGAAAT

>UN00864

GTTCTAGTTCCCTACGTAGTAACCGGTTCACTCTTCCCTCTTCTCCTTCTCTCACTCTGAAATGTGGAAAAAGGGCAAGAAGAGAACCGACGAGGAGGCCGCCGGAGGCGACTCCGACGGCGCTCCGCCGTCGAAGAAGCTTGCGAAGGGCGACTCCGAAGAAGATGGCATCGTCGTCTGCGAGTTGTCGAAGAACCGGAGGGTTTCAGTCCGGAGCTGGCAAGGGAAAATCGTGGTTGATATCAGGGAGTTTTATGTTAAGGATGGAAAGACGCTCCCAGGGAAAAAAGGCATCTCACTTTCCATGGATCAGTGGAAAATACTTCGGGAGCATATCAATGATATCAATGAAGACTAGTAGGGTGAGAATGCATAGTTTCGAAATGGTTCCTACTGATCCCTTTTGTCCAGTCACTGATGAATCAGACGTTGTGTTCACTGATCTAATGTTTTGTAATTAAAGATGTCGAAAGGCTCTGTCAGTTATGTTGAACTAGATGAATGTGGTTAACTATCTCAAATATGCTGGTACATTTGACTGATTAGGCCCGTTTCTTATGTTCTAGCGAAAGTATCGAGTCCTGGCTGAGAAGGACTTAACCATTTCCTTTGTTTGATACTGTTAATGCGTTAACAAATTCATGGTAGTACTT

>UN00865

GTTTTTTTAGGTTTAGTTTACTTAAAGTATTCCTTGGTACTAGAAACCATTCACATTCAAGATTGGCTGTGGATCAGTTATAAAAGGCTGGGATGAAGGCGTGCTGGACATGCAAGTTGGAGAAGTCGCTCGCCTTCAGTGCACCCCGGACTATGGCTATGGAGCTAGTGGATTTCCAGCATGGGGAATACAGCCTAACTCTGTCTTGGTCTTTGAGATCGAAGTGCTCAGTGCACAGTAACTAGAAATGAGGAACTTTTAGAGCCTCTGAAGTCATGAACTAAACTGGAACACTTTTAATAGTGCTGCTGGAAGTAATATGGTTTCATGAAAAACTAAAAAGGACTTAGAACTAAAACC

>UN00866

TAAAGGTATTCTATTTCTTCTCTCTTGCTCTTCATCTCCGTTGAAAATCCGTCGCCGGTGATCGTATCGCCTTCGATAAGAAGTAATCATGGCTGGAGTGGGTCCGATCGCTCAGGACTGGGAACCGGTGGTGGTGCGCAAGAAAGCCCCCAACGCCGCCGCCAAGAAGGACGAGAAGGCCGTCAACGCCGCCAGGCGCAGCGGCGCTGAGATCGAGACCATCAGAAAATCTGTTGCTGGTACAAACAAAGCTGCTT

>UN00867

GTTTTTTAACGTTTTAGTTAAATTCCTGTGAAAAACACGATCGCCTGCCAGTGGAAGGCTTTCTGCGTTCAGTCAATCTACGCAGAGTGAATCGGTCCCTAAGGAACCCCCGAAAGGGCTGCCGTCCGATGGGTACACGAAAGTGACGAAGTTGCTTTGACTACTGAACCATGCCTGTCTGTTGGAGCGAATTGGATGATCGGGCCGAGGGCTGCCCCCTCTTCCCCTCGCTCTCCTTTCCCTAAGATGCACCTTGAGTCATCAAAGCCTTAACTAATTCAGAGGGGCTCGGCTGGCCCGGTCGCCCTACGCTACTGGCGCTTCCAAAGGCGAAGCTCTCGTCTTTGGCGACCAGCAAACGGAGGCTCTTCGACGACCTTTCGTTTTGAAGCAGGGGCGGCTTTTTTTTCTCTAAAGGCGAAGGCTCTGAAGTGGGCGAACACTCGGCCCAGCGGGCGAACATGCCGGGCTCTGCTCTCTCGCGACCCCTACTCTAATCAAATAAAGGCCCCTACTGAGAAAAAGGTGAGCACTGAAATGAGAAAGGAACGAGTGACGCAACACGACTTCGGTTCACACGTGAAAGTGCTTCGAAAGGTGTCCGCAGGTGCGCGGAGCCGGAGCCCGGCATGTTCGCCCGCTGGGCCGAGTGTTCGCCCACTTCAGAGCCTTCGCCTTTAGAGAAAAAAAAGCCGCCCCTGCTTCAAAACGAAAGGTACGTACGAAAGAAGACCTACCGTTTAGACTAGGTACGAACCAAAAA

>UN00868

GTTTACGTTAGTTCTTAAAGTACTTGTTGGTCCCTAGGACTGGGCGATAGAAGCCATGCCGACCTTTGTGTTTGTGAAGGAAGGTACCATCATCGACAAGGTTGTTGGTGCTAACAAGGTGGATTTGCCCAAGAAGATTGAAATGCACAAGTGATTCTTCCTCCTTCCGAGCTCCATGCTTATCAGATTGGAGCAGCGACGTGCGTGCTGTTATTAGGATATTGGACTTTCTATGCTCTTATGCTTTGCTAATAAGTGATGTAGTTTAGGCTTTCATGAGATCCAACCATTGAGCAGATACTGTGTGGCTAATGAATGTGAACTTGTTGCGCCTTATTTTGTTTGATTTGTTCATCAACTTTGTTCTGAATGGTTTCTATGGCCTTTGTGTAGATTTTTAATGCTCTTAAAGCTGTCGGAGATGAAAATATGGATTCTCATCACGAGCGTTATGTGGTGTCTTCATCTTAAAACTGCAGTTAGGATGTAACTGATTGGTAATAGTTTACTAGAAGAATTAACCCTACCGAACTTAAAATAACAAATTAAAGAAAAGGAAAACTTAAAAG

>UN00869

AAGTTTTAAAAACTTTTTGTTTTAAGTTTTACTTACGTTACGGTTCTAAGTATTTATATAGTAGCATCTAACAAGCATAATAACAATTAAGAGTTTTCAGCACATCGAAACAAGACAACAAACGTCTGAAGACTACAGACACCAACAGGAAACTAAATGCAGAGAAACATTCTGCAAATCAAAAGATTCGAAAACTGACTAATGCGTGTAATAACACTAGTCTCAAAGCCCCCTCAATTCTCACTAATATCGCTCACGATGTAACAGCTTCATAACCAGAATATCAATGTGTACTGACAATAAGACAACTACAACCTTAAGAAAGAACTTAAGTAAACTTAAACGAAAA

>UN00870

GGGTAAATCTCCGCCTAGTGGCTCACAGTTCCCAAAGTTACACAATCTATTTAACCATGTCAGCTAAAATGTGGGGTCCGGGTGCGGAACAAGGAGGAATACTACAGACATCTACGGGTAAACTTGACCTTAAAGAACCATACTATAGGCTCTCCCAACCACAACCTTATGCATGGGCTCAAGATCAGCAGCAACAGCAGCAGGCCCCTATGCCCTCACAGGGATTCTCCCCTCAAGCTCTGGAAGAAGGCGACGCCACCATATTGTTACAAATGCCCGAGAGCCCAGATAACTTACTAAACCGATAATCATCAATCTCGTATCTGTAAATTAGCCGTGTACTTCTGTCGATCCCTAAACTTACTCTACTCTGTGAACTCATACCTGCCTTCAAGCGCCTAATTAAAGAAACGCCATAAATGCATTTGGCTGCTGAGAGAAGTTCCTAATGGATGTGTAATATTTAAGTCGATGGAATACCAGTACCAGTGTTCCCAAATATTCTAGGTTAATTTATTGTGCTGAGTTCTTTGATGACAAATGATGGTATTATGATAAAAGGGTGCAGTTTTAGGAGAACTGTCCTCATTGTTCTTAAATATTGATAGGACTTGTAGTTTATTTATGTTATTGGCATCACCATTTTATGTAGCCCATAGTCTTGCCAAAGCTTACACAAGCAAGTCATCCCT

>UN00871

GTTCTAAAATTCTAGAAGTTTTATTGATCCTGTCGAAAGAAAATTGTTGGGGAAATTGCATGCATGCAAGAAATCATCCATTAAAGATGCTAGAAAAGGCAATATTGTTGAAGAAAGTGTAGACAGCGAAGAGGAGGATGATGATGATGAGCCTCAGAGCAGAACGAATGCTTTTGCCAAAAAGAGAGCATTGCCTCCAATCACAACTCCACAGTCGAAGAAACAAAAATGAAATGTTCAGACACTAGACAGTCTAATCCTAAATTATGTGTTCTTTTATTTCTCCACTTCGCACTAAATTTTACTAGCTCCGCTCATCCTAAGATTTTGTAGTTGATCAGCAATTAAACATGCAGAAGCTTTGTAAC

>UN00872

AACGAAAGAAAAGTAACAAGAATAGAATAGAATGATGACCAACATGACGAACTTTAGAGAGGACGTGCAAGAAGCACCGCAGTTATTTTTGGTAGCGAGTCGGTTAAAGATATTTCATCAGAGAGATGTTTAGGGGGTATAAAGAACTATATAGAGCCTGGAACCCTCTTCTCATAATTTTAGTGTTTTTAGATGTCTTGCAGAAAACATTTGTACCAGTGTTTTAGCTCTGATTGTGGGAACAAATACAATTTCAGGGAAGAAATAACAAAAGTTTTTCTTACTACTAACGT

>UN00873

CGTAGTCTATTCGGGGTAGTAAGTCTTGGTATCCGGCGGAGTTCGATGAGTGGATCAGGGAGGTCGACGTGGCGGCGGACGGGACGATCAAGTACGAGGATTTCATTGTGAGGATGGTGGCTAAGTGACGAAACCCTAGGATCTGAGATGAGATCGAATCAAGATTGAAGAATGTGACTCTTTAGAATTGGTGTTGGTGTGCTTAGGAGTTGATCTGCTTGTTTTATCGTCAAAACGTTTTTGACCTTTGCTTTTTCTATTTATTAATGTTGAAACCATCTGTAGCCTATTGGTACATTTGTTTTCTTTTGCCTCTTTTGAATTTATGGTGCATTATGGTTGTACCTTACTCTAATCCTTGGGGGTGTGAGCTAATGTATGAATCTATTGTTGTCTATATTTCTAATTTTTGTAAGTAAATTGTTGTACT

>UN00874

AGTCACAAAACACATCATTTCAGTCACCTACAGTTCTTCCCGAAGCCATTATTGACTTGATCAAAATCAATGTAAGCCAACATTATCTTGGCTTAAAAGAAAACAAAGATCATGACAGATATTCAATTTTCAAACAAGCATAACTGACATTAACAACTTGGTCAAAAGGTGCAAGATATCCACCCCAATCGCCGAATTGCTTGAGTCAAGCAAATTAATCTTTAAAAAATAAATAAAAAATAAATAAAATAAAAACAACCGGTTCATAATCCTTGTAGATAGATGTAAGGTTCATTATTAATGAGCAATATTTGGGGGAAAAGCAAGCCTCCACTCGGCAGTTACAGATTTGTTATCAATGAGCTTGGTGGATCAGTCATCCCAGCTTGTCAACTACAAACAGTGAGAGAGCTAGAGACTCGCATCCAGTTGGCAGTTACAGATGGATTCCTGTTCCAACATATAAATTTGCATTAGAAAGAATTTAGTCTCACCTCACATTTTGAATTTATAACCTTTGATCCGTTACCGGTTAGCAATCAGGAGACTTGTTGATTTTCCAACTTTTCACTTCCAACCTCCTCTGTTCCACTGGAATGATCTGTTATCCTAACATCATCGTCAGTTAAG

>UN00875

AACTTTTAGTTTAATTTAATTTGGTTCTTAATTAACTAGTACTTCAGAGTATCAACAAAACAGAATGAAGGCTAGCTGGCAAAAATAAGATCTTAATTATTACACATAGCGTTCTGATACCAATTTTGTTTCATTCCCCAAACTGACATCCCATAATTTGCCGAGAGAAGCAACAAGCATGAAGACCCTTCACCTGATATCGGATTCATTACCTTAAGCTTCAGCAAACCTTTGAGATGAGACAAGCACTCCTTACTTGAATTAGCTAAGAATCCACCAAGGGTTTAGAAAGAAACGGCAAAGAAAGATCATCAACAAAACTAATCCAAATGCAAGACAGGGTTTCAGTTCCCAAAACCTGTGAGCGAAACCAGAGAGAGAGGATCGGGACTTGAAACCAGAGAGAGAGGCGGCGGCAGAAGGGGGAAGGTAGCGACGGGGGAGGTCGGTCGGTCGGGGGGTCGGGGGTCGGTCGGTTCGGTTCGGGTT

>UN00876

GTCGGCGGGCATCGGCTGGCGACCGACGGCGGCGATGGGCGGCAGCAAACAACCCCGACGTTGACCAGCGTCTCGTCTCCCCATCGCACGCTACATGTTCGACGATATGTCAACGCACATCCAGTGGAAGAACCGCCCCGCCGTAGGCTCGCAACGTCAACGTCGATTCGTTCGACAATACCCGAGGTAACGAGCCAGGGGCCCGGAGAGTCGCAGGTTGACGGCGATGCACTTTAGCTTCAGCCATCTATTTTAGTTATATTTTCTTTGAGCTCCACTGTAGACTTATGTTTAGACGTTTGAGAGTTTTATTCTAGAAGTTGTAAAGGCTATGTGCCTTATTTTCTTTTGTCAGACATTTTGTATTTTGAGATTTGATGAGAACTCCAGTTTATTTATTTCAGACATTTATTTGTACTCTAACATATTAGATGGTGAAATATTAATTTGATCTCTATTATTTCATAC

>UN00877

TATATGTTAAGCCACCTAACAAGACTCAACAATTGAAGATGCATCATGAAATTATTGAAGATGCATCATGAACAATAAGAGCACCATACAGGAAATTATTGAAGATGCATCATGAAATGGAAATTGTAGTTCAACCAATGAACACGTGAGAGTTTCCAAGCTCCAAAGAGGCATCCAATGAGTTGAAACATAAAGGTAGTGAAATAAGCAAAATTGTCAAAGCAATTACATTACATAATATAGTGCAAATTGCAATTGTTCGCTAATGCAAAGAGTACAAAACAAATAACTAGTAGCCTAAATCCTCTCATTAAAACAGCTGATAATAACCCCACAAGAAAAATTCCTACTTAGATCTCTGCCTCATCTTTCGGCGCTTCCTCTTAAGTCTCCTCATGCGCTTCTTCTTCCAACTTAAGACCCTAACAAATAACAAAACGAAAAACGGAAAAAATAAAAACGAAAAAAATAAAAAA

>UN00878

TTTTCTTTTTCCGTTTTCTTTTGGTTTCCCCTTATTGGTTTCAAGAGTCCGACCAGCGATCCCCGCCTCCGCCTTCCGCCGCCCCCCTCGGCCTCGTCGTCGGCAACTACTGCCACGACGTCCTCTTCATCGACGGCCGCCCCTCGCCCGCTCCCTCGGCGGCGCCGCCTCCTTCATCTCCAACGTCGTCGACCCTCACCTCCCCGGCTCCTTCCTCTACGTATCCAAGGTGGGCCCCGACTTCTCCTTCCCCT

>UN00879

TTGAGAAGGCTGCAGCTCCTGAGGTAGACAAGGAAGCAGGAAGATGTCAAACCTGTTGAGGGTGACAAGGACAACAATGAGAATGTTGCTACTGAAGAAGAAGCTAAACCTGAAGATAAGGAGATGACTCTTGAGGAATATGAGAAAATTAGGGAGGAGAAAAGAAAAGCCCTACTTGCACTGAAGGCTGAAGAGAGAAAGGTTGTTATTGATAAGGAGCTGGAGTCAATGAAGCAACTTTCACTGAAGAAAGGGTCGGATGAAATTTTCATTAAATTGGTAAGCTACCTTTGAATTCTTTTTAGTTGATAAGTATTTGTTTTATTATCTCATGGTATCTGCTGCTTTTGCAATTAAACAATTGTCTTCCTAGGGTTCTGATAAGGAATCGAAAAAGAAAGAGACTGAGCGTGAAGAACGAGCCAAGAAGCATTTGAGCATCAATGAATTTCTGAAGCCAGCTGAAGGAGAAAGATACAACTCAGGTGGCCGTGGCCGAGGCAGAGGACGCGGTGATCGTGGCGGCAGAGGTGGCAGCTTGGCG

>UN00880

GTAAAGACTAGTTAGAAATAATTAATTTGTACTTGCCGCTCGCTCTTCAGTTGCTGCTCACTTGTGGCAAGACCCCACCCCCTGGCTACACTTGCACCATTTAAGAGGTGGACGCGAGGTTGTATTGATGGATGCATGGTAGCTAGATGATTGATGCATGAGACATGAGAGGACGCCGAGAGGAAAGTTTCTGGATTCATGTATGAGCGACGAGACATGAAAGGATGTCAAGTCCTTATTAATGATGTTAATTAATTTGCTAGCCTTGGTTTTCTATTTTTTCCATTGTGCTTTATTAGTTCAATTGTTTTCAAGGGGTTCAATTAATGTTATTGTGTTATGGTCATTGCTAAGACAAAGGAAAAATTGGTGGACTTTGTACTAGAAAT

>UN00881

GACAAATCATAAACTATATCTAATTAAATTGCAACATGTAAACAGTCGGATACATAATATGGATACCCATATGACCAATTAAATTAAGCTTTCATAGATCAAAAGCTAAGAAACCCTTATGTATACTAAAAAGAGGACAGAGCAACAAAAAGGCTATATAATCCCTTTCTTCACCTTTCCCCCCTCCACACCACAAACCCCAACAAAATAAAACCATAACCCATCACAGACCAAAACACGAACAAACAATAATCCTTTTTTCCAACATAGGAGACGAGGTATCAAAACCCATCTCTAAACTCCAAACTGATCTAATATACTAGCATTACCAAAACATTAAAAAAAAGGGAGATTTTTGAGGATCAAGATCCATTCACCATCACTTTGCCGAAGGGCATTCCCTCGCAAAGTGCCCCGCTTCTCCACAGTTGTAGCATCCACCGCCGCCACCGCCACCGCCGCCACCGCCGTAACGGCCGCCACCGCCTCCGCCTCCTTGAGTGCAGTCCCTCGCCATGTTGCCCAGTCTCACCACAGCTATAACAAGCCCCGCCGCCGCCTCCACCGCCGTAACTCCGACCGCCGCACGTATCCACCGCCGGATCCATAACCCCCGCCA

>UN00882

TTTTTTTAGTACCAACAAAAGAAAATCCTTTTTATTTTCACAGCTCCAGACACCATGCTCTCAGTCTTCCTTTTTATTTATTTGTTTTTTTAACATCCAGTTGTAAACATATTCTGCTTAGGGCTCAAATACCATGCTCTCAGCTTCCTTCTTTATCGACTGGAATAGCACTGAGGAAAGATATCGCTCTCCAAAGCTGGGGAAAACAACCACAATGAGTTTCCCTTCATTCTCTGGACGCTGTGCAATTCTAATGGCAGCAACAGCAGCAGCTCCAGATGATATTCCAACCAGCAACCCTTCTTTCAGGGCAAGAAGCTTTGCCATTTCAATAGCGTCATCACTTGAAACCTGAACTACTTCATCAATTAGATTAACATCCAAAACTCCAGGAATAAAACCAGACTACCTAAATTAACCT

>UN00883

CCTCCTTAATGGCGGTCGAAACGCTTCGCCTGAGAAAAGATTCCTTCGCCGTCGAGCCCCAAGCCCCGCCGCCGCCGCCGCAACCGCCGACGCCCAAGGAATCCCACTCCCACTACCGCGGCGTCCGCAAGCGCCCCTGGGGCCGCTTCGCCGCCGAGATCCGCGACCCGTGGAAGAAAACTCGCAAATGGCTCGGCACCTTCGACACCGCCGAGGAGGCTGCGAGAGCCTACGACGACGCCGCGAGAAGCCTCCGGGGCCCGAAGGCCAAGACGAACTTCGGATCCGAGCCCGATCGCGAGATCCGACCCGACTCCGCGTCCCACCGCCAGATCTGGTGGCGGGACGCCTCGATCGACGGCCGGGATCTGTTTCTTGGACCGCCGGGGAGCGGCGGGTCGGCGTTCAAGGGGTATCGGTTCGAGGCGGTGGCGGCGGCGCTCTCGACGGAGGAGGAG

>UN00884

GTACGACCTACGGGACAAAGACCCCAAGGAAGTTTGGTGGGCAGTTCTTGGTGCCGAGCTACCGAGGGTCGTCGTTTCTCGACCCAAAGGGCCGTAGGTGGGTCGACTGGGTTACGACAACGCGGTGGCATTGCCAGCTGGTGGACGAGGAGACGAGGAGGAGCTGAAGAAGGAGAACATAAAGGAAACGTCGTCGAGCGTTGGGAACATAAAGCTGAGCGTGACGAAGACGAAGCCGGAGACCGGGGAGCTCATCGGGGTGTTTGAGAGCGTGCAGCCTTCGGATACCGATCTGGGAGCCAAGGCGCCCAAGGATGTCAAGATTCAGGGGGTTTGGTACGCGCAGCTTGAATAGTAGAGGGGGTTTGTATCAACGGTGGGGGGTGTTTGTTTGTTAATTTGAGTAAATCTTGTTATTAATTGTAAACTTTAATTTTGGTTTATTGGGTCTATTCGTAGTTTACGTTA

>UN00885
[truncated: 18,756,732 more chars]
